# Supplementary material for: Synthesis and HPLC-ECD Study of Cytostatic Condensed O,N-Heterocycles Obtained from 3-Aminoflavanones
Source: Biomolecules. 2020 Oct 20;10(10):1462. doi: 10.3390/biom10101462 (PMC7593906; doi:10.3390/biom10101462)
Supplement: Supplementary file 1 [file biomolecules-10-01462-s001.zip › biomolecules-924992-final-supplementary materials/Biomolecules_SI_part_II_20201013_rev.pdf]

Supporting Information

(PART II.)

Synthesis and HPLC-ECD Study of Cytostatic Condensed O,N-Heterocycles Obtained from 3-Aminoflavanones

Ádám Szappanos [1], Attila Mándi [1], Katalin Gulácsi [1,†], Erika Lisztes [2], Balázs István Tóth [2], Tamás Bíró [3], Anita Kónya-Ábrahám [1], Attila Kiss-Szikszai [1], Attila Bényei [4], Sándor Antus [1], Tibor Kurtán [1,*]

[1] Department of Organic Chemistry, University of Debrecen, P. O. Box 400, 4002 Debrecen, Hungary Fax: +36-52-512-744 E-mail: kurtan.tibor@science.unideb.hu.

[2] Department of Physiology, University of Debrecen, Debrecen, Hungary

[3] Department of Immunology, University of Debrecen, Debrecen, Hungary

[4] Department og Physical Chemistry, University of Debrecen, Debrecen, Hungary

[†] deceased

* Correspondence: E-mail: kurtan.tibor@science.unideb.hu.; Fax: +36-52-512-744

Table of Contents

1 Antiproliferative activity experiments 6

Figure S262. Effect of synthesized compounds on the viable cell number of A2780 ovarian carcinoma (A-D). 6

Figure S263. Effect of synthesized compounds on the viable cell number of WM35 melanoma (E-H). 8

Figure S264. Concentration-dependent effect of *rac*-**19g** on the viability of A2780 cells. 10

Figure S265. Concentration-dependent effect of *rac*-**19g** on the viability of WM35 cells. 11

Figure S266. Concentration-dependent effect of *rac*-**19g** on the viability of HaCaT keratinocytes..........12

Figure S267. Concentration-dependent effect of *rac*-(4a*S**,5*R**,10b*R**)-**2b** on the viability of A2780 cells.......... 13

Figure S268. Concentration-dependent effect of *rac*-(4a*S**,5*R**,10b*R**)-**2b** on the viability of WM35 cells. 14

Figure S269. Concentration-dependent effect of *rac*-(4a*S**,5*R**,10b*R**)-**2b** on the viability of HaCaT keratinocytes……………………………………..15
Figure S270. Concentration-dependent effect of *rac*-(4a*S**,5*R**,10b*S**)-**2d** on the viability of A2780 cells. 16
Figure S271. Concentration-dependent effect of *rac*-(4a*S**,5*R**,10b*S**)-**2d** on the viability of WM35 cells.. 17
Figure S272. Concentration-dependent effect of *rac*-(4a*S**,5*R**,10b*S**)-**2d** on the viability of HaCaT keratinocytes……………………………………..18
Figure S273. Concentration-dependent effect of *rac*-**3e** on the viability of A2780 cells.. 19
Figure S274. Concentration-dependent effect of rac-**3e** on the viability of WM35 cells.. 20
Figure S275. Concentration-dependent effect of *rac*-**3e** on the viability of HaCaT keratinocytes………………………………………………………….21
Figure S276. Concentration-dependent effect of rac-**4b** on the viability of A2780 cells.. 22
Figure S277. Concentration-dependent effect of rac-**4b** on the viability of WM35 cells. 23
Figure S278. Concentration-dependent effect of *rac*-**4b** on the viability of HaCaT keratinocytes………………………………………………………….24
Figure S279. Concentration-dependent effect of *rac*-**4c** on the viability of A2780 cells.. 25
Figure S280. Concentration-dependent effect of *rac*-**4c** on the viability of WM35 cells. 26
Figure S281. Concentration-dependent effect of *rac*-**4c** on the viability of HaCaT keratinocytes………………………………………………………….27
Figure S282. Concentration-dependent effect of *rac*-**4g** on the viability of A2780 cells. 28
Figure S283. Concentration-dependent effect of *rac*-**4g** on the viability of WM35 cells. 29
Figure S284. Concentration-dependent effect of *rac*-**4g** on the viability of HaCaT keratinocytes………………………………………………………….30
2 Chiral HPLC-ECD analysis 31
Figure S285. HPLC-UV and –ECD traces of *rac*-**20a** on Chiralpak IA column with hexane/2-propanol 80:20 eluent monitored at 230 nm. 31
Figure S286. HPLC-ECD spectra of the first- [(4a*S*,5*R*,10b*R*), black] and second-eluting [(4a*R*,5*S*,10b*S*), red] enantiomers of *rac*-**20a**. 31
Figure S287. HPLC-UV and –ECD traces of *rac*-**20b** on Chiralpak IA column with hexane/2-propanol 80:20 eluent monitored at 230 nm. 32
Figure S288. HPLC-ECD spectra of the first- [(4a*S*,5*R*,10b*R*), black] and second-eluting [(4a*R*,5*S*,10b*S*), red] enantiomers of *rac*-**20b**. 32
Figure S289. HPLC-UV and –ECD traces of *rac*-**20c** on Chiralpak IA column with hexane/2-propanol 80:20 eluent monitored at 230 nm. 33
Figure S290. HPLC-ECD spectra of the first- [(4a*R*,5*S*,10b*S*), black] and second-eluting [(4a*S*,5*R*,10b*R*), red] enantiomers of *rac*-**20c**. 33
Figure S291. HPLC-UV and –ECD traces of *rac*-**20d** on Chiralpak IA column with hexane/2-propanol 80:20 eluent monitored at 280 nm. 34
Figure S292. HPLC-ECD spectra of the first- [(4a*R*,5*S*,10b*S*), black] and second-eluting [(4a*S*,5*R*,10b*R*), red] enantiomers of *rac*-**20d**. 34

Figure S293. HPLC-UV and –ECD traces of *rac*-**20e** on Chiralpak IA column with hexane/2-propanol 80:20 eluent monitored at 280 nm. 35
Figure S294. HPLC-ECD spectra of the first- [(4a*R*,5*S*,10b*S*), black] and second-eluting [(4a*S*,5*R*,10b*R*), red] enantiomers of *rac*-**20e**. 35
Figure S295. HPLC-UV and –ECD traces of *rac*-**20f** on Chiralpak IA column with hexane/2-propanol 80:20 eluent monitored at 225 nm................. 36
Figure S296. HPLC-ECD spectra of the first- [(4a*S*,5*R*,10b*R*), black] and second-eluting [(4a*R*,5*S*,10b*S*), red] enantiomers of *rac*-**20f**. 36
Figure S297. HPLC-UV and –ECD traces of *rac*-**20g** on Chiralpak IA column with hexane/2-propanol 90:10 eluent monitored at 225 nm. 37
Figure S298. HPLC-ECD spectra of the first- [(4a*S*,5*R*,10b*R*), black] and second-eluting [(4a*R*,5*S*,10b*S*), red] enantiomers of *rac*-**20g**...................... 37
Figure S299. HPLC-UV and –ECD traces of *rac*-**23a** on Chiralpak IA column with hexane/2-propanol 80:20 eluent monitored at 230 nm. 38
Figure S300. HPLC-ECD spectra of the first- [(4a*S*,5*S*,10b*S*), black] and second-eluting [(4a*R*,5*R*,10b*R*), red] enantiomers of *rac*-**23a**...................... 38
Figure S301. HPLC-UV and –ECD traces of *rac*-**23b** on Chiralpak IA column with hexane/2-propanol 80:20 eluent monitored at 270 nm................ 39
Figure S302. HPLC-ECD spectra of the first- [(4a*R*,5*R*,10b*R*), black] and second-eluting [(4a*S*,5*S*,10b*S*), red] enantiomers of *rac*-**23b**. 39
Figure S303. HPLC-UV and –ECD traces of *rac*-**23c** on Chiralpak IA column with hexane/2-propanol 80:20 eluent monitored at 270 nm. 40
Figure S304. HPLC-ECD spectra of the first- [(4a*R*,5*R*,10b*R*), black] and second-eluting [(4a*S*,5*S*,10b*S*), red] enantiomers of *rac*-**23c**. 40
Figure S305. HPLC-UV and –ECD traces of *rac*-**23d** on Chiralpak IA column with hexane/2-propanol 80:20 eluent monitored at 270 nm................ 41
Figure S306. HPLC-ECD spectra of the first- [(4a*S*,5*S*,10b*S*), black] and second-eluting [(4a*R*,5*R*,10b*R*), red] enantiomers of *rac*-**23d**. 41
Figure S307. HPLC-UV and –ECD traces of *rac*-**23e** on Chiralpak IA column with hexane/2-propanol 80:20 eluent monitored at 280 nm. 42
Figure S308. HPLC-ECD spectra of the first- [(4a*R*,5*R*,10b*R*), black] and second-eluting [(4a*S*,5*S*,10b*S*), red] enantiomers of *rac*-**23e**. 42
Figure S309. HPLC-UV and –ECD traces of *rac*-**23g** on Chiralpak IA column with hexane/2-propanol 80:20 eluent monitored at 230 nm. 43
Figure S310. HPLC-ECD spectra of the first- [(4a*R*,5*R*,10b*R*), black] and second-eluting [(4a*S*,5*S*,10b*S*), red] enantiomers of *rac*-**23g**...................... 43
Figure S311. HPLC-UV and –ECD traces of *rac*-**3a** on Chiralpak IA column with hexane/2-propanol 80:20 eluent monitored at 240 nm. 44
Figure S312. HPLC-ECD spectra of the first- [(4*R*), black] and second-eluting [(4*S*), red] enantiomers of *rac*-**3a**. 44
Figure S313. HPLC-UV and –ECD traces of *rac*-**3b** on Chiralpak IA column with hexane/2-propanol 80:20 eluent monitored at 250 nm................. 45
Figure S314. HPLC-ECD spectra of the first- [(4*R*), black] and second-eluting [(4*S*), red] enantiomers of *rac*-**3b**.. 45
Figure S315. HPLC-UV and –ECD traces of *rac*-**3c** on Chiralpak IA column with hexane/2-propanol 80:20 eluent monitored at 240 nm. 46
Figure S316. HPLC-ECD spectra of the first- [(4*S*), black] and second-eluting [(4*R*), red] enantiomers of *rac*-**3c**. 46
Figure S317. HPLC-UV and –ECD traces of *rac*-**3d** on Chiralpak IA column with hexane/2-propanol 80:20 eluent monitored at 240 nm................. 47
Figure S318. HPLC-ECD spectra of the first- [(4*R*), black] and second-eluting [(4*S*), red] enantiomers of *rac*-**3d**. 47

Figure S319. HPLC-UV and –ECD traces of *rac*-**3e** on Chiralpak IA column with hexane/2-propanol 80:20 eluent monitored at 240 nm. 48
Figure S320. HPLC-ECD spectra of the first- [(4*R*), black] and second-eluting [(4*S*), red] enantiomers of *rac*-**3e**. 48
Figure S321. HPLC-UV and –ECD traces of *rac*-**3f** on Chiralpak IA column with hexane/2-propanol 80:20 eluent monitored at 240 nm.................. 49
Figure S322. HPLC-ECD spectra of the first- [(4*R*), black] and second-eluting [(4*S*), red] enantiomers of *rac*-**3f**... 49
Figure S323. HPLC-UV and –ECD traces of *rac*-**3g** on Chiralpak IA column with hexane/2-propanol 80:20 eluent monitored at 240 nm. 50
Figure S324. HPLC-ECD spectra of the first- [(4*R*), black] and second-eluting [(4*S*), red] enantiomers of *rac*-**3g**. 50
Figure S325. HPLC-UV and –ECD traces of *rac*-**4a** on Chiralpak IA column with hexane/2-propanol 80:20 eluent monitored at 230 nm. 51
Figure S326. HPLC-ECD spectra of the first- [(4*R*), black] and second-eluting [(4*S*), red] enantiomers of *rac*-**4a**. 51
Figure S327. HPLC-UV and –ECD traces of *rac*-**4b** on Chiralpak IA column with hexane/2-propanol 90:10 eluent monitored at 235 nm.................. 52
Figure S328. HPLC-ECD spectra of the first- [(4*R*), black] and second-eluting [(4*S*), red] enantiomers of *rac*-**4b**. 52
Figure S329. HPLC-UV and –ECD traces of *rac*-**4c** on Chiralpak IA column with hexane/2-propanol 90:10 eluent monitored at 235 nm. 53
Figure S330. HPLC-ECD spectra of the first- [(4*R*), black] and second-eluting [(4*S*), red] enantiomers of *rac*-**4c**. 53
Figure S331. HPLC-UV and –ECD traces of *rac*-**4d** on Chiralpak IA column with hexane/2-propanol 80:20 eluent monitored at 235 nm.................. 54
Figure S332. HPLC-ECD spectra of the first- [(4*R*), black] and second-eluting [(4*S*), red] enantiomers of *rac*-**4d**. 54
Figure S333. HPLC-UV and –ECD traces of *rac*-**4e** on Chiralpak IC column with hexane/2-propanol 70:30 eluent monitored at 240 nm. 55
Figure S334. HPLC-ECD spectra of the first- [(4*R*), black] and second-eluting [(4*S*), red] enantiomers of *rac*-**4e**. 55
Figure S335. HPLC-UV and –ECD traces of *rac*-**4f** on Chiralpak IA column with hexane/2-propanol 80:20 eluent monitored at 230 nm.................. 56
Figure S336. HPLC-ECD spectra of the first- [(4*R*), black] and second-eluting [(4*S*), red] enantiomers of *rac*-**4f**... 56
Figure S337. HPLC-UV and –ECD traces of *rac*-**4g** on Chiralpak IA column with hexane/2-propanol 80:20 eluent monitored at 235 nm. 57
Figure S338. HPLC-ECD spectra of the first- [(4*R*), black] and second-eluting [(4*S*), red] enantiomers of *rac*-**4g**. 57
3. ECD calculations……………………………………………………………………………………………58
Figure S339. Structures and populations of the low-energy ωB97X/TZVP PCM/$CHCl_3$ conformers of (*R*)-**3e**……………………………………...58
Figure S340. HPLC-ECD spectra of the first (black line) and the second-eluting (red line) enantiomers of **3a** compared with the CAM-B3LYP/TZVP PCM/$CHCl_3$ // CAM-B3LYP/TZVP PCM/$CHCl_3$ spectrum of (*R*)-**3a** (dark yellow line). The bars represent rotational strength values for the single low-energy solution conformer……………………………………………………………………………………59
Figure S341. Single low-energy CAM-B3LYP/TZVP PCM/$CHCl_3$ conformer of (*R*)-**3a**……………………………………………………….59

Figure S342. HPLC-ECD spectra of the first-eluting enantiomer (black line, enantiomer corrected) of **3f** compared with the B3LYP/TZVP PCM/$CHCl_3$ // ωB97X/TZVP PCM/$CHCl_3$ spectrum of (*R*)-**3f** (red line). The bars represent rotational strength values for the lowest-energy solution conformer……60

Figure S343. Structures and populations of the low-energy ωB97X/TZVP PCM/$CHCl_3$ conformers of (*R*)-**3f**……60

Figure S344. Structures and populations of the low-energy ωB97X/TZVP PCM/$CHCl_3$ conformers of (*R*)-**4g**……61

Figure S345. HPLC-ECD spectra of the first-eluting enantiomer (black line) of **4f** compared with the CAM-B3LYP/TZVP PCM/$CHCl_3$ // CAM-B3LYP/TZVP PCM/$CHCl_3$ spectrum of (*R*)-**4f** (dark yellow line). The bars represent rotational strength values for the lowest-energy solution conformer……62

Figure S346. Structures and populations of the low-energy CAM-B3LYP/TZVP PCM/$CHCl_3$ conformers of (*R*)-**4f**……62

Figure S347. HPLC-ECD spectra of the first (black line) and the second-eluting (red line) enantiomers of **4a** compared with the BH&HLYP/TZVP PCM/$CHCl_3$ (blue line) and the PBE0/TZVP PCM/$CHCl_3$ (purple line) spectra of (*R*)-**4a** (level of DFT optimization: ωB97X/TZVP PCM/$CHCl_3$)……63

Figure S348. Structures and populations of the low-energy ωB97X/TZVP PCM/$CHCl_3$ conformers of (*R*)-**29a**……63

4. X-ray analysis of 17e……64

Figure S349. ORTEP view of **17e** drawn at 50% probability level with partial numbering scheme……64

Table S2. Experimental details of single crystal X-ray diffraction measurement of **17e**……64

1 Investigation of cellular viability

A

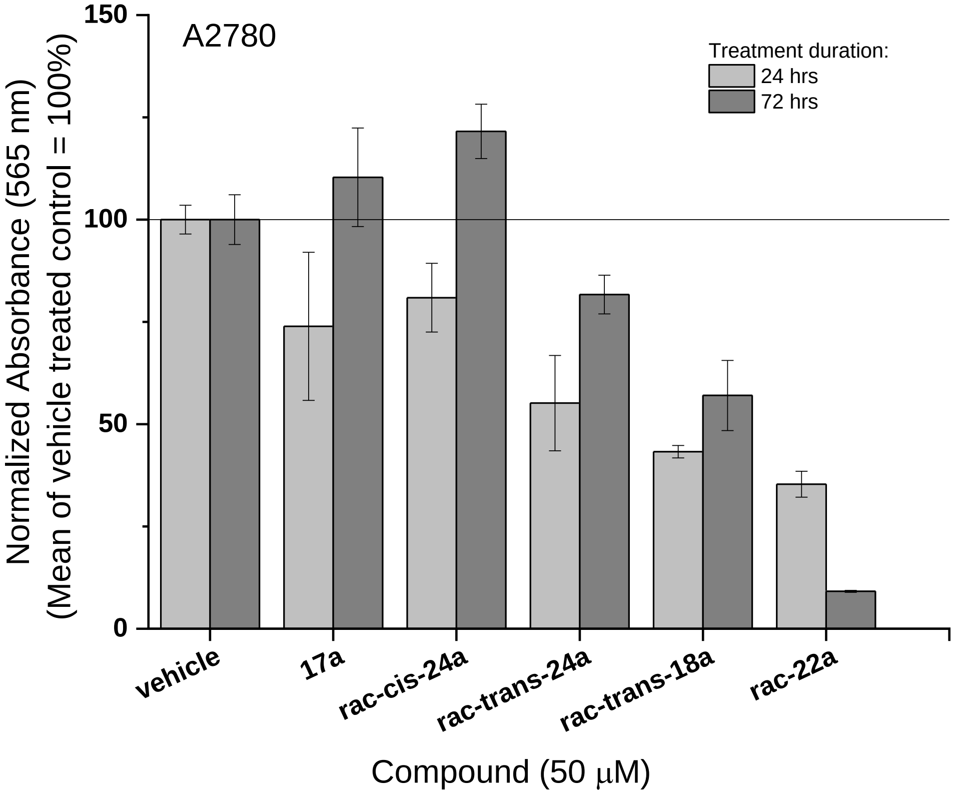

B

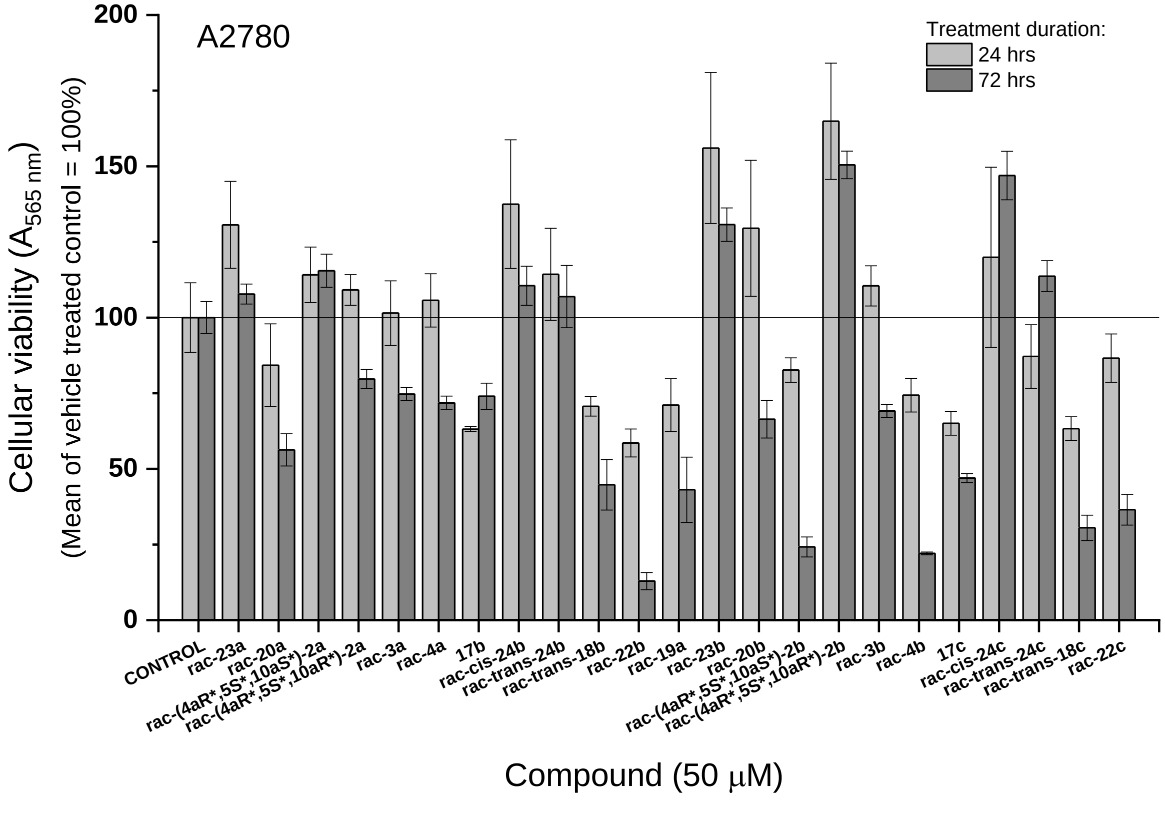

C

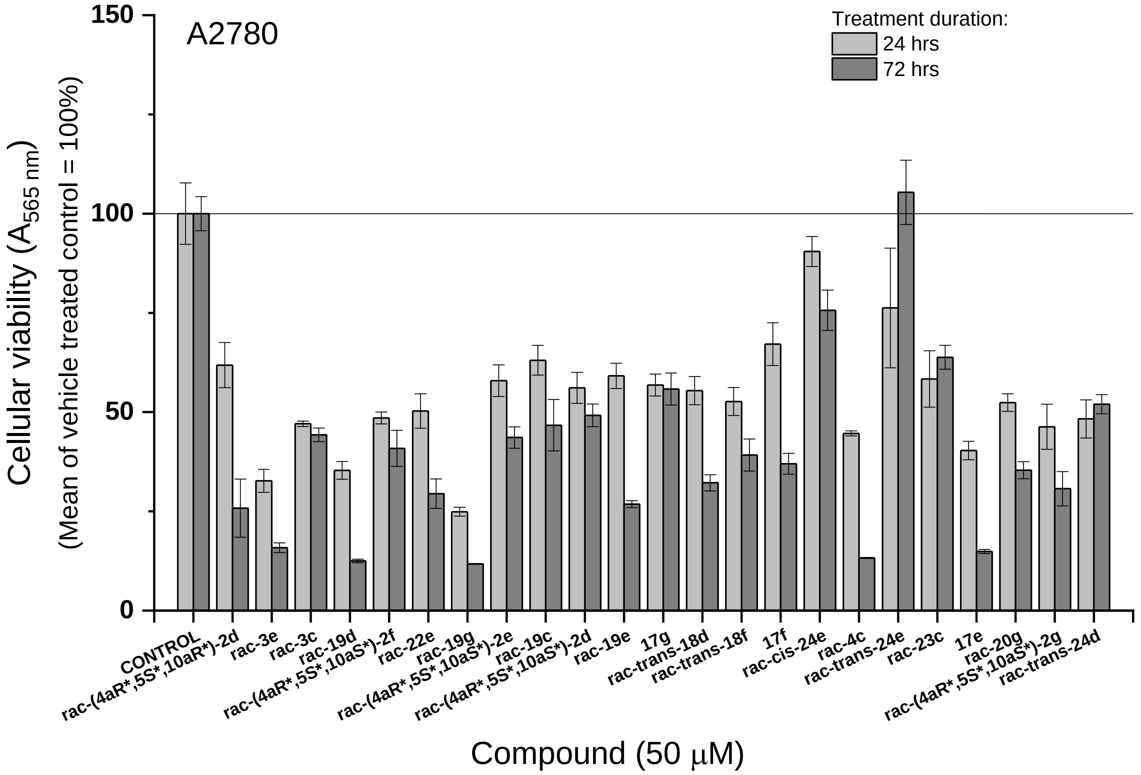

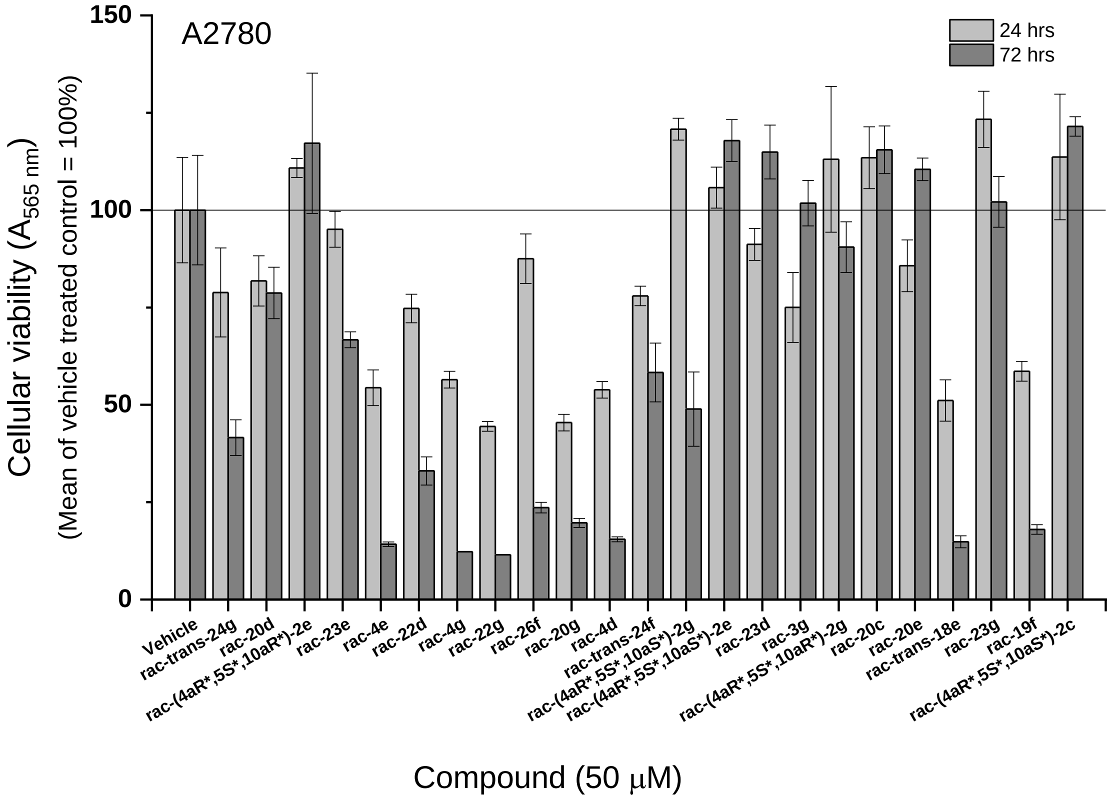

Figure S262. Effect of synthesized compounds on the viable cell number of A2780 ovarian carcinoma cell line (A-D). 50 μM solution of each compound was applied daily, for either 1 or 3 days. Control cells were treated with equal amount of vehicle solvent (DMSO). Data are presented as Mean ±SEM, N = 4 at each data point.

E

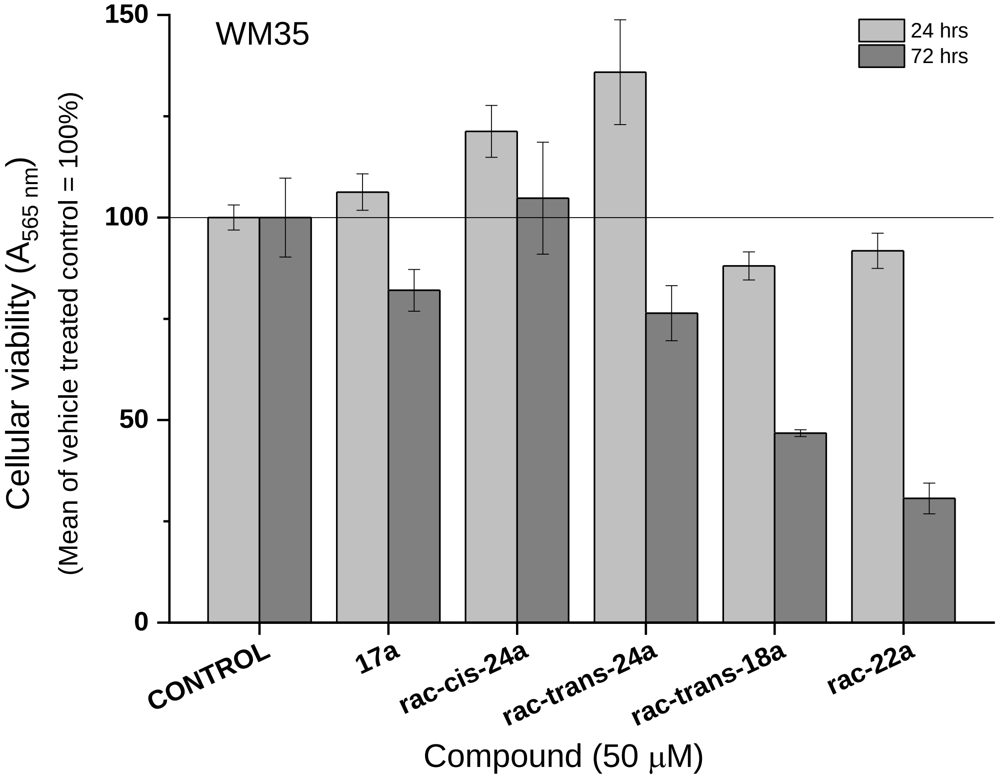

WM35
24 hrs
72 hrs
Cellular viability ($A_{565\ nm}$)
(Mean of vehicle treated control = 100%)
150
100
50
0
CONTROL
17a
rac-cis-24a
rac-trans-24a
rac-trans-18a
rac-22a
Compound (50 μM)

F

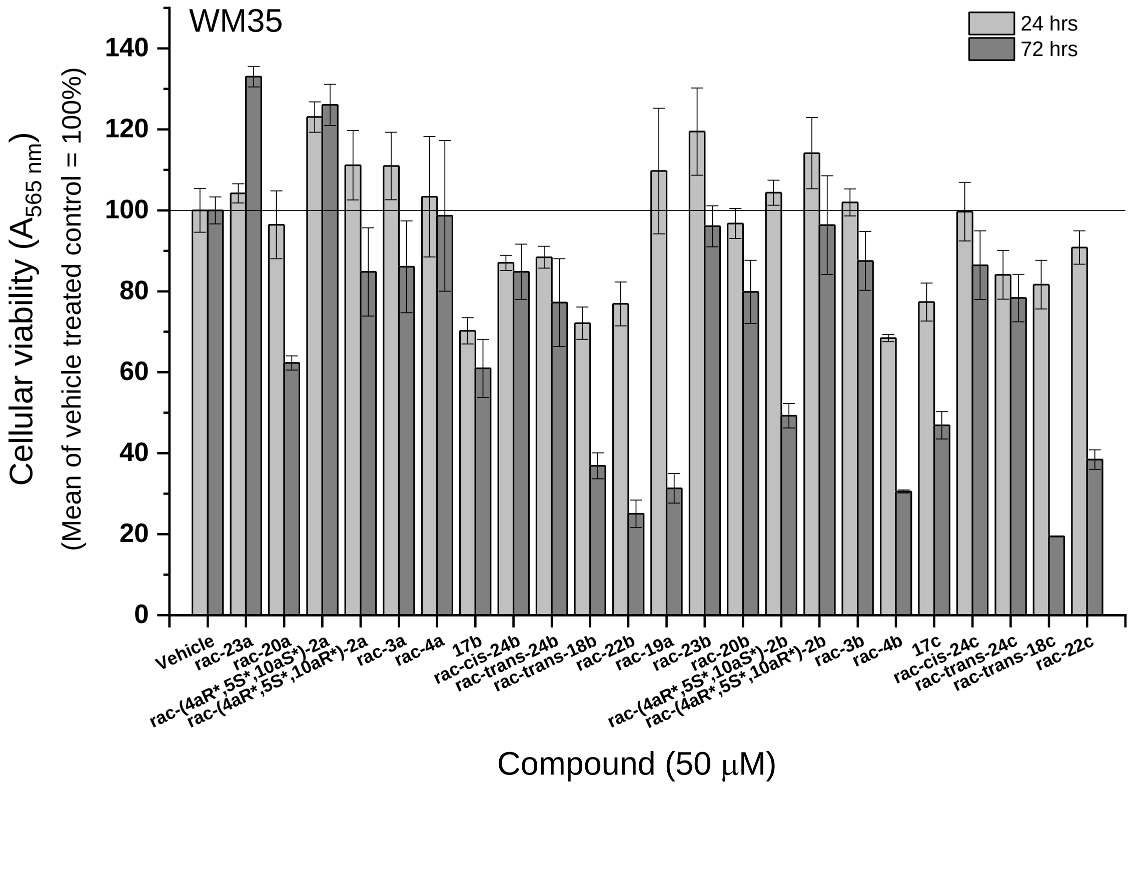

WM35
24 hrs
72 hrs
Cellular viability ($A_{565\ nm}$)
(Mean of vehicle treated control = 100%)
140
120
100
80
60
40
20
0
Vehicle
rac-23a
rac-20a
rac-(4aR*,5S*,10aS*)-2a
rac-(4aR*,5S*,10aR*)-2a
rac-3a
rac-4a
17b
rac-cis-24b
rac-trans-24b
rac-trans-18b
rac-22b
rac-19a
rac-23b
rac-20b
rac-(4aR*,5S*,10aS*)-2b
rac-(4aR*,5S*,10aR*)-2b
rac-3b
rac-4b
17c
rac-cis-24c
rac-trans-24c
rac-trans-18c
rac-22c
Compound (50 μM)

G

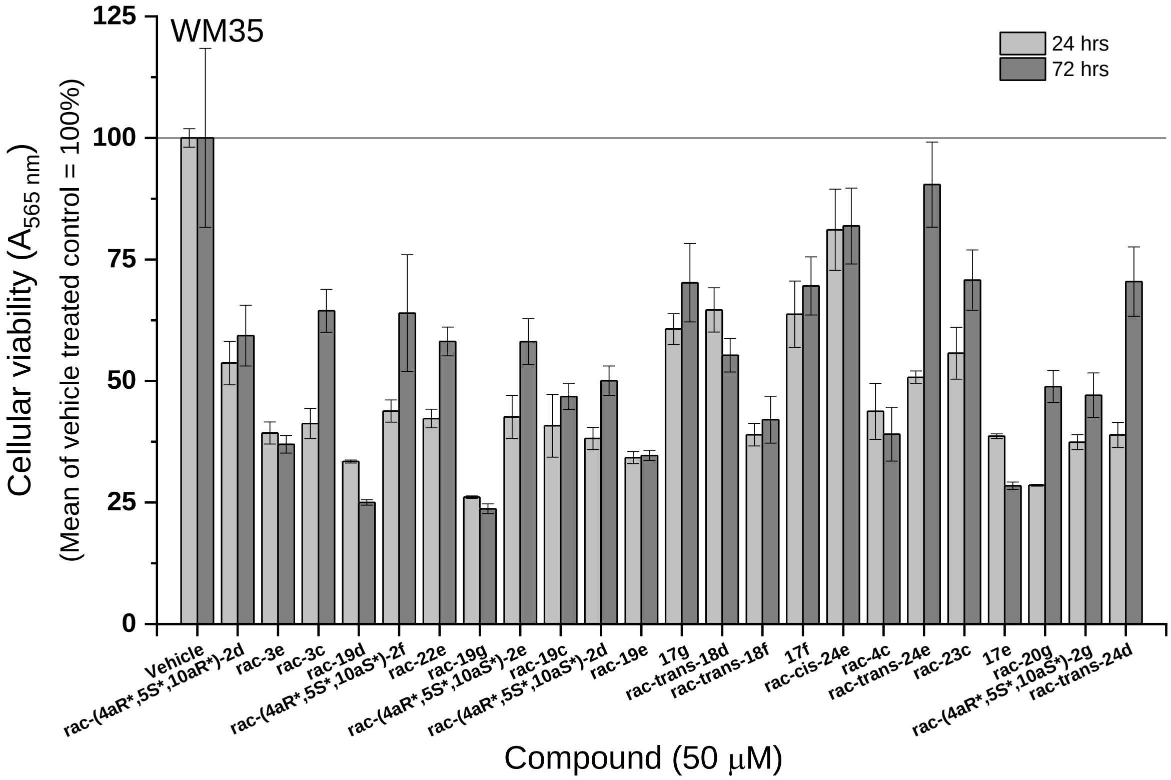

H

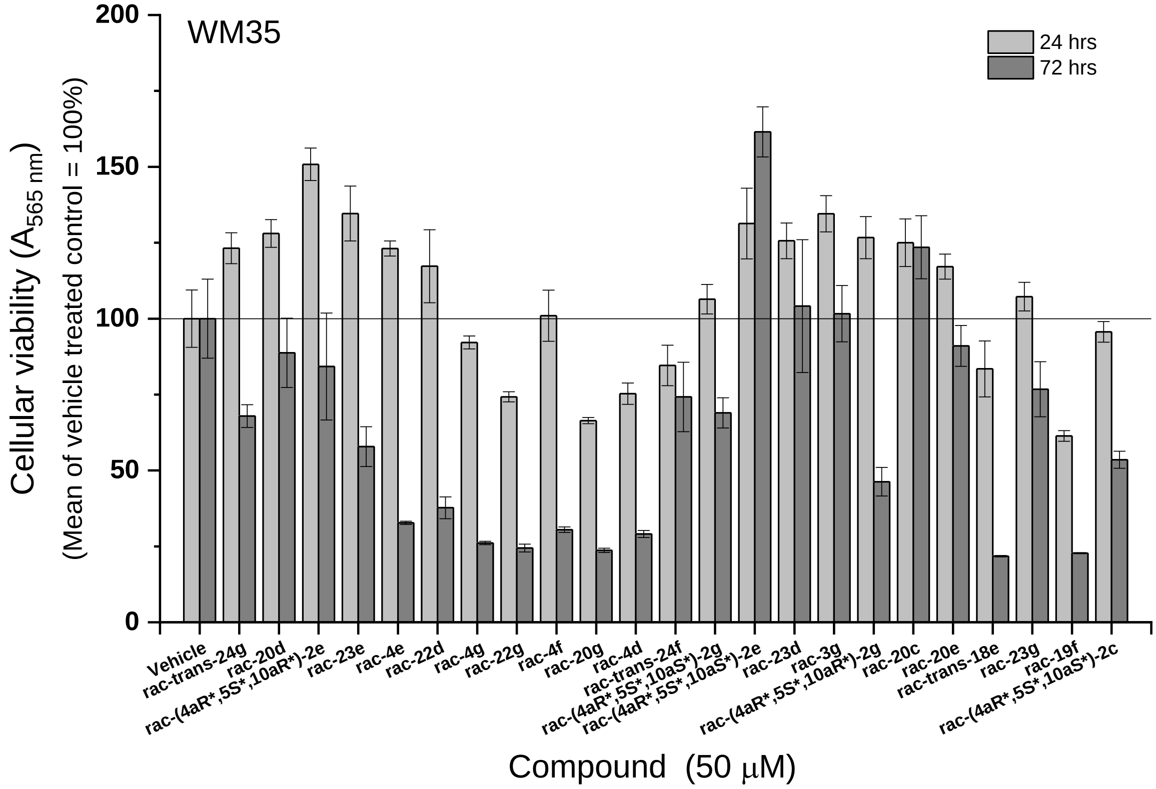

Figure S263. Effect of synthesized compounds on the viable cell number of WM35 melanoma cell line (E-H). 50 μM solution of each compound was applied daily, for either 1 or 3 days. Control cells were treated with equal amount of vehicle solvent (DMSO). Data are presented as Mean±SEM, N = 4 at each data point.

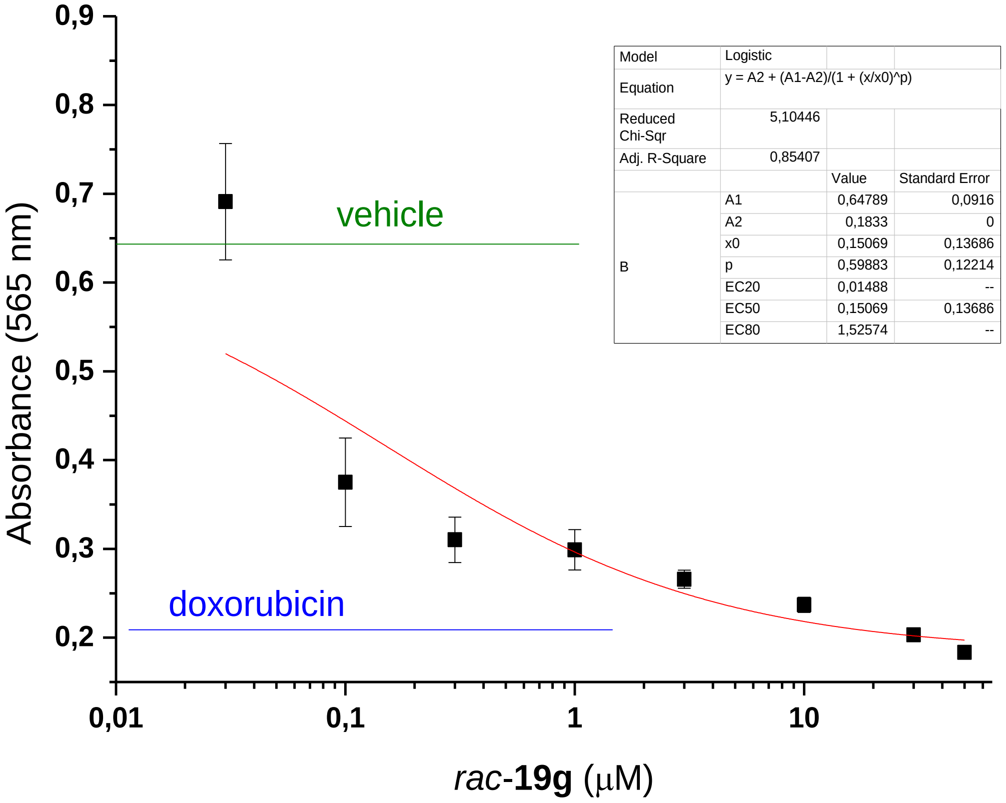

Figure S264. Concentration-dependent effect of rac-**19g** on the viability of A2780 cells. Cells were treated daily for 3 days, subjected for MTT assay and IC_{50} value was determined as described in the Experimental section. Green line indicates the value of negative control treated with equal amount of vehicle solvent (DMSO) and blue line represents the effect of 1 μg/ml doxorubicin used as positive control. Data are presented as Mean ±SEM, N=4 at each data point.

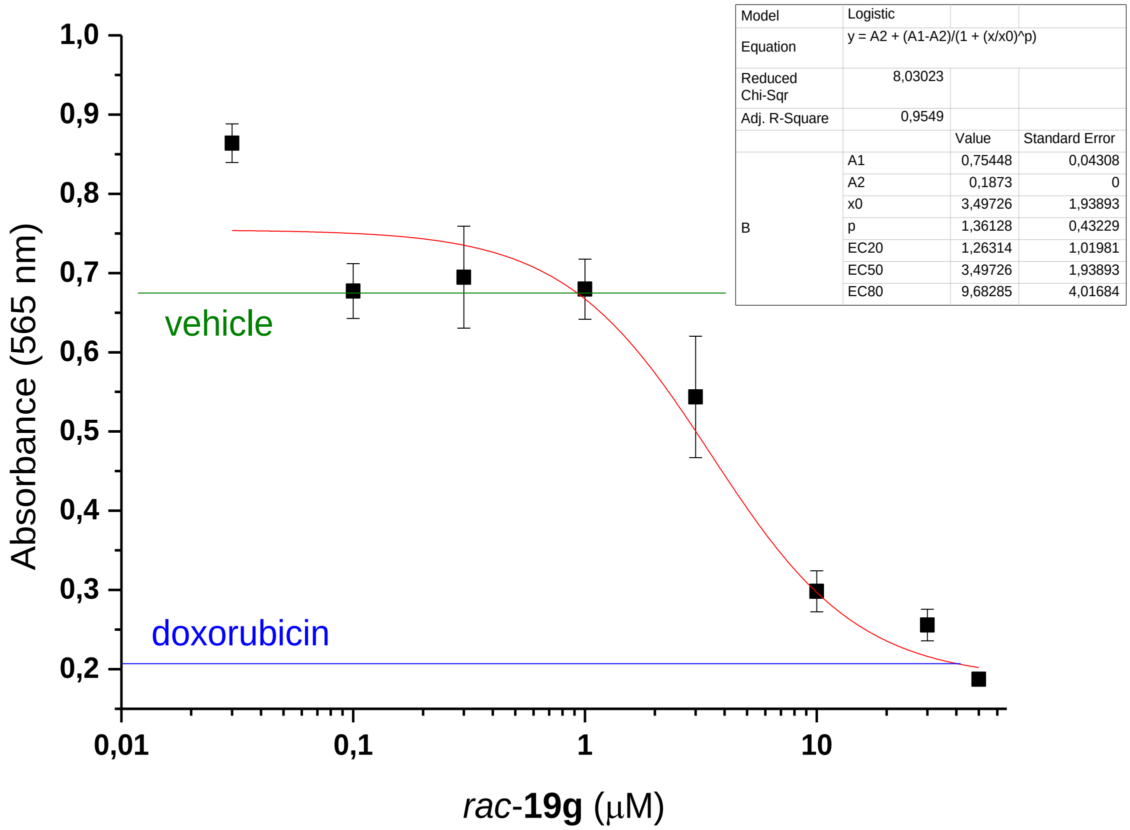

Figure S265. Concentration-dependent effect of rac-**19g** on the viability of WM35 cells. Cells were treated daily for 3 days, subjected for MTT assay and IC_{50} value was determined as described in the Experimental section. Green line indicates the value of negative control treated with equal amount of vehicle solvent (DMSO) and blue line represents the effect of 1 μg/ml doxorubicin used as positive control. Data are presented as Mean ±SEM, N=4 at each data point.

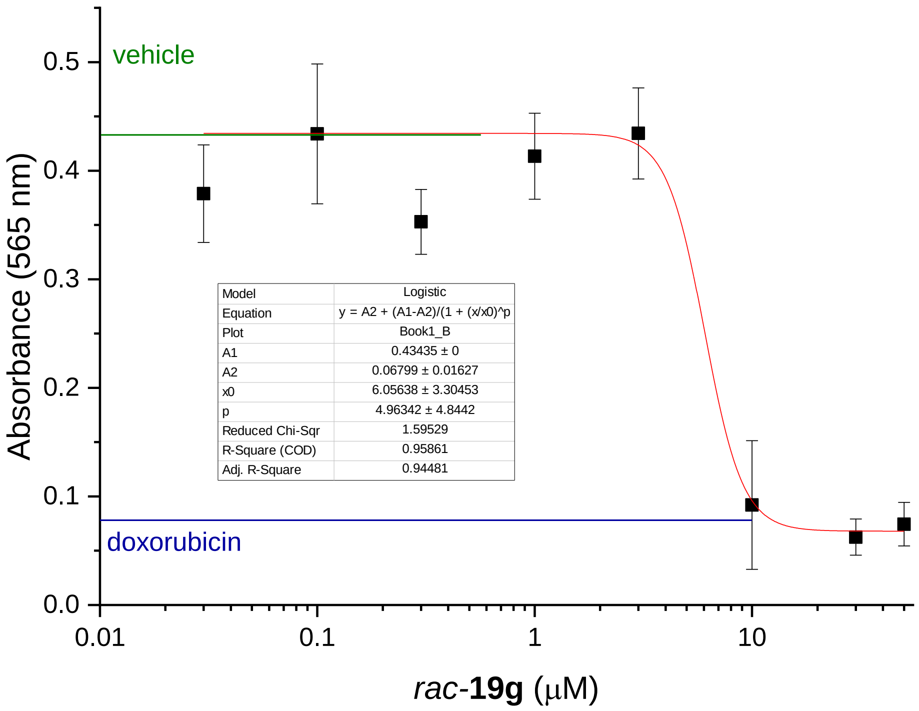

Figure S266. Concentration-dependent effect of rac-**19g** on the viability of HaCaT keratinocytes. Cells were treated daily for 3 days, subjected for MTT assay and IC_{50} value was determined as described in the Experimental section. Green line indicates the value of negative control treated with equal amount of vehicle solvent (DMSO) and blue line represents the effect of 1 μg/ml doxorubicin used as positive control. Data are presented as Mean ±SEM, N=4 at each data point.

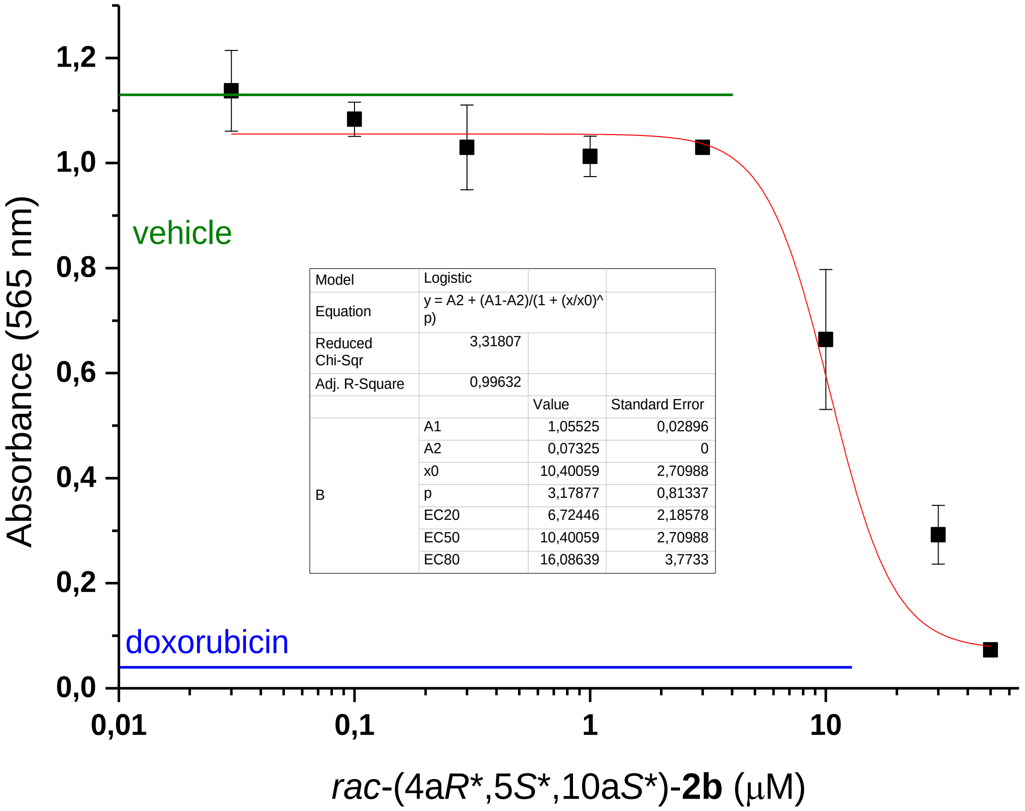

Figure S267. Concentration-dependent effect of rac-(4a*S**,5*R**,10b*R**)-**2b** on the viability of A2780 cells. Cells were treated daily for 3 days, subjected for MTT assay and IC_{50} value was determined as described in the Experimental section. Green line indicates the value of negative control treated with equal amount of vehicle solvent (DMSO) and blue line represents the effect of 1 μg/ml doxorubicin used as positive control. Data are presented as Mean ±SEM, N=4 at each data point.

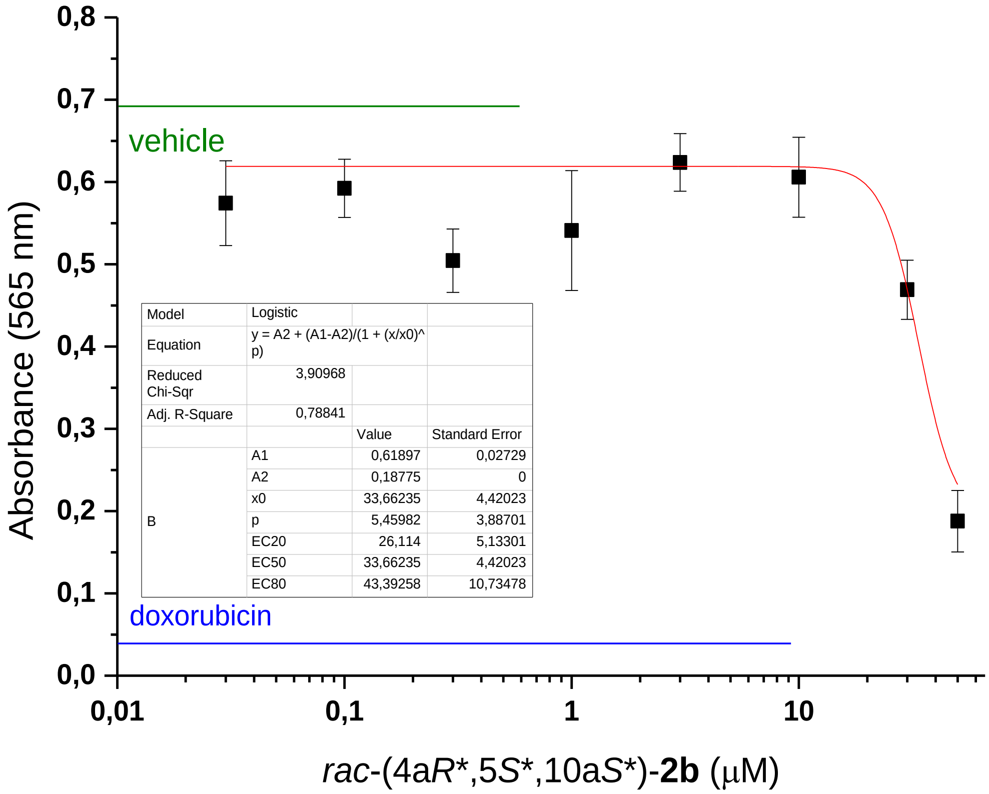

Figure S268. Concentration-dependent effect of rac-(4a*S**,5*R**,10b*R**)-**2b** on the viability of WM35 cells. Cells were treated daily for 3 days, subjected for MTT assay and IC_{50} value was determined as described in the Experimental section. Green line indicates the value of negative control treated with equal amount of vehicle solvent (DMSO) and blue line represents the effect of 1 μg/ml doxorubicin used as positive control. Data are presented as Mean ±SEM, N=4 at each data point.

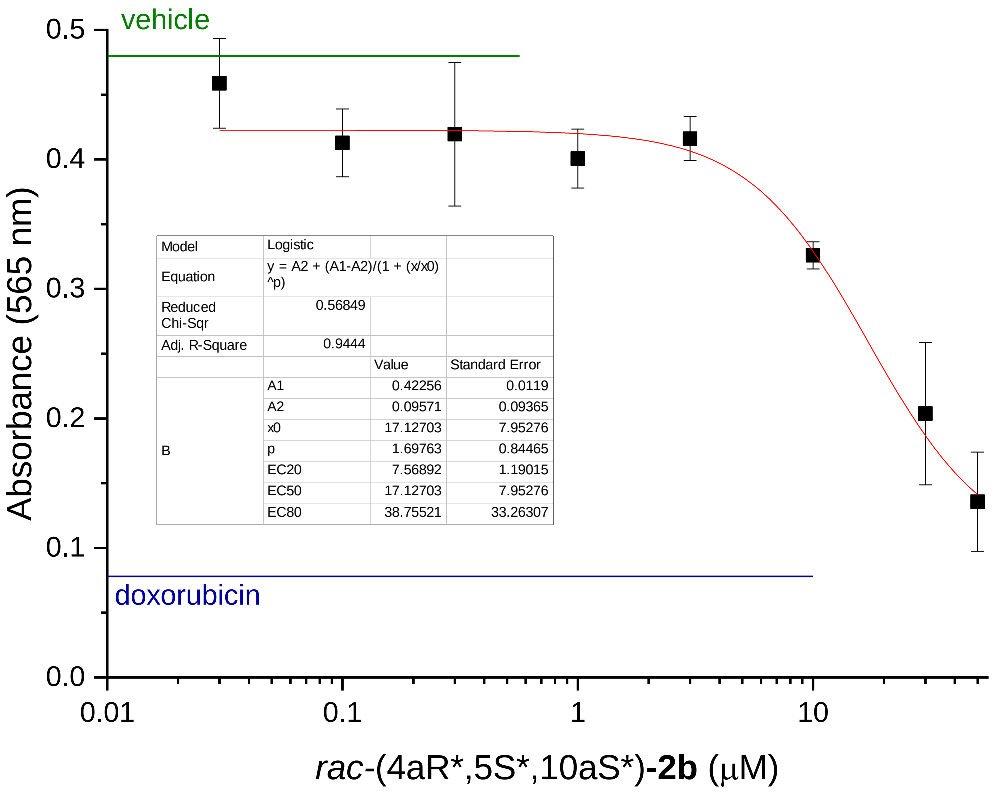

Figure S269. Concentration-dependent effect of rac-(4a*S**,5*R**,10b*R**)-**2b** on the viability of HaCaT keratinocytes. Cells were treated daily for 3 days, subjected for MTT assay and IC_{50} value was determined as described in the Experimental section. Green line indicates the value of negative control treated with equal amount of vehicle solvent (DMSO) and blue line represents the effect of 1 μg/ml doxorubicin used as positive control. Data are presented as Mean ±SEM, N=4 at each data point.

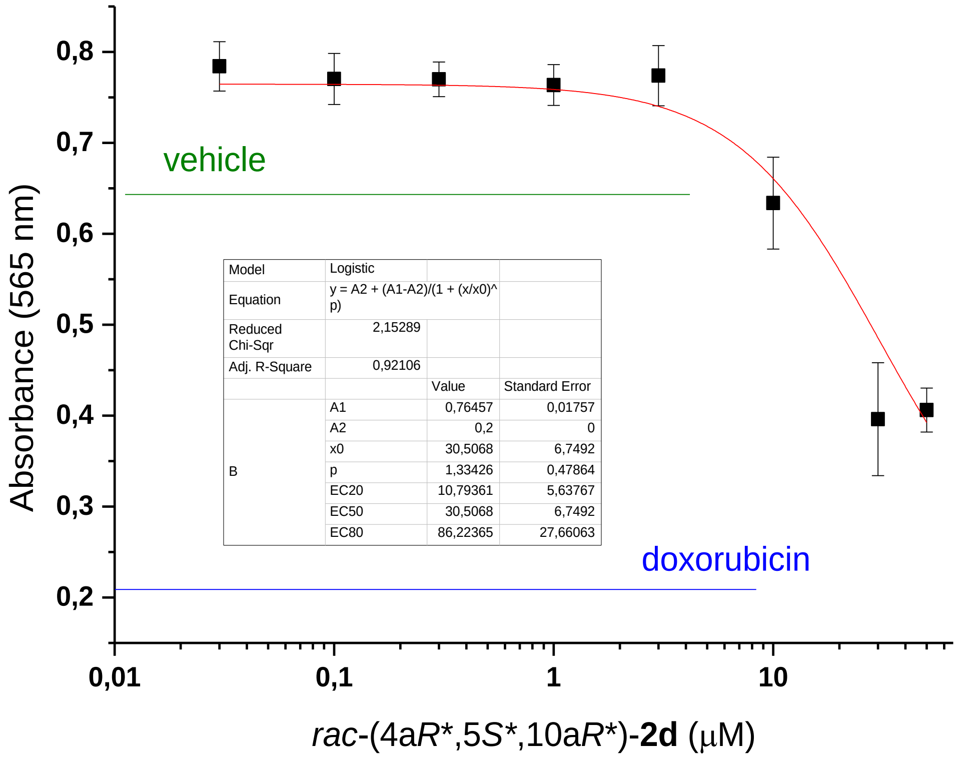

Figure S270. Concentration-dependent effect of rac-(4a*S**,5*R**,10b*S**)-**2d** on the viability of A2780 cells.Cells were treated daily for 3 days, subjected for MTT assay and IC_{50} value was determined as described in the Experimental section. Green line indicates the value of negative control treated with equal amount of vehicle solvent (DMSO) and blue line represents the effect of 1 μg/ml doxorubicin used as positive control. Data are presented as Mean ±SEM, N=4 at each data point.

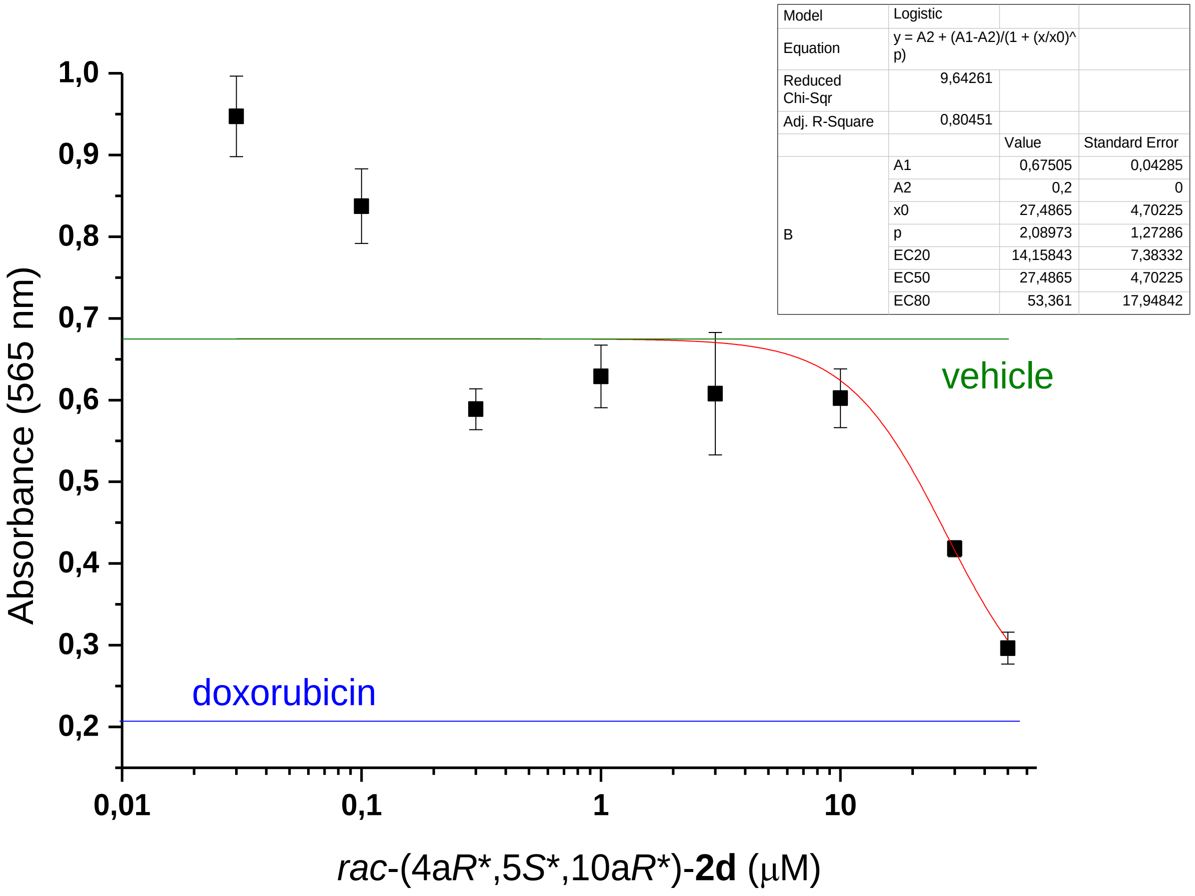

Figure S271. Concentration-dependent effect of rac-(4a*S**,5*R**,10b*S**)-**2d** on the viability of WM35 cells. Cells were treated daily for 3 days, subjected for MTT assay and IC_{50} value was determined as described in the Experimental section. Green line indicates the value of negative control treated with equal amount of vehicle solvent (DMSO) and blue line represents the effect of 1 μg/ml doxorubicin used as positive control. Data are presented as Mean ±SEM, N=4 at each data point.

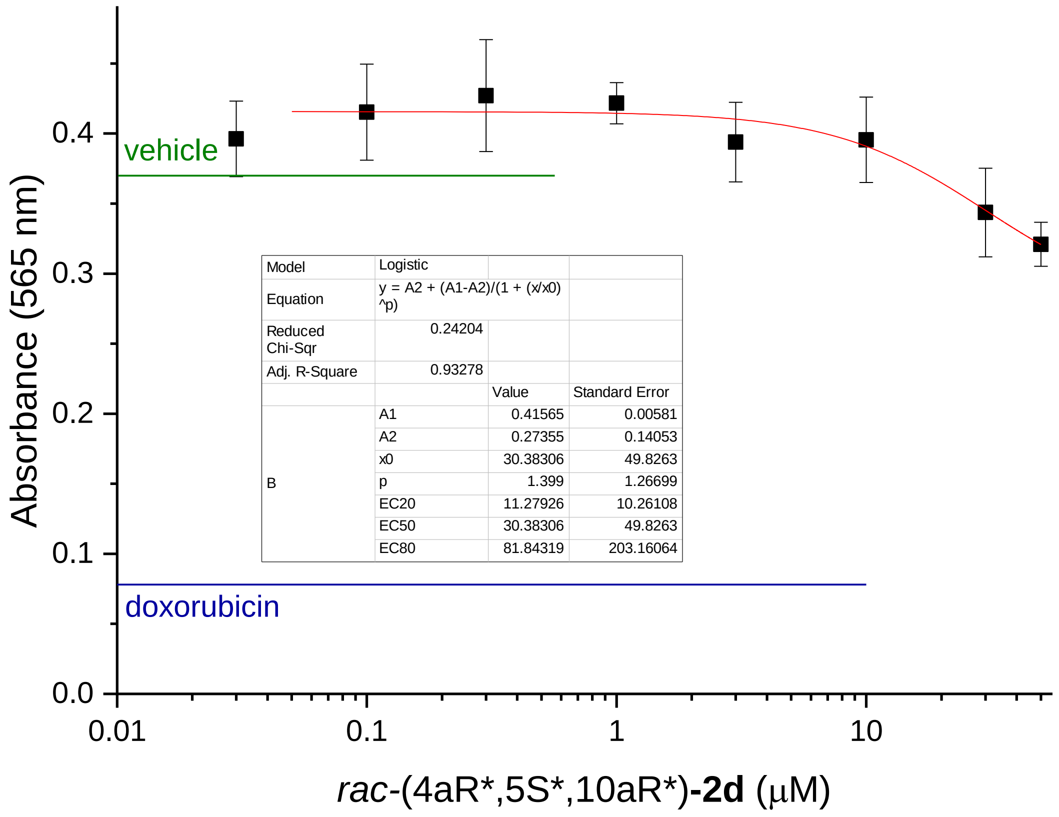

Figure S272. Concentration-dependent effect of rac-(4a*S**,5*R**,10b*S**)-**2d** on the viability of HaCaT keratinocytes. Cells were treated daily for 3 days, subjected for MTT assay and IC_{50} value was determined as described in the Experimental section. Green line indicates the value of negative control treated with equal amount of vehicle solvent (DMSO) and blue line represents the effect of 1 μg/ml doxorubicin used as positive control. Data are presented as Mean±SEM, N=4 at each data point.

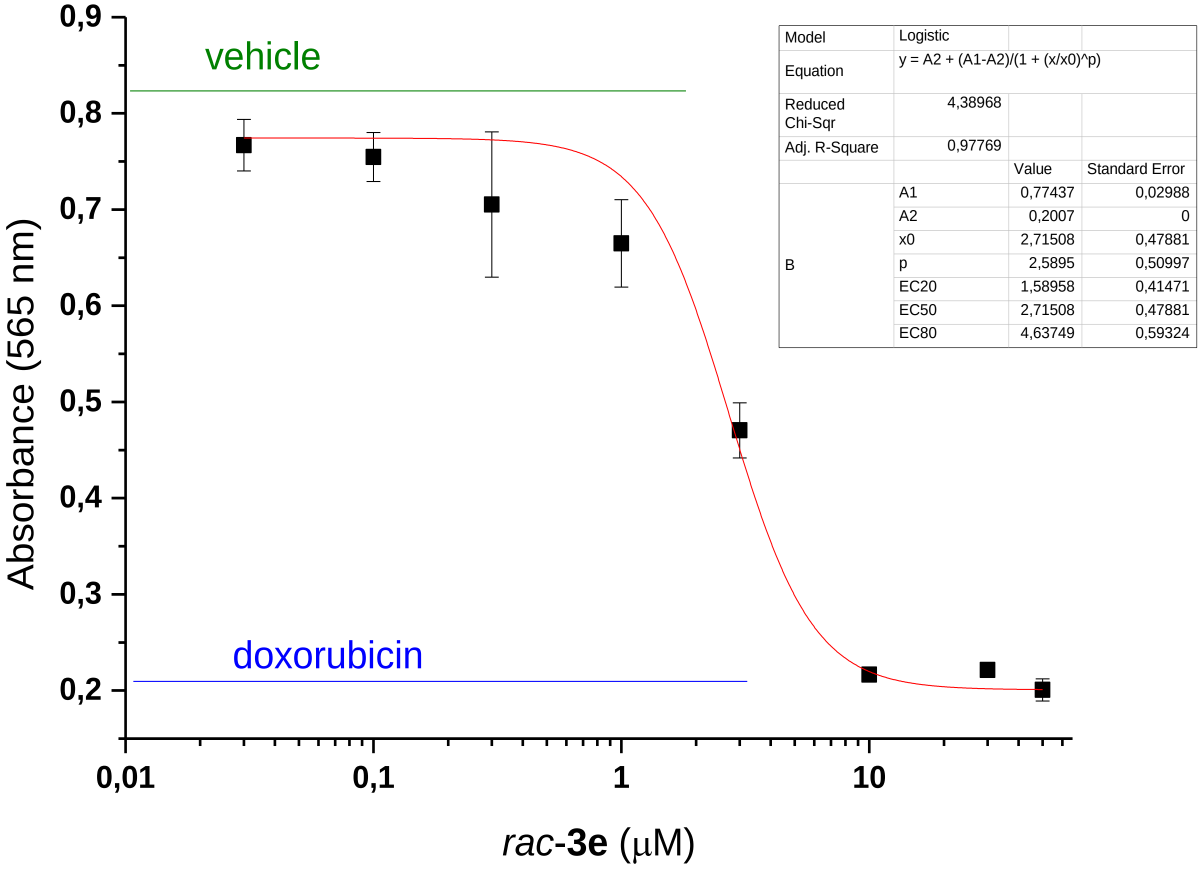

Figure S273. Concentration-dependent effect of rac-**3e** on the viability of A2780 cells. Cells were treated daily for 3 days, subjected for MTT assay and IC_{50} value was determined as described in the Experimental section. Green line indicates the value of negative control treated with equal amount of vehicle solvent (DMSO) and blue line represents the effect of 1 µg/ml doxorubicin used as positive control. Data are presented as Mean ±SEM, N=4 at each data point.

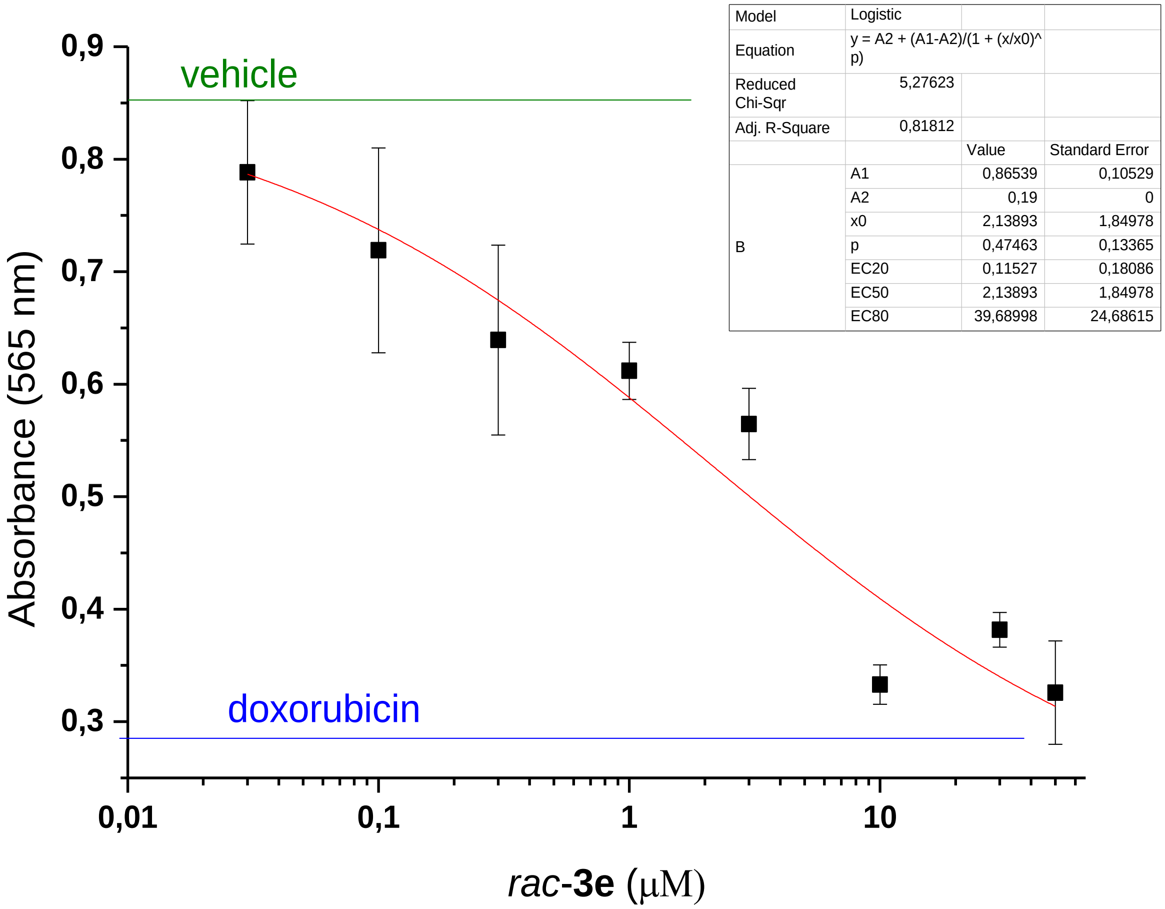

Figure S274. Concentration-dependent effect of rac-**3e** on the viability of WM35 cells. Cells were treated daily for 3 days, subjected for MTT assay and IC_{50} value was determined as described in the Experimental section. Green line indicates the value of negative control treated with equal amount of vehicle solvent (DMSO) and blue line represents the effect of 1 µg/ml doxorubicin used as positive control. Data are presented as Mean ±SEM, N=4 at each data point.

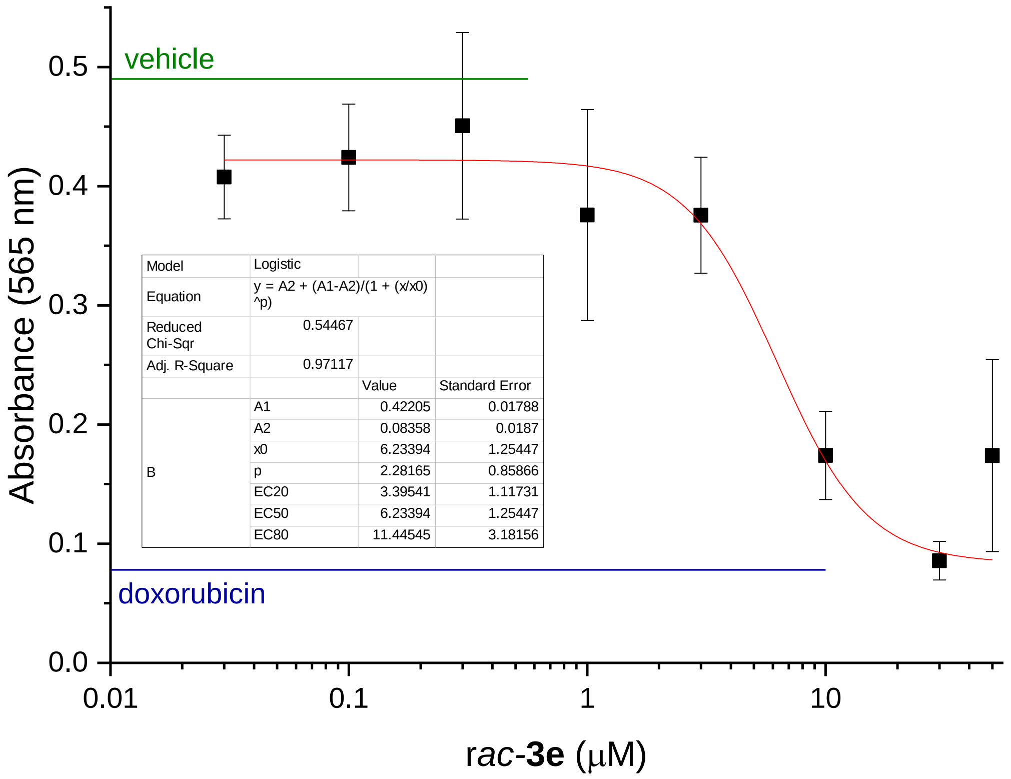

Figure S275. Concentration-dependent effect of *rac*-**3e** on the viability of HaCaT keratinocytes. Cells were treated daily for 3 days, subjected for MTT assay and IC50 value was determined as described in the Experimental section. Green line indicates the value of negative control treated with equal amount of vehicle solvent (DMSO) and blue line represents the effect of 1 μg/ml doxorubicin used as positive control. Data are presented as Mean ±SEM, N=4 at each data point.

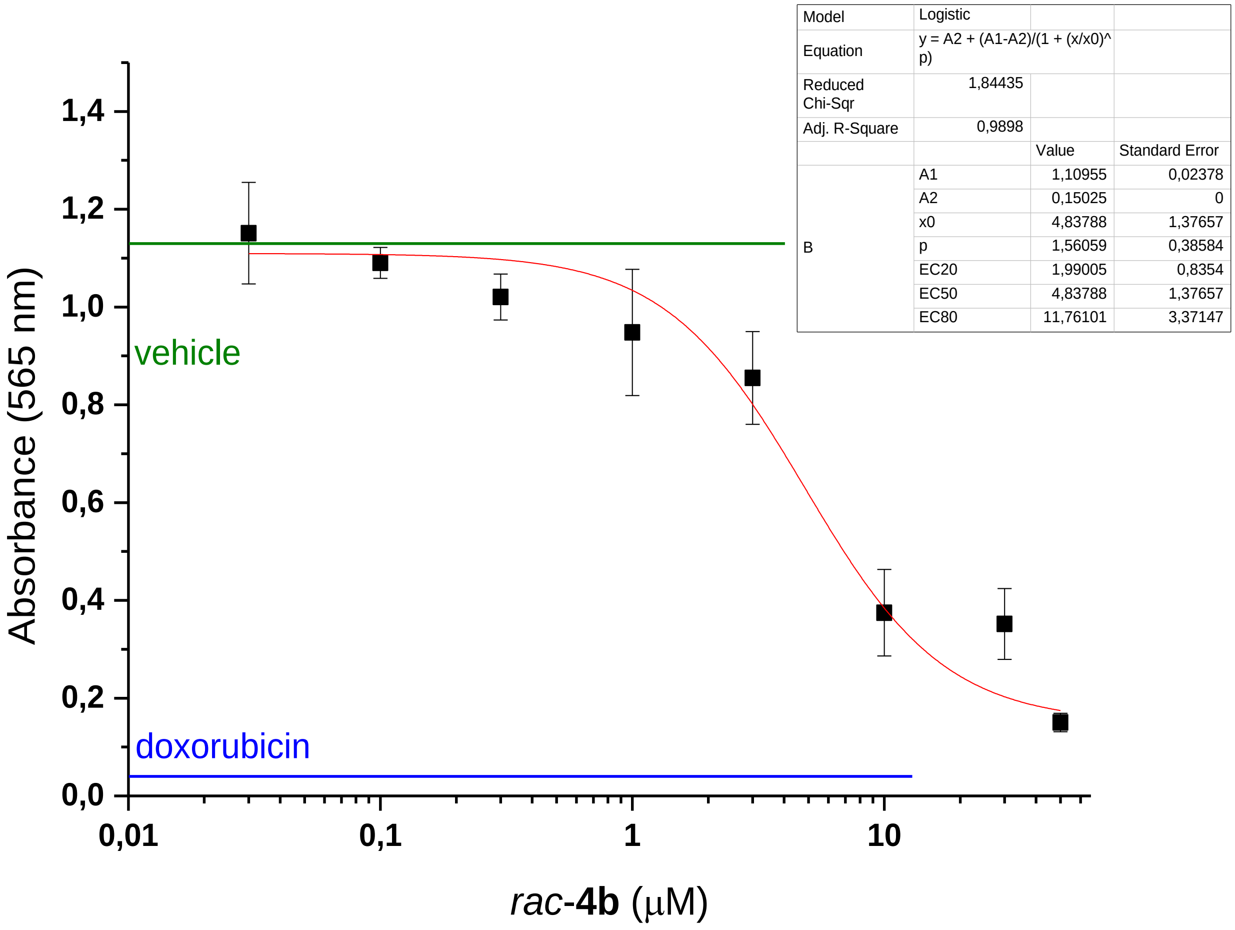

Figure S276. Concentration-dependent effect of *rac*-**4b** on the viability of A2780 cells. Cells were treated daily for 3 days, subjected for MTT assay and IC_{50} value was determined as described in the Experimental section. Green line indicates the value of negative control treated with equal amount of vehicle solvent (DMSO) and blue line represents the effect of 1 µg/ml doxorubicin used as positive control. Data are presented as Mean ±SEM, N=4 at each data point.

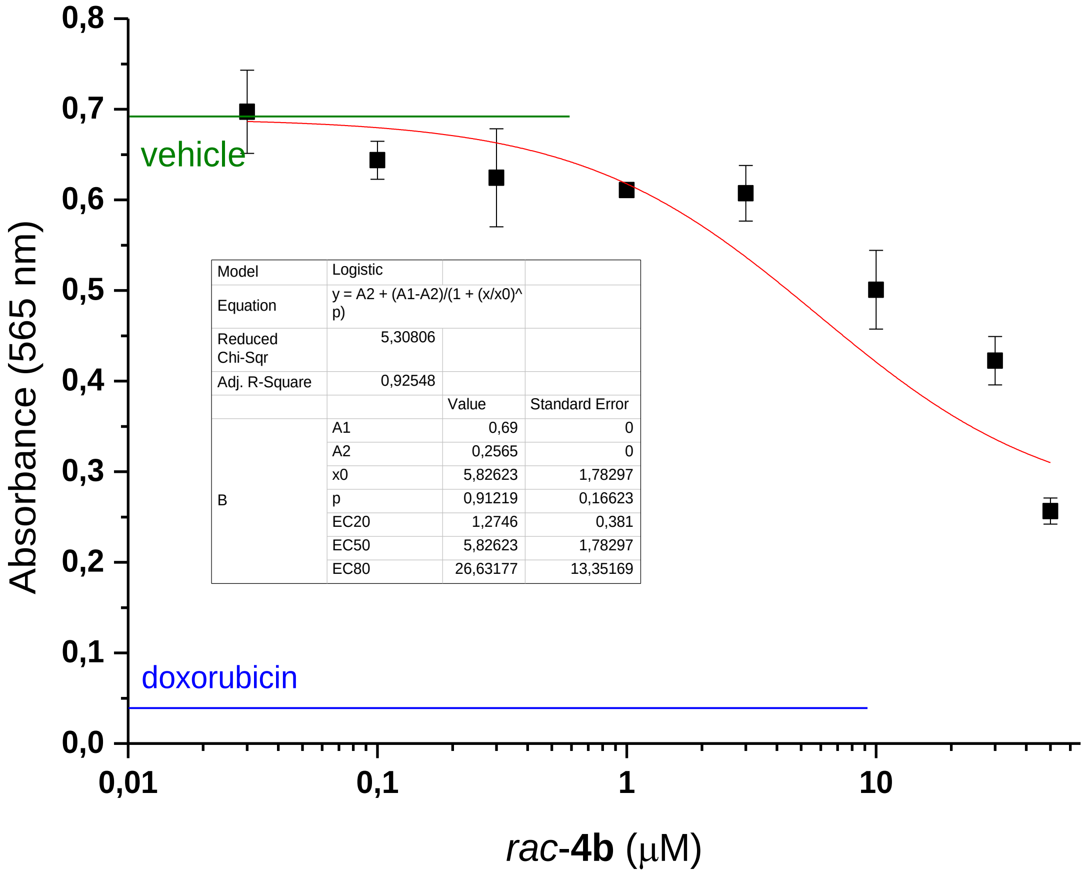

Figure S277. Concentration-dependent effect of *rac*-**4b** on the viability of WM35 cells. Cells were treated daily for 3 days, subjected for MTT assay and IC_{50} value was determined as described in the Experimental section. Green line indicates the value of negative control treated with equal amount of vehicle solvent (DMSO) and blue line represents the effect of 1 µg/ml doxorubicin used as positive control. Data are presented as Mean ±SEM, N=4 at each data point.

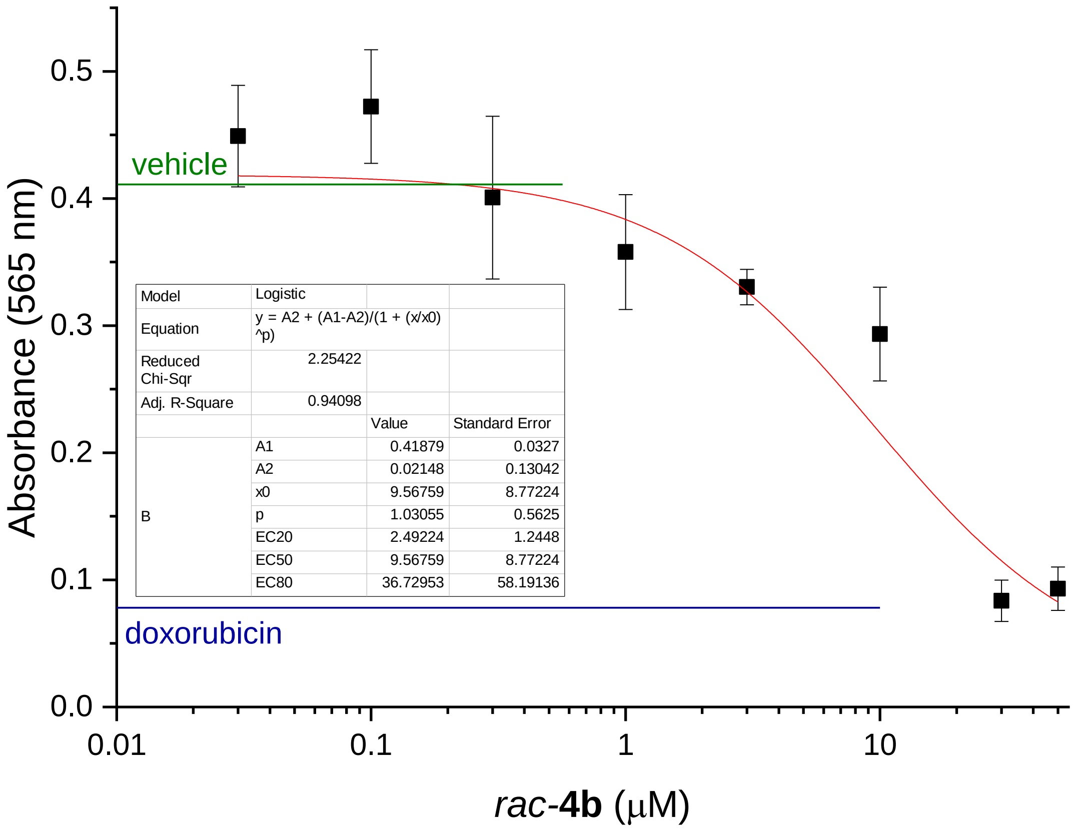

Figure S278. Concentration-dependent effect of *rac*-**4b** on the viability of HaCaT keratinocytes. Cells were treated daily for 3 days, subjected for MTT assay and IC_{50} value was determined as described in the Experimental section. Green line indicates the value of negative control treated with equal amount of vehicle solvent (DMSO) and blue line represents the effect of 1 μg/ml doxorubicin used as positive control. Data are presented as Mean±SEM, N=4 at each data point.

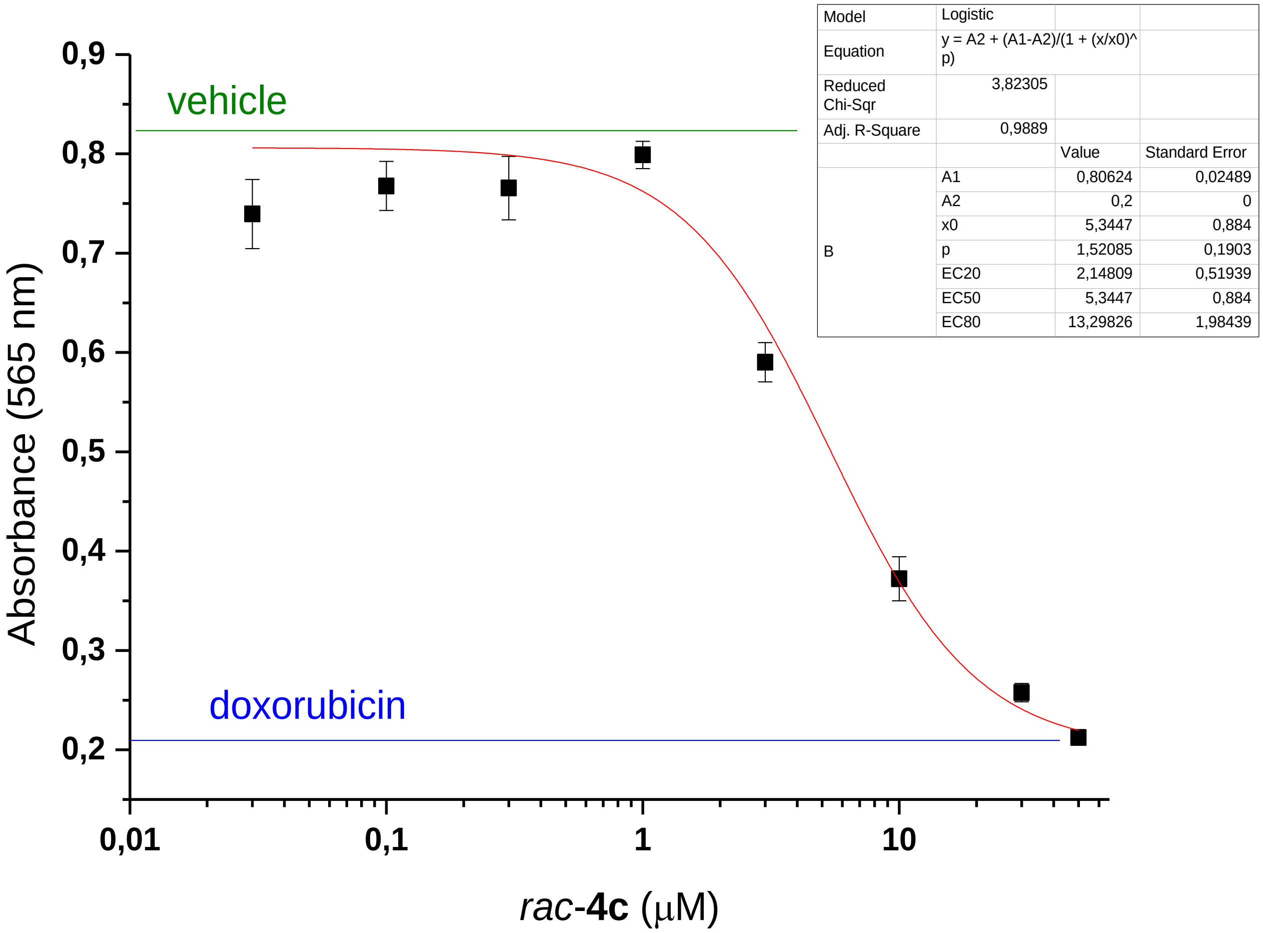

Figure S279. Concentration-dependent effect of *rac*-**4c** on the viability of A2780 cells. Cells were treated daily for 3 days, subjected for MTT assay and IC_{50} value was determined as described in the Experimental section. Green line indicates the value of negative control treated with equal amount of vehicle solvent (DMSO) and blue line represents the effect of 1 µg/ml doxorubicin used as positive control. Data are presented as Mean ±SEM, N=4 at each data point.

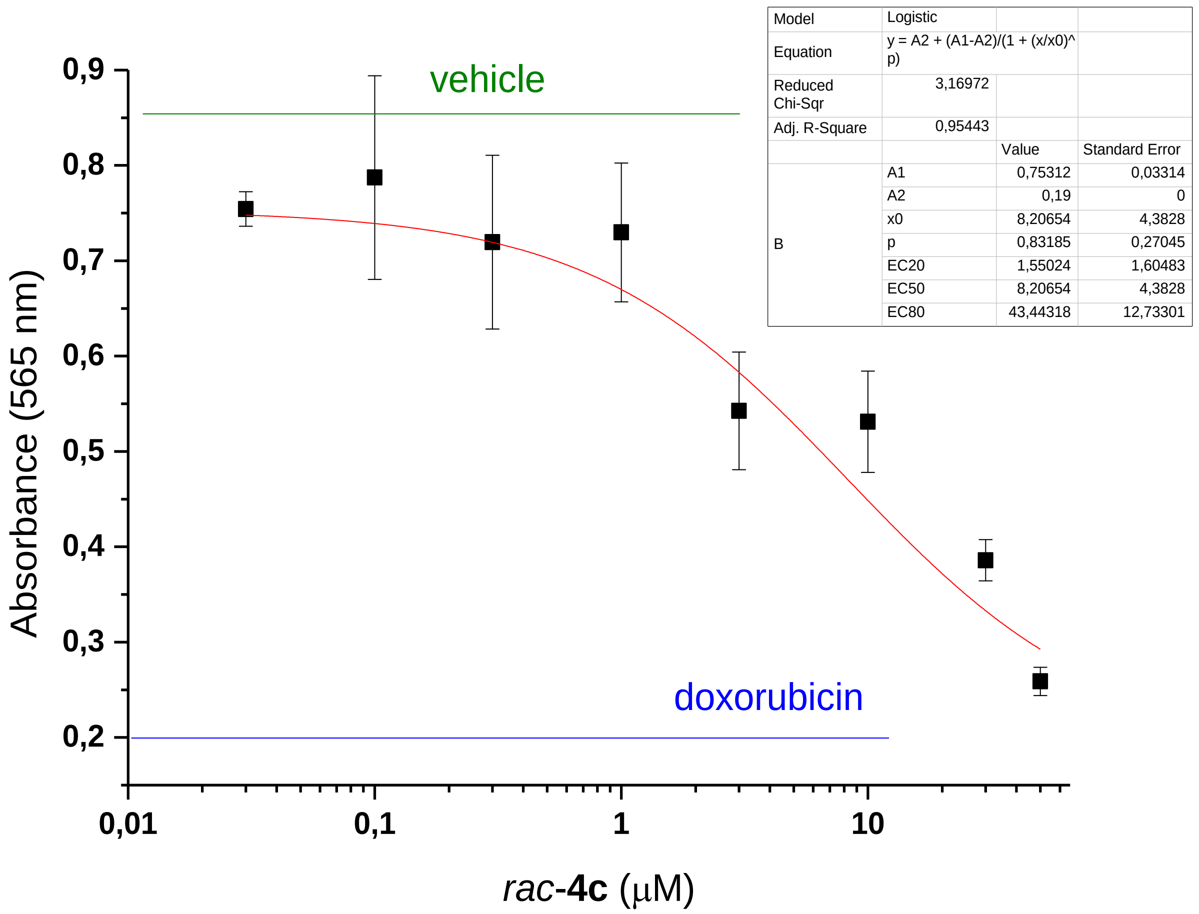

Figure S280. Concentration-dependent effect of *rac*-**4c** on the viability of WM35 cells. Cells were treated daily for 3 days, subjected for MTT assay and IC_{50} value was determined as described in the Experimental section. Green line indicates the value of negative control treated with equal amount of vehicle solvent (DMSO) and blue line represents the effect of 1 μg/ml doxorubicin used as positive control. Data are presented as Mean ±SEM, N=4 at each data point.

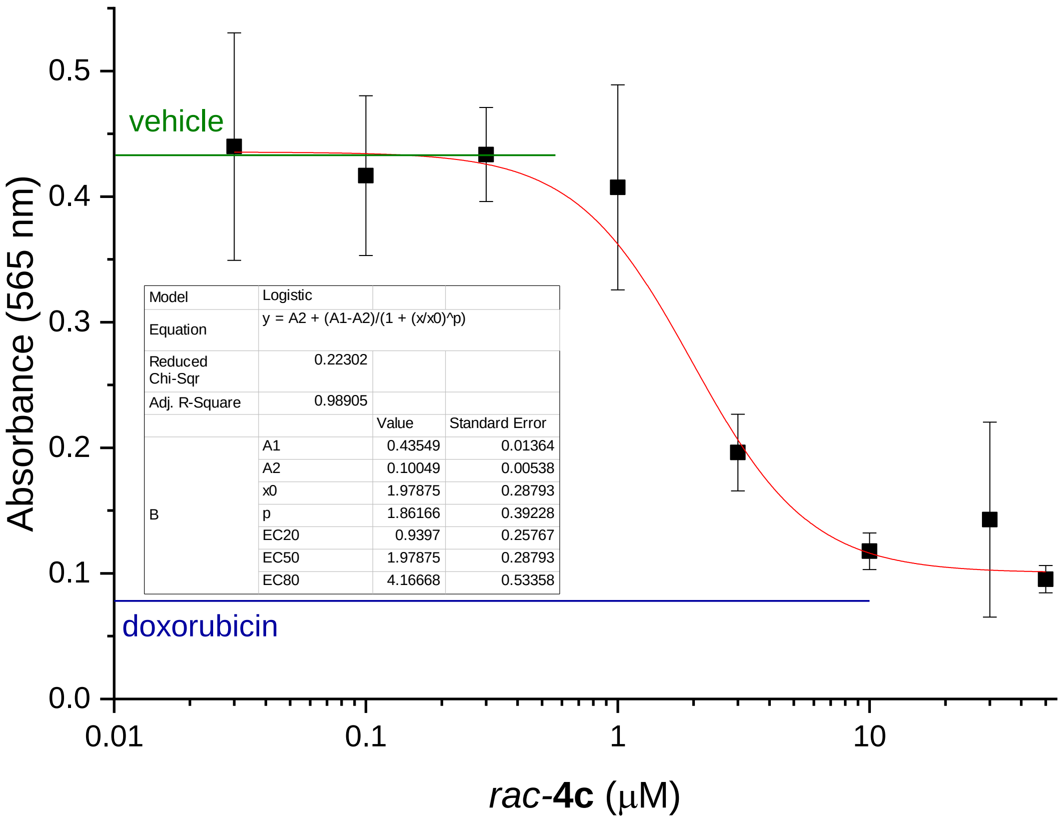

Figure S281. Concentration-dependent effect of *rac*-**4c** on the viability of HaCaT keratinocytes. Cells were treated daily for 3 days, subjected for MTT assay and IC_{50} value was determined as described in the Experimental section. Green line indicates the value of negative control treated with equal amount of vehicle solvent (DMSO) and blue line represents the effect of 1 μg/ml doxorubicin used as positive control. Data are presented as Mean ±SEM, N=4 at each data point.

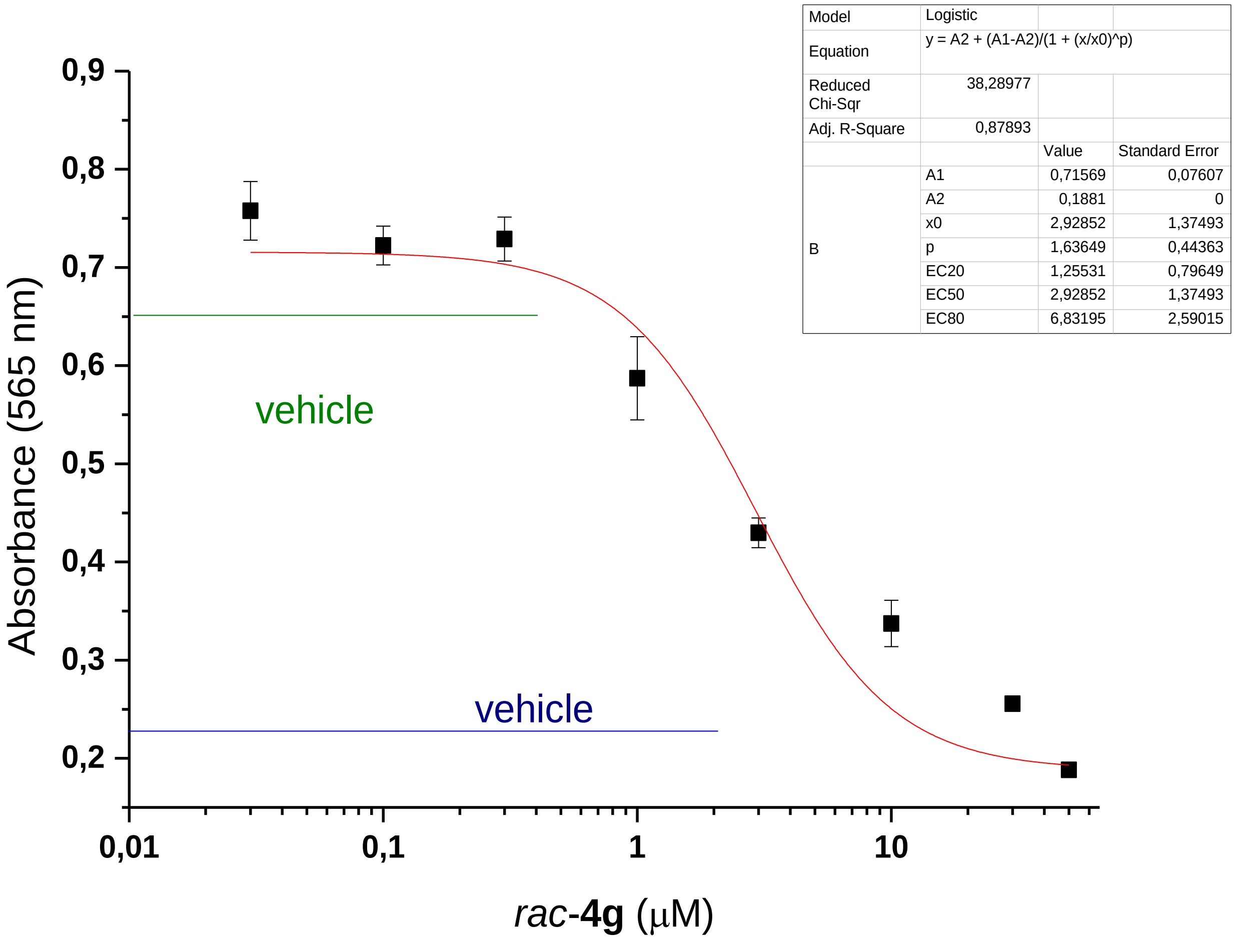

Figure S282. Concentration-dependent effect of *rac*-**4g** on the viability of A2780 cells. Cells were treated daily for 3 days, subjected for MTT assay and IC_{50} value was determined as described in the Experimental section. Green line indicates the value of negative control treated with equal amount of vehicle solvent (DMSO) and blue line represents the effect of 1 μg/ml doxorubicin used as positive control. Data are presented as Mean ±SEM, N=4 at each data point.

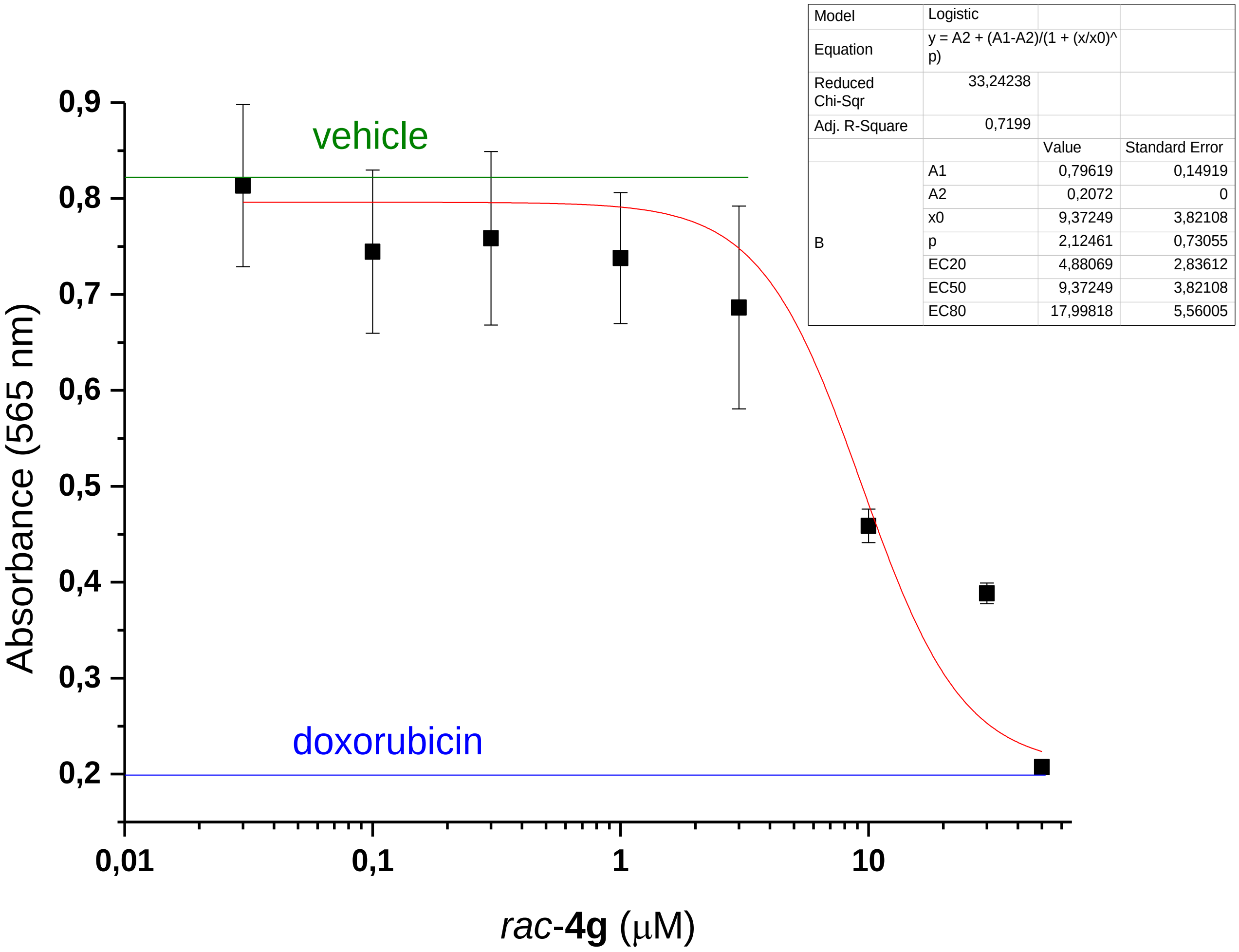

Figure S283. Concentration-dependent effect of *rac*-**4g** on the viability of WM35 cells. Cells were treated daily for 3 days, subjected for MTT assay and IC_{50} value was determined as described in the Experimental section. Green line indicates the value of negative control treated with equal amount of vehicle solvent (DMSO) and blue line represents the effect of 1 μg/ml doxorubicin used as positive control. Data are presented as Mean ±SEM, N=4 at each data point.

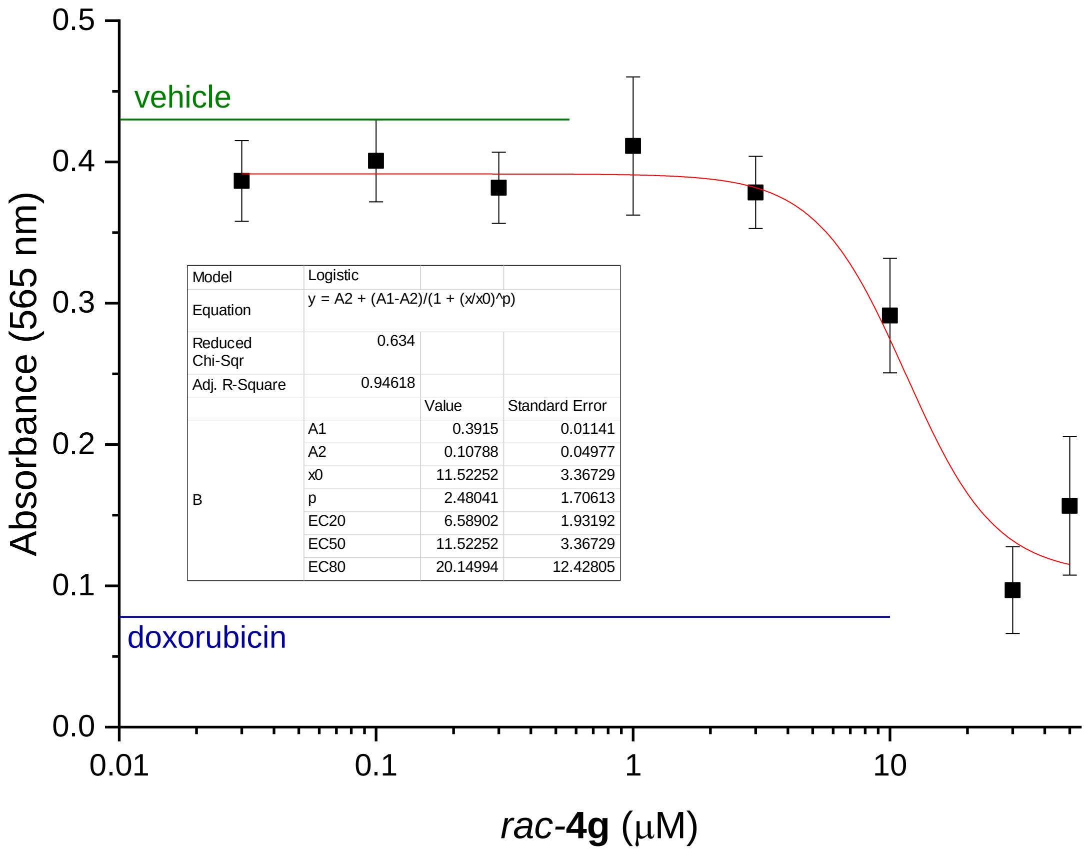

Figure S284. Concentration-dependent effect of *rac*-**4g** on the viability of HaCaT keratinocytes. Cells were treated daily for 3 days, subjected for MTT assay and IC_{50} value was determined as described in the Experimental section. Green line indicates the value of negative control treated with equal amount of vehicle solvent (DMSO) and blue line represents the effect of 1 µg/ml doxorubicin used as positive control. Data are presented as Mean ±SEM, N=4 at each data point.

2 Chiral HPLC-ECD analysis

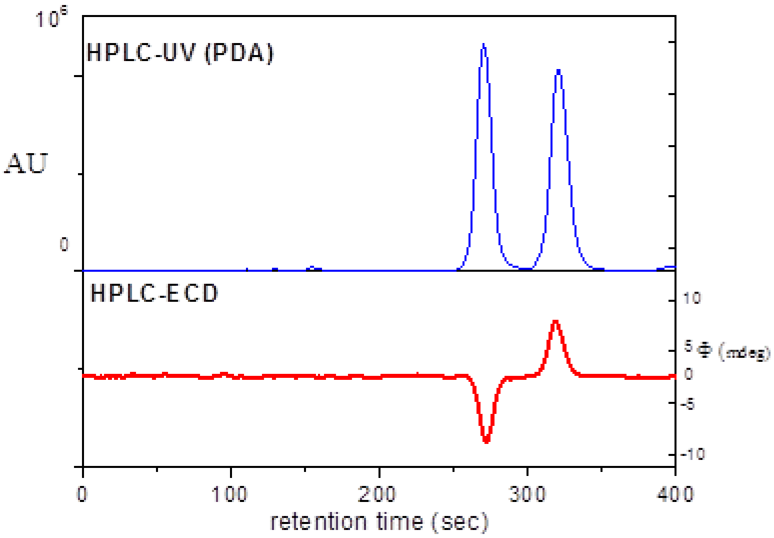

Figure S285. HPLC-UV and –ECD traces of *rac*-**20a** on Chiralpak IA column with hexane/2-propanol 80:20 eluent monitored at 230 nm.

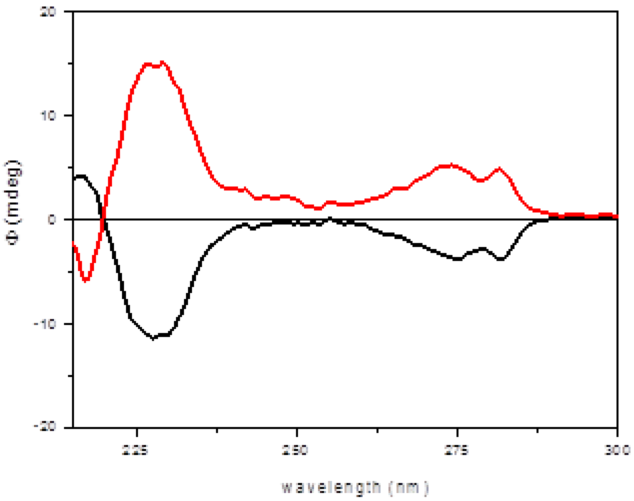

Figure S286. HPLC-ECD spectra of the first- [(4a*S*,5*R*,10b*R*), black] and second-eluting [(4a*R*,5*S*,10b*S*), red] enantiomers of *rac*-**20a**.

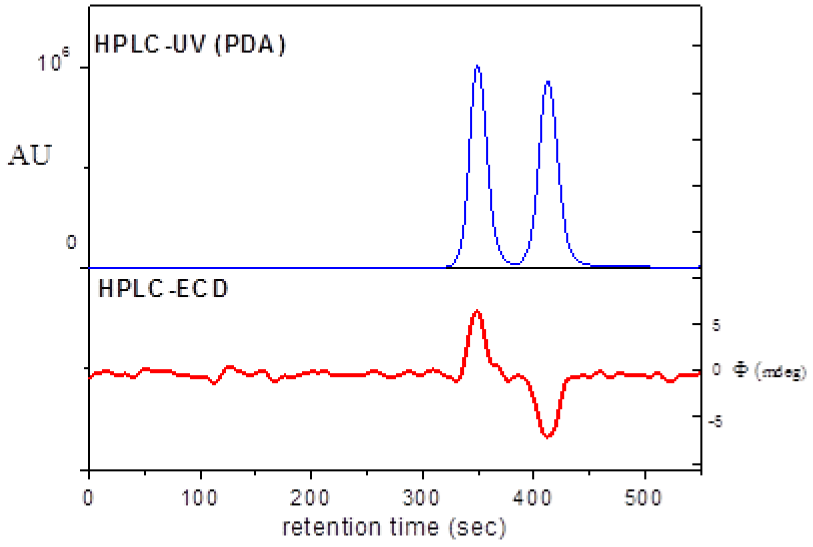

Figure S287. HPLC-UV and –ECD traces of *rac*-**20b** on Chiralpak IA column with hexane/2-propanol 80:20 eluent monitored at 230 nm.

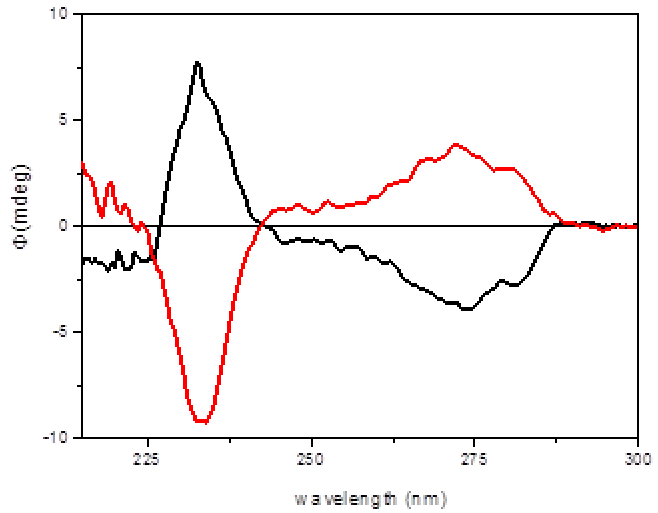

Figure S288. HPLC-ECD spectra of the first- [(4a*S*,5*R*,10b*R*), black] and second-eluting [(4a*R*,5*S*,10b*S*), red] enantiomers of *rac*-**20b**.

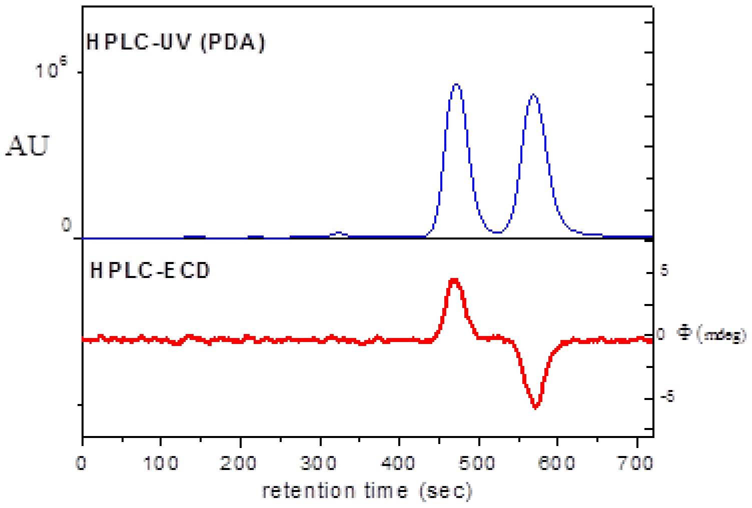

Figure S289. HPLC-UV and –ECD traces of *rac*-**20c** on Chiralpak IA column with hexane/2-propanol 80:20 eluent monitored at 230 nm.

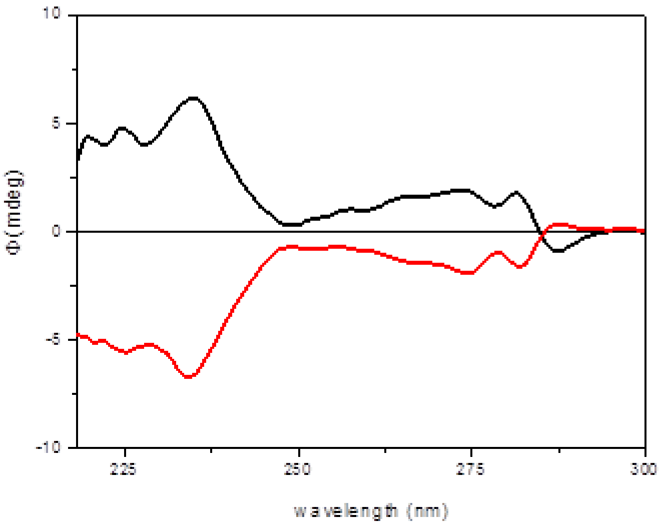

Figure S290. HPLC-ECD spectra of the first- [(4a*R*,5*S*,10b*S*), black] and second-eluting [(4a*S*,5*R*,10b*R*), red] enantiomers of *rac*-**20c**.

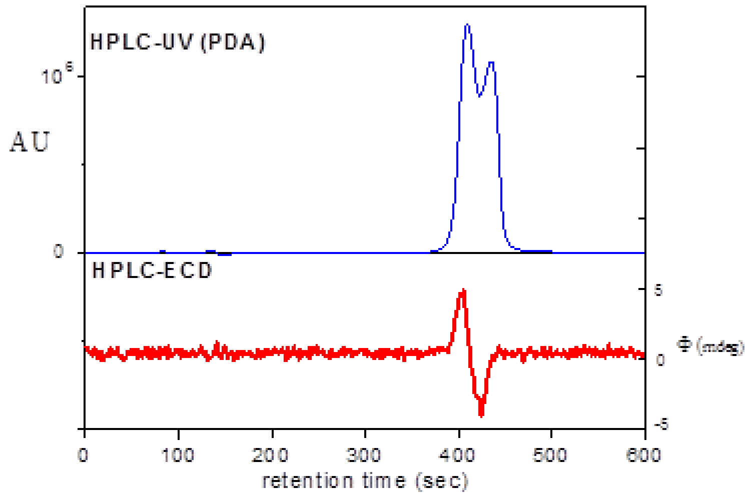

Figure S291. HPLC-UV and –ECD traces of *rac*-**20d** on Chiralpak IA column with hexane/2-propanol 80:20 eluent monitored at 280 nm.

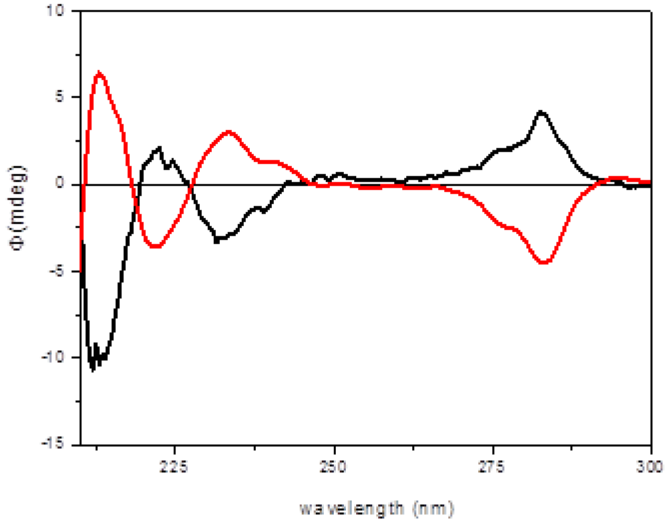

Figure S292. HPLC-ECD spectra of the first- [(4a*R*,5*S*,10b*S*), black] and second-eluting [(4a*S*,5*R*,10b*R*), red] enantiomers of *rac*-**20d**.

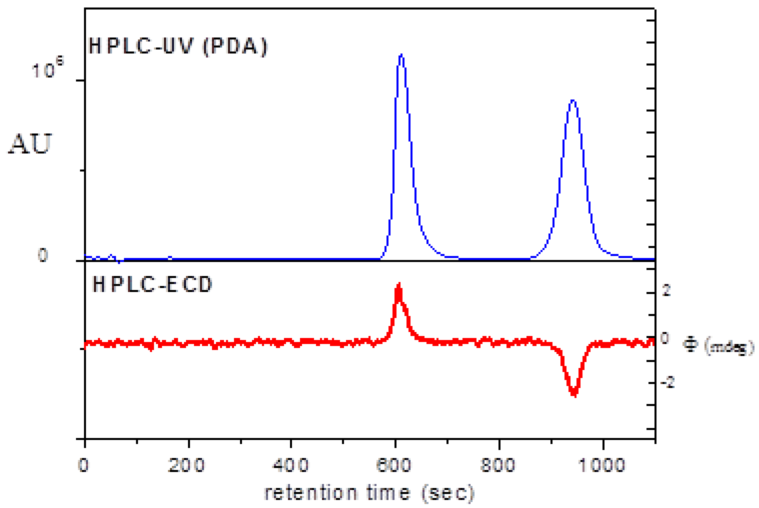

Figure S293. HPLC-UV and –ECD traces of *rac*-**20e** on Chiralpak IA column with hexane/2-propanol 80:20 eluent monitored at 280 nm.

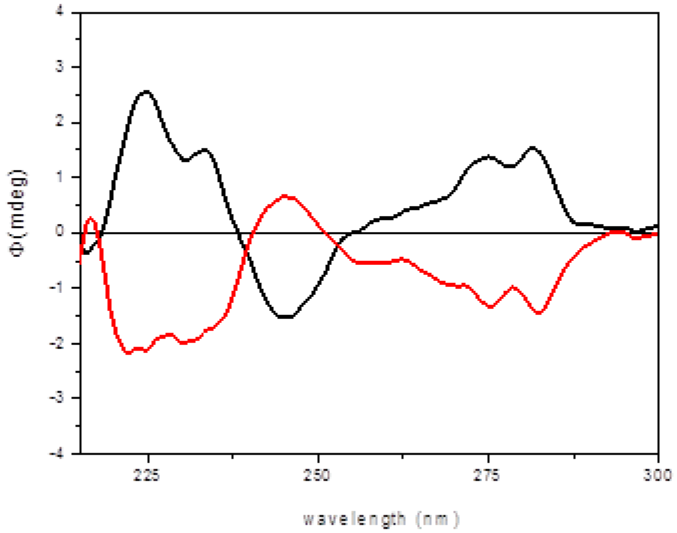

Figure S294. HPLC-ECD spectra of the first- [(4a*R*,5*S*,10b*S*), black] and second-eluting [(4a*S*,5*R*,10b*R*), red] enantiomers of *rac*-**20e**.

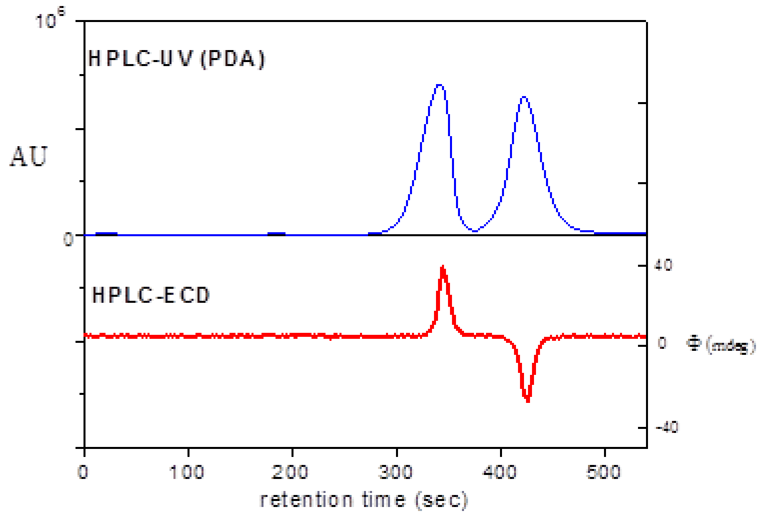

Figure S295. HPLC-UV and –ECD traces of *rac*-**20f** on Chiralpak IA column with hexane/2-propanol 80:20 eluent monitored at 225 nm.

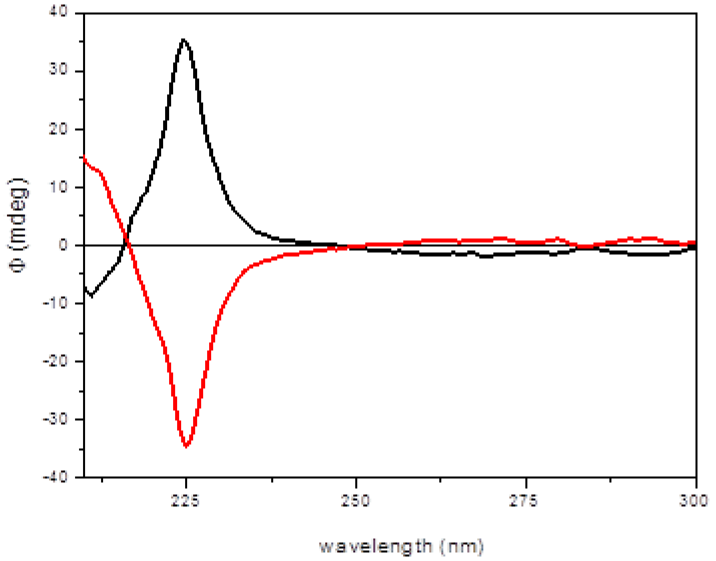

Figure S296. HPLC-ECD spectra of the first- [(4a*S*,5*R*,10b*R*), black] and second-eluting [(4a*R*,5*S*,10b*S*), red] enantiomers of *rac*-**20f**.

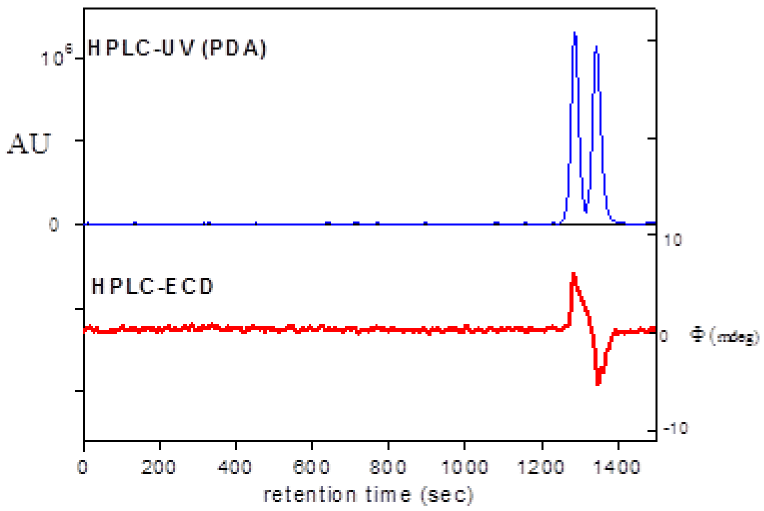

Figure S297. HPLC-UV and –ECD traces of *rac*-**20g** on Chiralpak IA column with hexane/2-propanol 90:10 eluent monitored at 225 nm.

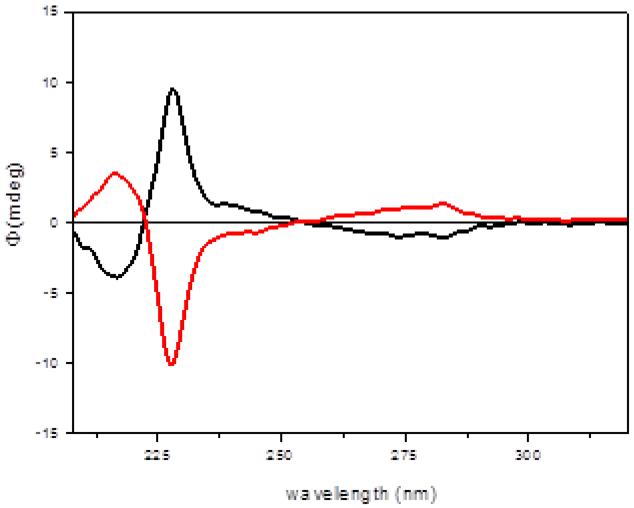

Figure S298. HPLC-ECD spectra of the first- [(4a*S*,5*R*,10b*R*), black] and second-eluting [(4a*R*,5*S*,10b*S*), red] enantiomers of *rac*-**20g**.

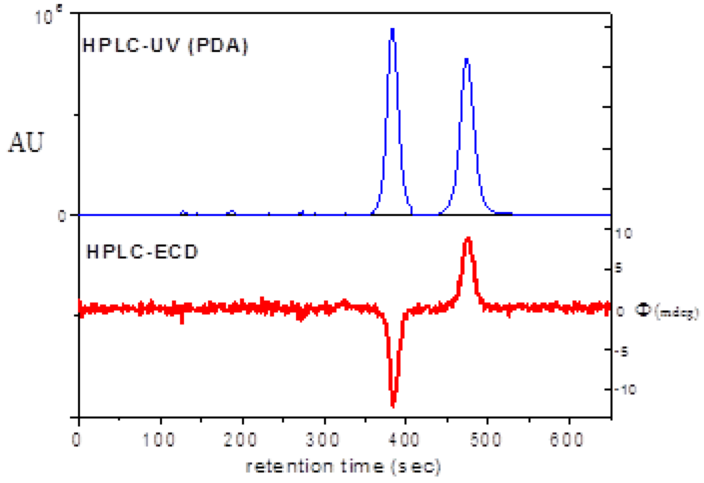

Figure S299. HPLC-UV and –ECD traces of *rac*-**23a** on Chiralpak IA column with hexane/2-propanol 80:20 eluent monitored at 230 nm.

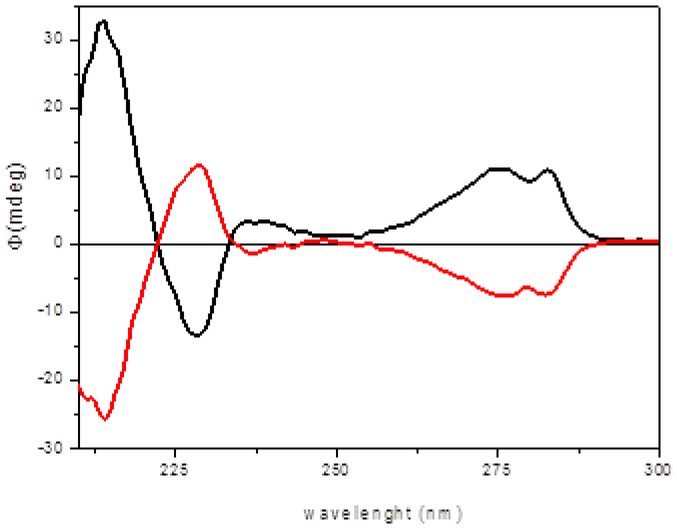

Figure S300. HPLC-ECD spectra of the first- [(4a*S*,5*S*,10b*S*), black] and second-eluting [(4a*R*,5*R*,10b*R*), red] enantiomers of *rac*-**23a**.

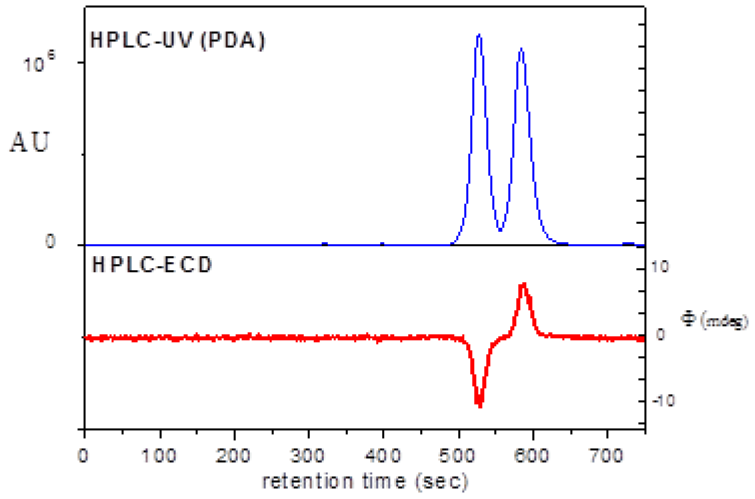

Figure S301. HPLC-UV and –ECD traces of *rac*-**23b** on Chiralpak IA column with hexane/2-propanol 80:20 eluent monitored at 270 nm.

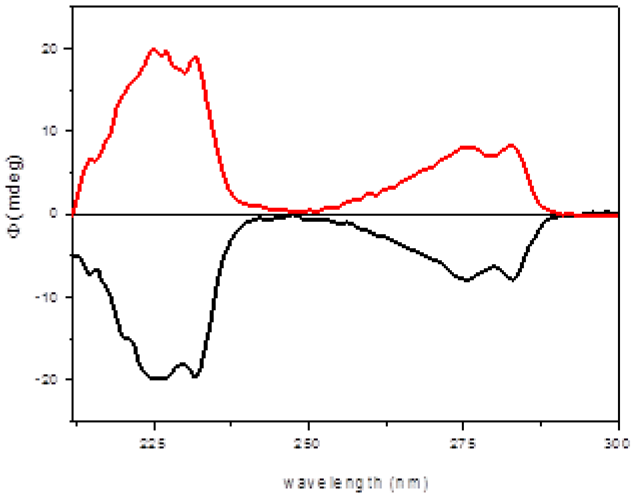

Figure S2302. HPLC-ECD spectra of the first- [(4a*R*,5*R*,10b*R*), black] and second-eluting [(4a*S*,5*S*,10b*S*), red] enantiomers of *rac*-**23b**.

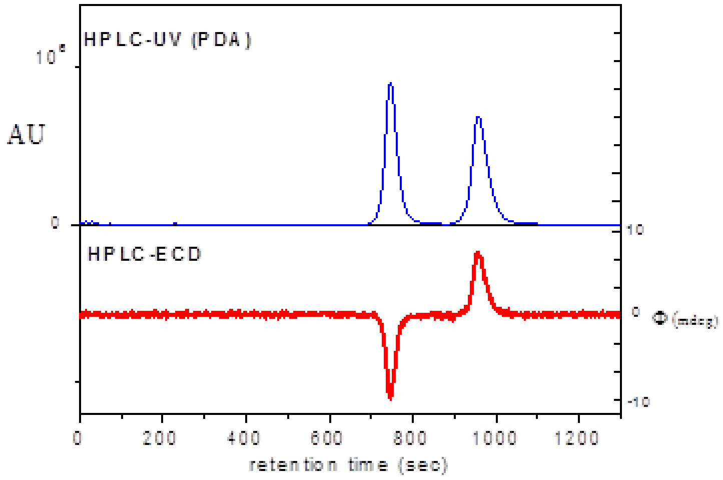

Figure S303. HPLC-UV and –ECD traces of *rac*-**23c** on Chiralpak IA column with hexane/2-propanol 80:20 eluent monitored at 270 nm.

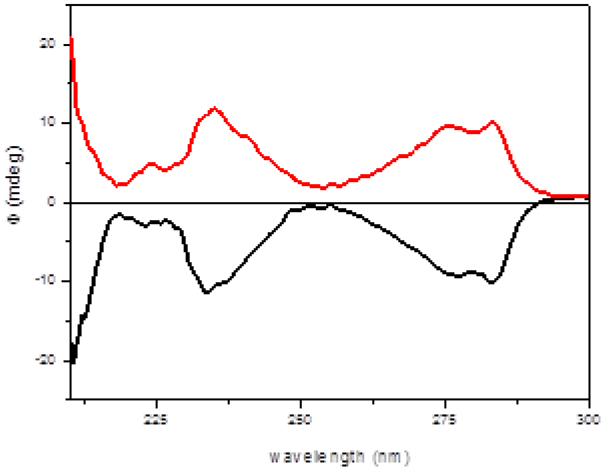

Figure S304. HPLC-ECD spectra of the first- [(4a*R*,5*R*,10b*R*), black] and second-eluting [(4a*S*,5*S*,10b*S*), red] enantiomers of *rac*-**23c**.

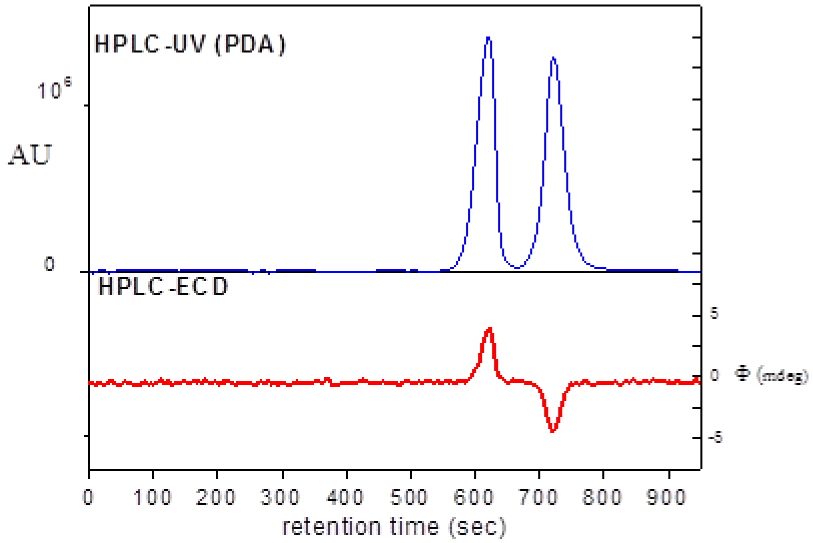

Figure S305. HPLC-UV and –ECD traces of *rac*-**23d** on Chiralpak IA column with hexane/2-propanol 80:20 eluent monitored at 270 nm.

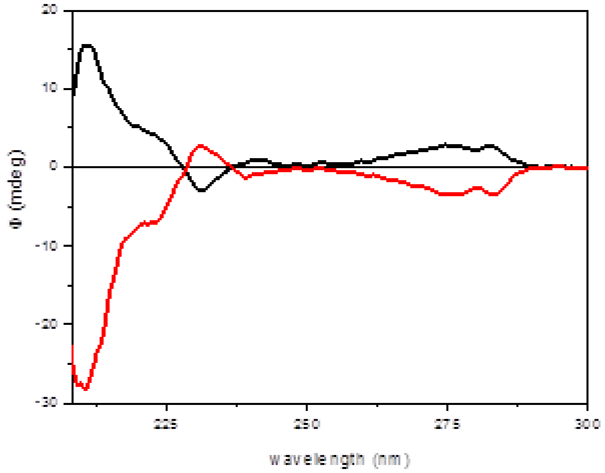

Figure S306. HPLC-ECD spectra of the first- [(4a*S*,5*S*,10b*S*), black] and second-eluting [(4a*R*,5*R*,10b*R*), red] enantiomers of *rac*-**23d**.

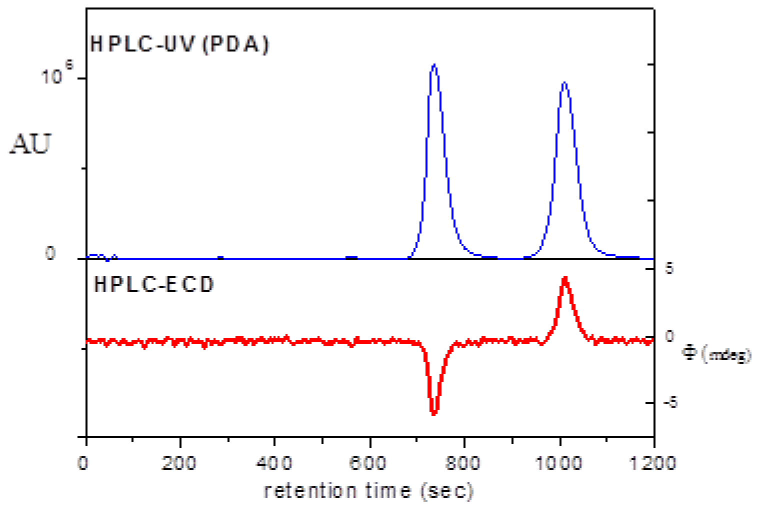

Figure S307. HPLC-UV and –ECD traces of *rac*-**23e** on Chiralpak IA column with hexane/2-propanol 80:20 eluent monitored at 280 nm.

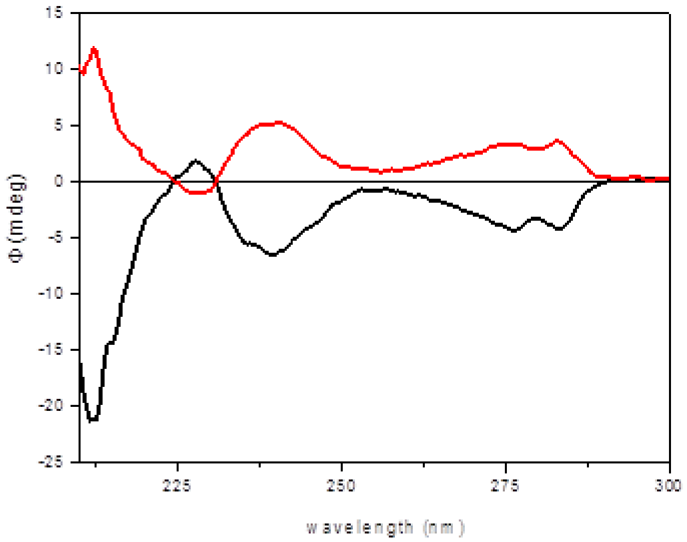

Figure S308. HPLC-ECD spectra of the first- [(4a*R*,5*R*,10b*R*), black] and second-eluting [(4a*S*,5*S*,10b*S*), red] enantiomers of *rac*-**23e**.

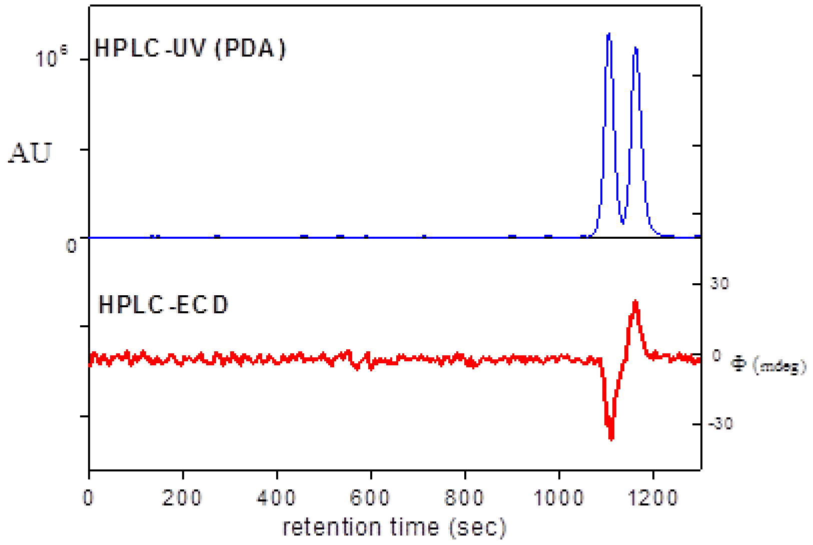

Figure S309. HPLC-UV and –ECD traces of *rac*-**23g** on Chiralpak IA column with hexane/2-propanol 80:20 eluent monitored at 230 nm.

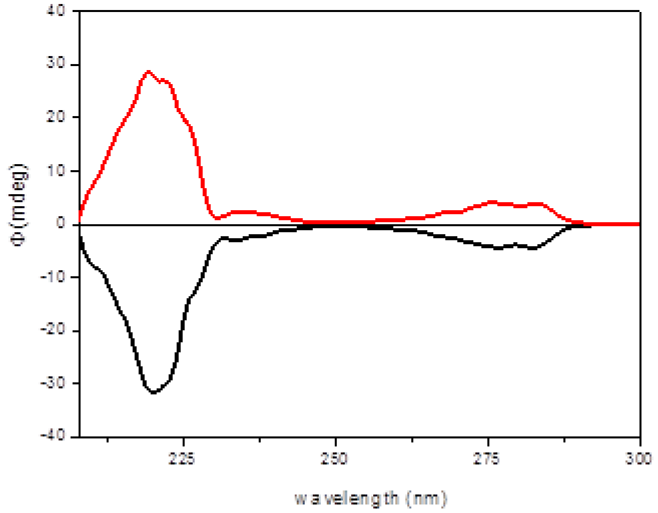

Figure S310. HPLC-ECD spectra of the first- [(4a*R*,5*R*,10b*R*), black] and second-eluting [(4a*S*,5*S*,10b*S*), red] enantiomers of *rac*-**23g**.

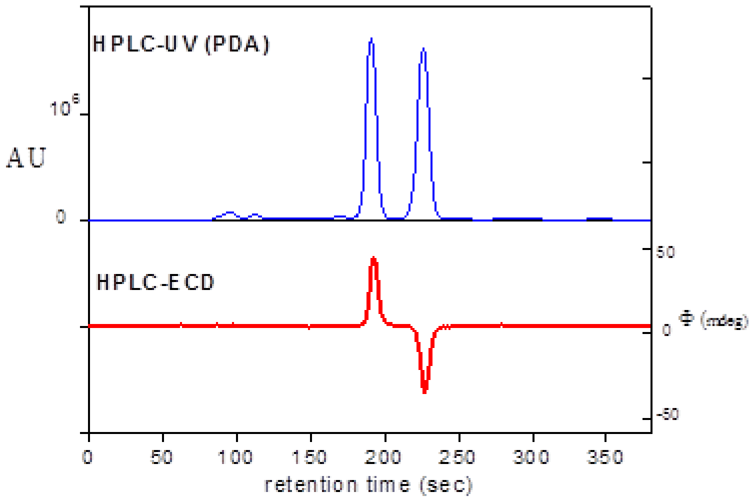

Figure S311. HPLC-UV and –ECD traces of *rac*-**3a** on Chiralpak IA column with hexane/2-propanol 80:20 eluent monitored at 240 nm.

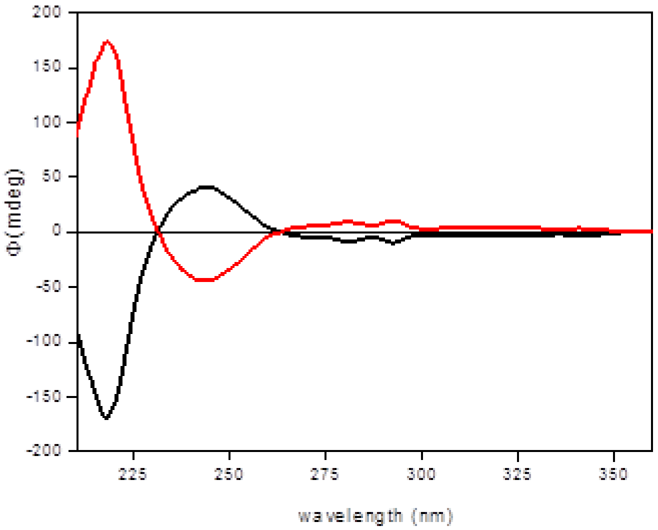

Figure S312. HPLC-ECD spectra of the first- [(4*R*), black] and second-eluting [(4*S*), red] enantiomers of *rac*-**3a**.

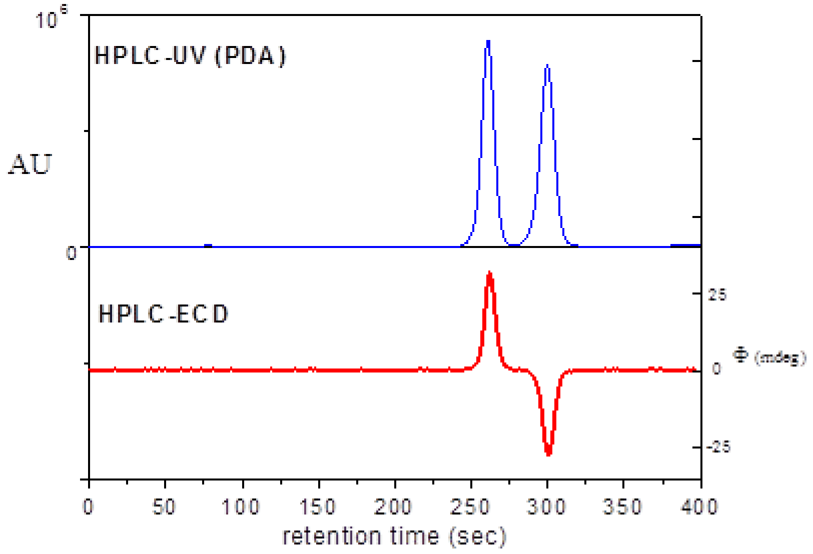

Figure S313. HPLC-UV and –ECD traces of *rac*-**3b** on Chiralpak IA column with hexane/2-propanol 80:20 eluent monitored at 250 nm.

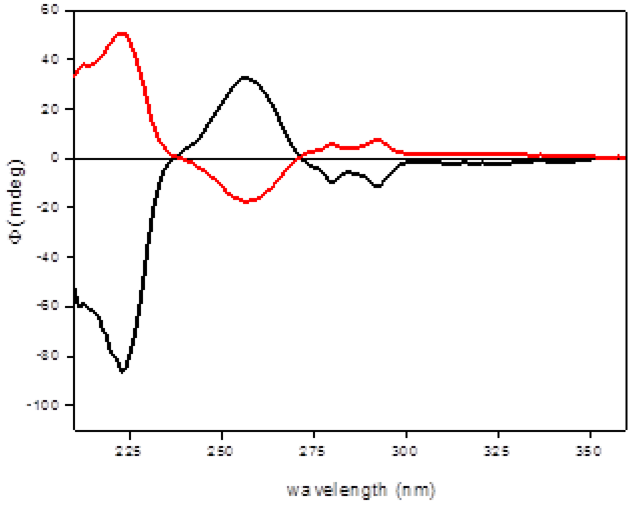

Figure S314. HPLC-ECD spectra of the first- [(4*R*), black] and second-eluting [(4*S*), red] enantiomers of *rac*-**3b**.

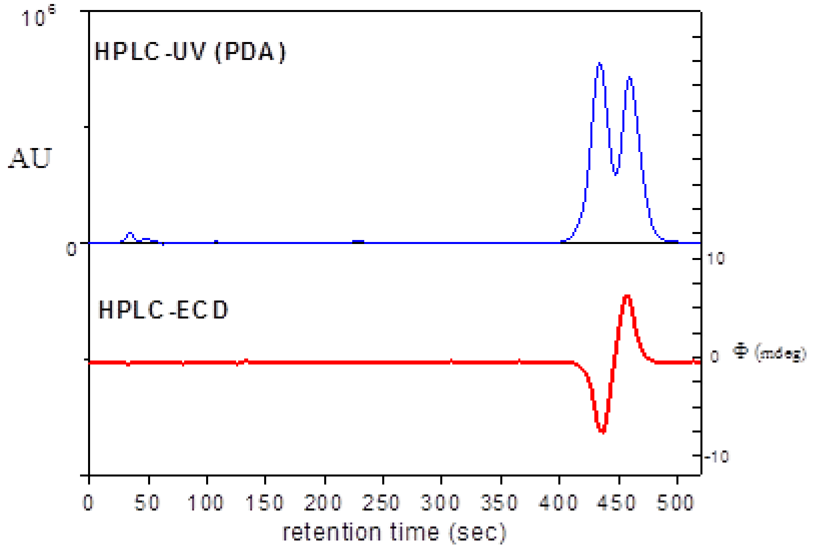

Figure S315. HPLC-UV and –ECD traces of *rac*-**3c** on Chiralpak IA column with hexane/2-propanol 80:20 eluent monitored at 240 nm.

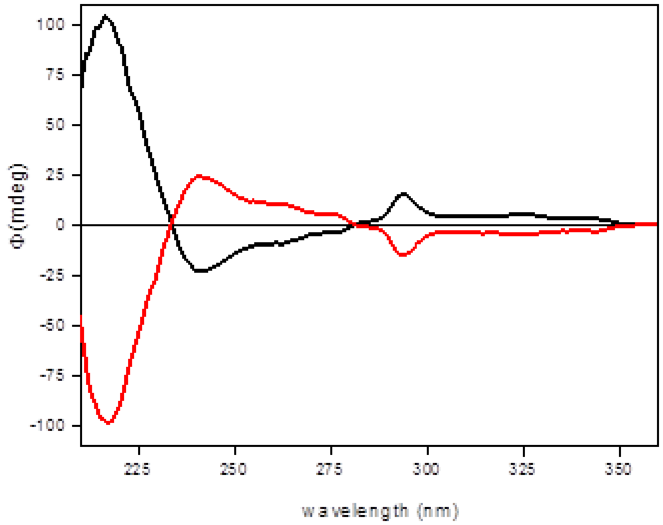

Figure S316. HPLC-ECD spectra of the first- [(4*S*), black] and second-eluting [(4*R*), red] enantiomers of *rac*-**3c**.

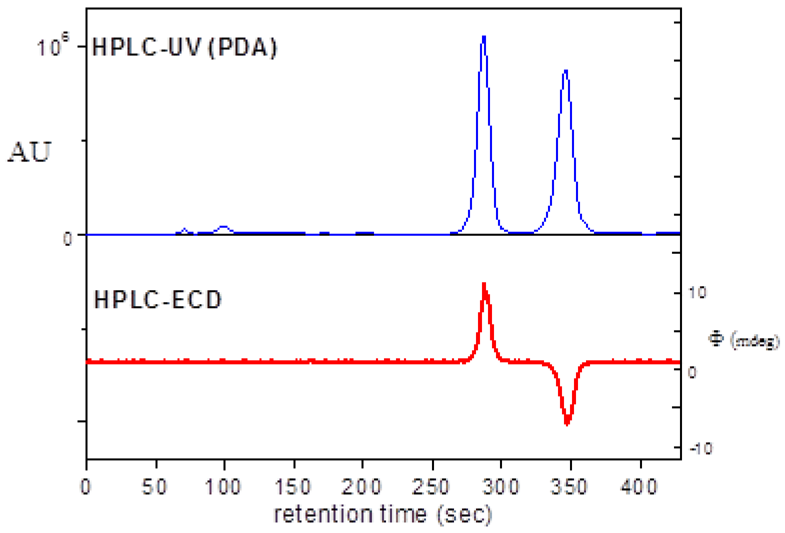

Figure S317. HPLC-UV and –ECD traces of *rac*-**3d** on Chiralpak IA column with hexane/2-propanol 80:20 eluent monitored at 240 nm.

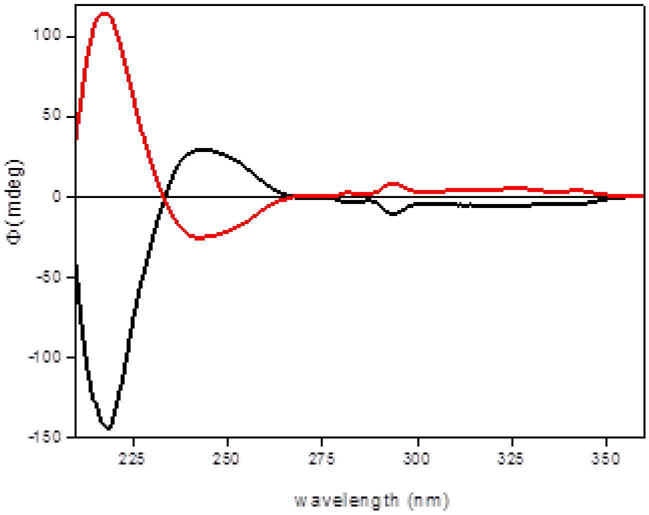

Figure S318. HPLC-ECD spectra of the first- [(4*R*), black] and second-eluting [(4*S*), red] enantiomers of *rac*-**3d**.

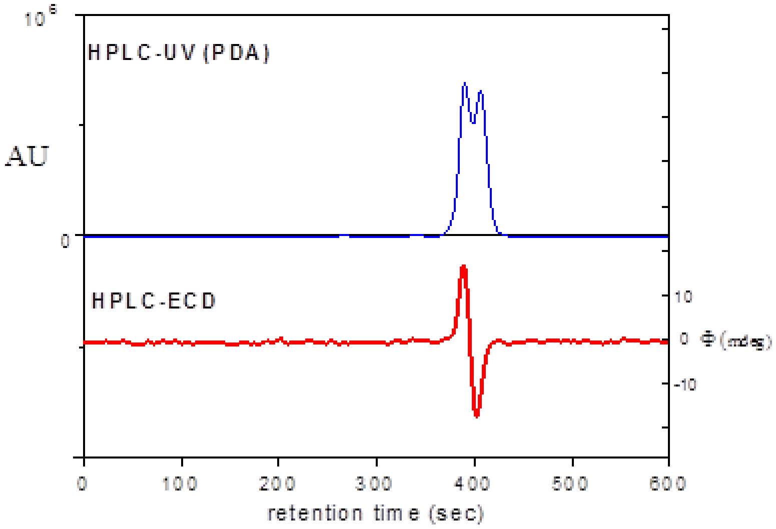

Figure S319. HPLC-UV and –ECD traces of *rac*-**3e** on Chiralpak IA column with hexane/2-propanol 80:20 eluent monitored at 240 nm.

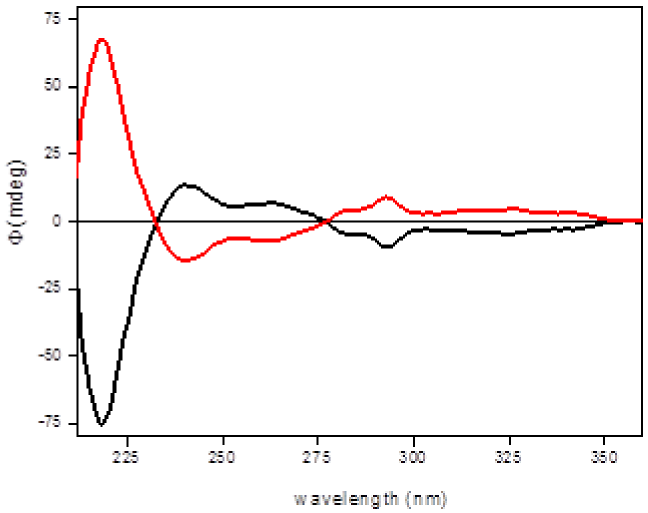

Figure S320. HPLC-ECD spectra of the first- [(4*R*), black] and second-eluting [(4*S*), red] enantiomers of *rac*-**3e**.

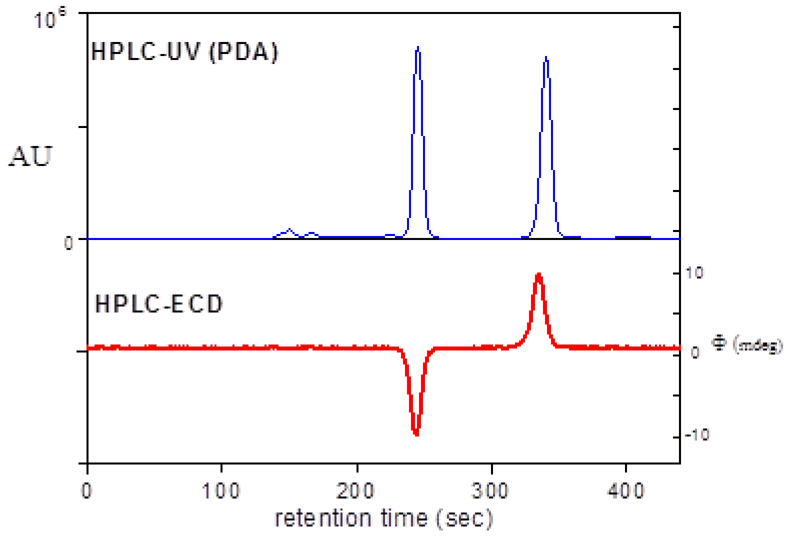

Figure S321. HPLC-UV and –ECD traces of *rac*-**3f** on Chiralpak IA column with hexane/2-propanol 80:20 eluent monitored at 240 nm.

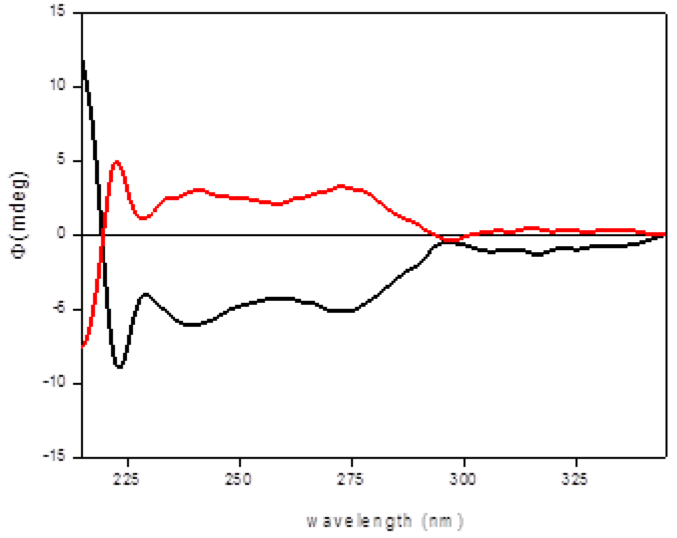

Figure S322. HPLC-ECD spectra of the first- [(4*R*), black] and second-eluting [(4*S*), red] enantiomers of *rac*-**3f**.

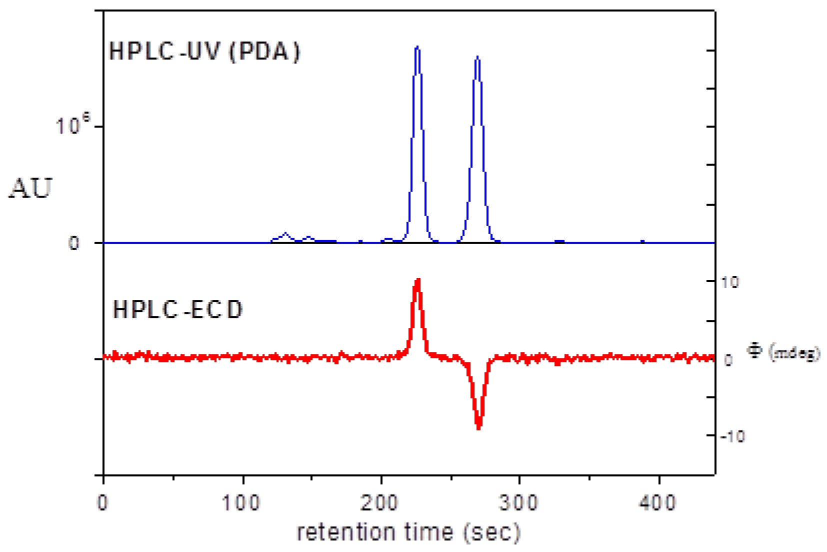

Figure S323. HPLC-UV and –ECD traces of *rac*-**3g** on Chiralpak IA column with hexane/2-propanol 80:20 eluent monitored at 240 nm.

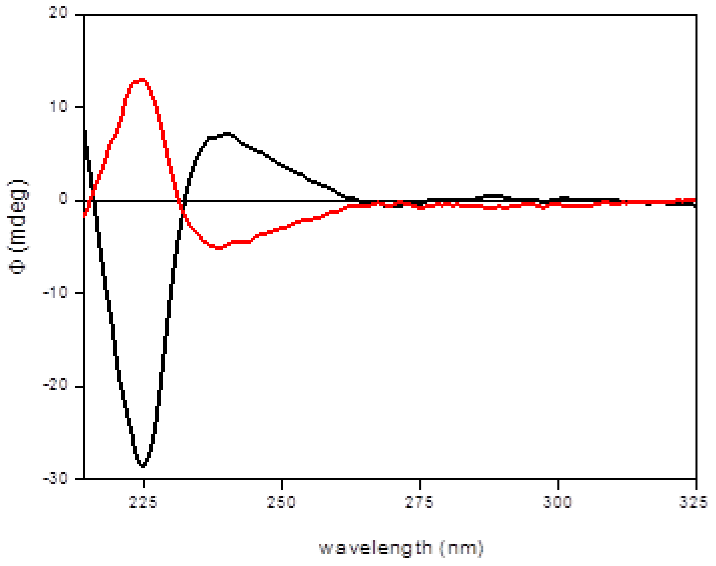

Figure S324. HPLC-ECD spectra of the first- [(4*R*), black] and second-eluting [(4*S*), red] enantiomers of *rac*-**3g**.

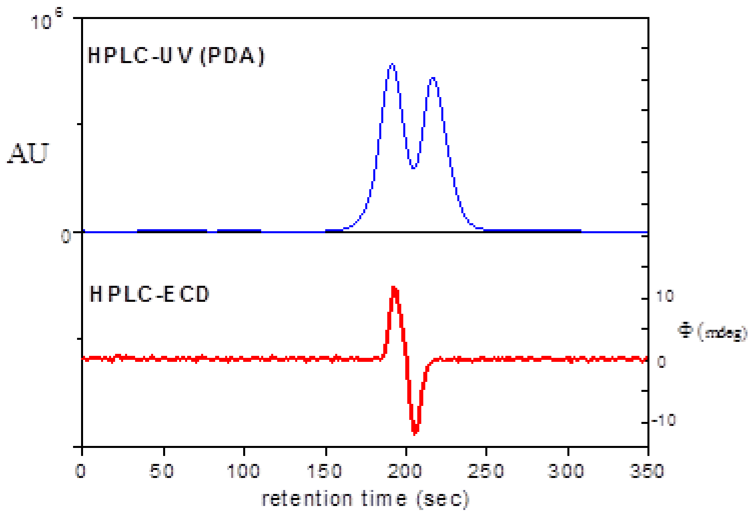

Figure S325. HPLC-UV and –ECD traces of *rac*-**4a** on Chiralpak IA column with hexane/2-propanol 80:20 eluent monitored at 230 nm.

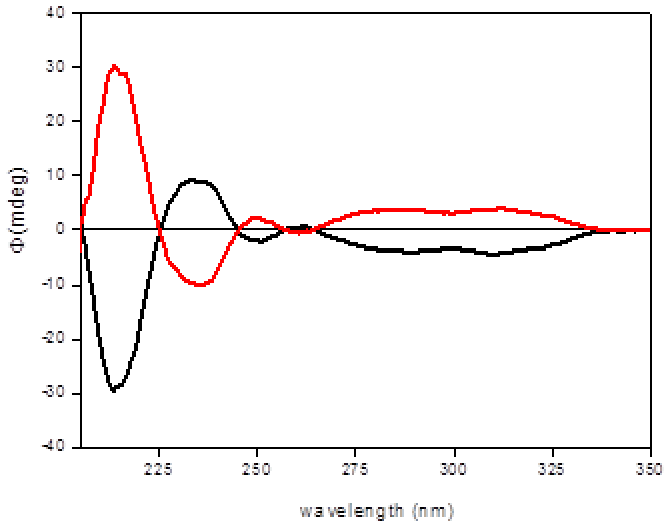

Figure S326. HPLC-ECD spectra of the first- [(4*R*), black] and second-eluting [(4*S*), red] enantiomers of *rac*-**4a**.

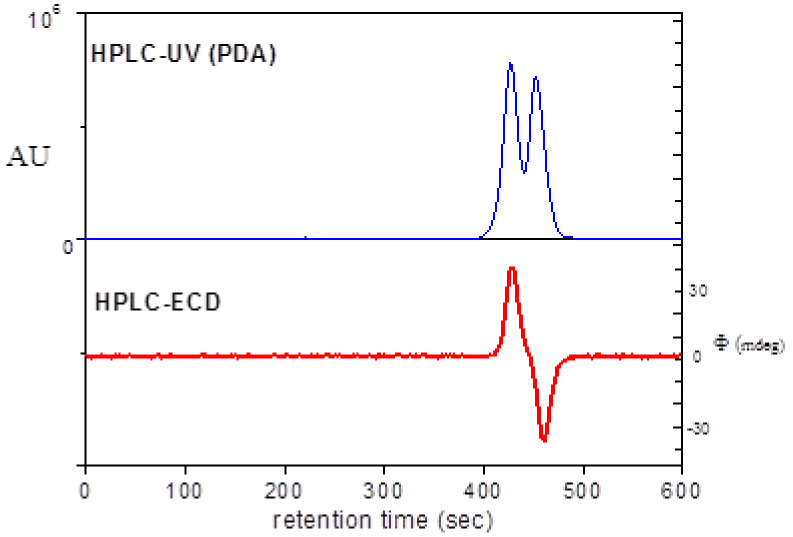

Figure S327. HPLC-UV and –ECD traces of *rac*-**4b** on Chiralpak IA column with hexane/2-propanol 90:10 eluent monitored at 235 nm.

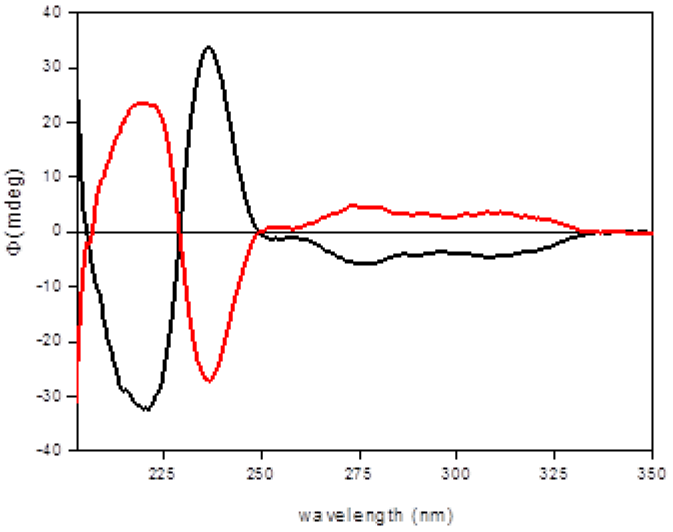

Figure S328. HPLC-ECD spectra of the first- [(4*R*), black] and second-eluting [(4*S*), red] enantiomers of *rac*-**4b**.

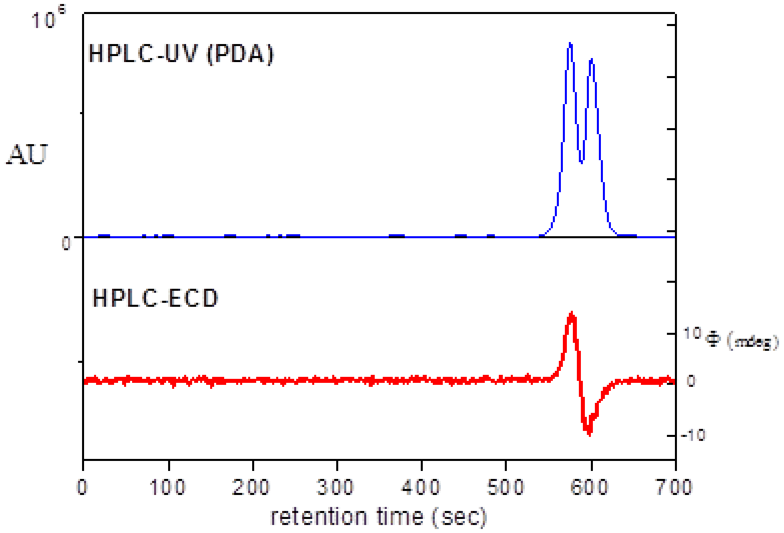

Figure S329. HPLC-UV and –ECD traces of *rac*-**4c** on Chiralpak IA column with hexane/2-propanol 90:10 eluent monitored at 235 nm.

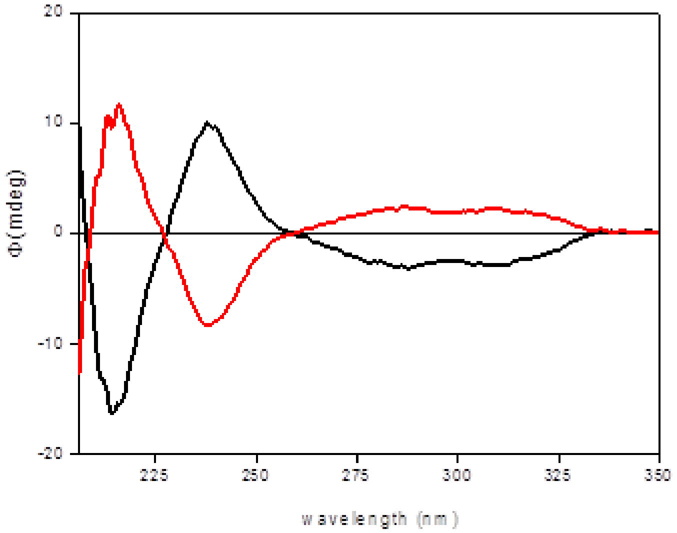

Figure S330. HPLC-ECD spectra of the first- [(4*R*), black] and second-eluting [(4*S*), red] enantiomers of *rac*-**4c**.

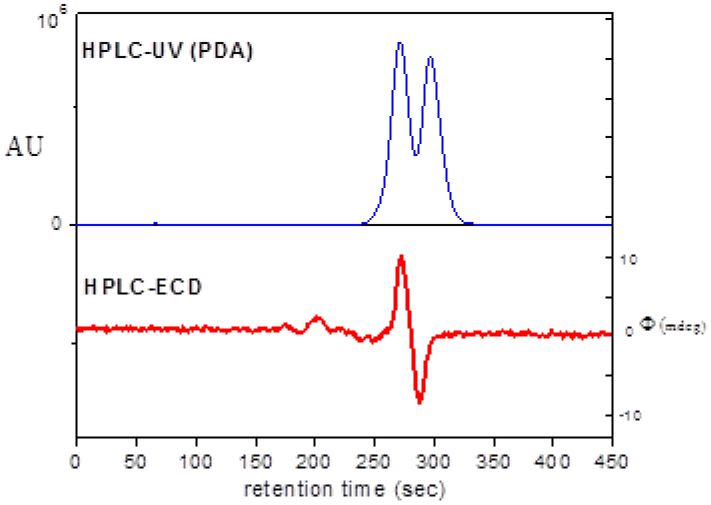

Figure S331. HPLC-UV and –ECD traces of *rac*-**4d** on Chiralpak IA column with hexane/2-propanol 80:20 eluent monitored at 235 nm.

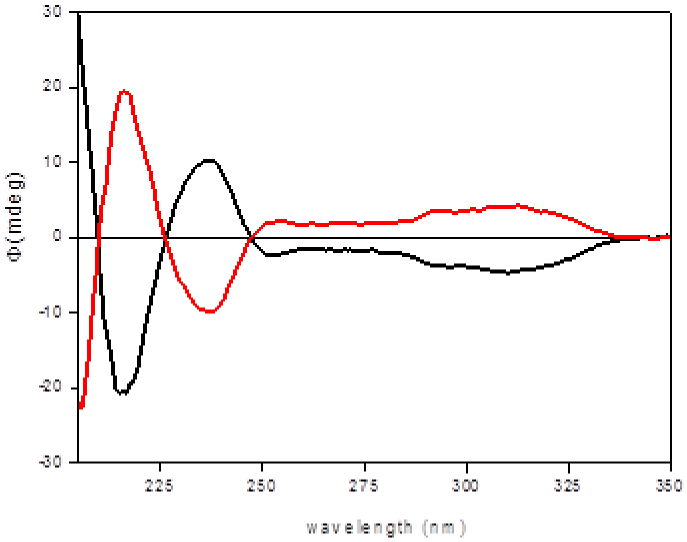

Figure S332. HPLC-ECD spectra of the first- [(4*R*), black] and second-eluting [(4*S*), red] enantiomers of *rac*-**4d**.

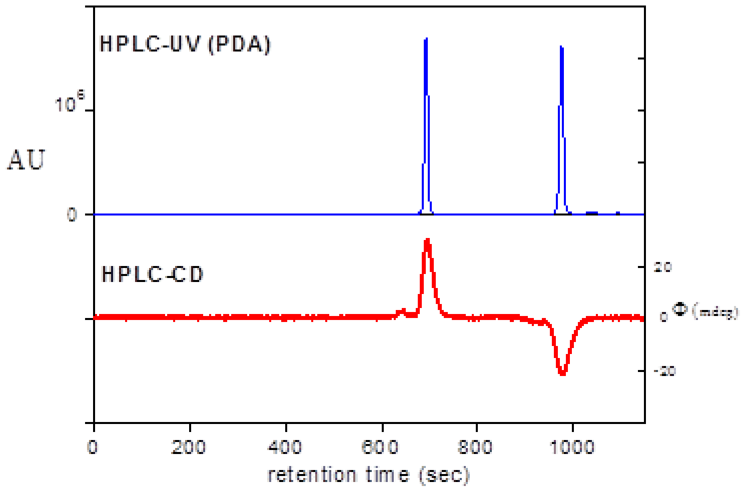

Figure S333. HPLC-UV and –ECD traces of *rac*-**4e** on Chiralpak IC column with hexane/2-propanol 70:30 eluent monitored at 240 nm.

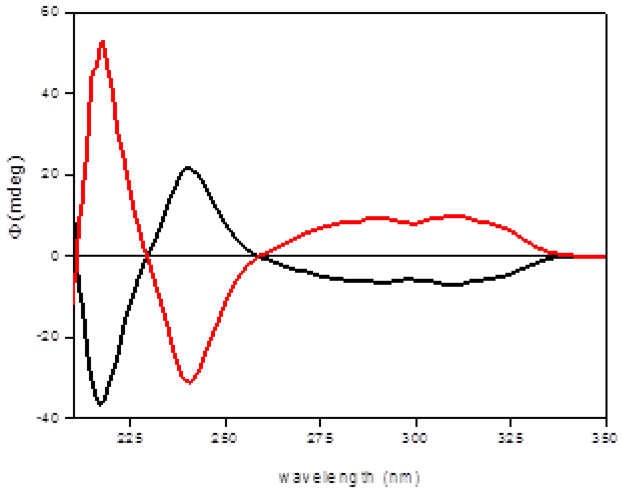

Figure S334. HPLC-ECD spectra of the first- [(4*R*), black] and second-eluting [(4*S*), red] enantiomers of *rac*-**4e**.

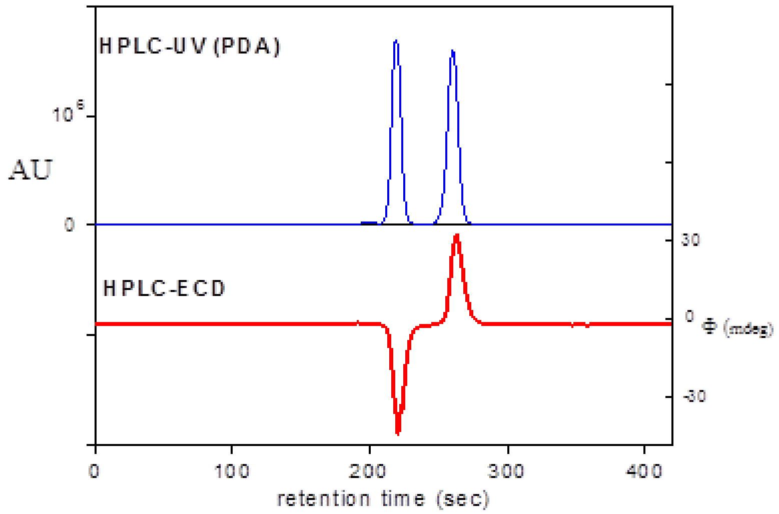

Figure S335. HPLC-UV and –ECD traces of *rac*-**4f** on Chiralpak IA column with hexane/2-propanol 80:20 eluent monitored at 230 nm.

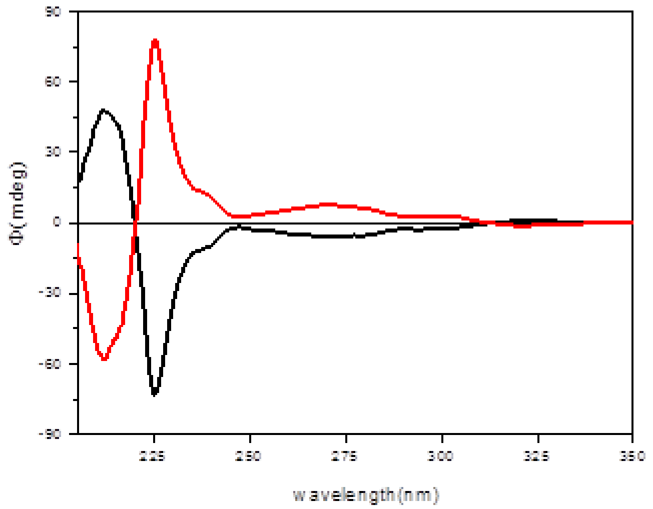

Figure S336. HPLC-ECD spectra of the first- [(4*R*), black] and second-eluting [(4*S*), red] enantiomers of *rac*-**4f**.

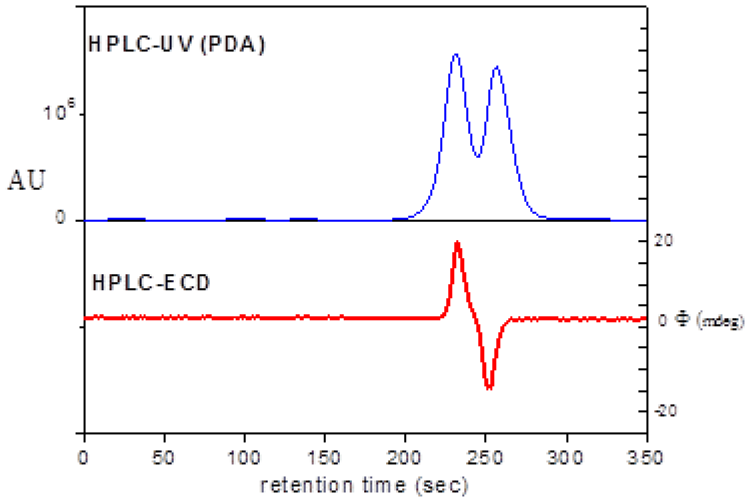

Figure S337. HPLC-UV and –ECD traces of *rac*-**4g** on Chiralpak IA column with hexane/2-propanol 80:20 eluent monitored at 235 nm.

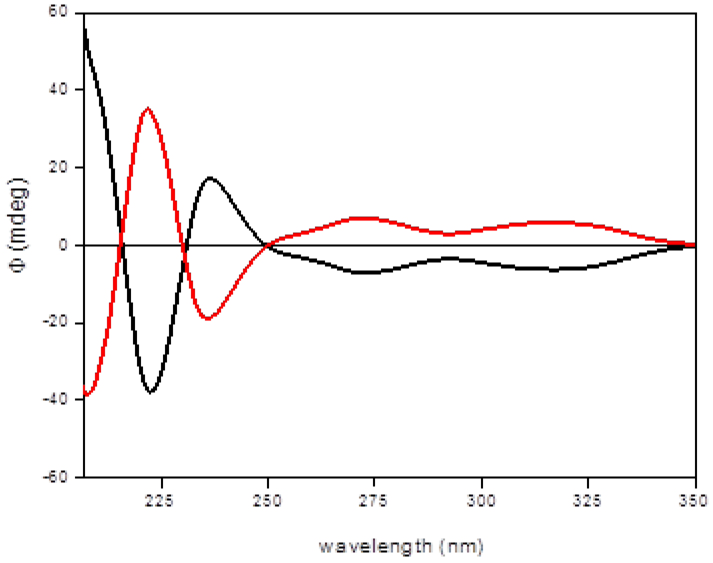

Figure S338. HPLC-ECD spectra of the first- [(4*R*), black] and second-eluting [(4*S*), red] enantiomers of *rac*-**4g**.

3. ECD calculations

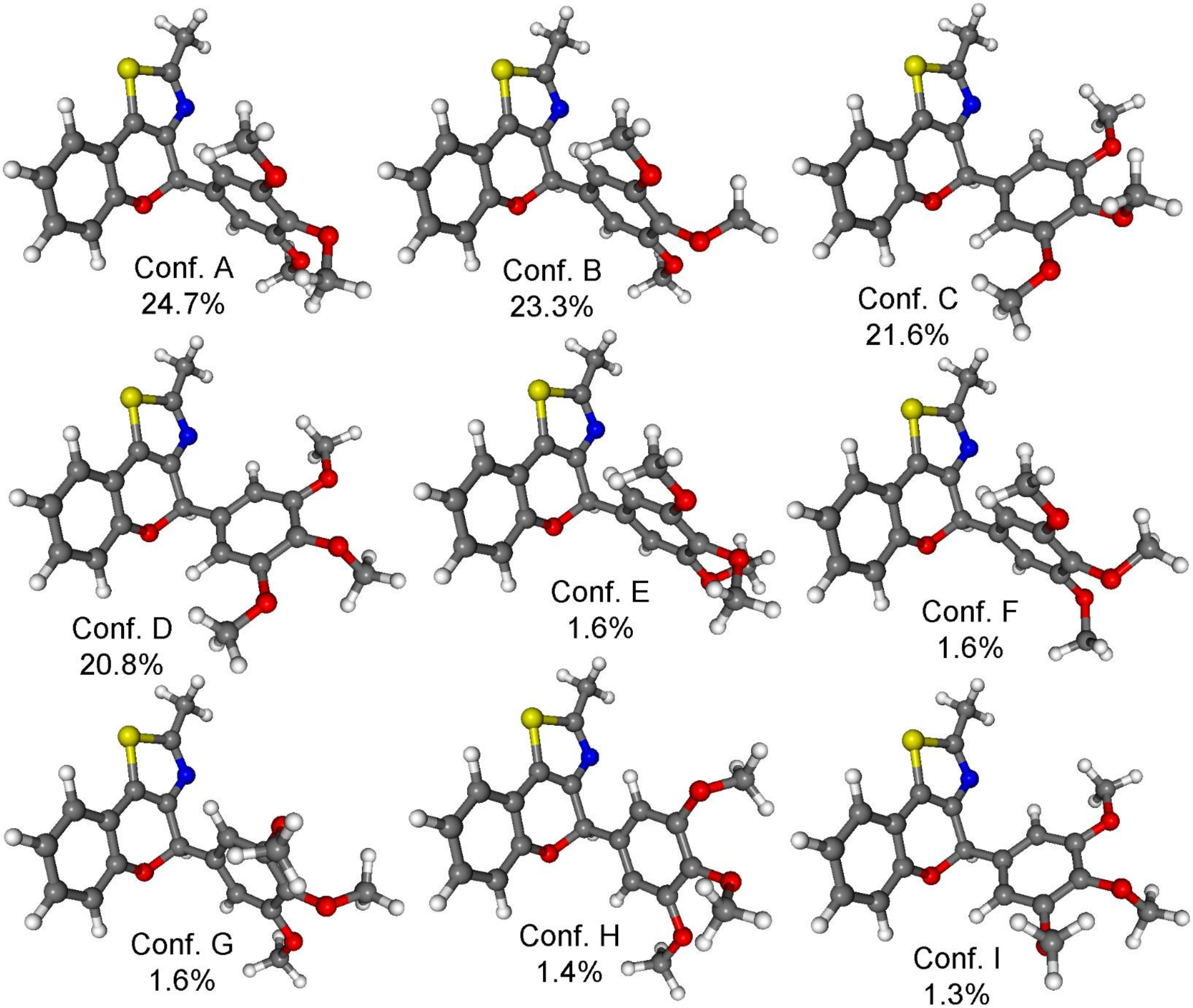

Figure S339. Structures and populations of the low-energy ωB97X/TZVP PCM/$CHCl_3$ conformers of (*R*)-**3e**.

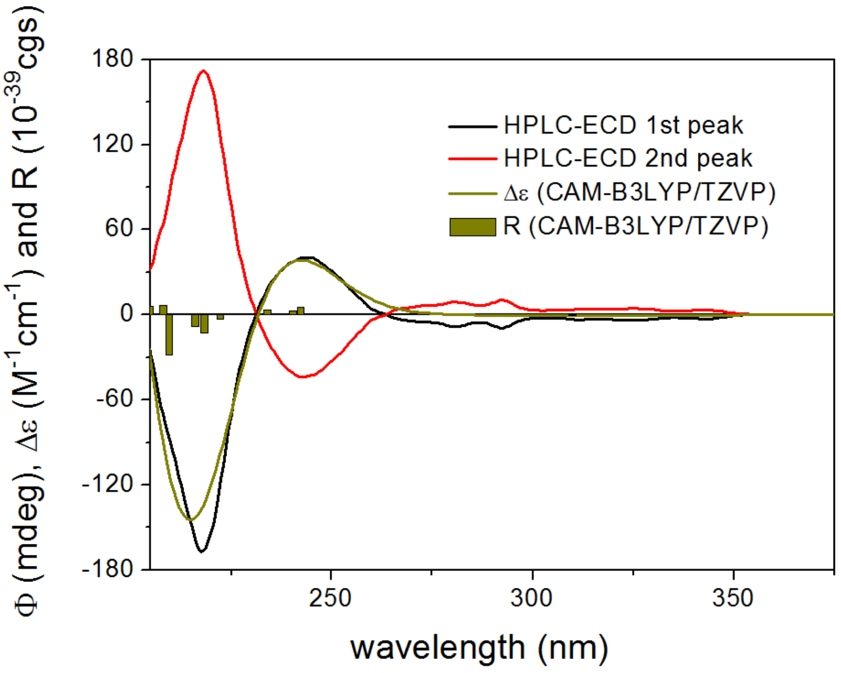

Figure S340. HPLC-ECD spectra of the first (black line) and the second-eluting (red line) enantiomers of **3a** compared with the CAM-B3LYP/TZVP PCM/$CHCl_3$ // CAM-B3LYP/TZVP PCM/$CHCl_3$ spectrum of (*R*)-**3a** (dark yellow line). The bars represent rotational strength values for the single low-energy solution conformer.

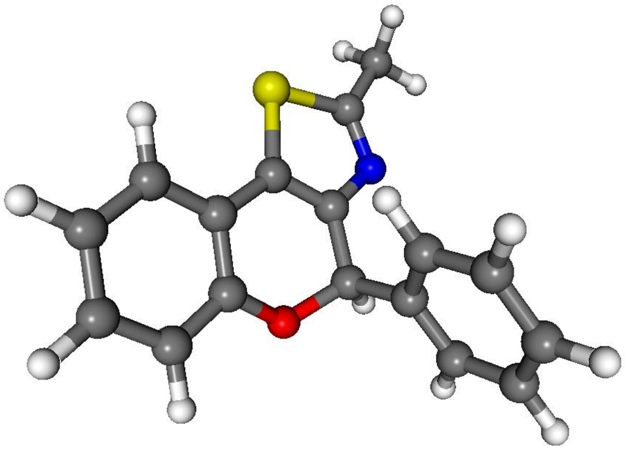

Figure S341. Single low-energy CAM-B3LYP/TZVP PCM/$CHCl_3$ conformer of (*R*)-**3a**.

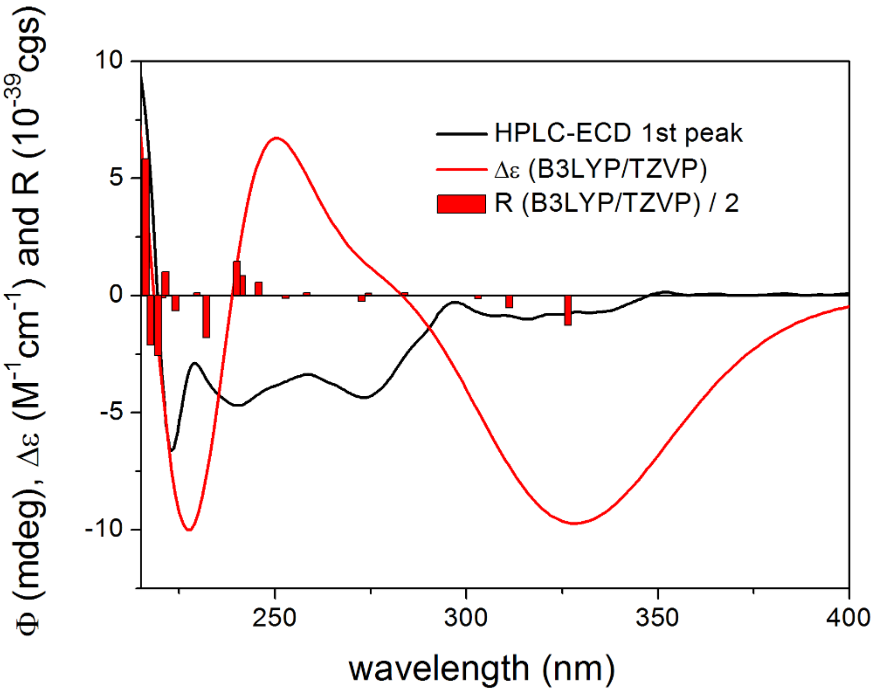

Figure S342. HPLC-ECD spectra of the first-eluting enantiomer (black line, enantiomer corrected) of **3f** compared with the B3LYP/TZVP PCM/$CHCl_3$ // ωB97X/TZVP PCM/$CHCl_3$ spectrum of (*R*)-**3f** (red line). The bars represent rotational strength values for the lowest-energy solution conformer.

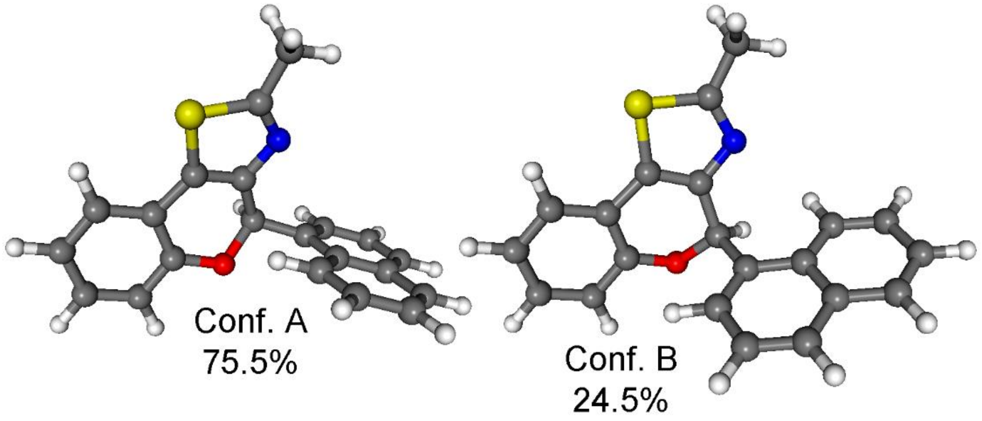

Figure S343. Structures and populations of the low-energy ωB97X/TZVP PCM/$CHCl_3$ conformers of (*R*)-**3f**.

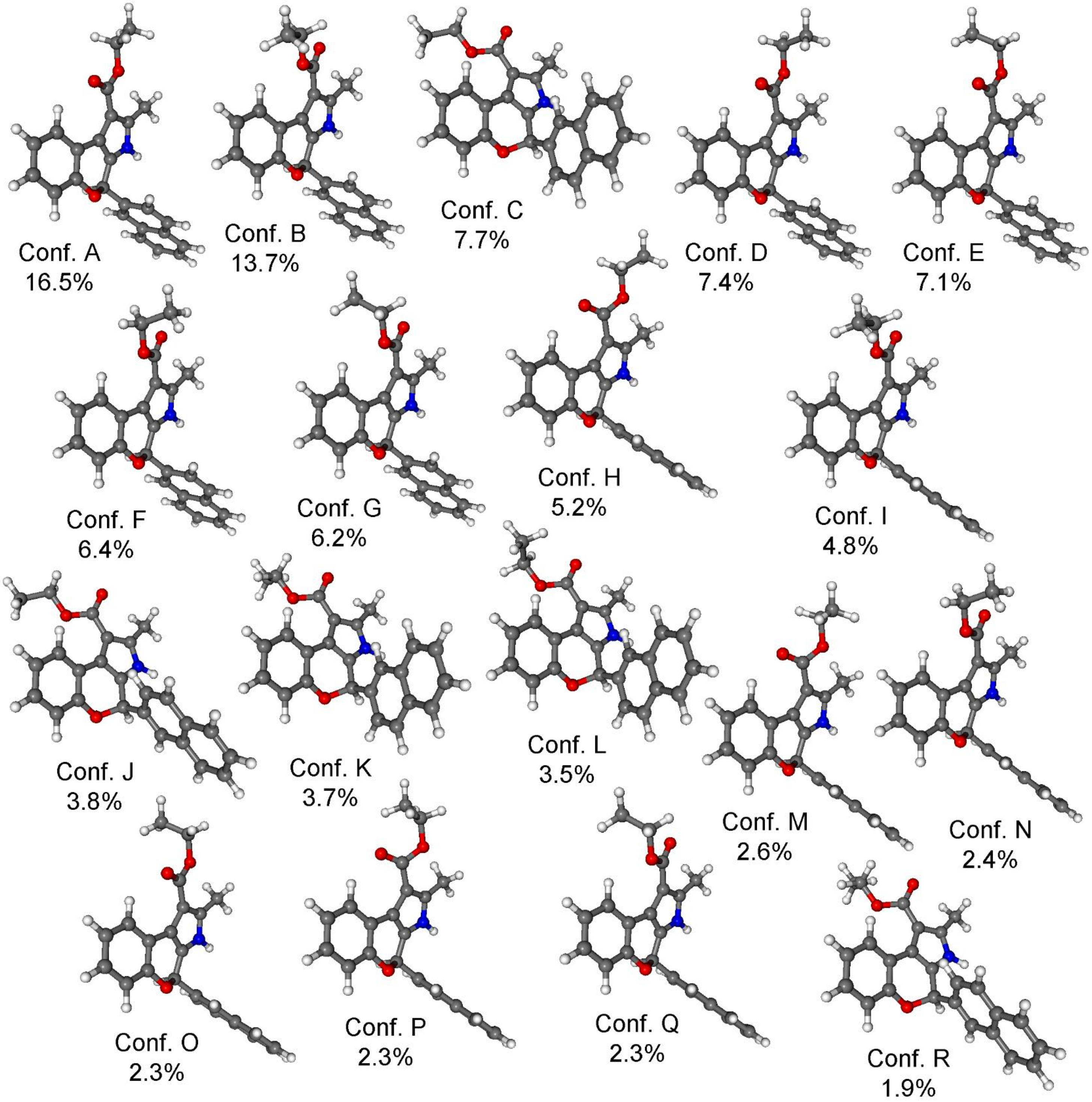

Figure S344. Structures and populations of the low-energy ωB97X/TZVP PCM/$CHCl_3$ conformers of (*R*)-**4g**.

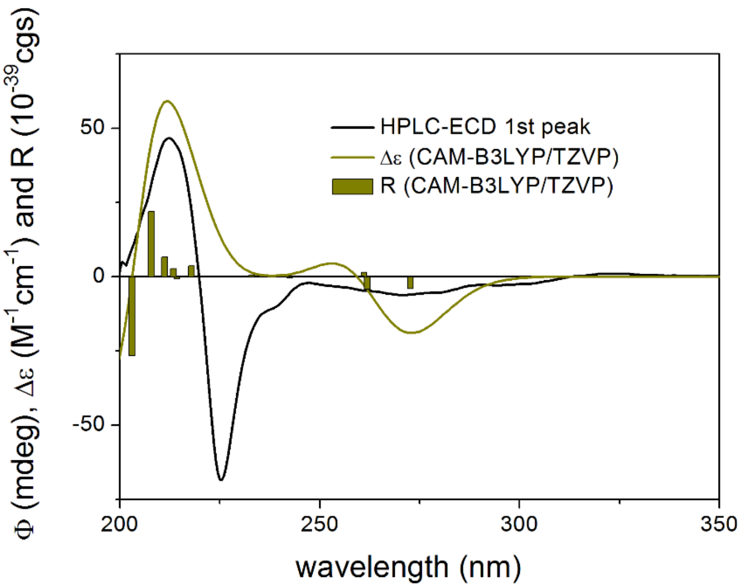

Figure S345. HPLC-ECD spectra of the first-eluting enantiomer (black line) of **4f** compared with the CAM-B3LYP/TZVP PCM/$CHCl_3$ // CAM-B3LYP/TZVP PCM/$CHCl_3$ spectrum of (*R*)-**4f** (dark yellow line). The bars represent rotational strength values for the lowest-energy solution conformer.

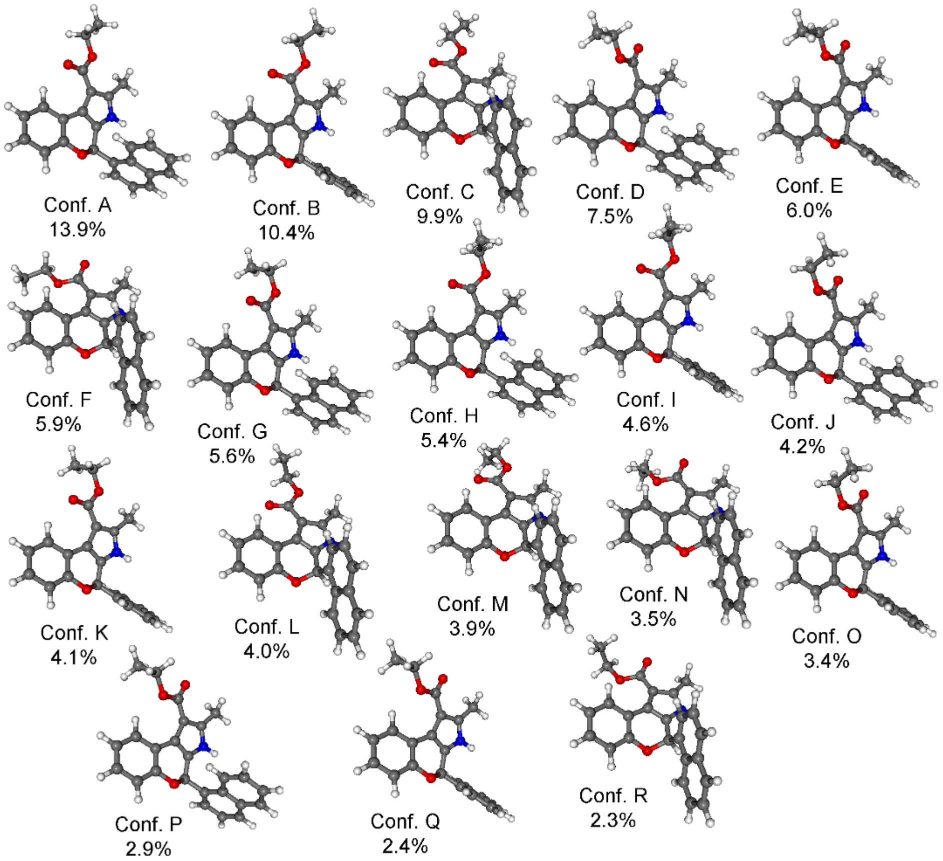

Figure S346. Structures and populations of the low-energy CAM-B3LYP/TZVP PCM/$CHCl_3$ conformers of (*R*)-**4f**.

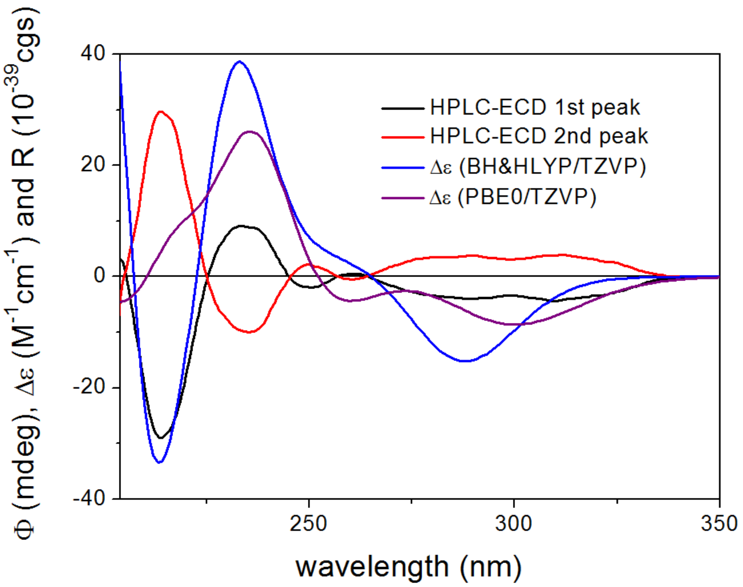

Figure S347. HPLC-ECD spectra of the first (black line) and the second-eluting (red line) enantiomers of **4a** compared with the BH&HLYP/TZVP PCM/$CHCl_3$ (blue line) and the PBE0/TZVP PCM/$CHCl_3$ (purple line) spectra of (*R*)-**4a** (level of DFT optimization: ωB97X/TZVP PCM/$CHCl_3$).

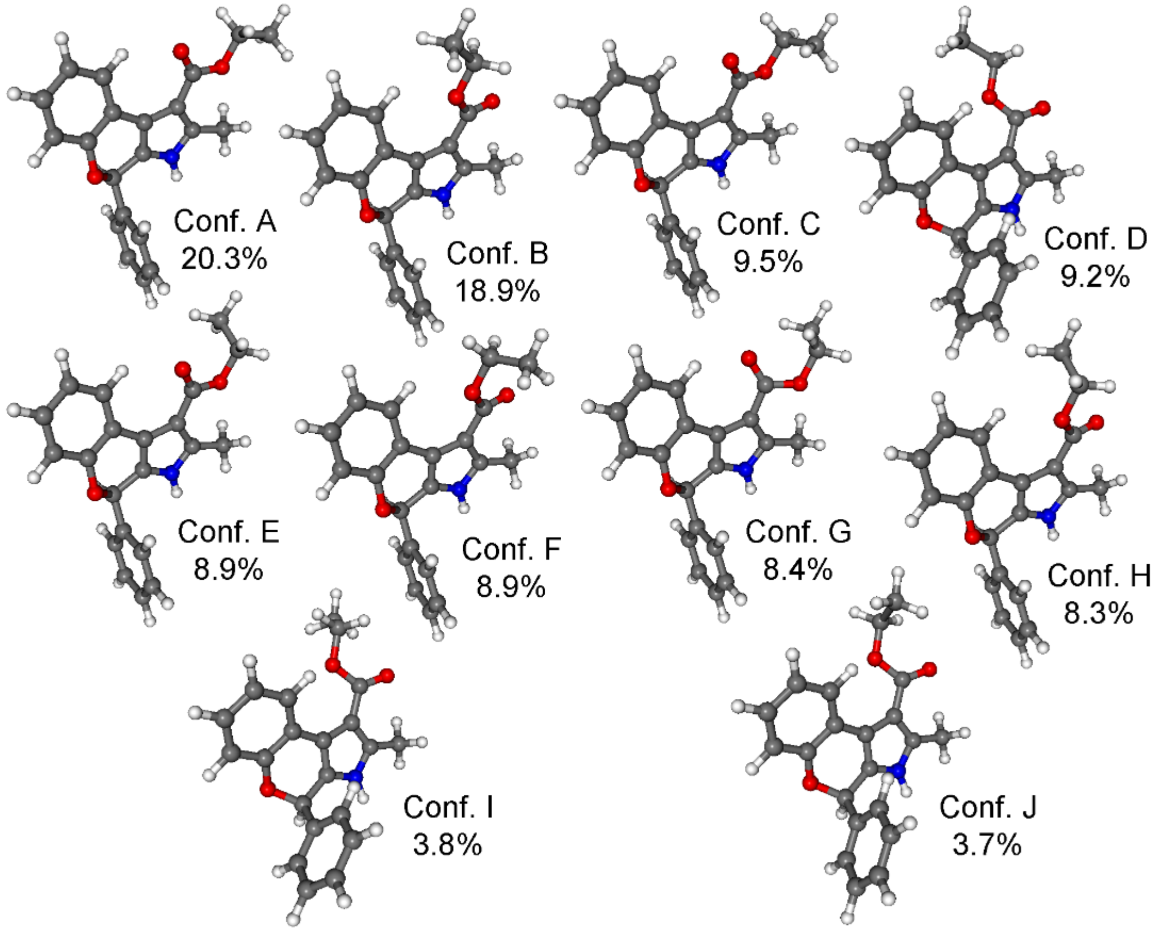

Figure S348. Structures and populations of the low-energy ωB97X/TZVP PCM/$CHCl_3$ conformers of (*R*)-**29a**.

4. X-ray analysis of **17e**

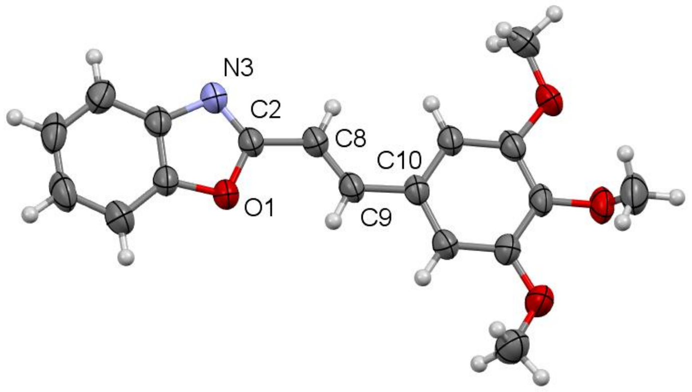

Figure S349. ORTEP view of **17e** drawn at 50% probability level with partial numbering scheme. Selected bond lengths (Å) and angles (°): N3–C2 1.290(3), O1-C2 1.368(3), C8-C9 1.323(4), C2-C8 1.442(4), N3-C2-O1 115.6(2), O1-C2-C8 116.9(2), C2-C8-C9 7124.2(3).

Table S2. Experimental details of single crystal X-ray diffraction measurement of **17e**

**Crystal data**	
Chemical formula	$C_{18}H_{17}NO_4$
M_r	311.32
Crystal system, space group	Monoclinic, $P2_1/n$ (No. 14)
Temperature (K)	298
a, *b*, *c* (Å)	11.206 (9), 12.132 (9), 11.723 (6)
β (°)	105.97 (5)
V (Å^3)	1532.1 (19)
Z	4
Radiation type	Mo *K*α
μ (mm^{-1})	0.10
Crystal size (mm)	0.35 × 0.3 × 0.25
**Data collection**	
Diffractometer	Enraf Nonius MACH3
Absorption correction	–
No. of measured, independent and observed [$I > 2\sigma(I)$] reflections	3093, 2892, 1912
R_{int}	0.020
$(\sin \theta/\lambda)_{max}$ (Å^{-1})	0.609
**Refinement**	
$R[F^2 > 2\sigma(F^2)]$, $wR(F^2)$, S	0.061, 0.190, 1.12
No. of reflections	2892
No. of parameters	211

H-atom treatment	H-atom parameters constrained
$\Delta\rangle_{max}$, $\Delta\rangle_{min}$ (e Å^{-3})	0.25, -0.25
