# Supplementary material for: Synthesis and HPLC-ECD Study of Cytostatic Condensed O,N-Heterocycles Obtained from 3-Aminoflavanones
Source: Biomolecules. 2020 Oct 20;10(10):1462. doi: 10.3390/biom10101462 (PMC7593906; doi:10.3390/biom10101462)
Supplement: Supplementary file 1 [file biomolecules-10-01462-s001.zip › biomolecules-924992-final-supplementary materials/Biomolecules_SI_part_I_20201013_rev.pdf]

Supporting Information

(PART I.)

Synthesis and HPLC-ECD Study of Cytostatic Condensed O,N-Heterocycles Obtained from 3-Aminoflavanones

Ádám Szappanos [1], Attila Mándi [1], Katalin Gulácsi [1,†], Erika Lisztes [2], Balázs István Tóth [2], Tamás Bíró [3], Anita Kónya-Ábrahám [1], Attila Kiss-Szikszai [1], Attila Bényei [4], Sándor Antus [1], Tibor Kurtán [1,*]

[1] Department of Organic Chemistry, University of Debrecen, P. O. Box 400, 4002 Debrecen, Hungary Fax: +36-52-512-744 E-mail: kurtan.tibor@science.unideb.hu.

[2] Department of Physiology, University of Debrecen, Debrecen, Hungary

[3] Department of Immunology, University of Debrecen, Debrecen, Hungary

[4] Department og Physical Chemistry, University of Debrecen, Debrecen, Hungary

[†] deceased

* Correspondence: E-mail: kurtan.tibor@science.unideb.hu.; Fax: +36-52-512-744

Table of Contents

1 Experimental Section of known compounds 9

1.1. General Procedure for the Synthesis of chalcone analogues (**15a-g**) 9

1.2. General Procedure for the Synthesis of 4-Chromanone Derivatives (**6a-g**) 10

1.3. General Procedure for the Synthesis of oxime analogues (**16a-g**) 12

1.4. General Procedure for Neber rearrangement (*rac-cis*-**1a-g,** *rac-trans*-**1a-g, 17a-g**) 14

1.5. References of known compounds 15

2 1D- and 2D-NMR spectra 17

Figure S1. ^{1}H-NMR spectrum of **16a** in $CDCl_3$ 17

Figure S2. ^{13}C-NMR spectrum of **16a** in $CDCl_3$ 18

Figure S3. ^{1}H-NMR spectrum of **16b** in $CDCl_3$ 19

Figure S4. ^{13}C-NMR spectrum of **16b** in $CDCl_3$ 20

Figure S5. ^{1}H-NMR spectrum of **16c** in $CDCl_3$ 21

Figure S6. ^{13}C-NMR spectrum of **16c** in $CDCl_3$ 22

Figure 7. ^{1}H-NMR spectrum of **16d** in $CDCl_3$ 23

Figure S8. ^{13}C-NMR spectrum of **16d** in $CDCl_3$ 24

Figure S9. ^{1}H-NMR spectrum of **16e** in $CDCl_3$ 25

Figure S10. ^{13}C-NMR spectrum of **16e** in $CDCl_3$ 26

Figure S11. ^{1}H-NMR spectrum of **16f** in DMSO-d_6 27
Figure S12. ^{13}C-NMR spectrum of **16f** in DMSO-d_6 28
Figure S13. ^{1}H-NMR spectrum of **16g** in DMSO-d_6 29
Figure S14. ^{13}C-NMR spectrum of **16g** in DMSO-d_6 30
Figure S15. ^{1}H-NMR spectrum of **5a** in $CDCl_3$ 31
Figure S16. ^{13}C-NMR spectrum of **5a** in $CDCl_3$ 32
Figure S17. ^{1}H-NMR spectrum of **5b** in $CDCl_3$ 33
Figure 18. ^{13}C-NMR spectrum of **5b** in $CDCl_3$ 34
Figure S19. ^{1}H-NMR spectrum of **5c** in $CDCl_3$ 35
Figure 20. ^{13}C-NMR spectrum of **5c** in $CDCl_3$ 36
Figure 21. ^{1}H-NMR spectrum of **5d** in $CDCl_3$ 37
Figure S22. ^{13}C-NMR spectrum of **5d** in $CDCl_3$ 38
Figure S23. ^{1}H-NMR spectrum of **5e** in $CDCl_3$ 39
Figure S24. ^{13}C-NMR spectrum of **5e** in $CDCl_3$ 40
Figure S25. ^{1}H-NMR spectrum of **5f** in $CDCl_3$ 41
Figure S26. ^{13}C-NMR spectrum of **5f** in $CDCl_3$ 42
Figure S27. ^{1}H-NMR spectrum of **5g** in $CDCl_3$ 43
Figure S28. ^{13}C-NMR spectrum of **5g** in $CDCl_3$ 44
Figure S29. ^{1}H-NMR spectrum of *rac-trans*-**1a** in DMSO-d_6 45
Figure S30. ^{13}C-NMR spectrum of *rac-trans*-**1a** in DMSO-d_6 46
Figure S31. ^{1}H-NMR spectrum of *rac-trans*-**1b** in DMSO-d_6 47
Figure S32. ^{13}C-NMR spectrum of *rac-trans*-**1b** in DMSO-d_6 48
Figure S33. ^{1}H-NMR spectrum of *rac-trans*-**1c** in DMSO-d_6 49
Figure S34. ^{13}C-NMR spectrum of *rac-trans*-**1c** in DMSO-d_6 50
Figure S35. ^{1}H-NMR spectrum of *rac-trans*-**1d** in DMSO-d_6 51
Figure S36. ^{13}C-NMR spectrum of *rac-trans*-**1d** in DMSO-d_6 52
Figure S37. ^{1}H-NMR spectrum of *rac-trans*-**1e** in DMSO-d_6 53
Figure S38. ^{13}C-NMR spectrum of *rac-trans*-**1e** in DMSO-d_6 54
Figure S39. ^{1}H-NMR spectrum of *rac-trans*-**1f** in DMSO-d_6 55
Figure S40. ^{13}C-NMR spectrum of *rac-trans*-**1f** in DMSO-d_6 56
Figure S41. COSY-spectrum of *rac-trans*-**1f** in DMSO-d_6 57
Figure S42. HSQC-spectrum of *rac-trans*-**1f** in DMSO-d_6 58
Figure S43. ^{1}H-NMR spectrum of *rac-trans*-**1g** in DMSO-d_6 59
Figure S44. ^{13}C-NMR spectrum of *rac-trans*-**1g** in DMSO-d_6 60
Figure S45. ^{1}H-NMR spectrum of *rac-cis*-**1a** in DMSO-d_6 61
Figure S46. ^{13}C-NMR spectrum of *rac-cis*-**1a** in DMSO-d_6 62
Figure S47. ^{1}H-NMR spectrum of *rac-cis*-**1b** in DMSO-d_6 63

Figure S48. ^{13}C-NMR spectrum of *rac-cis*-**1b** in DMSO-d_6 64
Figure S49. ^{1}H-NMR spectrum of *rac-cis*-**1c** in DMSO-d_6 65
Figure S50. ^{13}C-NMR spectrum of *rac-cis*-**1c** in DMSO-d_6 66
Figure S51. ^{1}H-NMR spectrum of *rac-cis*-**1d** in DMSO-d_6 67
Figure S52. ^{13}C-NMR spectrum of *rac-cis*-**1d** in DMSO-d_6 68
Figure S53. ^{1}H-NMR spectrum of *rac-cis*-**1e** in DMSO-d_6 69
Figure S54. ^{13}C-NMR spectrum of *rac-cis*-**1e** in DMSO-d_6 70
Figure S55. ^{1}H-NMR spectrum of **17a** in $CDCl_3$ 71
Figure S56. ^{13}C-NMR spectrum of **17a** in $CDCl_3$ 72
Figure S57. ^{1}H-NMR spectrum of **17b** in $CDCl_3$ 73
Figure S58. ^{13}C-NMR spectrum of **17b** in $CDCl_3$ 74
Figure S59. ^{1}H-NMR spectrum of **17c** in $CDCl_3$ 75
Figure S60. ^{13}C-NMR spectrum of **17c** in $CDCl_3$ 76
Figure S61. COSY-spectrum of **17c** in $CDCl_3$ 77
Figure S62. HSQC-spectrum of **17c** in $CDCl_3$ 78
Figure S63. ^{1}H-NMR spectrum of **17e** in $CDCl_3$ 79
Figure S64. ^{13}C-NMR spectrum of **17e** in $CDCl_3$ 80
Figure S65. COSY-spectrum of **17e** in $CDCl_3$ 81
Figure S66. HSQC-spectrum of **17e** in $CDCl_3$ 82
Figure S67. HMBC-spectrum of **17e** in $CDCl_3$ 83
Figure S68. ^{1}H-NMR spectrum of **17f** in $CDCl_3$ 84
Figure S69. ^{13}C-NMR spectrum of **17f** in $CDCl_3$ 85
Figure S70. ^{1}H-NMR spectrum of **17g** in $CDCl_3$ 86
Figure S71. ^{13}C-NMR spectrum of **17g** in $CDCl_3$ 87
Figure S72. ^{1}H-NMR spectrum of *rac-trans*-**18a** in $CDCl_3$ 88
Figure S73. ^{13}C-NMR spectrum of *rac-trans*-**18a** in $CDCl_3$ 89
Figure S74. ^{1}H-NMR spectrum of *rac-trans*-**18b** in DMSO-d_6 90
Figure S75. ^{13}C-NMR spectrum of *rac-trans*-**18b** in DMSO-d_6 91
Figure S76. ^{1}H-NMR spectrum of *rac-trans*-**18c** in DMSO-d_6 92
Figure S77. ^{13}C-NMR spectrum of *rac-trans*-**18c** in DMSO-d_6 93
Figure S78. ^{1}H-NMR spectrum of *rac-trans*-**18d** in DMSO-d_6 94
Figure S79. ^{13}C-NMR spectrum of *rac-trans*-**18d** in DMSO-d_6 95
Figure S80. ^{1}H-NMR spectrum of *rac-trans*-**18e** in $CDCl_3$ 96
Figure S81. ^{13}C-NMR spectrum of *rac-trans*-**18e** in $CDCl_3$ 97
Figure S82. ^{1}H-NMR spectrum of *rac-trans*-**18f** in DMSO-d_6 98
Figure S83. ^{13}C-NMR spectrum of *rac-trans*-**18f** in $CDCl_3$ 99
Figure S84. HSQC-spectrum of *rac-trans*-**18f** in DMSO-d_6 100

Figure S85. ^{1}H-NMR spectrum of *rac-trans*-**18g** in DMSO-d_6 101
Figure S86. ^{13}C-NMR spectrum of *rac-trans*-**18g** in $CDCl_3$ 102
Figure S87. ^{1}H-NMR spectrum of *rac*-**19a** in Acetone-d_6 103
Figure S88. ^{13}C-NMR spectrum of *rac*-**19a** in Acetone-d_6 104
Figure S89. ^{1}H-NMR spectrum of *rac*-**19b** in $CDCl_3$ 105
Figure S90. ^{13}C-NMR spectrum of *rac*-**19b** in $CDCl_3$ 106
Figure S91. ^{1}H-NMR spectrum of *rac*-**19c** in $CDCl_3$ 107
Figure S92. ^{13}C-NMR spectrum of *rac*-**19c** in $CDCl_3$ 108
Figure S93. ^{1}H-NMR spectrum of *rac*-**19d** in Acetone-d_6 109
Figure S94. ^{13}C-NMR spectrum of *rac*-**19d** in Acetone-d_6 110
Figure S95. COSY-spectrum of *rac*-**19d** in Acetone-d_6 111
Figure S96. HSQC-spectrum of *rac*-**19d** in Acetone-d_6 112
Figure S97. ^{1}H-NMR spectrum of *rac*-**19e** in $CDCl_3$ 113
Figure S98. ^{13}C-NMR spectrum of *rac*-**19e** in $CDCl_3$ 114
Figure S99. COSY-spectrum of *rac*-**19e** in $CDCl_3$ 115
Figure S100. COSY-spectrum of *rac*-**19e** in $CDCl_3$ 116
Figure S101. ^{1}H-NMR spectrum of *rac*-**19f** in DMSO-d_6 117
Figure S102. ^{13}C-NMR spectrum of *rac*-**19f** in DMSO-d_6 118
Figure S103. HSQC spectrum of *rac*-**19f** in DMSO-d_6 119
Figure S104. ^{1}H-NMR spectrum of *rac*-**19g** in Acetone-d_6 120
Figure S105. ^{13}C-NMR spectrum of *rac*-**19g** in Acetone-d_6 121
Figure S106. ^{1}H-NMR spectrum of *rac*-**22a** in $CDCl_3$ 122
Figure S107. ^{13}C-NMR spectrum of *rac*-**22a** in $CDCl_3$ 123
Figure S108. COSY spectrum of *rac*-**22a** in $CDCl_3$ 124
Figure S109. HSQC spectrum of *rac*-**22a** in $CDCl_3$ 125
Figure S110. NOESY spectrum of *rac*-**22a** in $CDCl_3$ 126
Figure S111. ^{1}H-NMR spectrum of *rac*-**22b** in $CDCl_3$ 127
Figure S112. ^{13}C-NMR spectrum of *rac*-**22b** in $CDCl_3$ 128
Figure S113. ^{1}H-NMR spectrum of *rac*-**22c** in $CDCl_3$ 129
Figure S114. ^{13}C-NMR spectrum of *rac*-**22c** in $CDCl_3$ 130
Figure S115. ^{1}H-NMR spectrum of *rac*-**22d** in $CDCl_3$ 131
Figure S116. ^{13}C-NMR spectrum of *rac*-**22d** in $CDCl_3$ 132
Figure S117. ^{1}H-NMR spectrum of *rac*-**22e** in $CDCl_3$ 133
Figure S118. ^{13}C-NMR spectrum of *rac*-**22e** in $CDCl_3$ 134
Figure S119. ^{1}H-NMR spectrum of *rac*-**22g** in Acetone-d_6 135
Figure S120. ^{13}C-NMR spectrum of *rac*-**22g** in Acetone-d_6 136
Figure S121. ^{1}H-NMR spectrum of *rac*-**20a** in $CDCl_3$ 137

Figure S122. ^{13}C-NMR spectrum of *rac*-**20a** in $CDCl_3$ 138
Figure S123. COSY spectrum of *rac*-**20a** in $CDCl_3$ 139
Figure S124. HSQC spectrum of *rac*-**20a** in $CDCl_3$ 140
Figure S125. ^{1}H-NMR spectrum of *rac*-**20b** in DMSO-d_6 141
Figure S126. ^{13}C-NMR spectrum of *rac*-**20b** in DMSO-d_6 142
Figure S127. ^{1}H-NMR spectrum of *rac*-**20c** in DMSO-d_6 143
Figure S128. ^{13}C-NMR spectrum of *rac*-**20c** in DMSO-d_6 144
Figure S129. ^{1}H-NMR spectrum of *rac*-**20d** in $CDCl_3$ 145
Figure S130. ^{13}C-NMR spectrum of *rac*-**20d** in $CDCl_3$ 146
Figure S131. COSY-spectrum of *rac*-**20d** in $CDCl_3$ 147
Figure S132. COSY-spectrum of *rac*-**20d** in $CDCl_3$ 148
Figure S133. ^{1}H-NMR spectrum of *rac*-**20e** in $CDCl_3$ 149
Figure S134. ^{13}C-NMR spectrum of *rac*-**20e** in $CDCl_3$ 150
Figure S135. ^{1}H-NMR spectrum of *rac*-**20f** in DMSO-d_6 151
Figure S136. ^{13}C-NMR spectrum of *rac*-**20f** in DMSO-d_6 152
Figure S137. HSQC spectrum of *rac*-**20f** in DMSO-d_6 153
Figure S138. ^{1}H-NMR spectrum of *rac*-**20g** in DMSO-d_6 154
Figure S139. ^{13}C-NMR spectrum of *rac*-**20g** in DMSO-d_6 155
Figure S140. ^{1}H-NMR spectrum of *rac*-**23a** in $CDCl_3$ 156
Figure S141. ^{13}C-NMR spectrum of *rac*-**23a** in $CDCl_3$ 157
Figure S142. ^{1}H-NMR spectrum of *rac*-**23b** in $CDCl_3$ 158
Figure S143. ^{13}C-NMR spectrum of *rac*-**23b** in $CDCl_3$ 159
Figure S144. COSY spectrum of *rac*-**23b** in $CDCl_3$ 160
Figure S145. HSQC spectrum of *rac*-**23b** in $CDCl_3$ 161
Figure S146. ^{1}H-NMR spectrum of *rac*-**23c** in $CDCl_3$ 162
Figure S147. ^{13}C-NMR spectrum of *rac*-**23c** in $CDCl_3$ 163
Figure S148. ^{1}H-NMR spectrum of *rac*-**23d** in $CDCl_3$ 164
Figure S149. ^{13}C-NMR spectrum of *rac*-**23d** in $CDCl_3$ 165
Figure S150. ^{1}H-NMR spectrum of *rac*-**23e** in $CDCl_3$ 166
Figure S151. ^{13}C-NMR spectrum of *rac*-**23e** in $CDCl_3$ 167
Figure S152. ^{1}H-NMR spectrum of *rac*-**23g** in $CDCl_3$ 168
Figure S153. ^{13}C-NMR spectrum of *rac*-**23g** in $CDCl_3$ 169
Figure S154. ^{1}H-NMR spectrum of *rac*-(4a*R**,5*S**,10a*S**)-**2a** in DMSO-d_6 170
Figure S155. ^{13}C-NMR spectrum of *rac*-(4a*R**,5*S**,10a*S**)-**2a** in DMSO-d_6 171
Figure S156. ^{1}H-NMR spectrum of *rac*-(4a*R**,5*S**,10a*R**)-**2a** in $CDCl_3$ 172
Figure S157. ^{13}C-NMR spectrum of *rac*-(4a*R**,5*S**,10a*R**)-**2a** in $CDCl_3$ 173
Figure S158. COSY spectrum of *rac*-(4a*R**,5*S**,10a*R**)-**2a** in $CDCl_3$ 174

Figure S159. HSQC spectrum of *rac*-(4a*R**,5*S**,10a*R**)-**2a** in $CDCl_3$ 175
Figure S160. NOESY spectrum of *rac*-(4a*R**,5*S**,10a*R**)-**2a** in $CDCl_3$ 176
Figure S161. ^{1}H-NMR spectrum of *rac*-(4a*R**,5*R**,10a*R**)-**2a** in Acetone-d_6 177
Figure S162. ^{13}C-NMR spectrum of *rac*-(4a*R**,5*R**,10a*R**)-**2a** in Acetone-d_6 178
Figure S163. COSY spectrum of *rac*-(4a*R**,5*R**,10a*R**)-**2a** in Acetone-d_6 179
Figure S164. HSQC spectrum of *rac*-(4a*R**,5*R**,10a*R**)-**2a** in Acetone-d_6 180
Figure 165. NOESY spectrum of *rac*-(4a*R**,5*R**,10a*R**)-**2a** in Acetone-d_6 181
Figure S166. ^{1}H-NMR spectrum of *rac*-(4a*R**,5*S**,10a*S**)-**2b** in DMSO-d_6 182
Figure S167. ^{13}C-NMR spectrum of *rac*-(4a*R**,5*S**,10a*S**)-**2b** in DMSO-d_6 183
Figure S168. ^{1}H-NMR spectrum of *rac*-(4a*R**,5*S**,10a*R**)-**2b** in $CDCl_3$ 184
Figure S169. ^{13}C-NMR spectrum of *rac*-(4a*R**,5*S**,10a*R**)-**2b** in $CDCl_3$ 185
Figure S170. ^{1}H-NMR spectrum of *rac*-(4a*R**,5*R**,10a*R**)-**2b** in Acetone-d_6 186
Figure S171. ^{13}C-NMR spectrum of *rac*-(4a*R**,5*R**,10a*R**)-**2b** in Acetone-d_6 187
Figure S172. ^{1}H-NMR spectrum of *rac*-(4a*R**,5*S**,10a*S**)-**2c** in $CDCl_3$ 188
Figure S173. ^{13}C-NMR spectrum of *rac*-(4a*R**,5*S**,10a*S**)-**2c** in $CDCl_3$ 189
Figure S174. ^{1}H-NMR spectrum of *rac*-(4a*R**,5*S**,10a*R**)-**2c** in $CDCl_3$ 190
Figure S175. ^{13}C-NMR spectrum of *rac*-(4a*R**,5*S**,10a*R**)-**2c** in $CDCl_3$ 191
Figure S176. ^{1}H-NMR spectrum of *rac*-(4a*R**,5*R**,10a*R**)-**2c** in Acetone-d_6 192
Figure S177. ^{13}C-NMR spectrum of *rac*-(4a*R**,5*R**,10a*R**)-**2c** in Acetone-d_6 193
Figure S178. ^{1}H-NMR spectrum of *rac*-(4a*R**,5*S**,10a*S**)-**2d** in DMSO-d_6 194
Figure S179. ^{13}C-NMR spectrum of *rac*-(4a*R**,5*S**,10a*S**)-**2d** in DMSO-d_6 195
Figure S180. COSY spectrum of *rac*-(4a*R**,5*S**,10a*S**)-**2d** in DMSO-d_6 196
Figure S181. ^{1}H-NMR spectrum of *rac*-(4a*R**,5*S**,10a*R**)-**2d** in $CDCl_3$ 197
Figure S182. ^{13}C-NMR spectrum of *rac*-(4a*R**,5*S**,10a*R**)-**2d** in $CDCl_3$ 198
Figure S183. ^{1}H-NMR spectrum of *rac*-(4a*R**,5*R**,10a*R**)-**2d** in Acetone-d_6 199
Figure S184. ^{13}C-NMR spectrum of *rac*-(4a*R**,5*R**,10a*R**)-**2d** in Acetone-d_6 200
Figure S185. ^{1}H-NMR spectrum of *rac*-(4a*R**,5*S**,10a*S**)-**2e** in $CDCl_3$ 201
Figure S186. ^{13}C-NMR spectrum of *rac*-(4a*R**,5*S**,10a*S**)-**2e** in $CDCl_3$ 202
Figure S187. ^{1}H-NMR spectrum of *rac*-(4a*R**,5*S**,10a*R**)-**2e** in $CDCl_3$ 203
Figure S188. ^{13}C-NMR spectrum of *rac*-(4a*R**,5*S**,10a*R**)-**2e** in $CDCl_3$ 204
Figure S189. ^{1}H-NMR spectrum of *rac*-(4a*R**,5*R**,10a*R**)-**2e** in Acetone-d_6 205
Figure S190. ^{13}C-NMR spectrum of *rac*-(4a*R**,5*R**,10a*R**)-**2e** in Acetone-d_6 206
Figure S191. ^{1}H-NMR spectrum of *rac*-(4a*R**,5*S**,10a*S**)-**2f** in DMSO-d_6 207
Figure S192. ^{13}C-NMR spectrum of *rac*-(4a*R**,5*S**,10a*S**)-**2f** in DMSO-d_6 208
Figure S193. COSY spectrum of *rac*-(4a*R**,5*S**,10a*S**)-**2f** in DMSO-d_6 209
Figure S194. COSY spectrum of *rac*-(4a*R**,5*S**,10a*S**)-**2f** in DMSO-d_6 (maximized) 210
Figure S195. HSQC spectrum of *rac*-(4a*R**,5*S**,10a*S**)-**2f** in DMSO-d_6 211

Figure S196. HSQC spectrum of rac-(4aR*,5S*,10aS*)-**2f** in DMSO-d_6 (maximized) 212
Figure S197. ^{1}H-NMR spectrum of *rac*-(4a*R**,5*S**,10a*S**)-**2g** in $CDCl_3$ 213
Figure S198. ^{13}C-NMR spectrum of *rac*-(4a*R**,5*S**,10a*S**)-**2g** in $CDCl_3$ 214
Figure S199. ^{1}H-NMR spectrum of *rac*-(4a*R**,5*S**,10a*R**)-**2g** in $CDCl_3$ 215
Figure S200. ^{13}C-NMR spectrum of *rac*-(4a*R**,5*S**,10a*R**)-**2g** in $CDCl_3$ 216
Figure S201. ^{1}H-NMR spectrum of *rac*-(4a*R**,5*R**,10a*R**)-**2g** in Acetone-d_6 217
Figure S202. ^{13}C-NMR spectrum of *rac*-(4a*R**,5*R**,10a*R**)-**2g** in Acetone-d_6 218
Figure S203. ^{1}H-NMR spectrum of *rac-trans*-**24a** in $CDCl_3$ 219
Figure S204. ^{13}C-NMR spectrum of *rac-trans*-**24a** in $CDCl_3$ 220
Figure S205. ^{1}H-NMR spectrum of *rac-trans*-**24b** in $CDCl_3$ 221
Figure S206. ^{13}C-NMR spectrum of *rac-trans*-**24b** in $CDCl_3$ 222
Figure S207. ^{1}H-NMR spectrum of *rac-trans*-**24c** in DMSO-d_6 223
Figure S208. ^{13}C-NMR spectrum of *rac-trans*-**24c** in DMSO-d_6 224
Figure S209. ^{1}H-NMR spectrum of *rac-trans*-**24d** in DMSO-d_6 225
Figure S210. ^{13}C-NMR spectrum of *rac-trans*-**24d** in DMSO-d_6 226
Figure S211. ^{1}H-NMR spectrum of *rac-trans*-**24e** in CD_3OD 227
Figure S212. ^{13}C-NMR spectrum of *rac-trans*-**24e** in CD_3OD 228
Figure S213. ^{1}H-NMR spectrum of *rac-trans*-**24f** in DMSO-d_6 229
Figure S214. ^{13}C-NMR spectrum of *rac-trans*-**24f** in DMSO-d_6 230
Figure S215. HSQC spectrum of *rac-trans*-**24f** in DMSO-d_6 231
Figure S216. ^{1}H-NMR spectrum of *rac-trans*-**24g** in DMSO-d_6 232
Figure S217. ^{13}C-NMR spectrum of *rac-trans*-**24g** in DMSO-d_6 233
Figure S218. ^{1}H-NMR spectrum of *rac-cis*-**24a** in $CDCl_3$ 234
Figure S219. ^{13}C-NMR spectrum of *rac-cis*-**24a** in $CDCl_3$ 235
Figure S220. ^{1}H-NMR spectrum of *rac-cis*-**24b** in $CDCl_3$ 236
Figure S221. ^{13}C-NMR spectrum of *rac-cis*-**24b** in $CDCl_3$ 237
Figure S222. ^{1}H-NMR spectrum of *rac-cis*-**24c** in $CDCl_3$ 238
Figure S223. ^{13}C-NMR spectrum of *rac-cis*-**24c** in $CDCl_3$ 239
Figure S224. ^{1}H-NMR spectrum of *rac-cis*-**24d** in $CDCl_3$ 240
Figure S225. ^{13}C-NMR spectrum of *rac-cis*-**24d** in $CDCl_3$ 241
Figure S226. ^{1}H-NMR spectrum of *rac-cis*-**24e** in $CDCl_3$ 242
Figure S227. ^{13}C-NMR spectrum of *rac-cis*-**24e** in $CDCl_3$ 243
Figure S228. ^{1}H-NMR spectrum of *rac*-**3a** in $CDCl_3$ 244
Figure S229. ^{13}C-NMR spectrum of *rac*-**3a** in $CDCl_3$ 245
Figure S230. COSY spectrum of *rac*-**3a** in $CDCl_3$ 246
Figure S231. HSQC spectrum of *rac*-**3a** in $CDCl_3$ 247
Figure S232. ^{1}H-NMR spectrum of *rac*-**3b** in $CDCl_3$ 248

Figure S233. ^{13}C-NMR spectrum of *rac*-**3b** in $CDCl_3$ 249
Figure S234. ^{1}H-NMR spectrum of *rac*-**3c** in $CDCl_3$ 250
Figure S235. ^{13}C-NMR spectrum of *rac*-**3c** in $CDCl_3$ 251
Figure S236. ^{1}H-NMR spectrum of *rac*-**3d** in $CDCl_3$ 252
Figure S237. ^{13}C-NMR spectrum of *rac*-**3d** in $CDCl_3$ 253
Figure S238. HMBC-spectrum of *rac*-**3d** in $CDCl_3$ 254
Figure S239. ^{1}H-NMR spectrum of *rac*-**3e** in $CDCl_3$ 255
Figure S240. ^{13}C-NMR spectrum of *rac*-**3e** in $CDCl_3$ 256
Figure 241. ^{1}H-NMR spectrum of *rac*-**3f** in $CDCl_3$ 257
Figure S242. ^{13}C-NMR spectrum of *rac*-**3f** in $CDCl_3$ 258
Figure S243. ^{1}H-NMR spectrum of *rac*-**3g** in $CDCl_3$ 259
Figure S244. ^{13}C-NMR spectrum of *rac*-**3g** in $CDCl_3$ 260
Figure S245. ^{1}H-NMR spectrum of *rac*-**4a** in $CDCl_3$ 261
Figure S246. ^{13}C-NMR spectrum of *rac*-**4a** in $CDCl_3$ 262
Figure S247. ^{1}H-NMR spectrum of *rac*-**4b** in $CDCl_3$ 263
Figure S248. ^{13}C-NMR spectrum of *rac*-**4b** in $CDCl_3$ 264
Figure S249. ^{1}H-NMR spectrum of *rac*-**4c** in $CDCl_3$ 265
Figure S250. ^{13}C-NMR spectrum of *rac*-**4c** in $CDCl_3$ 266
Figure S251. ^{1}H-NMR spectrum of *rac*-**4d** in $CDCl_3$ 267
Figure S252. ^{13}C-NMR spectrum of *rac*-**4d** in $CDCl_3$ 268
Figure S253. ^{1}H-NMR spectrum of *rac*-**4e** in $CDCl_3$ 269
Figure S254. ^{13}C-NMR spectrum of *rac*-**4e** in $CDCl_3$ 270
Figure S255. ^{1}H-NMR spectrum of *rac*-**4f** in DMSO-d_6 271
Figure S256. ^{13}C-NMR spectrum of *rac*-**4f** in DMSO-d_6 272
Figure S257. COSY-spectrum of *rac*-**4f** in DMSO-d_6 273
Figure S258. HSQC-spectrum of *rac*-**4f** in DMSO-d_6 274
Figure S259. HMBC-spectrum of *rac*-**4f** in DMSO-d_6 275
Figure S260. ^{1}H-NMR spectrum of *rac*-**4g** in $CDCl_3$ 276
Figure S261. ^{13}C-NMR spectrum of *rac*-**4g** in $CDCl_3$ 277
Table S1. Yields in the preparation of thiazole-condensed derivatives *rac*-**3a-g** from *rac-cis*- and *trans*-**1a-g** .... **Error! Bookmark not defined.**

1 Experimental Section of known compounds

1.1 General Procedure for the Synthesis of chalcone analogues (15a-g)

2-hidroxyacetophenone **13** (24.24 mmol) was dissolved in ethanol (165 mL) under stirring. To this was added NaOH (72.72 mmol in 5 mL water) and stirred for 5 min. To this reaction mixture, the aromatic aldehyde (29.09 mmol) was then added and stirring continued at room temperature for 24 h. Reaction was monitored by TLC. After completion of reaction, the mixture was poured over crushed ice and neutralized with 10 % HCl solution. The separated solid was filtered and washed well with water. Crude product was dried and recrystallized from ethanol to obtain the desired product in pure form.

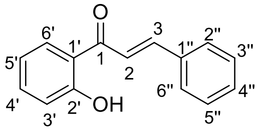

(E)-1-(2-Hydroxyphenyl)-3-phenylprop-2-en-1-one (15a) [1]

Yellow crystals. Yield: 92%. m.p. 87-88 ℃; ^{1}H-NMR (400 MHz, $CDCl_3$) δ: 6.93 (m, 1H, 5'-H), 7.02 (dd, *J* = 8.4 Hz, 1H, 2.0 Hz, 3'-H), 7.38 (m, 3H, 2"-H, 4"-H, 6"-H), 7.48 (m, 1H, 4'-H), 7.60 (m, 3H, 3-H, 3"-H, 5"-H), 7.89 (dd, *J* = 8.4, 1.2 Hz, 1H, 6'-H), 7.90 (d, *J* = 15.4 Hz, 1H, 2-H), 12.90 (s, 1H, OH); ^{13}C-NMR (101 MHz, $CDCl_3$) δ: 118.6 (C-3'), 118.8 (C-3), 120.0 (C-1'), 120.1 (C-5'), 128.6 (C-1"), 129.0 (C-6'), 129.6 (C-3", C-5"), 130.9 (C-4"), 134.5 (C-2", C-6"), 136.3 (C-4'), 145.4 (C-2), 163.5 (C-2'), 193.7 (C-1)

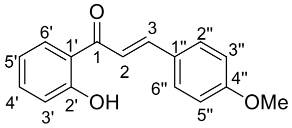

(E)-1-(2-Hydroxyphenyl)-3-(4-methoxyphenyl)prop-2-en-1-one (15b) [2]

Yellow crystals. Yield: 65%. m.p. 90-92 ℃; ^{1}H-NMR (400 MHz, $CDCl_3$) δ: 3.85 (s, 3H, 4'-OCH_3), 6.89 (m, 3H, 5'-H, 3"-H, 5"-H), 7.00 (dd, *J* = 8.4, 0.8 Hz, 3'-H), 7.47 (m, 1H, 4'-H), 7.53 (d, *J* = 15.7 Hz, 1H, 3-H), 7.61 (d, 2H, 2"-H, 6"-H), 7.88 (d, *J* = 15.7 Hz, 1H, 2-H), 7.90 (dd, *J* = 8.4, 2.0 Hz, 1H, 6'-H), 12.91 (s, 1H, OH); ^{13}C-NMR (101 MHz, $CDCl_3$) δ: 55.4 (C-OCH_3), 114.5 (C-3", C-5"), 117.6 (C-3'), 118.6 (C-3), 118.7 (C-1'), 120.1 (C-5'), 127.4 (C-1"), 129.5 (C-2", C-6"), 130.5 (C-6'), 136.1 (C-4'), 145.4 (C-2), 162.0 (C-4"), 163.5 (C-2'), 193.7 (C-1)

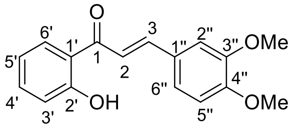

(E)-1-(2-Hydroxyphenyl)-3-(3,4-dimethoxyphenyl)prop-2-en-1-one (15c) [3]

Yellow crystals. Yield: 79%. m.p. 120-122 ℃; ^{1}H-NMR (400 MHz, $CDCl_3$) δ: 3.94 (s, 3H, 3'-OCH_3), 3.96 (s, 3H, 4'-OCH_3), 6.90 (m, 2H, 5"-H, 5'-H), 7.01 (dd, *J* = 8.4, 0.8 Hz, 1H, 6"-H), 7.17 (d, *J* = 1.6 Hz, 1H, 2"-H), 7.25 (dd, J = 8.4, 2.0 Hz, 1H, 3'-H), 7.45 (m, 1H, 4'-H), 7.50 (d, J = 15.2 Hz, 1H, 3-H), 7.86 (d, J = 15,6 Hz, 1H, 2-H), 7.92 (dd, J = 8.4, 1.2 Hz, 1H, 6'-H), 12.95 (s, 1H, OH); ^{13}C-NMR (101 MHz, $CDCl_3$) δ: 56.1 (2xC-OCH_3), 110.4 (C-5"), 111.2 (C-2"), 117.8 (C-3'), 118.6 (C-6"), 118.8 (C-3), 120.2 (C-1'), 123.7 (C-5'), 127.7 (C-1"), 129.6 (C-6'), 136.2 (C-4'), 145.7 (C-2), 149.4 (C-3"), 151.9 (C-4"), 163.6 (C-2'), 193.6 (C-1)

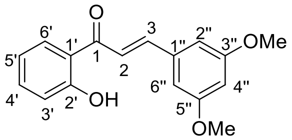

(E)-1-(2-Hydroxyphenyl)-3-(3,5-dimethoxyphenyl)prop-2-en-1-one (15d) [4]

Yellow crystals. Yield: 85%. m.p. 125-127 ℃; ^{1}H-NMR (360 MHz, CDCl$_3$) δ: 3.83 (s, 6H, 2xOCH$_3$), 6.53 (t, J = 3.6 Hz, 4”-H), 6.77 (d, 2H, 2”-H, 6”-H), 6.91 (m, 1H, 5’-H), 7.01 (d, J = 7.2 Hz, 1H, 3’-H), 7.47 (m, 1H, 4’-H), 7.56 (d, J = 14.4 Hz, 1H, 3-H), 7.79 (d, J = 14.4 Hz, 1H, 2-H), 7.88 (dd, J = 7.2, 3.6 Hz, 1H, 6’-H), 12.80 (s, 1H, OH); ^{13}C NMR (90 MHz, CDCl$_3$) δ: 55.3 (2xC-OCH$_3$), 102.9 (C-4”), 106.4 (C-2”, C-6”), 118.5 (C-3’), 118.7 (C-3), 119.8 (C-1’), 120.4 (C-5’), 129.5 (C-6’), 136.2 (C-1”), 145.3 (C-2), 160.9 (C-3”, C-5”), 163.4 (C-2’), 193.5 (C-1)

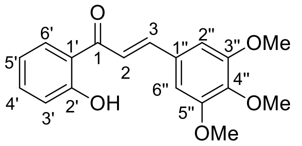

(E)-1-(2-Hydroxyphenyl)-3-(3,4,5-trimethoxyphenyl)prop-2-en-1-one (15e) [5]

Yellow crystals. Yield: 82%. m.p. 156-158 ℃; ^{1}H NMR (360 MHz, CDCl$_3$) δ: 3.92 (s, 3H, 4”-OCH$_3$), 3.93 (s, 6H, 2xOCH$_3$), 6.88 (s, 2H, 2”-H, 6”-H), 6.92 (m, 1H, 5’-H), 7.01 (dd, J = 10.8, 3.6 Hz, 1H, 3’-H), 7.47 (m, 1H, 4’-H), 7.51 (d, J = 18.0 Hz, 1H, 3-H), 7.81 (d, J = 18.0 Hz, 1H, 2-H), 7.92 (dd, J = 7.2, 3.6 Hz, 1H, 6’-H), 12.87 (s, 1H, OH); ^{13}C-NMR (90 MHz, CDCl$_3$) δ: 56.1 (2xC-OCH$_3$), 60.9 (C-OCH$_3$), 105.8 (C-2”, C-6”), 118.4 (C-3’), 118.7 (C-3), 119.0 (C-5’), 119.8 (C-1’), 129.5 (C-6’), 129.9 (C-1”, C-4”), 136.2 (C-6’), 145.5 (C-2), 153.3 (C-3”, C-5”), 163.4 (C-2’), 193.3 (C-1)

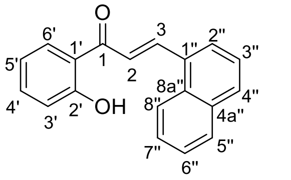

(E)-1-(2-Hydroxyphenyl)-3-(1-naphthyl)prop-2-en-1-one (15f) [6]

Yellow crystals Yield: 98 %. m.p. 107-109 °C, ^{1}H NMR (360 MHz, CDCl$_3$) δ: 6.95 (t, J = 7.9 Hz, 1 H, 5’-H), 7.04 (d, J = 8.3 Hz, 1 H, 3’-H), 7.55 (m, 4 H, 4’-H, 3”-H, 6”-H, 7”-H), 7.72 (d, J = 15.5 Hz, 1 H, 2-H), 7.93 (m, 4 H, 2”-H, 4”-H, 5”-H, 8”-H), 8.26 (d, J = 8.3 Hz, 1 H, 6’-H), 8.76 (d, J = 15.1 Hz, 1 H, 3-H), 12.87 (s, 1 H, OH). ^{13}C NMR (90 MHz, CDCl$_3$) δ: 118.8 (C-3’), 119.0 (C-5’), 120.1 (C-1’), 122.8 (C-2), 123.5 (C-8”), 125.4 (C-2”), 125.5 (C-3”), 126.5 (C-6”), 127,2 (C-7”), 128.9 (C-6’), 129,8 (C-4”), 131.3 (C-5”), 131.9 (C-8a”), 132.1 (C-4a”), 133.8 (C-1”), 136.6 (C-4’), 142.4 (C-3), 163.8 (C-2’), 193.6 (C-1).

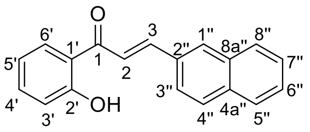

(E)-1-(2-Hydroxyphenyl)-3-(2-naphthyl)prop-2-en-1-one (15g) [6]

Yellow crystals. Yield: 96 %. m.p. 150-152 °C, ^{1}H NMR (360 MHz, CDCl$_3$) δ: 6.95 (t, J = 7.2 Hz, 1 H, 5’-H), 7.02 (d, J = 8.3 Hz, 1 H, 3’-H), 7.52 (m, 3 H, H-3”, 6”-H, 7”-H), 7.75 (m, 2 H, 3-H, 4’-H), 7.84 (m, 3 H, 4”-H, 5”-H, 8”-H), 7.93 (d, J = 8,3 Hz, 1 H, 6’-H), 8.03 (m, 2 H, 2-H, 1”-H), 12.88 (s, 1 H, OH). ^{13}C NMR (90 MHz, CDCl$_3$) δ: 118.1 (C-3’), 118.4 (C-2), 119.6 (C-1’), 119.6 (C-5’), 123.1 (C-3”), 126.4 (C-6”), 127.1 (C-7”), 127.3 (C-5”), 128.2 (C-1”), 128.3 (C-8”), 129.2 (C-4”), 130.6 (C-6’), 131.6 (C-2”), 132.8 (C-4a”), 134.0 (C-8a”), 135.9 (C-4’), 145.0 (C-3), 163.1 (C-2’), 193.1 (C-1).

1.2 General Procedure for the Synthesis of 4-Chromanone Derivatives (6a-g)

To a stirred solution of chalcone derivatives **15a-g** (22.30 mmol) in ethanol (300 mL) was added sodium acetate solution (25 g in 65 mL water). The reaction mixture was refluxed for 5 h. After cooling to room temperature, a part of the solvent was removed under reduced pressure, and then extracted with ethyl acetate more times. The combined organic layers were washed with water, dried over MgSO$_4$, filtered and solvent was evaporated under reduced pressure. The residue was triturated with cold diethyl ether. The precipitate was filtered and dried on air.

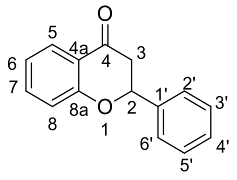

(±)-2-Phenylchroman-4-one (6a) [7]

White crystals. Yield: 70%. m.p. 75-77 ℃; ^{1}H NMR (400 MHz, $CDCl_3$) δ: 2.88 (dd, *J* = 16.9, 2.9 Hz, 1H, 3-H_a), 3.08 (dd, *J* = 16.9, 13.3 Hz, 1H, 3-H_b), 5.47 (dd, *J* = 13.3, 2.9 Hz, 1H, 2-H), 7.01 (m, 2H, 6-H, 8-H), 7.33 (m, 6H, 7-H, 2’-H, 3’-H, 4’-H, 5’-H, 6’-H), 7.93 (dd, *J* = 8.0, 1.8 Hz, 1H, 5-H); ^{13}C NMR (101 MHz, $CDCl_3$) δ: 44.6 (C-3), 79.6 (C-2), 118.1 (C-8), 120.9 (C-4a), 121.6 (C-6), 126.1 (C-5), 127.0 (C-2’, C-6’), 128.7 (C-4’), 128.8 (C-3’, C-5’), 136.2 (C-7), 138.7 (C-1’), 161.5 (C-8a), 191.9 (C-4)

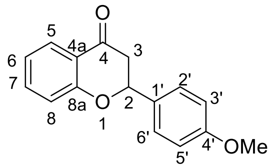

(±)-2-(4-Methoxyphenylphenyl)chromanone (6b) [8]

Pale yellow crystals. Yield: 75%. m.p. 86-88 ℃; ^{1}H NMR (400 MHz, $CDCl_3$) δ: 2,85 (dd, *J* = 16.8, 2.8 Hz, 1H, 3-H_a), 3,10 (dd, *J* = 13.3, 2.8 Hz, 1H, 3-H_b), 3.83 (s, 3H, OCH_3), 5.43 (dd, *J* = 13.3, 2.8 Hz, 1H, 2-H), 6.96 (m, 2H, 3’-H, 5’-H), 7.03 (m, 2H, 6-H, 8-H), 7.41 (m, 2H, 2’-H, 6’-H), 7.48 (m, 1H, 7-H), 7.92 (dd, *J* = 8.4, 1.6 Hz, 5’-H); ^{13}C NMR (101 MHz, $CDCl_3$) δ: 44.4 (C-3), 55.3 (C-OCH_3), 79.3 (C-2), 114.2 (C-3’, C-5’), 118.1 (C-8), 120.9 (C-4a), 121.4 (C-6), 127.0 (C-5), 127.7 (C-2’, C-6’), 130.7 (C-7), 136.1 (C-1’), 159.9 (C-8a), 161.6 (C-4’), 192.2 (C-4)

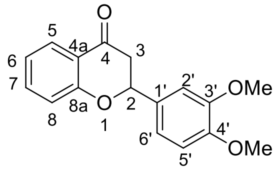

(±)-2-(3,4-Dimethoxyphenylphenyl)chromanone (6c) [9]

Pale yellow crystals. Yield: 54%. m.p. 120-122 ℃; ^{1}H NMR (400 MHz, $CDCl_3$) δ: 2.83 (dd, *J* = 16.8, 2.8 Hz, 1H, 3-H_a), 3.06 (dd, *J* = 17.2, 13.6 Hz, 1H, 3-H_b), 3.89 (d, 2H, 2xOCH_3), 5.39 (dd, *J* = 13.2, 2.8 Hz, 1H, 2-H), 6.89 (d, *J* = 8.4 Hz, 1H, 5’-H), 6.99 (m, 4H, 6-H, 8-H, 2’-H, 6’-H), 7.47 (m, 1H, 7-H), 7.90 (dd, *J* = 8.4, 2.0 Hz, 1H, 5-H); ^{13}C NMR (101 MHz, $CDCl_3$) δ: 44.5 (C-3), 55.9 (2xOCH_3), 79.5 (C-2), 109.4 (C-5’), 111.1 (C-2’), 118.1 (C-8), 118.8 (C-6’), 120.9 (C-4a), 121.6 (C-6), 127.0 (C-5), 131.2 (C-1’), 136.1 (C-7), 149.2 (C-4’), 149.4 (C-3’), 161.5 (C-8a), 192.0 (C-4)

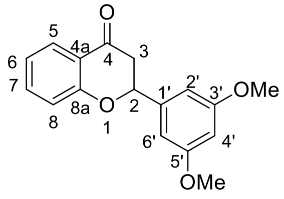

(±)-2-(3,5-Dimethoxyphenylphenyl)chromanone (6d) [10]

Pale yellow crystals. Yield: 59%. m.p. 87-88 ℃; ^{1}H NMR (400 MHz, $CDCl_3$) δ: 2.82 (dd, *J* = 16.0, 4.0 Hz, 1H, 3-H_a), 2.99 (dd, *J* = 16.0, 12.0 Hz, 1H, 3-H_b), 3.79 (s, 6H, 2xOCH_3), 5.35 (dd, *J* = 16.0, 4.0 Hz, 1H, 2-H), 6.44 (t, *J* = 4.0 Hz, 1H, 4’-H), 6.61 (d, 2H, 2’-H, 6’-H), 7.00 (m, 2H, 5-H, 7-H), 7.46 (m, 1H, 6-H), 7.89 (dd, *J* = 8.0, 0.8 Hz, 1H, 8-H); ^{13}C NMR (101 MHz, $CDCl_3$) δ: 44.5 (C-3), 55.2 (C-2xOCH_3), 79.3 (C-2), 100.2 (C-4’), 104.0 (C-2’, C-6’), 118.0 (C-8), 120.7 (C-4a), 121.4 (C-6), 126.8 (C-5), 136.0 (C-7), 140.9 (C-1’), 160.9 (C-3’, C-5’), 161.2 (C-8a), 191.6 (C-4)

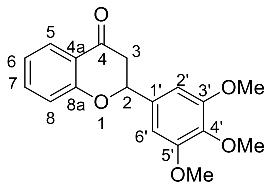

(±)-2-(3,4,5-Trimethoxyphenylphenyl)chromanone (6e) [10]

Pale yellow crystals. Yield: 65%. m.p. 124-126 ℃; ^{1}H NMR (360 MHz, $CDCl_3$) δ: 2.86 (dd, J = 14.4, 3.6 Hz, 1H, 3-H_a), 3.06 (dd, J = 18.0, 14.4 Hz, 1H, 3-H_b), 3.87 (m, 9H, 3xOCH_3), 5.39 (dd, J = 14.4, 3.6 Hz, 1H, 2-H), 6.71 (s, 2H, 2'-H, 6'-H), 7.07 (m, 2H, 2'-H, 6'-H), 7.50 (m, 1H, 6-H), 7.93 (dd, J = 7.2, 0.8 Hz, 1H, 8-H); ^{13}C NMR (90 MHz, $CDCl_3$) δ: 44.8 (C-3), 56.2 (C-2xOCH_3), 60.8 (C-OCH_3), 79.8 (C-2), 103.3 (C-2', C-6'), 110.0 (C-4'), 118.1 (C-8), 120.9 (C-4a), 121.7 (C-6), 127.1 (C-5), 134.3 (C-3', C-5'), 136.2 (C-7), 153.6 (C-1'), 161.4 (C-8a), 191.9 (C-4)

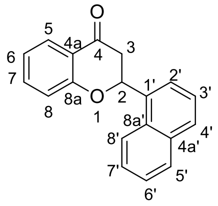

(±)-2-(1-Naphthyl)chromanone (6f) [6]

Yield: 54%. m.p. 99-101 ℃; ^{1}H NMR (360 MHz, $CDCl_3$) δ: 3.05 (dd, J = 16.9, 2.8 Hz, 1 H, 3-H_a), 3.24 (m, 1 H, 3-H_b), 6.19 (dd, J = 13.3, 2.8 Hz, 1 H, 2-H), 7.08 (m, 2 H, 6-H, 8-H), 7.53 (m, 4 H, 7-H, 2'-H, 3'-H, 7'-H), 7.75 (d, J = 6.8 Hz, 1 H, 4'-H), 7.89 (m, 2 H, 5'-H, 6'-H), 8.00 (m, 2 H, 5-H, 8'-H). ^{13}C NMR (90 MHz, $CDCl_3$) δ: 43.9 (C-3), 76.8 (C-2), 118.2 (C-8), 121.1 (C-4a), 121.7 (C-6), 122.7 (C-8'), 123.8 (C-3'), 125.9 (C-7'), 126.6 (C-2), 127.1 (C-5), 129.0 (C-4'), 129.3 (C-5'), 130.1 (C-8a'), 133.8 (C-4a'), 134.1 (C-1), 136.2 (C-7), 161.7 (C-8a), 192.2 (C-4).

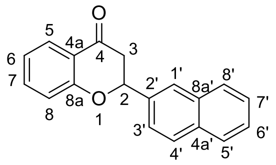

(±)-2-(2-Naphthyl)chromanone (6g) [6]

White crystals. Yield: 65%. m.p. 108-110 ℃; ^{1}H NMR (360 MHz, $CDCl_3$) δ: 2.90 (dd, J = 16.5, 2.5 Hz, 1H, 3-H_a), 3.09 (m, 1H, 3-H_b), 5.56 (dd, J = 13.3, 2.2 Hz, 1H, 2-H), 7.05 (m, 2H, 6-H, 8-H), 7.50 (m, 4H, 7-H, 6'-H, 7'-H, 8'-H), 7.88 (m, 5H, 5-H, 1'-H, 3'-H, 4'-H, 5'-H). ^{13}C NMR (90 MHz, $CDCl_3$) δ: 44.5 (C-3), 79.6 (C-2), 118.1 (C-8), 120.9 (C-4a), 121.6 (C-6), 123.6 (C-3'), 125.3 (C-1'), 126.5 (C-4', C-6'), 127.0 (C-7'), 127.7 (C-5), 128.1 (C-5'), 128.7 (C-8a'), 133.0 (C-4a'), 136.0 (C-2'), 136.1 (C-7), 161.4 (C-8a), 191.8 (C-4).

1.3 General Procedure for the Synthesis of oxime analogues (16a-g)

Flavanone derivative **6a-g** (15.61 mmol) and Hydroxylamine hydrochloride salt (23.41 mmol) were suspended in a mixture of Ethanol: Water 5:2 (40 mL). Then NaOH (5.20 mmol) was added at 0 ℃. Under reflux Hydroxylamine hydrochloride salt (15.61 mmol) and NaOH (5.20 mmol) were put to the reaction after every 45 minutes until the starting materiel was completely disappeared. After cooling the reaction to room temperature, the pH was ad*J*usted about 5 with concentrated HCl solution which provided a white suspension. Filtration and washing with water afforded the pure product.

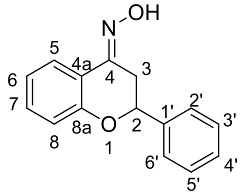

(*E*)-2-phenylchroman-4-one oxime (16a) [11]

White crystals. Yield: 97%. m.p 160-162 ℃; 1H-NMR (400 MHz, CDCl3, 25°C) δ: 2.74 (dd, *J* = 17.2, 12.8 Hz, 1 H, 3-Ha), 3.57 (dd, *J* = 17.2, 2.8 Hz, 1 H, 3-Hb), 5.09 (dd, *J* = 12.4, 2.4 Hz, 1 H, 2-H), 6.96 (m, 2 H, 6-H, 8-H), 7.27 (m, 1 H, 7-H), 7.36 (m, 6 H, 2'-H, 3'-H, 4'-H, 5'-H, 6'-H), 7.83 (d, *J* = 6.8 Hz, 1 H, 5-H), 8.89 (bs, 1 H, OH); 13C-NMR (101 MHz, CDCl3, 25 ℃) δ: 30.6 (C-3), 77.2 (C-2), 118.1 (C-4a), 118.2 (C-8), 121.8 (C-6), 124.0 (C-5), 126.3 (C-2', C-6'), 128.6 (C-4'), 128.8 (C-3', C-5'), 131.5 (C-7), 139.9 (C-1'), 150.4 (C-4), 156.9 (C-8a); HRMS (ESI) calcd. for C15H13NO2 [M+H]+ 240.102; found 239.09

(*E*)-2-(4-methoxyphenyl)chroman-4-one oxime (16b)

White crystals, Yield: 93%.: m.p. 171-173 ℃; ^{1}H-NMR (400 MHz, $CDCl_3$) δ: 2.75 (dd, *J* = 17.2, 12.4 Hz, 1 H, 3-Ha), 3.52 (dd, *J* = 17.2, 2.8 Hz, 1 H, 3-Hb), 3.83 (s, 3 H, 4'-OCH_3), 5.04 (dd, *J* = 12.4, 2.8 Hz, 1 H, 2-H), 6.93 (m, 4 H, 6-H, 8-H, 3'-H, 5'-H), 7.27 (m, 1 H, 7-H), 7.40 (d, 2 H, 2'-H, 6'-H), 7.82 (dd, *J* = 8.0, 1.6 Hz, 1 H, 5-H), 8.64 (bs, 1 H, OH); ^{13}C-NMR (101 MHz, $CDCl_3$) δ: 30.6 (C-3), 55.8 (C-OCH_3), 77.3 (C-2), 114.5 (C-3', C-5'), 118.4 (C-4a), 118.5 (C-8), 122.0 (C-6), 124.3 (C-5), 128.1 (C-2', C-6'), 131.8 (C-7), 132.3 (C-1'), 150.9 (C-4), 157.2 (C-8a), 160.2 (C-4'); HRMS (ESI) calcd. for $C_{16}H_{15}NO_3$ $[M+H]^+$ 270.113; found 270.111

(*E*)-2-(3,4-dimethoxyphenyl)chroman-4-one oxime (16c)

White crystals. Yield: 85%. m.p. 151-153 ℃; ^{1}H-NMR (400 MHz, $CDCl_3$) δ: 2.78 (dd, *J* = 17.2, 12.4 Hz, 1 H, 3-Ha), 3.54 (dd, *J* = 17.2, 3.2 Hz, 1 H, 3-Hb), 3.89 (d, 6 H, 2xOCH_3), 5.03 (dd, *J* = 12.4, 3.2 Hz, 1 H, 2-H), 6.87 (d, *J* = 8.0 Hz, 1 H, 5'-H), 6.95 (m, 4 H, 6-H, 8-H, 2'-H, 6'-H), 7.27 (m, 1 H, 7-H), 7.82 (dd, *J* = 8.0, 1.6 Hz, 1 H, 5-H), 9.32 (bs, 1 H, OH); ^{13}C-NMR (101 MHz, $CDCl_3$) δ: 30.4 (C-3), 56.0 (2xC-OCH_3), 77.1 (C-2), 109.5 (C-5'), 111.2 (C-2'), 118.0 (C-4a), 118.2 (C-8), 118.9 (C-6'), 121.7 (C-6), 123.9 (C-5), 131.4 (C-7), 132.3 (C-1'), 149.2 (C-4'), 150.4 (C-4, C-3'), 156.8 (C-8a); HRMS (ESI) calcd. for $C_{17}H_{17}NaNO_4$ $[M+Na]^+$ 322.105; found 322.105

(*E*)-2-(3,5-dimethoxyphenyl)chroman-4-one oxime (16d)

White crystals. Yield: 88%. m.p. 130-132 ℃; ^{1}H-NMR (400 MHz, $CDCl_3$) δ: 2.73 (dd, *J* = 17.2, 12.4 Hz, 1 H, 3-Ha), 3.55 (dd, *J* = 17.2, 2.8 Hz, 1 H, 3-Hb), 3.81 (s, 6 H, 2xOCH_3), 5.02 (dd, *J* = 12.4, 2.8 Hz, 1 H, 2-H), 6.45 (t, *J* = 2.0 Hz, 1 H, 4'-H), 6.64 (d, 2 H, 2'-H, 6'-H), 6.97 (m, 2 H, 6-H, 8-H), 7.27 (m, 1 H, 7-H), 7.82 (dd, *J* = 8.0, 1.6 Hz, 1 H, 5-H), 8.97 (bs, 1 H, OH); ^{13}C-NMR (101 MHz, $CDCl_3$) δ = 30.6 (C-3), 55.5 (2xC-OCH_3), 77.2 (C-2), 100.4 (C-4'), 104.3 (C-2', C-6'), 118.1 (C-4a), 118.2 (C-8), 121.8 (C-6), 124.0 (C-5), 131.5 (C-7), 142.2 (C-1'), 150.3 (C-4), 156.7 (C-8a), 161.2 (C-3', C-5'); HRMS (ESI) calcd. for $C_{17}H_{17}NaNO_4$ $[M+Na]^+$ 322.105; found 322.105

(*E*)-2-(3,4,5-trimethoxyphenyl)chroman-4-one oxime (16e)

White crystals. Yield: 95%. m.p. 165-167 ℃; ^{1}H-NMR (360 MHz, $CDCl_3$) δ: 2.76 (dd, *J* = 17.3, 12.6 Hz, 1 H, 3-Ha), 3.56 (dd, *J* = 17.3, 2.5 Hz, 1 H, 3-Hb), 3.87 (d, 9 H, 3xOCH_3), 5.02 (dd, *J* = 12.6, 2.5 Hz, 1 H, 2-H), 6.72 (s, 2 H, 2'-H, 6'-H), 7.00 (m, 2 H, 6-H, 8-H), 7.28 (t, *J* = 8.0 Hz, 1 H, 7-H), 7.83 (d, *J* = 7.9 Hz, 1 H, 5-H), 9.32 (bs, 1 H, OH); ^{13}C-NMR (91 MHz, $CDCl_3$) δ: 30.7 (C-3), 56.2 (2xC-OCH_3), 61.0 (C-OCH_3), 77.4 (C-2), 103.4 (C-2', C-6'), 118.0 (C-4a), 118.2 (C-8), 121.8 (C-6), 123.9 (C-5), 131.5 (C-7), 135.4 (C-1'), 138.0 (C-4'), 150.1 (C-4), 153.5 (C-3', C-5'), 156.6 (C-8a); HRMS (ESI) calcd. for $C_{18}H_{19}NO_5$ $[M+H]^+$ 330.134; found 330.134

(*E*)-2-(naphthalen-1-yl)chroman-4-one oxime (16f)

White crystals. 81%. m.p. 219-221 ℃; ^{1}H-NMR (400 MHz, DMSO-d6) δ: 2.85 (dd, *J* = 17.2, 12.0 Hz, 1 H, 3-Ha), 3.51 (dd, *J* = 17.2, 3.2 Hz, 1 H, 3-Hb), 5.93 (dd, *J* = 11.6 , 2.8 Hz, 1 H, 2-H), 6.97 (m, 2 H, 6-H, 8-H), 7.29 (m, 1 H, 7-H), 7.55 (m, 3 H, 2'-H, 3'-H, 7'-H), 7.74 (d, *J* = 7.2 Hz, 1 H, 5-H), 7.89 (d, *J* = 6.8 Hz, 1 H, 6'-H), 7.94 (m, 2 H, 4'-H, 5'-H), 8.18 (d, *J* = 7.2 Hz, 1 H, 8'-H), 11.4 (s, 1 H, OH); ^{13}C-NMR (101 MHz, DMSO-d6) δ: 29.2 (C-3), 73.6 (C-2), 117.7 (C-8), 119.2 (C-4a), 121.5 (C-6), 123.4 (C-8'), 123.5 (C-5), 123.9 (C-2'), 125.4 (C-3'), 125.8 (C-7'), 126.5 (C-6'), 128.7 (C-4', C-5'), 130.1 (C-8a'), 130.5

(C-7), 133.4 (C-4a'), 135.4 (C-1'), 147.4 (C-4), 155.9 (C-8a); HRMS (ESI) calcd. for $C_{19}H_{15}NO_2$ $[M+H]^+$ 290.118; found 290.117

(*E*)-2-(naphthalen-2-yl)chroman-4-one oxime (16g)

White crystals. Yield: 98%. m.p. 203-205 ℃; ^{1}H-NMR (400 MHz, DMSO-d6) δ: 2.80 (dd, *J* = 17.2, 12.0 Hz, 1 H, 3-Ha), 3.44 (dd, *J* = 17.2, 3.2 Hz, 1 H, 3-Hb), 5.34 (dd, *J* = 12.0, 3.2 Hz, 1 H, 2-H), 6.99 (m, 2 H, 6-H, 8-H), 7.71 (m, 1 H, 7-H), 7.29 (m, 2 H, 3'-H, 7'-H), 7.66 (dd, *J* = 8.4, 1.6 Hz, 1 H, 5-H), 7.85 (dd, *J* = 8.0, 1.6 Hz, 1 H, 6'-H), 7.93 (m, 3 H, 4'-H, 5'-H, 8'-H), 8.03 (s, 1 H, 1'-H), 11.38 (s, 1 H, OH); ^{13}C-NMR (101 MHz, DMSO-d6) δ: 29.8 (C-3), 76.4 (C-2), 117.7 (C-8), 119.0 (C-4a), 121.5 (C-6), 123.4 (C-5), 124.4 (C-1'), 125.2 (C-8'), 126.4 (C-3', C-7'), 127.6 (C-6'), 128.0 (C-5'), 128.2 (C-4'), 130.6 (C-7), 132.7 (C-8a'), 132.7 (C-4a'), 137.5 (C-2'), 147.3 (C-4), 155.7 (C-4); HRMS (ESI) calcd. for $C_{19}H_{15}NO_2$ $[M+H]^+$ 290.118; found 290.118

1.4 General Procedure for Neber rearrangement (*rac-cis*-1a-g, *rac-trans*-1a-g, 17a-g)

Tosyl oxime derivative **5a-g** (8.895 mmol) was dissolved in anhydrous Toluene (50 mL) under inert atmosphere and then 10.9 mL NaOEt (940 mg in 50 mL EtOH) was dropped to the solution. Stirring for 1 day at room temperature afforded an orange suspension. The suspension was filtered through cellite and washed with EtOH. Concentration of the filtrate under vacuum provided the crude product as an orange oil. Then it was solved in CH2Cl2 and 3 N HCl solution (3 mL) was added to it. After 2 hours stirring at room temperature resulted in an orange suspension. Filtration and washing with Acetone provided the cis relative configurational product as white powder. Then the filtrate was thoroughly concentrated under reduced pressure and trituration with Acetone afforded the trans relative configurational product as off-white powder. The residue filtrate was purified after concentration by column chromatography using an eluent of Toluene : Ethyl Acetate (4:1). The benzoxazole derivate was obtained by this procedure.

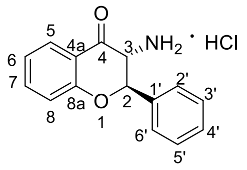

(±)-(2*R,3*R**)-3-amino-2-phenyl-2,3-dihydro-4H-chroman-4-one hydrochloride (*rac-trans*-1a) [12]**

off-White solid. Yield: 30%. m.p. 197-199 ℃; 1H-NMR (400 MHz, DMSO-d6, 25°C) δ: 5.01 (d, *J* = 12.4 Hz, 1 H, 3-H), 5.77 (d, *J* = 12.4 Hz, 1 H, 2-H), 7.13 (d, *J* = 8 Hz, 1 H, 8-H), 7.20 (m, 1 H, 6-H), 7.50 (m, 3 H, 3'-H, 4'-H, 5'-H), 7.68 (m, 3 H, 7-H, 2'-H, 6'-H), 7.87 (dd, *J* = 8.0, 1.6 Hz, 1 H, 5-H), 8.72 (bs, 3 H, NH_3); ^{13}C-NMR (101 MHz, DMSO-d6, 25°C) δ: 55.7 (C-3), 80.2 (C-2), 118.0 (C-8), 118.5 (C-4a), 122.5 (C-6), 126.9 (C-5), 128.6 (C-2', C-6'), 128.8 (C-3', C-5'), 129.9 (C-4'), 134.3 (C-1'), 137.6 (C-7), 160.8 (C-8a), 187.6 (C-4); HRMS (ESI) calcd. for $C_{15}H_{13}NO_2$ $[M+H]^+$ 240.102; found 240.101

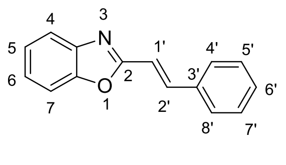

2-(*E*)-2-phenylethenyl-1,3-benzoxazole (17a) [13]

Pale yellow crystals. Yield: 15%. m.p. 62-64 ℃; ^{1}H-NMR (400 MHz, $CDCl_3$) δ: 7.05 (d, *J* = 16.4 Hz, 1 H, 2'-H), 7.30 (m, 2 H, 4-H, 7-H), 7.36 (m, 3 H, 5'-H, 6'-H, 7'-H), 7.50 (m, 1 H, 5-H), 7.57 (d, 2 H, 4'-H, 8'-H), 7.69 (m, 1 H, 6-H), 7.76 (d, *J* = 16.4 Hz, 1 H, 1'-H); ^{13}C-NMR (101 MHz, $CDCl_3$) δ: 110.4 (C-2'), 114.0 (C-5), 119.9 (C-6), 124.6 (C-4), 125.3 (C-7), 127.6 (C-5', C-7'), 129.0 (C-4', C-8'), 129.9 (C-6'), 135.2 (C-3'), 139.6 (C-1'), 142.2 (C-3a), 150.5 (C-7a), 162.9 (C-2); HRMS (ESI) calcd. for $C_{15}H_{11}NO$ $[M+H]^+$ 222.092; found 222.089

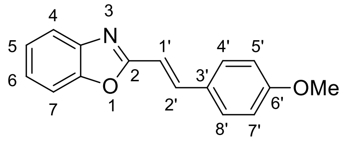

2-[(*E*)-2-(4-methoxyphenyl)ethenyl]-1,3-benzoxazole (17b) [14]

White chrystals. Yield: 20%. m.p. 128-130 ℃; ^{1}H-NMR (400 MHz, $CDCl_3$, 25°C) δ: 3.79 (s, 3 H, OCH_3), 6.88 (m, 3 H, β-H, 3’-H, 5’-H), 7.27 (m, 2 H, 4-H, 7-H), 7.46 (m, 3 H, 5-H, 2’-H, 6’-H), 7.67 (m, 2 H, α-H, 6-H); ^{13}C-NMR (101 MHz, $CDCl_3$, 25°C) δ: 55.4 (C-OCH_3), 110.2 (C-5), 111.5 (C-β), 114.4 (C-3’, C-5’), 119.7 (C-6), 124.4 (C-4), 124.9 (C-7), 127.9 (C-1’), 129.1 (C-2’, C-6’), 139.1 (C-β), 142.3 (C-3a), 150.4 (C-7a), 161.0 (C-4’), 163.2 (C-2); HRMS (ESI) calcd. for $C_{16}H_{13}NO_2$ $[M+H]^+$ 252.102; found

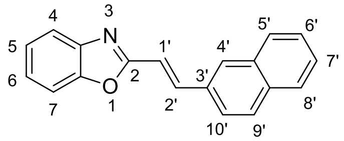

2-[(*E*)-2-naphthlaen-2-ylethenyl]-1,3-benzoxazole (17g) [13]

White crystals. 11%. m.p. 129-131 ℃; ^{1}H-NMR (400 MHz, $CDCl_3$) δ: 7.11 (d, J = 16.4 Hz, 1 H, 2’-H), 7.28 (m, 2 H, 4-H, 7-H), 7.45 (m, 3 H, 5-H, 4’-H, 8’-H), 7.68 (m, 2 H, 6-H, 7’-H), 7.77 (m, 3 H, 5’-H, 6’-H, 9’-H), 7.90 (m, 2 H, 10’-H, 1’-H); ^{1}H-NMR (400 MHz, $CDCl_3$) δ: 110.4 (C-5), 114.1 (C-2’), 120.0 (C-6), 123.2 (C-10’), 124.6 (C-4), 125.3 (C-7), 126.8 (C-9’), 127.1 (C-4’), 127.9 (C-8’), 128.5 (C-7’), 128.8 (C-6’), 129.2 (C-5’), 132.7 (C-5a’), 133.5 (C-9a’), 134.0 (C-3’), 139.5 (C-1’), 142.3 (C-3a), 150.5 (C-7a), 162.9 (C-2); HRMS (ESI) calcd. for $C_{19}H_{13}NO$ $[M+H]^+$ 272.107; found 272.107

1.5 References of known compounds

[1] Krohn, K.; Ahmed, I.; John, M. Enantioselective Synthesis of Flavan-3-ols Using a Mitsunobu Cyclization. *Synthesis* **2009**, 10.1055/s-0028-1083361, 779-786, doi:10.1055/s-0028-1083361.

[2] Jagadeesh, M.; Lavanya, M.; Babu, B.H.; Hong, K.; Ma, R.; Kim, J.; Kim, T.K. Synthesis and detailed spectroscopic characterization of various hydroxy-functionalized fluorescent chalcones: A combined experimental and theoretical study. *Spectroc. Acta Pt. A-Molec. Biomolec. Spectr*. **2015**, 150, 557-564, doi:10.1016/j.saa.2015.05.085.

[3] Li, X.; Chen, G.L.; Zhang, X.J.; Zhang, Q.; Zheng, S.L.; Wang, G.D.; Chen, Q.H. A new class of flavonol-based anti-prostate cancer agents: Design, synthesis, and evaluation in cell models. *Bioorg. Med. Chem. Lett*. **2016**, 26, 4241-4245, doi:10.1016/j.bmcl.2016.07.050.

[4] Britton, R.G.; Horner-Glister, E.; Pomenya, O.A.; Smith, E.E.; Denton, R.; Jenkins, P.R.; Steward, W.P.; Brown, K.; Gescher, A.; Sale, S. Synthesis and biological evaluation of novel flavonols as potential anti-prostate cancer agents. *Eur. J. Med. Chem*. **2012**, 54, 952-958, doi:10.1016/j.ejmech.2012.06.031.

[5] Dias, T.A.; Duarte, C.L.; Lima, C.F.; Proenca, M.F.; Pereira-Wilson, C. Superior anticancer activity of halogenated chalcones and flavonols over the natural flavonol quercetin. Eur. J. Med. Chem. **2013**, 65, 500-510, doi:10.1016/j.ejmech.2013.04.064.

[6] Muller, B.M.; Litberg, T.J.; Yocum, R.A.; Pniewski, C.A.; Adler, M.J. Extended Aromatic and Heteroaromatic Ring Systems in the Chalcone-Flavanone Molecular Switch Scaffold. *J. Org. Chem*. **2016**, 81, 5775-5781, doi:10.1021/acs.joc.6b00986.

[7] Safavi, M.; Esmati, N.; Ardestani, S.K.; Emami, S.; Ajdari, S.; Davoodi, J.; Shafiee, A.; Foroumadi, A. Halogenated flavanones as potential apoptosis-inducing agents: Synthesis and biological activity evaluation. *Eur. J. Med. Chem*. **2012**, 58, 573-580, doi:10.1016/j.ejmech.2012.10.043.

[8] Chimenti, F.; Fioravanti, R.; Bolasco, A.; Chimenti, P.; Secci, D.; Rossi, F.; Yanez, M.; Orallo, F.; Ortuso, F.; Alcaro, S., et al. A new series of flavones, thioflavones, and flavanones as selective monoamine oxidase-B inhibitors. *Bioorg. Med. Chem.* **2010**, 18, 1273-1279, doi:10.1016/j.bmc.2009.12.029.

[9] Cabrera, M.; Simoens, M.; Falchi, G.; Lavaggi, M.L.; Piro, O.E.; Castellano, E.E.; Vidal, A.; Azqueta, A.; Monge, A.; de Cerain, A.L., et al. Synthetic chalcones, flavanones, and flavones as antitumoral agents: Biological evaluation and structure-activity relationships. *Bioorg. Med. Chem*. **2007**, 15, 3356-3367, doi:10.1016/j.bmc.2007.03.031.

[10] Kondhare, D.D.; Gyananath, G.; Tamboli, Y.; Kumbhar, S.S.; Choudhari, P.B.; Bhatia, M.S.; Zubaidha, P.K. An efficient synthesis of flavanones and their docking studies with aldose reductase. *Med. Chem. Res*. **2017**, 26, 987-998, doi:10.1007/s00044-017-1813-1.

[11] Zhu, Z.Z.; Tang, X.D.; Cen, J.H.; Li, J.X.; Wu, W.Q.; Jiang, H.F. Copper-catalyzed synthesis of thiazol-2-yl ethers from oxime acetates and xanthates under redox-neutral conditions. *Chem. Commun*. **2018**, 54, 3767-3770, doi:10.1039/c8cc00445e.

[12] O'Brien, C.; Philbin, E.M.; Ushioda, S.; Wheeler, T.S. The synthesis of 3-aminoflavanones. *Tetrahedron* **1963**, *19*, 373-377, doi:10.1016/S0040-4020(01)99183-5

[13] Lee, W.C.; Wang, T.H.; Ong, T.G. Ligand promoted Pd-catalyzed dehydrogenative alkenylation of hetereoarenes. *Chem. Commun*. **2014**, 50, 3671-3673, doi:10.1039/c3cc48750d.

[14] Neumann, K.T.; Lindhardt, A.T.; Bang-Andersen, B.; Skrydstrup, T. Access to 2-(Het)aryl and 2-Styryl Benzoxazoles via Palladium-Catalyzed Aminocarbonylation of Aryl and Vinyl Bromides. *Organic Letters* **2015**, 17, 2094-2097, doi:10.1021/acs.orglett.5b00642.

2 1D- and 2D-NMR spectra

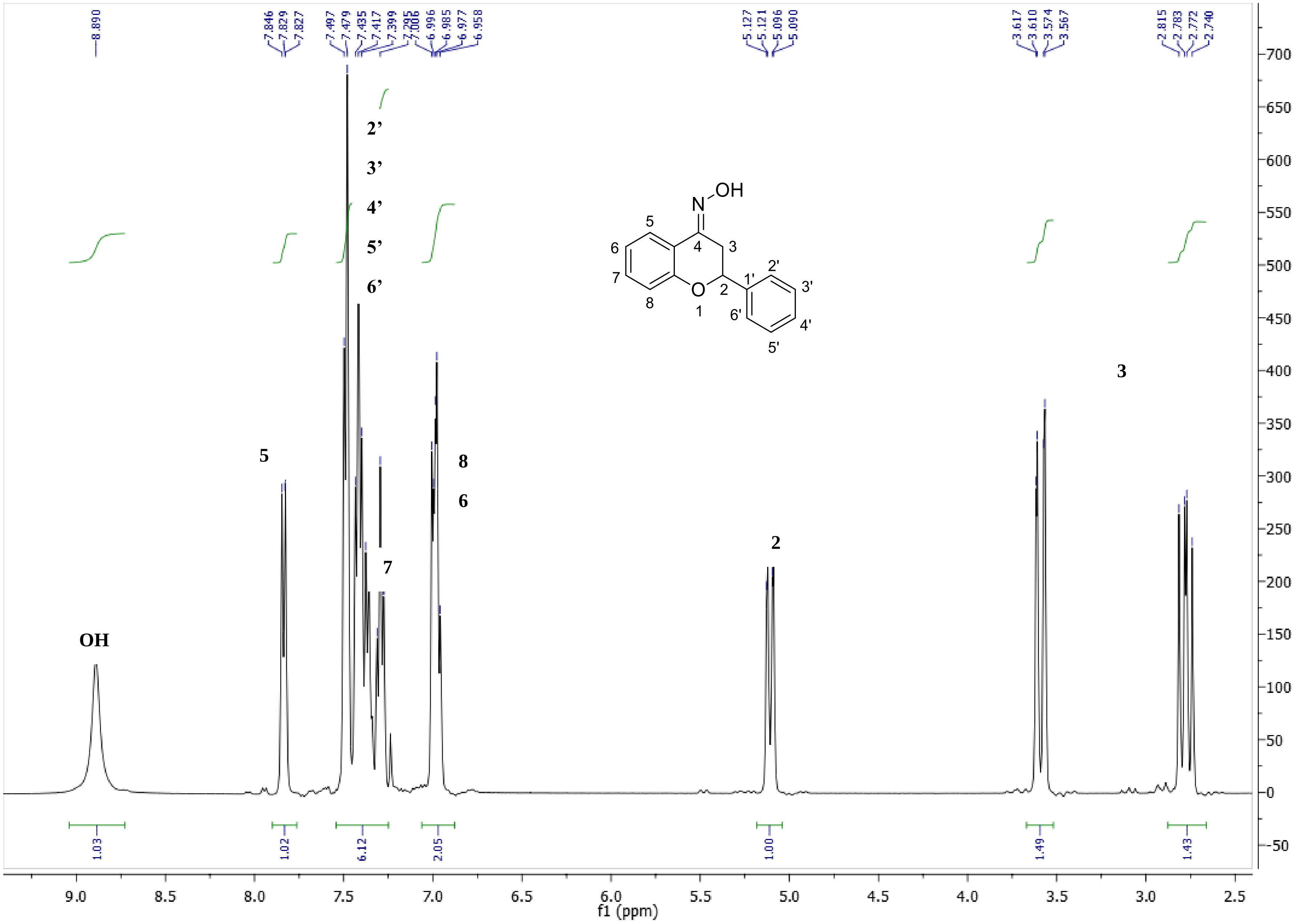

Figure S1. ^{1}H-NMR spectrum of **16a** in $CDCl_3$

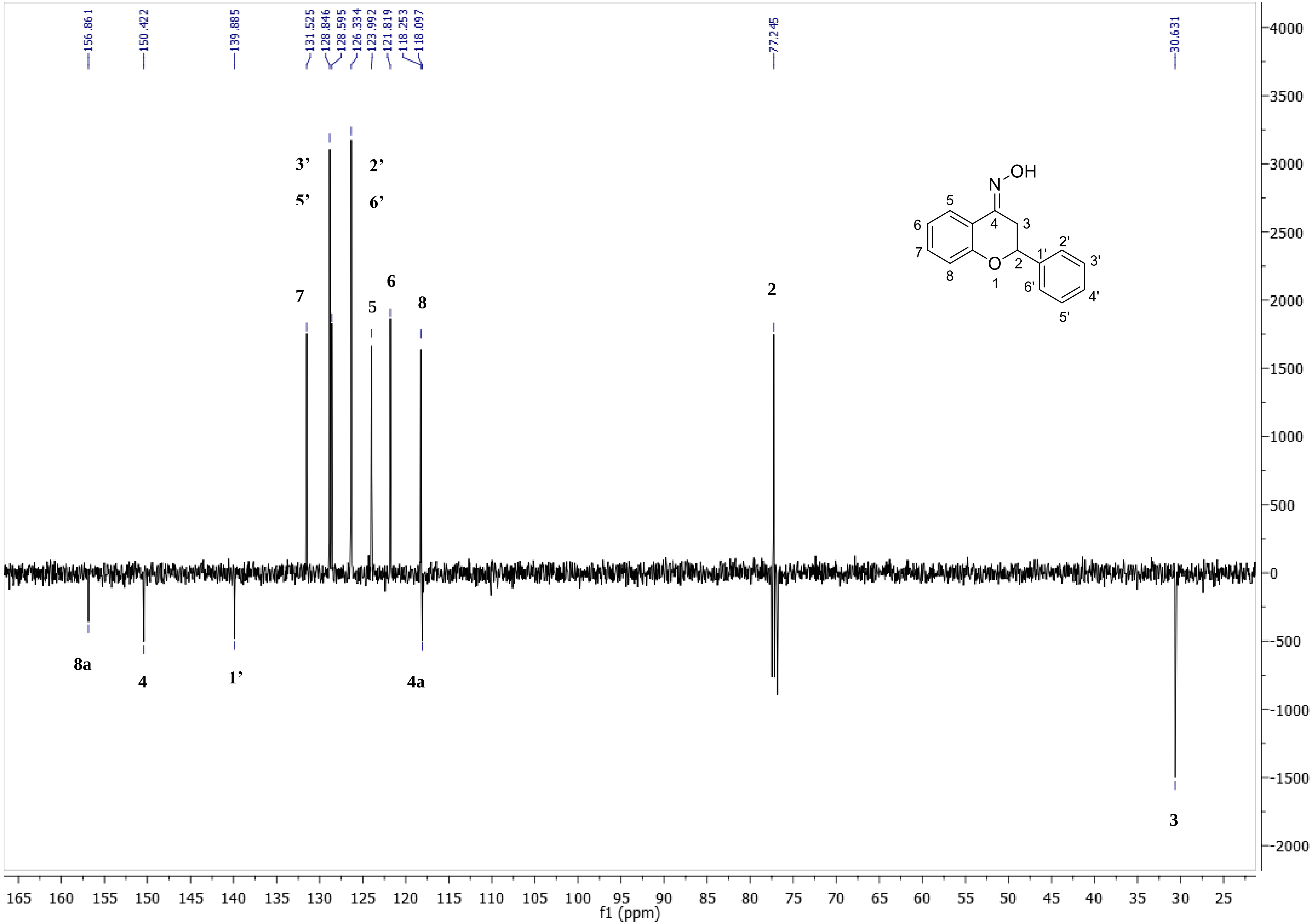

Figure S2. ^{13}C-NMR spectrum of **16a** in $CDCl_3$

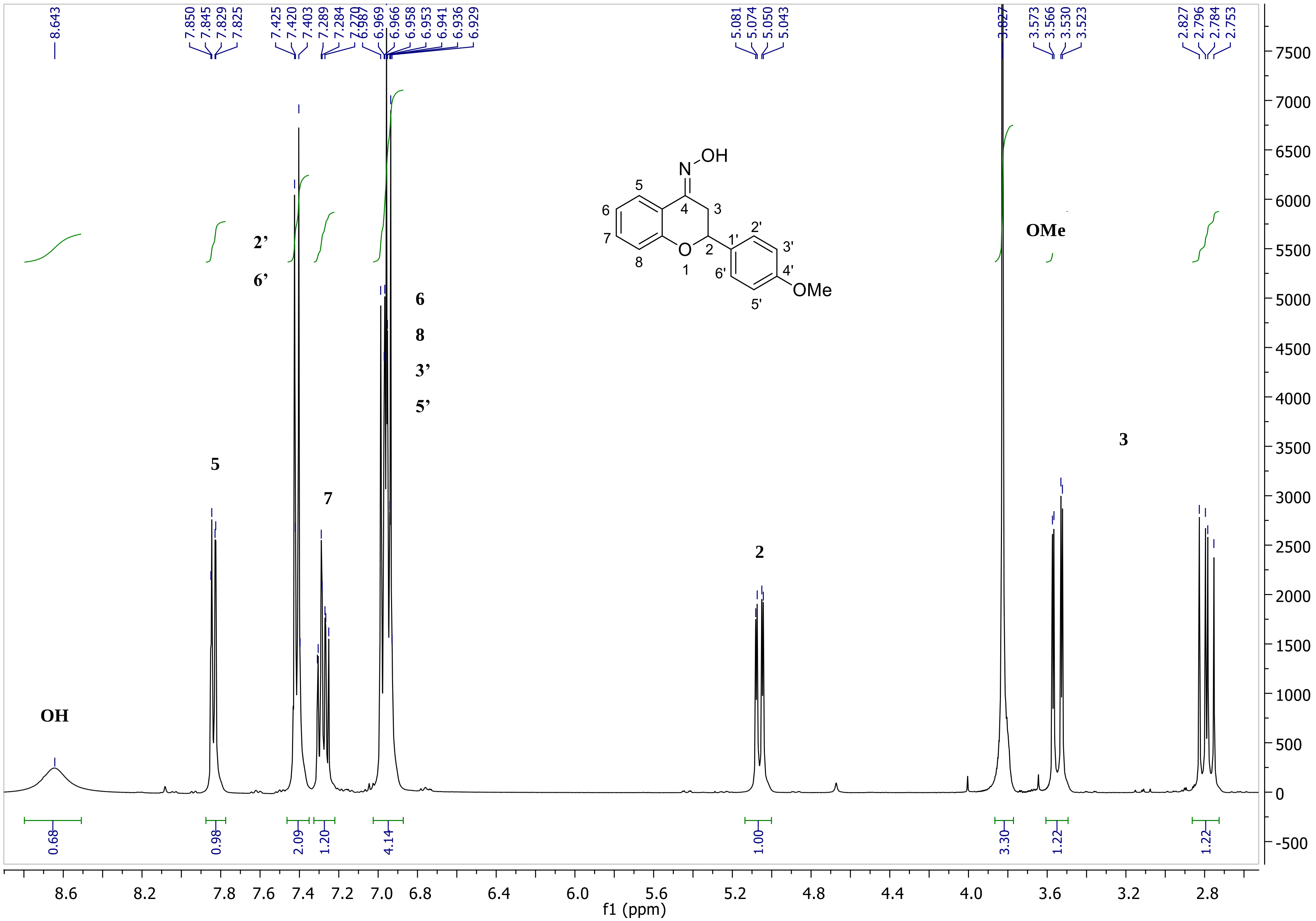

Figure S3. ^{1}H-NMR spectrum of **16b** in $CDCl_3$

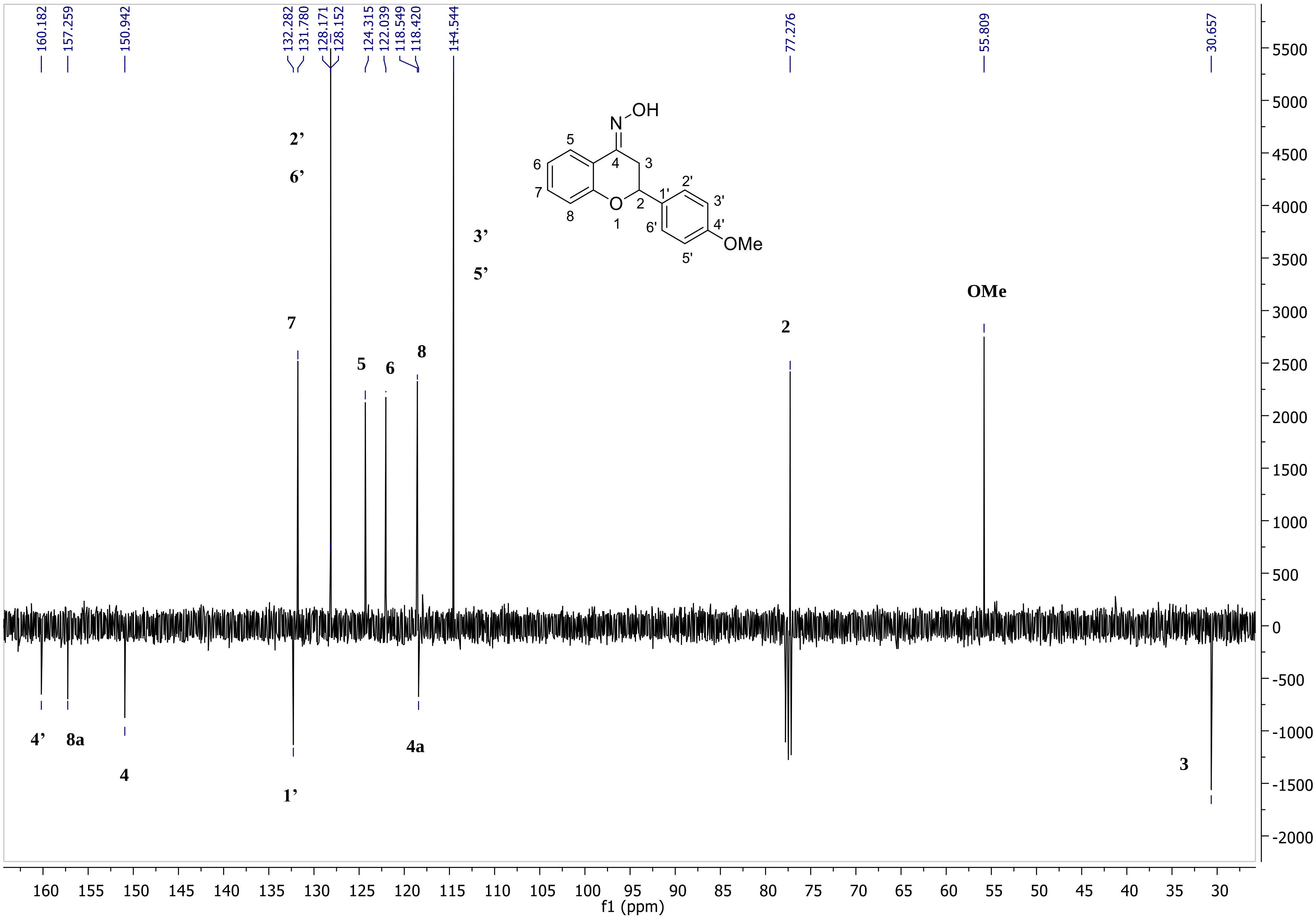

Figure S4. ^{13}C-NMR spectrum of **16b** in $CDCl_3$

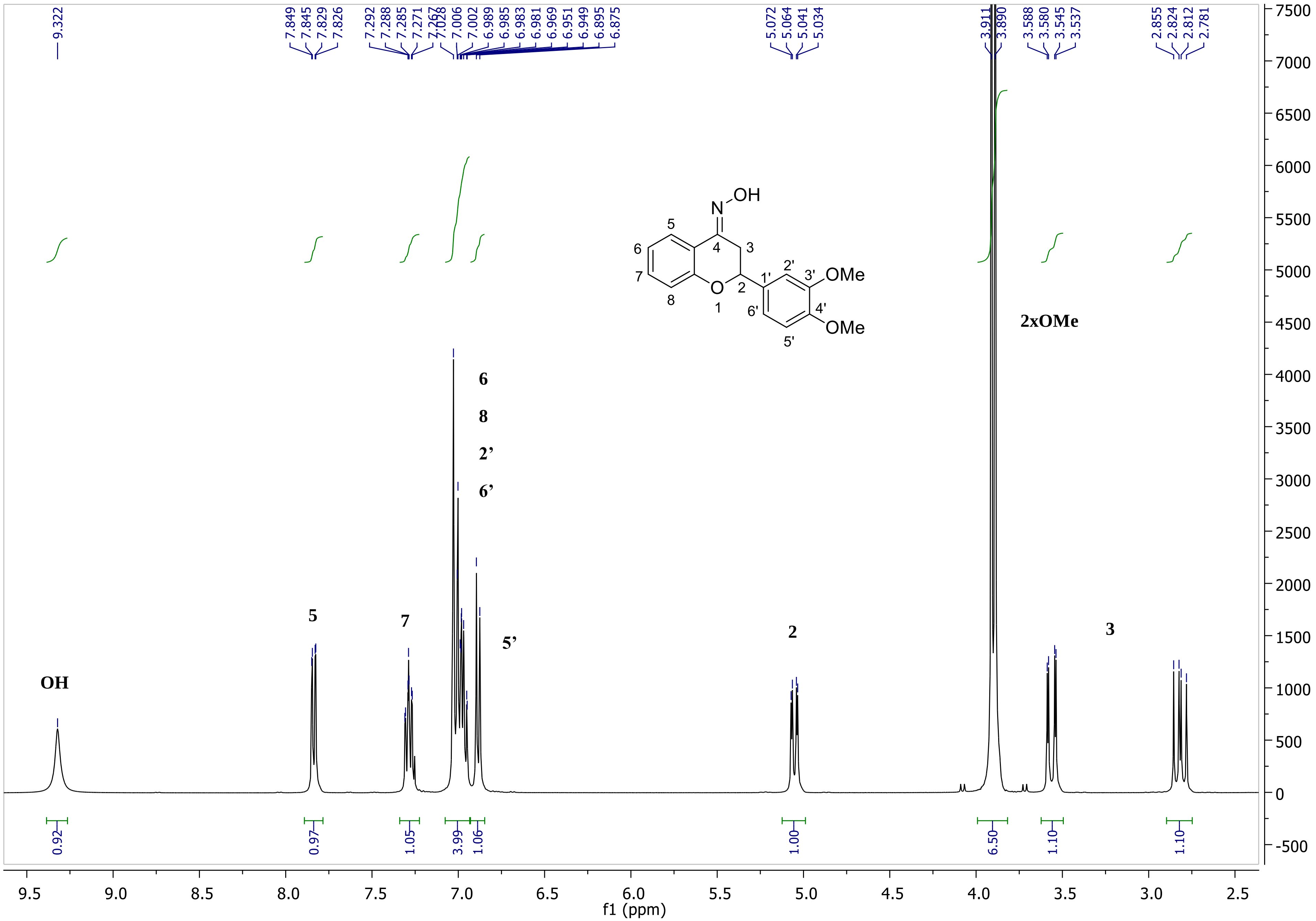

Figure S5. ^{1}H-NMR spectrum of **16c** in $CDCl_3$

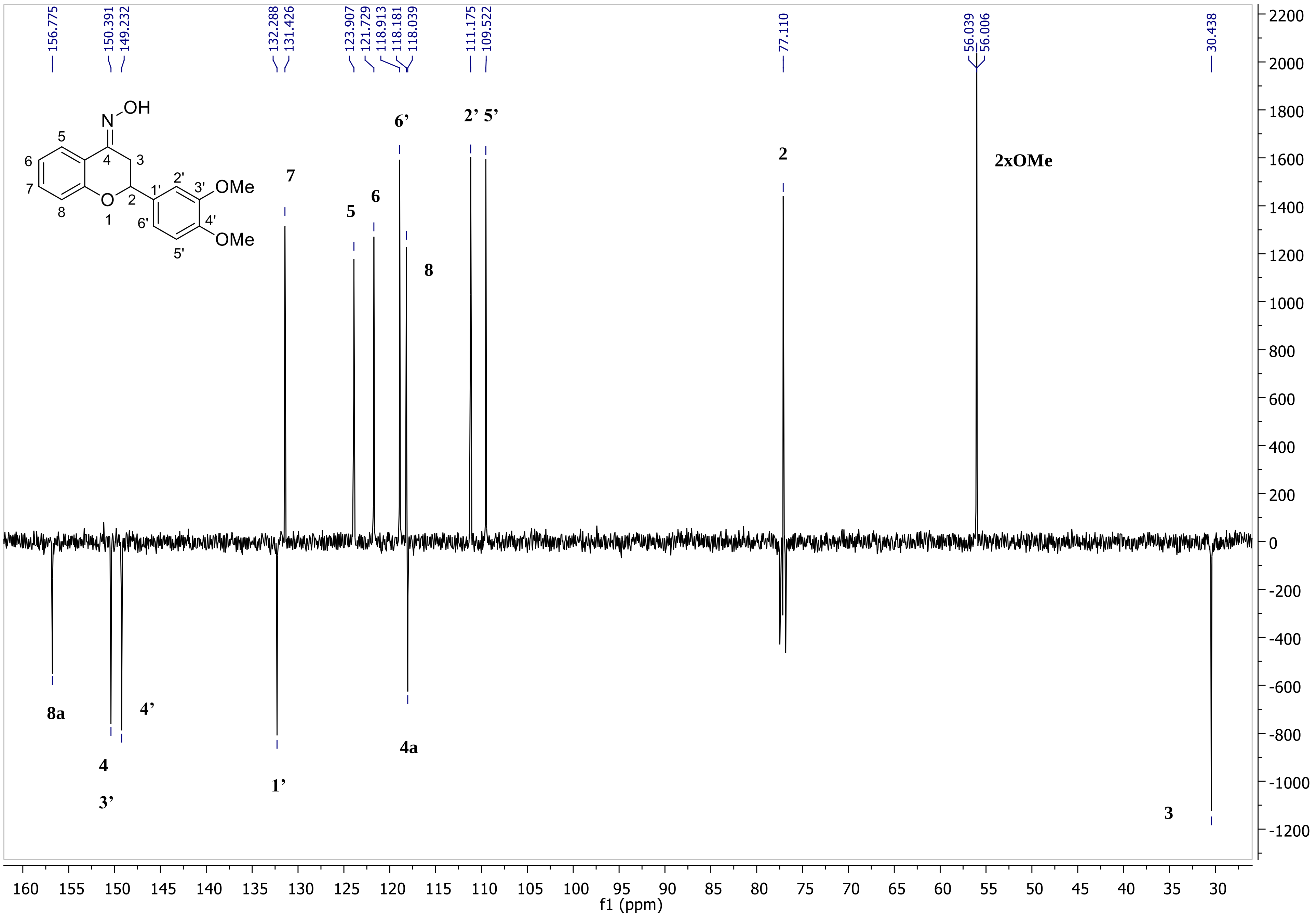

Figure S6. ^{13}C-NMR spectrum of **16c** in $CDCl_3$

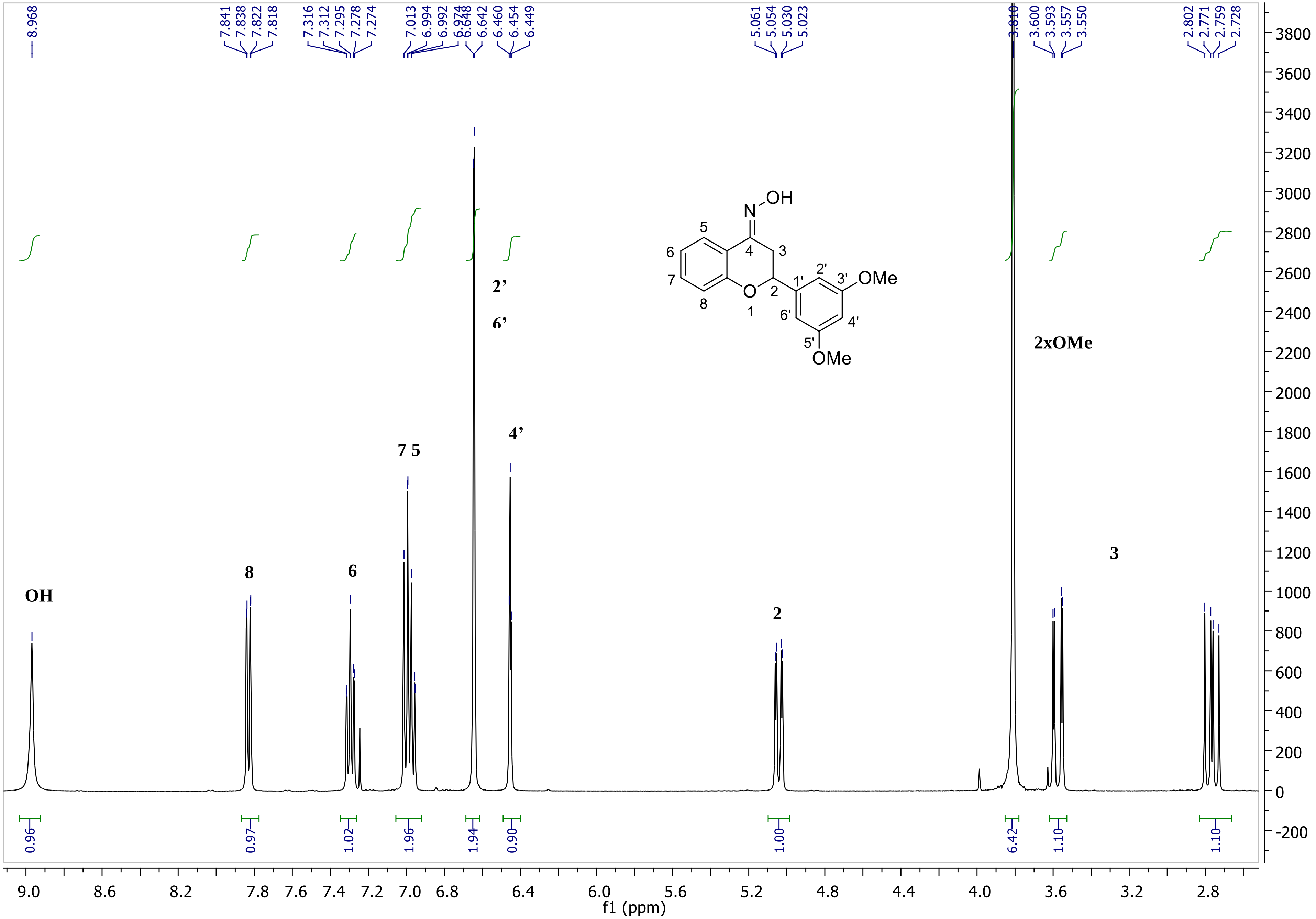

Figure 7. ^{1}H-NMR spectrum of **16d** in $CDCl_3$

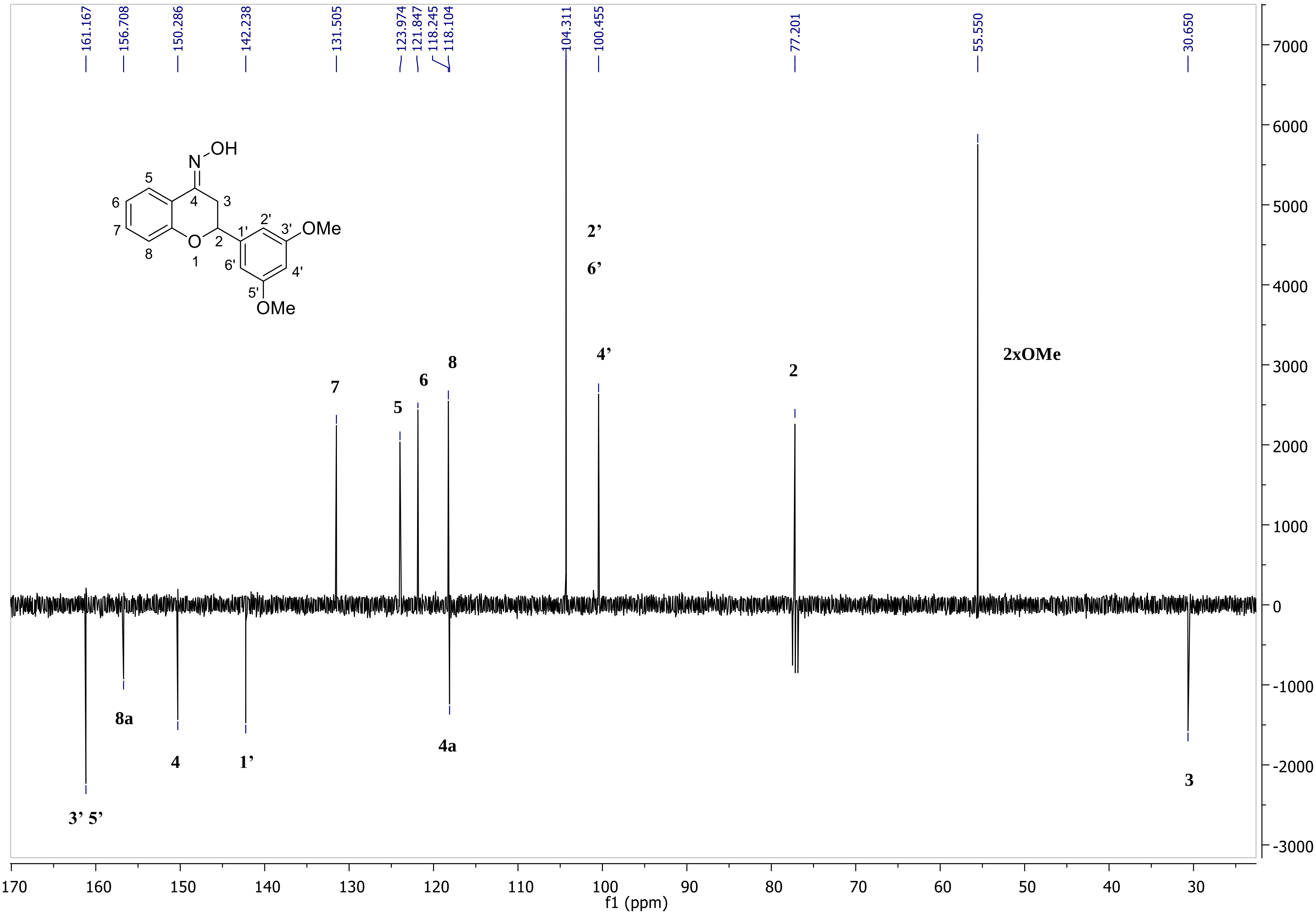

Figure S8. ^{13}C-NMR spectrum of **16d** in $CDCl_3$

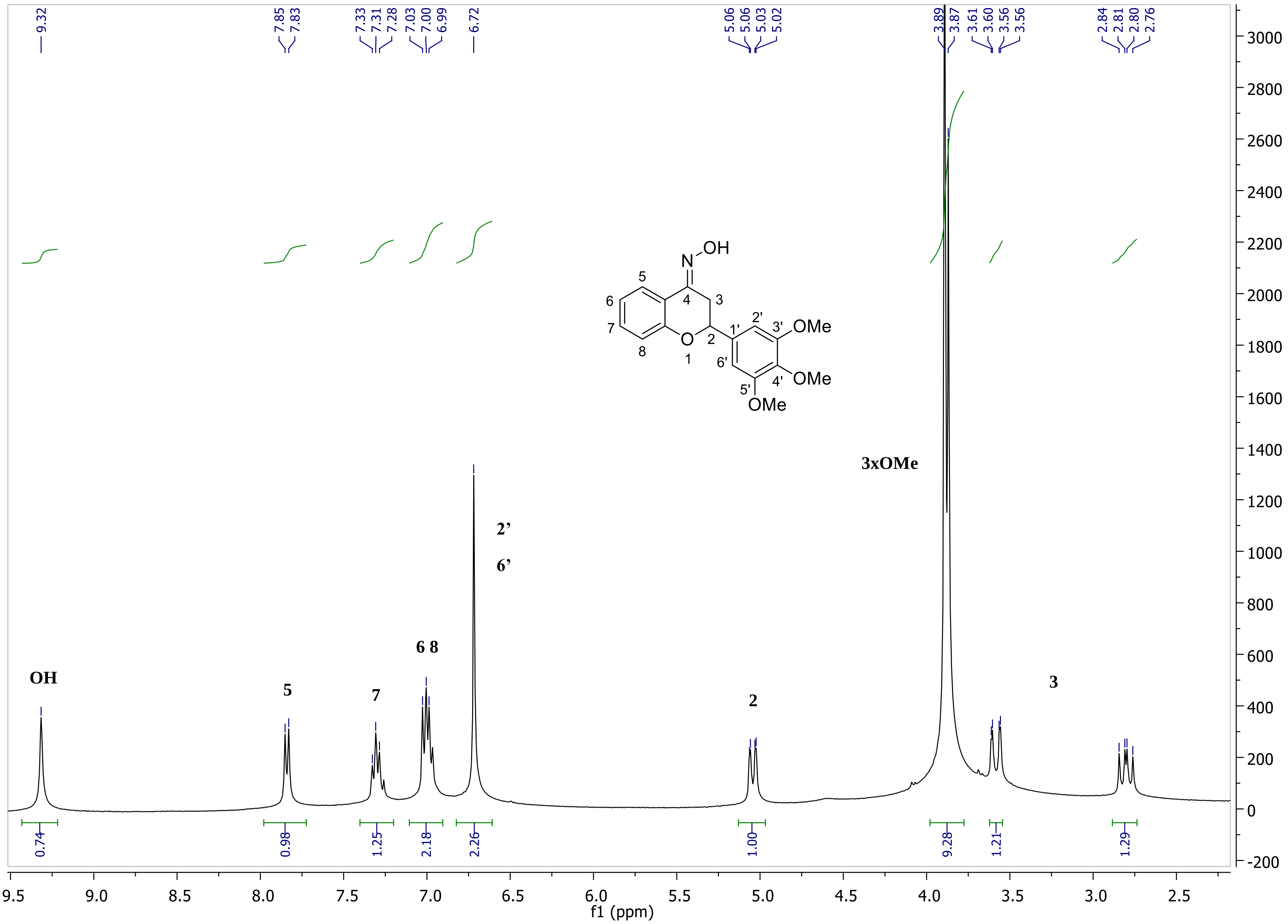

Figure S9. ^{1}H-NMR spectrum of **16e** in $CDCl_3$

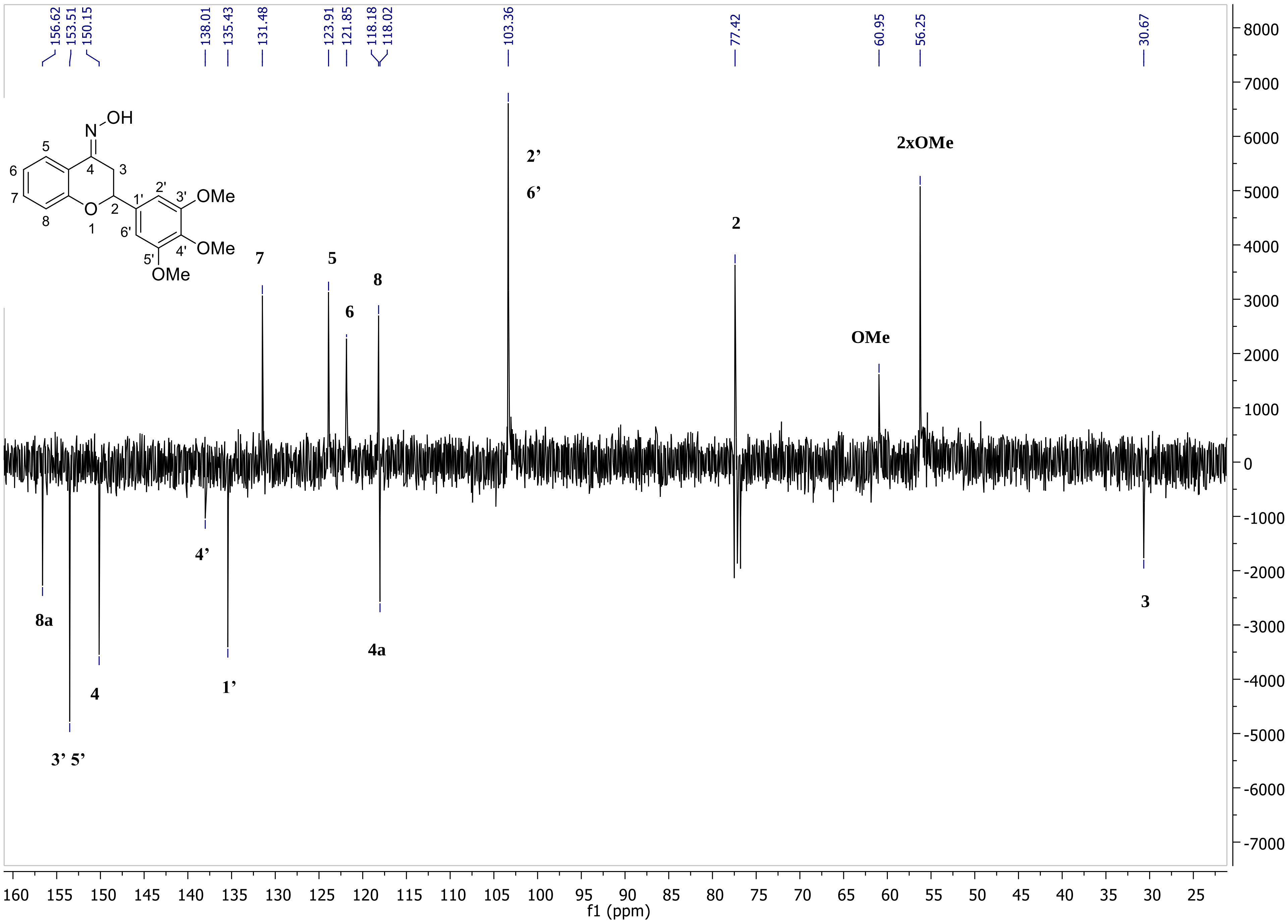

Figure S10. ^{13}C-NMR spectrum of **16e** in $CDCl_3$

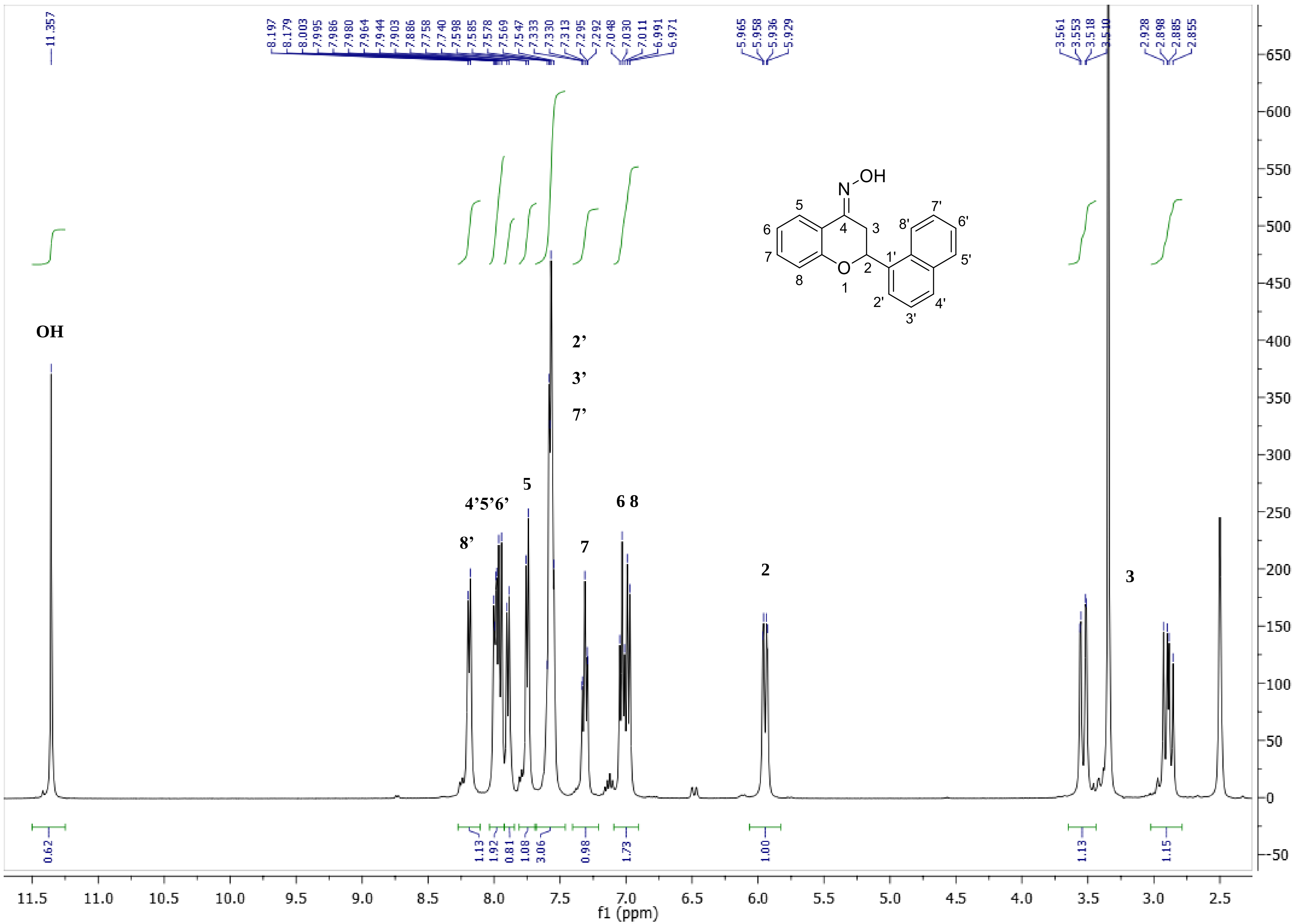

Figure S11. ^{1}H-NMR spectrum of **16f** in DMSO-d_6

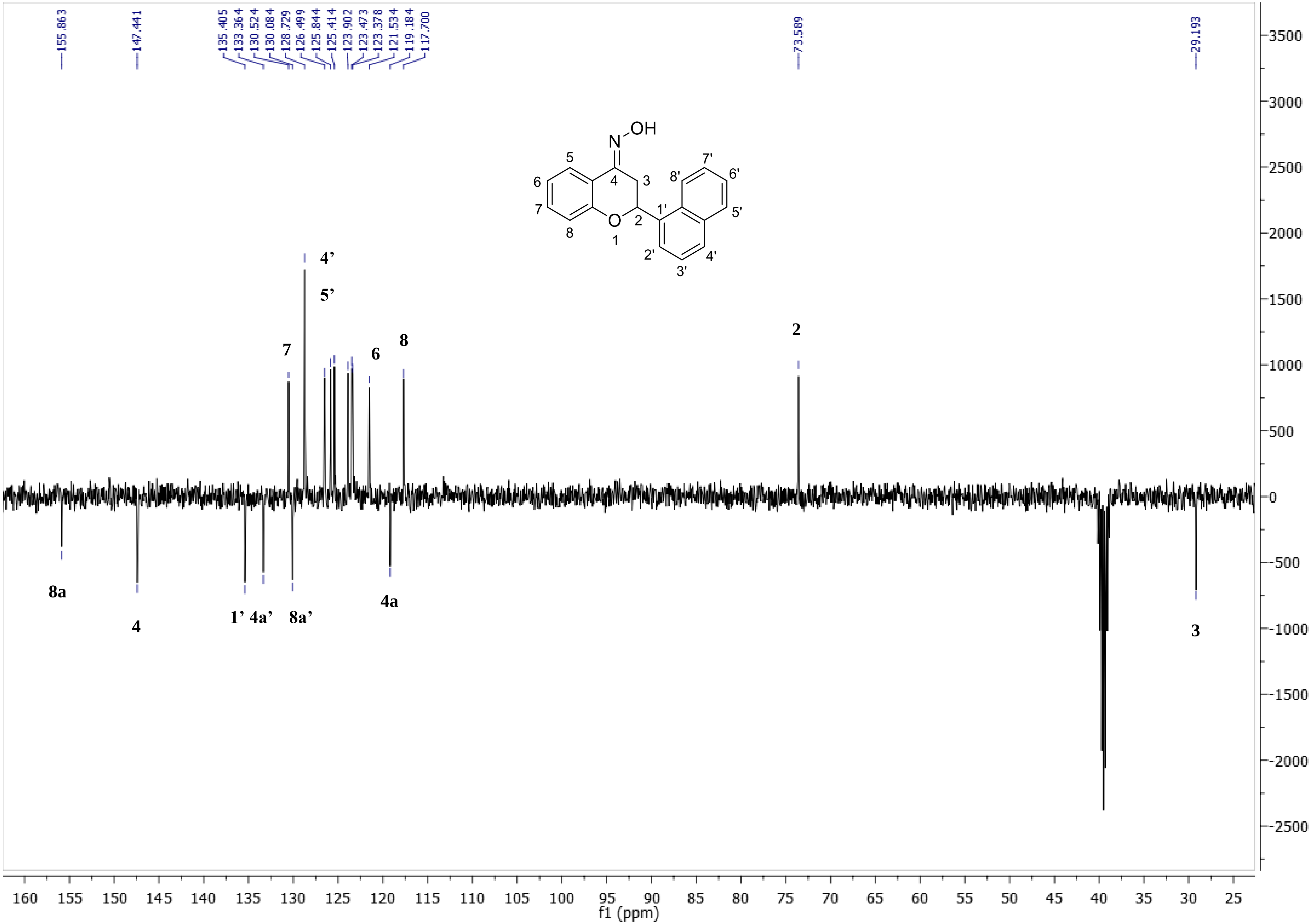

Figure S12. ^{13}C-NMR spectrum of **16f** in DMSO-d_6

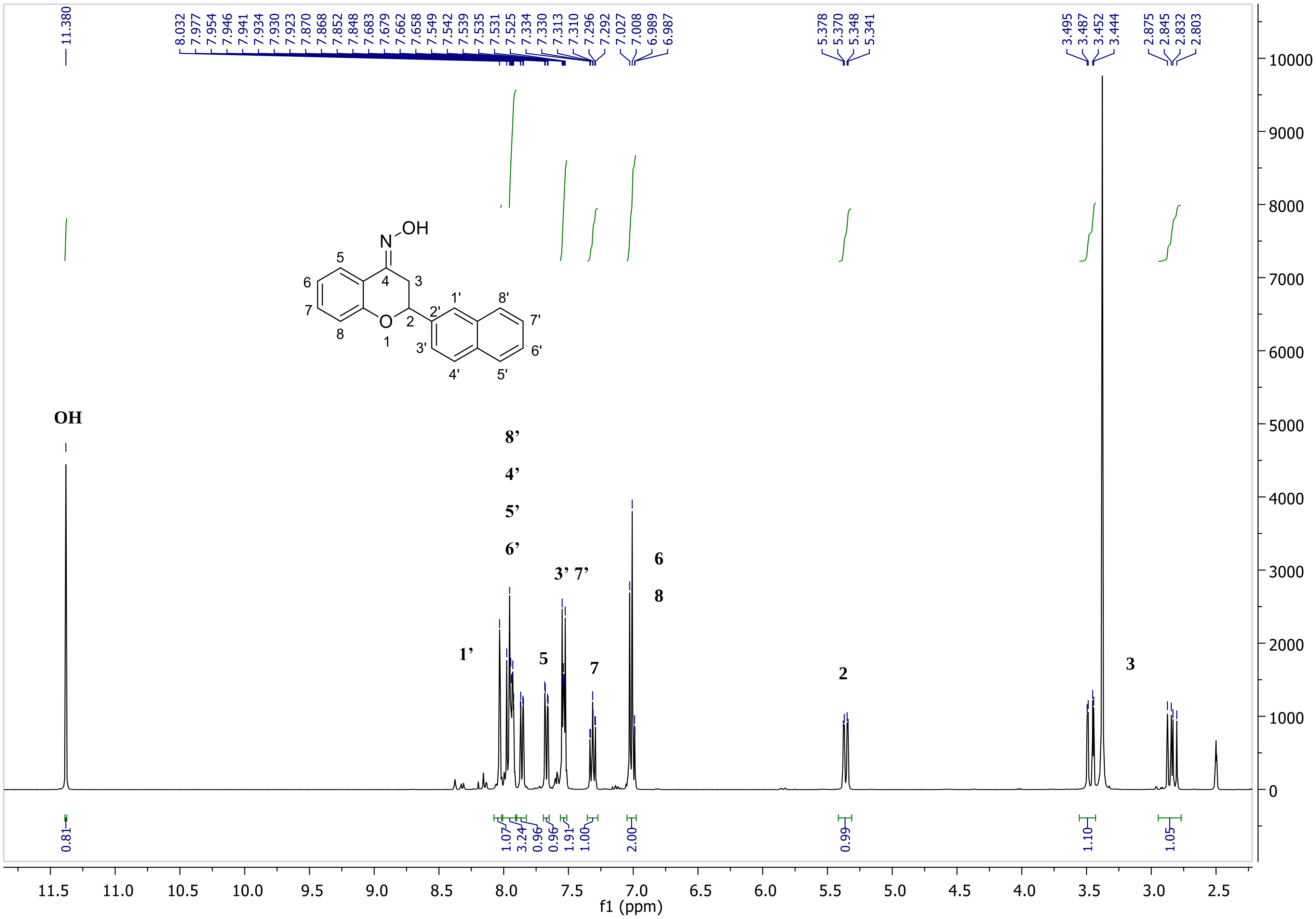

Figure S13. ^{1}H-NMR spectrum of **16g** in DMSO-d_6

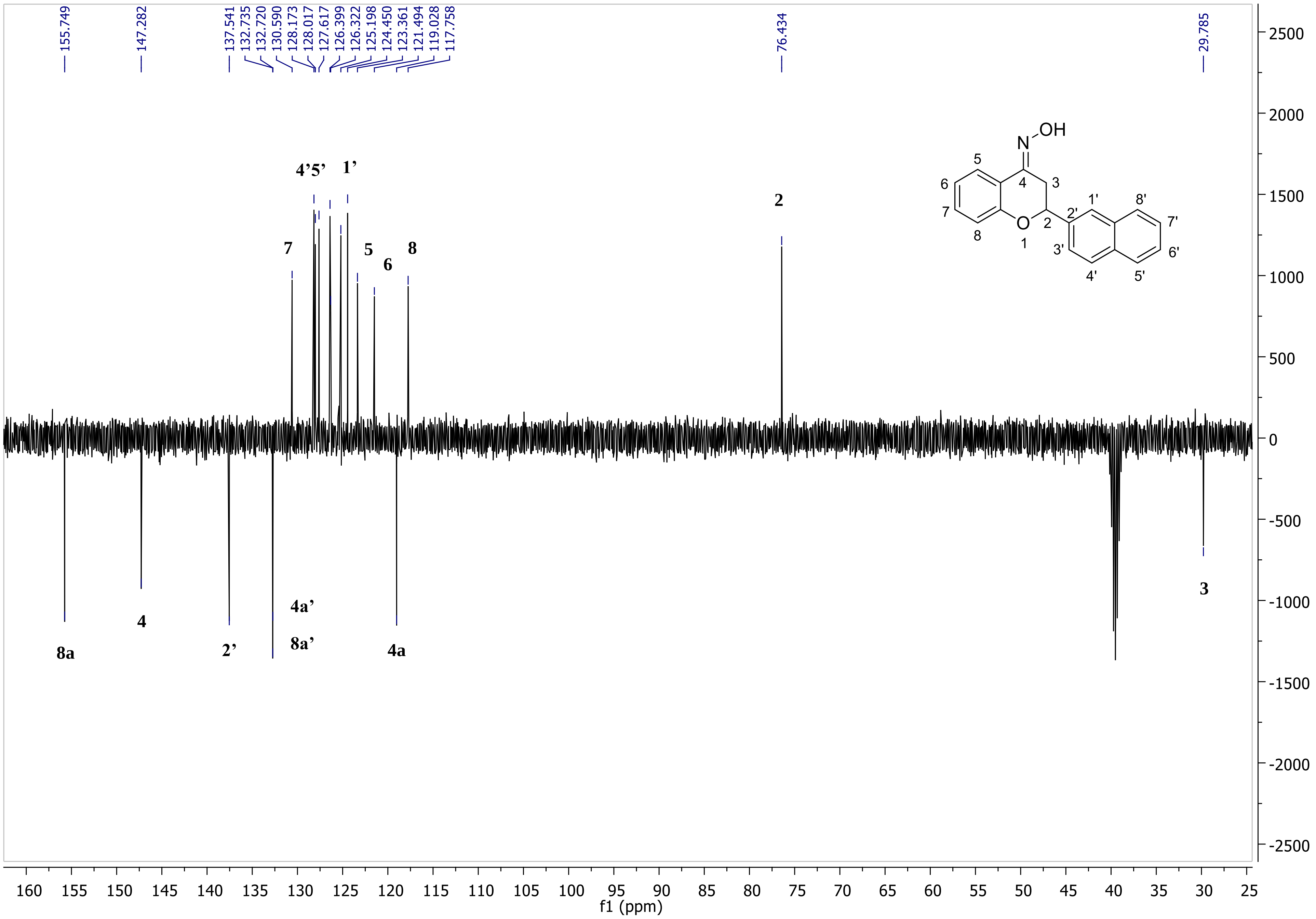

Figure S14. ^{13}C-NMR spectrum of **16g** in DMSO-d_6

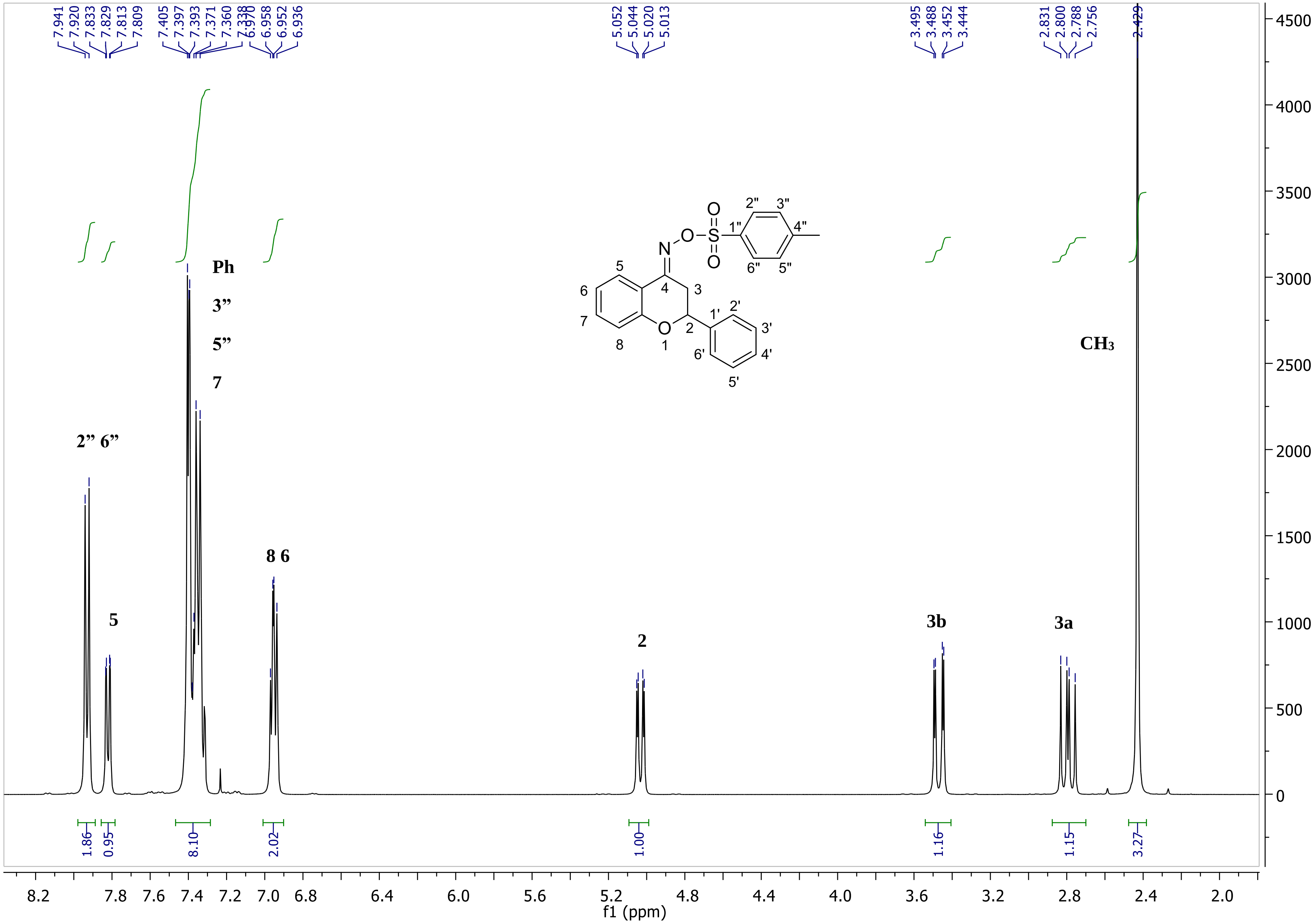

Figure S15. ^{1}H-NMR spectrum of **5a** in $CDCl_3$

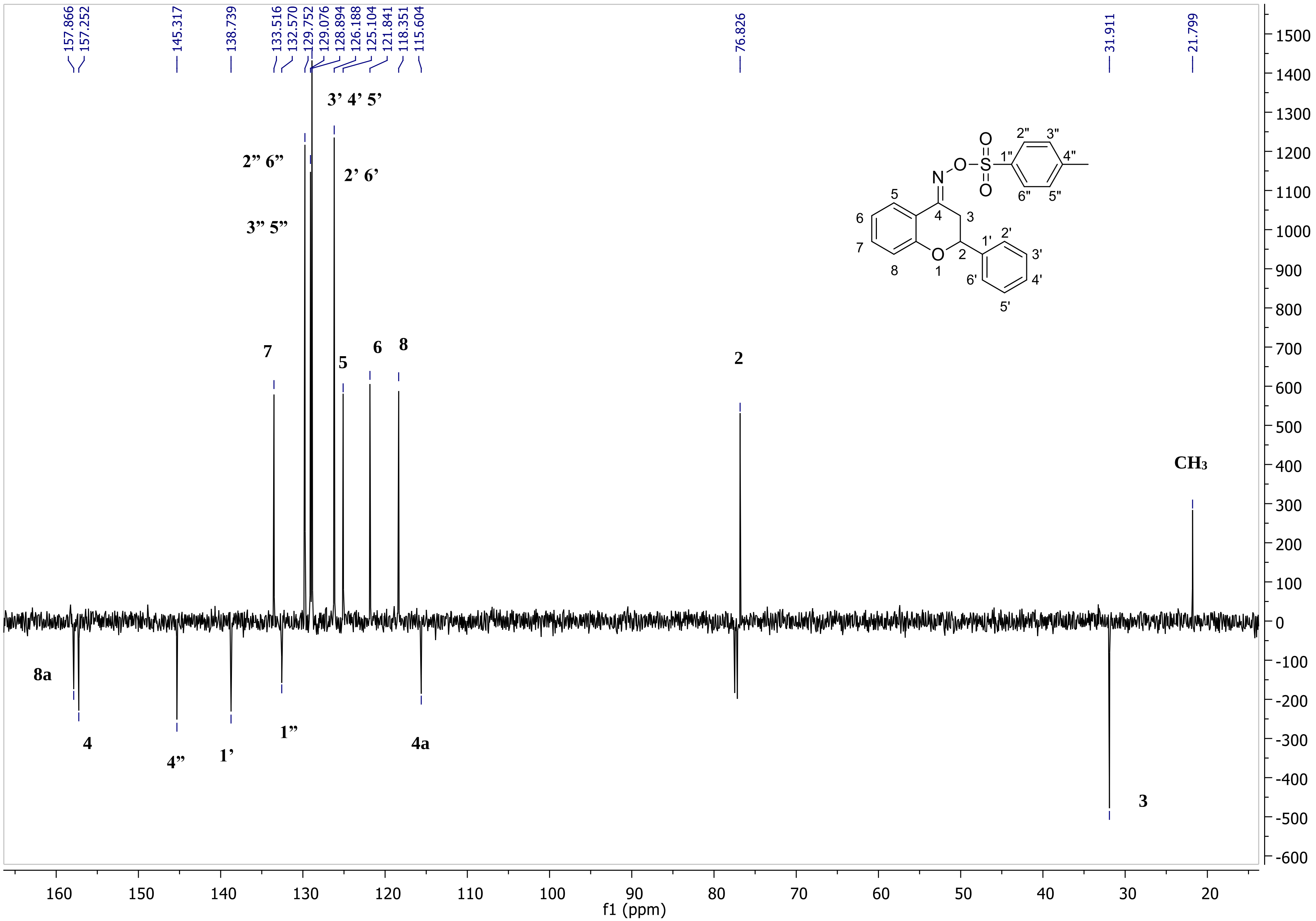

Figure S16. ^{13}C-NMR spectrum of **5a** in $CDCl_3$

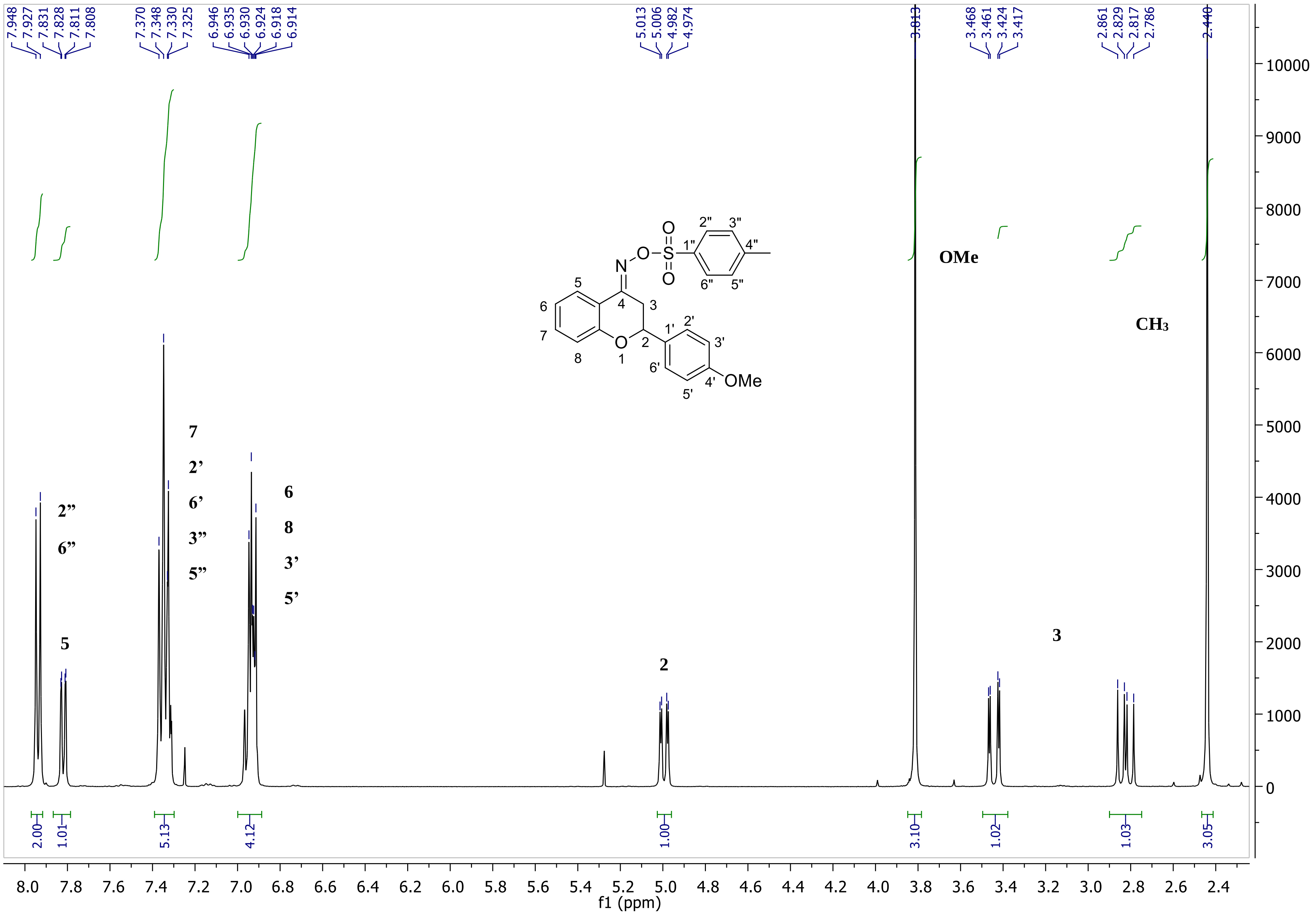

Figure S17. ^{1}H-NMR spectrum of **5b** in $CDCl_3$

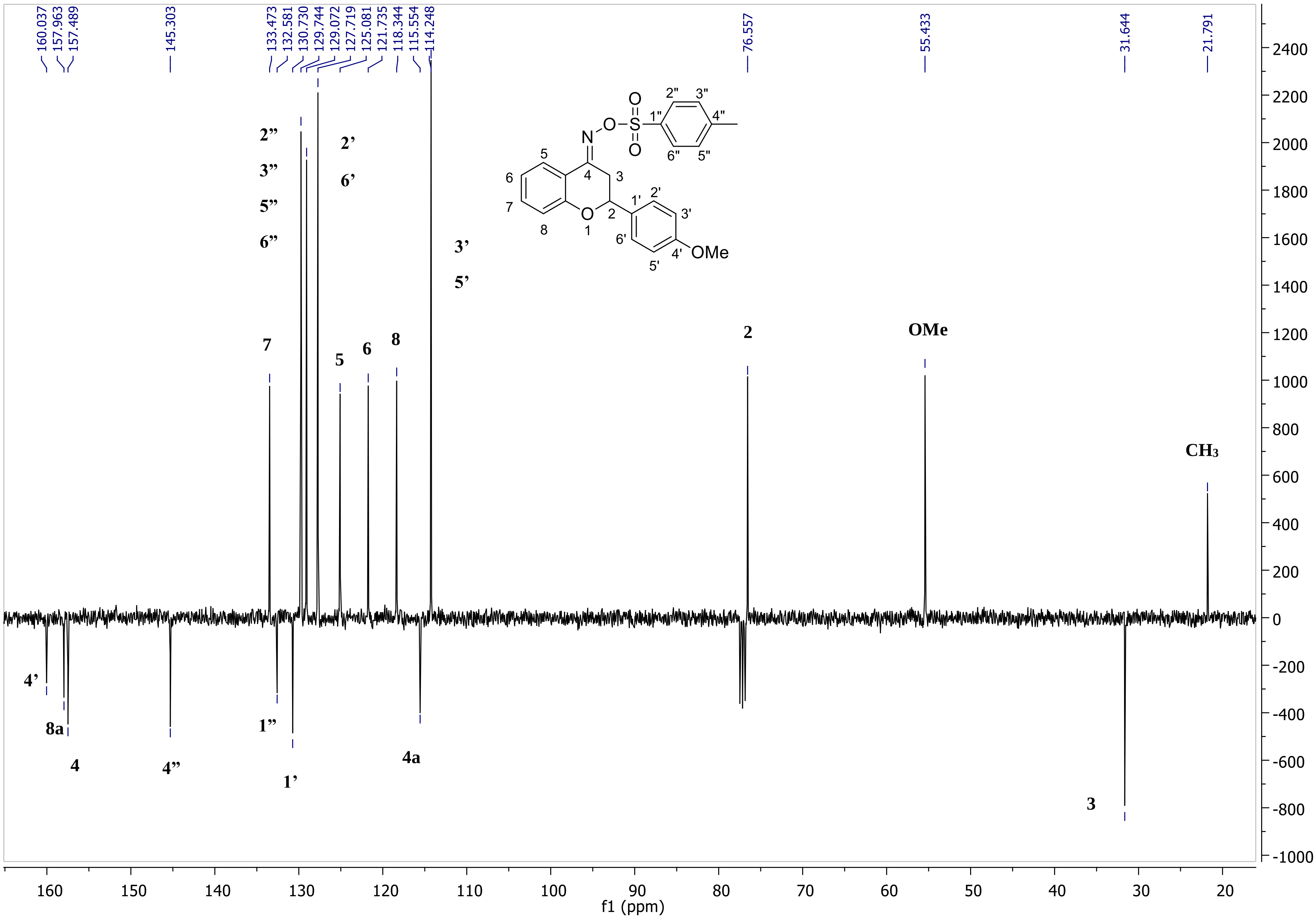

Figure 18. ^{13}C-NMR spectrum of **5b** in $CDCl_3$

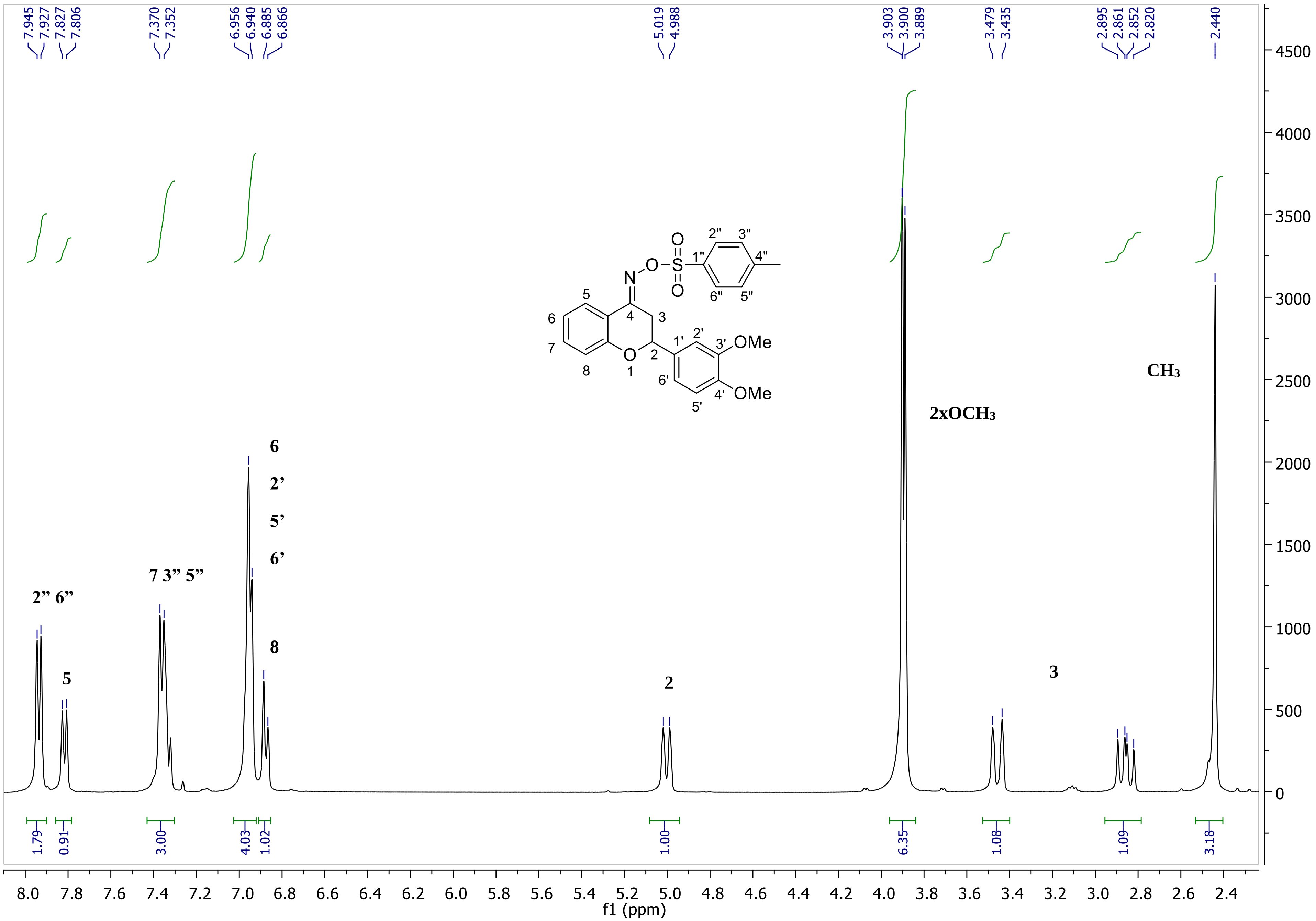

Figure S19. ^{1}H-NMR spectrum of **5c** in $CDCl_3$

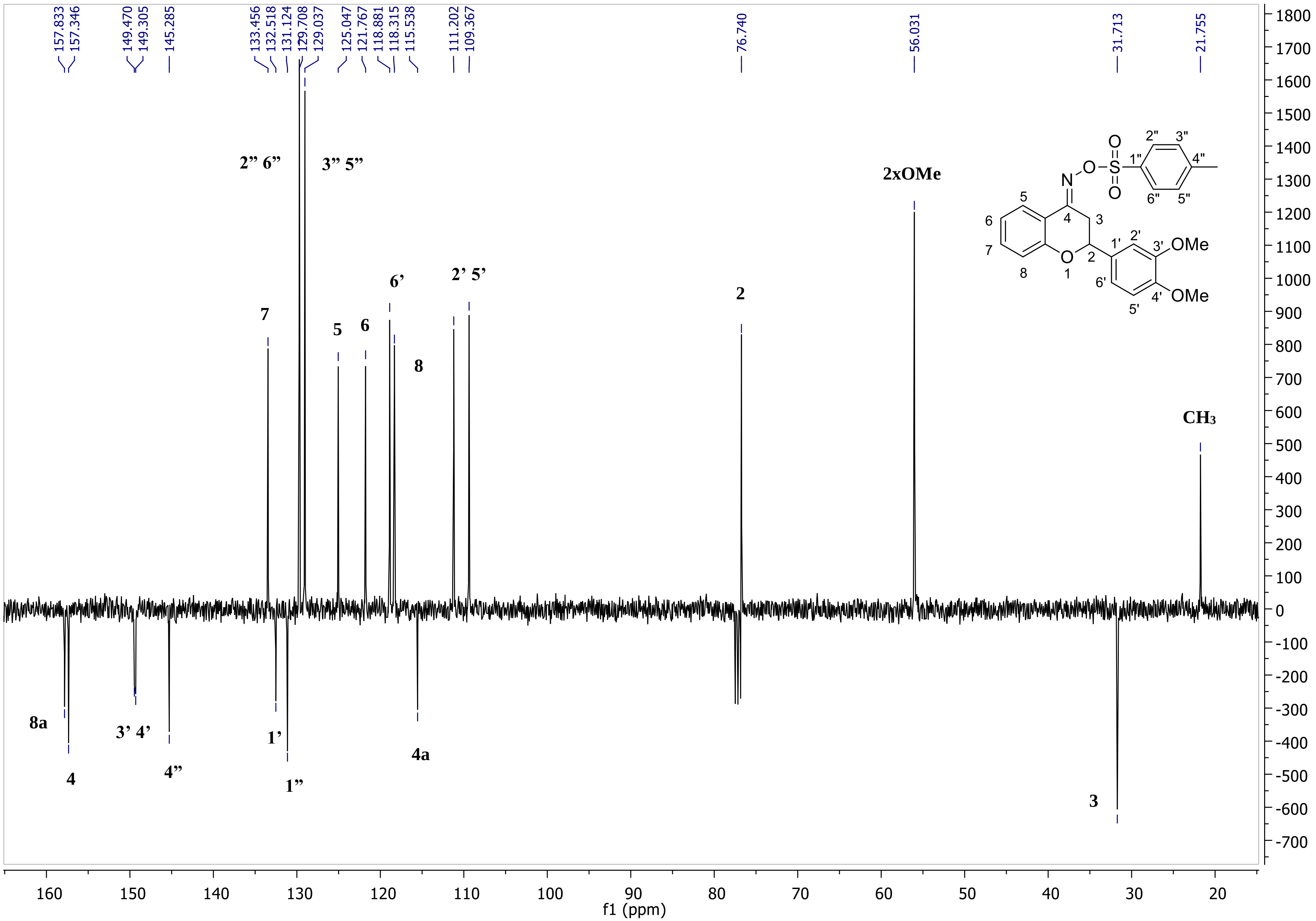

Figure 20. ^{13}C-NMR spectrum of **5c** in $CDCl_3$

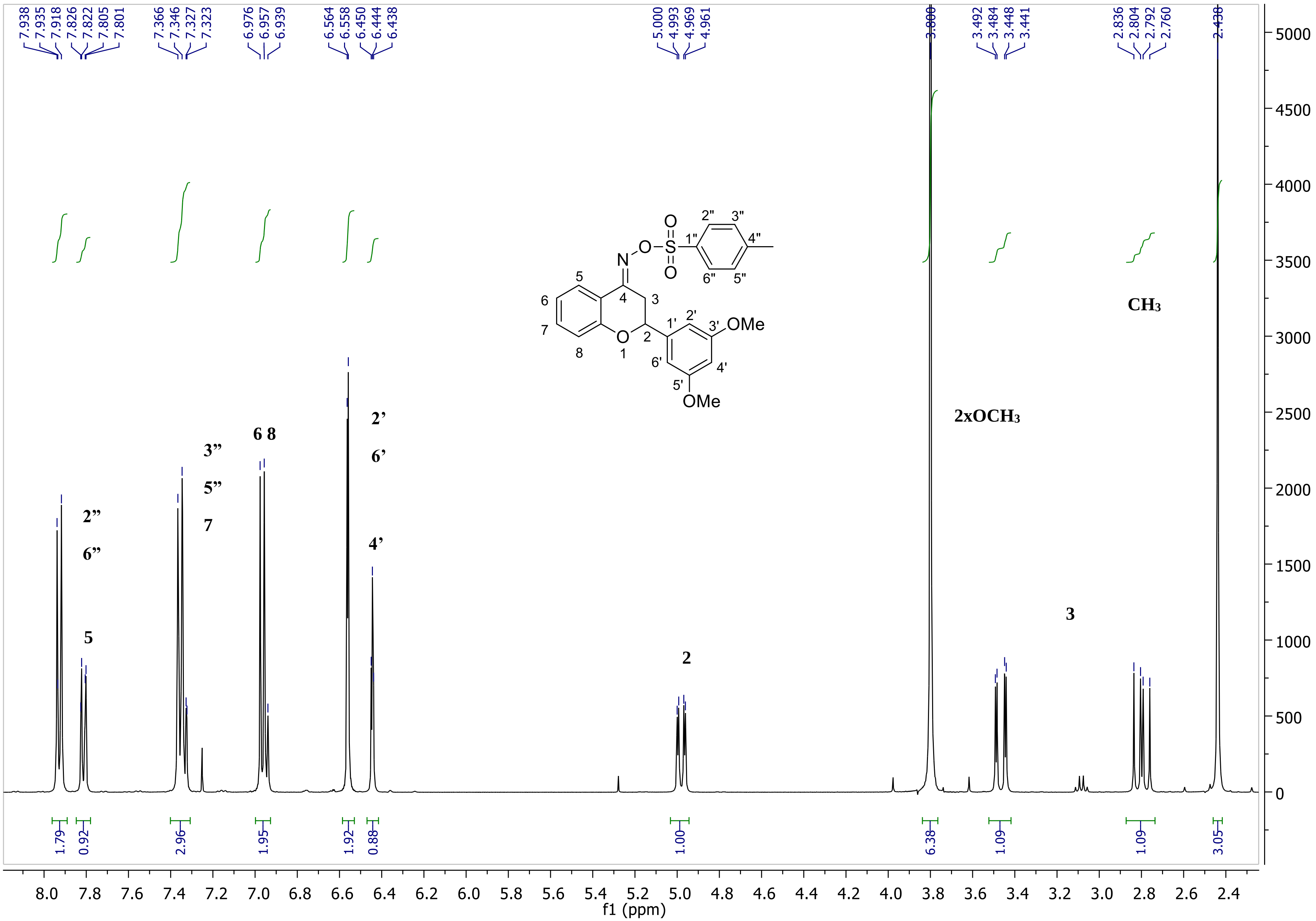

Figure 21. ^{1}H-NMR spectrum of **5d** in $CDCl_3$

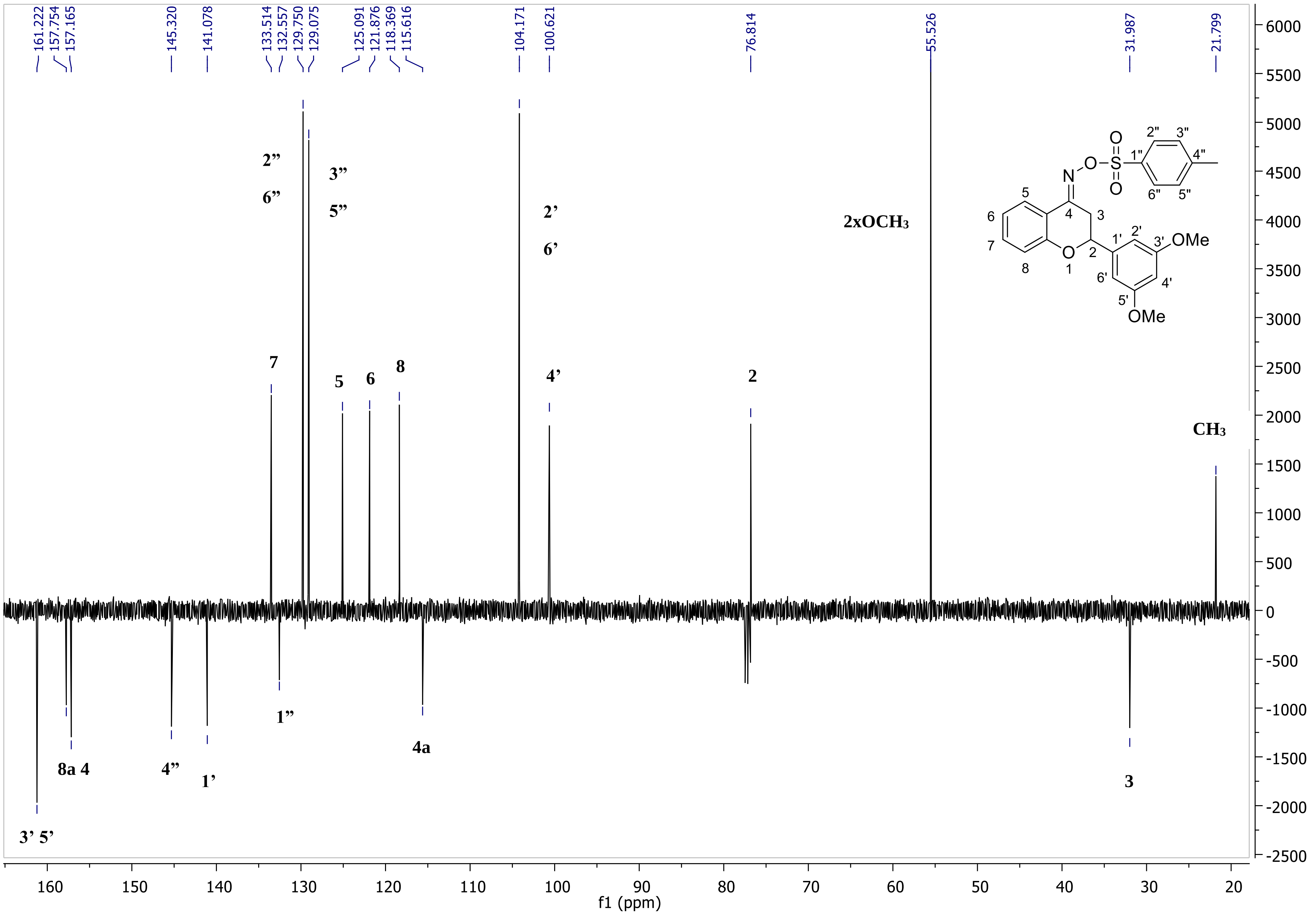

Figure S22. ^{13}C-NMR spectrum of **5d** in $CDCl_3$

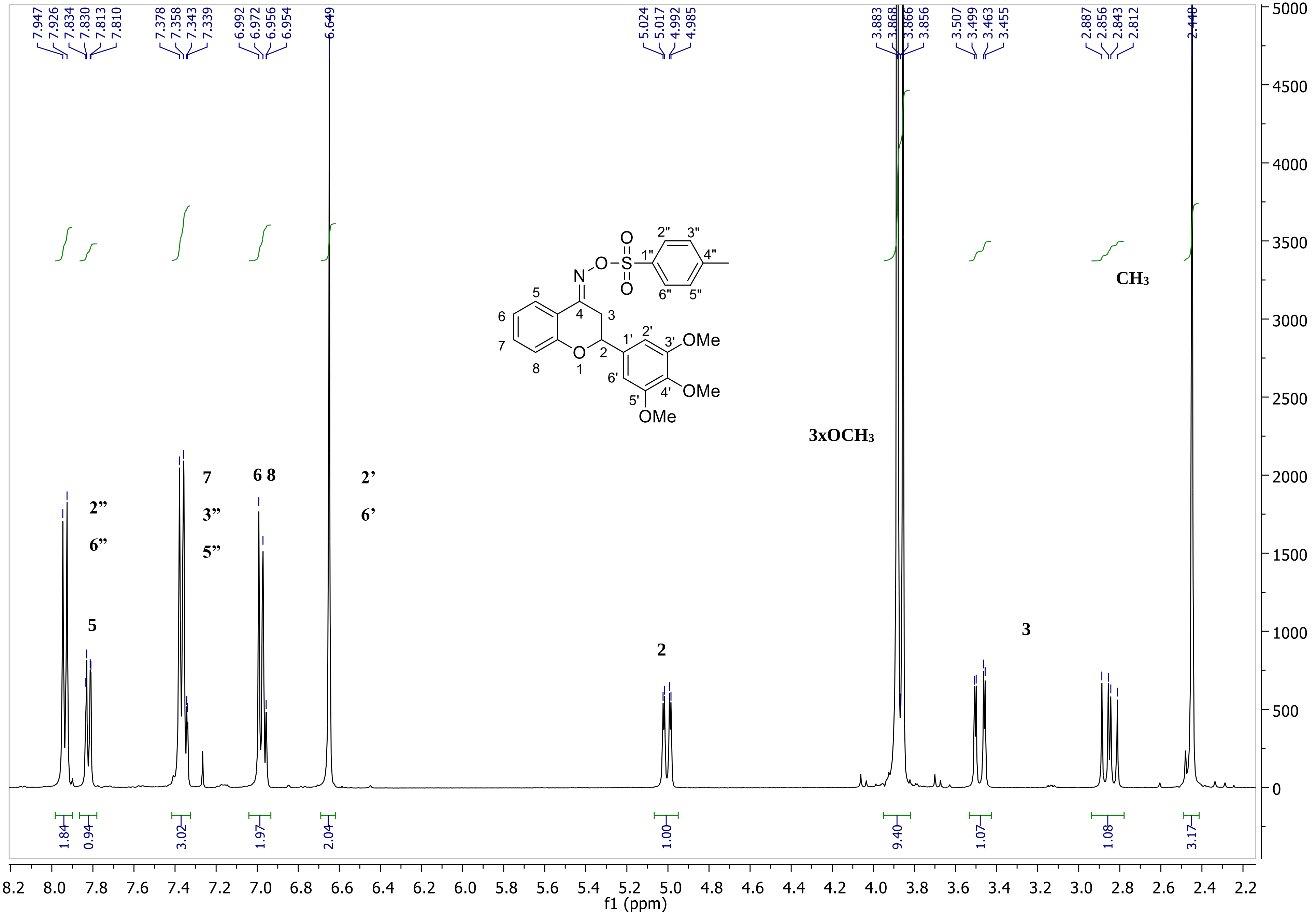

Figure S23. ^{1}H-NMR spectrum of **5e** in $CDCl_3$

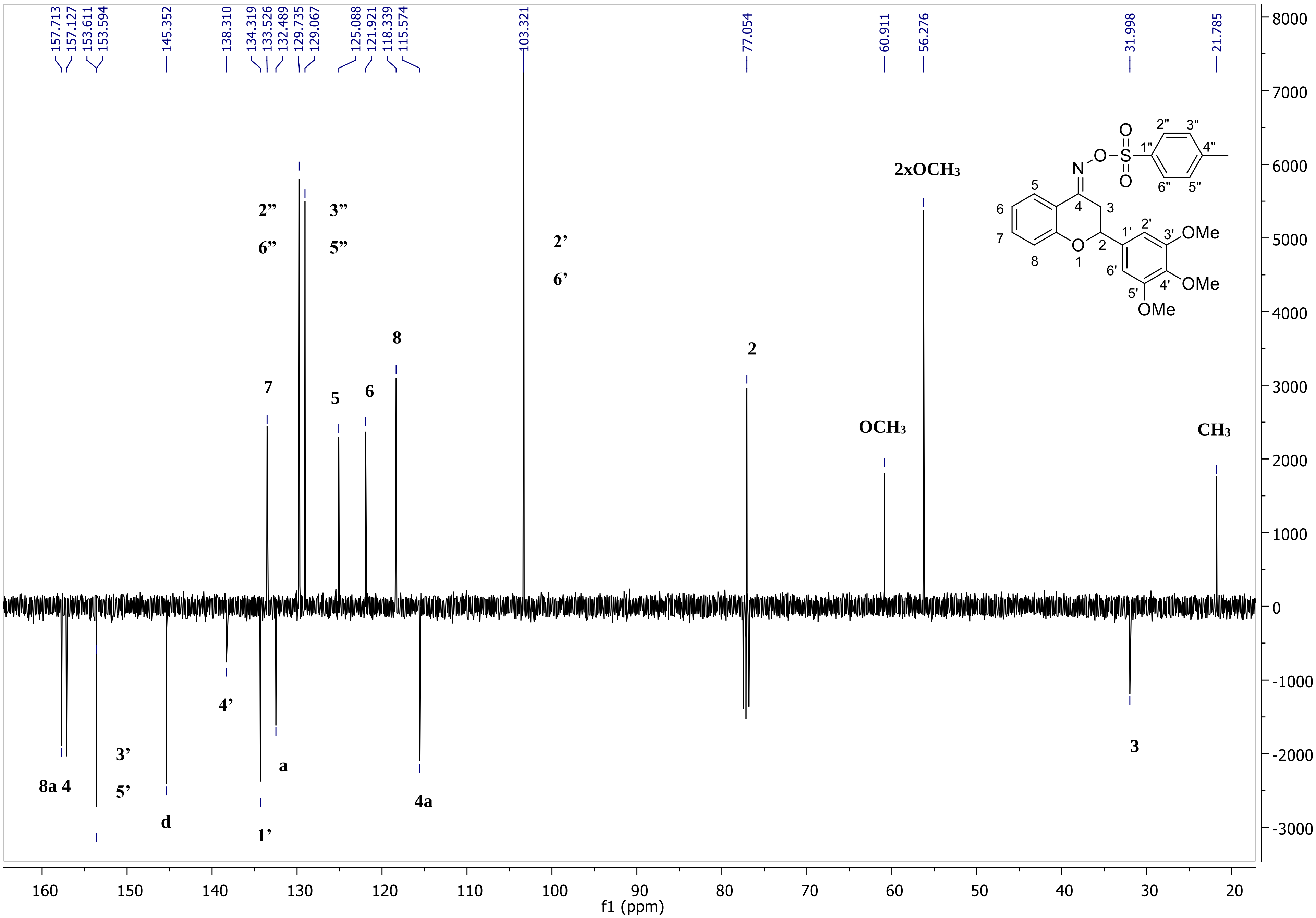

Figure S24. ^{13}C-NMR spectrum of **5e** in $CDCl_3$

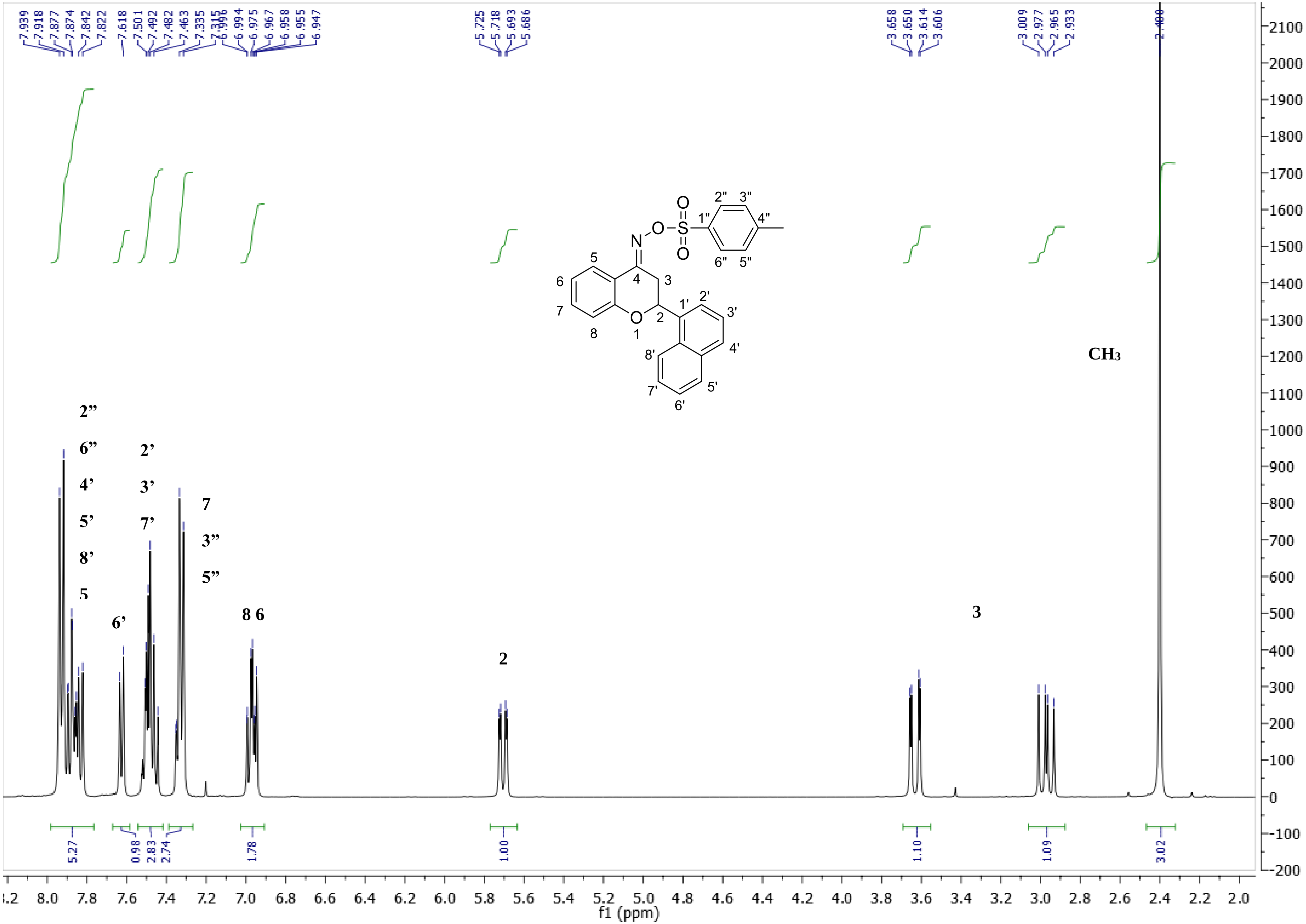

Figure S25. ^{1}H-NMR spectrum of **5f** in $CDCl_3$

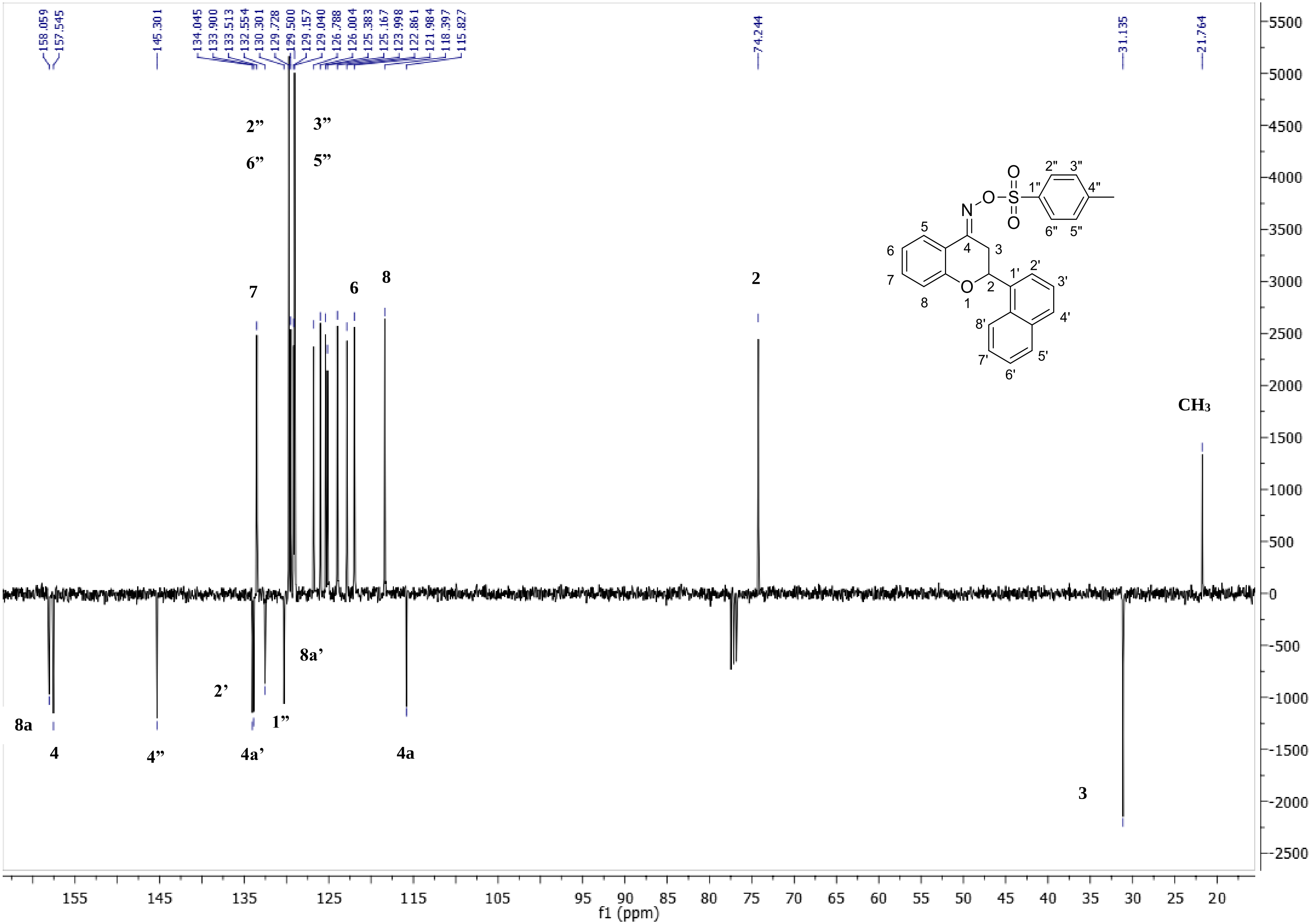

Figure S26. ^{13}C-NMR spectrum of **5f** in $CDCl_3$

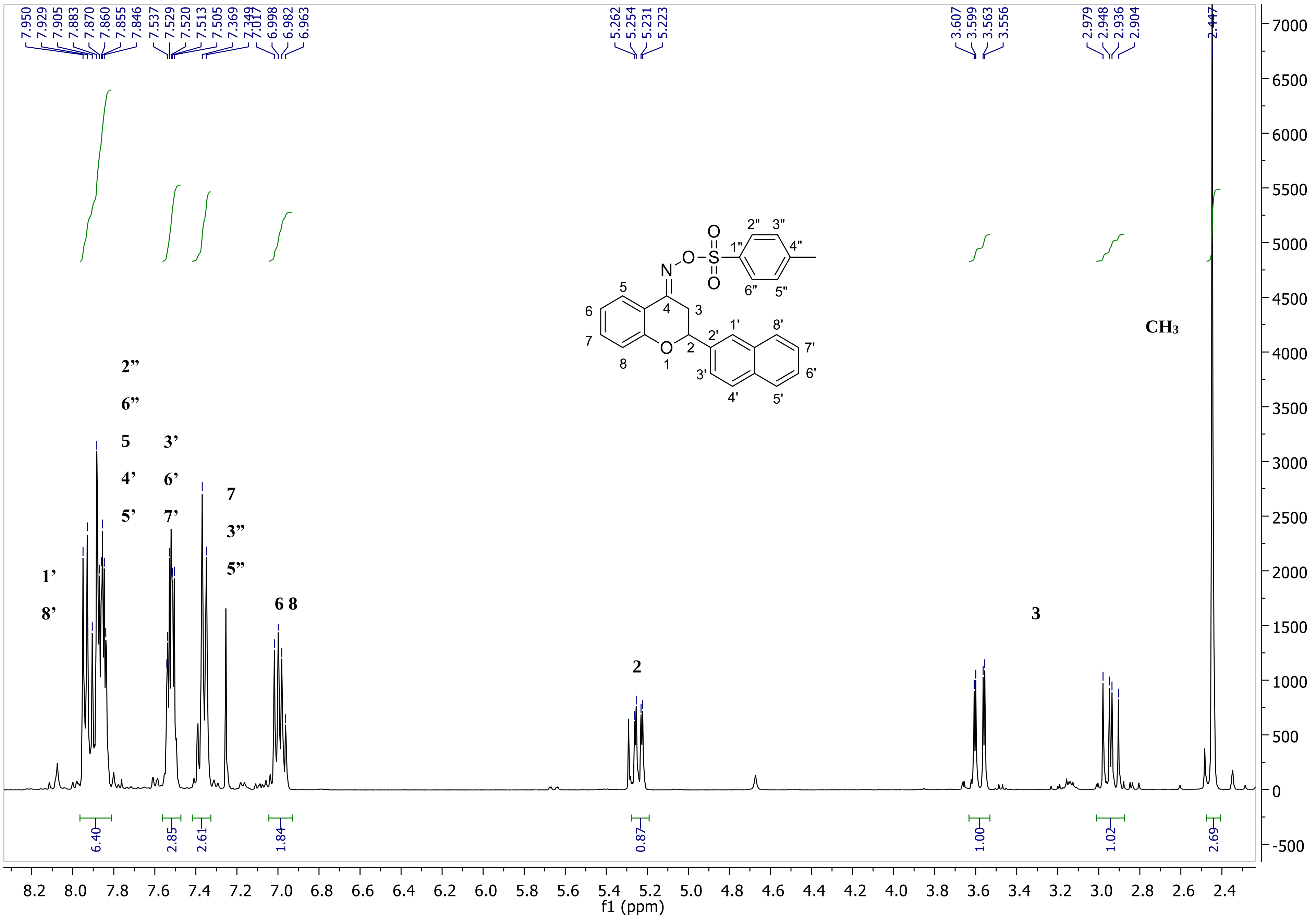

Figure S27. ^{1}H-NMR spectrum of **5g** in $CDCl_3$

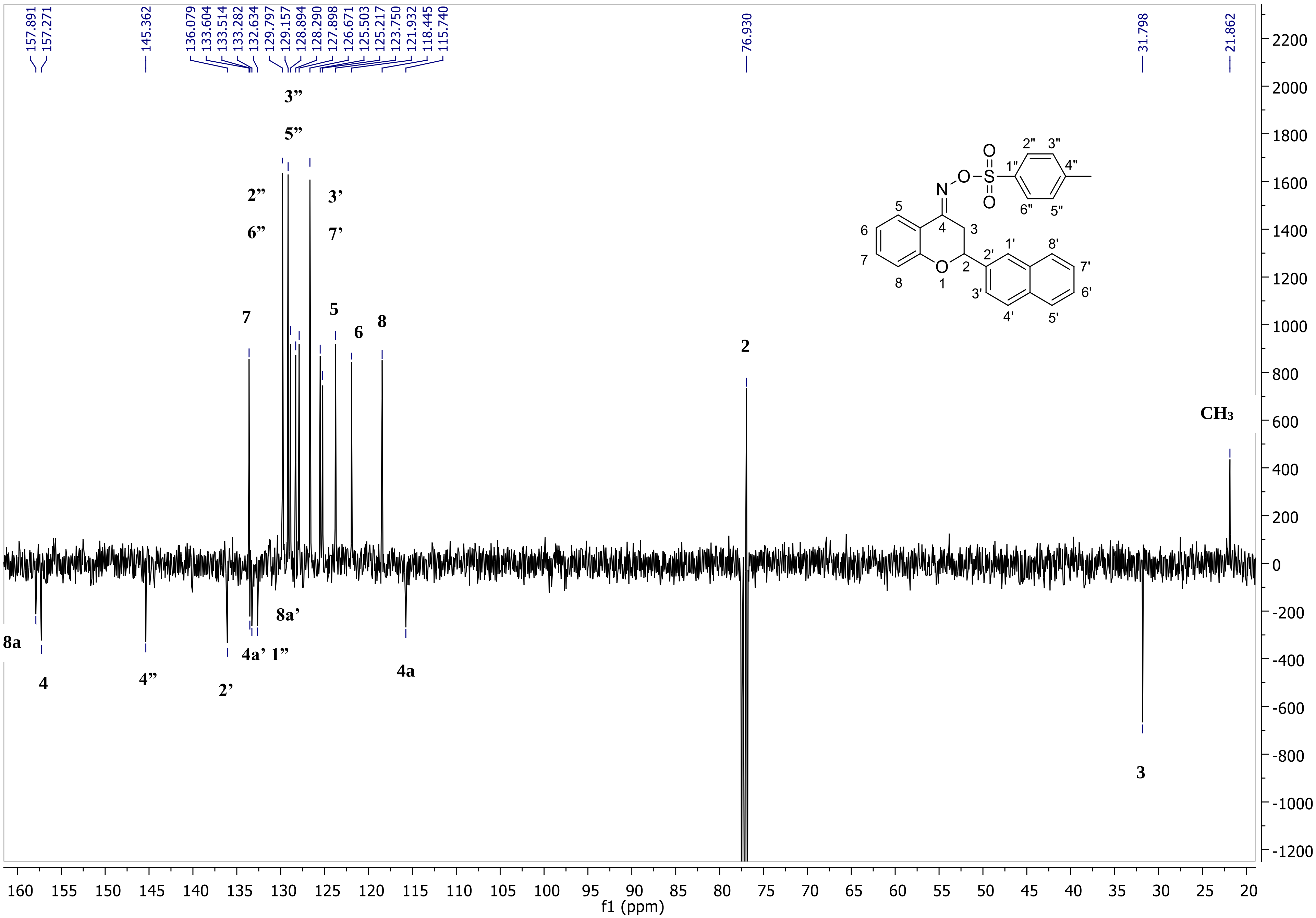

Figure S28. ^{13}C-NMR spectrum of **5g** in $CDCl_3$

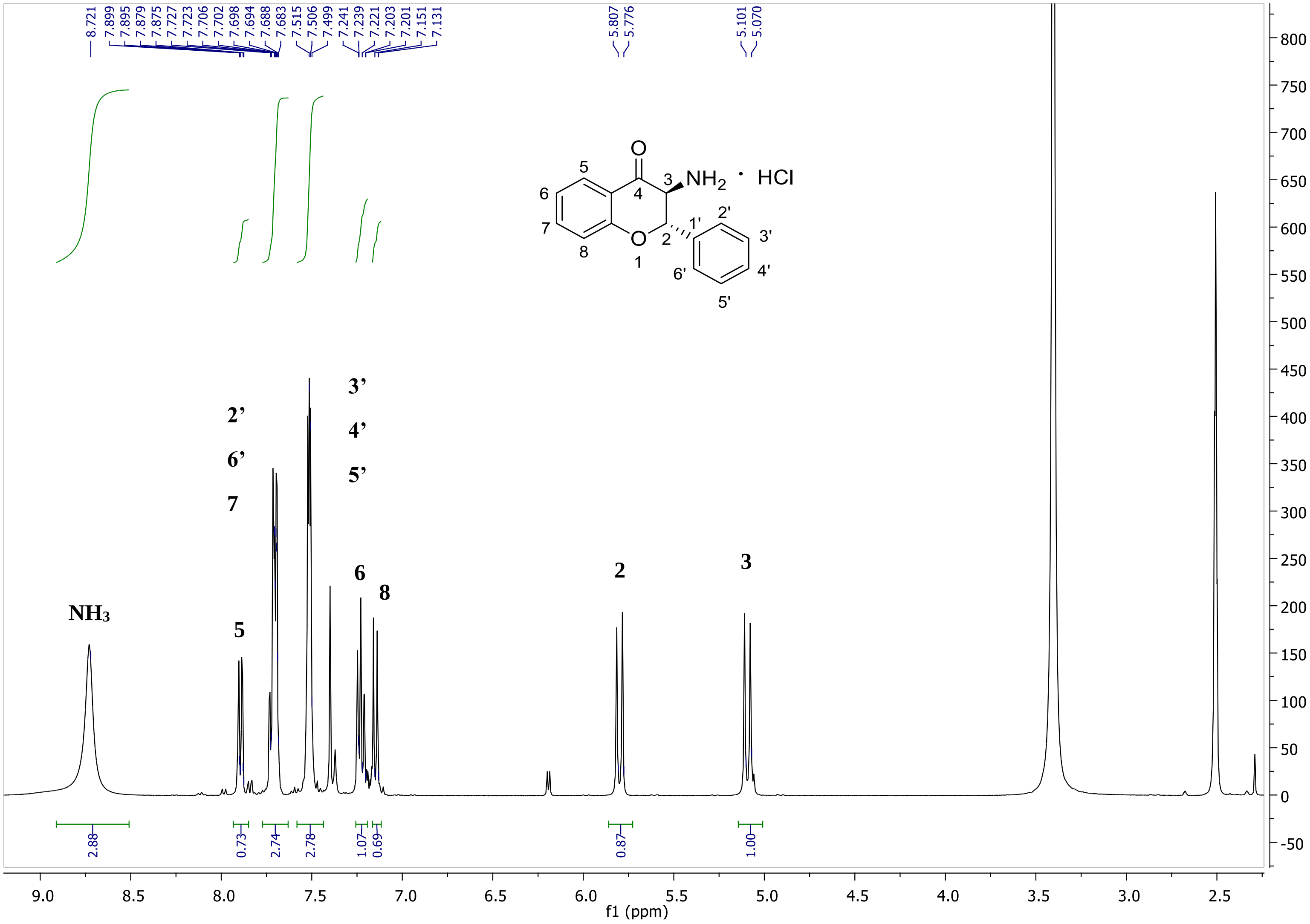

Figure S29. ^{1}H-NMR spectrum of *rac-trans*-**1a** in DMSO-d$_6$

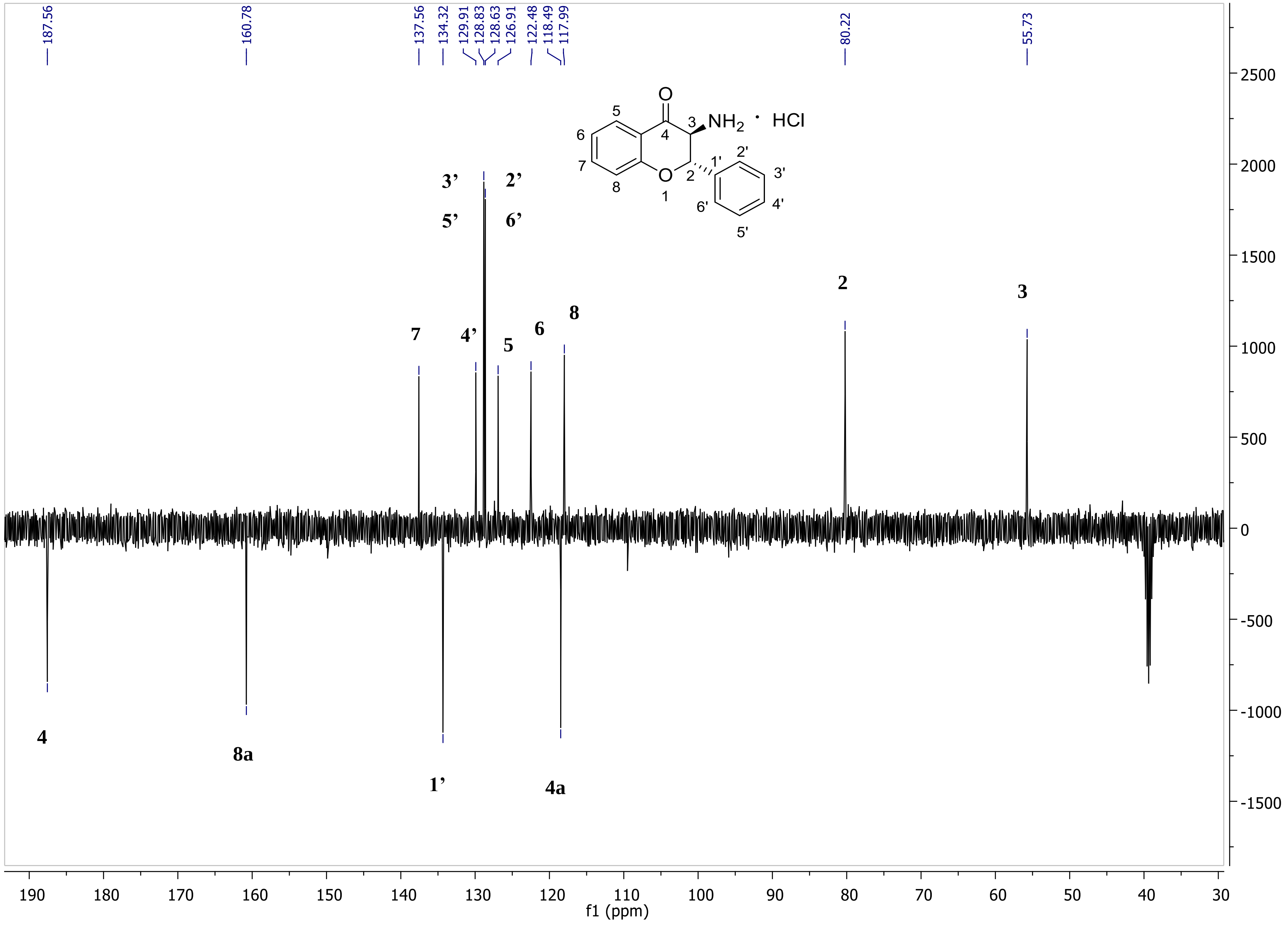

Figure S30. ^{13}C-NMR spectrum of *rac*-*trans*-**1a** in DMSO-d$_6$

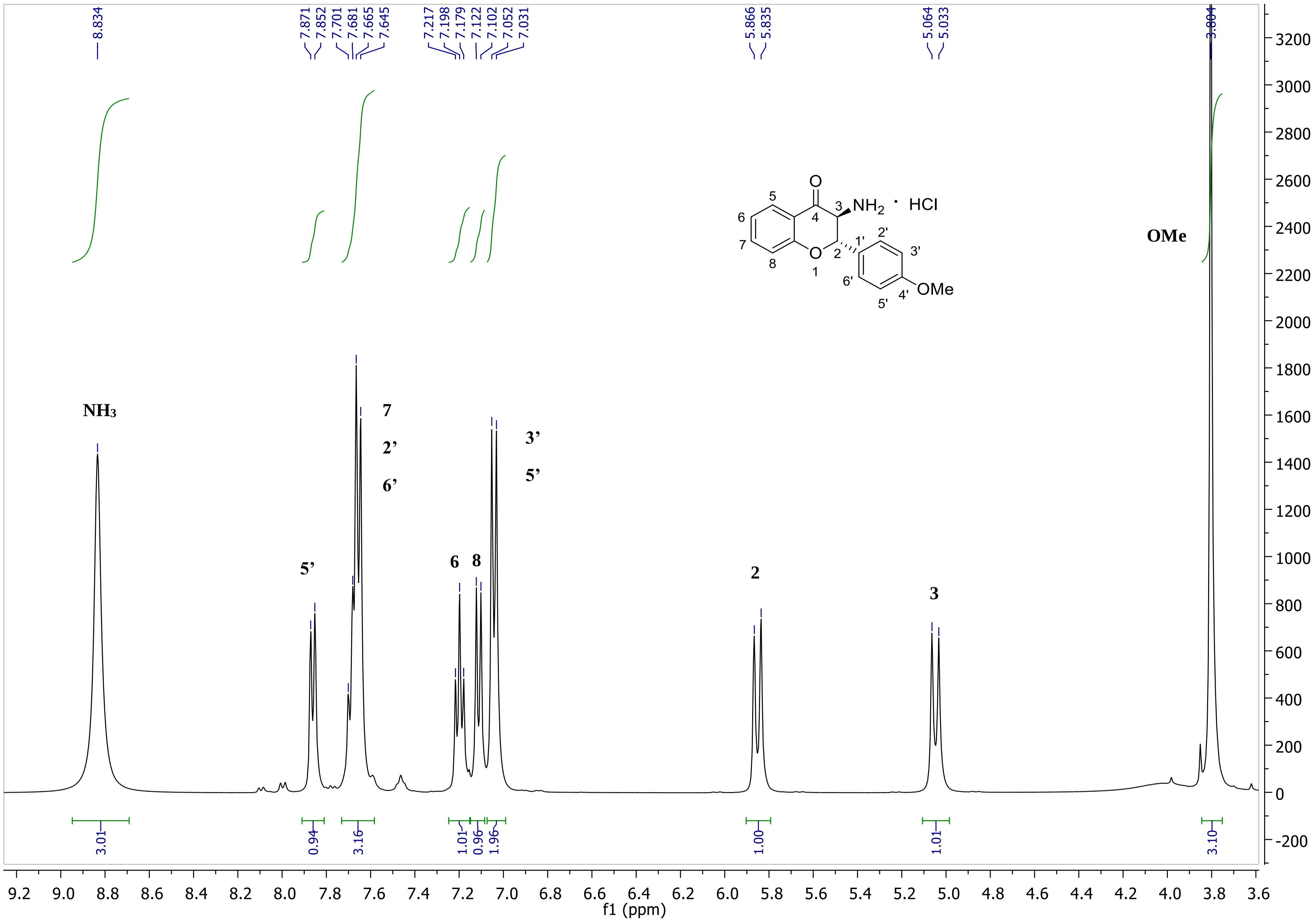

Figure S31. ^{1}H-NMR spectrum of *rac-trans*-**1b** in DMSO-d$_6$

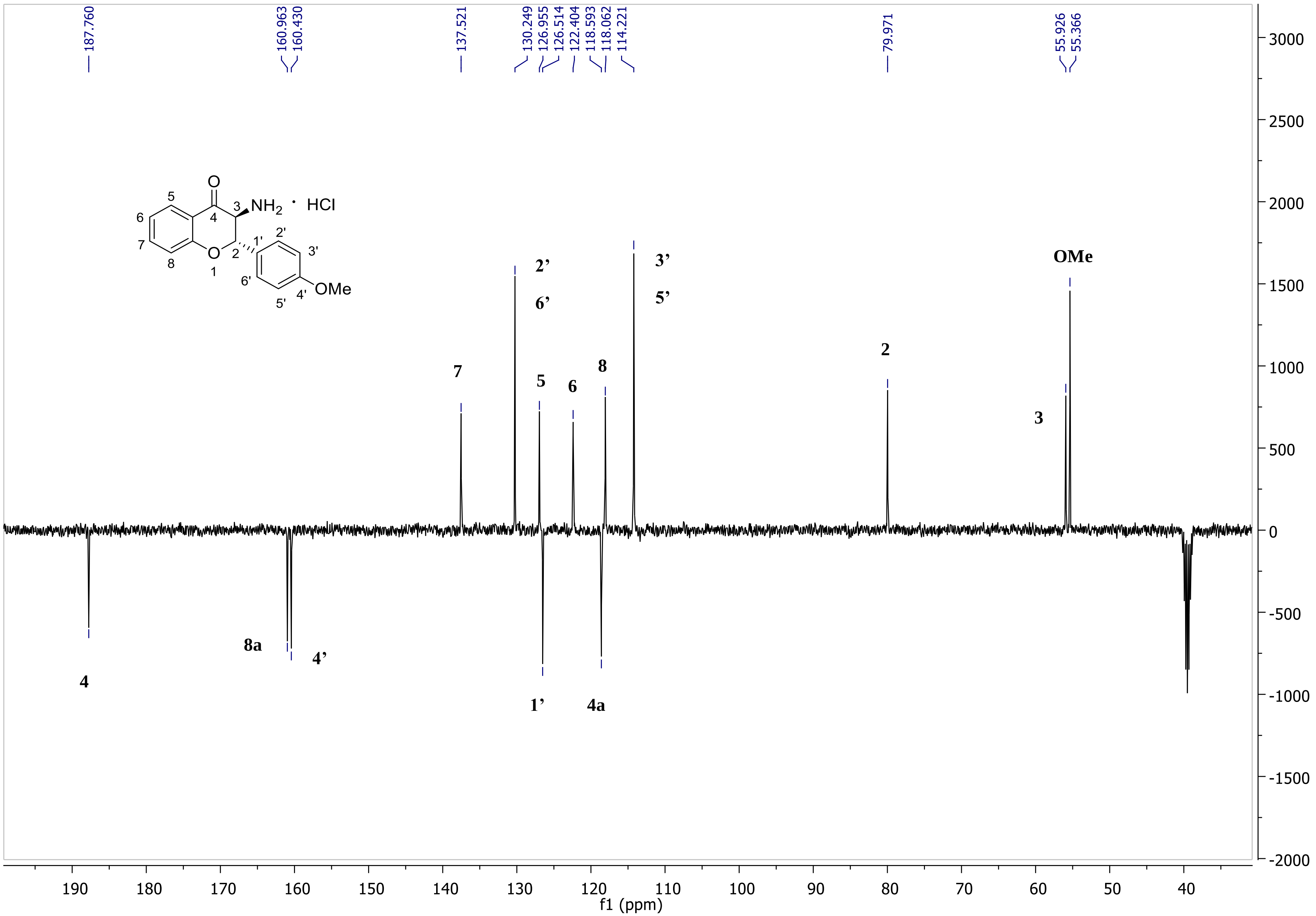

Figure S32. ^{13}C-NMR spectrum of *rac-trans*-**1b** in DMSO-d_6

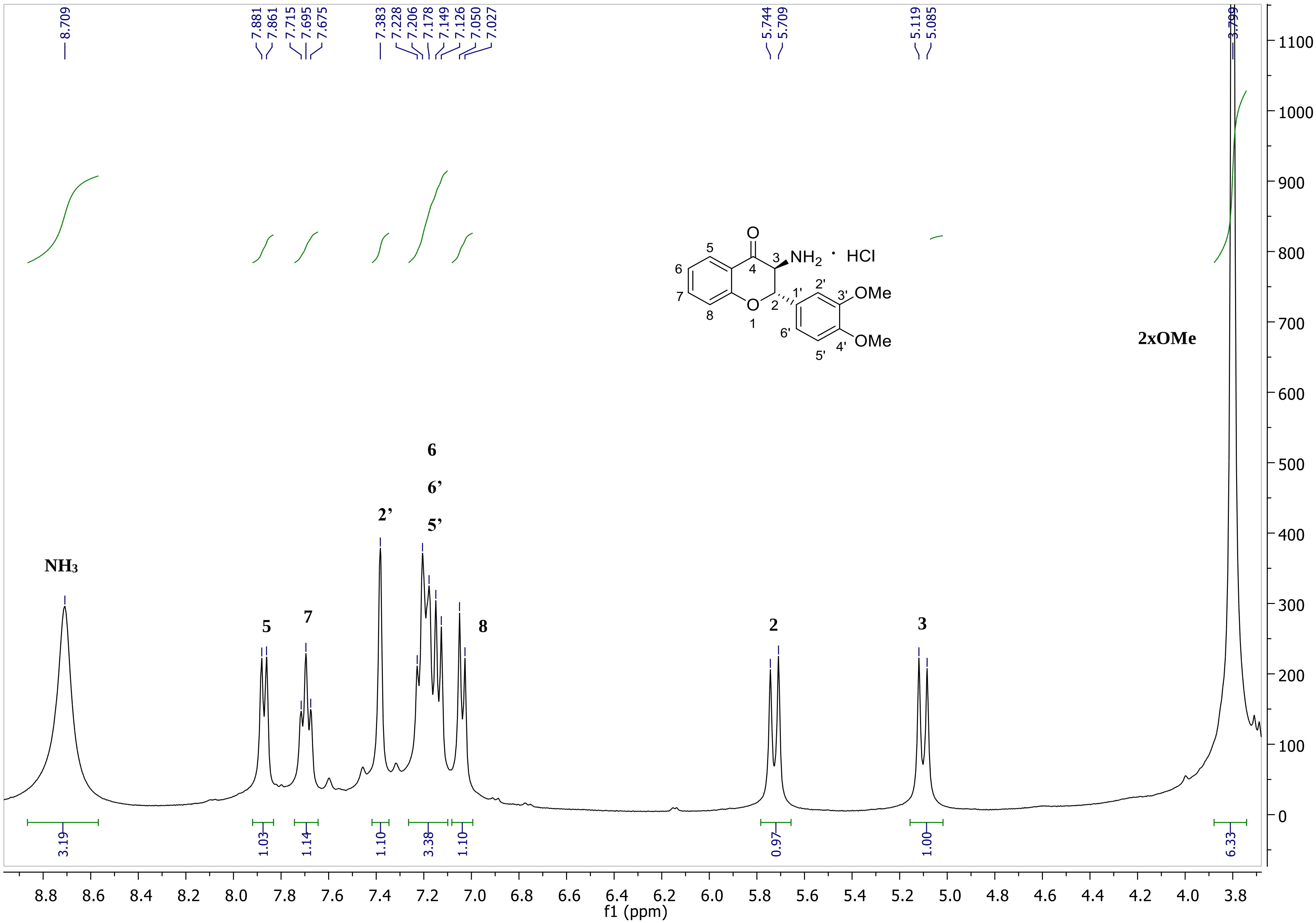

Figure S33. ^{1}H-NMR spectrum of *rac*-*trans*-**1c** in DMSO-d$_6$

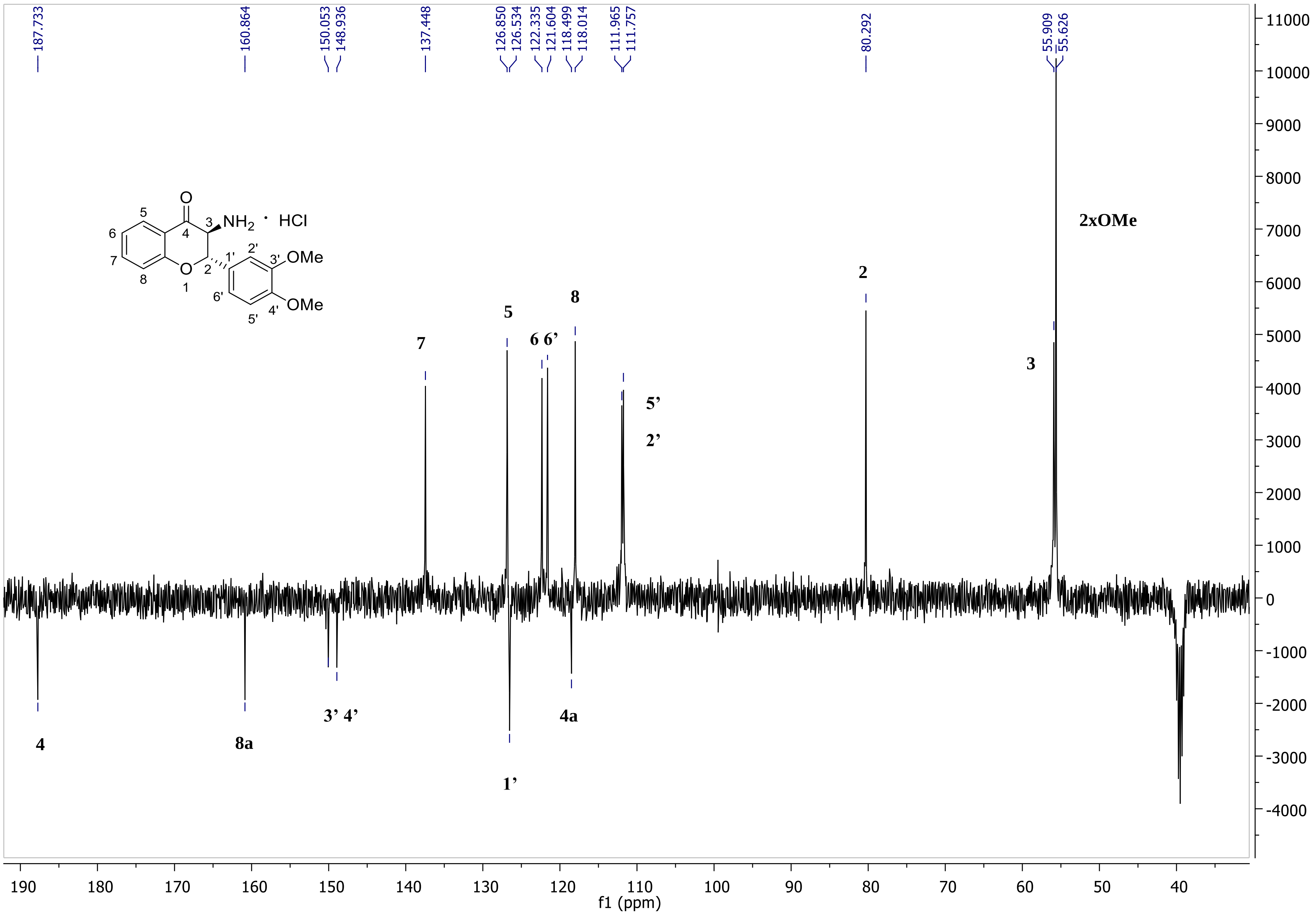

Figure S34. ^{13}C-NMR spectrum of *rac-trans*-**1c** in DMSO-d$_6$

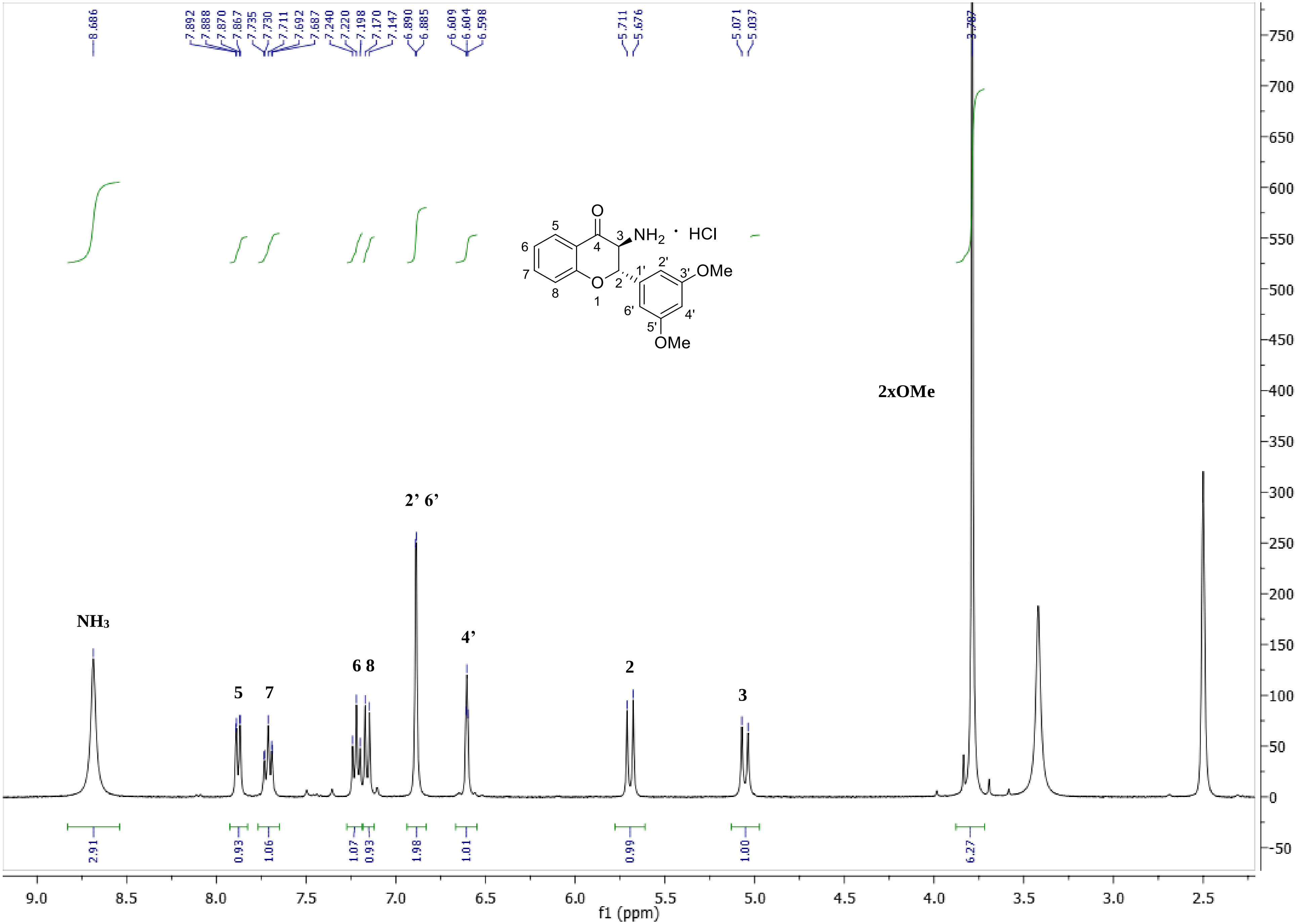

Figure S35. ^{1}H-NMR spectrum of *rac-trans*-**1d** in DMSO-d$_6$

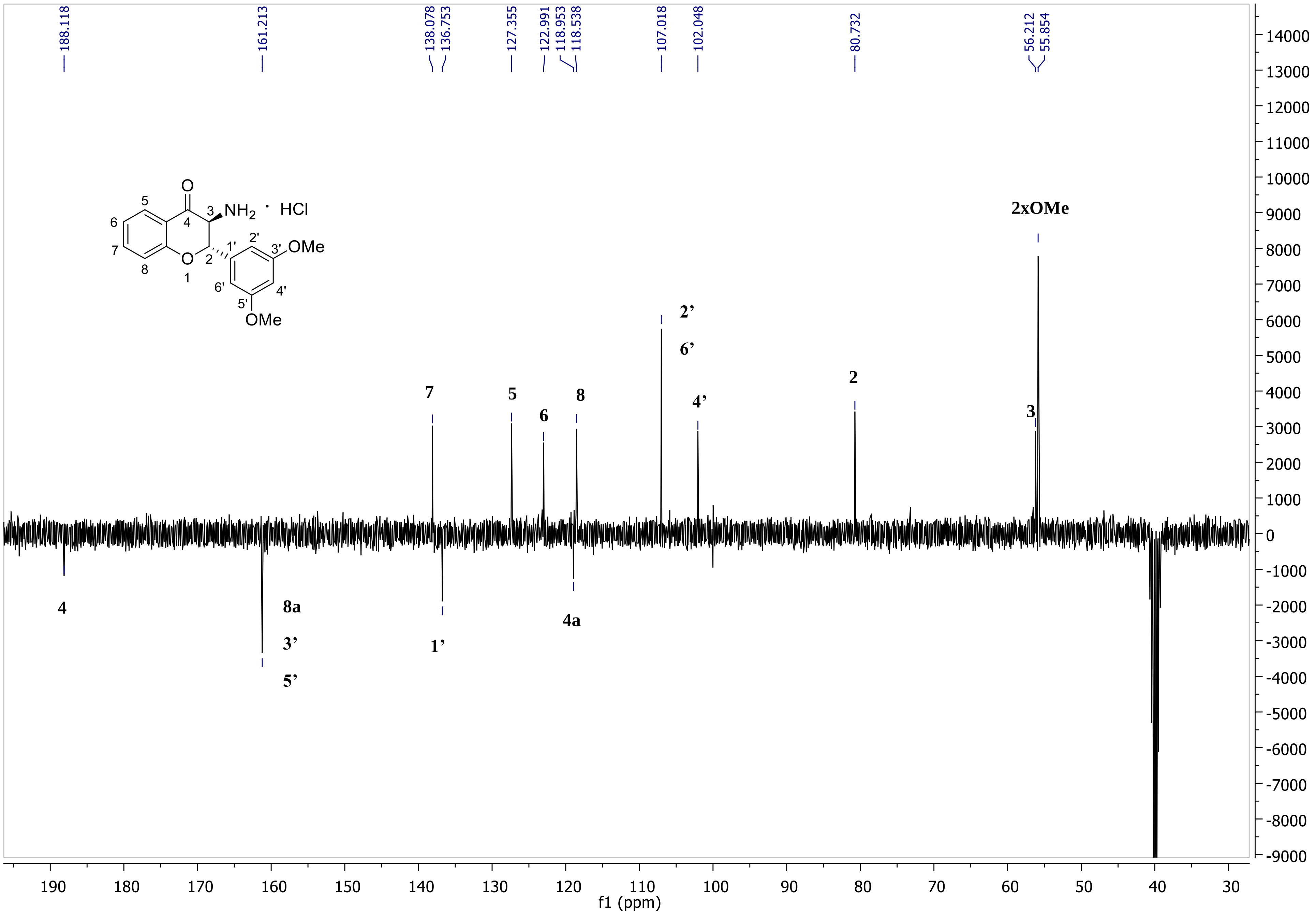

Figure S36. ^{13}C-NMR spectrum of *rac-trans*-**1d** in DMSO-d_6

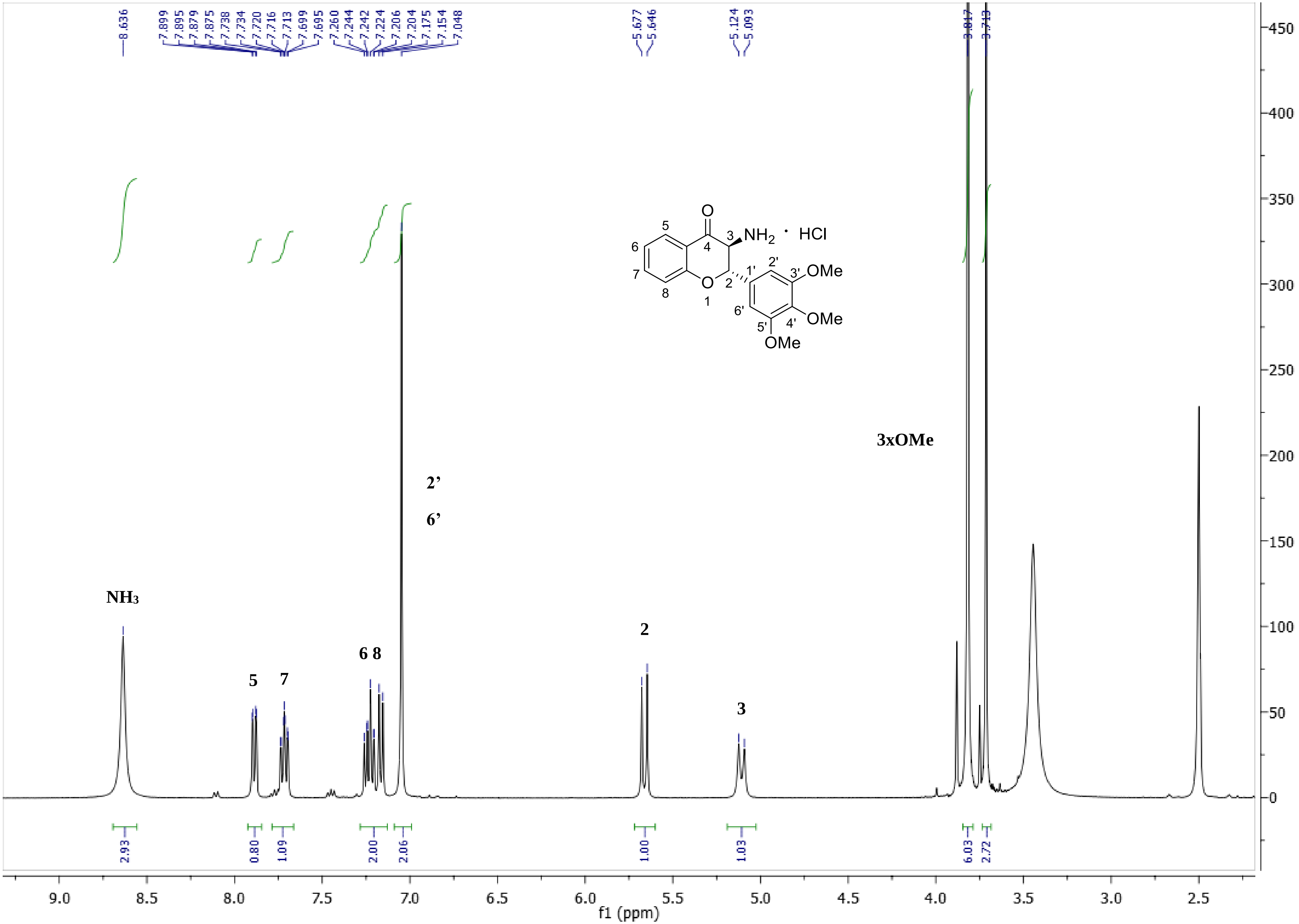

Figure S37. ^{1}H-NMR spectrum of *rac-trans*-**1e** in DMSO-d$_6$

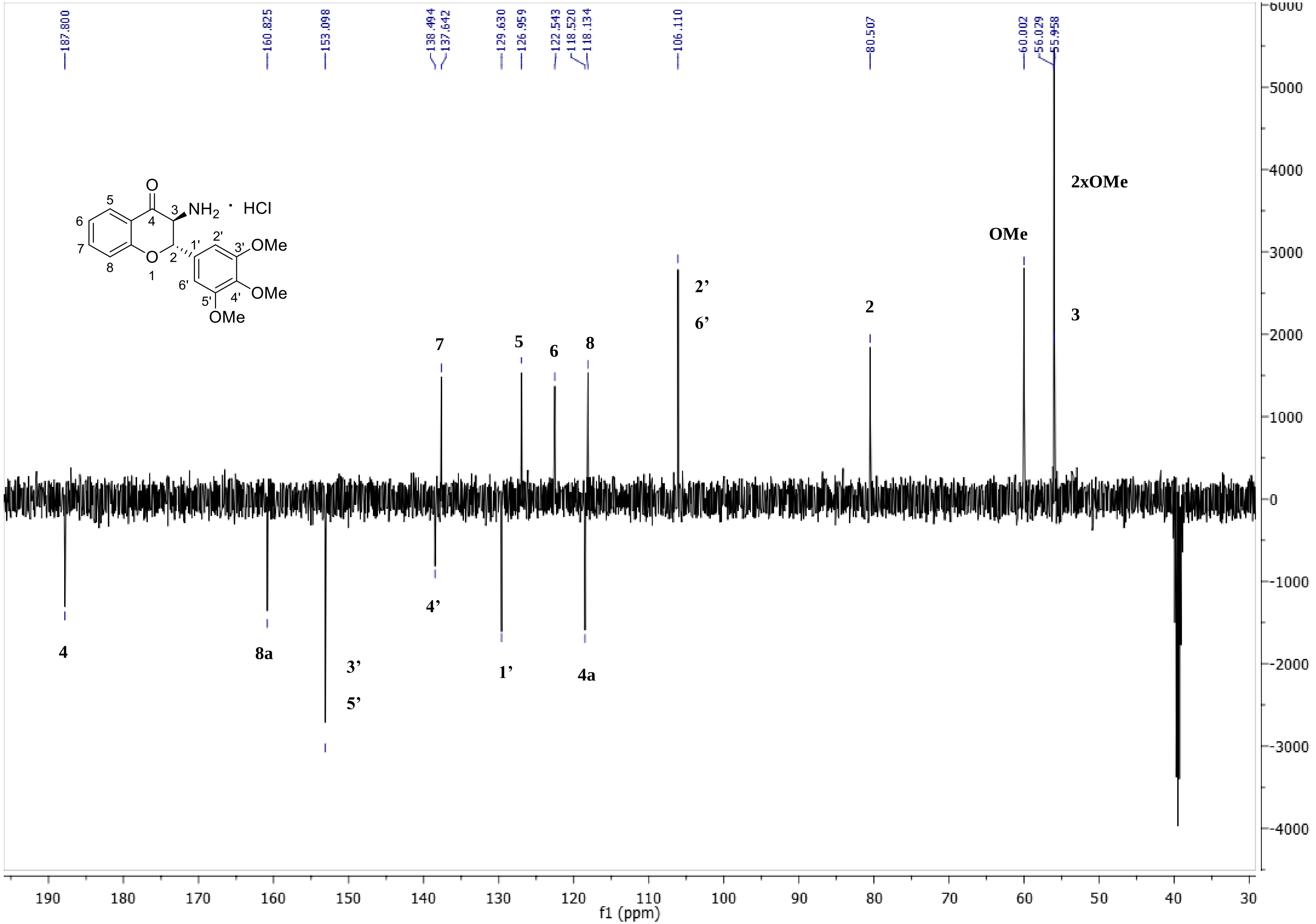

Figure S38. ^{13}C-NMR spectrum of *rac-trans*-**1e** in DMSO-d_6

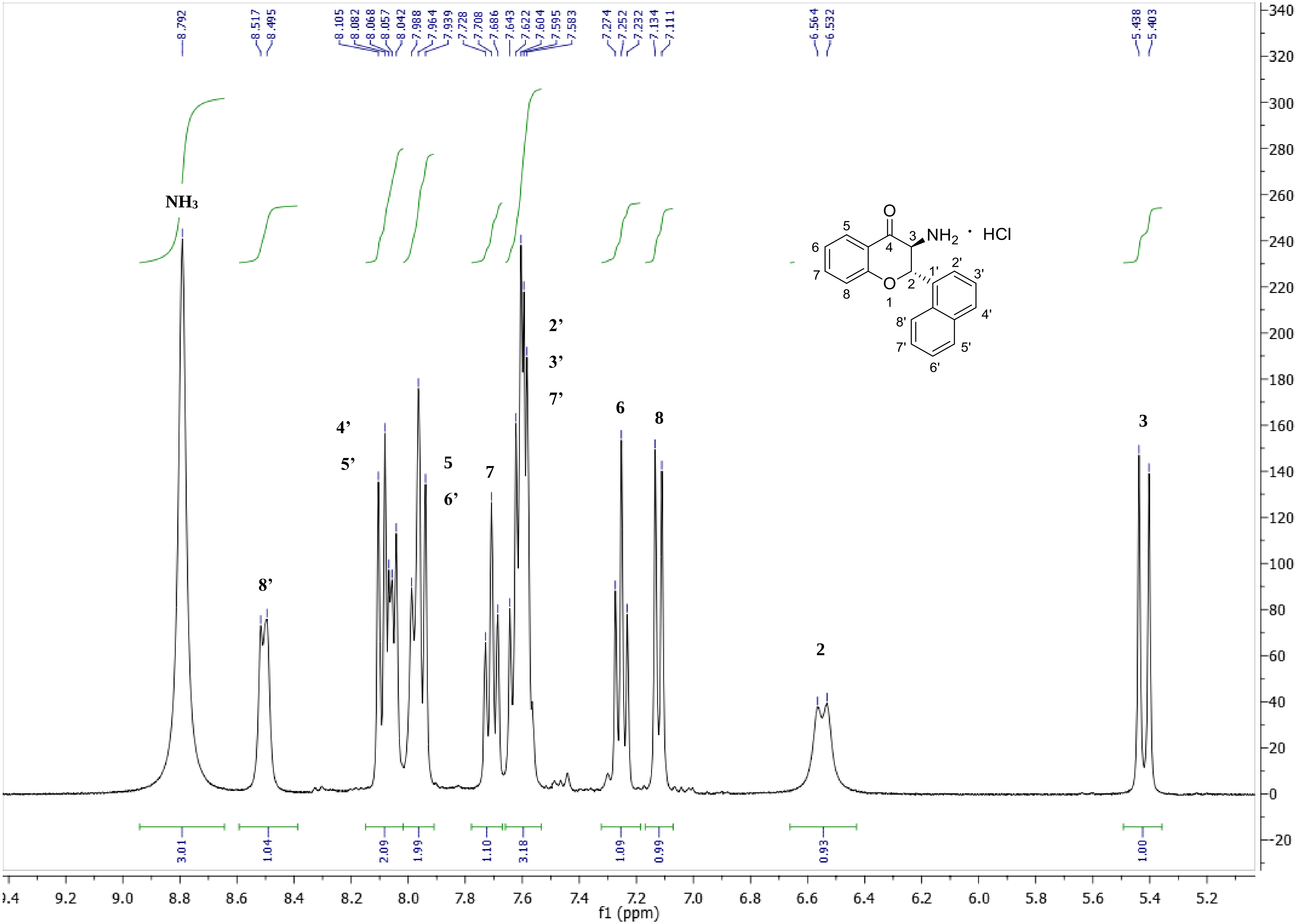

Figure S39. ^{1}H-NMR spectrum of *rac-trans*-**1f** in DMSO-d$_6$

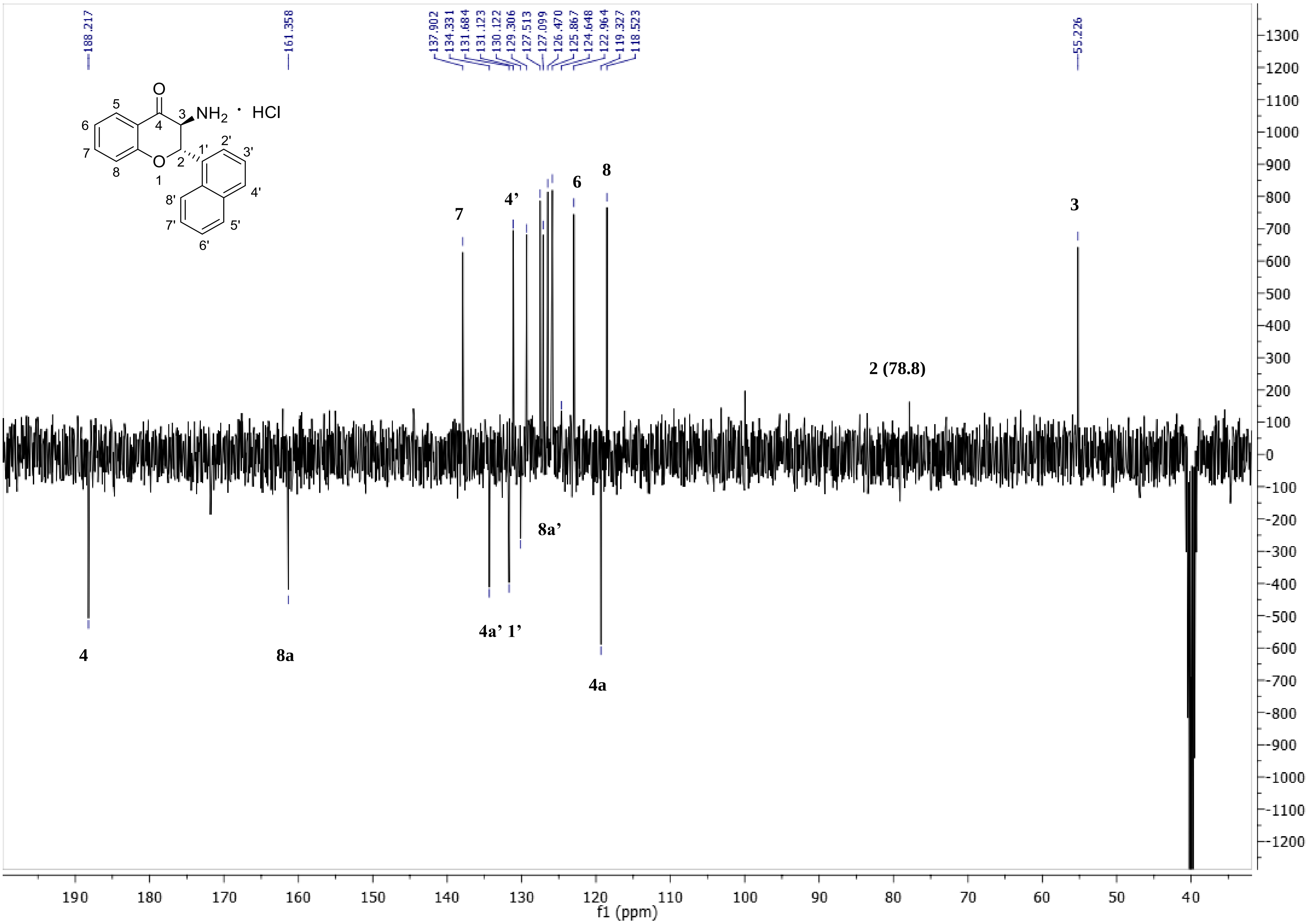

Figure S40. ^{13}C-NMR spectrum of *rac-trans*-**1f** in DMSO-d_6

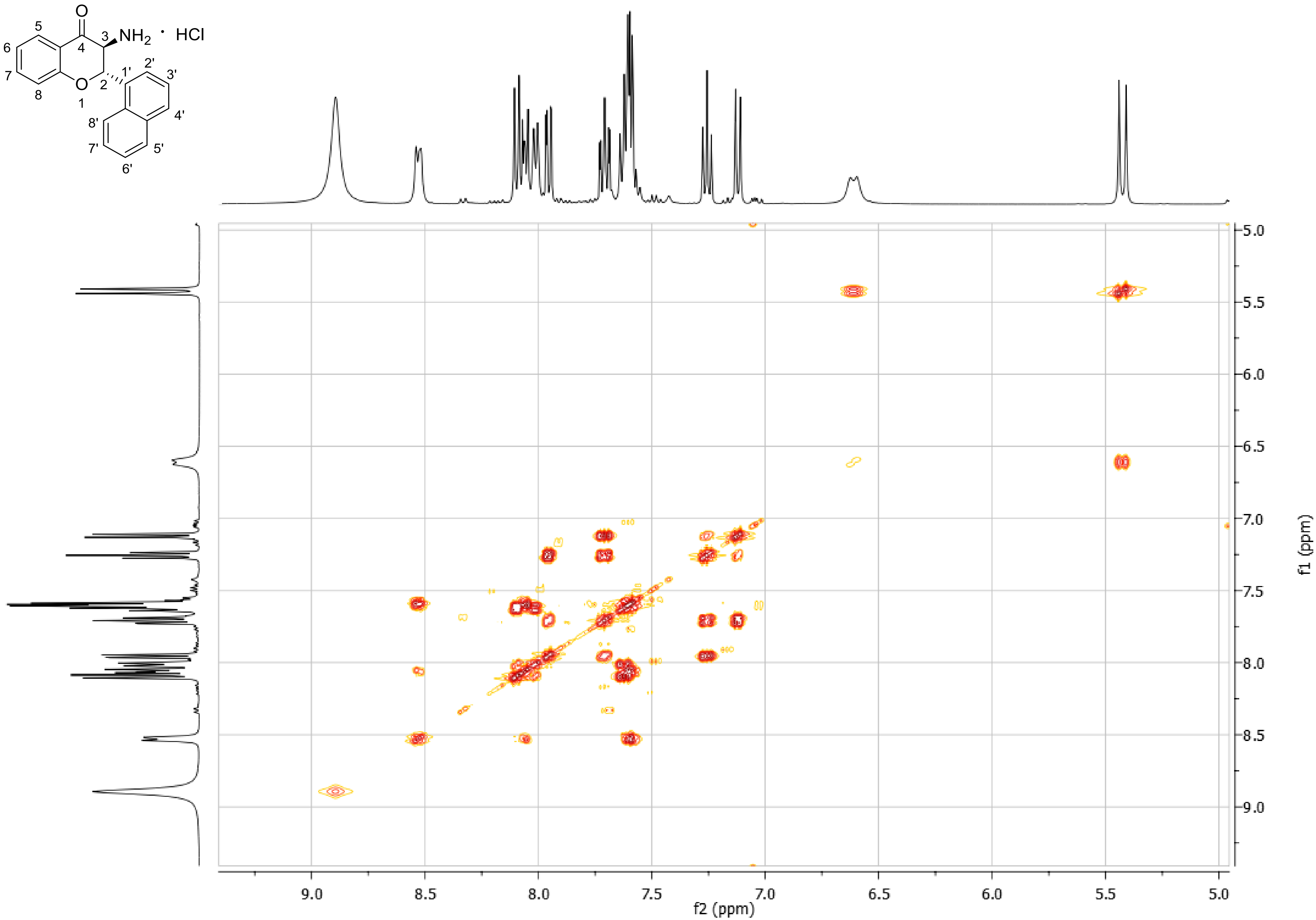

Figure S41. COSY-spectrum of *rac-trans*-**1f** in DMSO-d_6

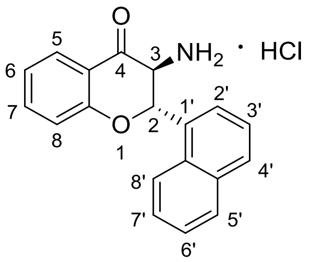

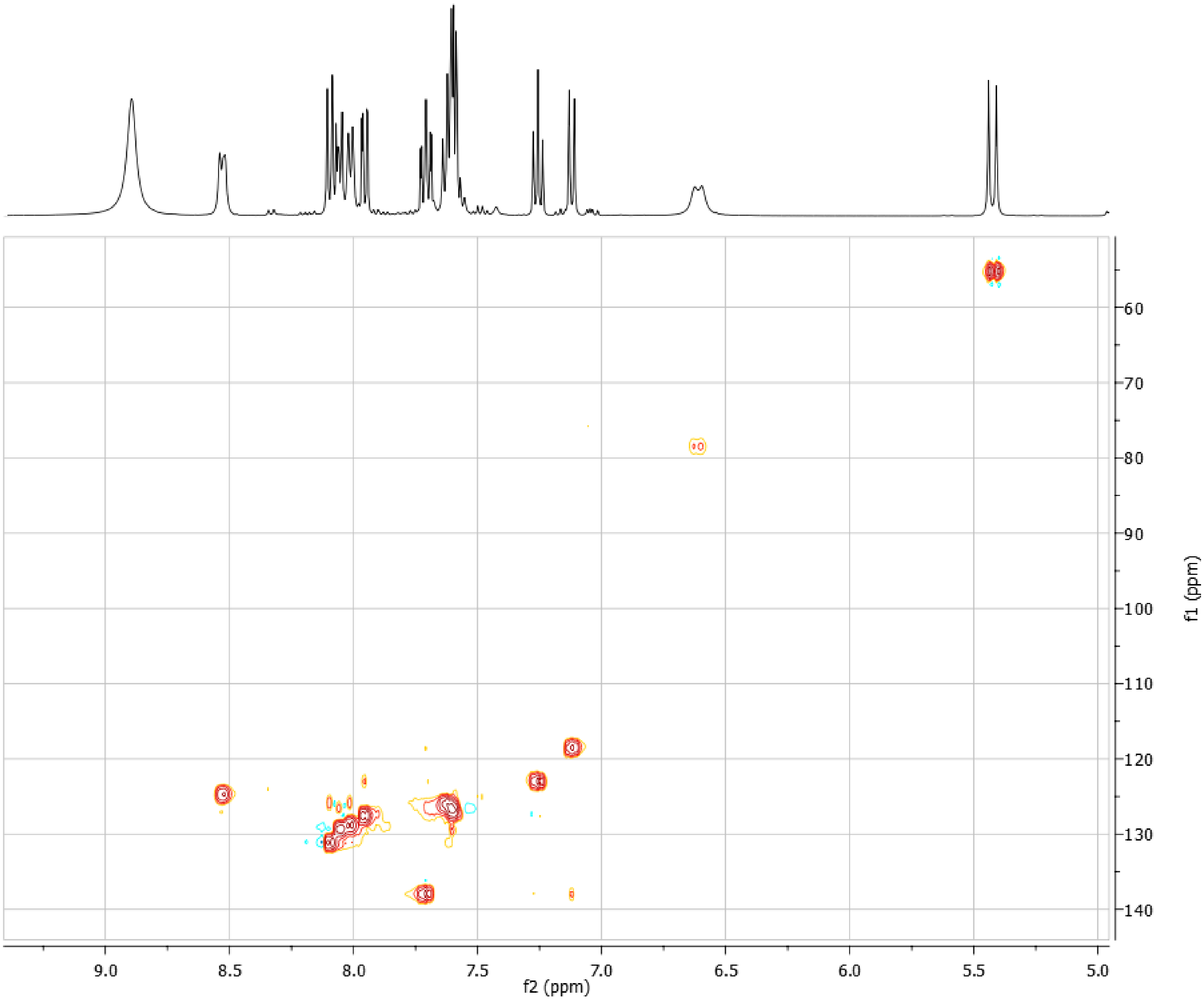

Figure S42. HSQC-spectrum of *rac-trans*-**1f** in DMSO-d_6

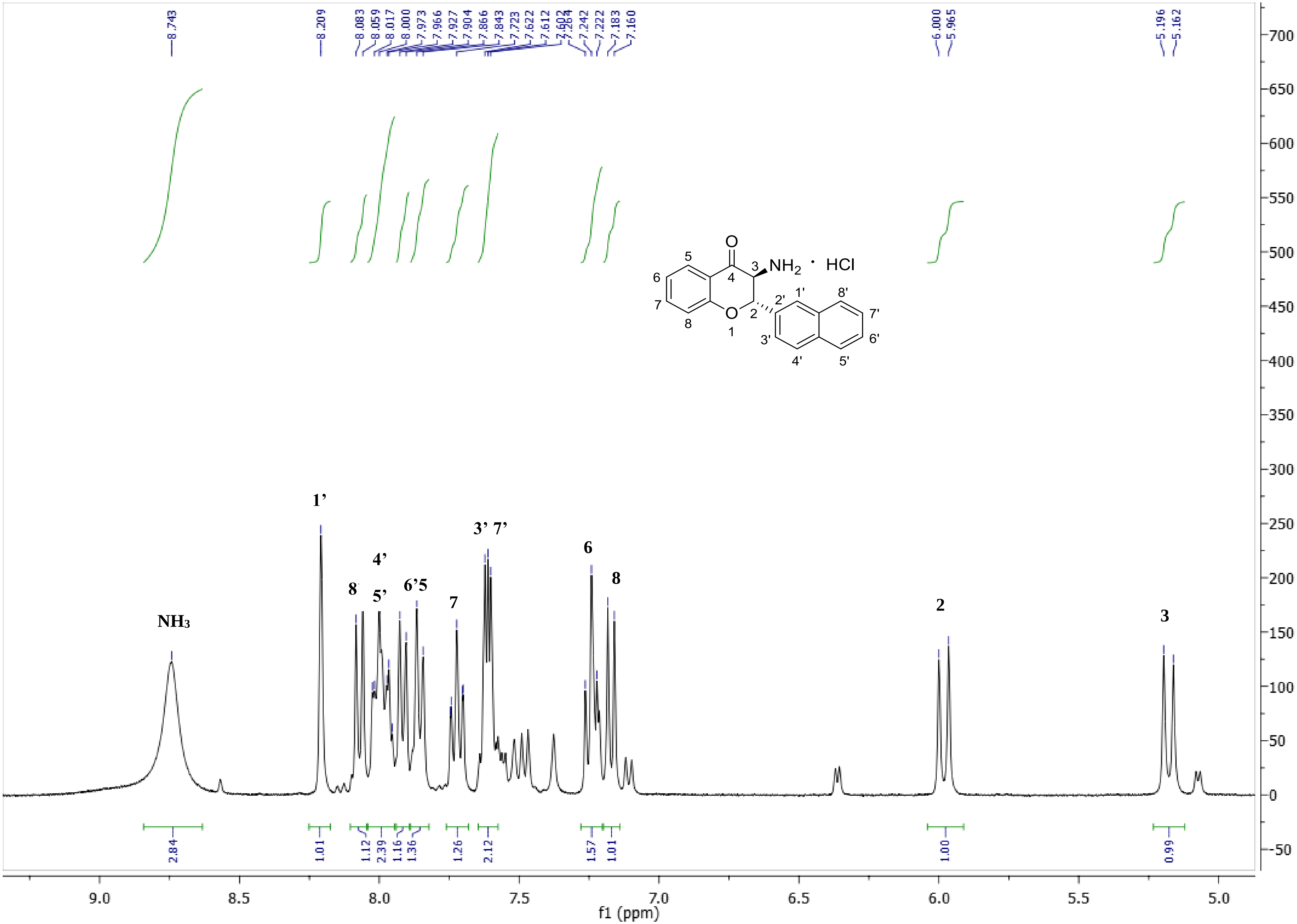

Figure S43. ^{1}H-NMR spectrum of *rac-trans*-**1g** in DMSO-d$_6$

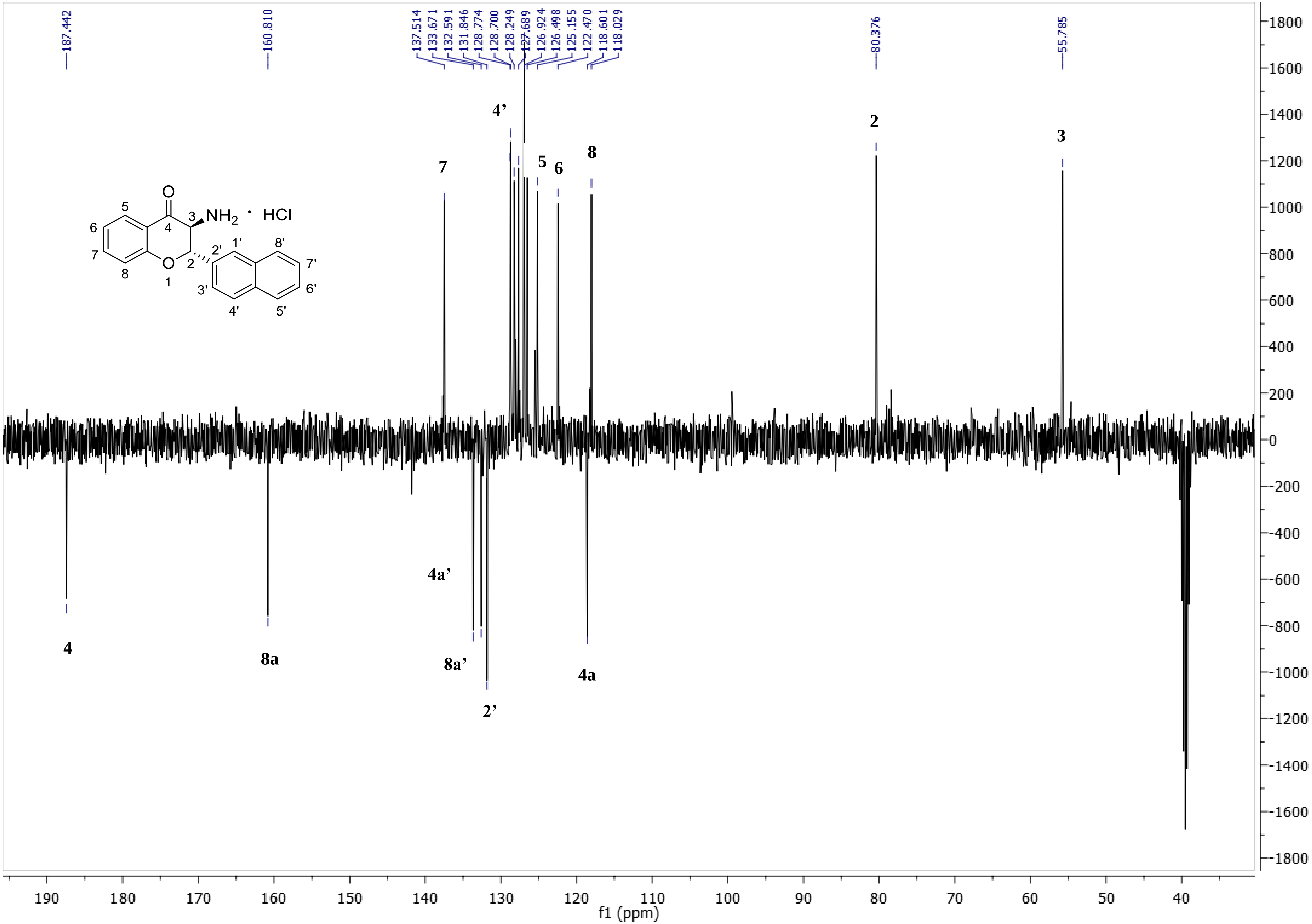

Figure S44. ^{13}C-NMR spectrum of *rac*-*trans*-**1g** in DMSO-d$_6$

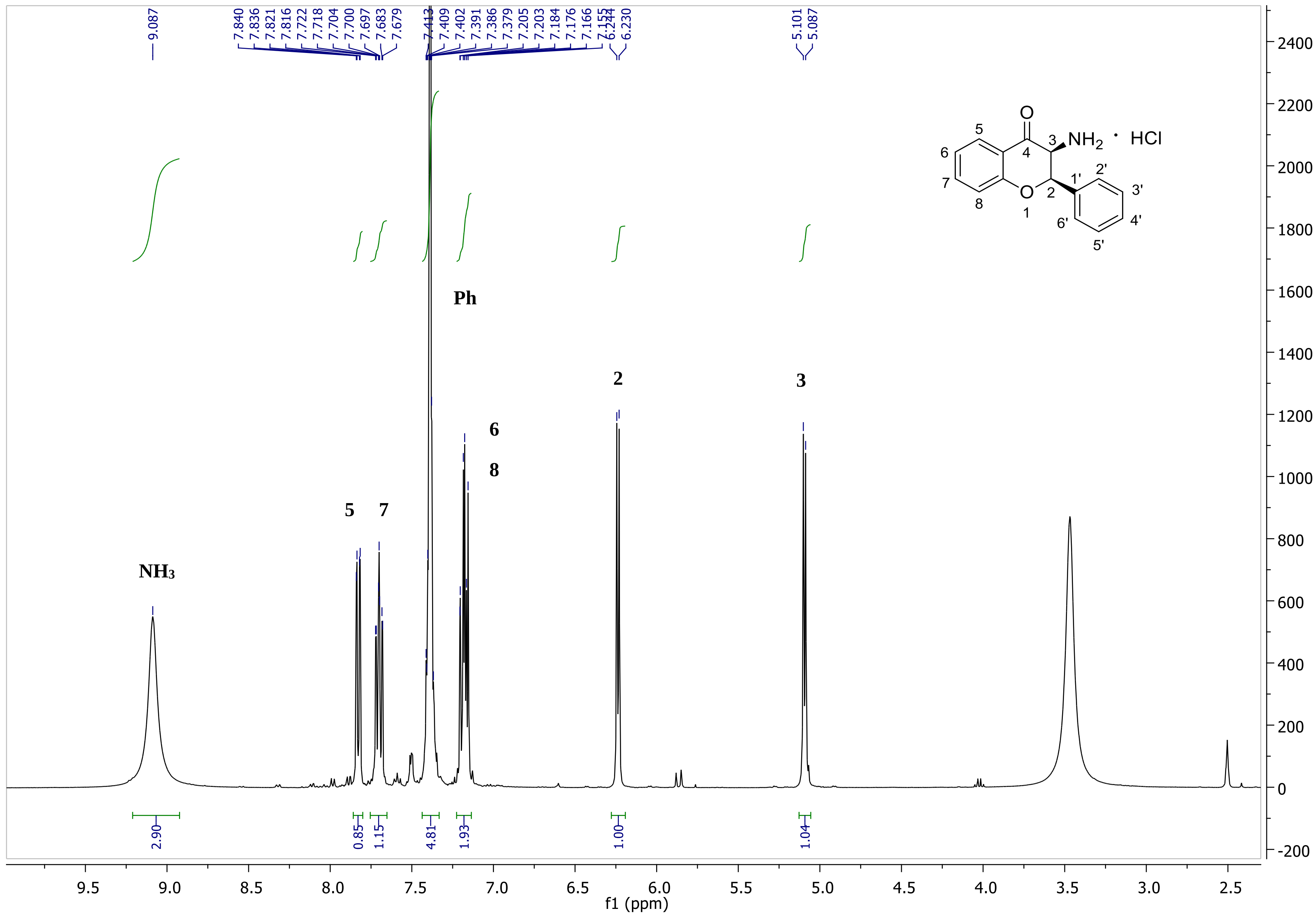

Figure S45. ^{1}H-NMR spectrum of *rac*-*cis*-**1a** in DMSO-d_6

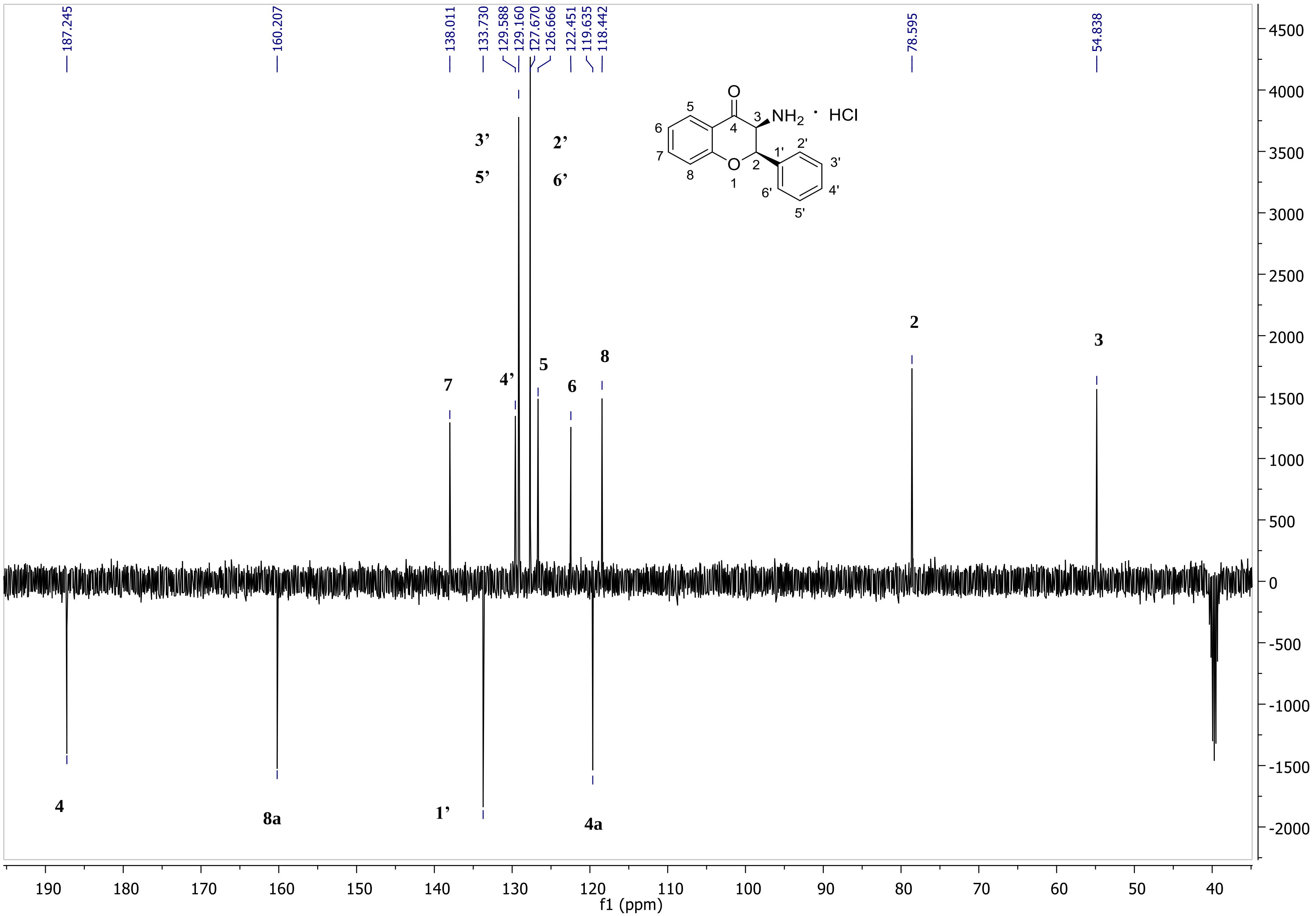

Figure S46. ^{13}C-NMR spectrum of *rac*-*cis*-**1a** in DMSO-d_6

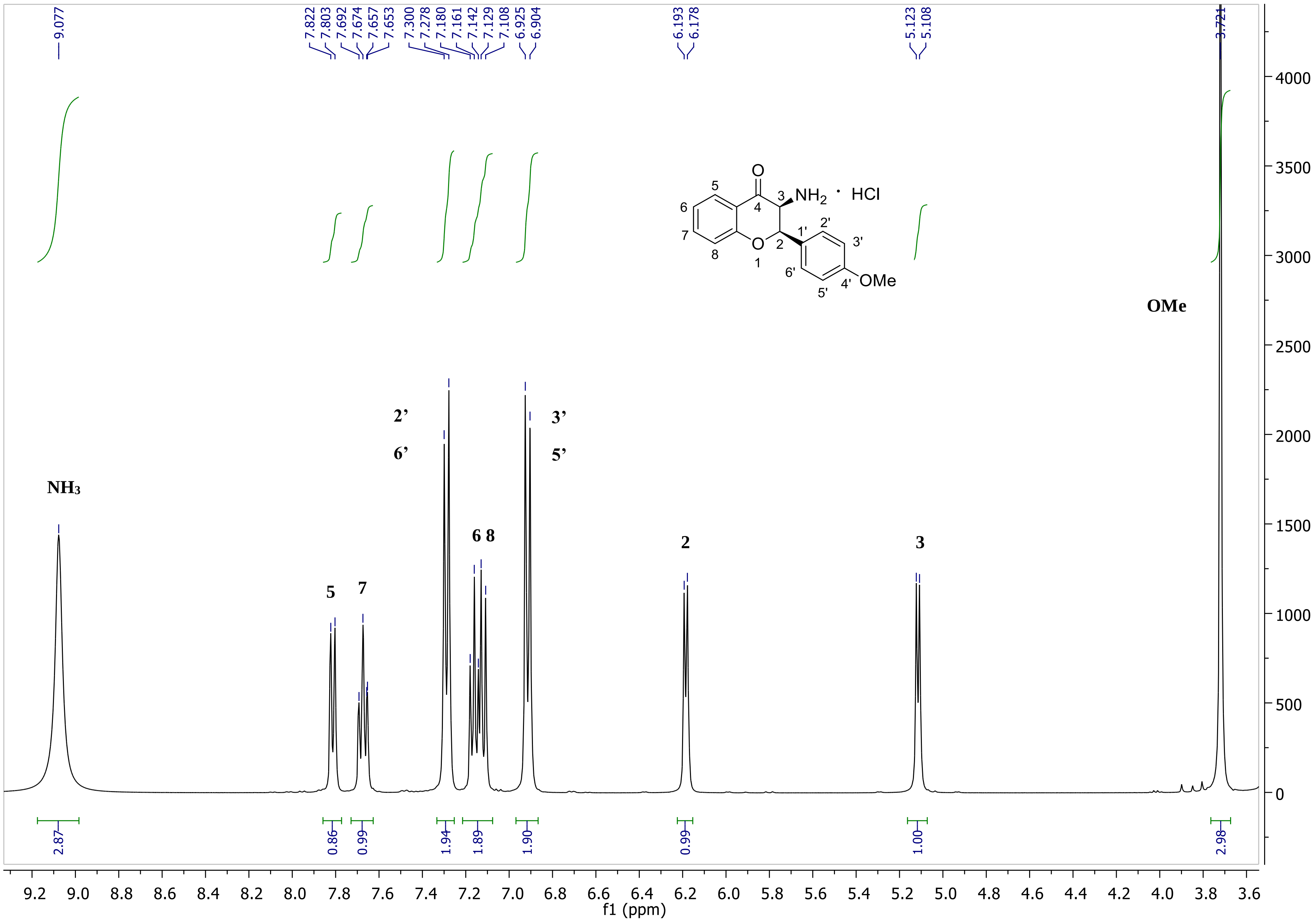

Figure S47. ^{1}H-NMR spectrum of *rac*-*cis*-**1b** in DMSO-d$_6$

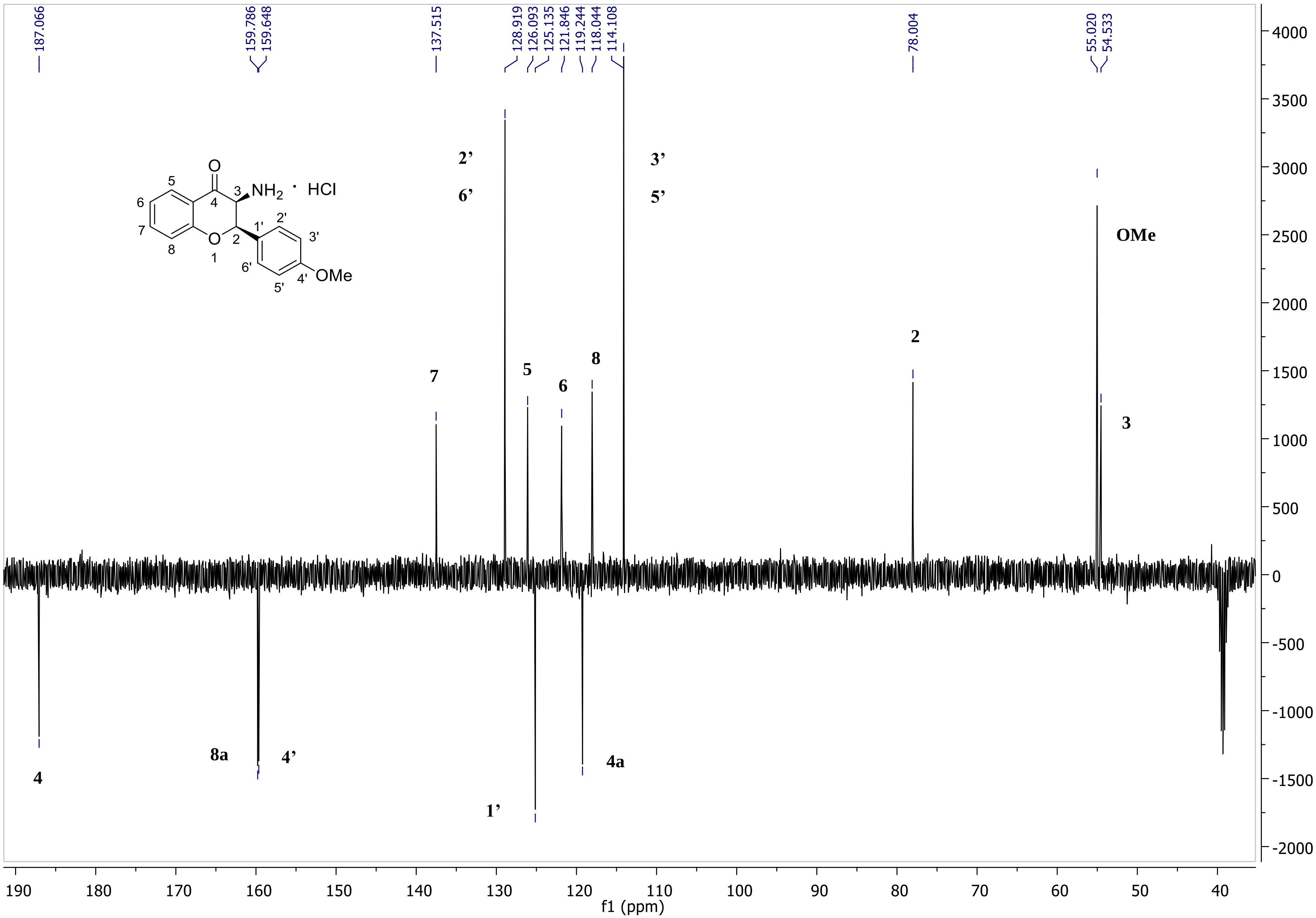

Figure S48. ^{13}C-NMR spectrum of *rac*-*cis*-**1b** in DMSO-d$_6$

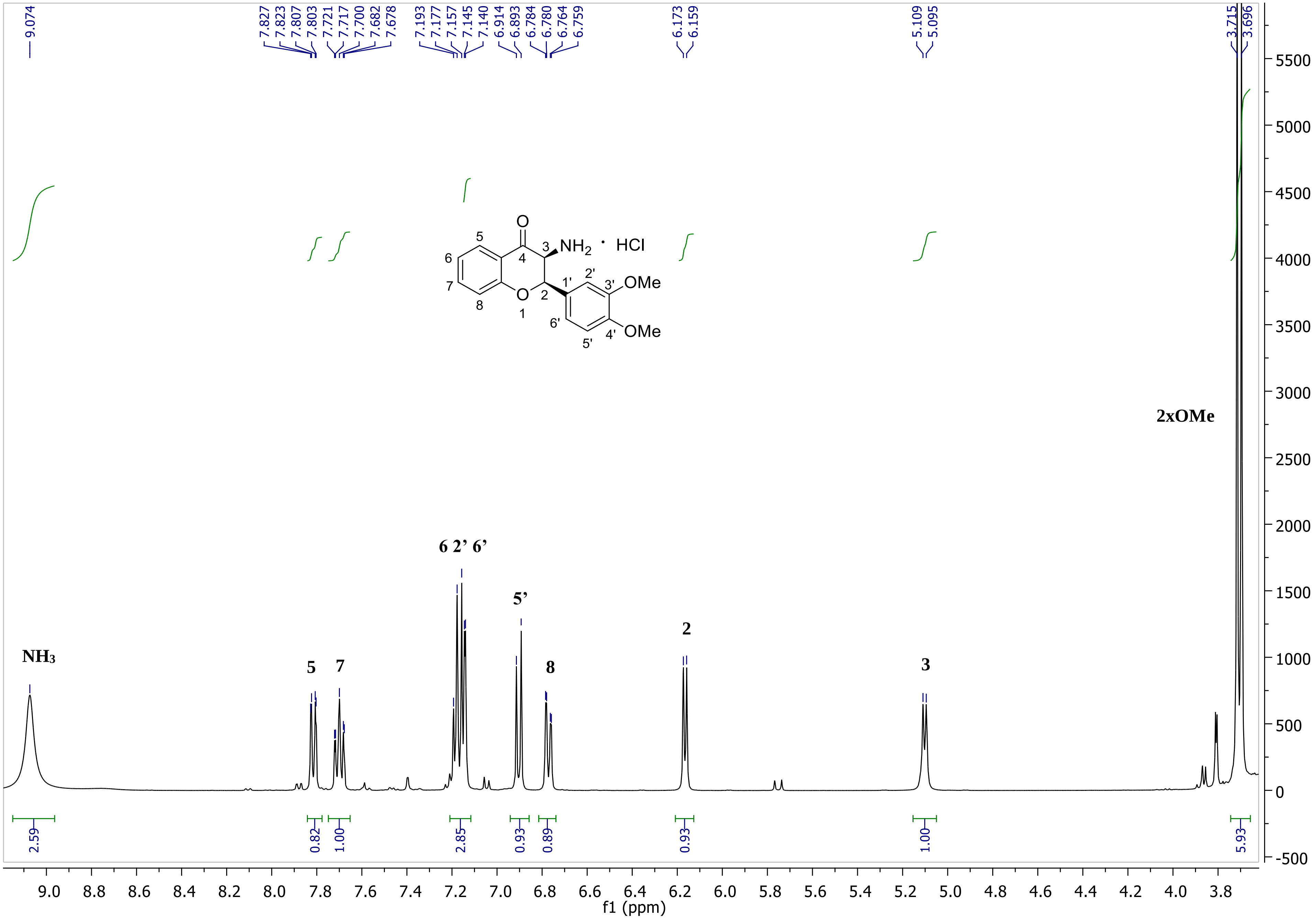

Figure S49. ^{1}H-NMR spectrum of *rac-cis*-**1c** in DMSO-d$_6$

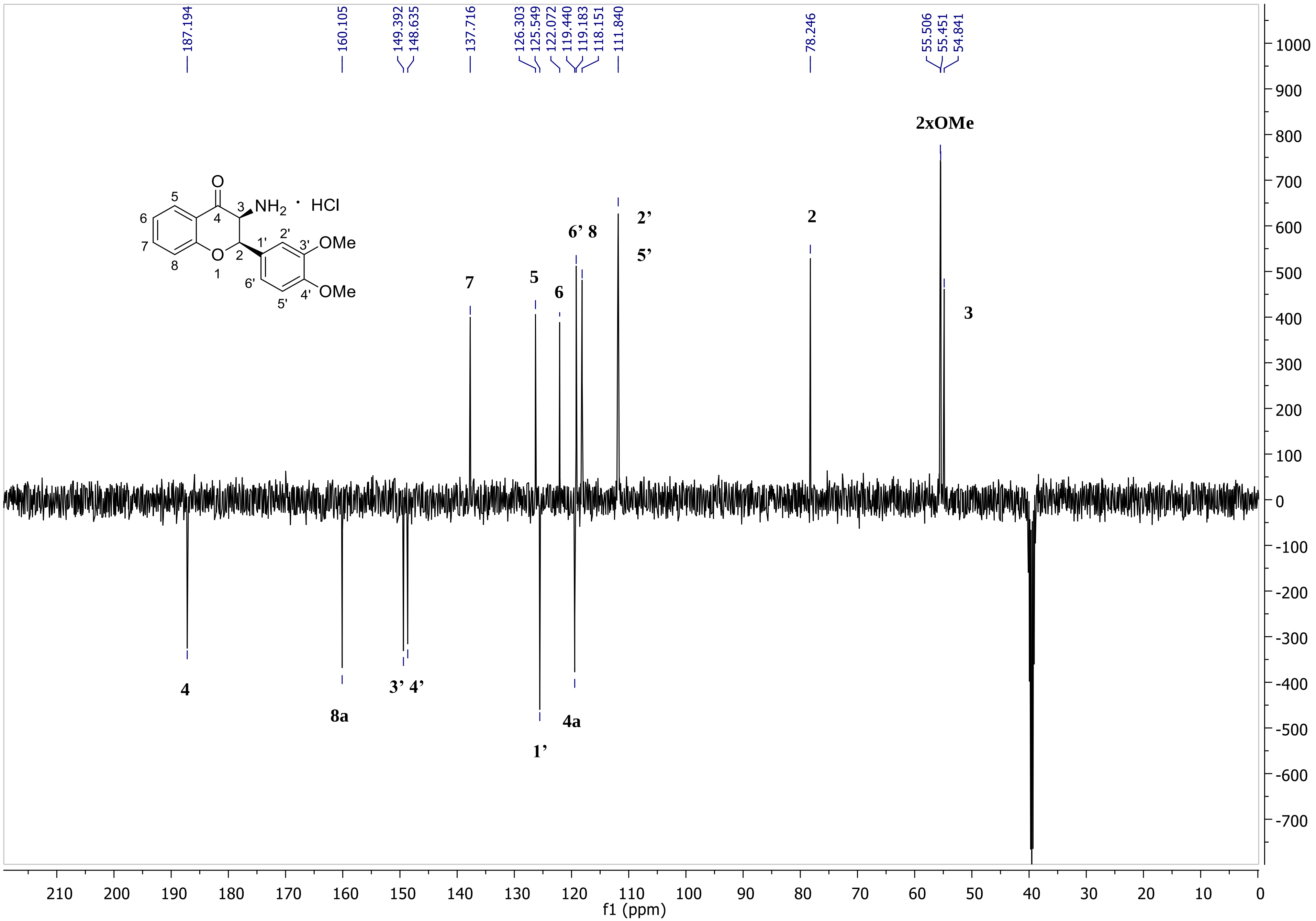

Figure S50. ^{13}C-NMR spectrum of *rac*-*cis*-**1c** in DMSO-d$_6$

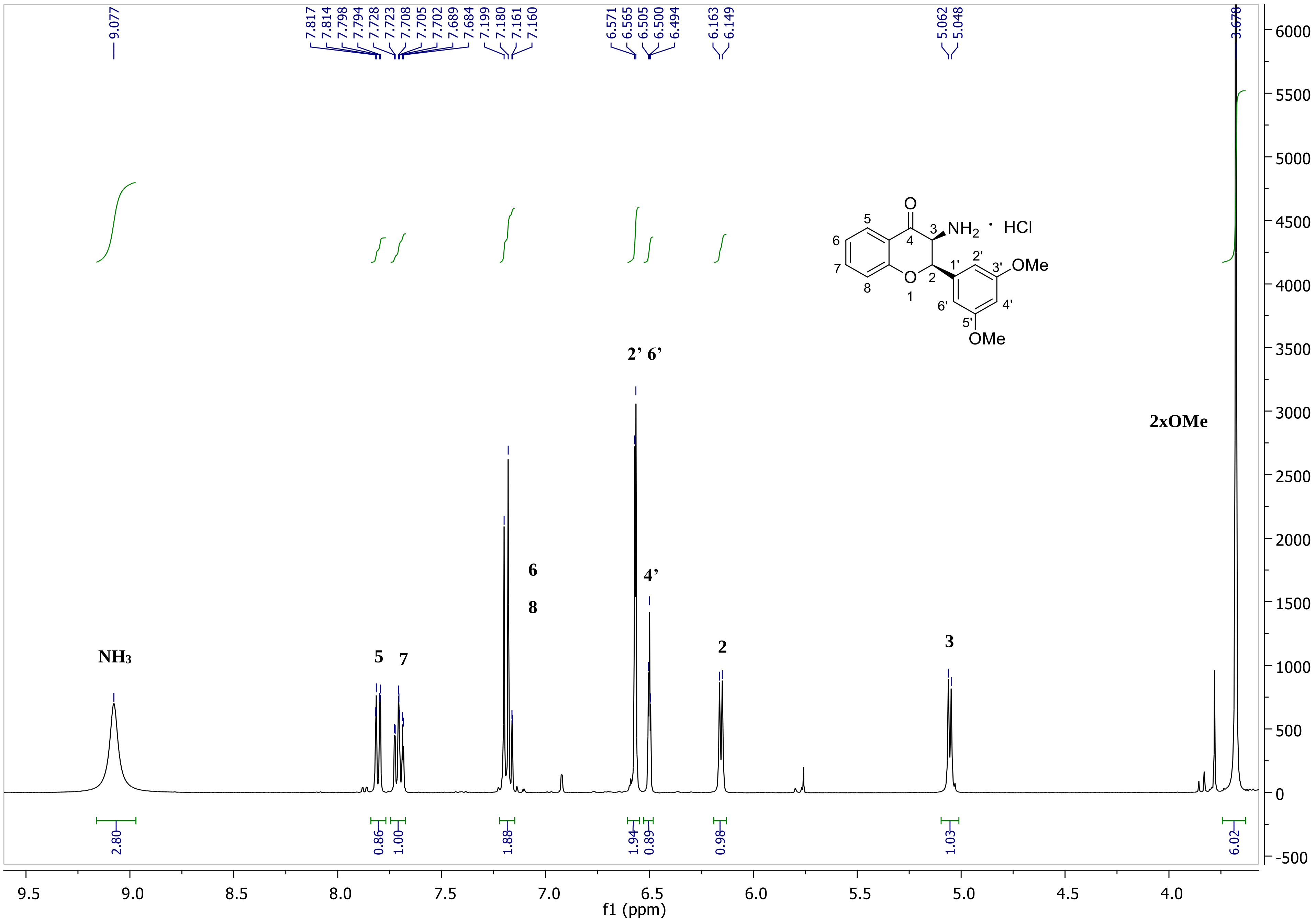

Figure S51. ^{1}H-NMR spectrum of *rac-cis*-**1d** in DMSO-d$_6$

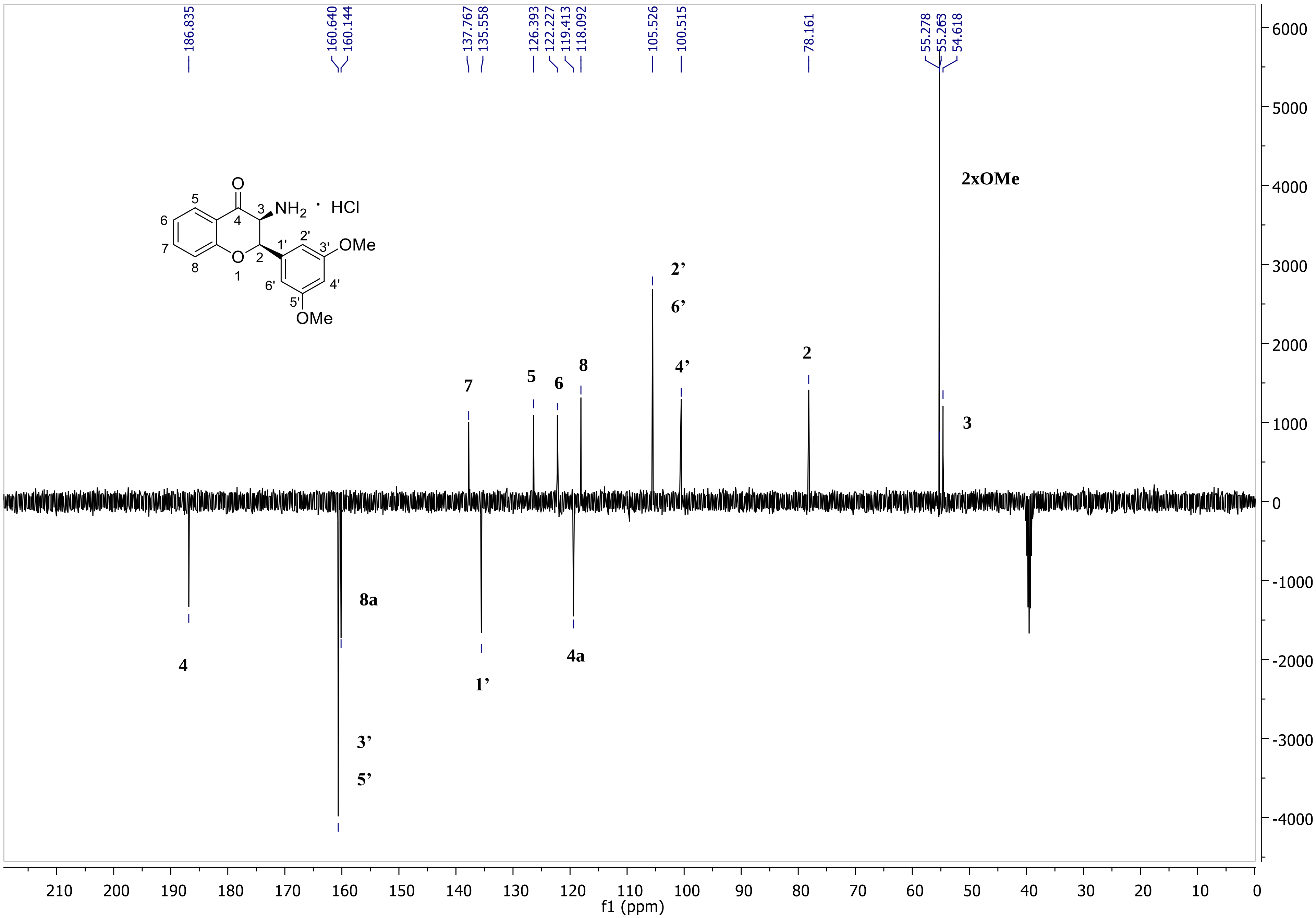

Figure S52. ^{13}C-NMR spectrum of *rac*-*cis*-**1d** in DMSO-d_6

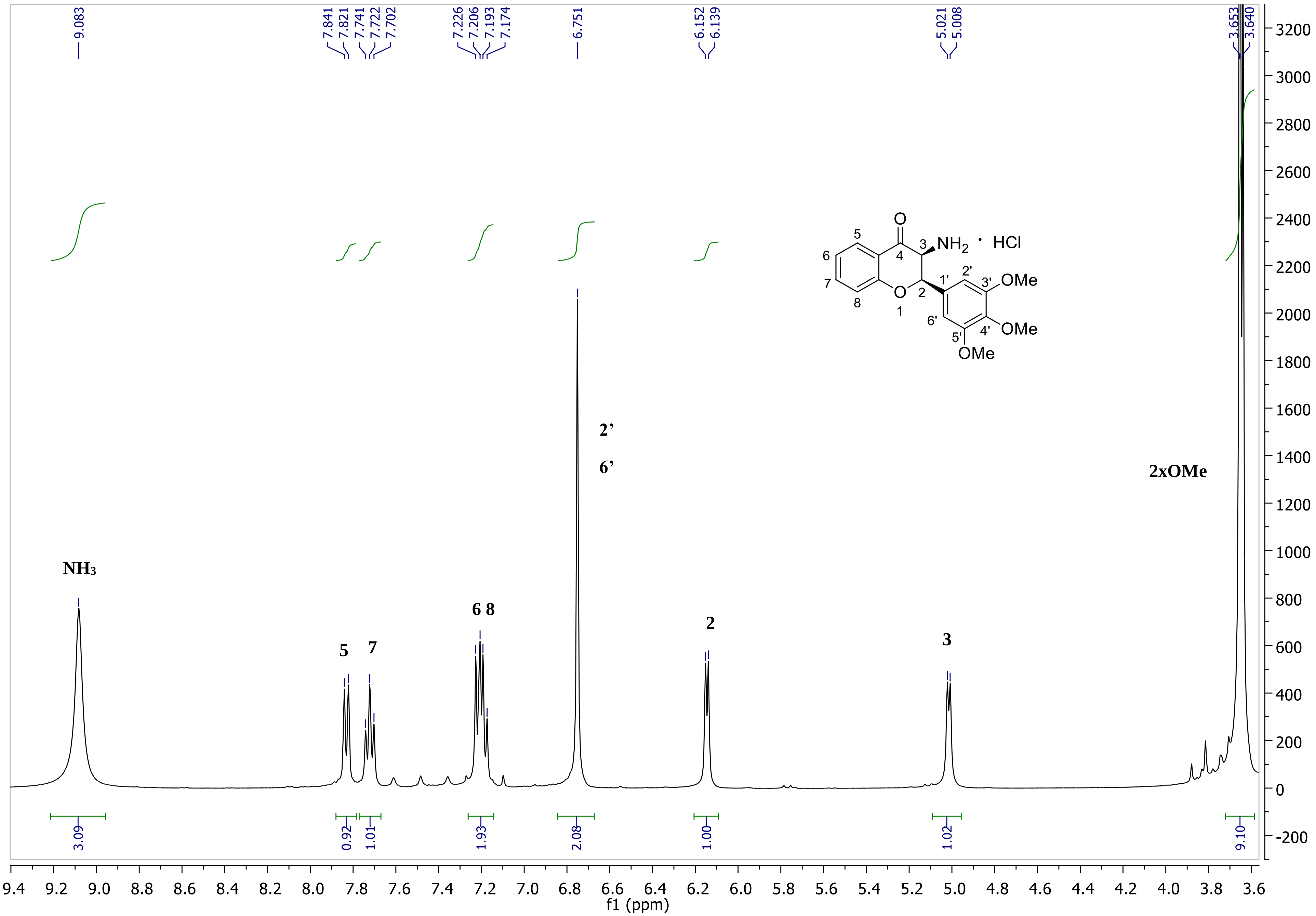

Figure S53. ^{1}H-NMR spectrum of *rac*-*cis*-**1e** in DMSO-d_6

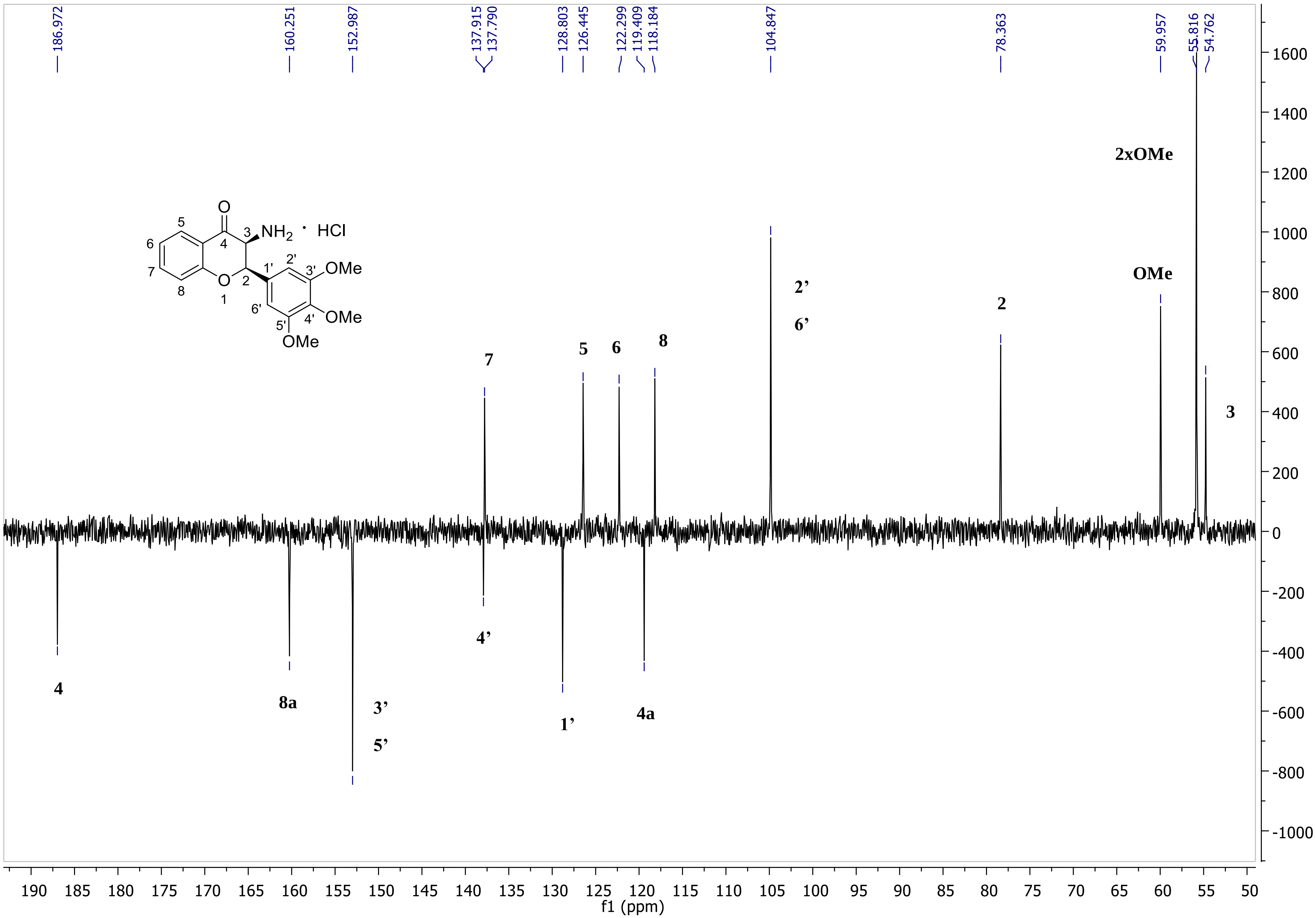

Figure S54. ^{13}C-NMR spectrum of *rac*-*cis*-**1e** in DMSO-d_6

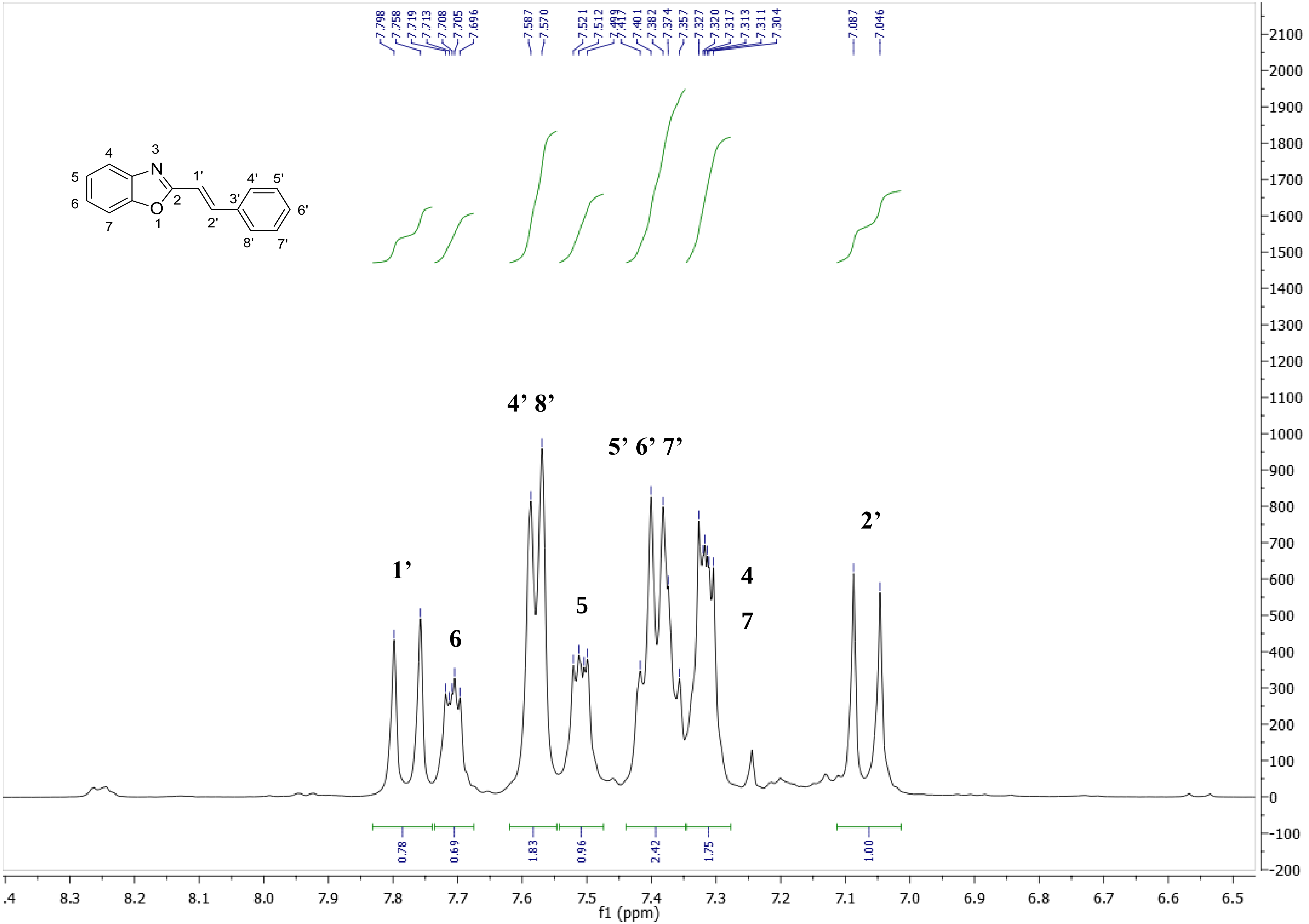

Figure S55. ^{1}H-NMR spectrum of **17a** in $CDCl_3$

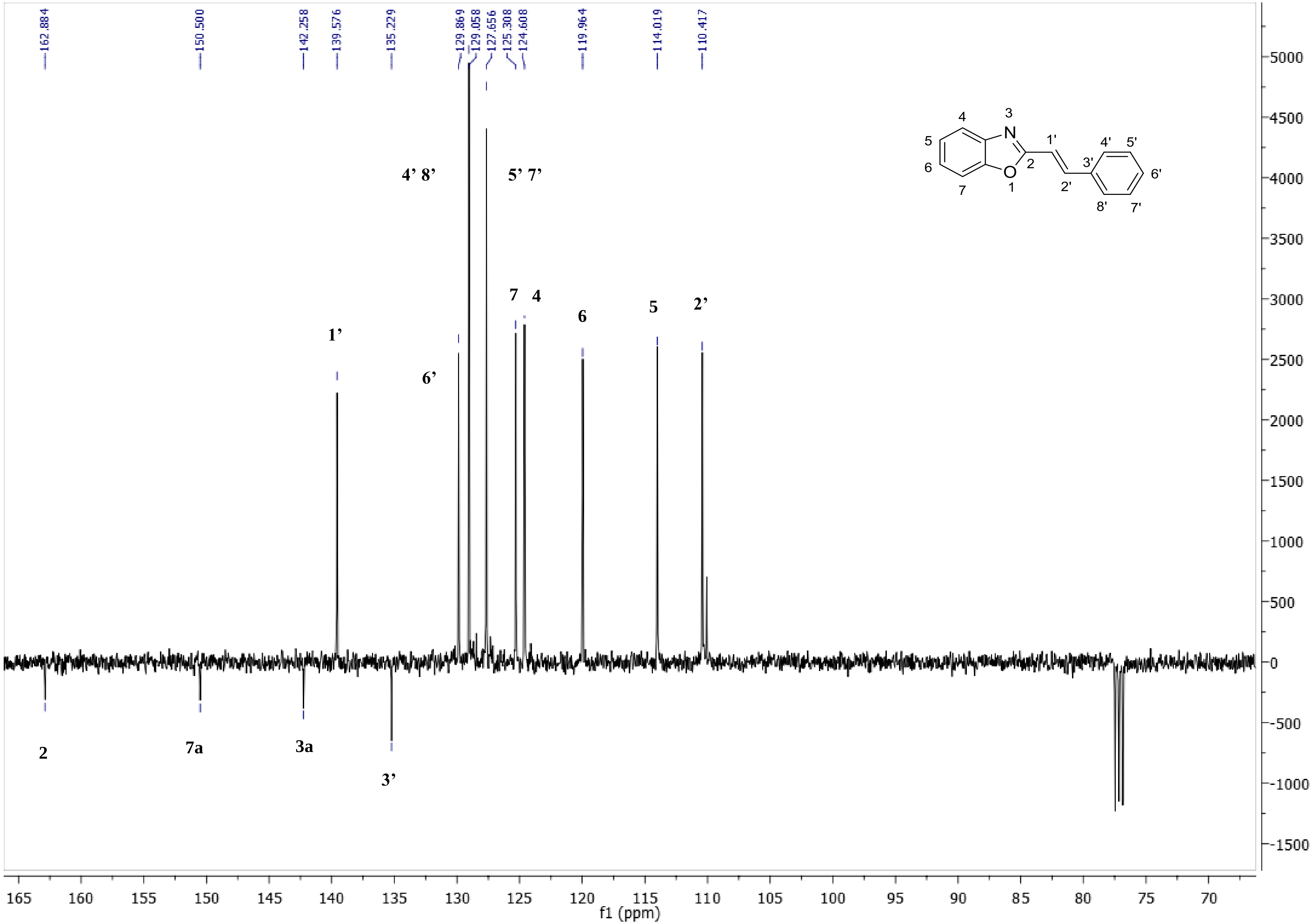

Figure S56. ^{13}C-NMR spectrum of **17a** in $CDCl_3$

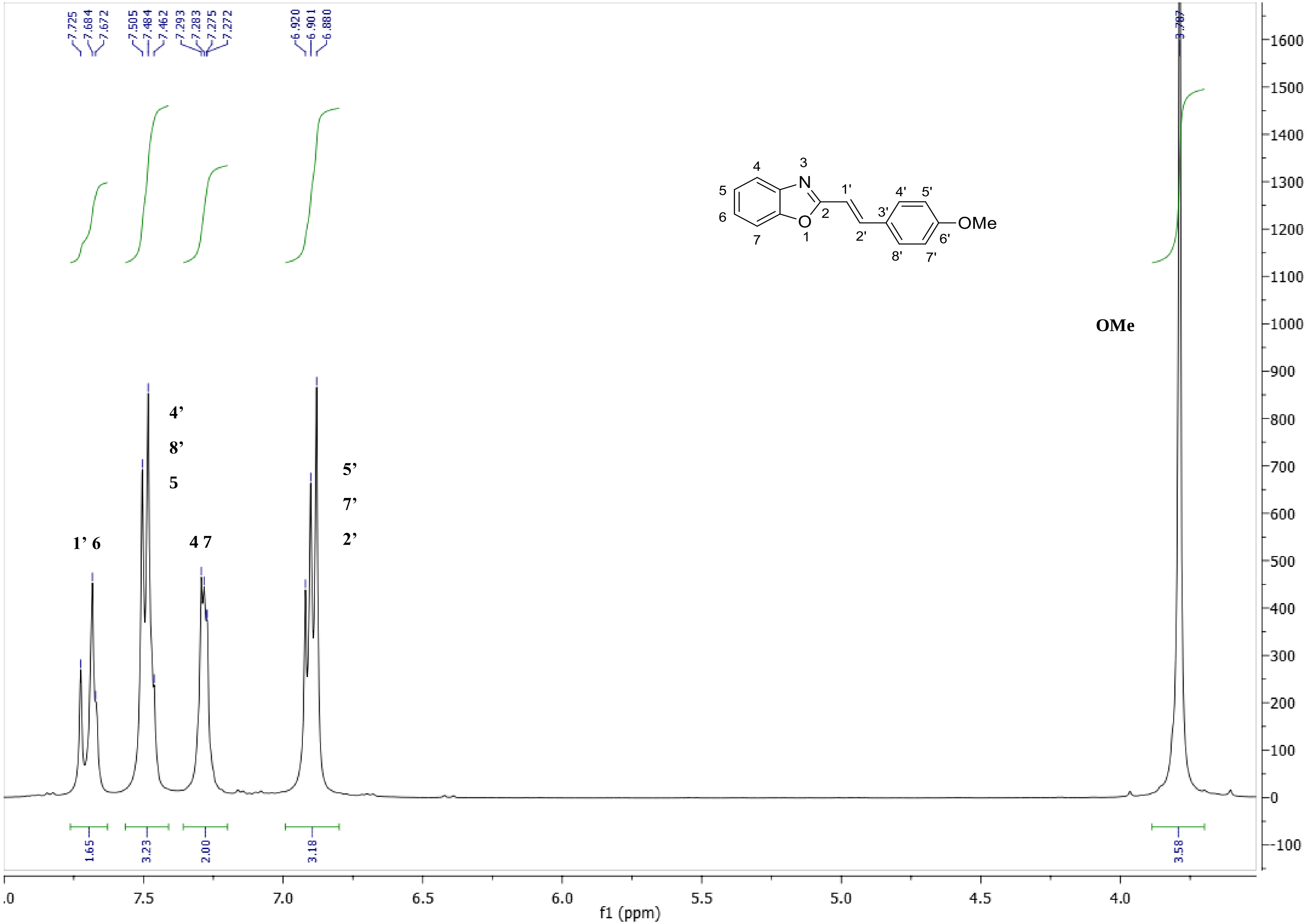

Figure S57. ^{1}H-NMR spectrum of **17b** in $CDCl_3$

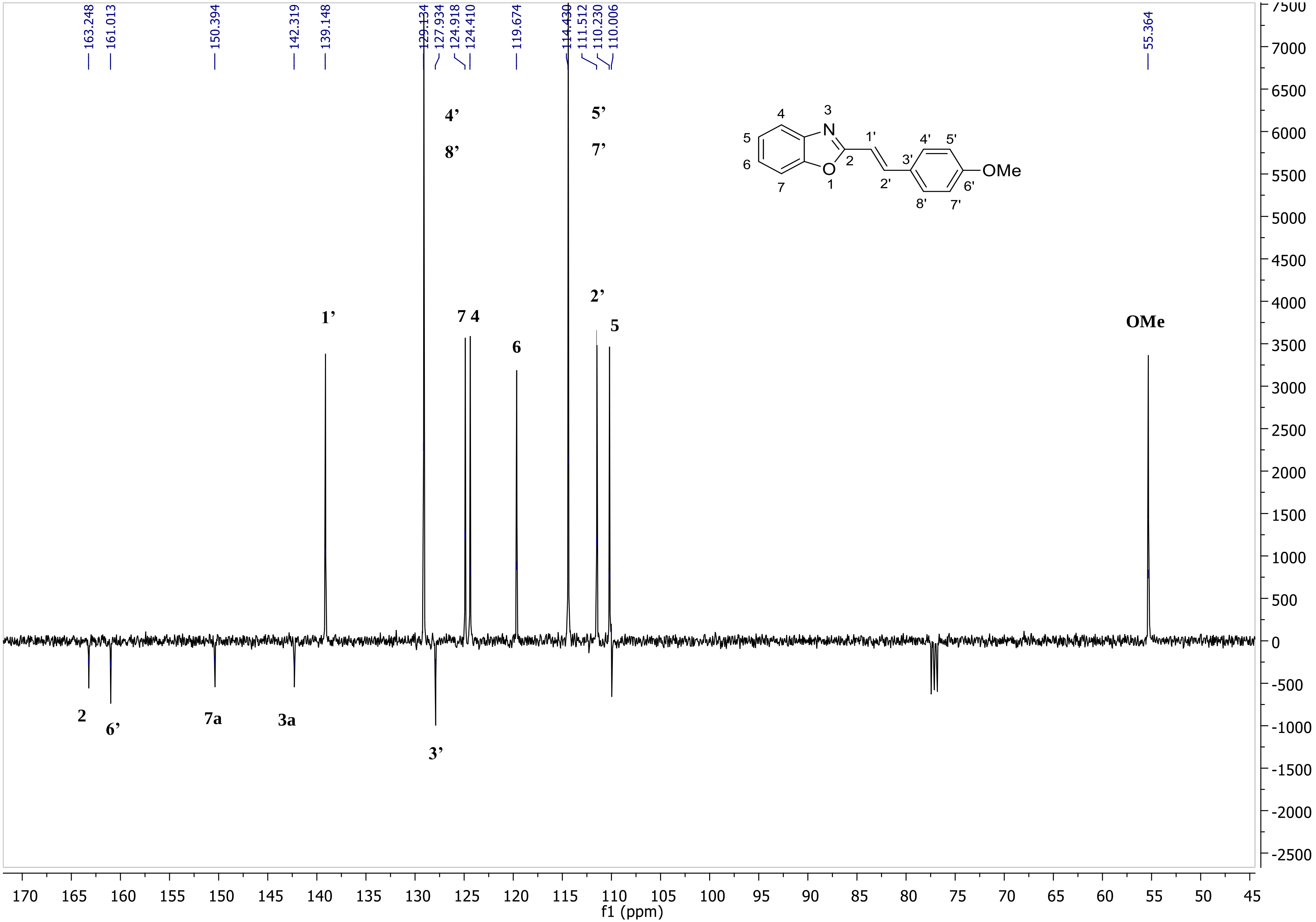

Figure S58. ^{13}C-NMR spectrum of **17b** in $CDCl_3$

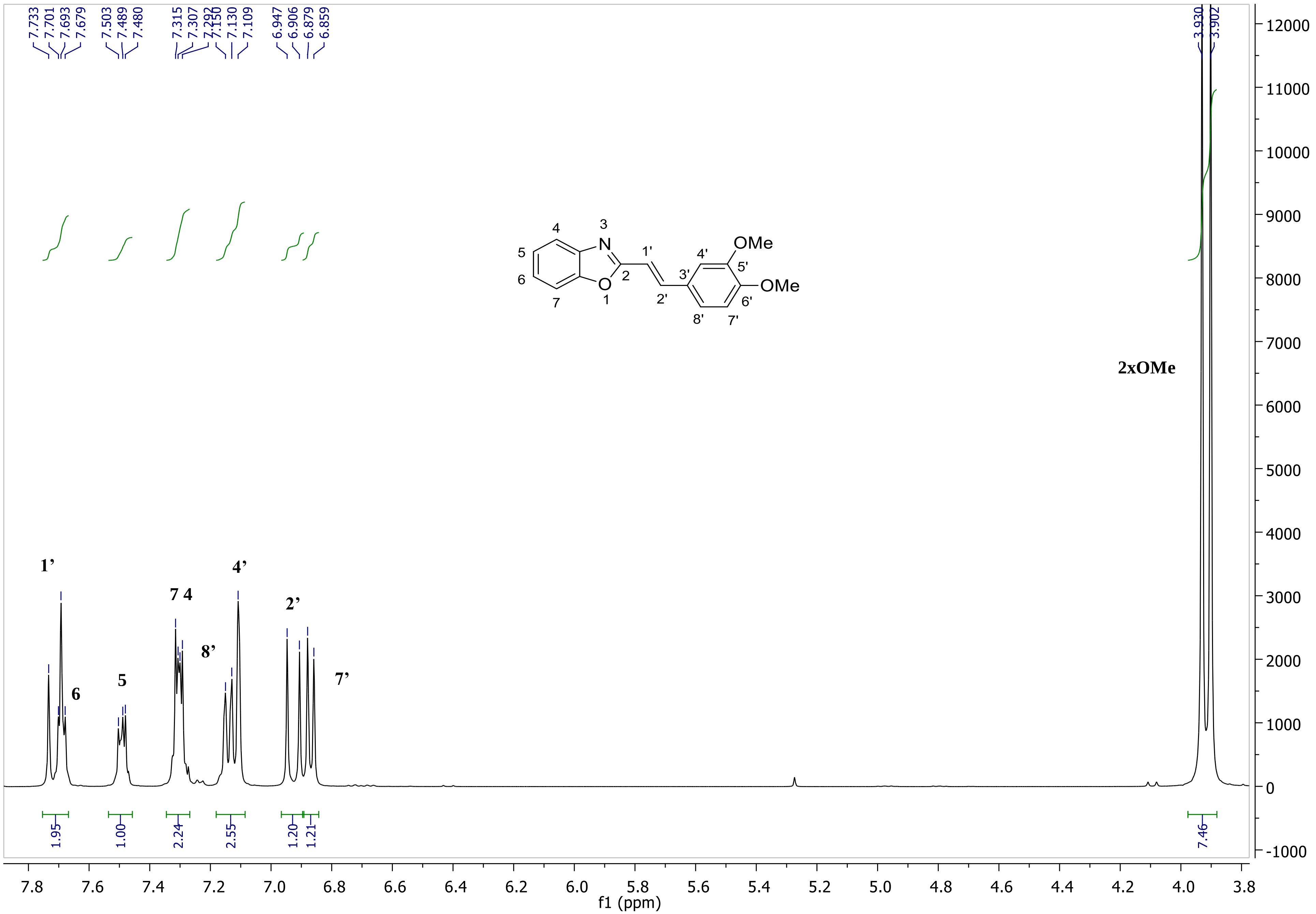

Figure S59. ^{1}H-NMR spectrum of **17c** in $CDCl_3$

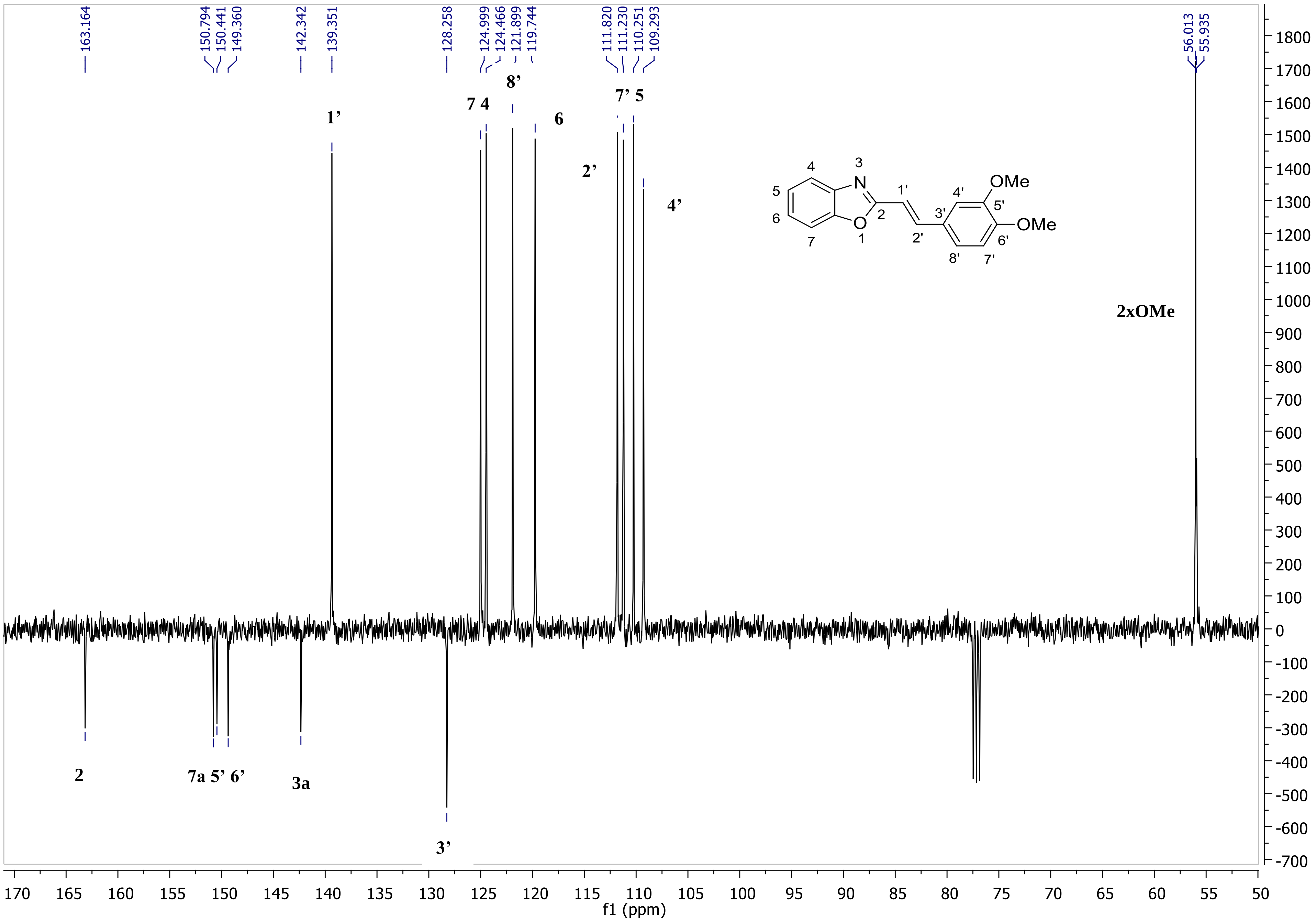

Figure S60. ^{13}C-NMR spectrum of **17c** in $CDCl_3$

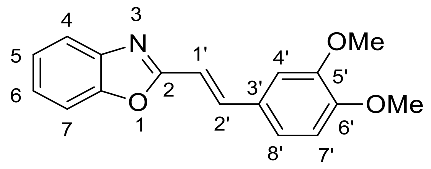

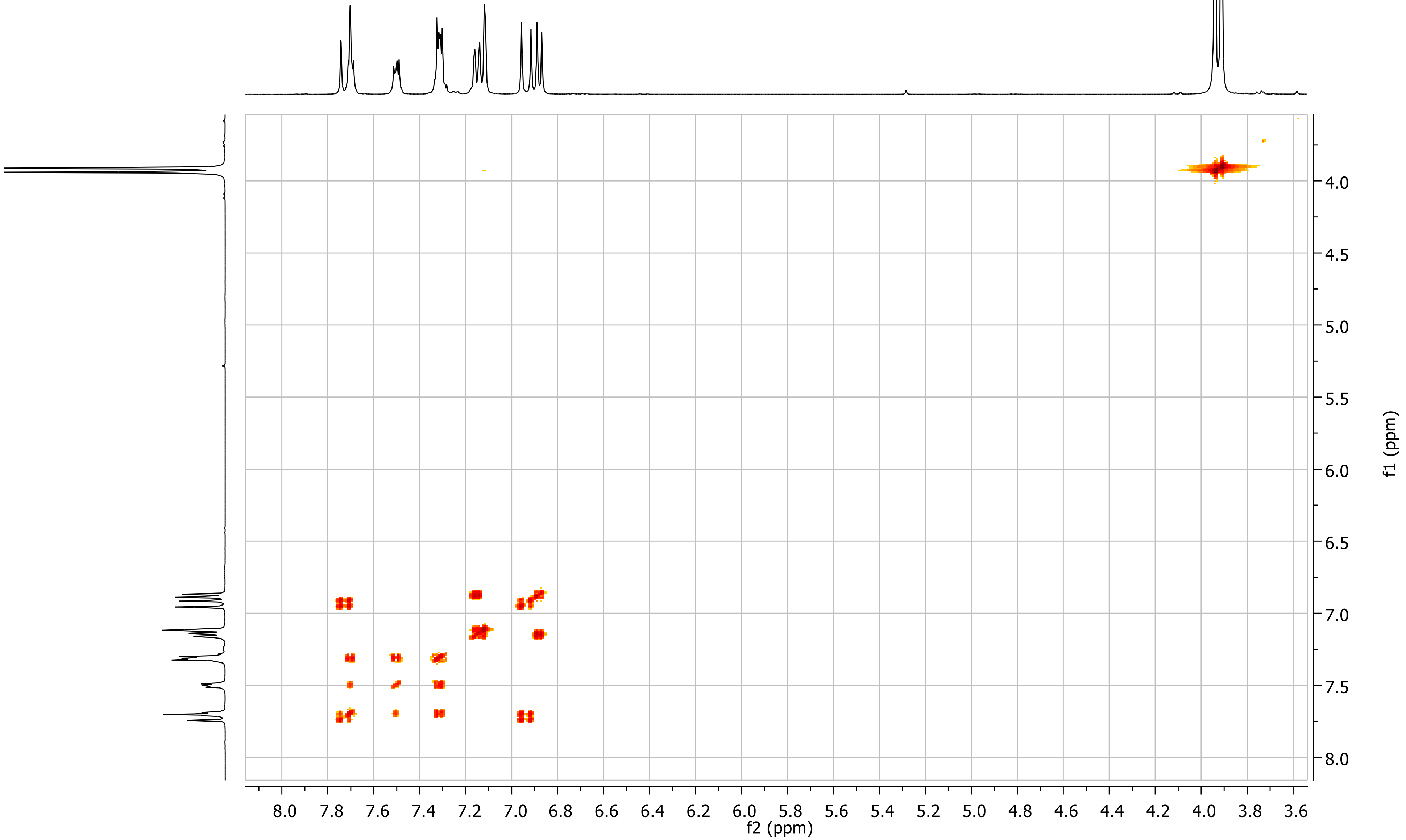

Figure S61. COSY-spectrum of **17c** in $CDCl_3$

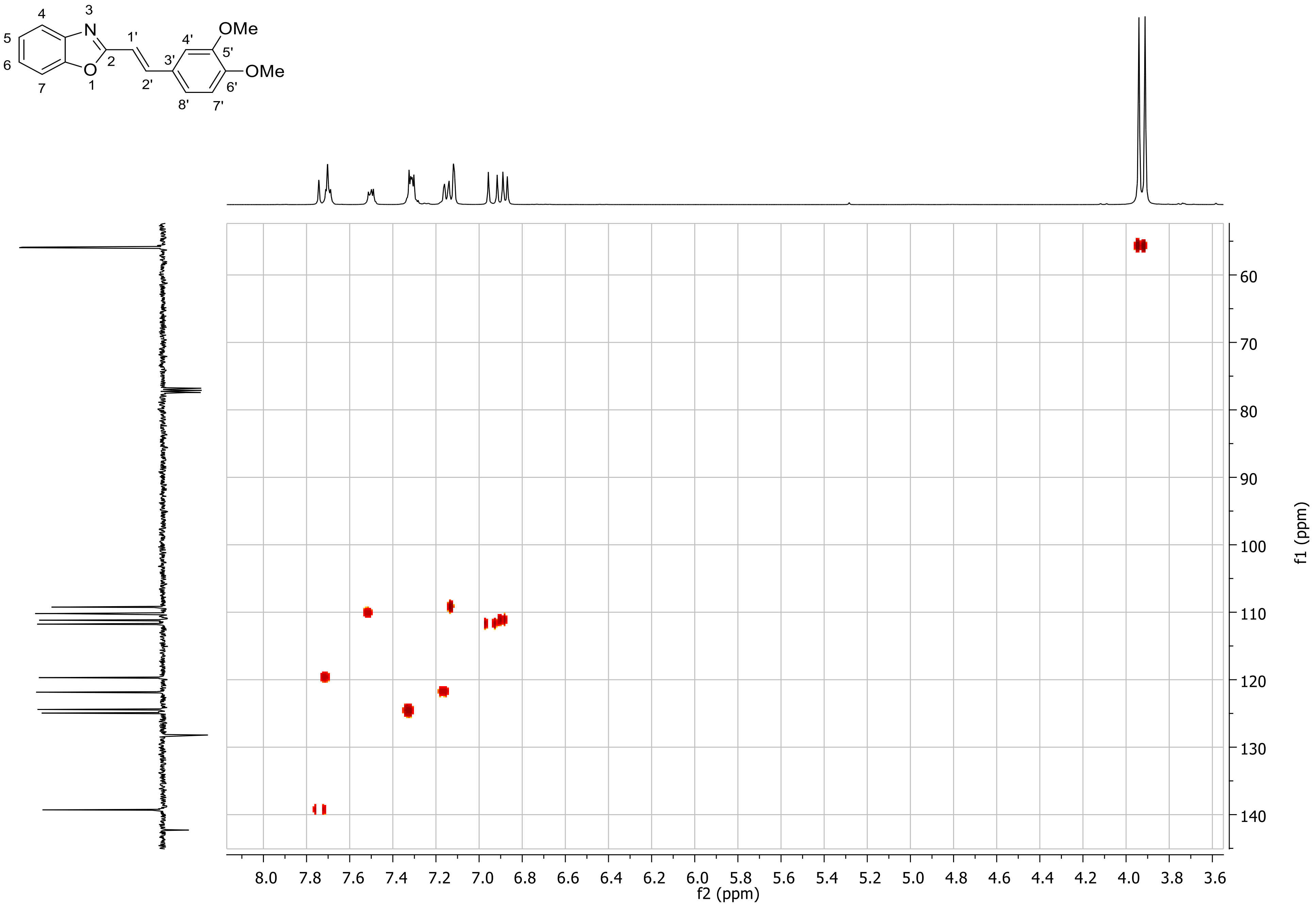

Figure S62. HSQC-spectrum of **17c** in $CDCl_3$

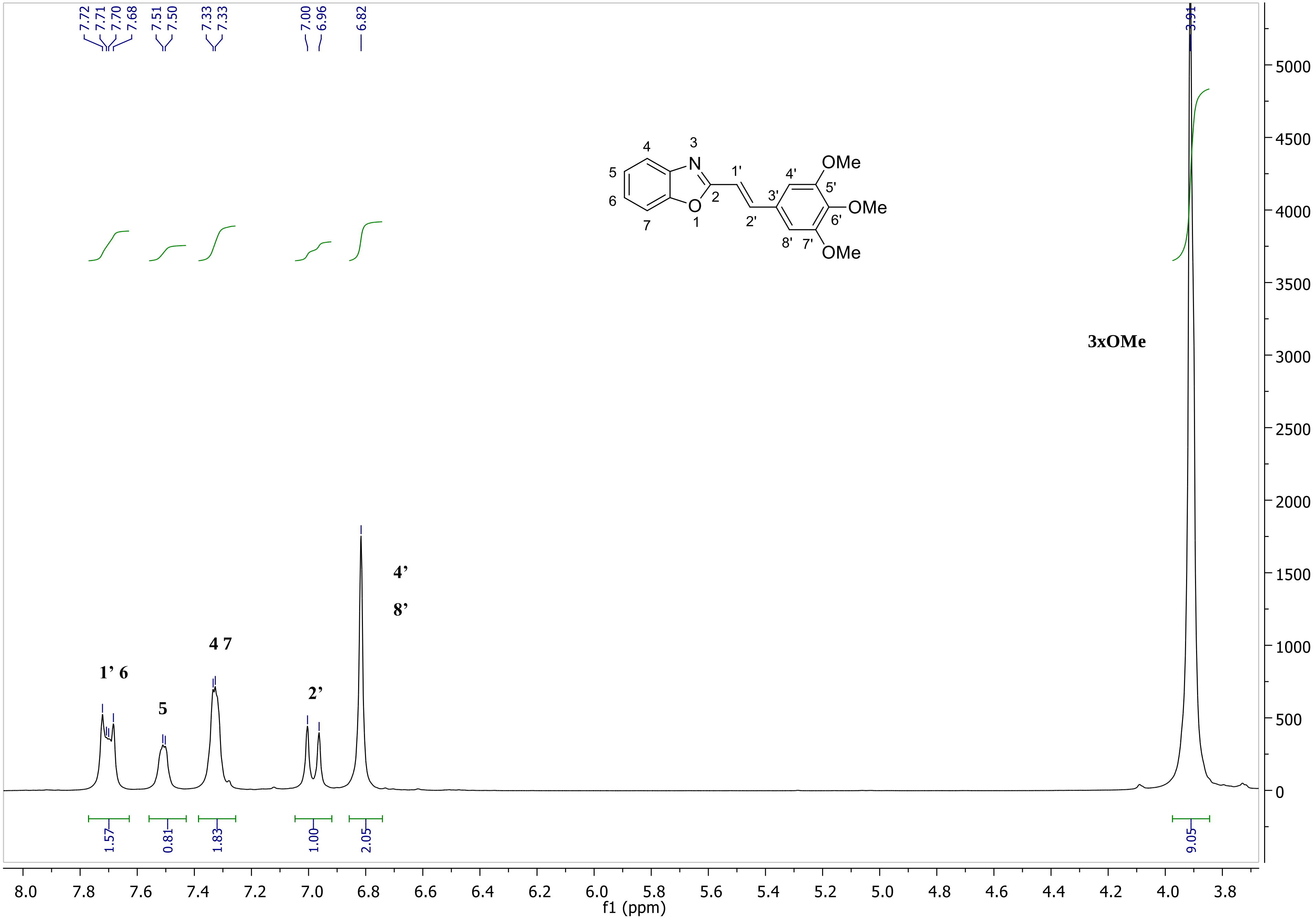

Figure S63. ^{1}H-NMR spectrum of **17e** in $CDCl_3$

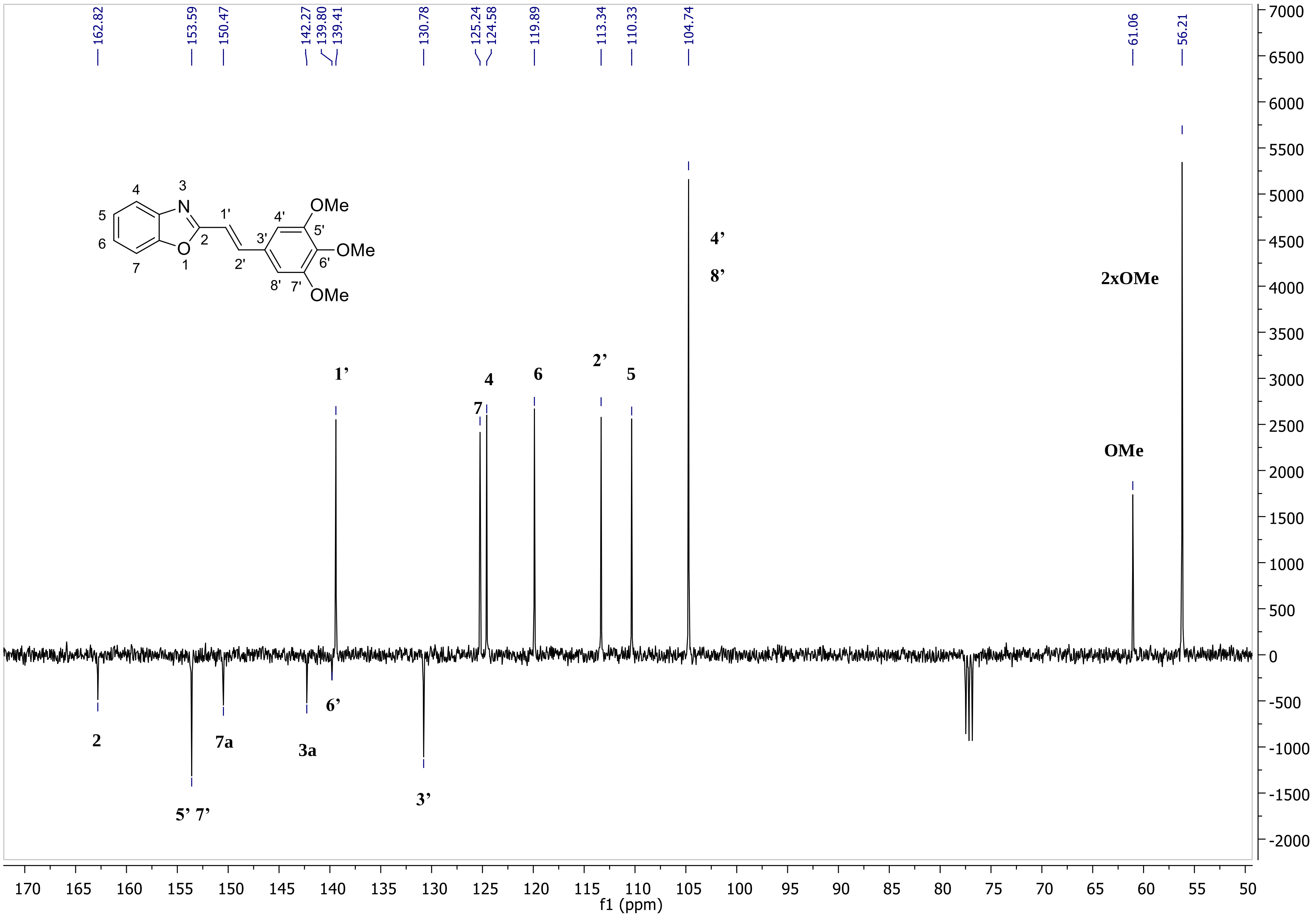

Figure S64. ^{13}C-NMR spectrum of **17e** in $CDCl_3$

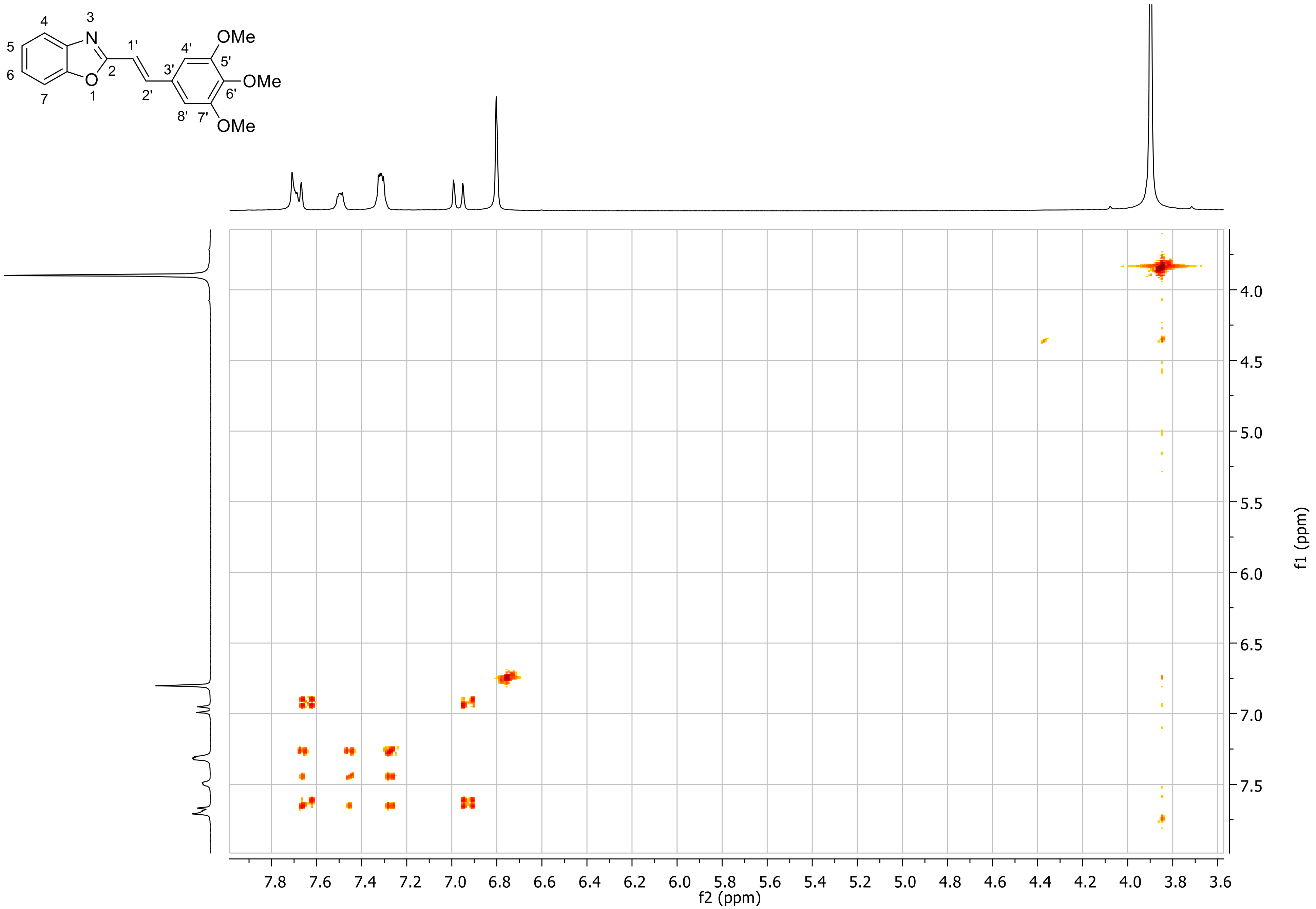

Figure S65. COSY-spectrum of **17e** in $CDCl_3$

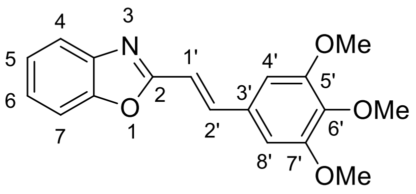

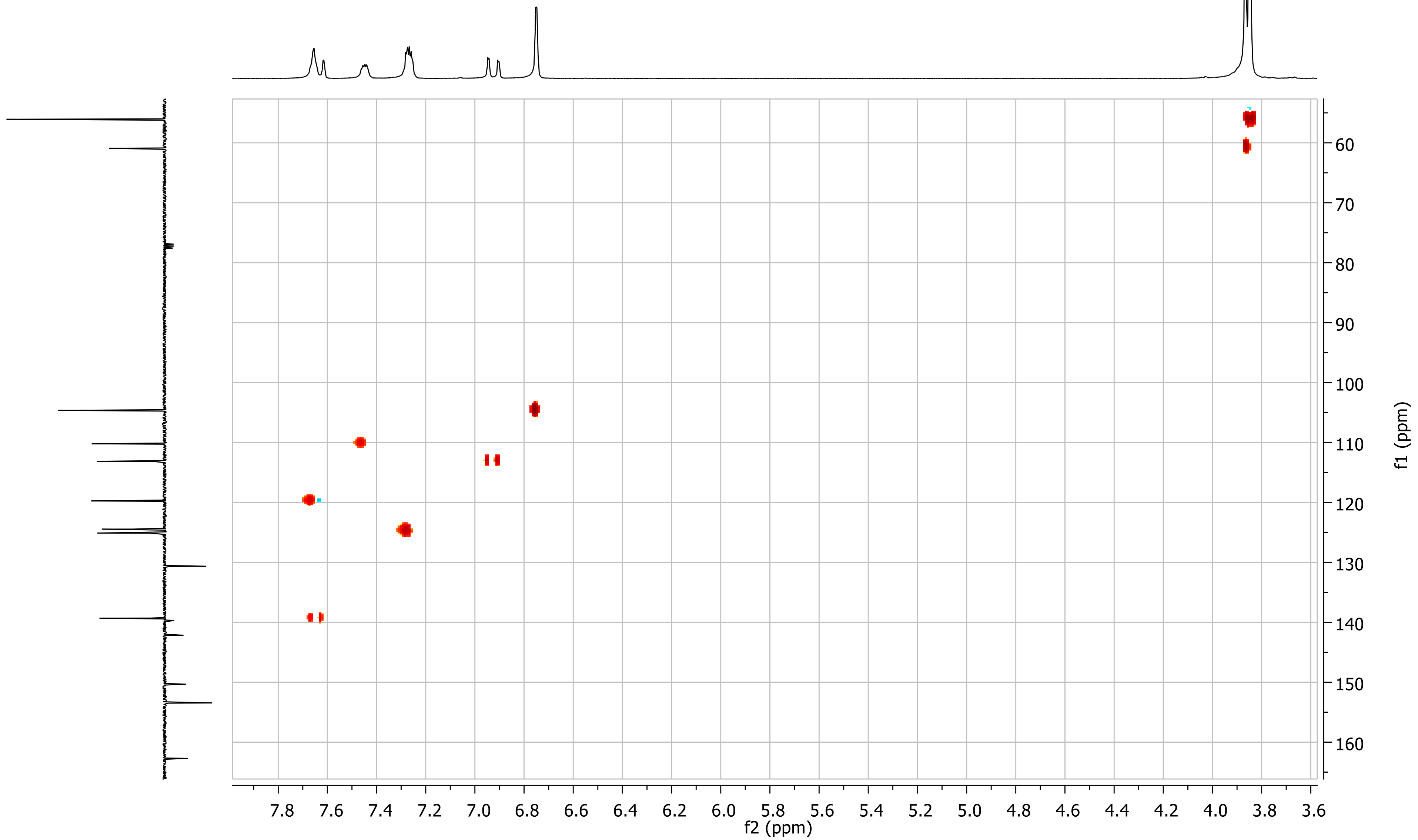

Figure S66. HSQC-spectrum of **17e** in $CDCl_3$

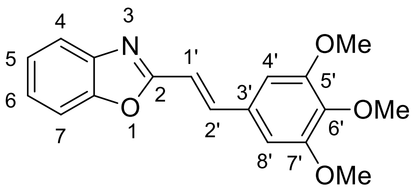

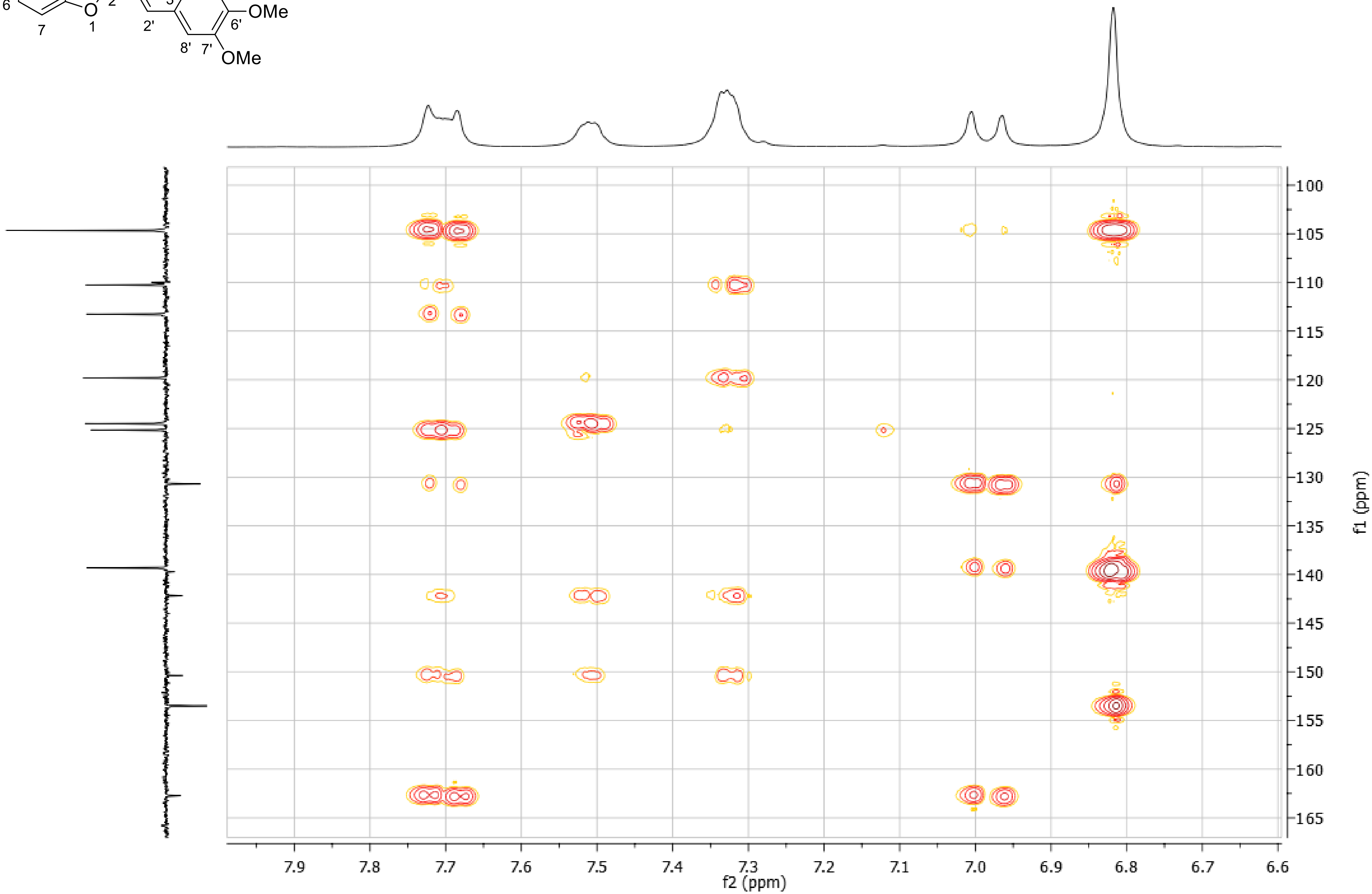

Figure S67. HMBC-spectrum of **17e** in $CDCl_3$

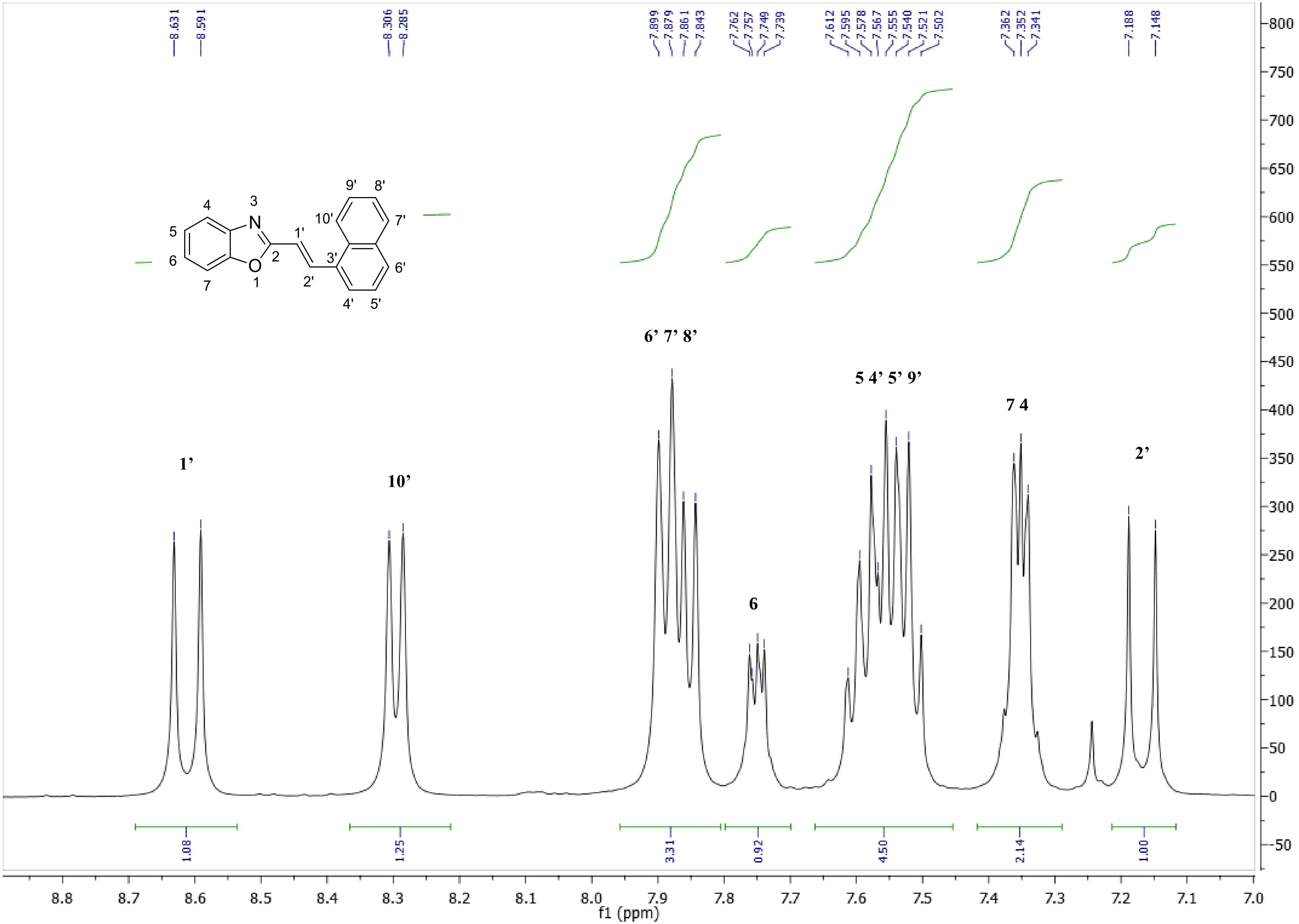

Figure S68. ^{1}H-NMR spectrum of **17f** in $CDCl_3$

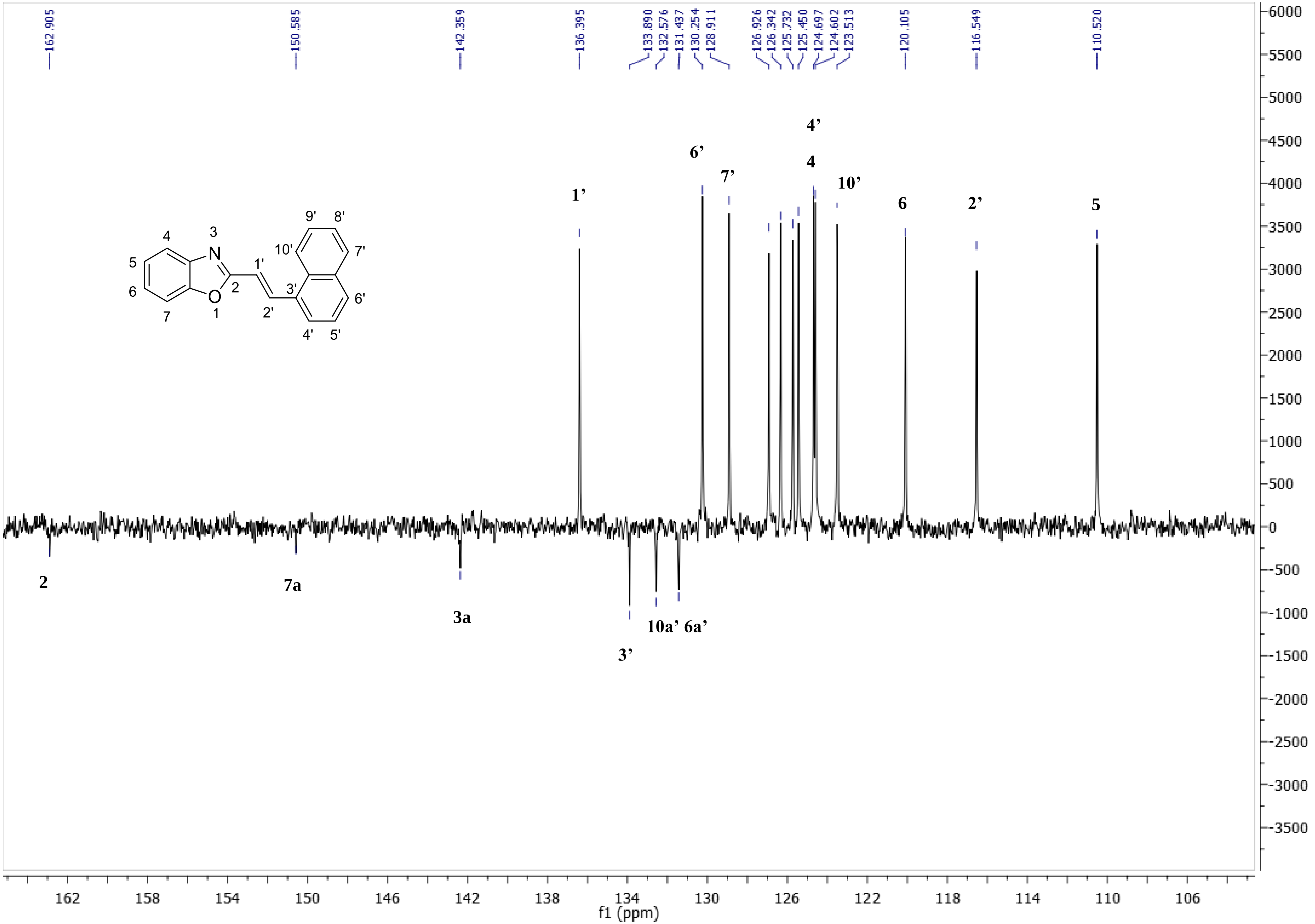

Figure S69. ^{13}C-NMR spectrum of **17f** in $CDCl_3$

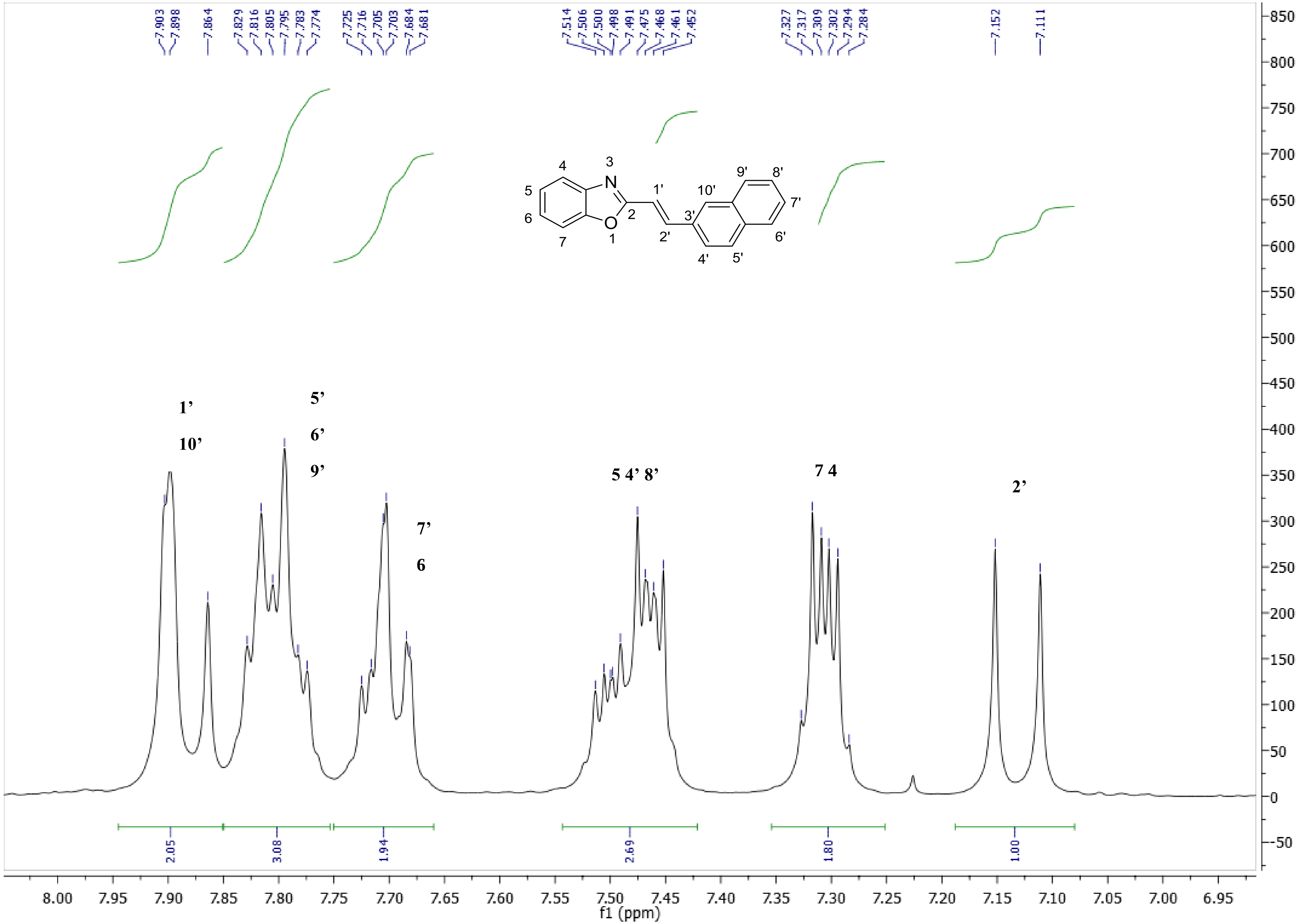

Figure S70. ^{1}H-NMR spectrum of **17g** in $CDCl_3$

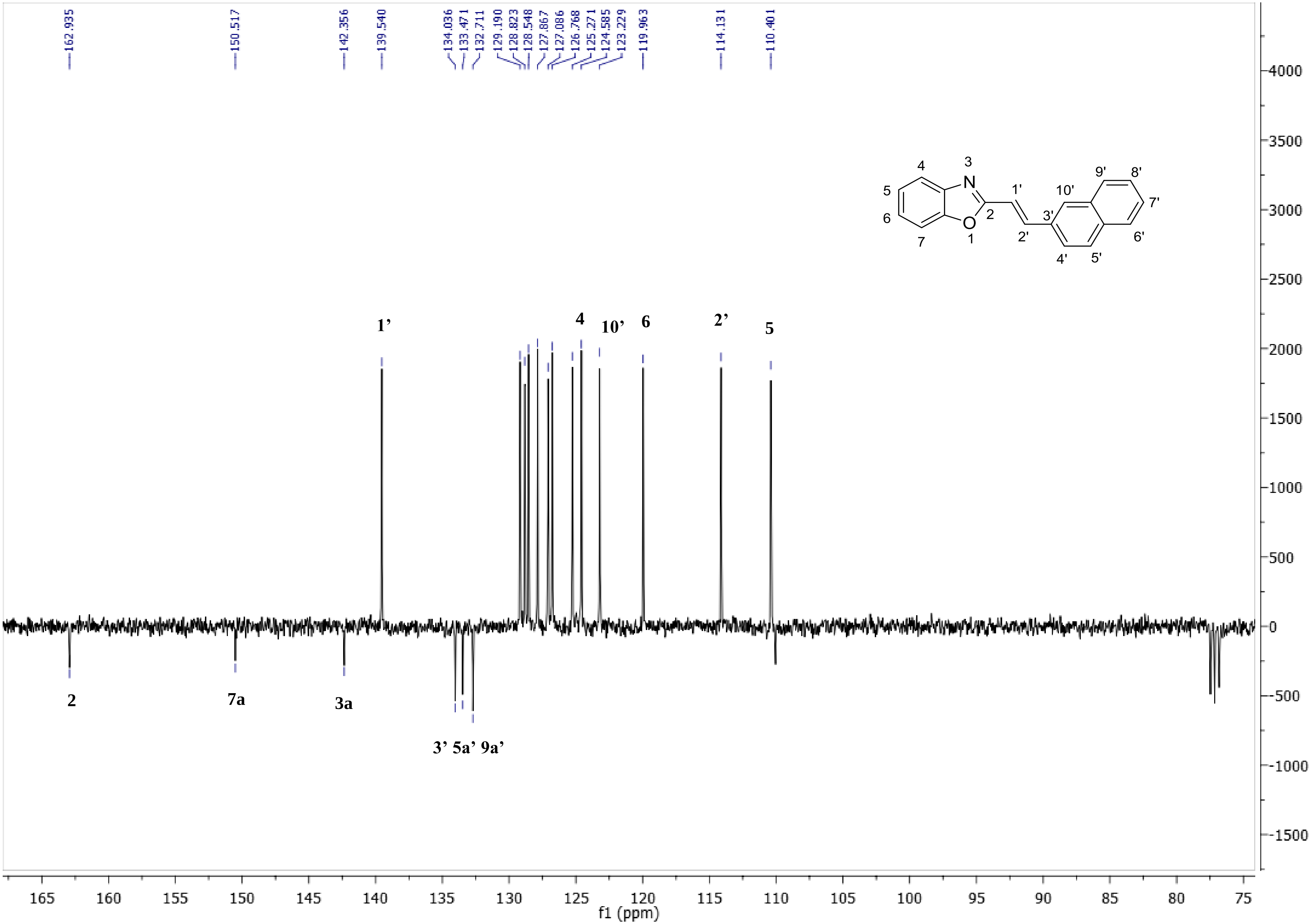

Figure S71. ^{13}C-NMR spectrum of **17g** in $CDCl_3$

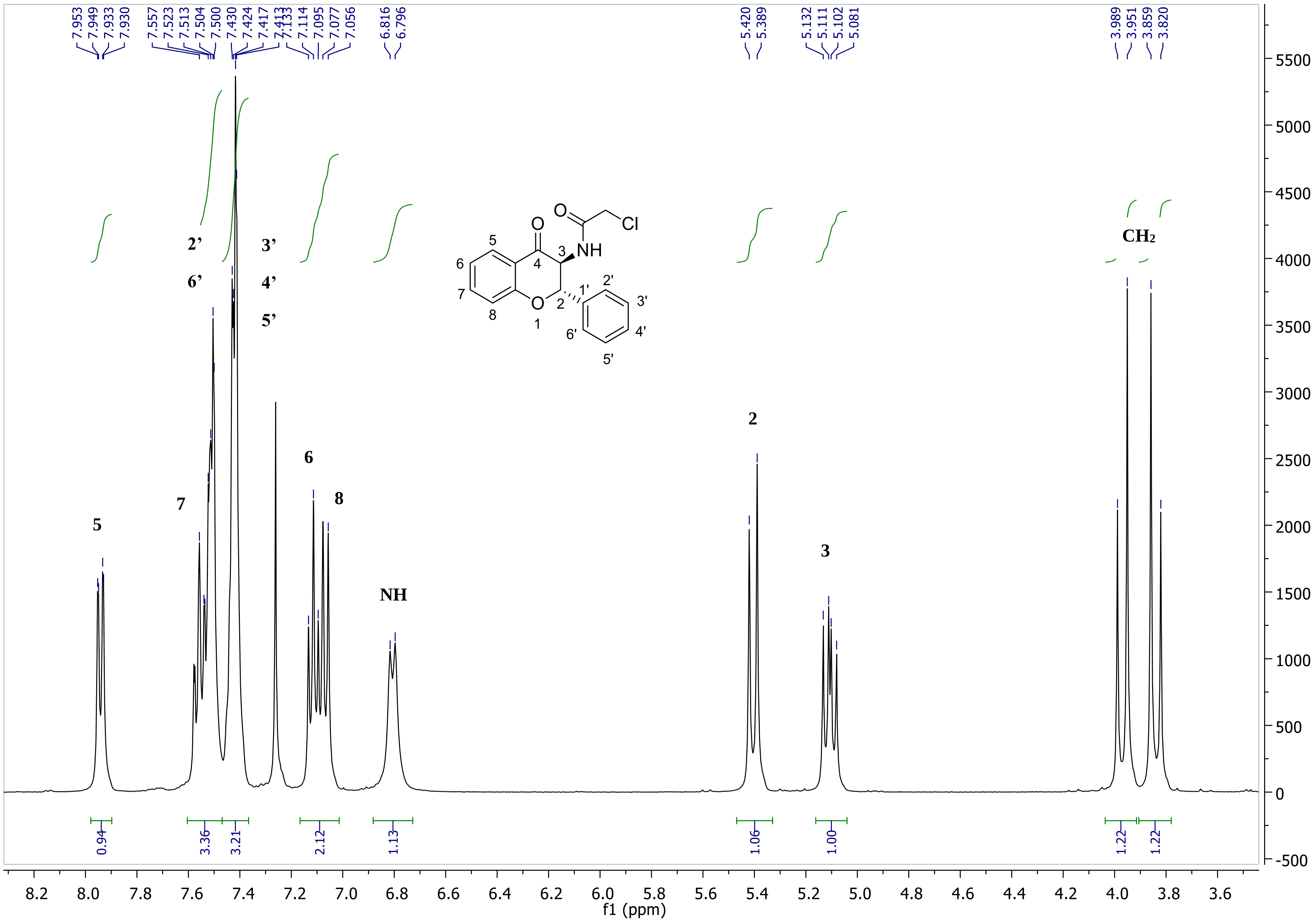

Figure S72. ^{1}H-NMR spectrum of *rac-trans*-**18a** in $CDCl_3$

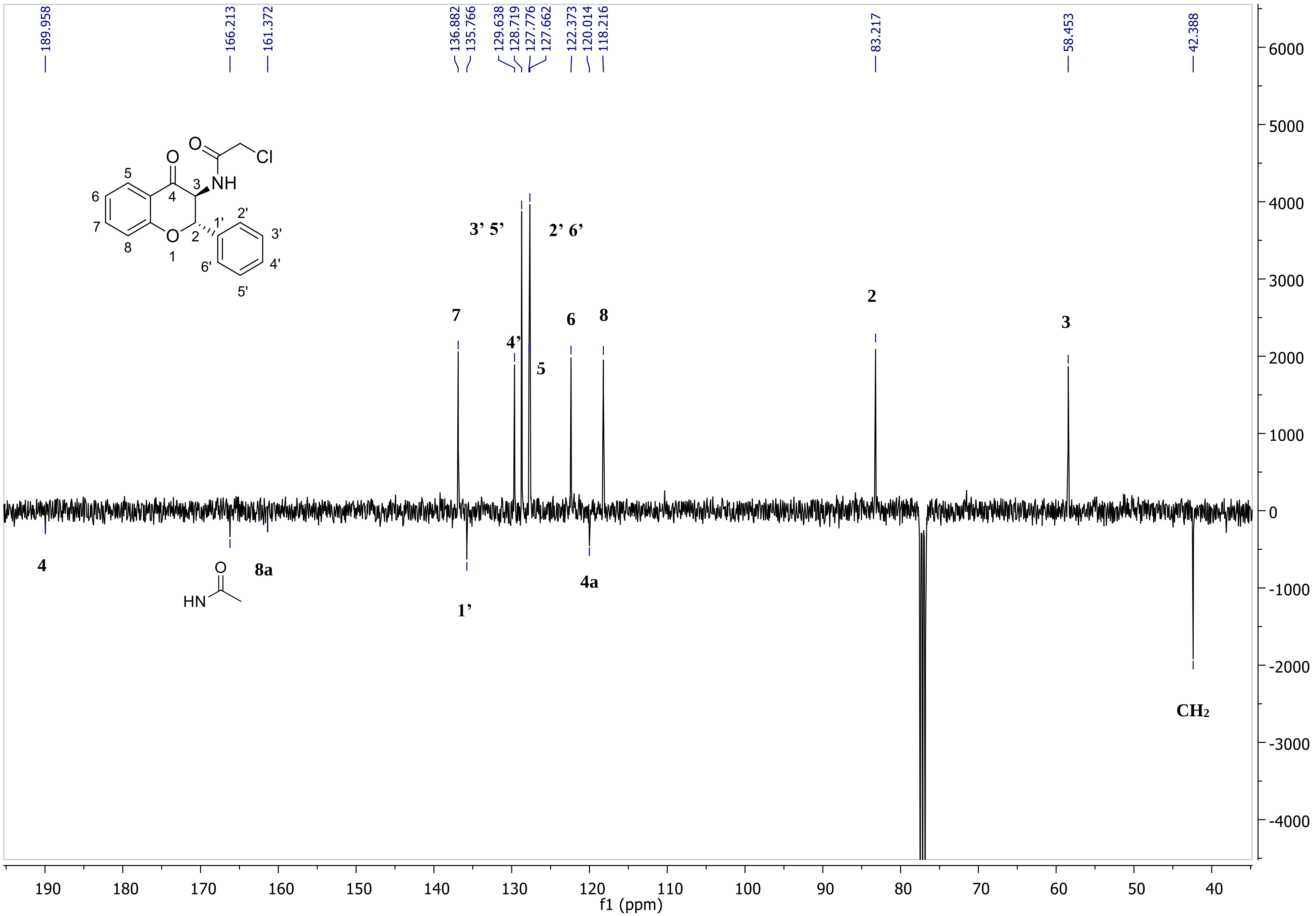

Figure S73. ^{13}C-NMR spectrum of *rac*-*trans*-**18a** in $CDCl_3$

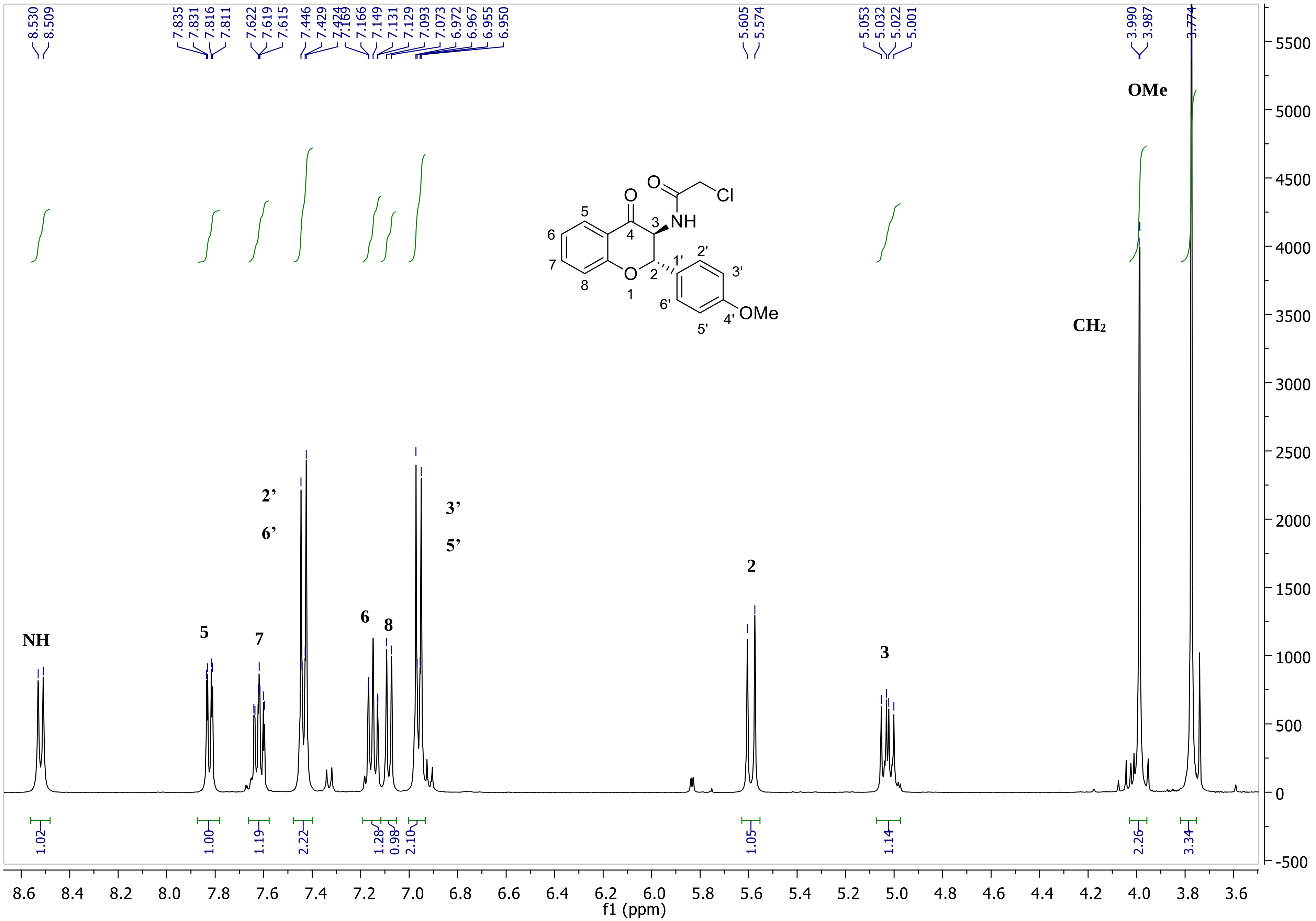

Figure S74. ^{1}H-NMR spectrum of *rac*-*trans*-**18b** in DMSO-d$_6$

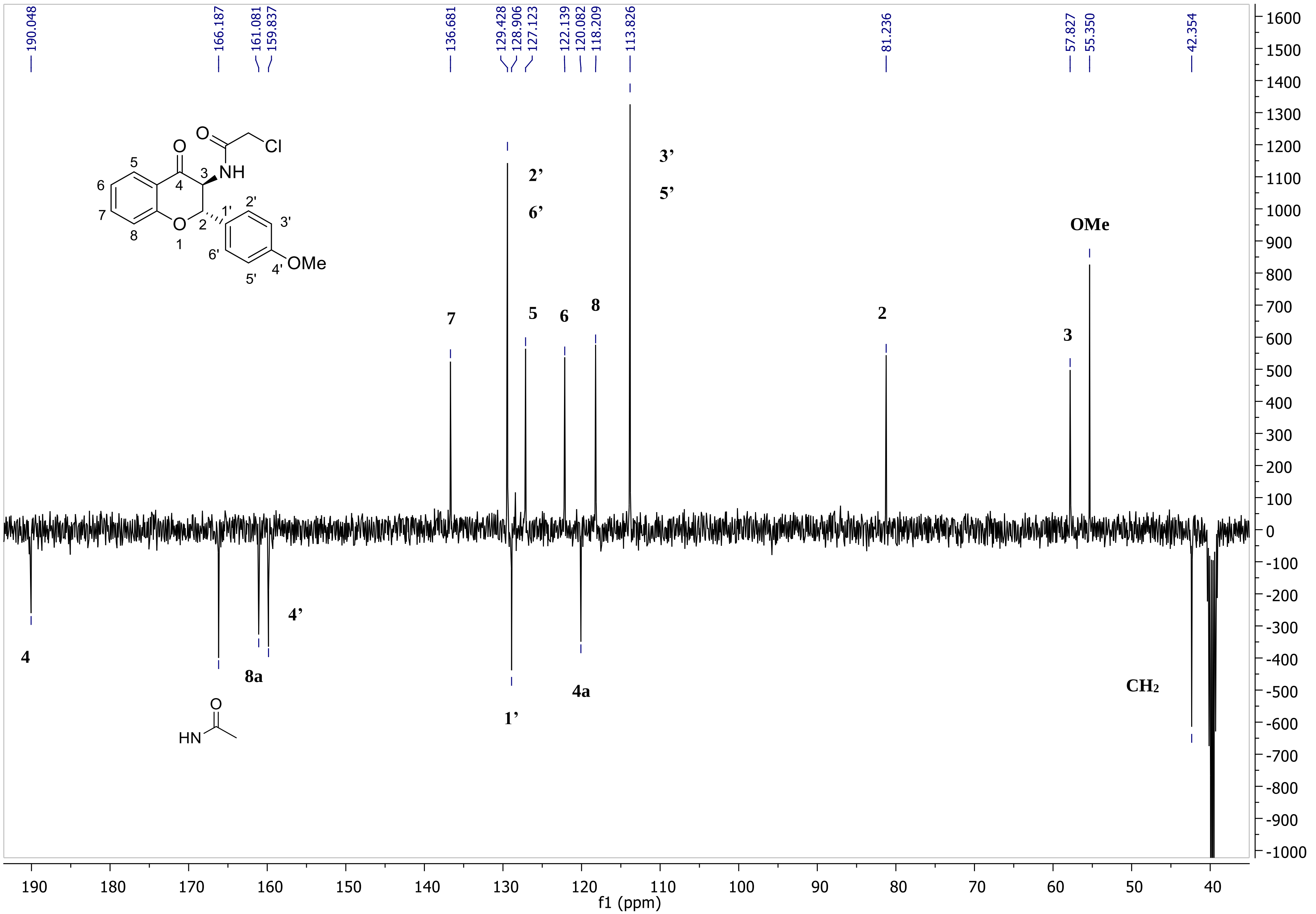

Figure S75. ^{13}C-NMR spectrum of *rac*-*trans*-**18b** in DMSO-d_6

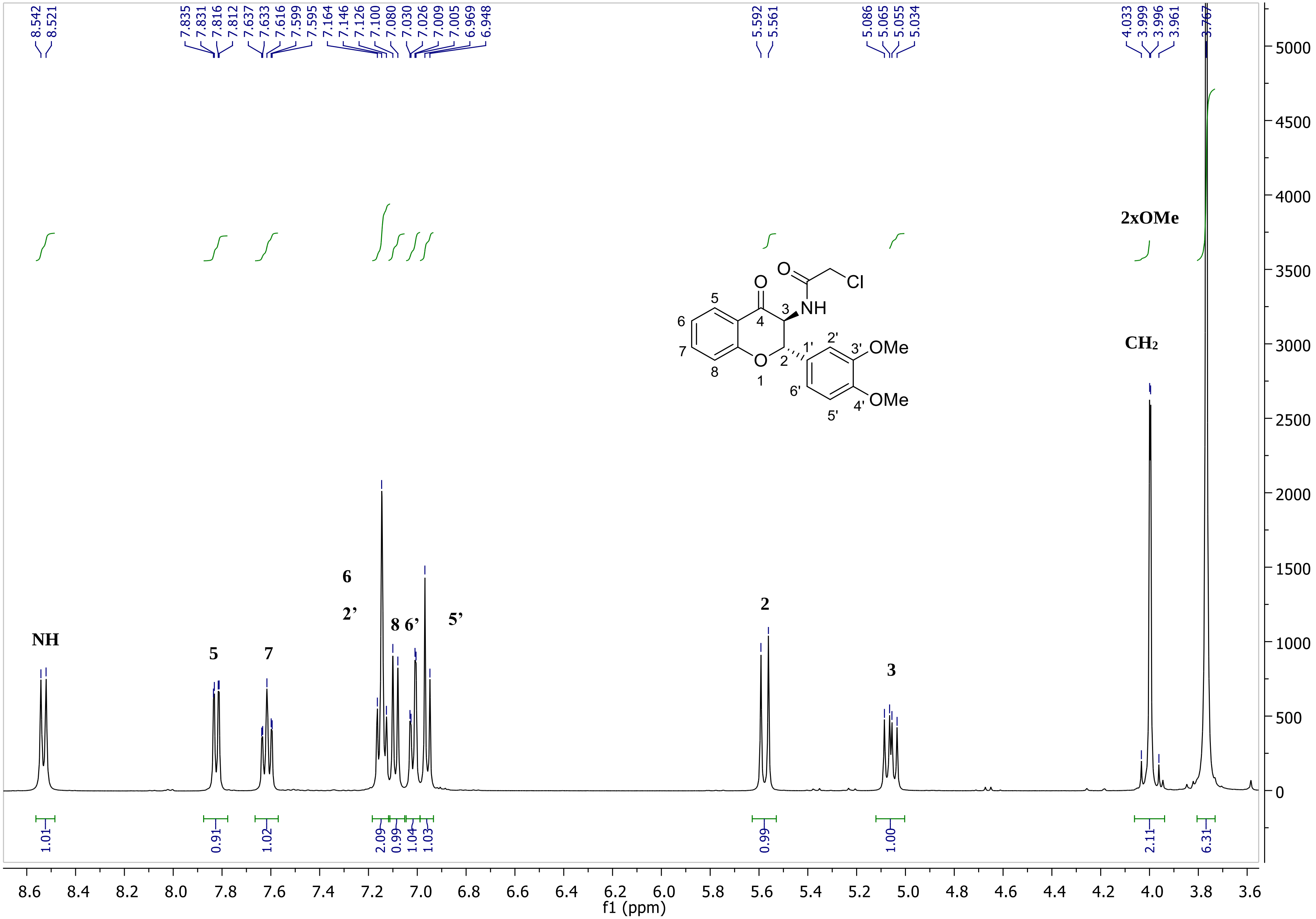

Figure S76. ^{1}H-NMR spectrum of *rac*-*trans*-**18c** in DMSO-d_6

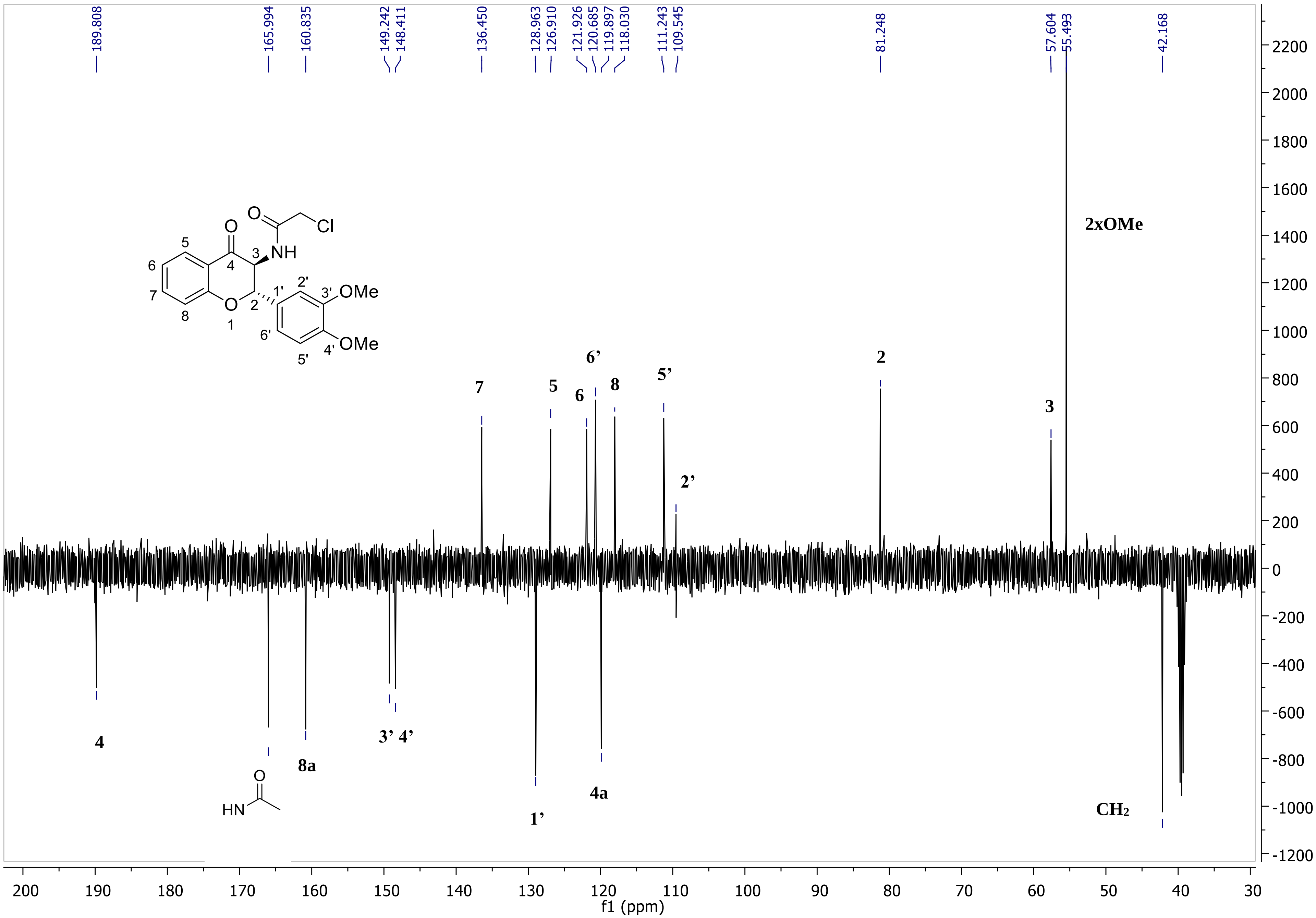

Figure S77. ^{13}C-NMR spectrum of *rac*-*trans*-**18c** in DMSO-d$_6$

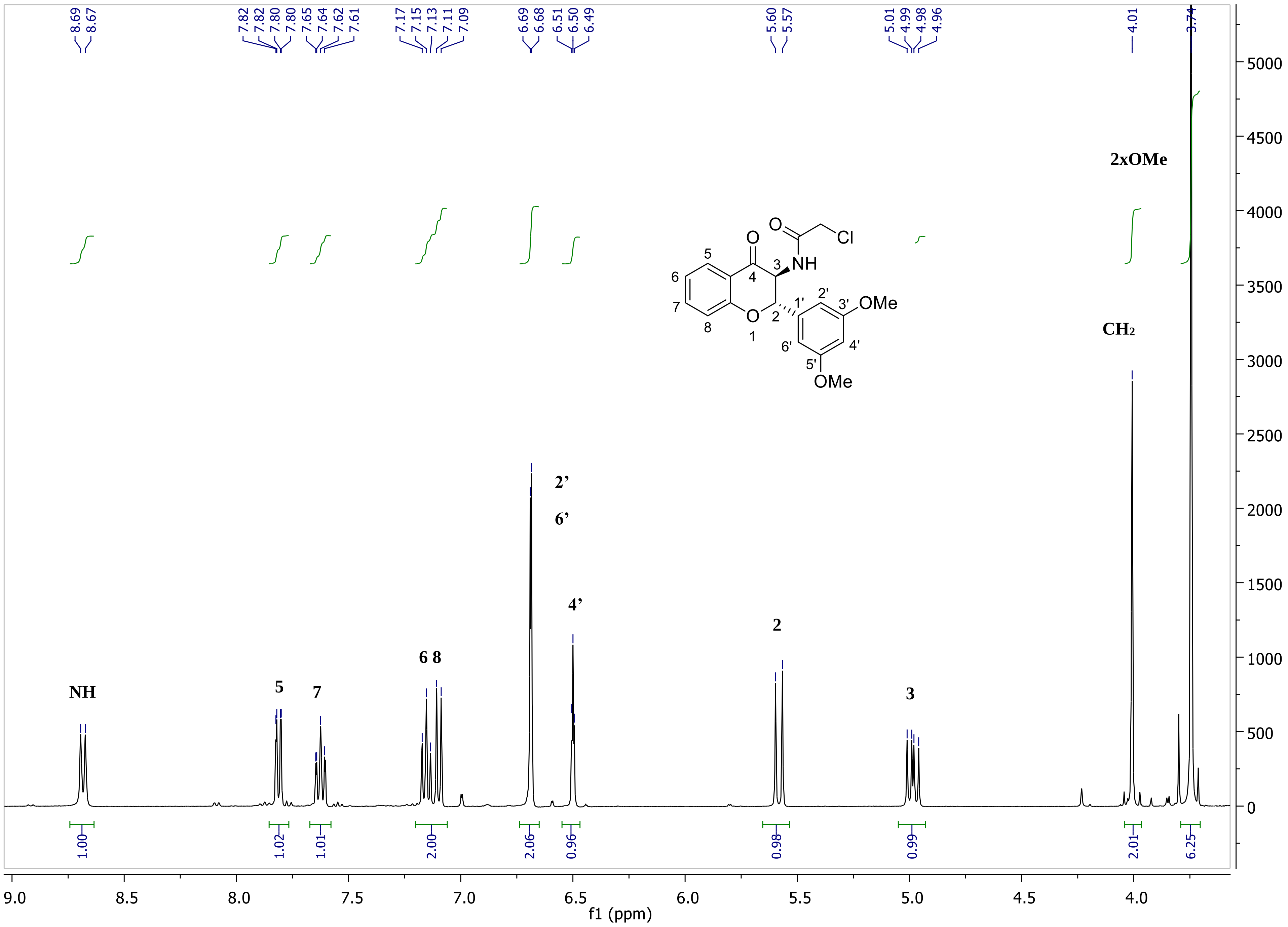

Figure S78. ^{1}H-NMR spectrum of *rac-trans*-**18d** in DMSO-d$_6$

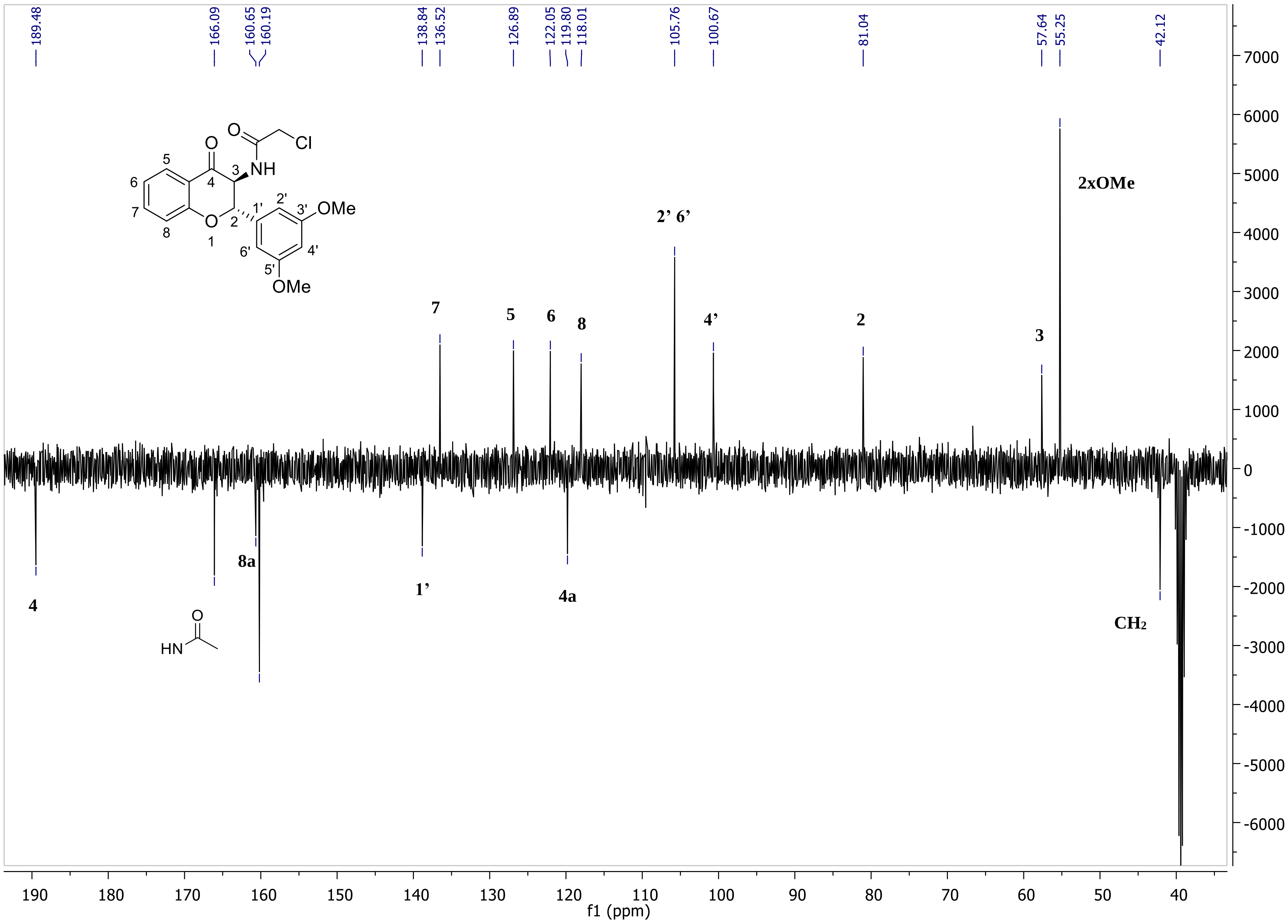

Figure S79. ^{13}C-NMR spectrum of *rac*-*trans*-**18d** in DMSO-d_6

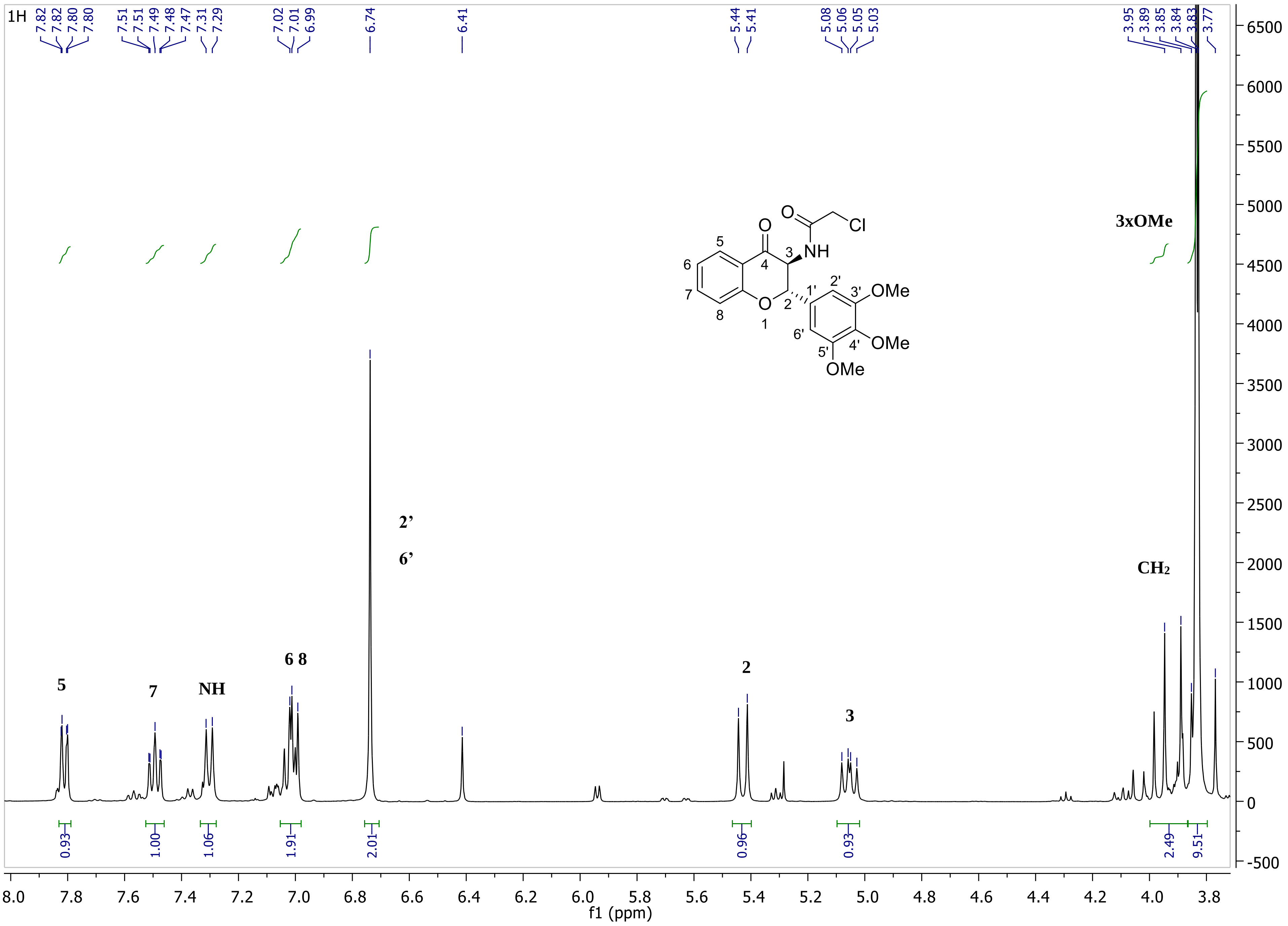

Figure S80. ^{1}H-NMR spectrum of *rac-trans*-**18e** in $CDCl_3$

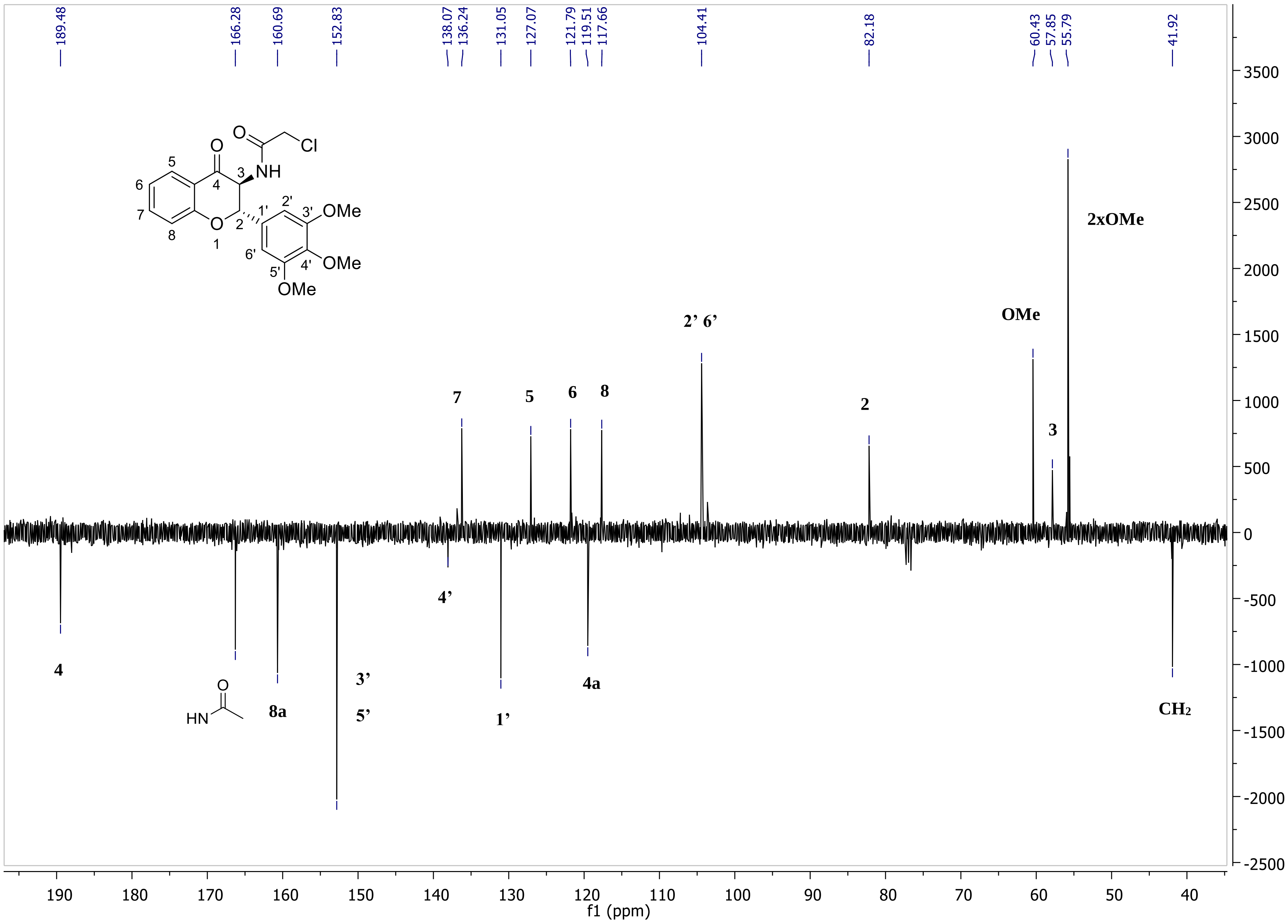

Figure S81. ^{13}C-NMR spectrum of *rac*-*trans*-**18e** in $CDCl_3$

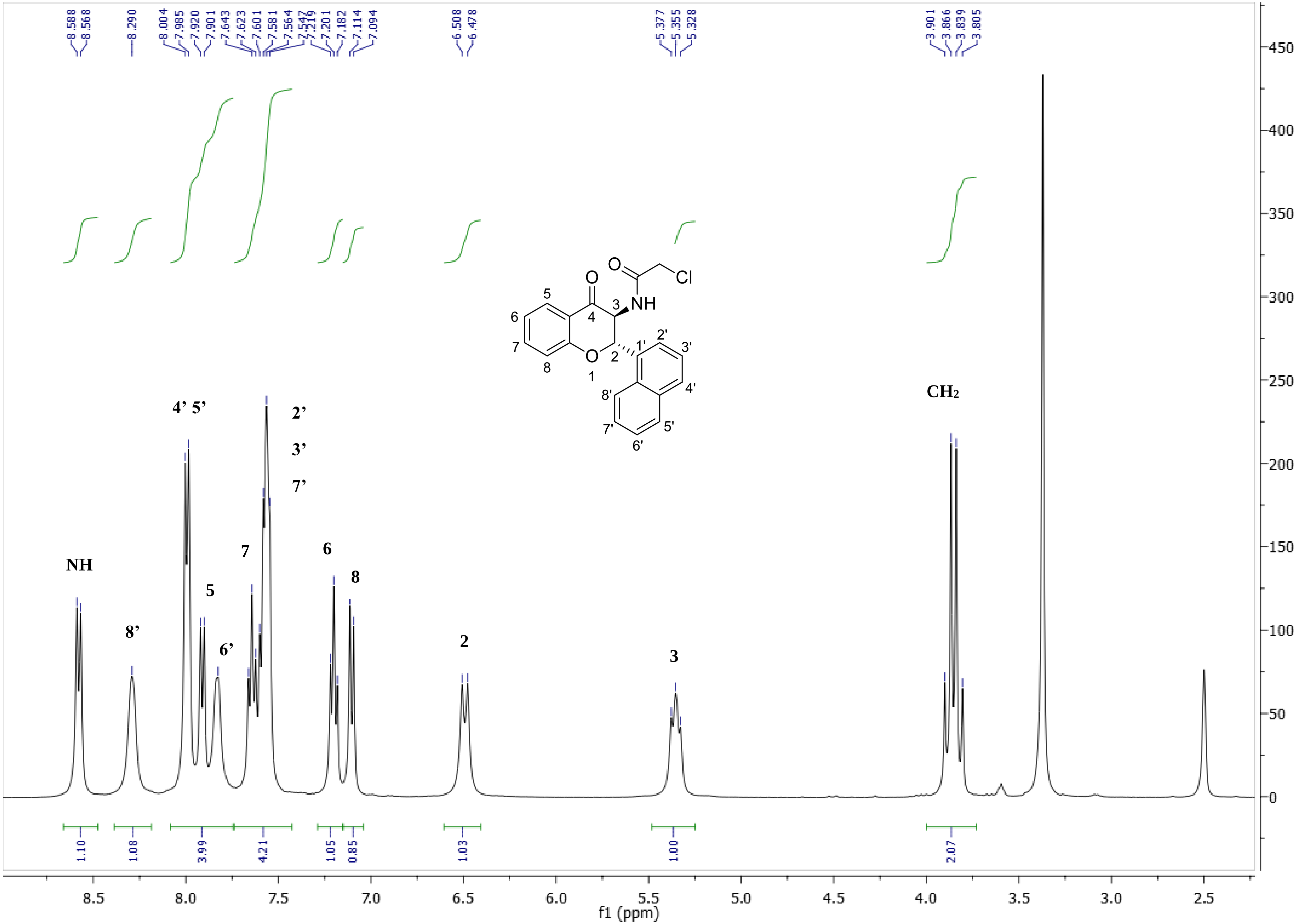

Figure S82. ^{1}H-NMR spectrum of *rac-trans*-**18f** in DMSO-d_6

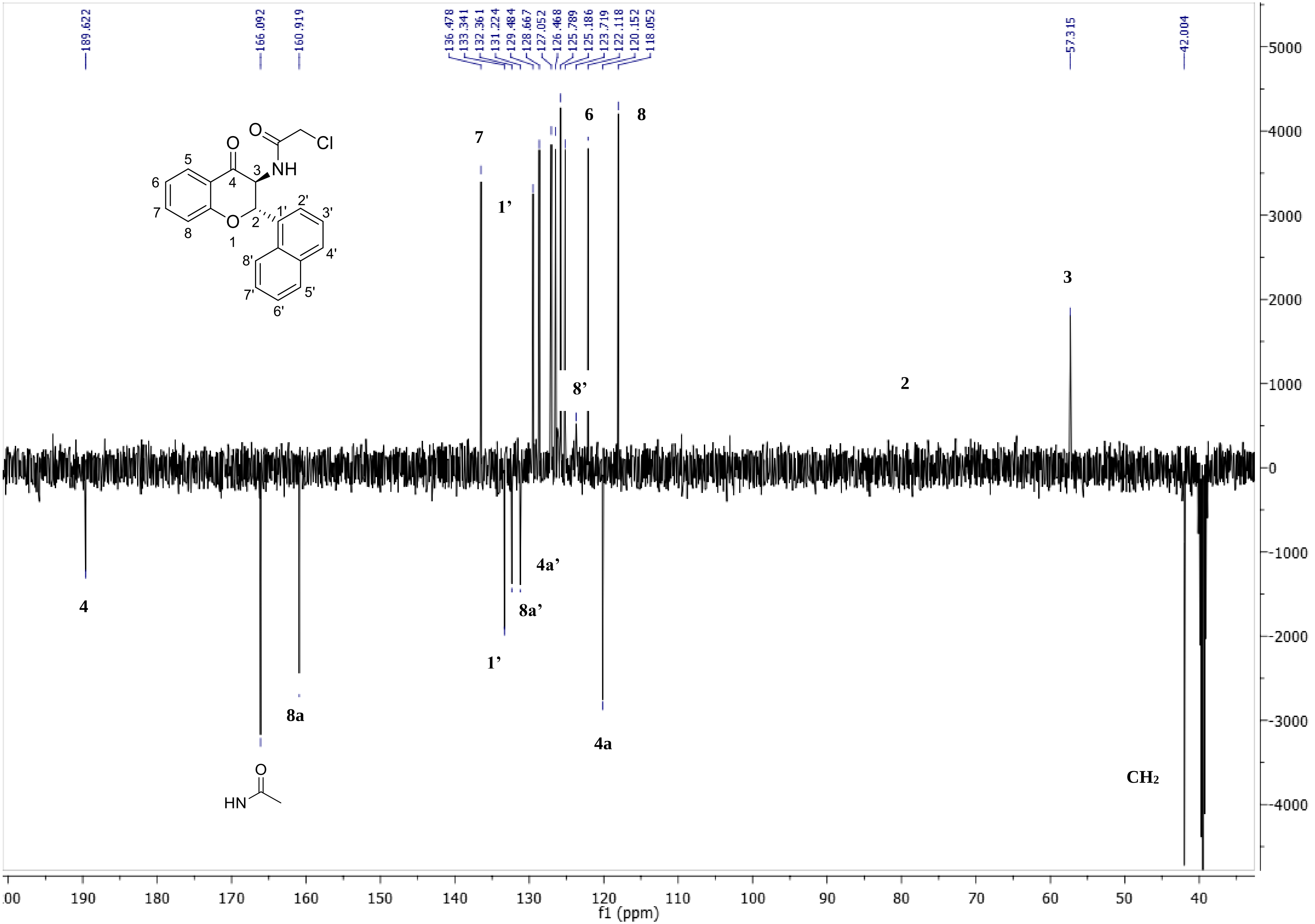

Figure S83. ^{13}C-NMR spectrum of *rac*-*trans*-**18f** in $CDCl_3$

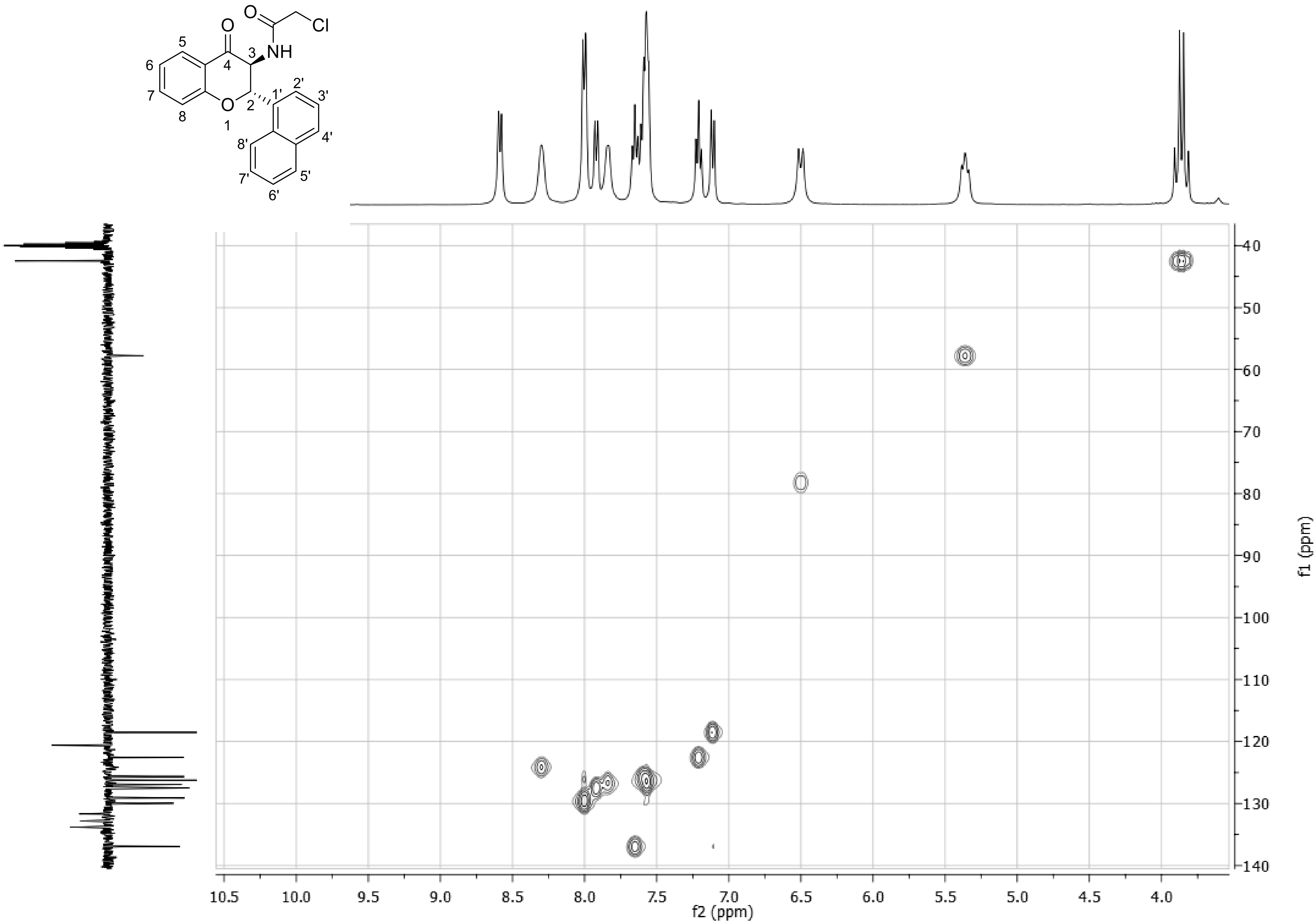

Figure S84. HSQC-spectrum of *rac-trans*-**18f** in DMSO-d_6

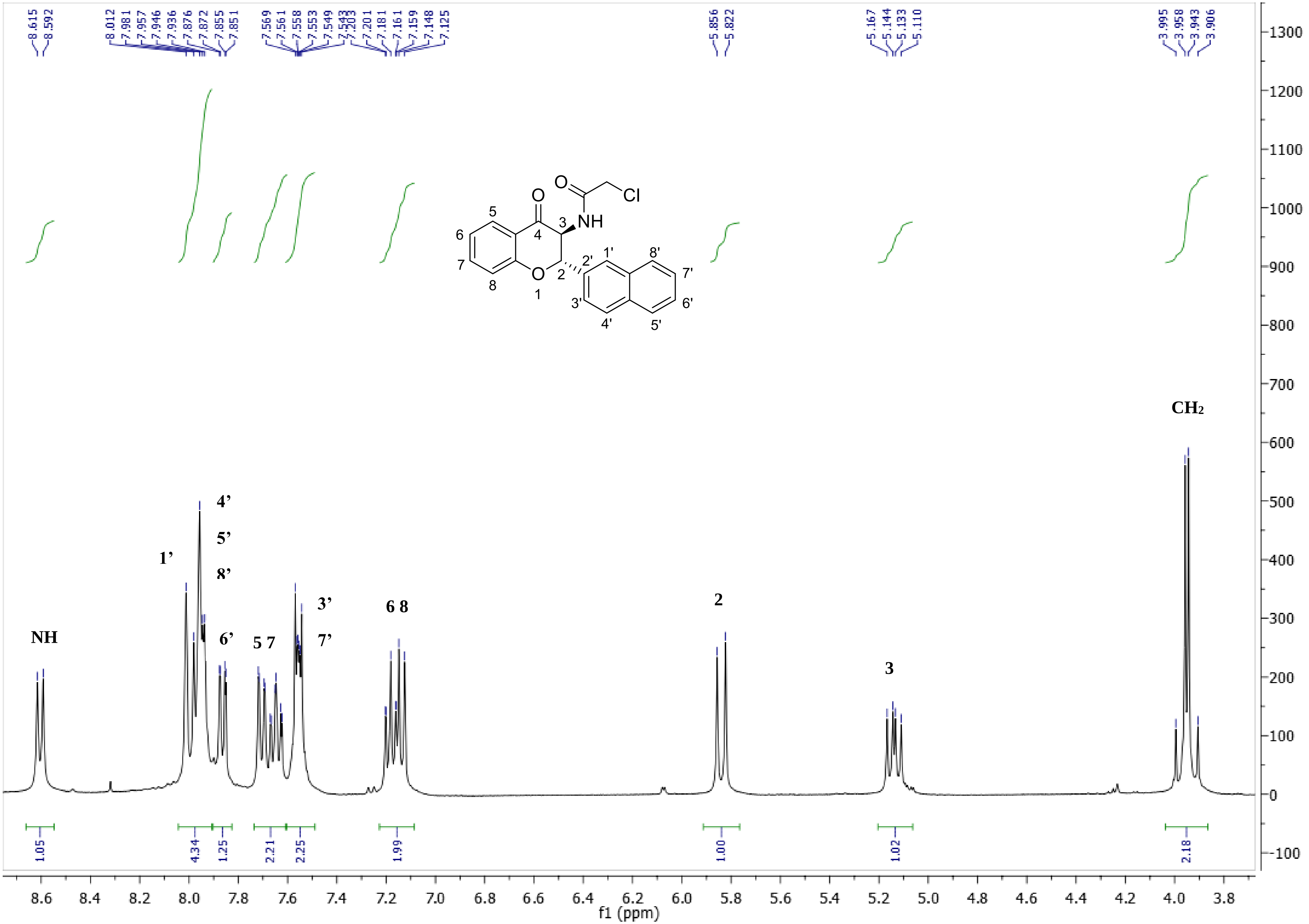

Figure S85. ^{1}H-NMR spectrum of *rac-trans*-**18g** in DMSO-d_6

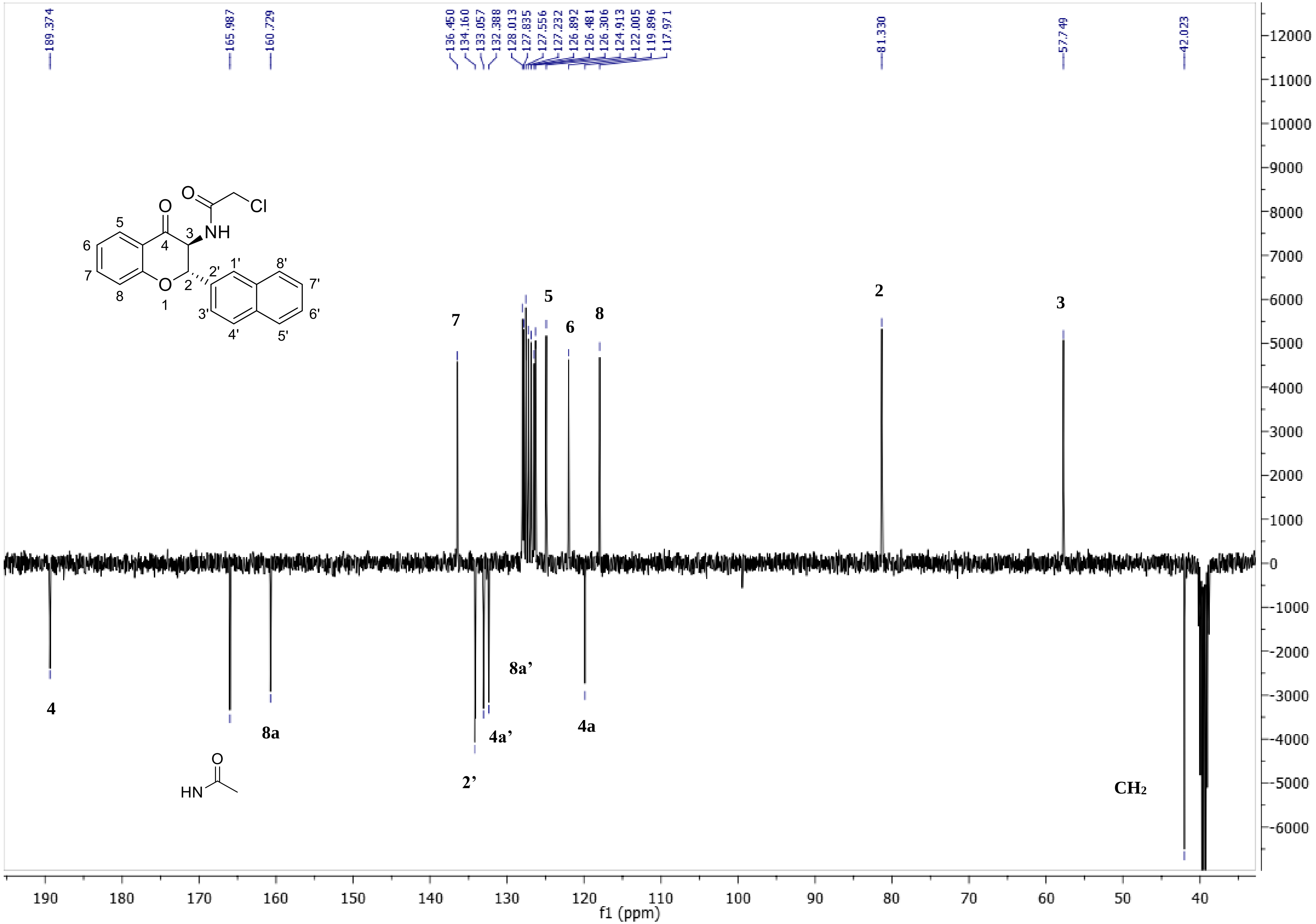

Figure S86. ^{13}C-NMR spectrum of *rac*-*trans*-**18g** in $CDCl_3$

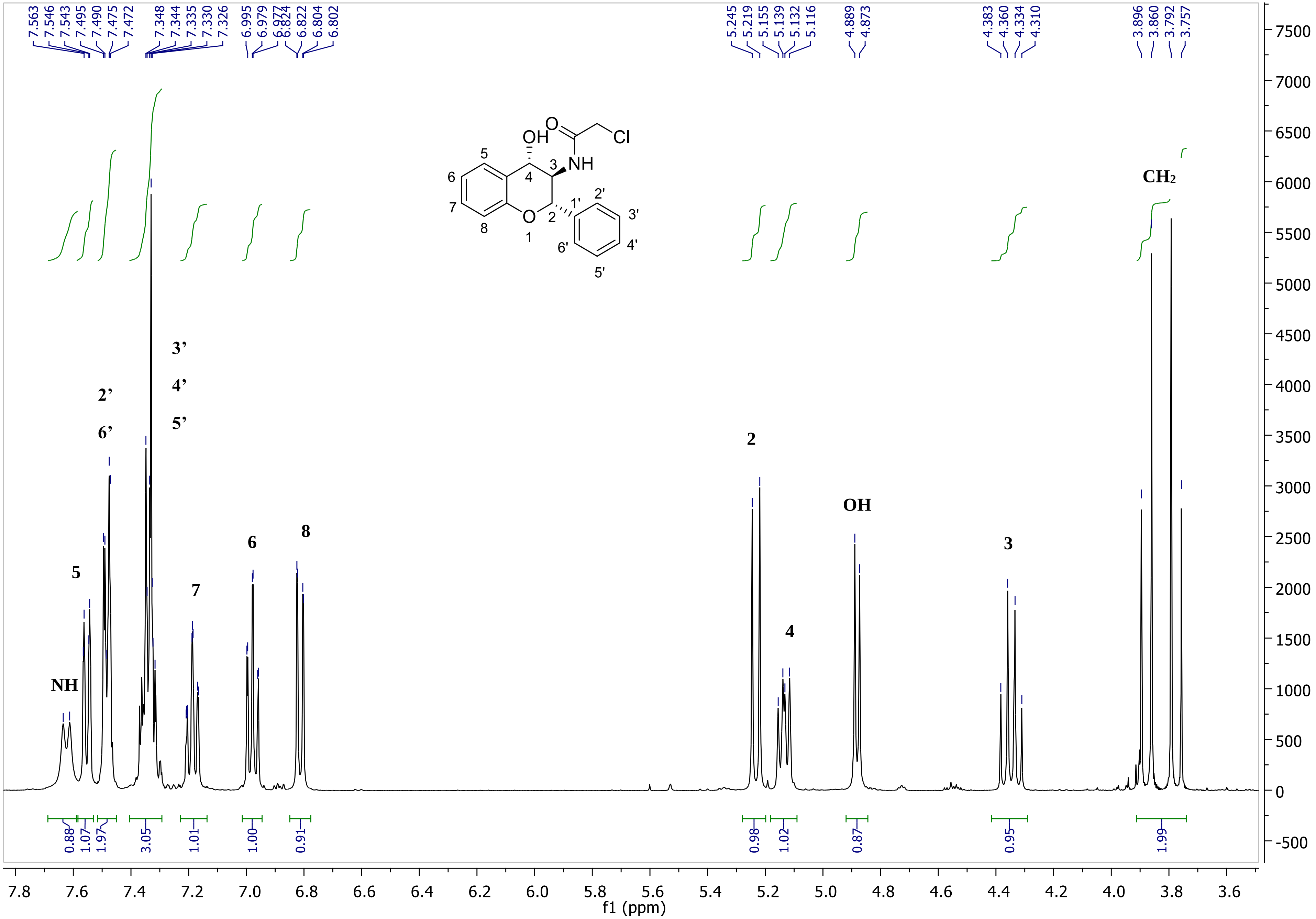

Figure S87. ^{1}H-NMR spectrum of *rac*-**19a** in Acetone-d_6

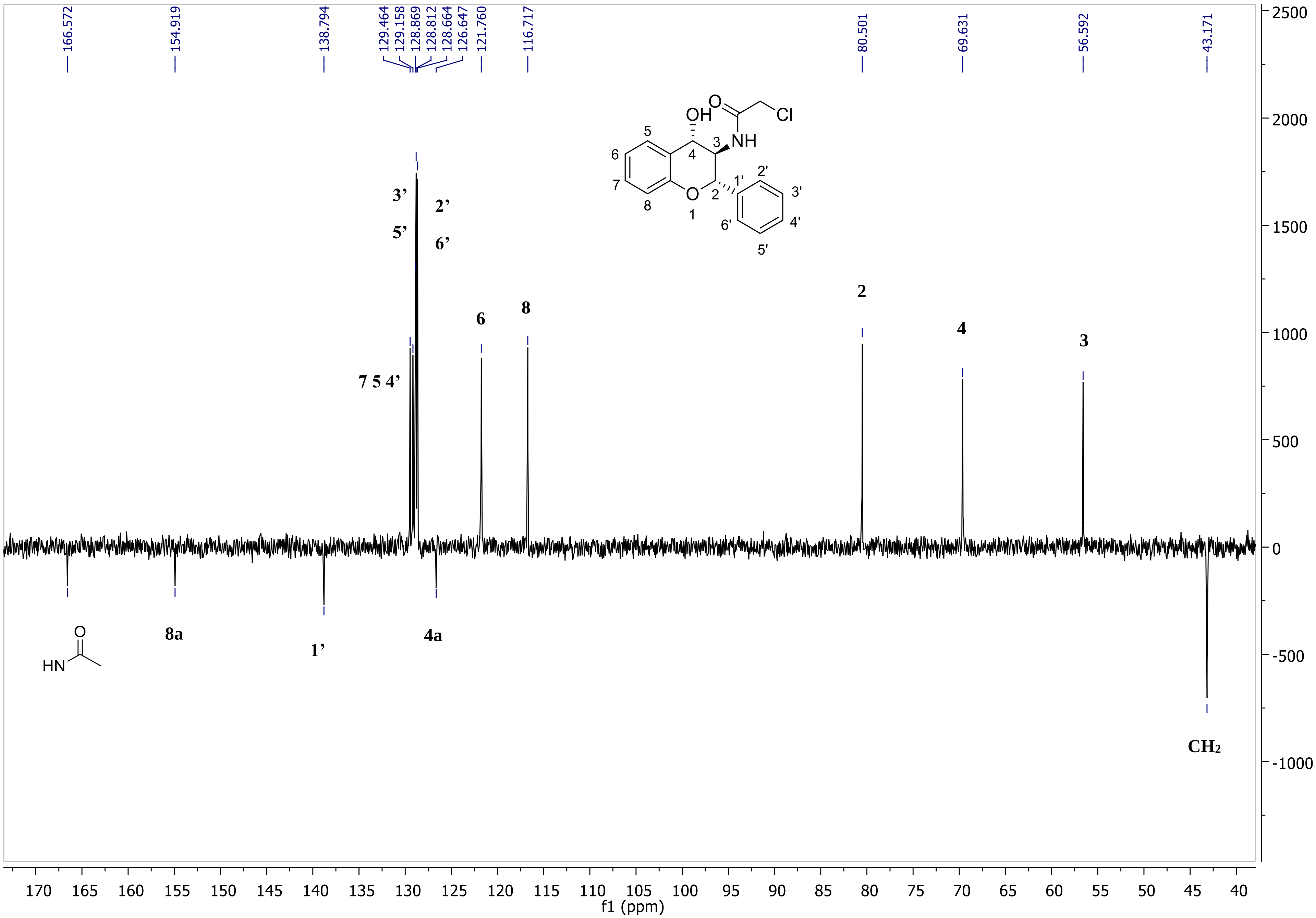

Figure S88. ^{13}C-NMR spectrum of *rac*-**19a** in Acetone-d$_6$

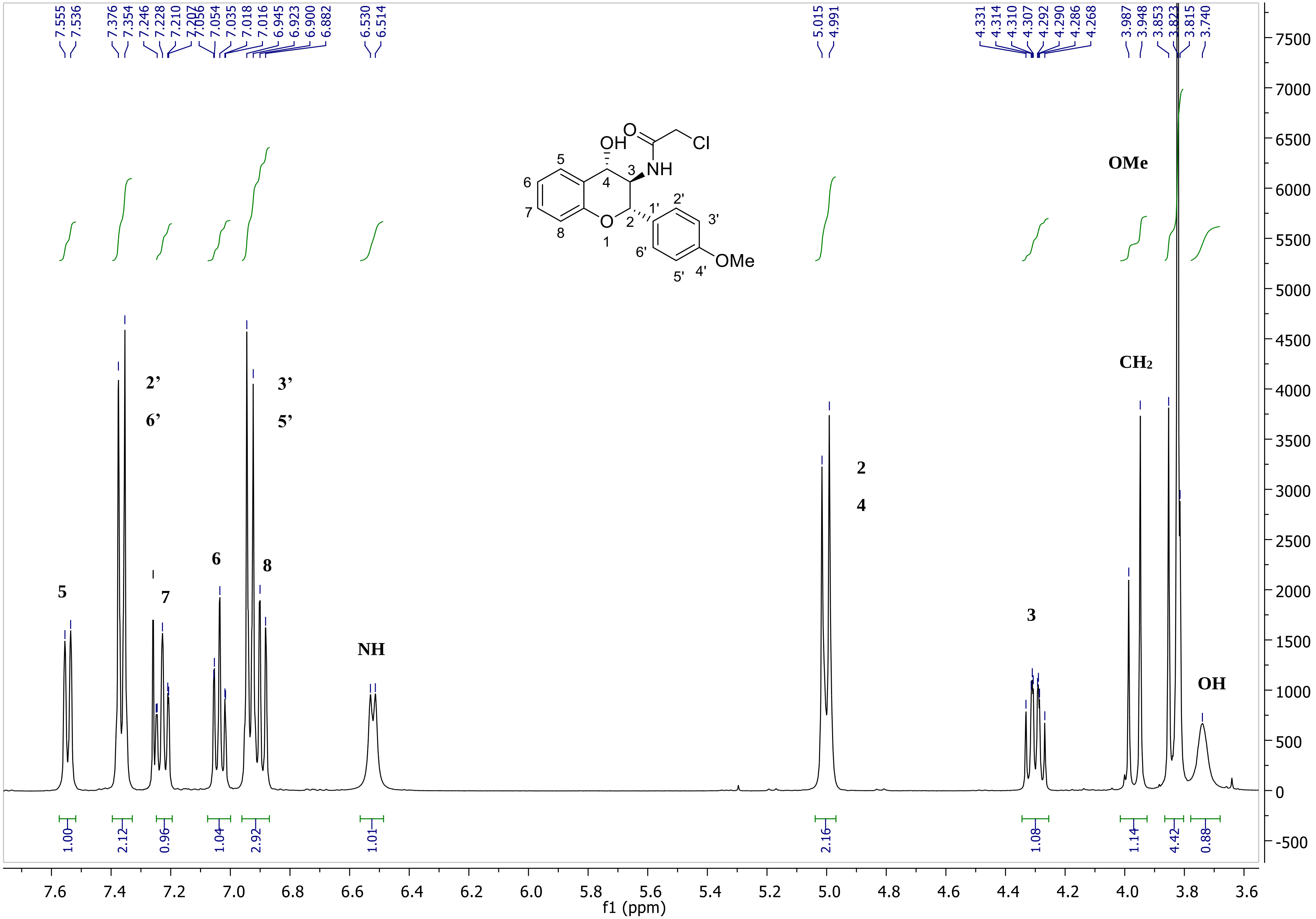

Figure S89. ^{1}H-NMR spectrum of *rac*-**19b** in $CDCl_3$

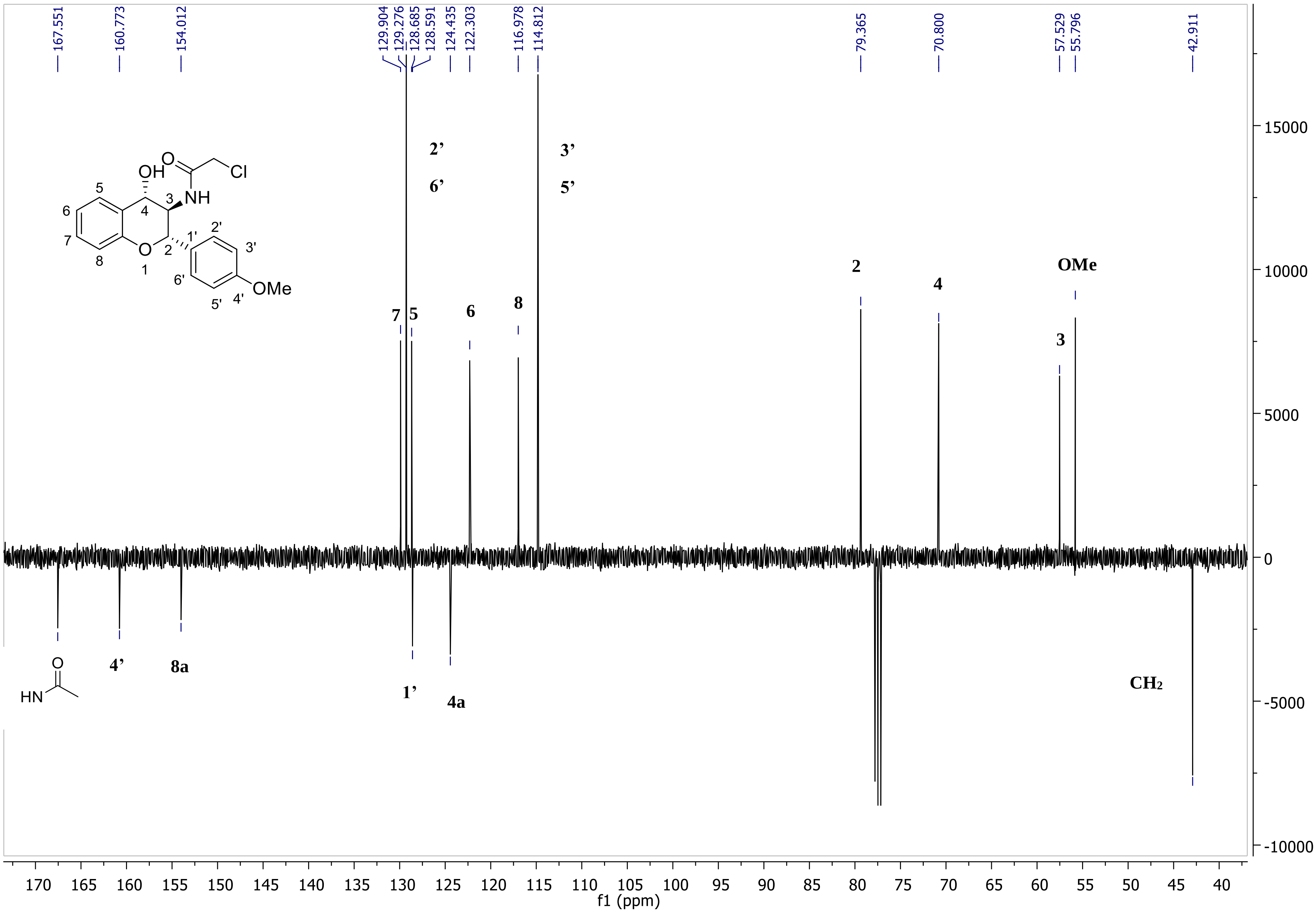

Figure S90. ^{13}C-NMR spectrum of *rac*-**19b** in $CDCl_3$

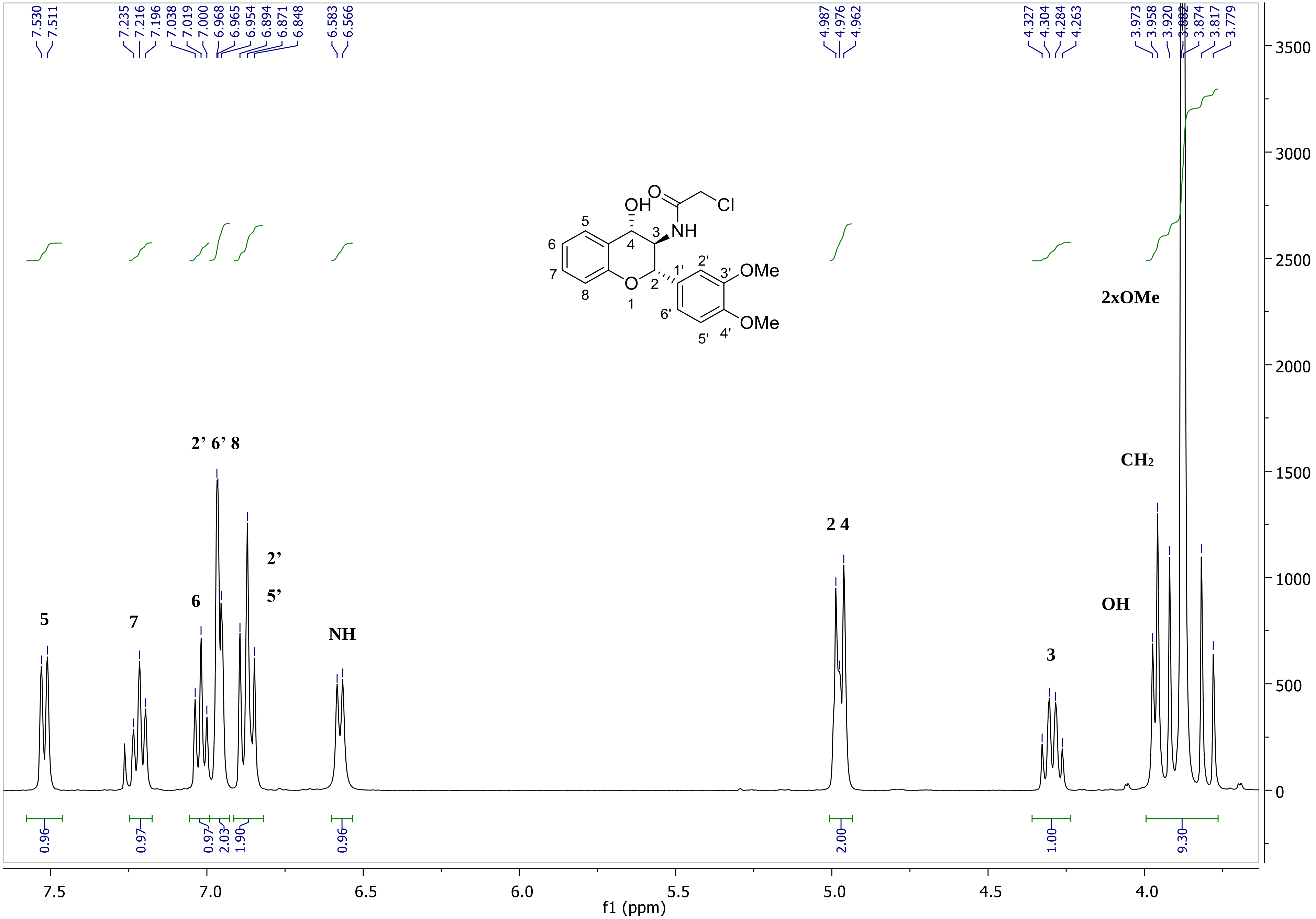

Figure S91. ^{1}H-NMR spectrum of *rac*-**19c** in $CDCl_3$

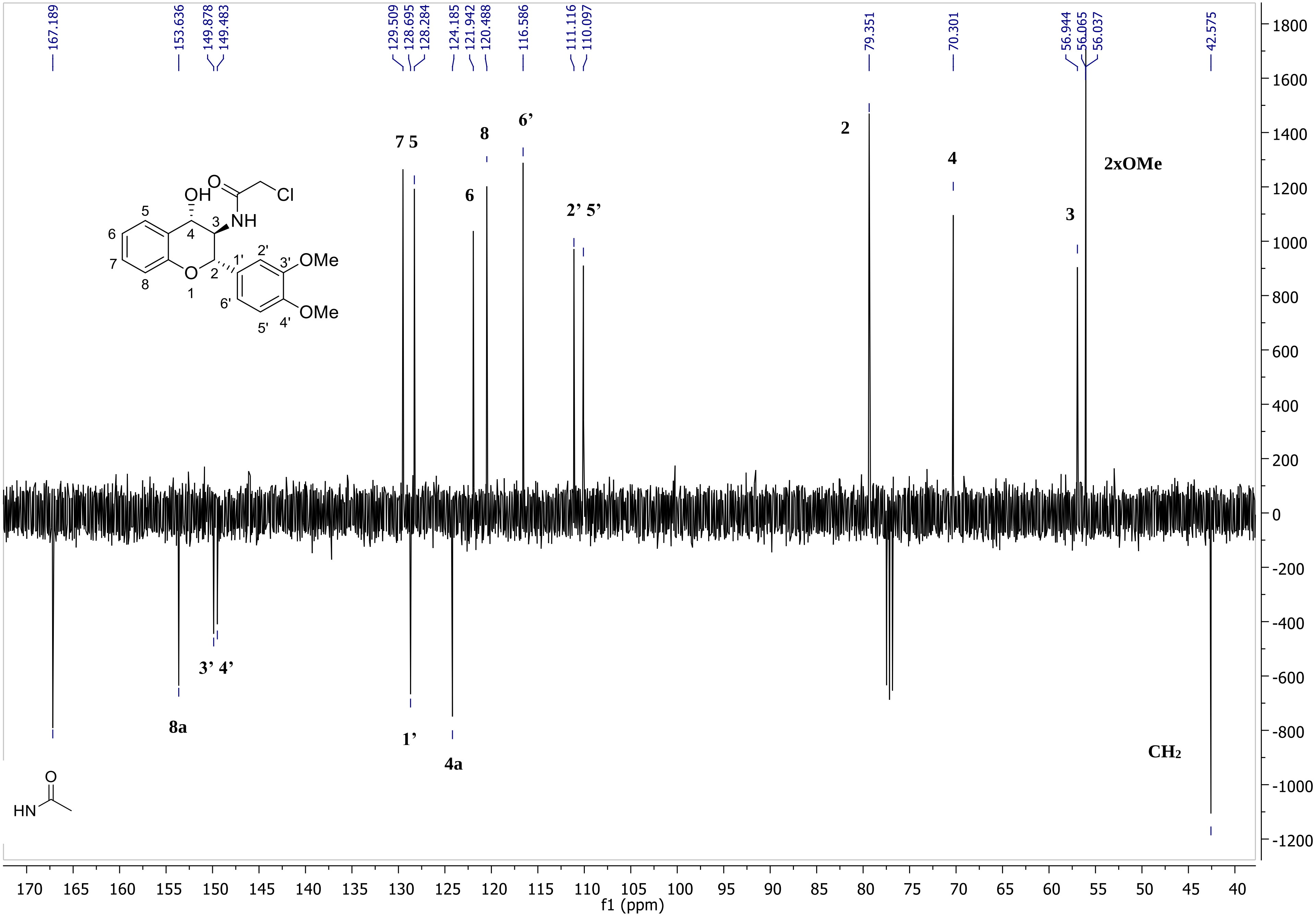

Figure S92. ^{13}C-NMR spectrum of *rac*-**19c** in $CDCl_3$

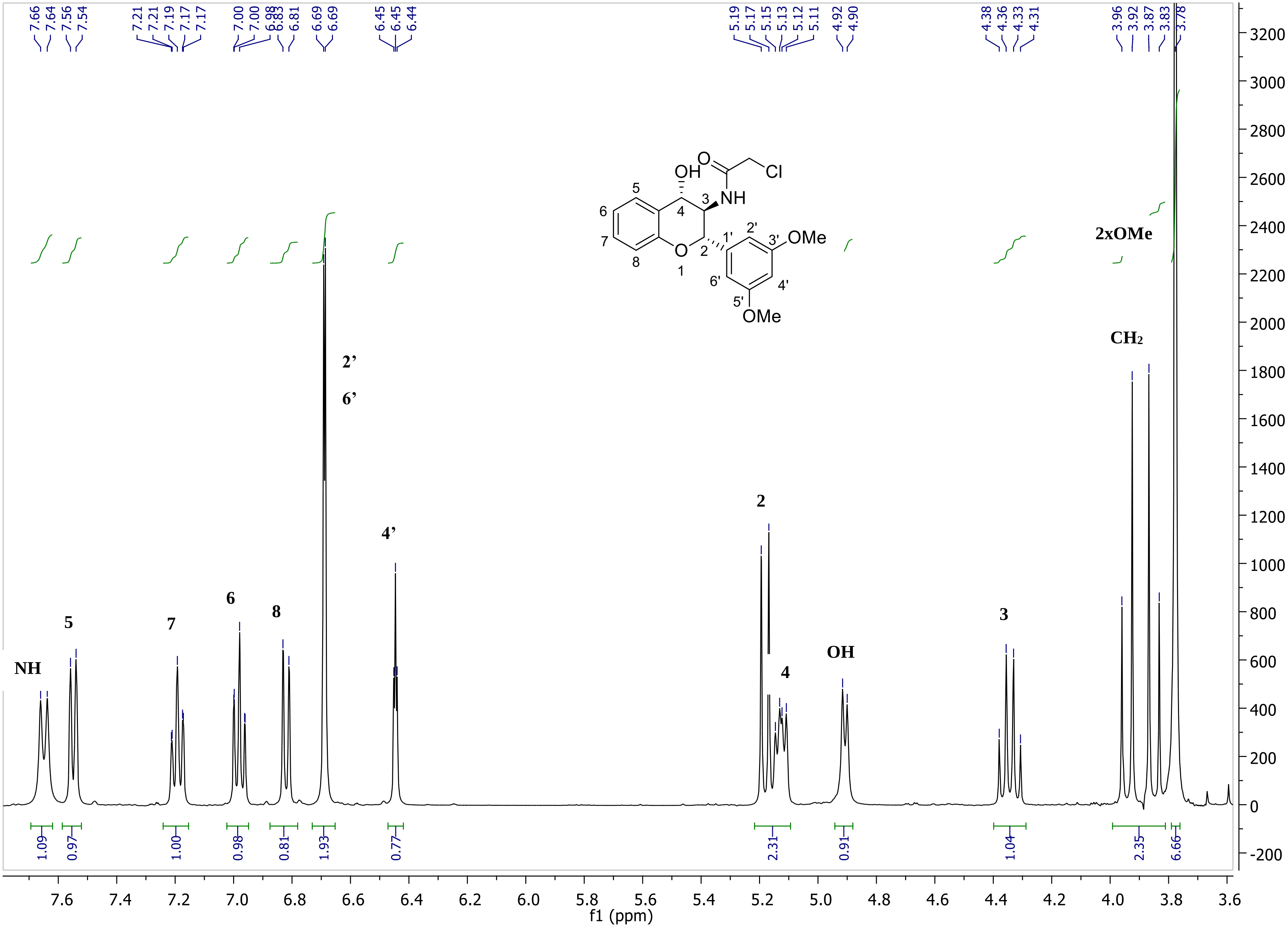

Figure S93. ^{1}H-NMR spectrum of *rac*-**19d** in Acetone-d_6

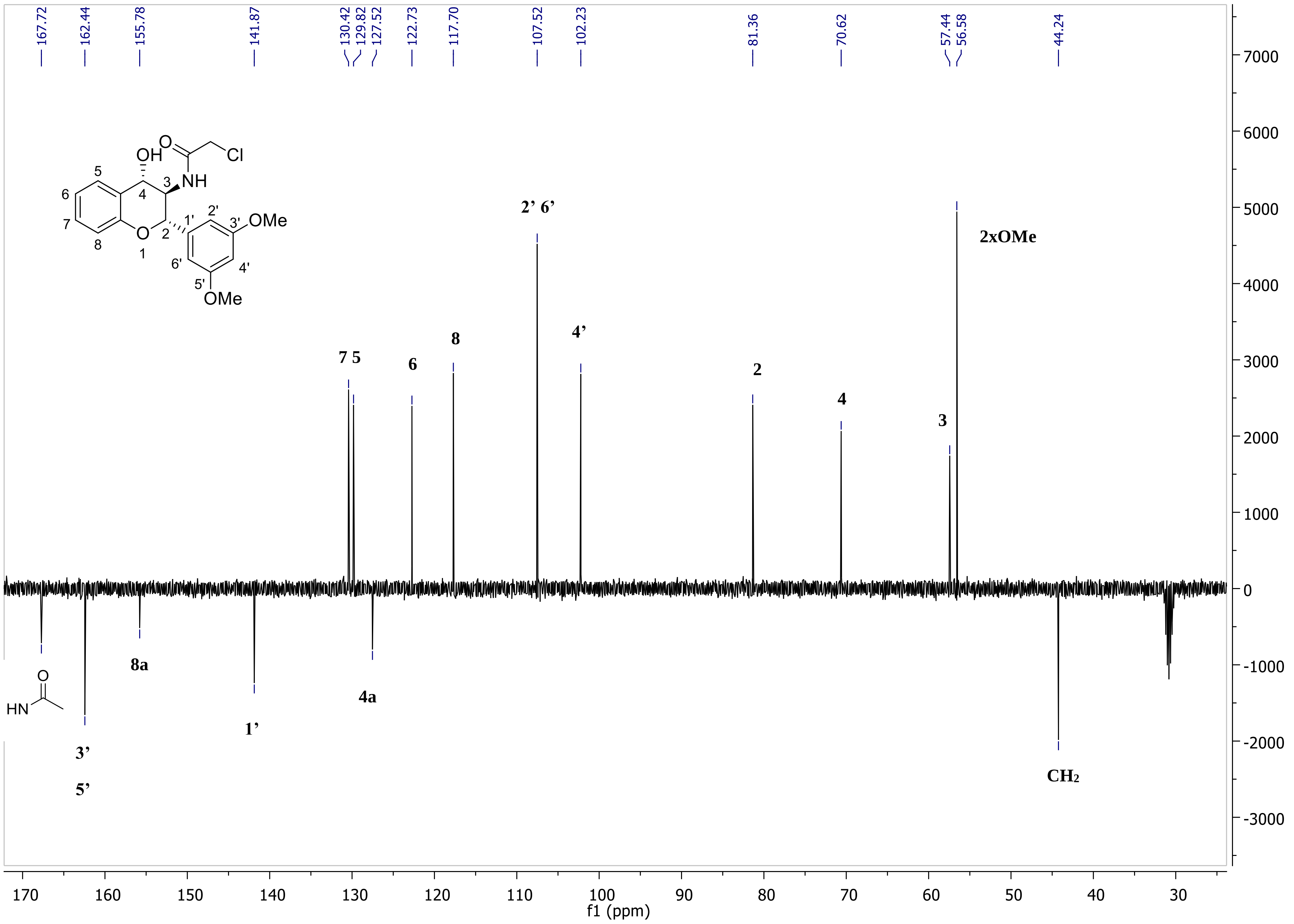

Figure S94. ^{13}C-NMR spectrum of *rac*-**19d** in Acetone-d_6

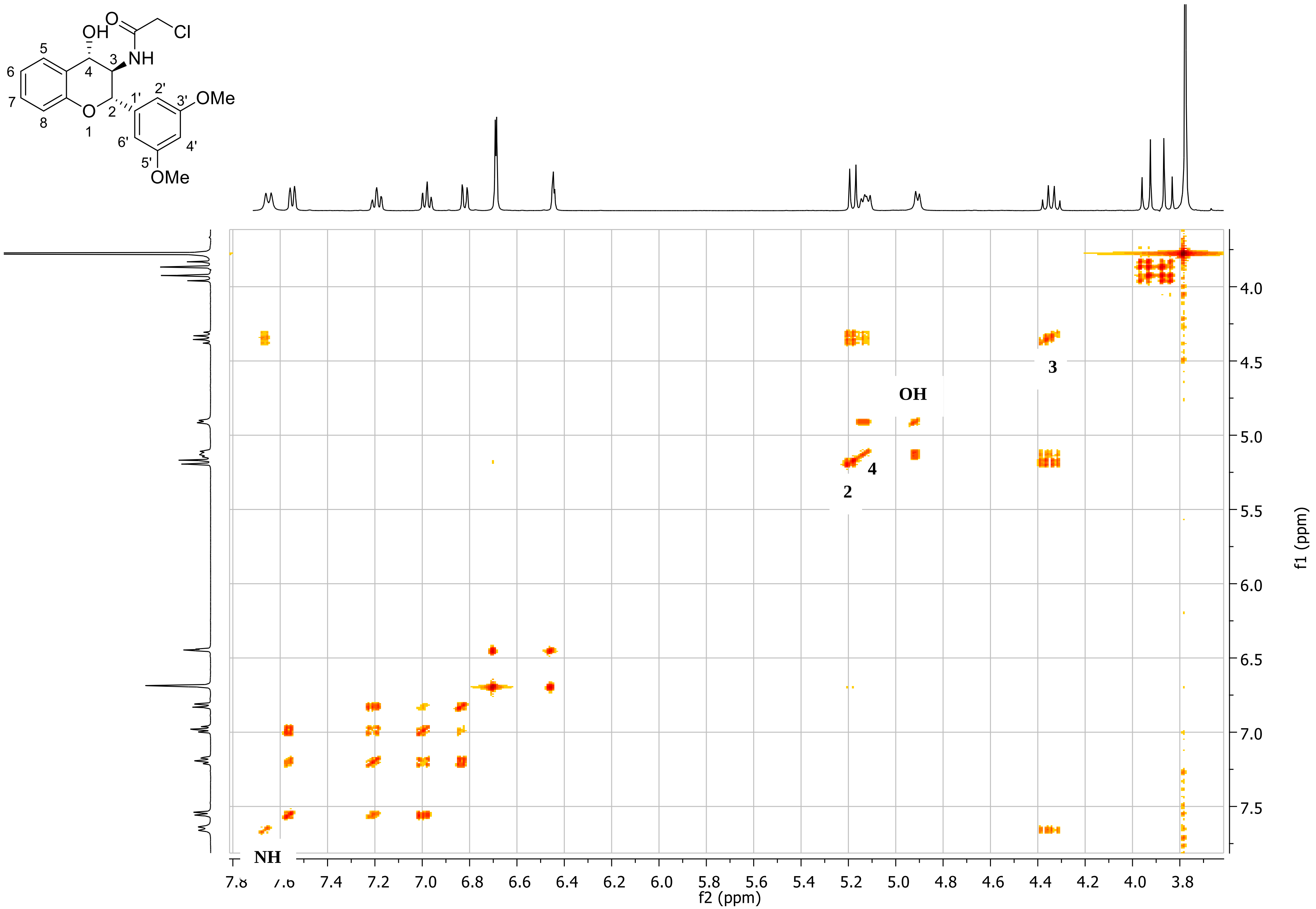

Figure S95. COSY-spectrum of *rac*-**19d** in Acetone-d_6

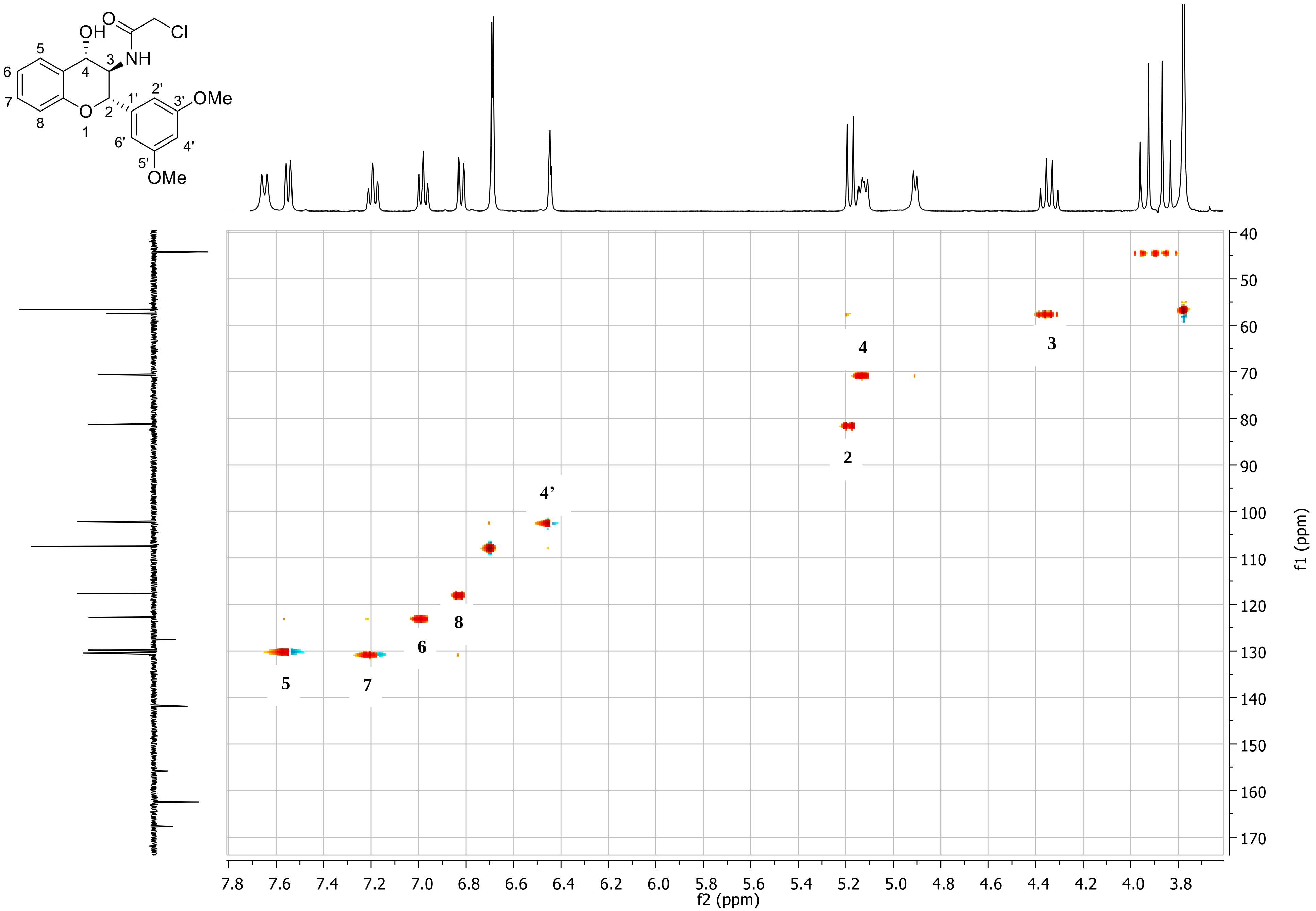

Figure S96. HSQC-spectrum of *rac*-**19d** in Acetone-d_6

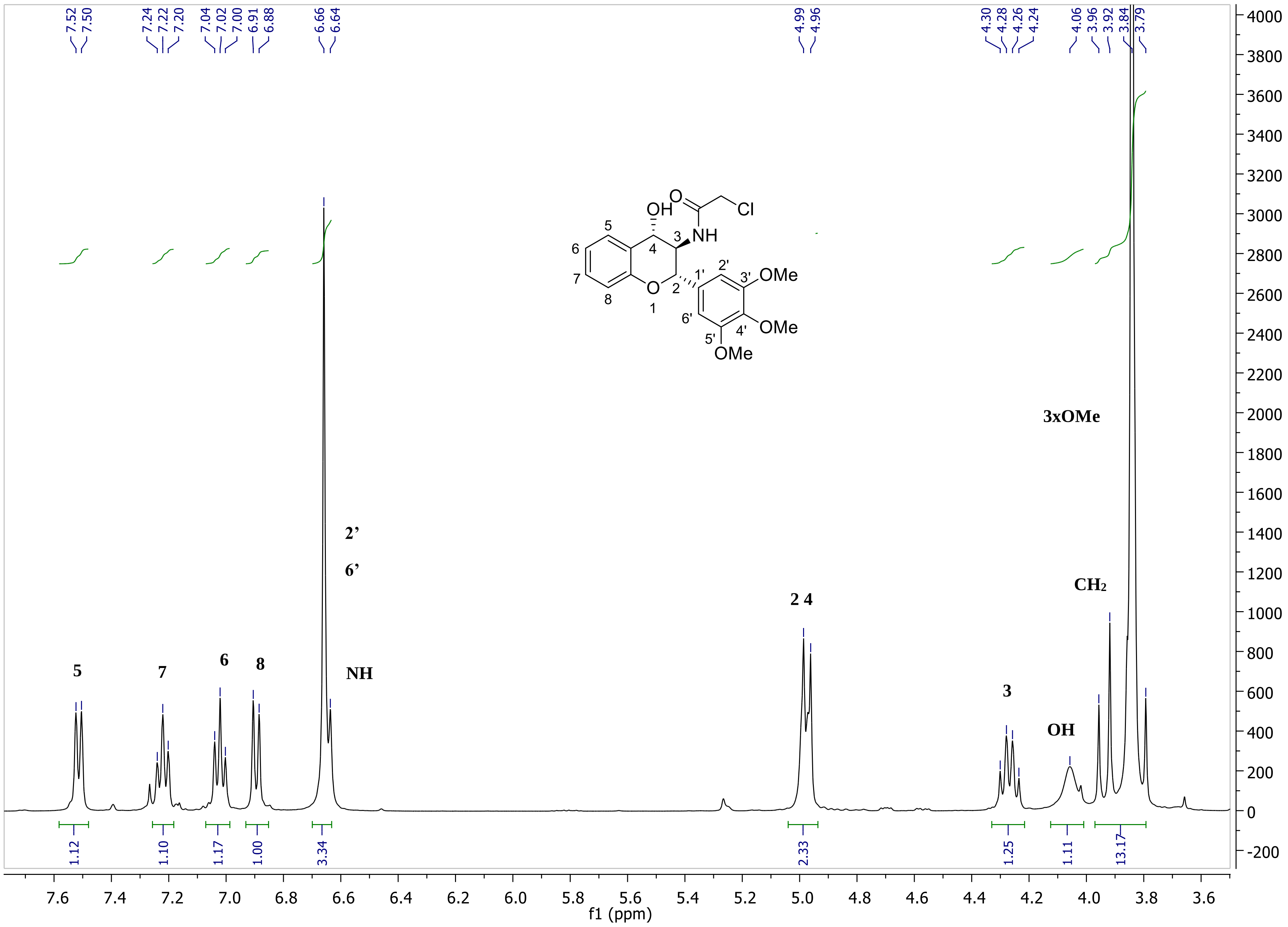

Figure S97. ^{1}H-NMR spectrum of *rac*-**19e** in $CDCl_3$

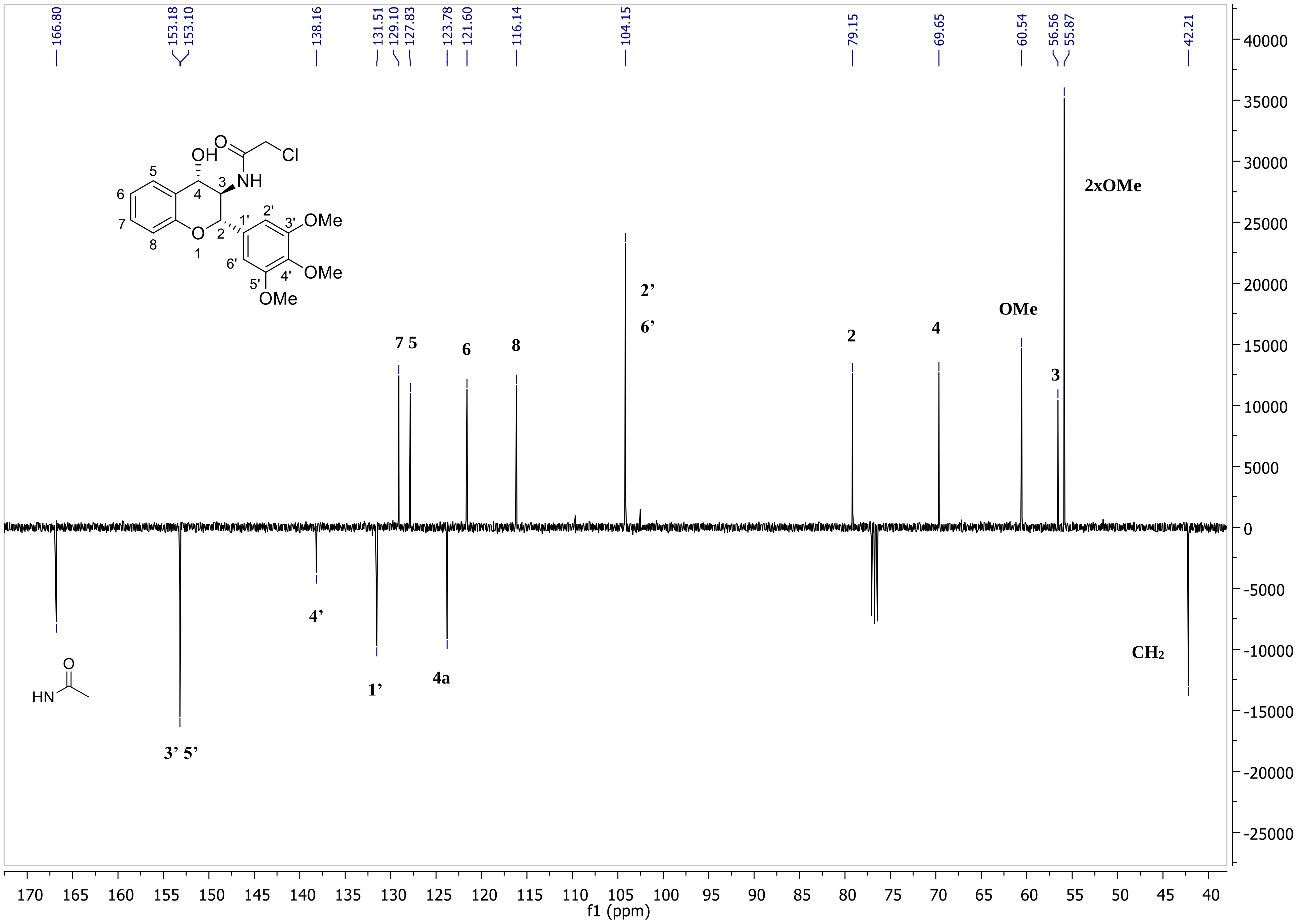

Figure S98. ^{13}C-NMR spectrum of *rac*-**19e** in $CDCl_3$

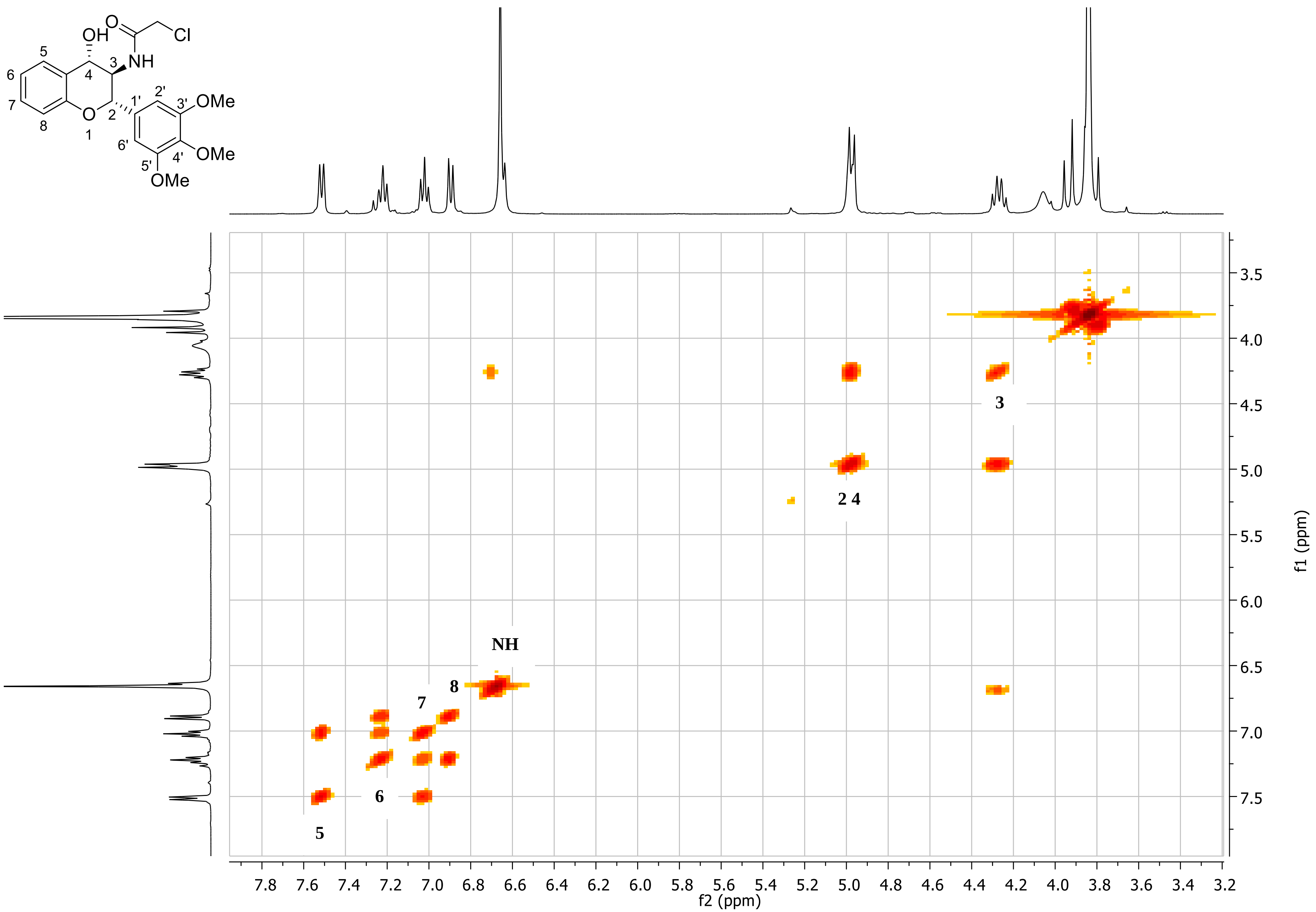

Figure S99. COSY-spectrum of *rac*-**19e** in $CDCl_3$

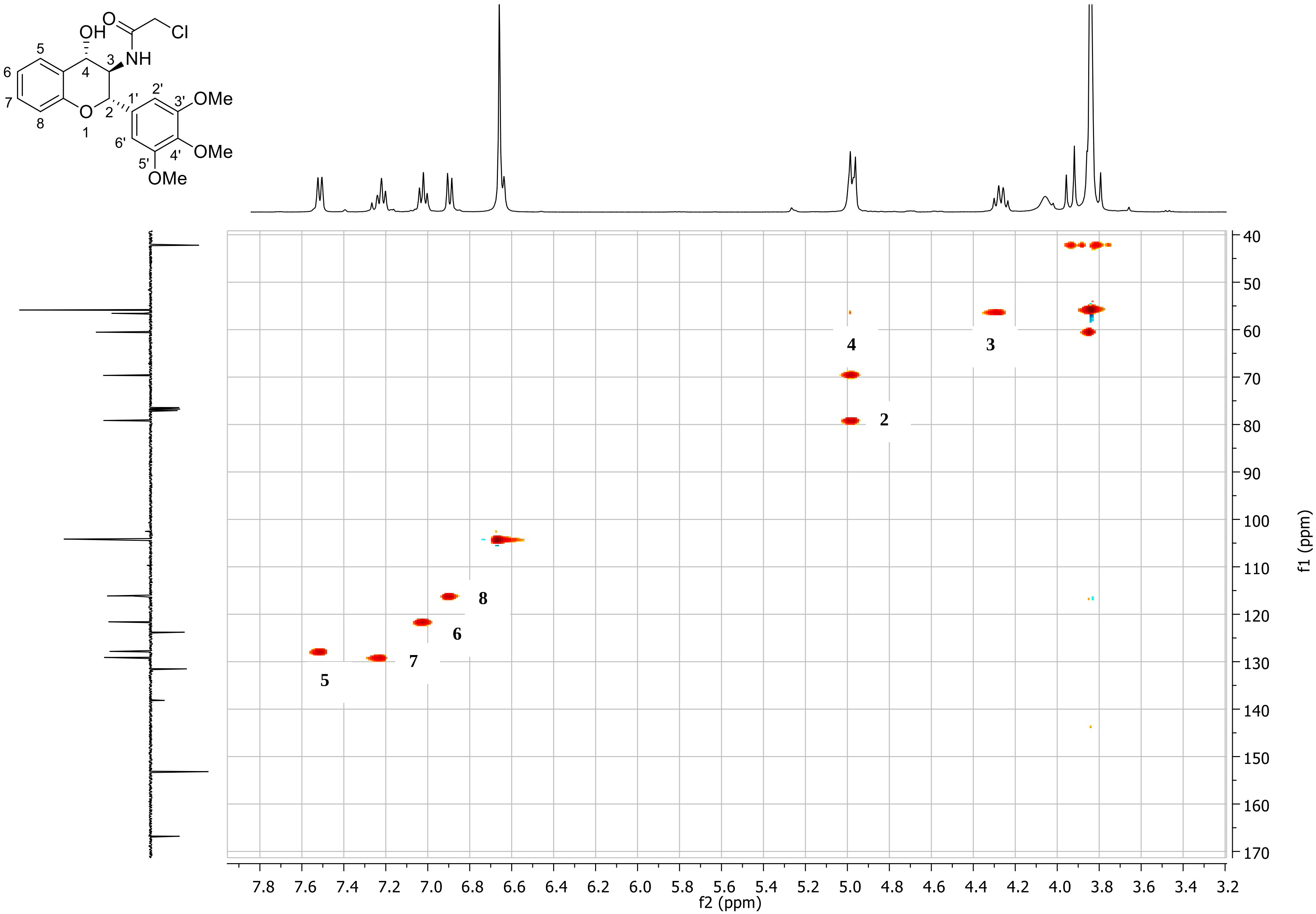

Figure S100. COSY-spectrum of *rac*-**19e** in $CDCl_3$

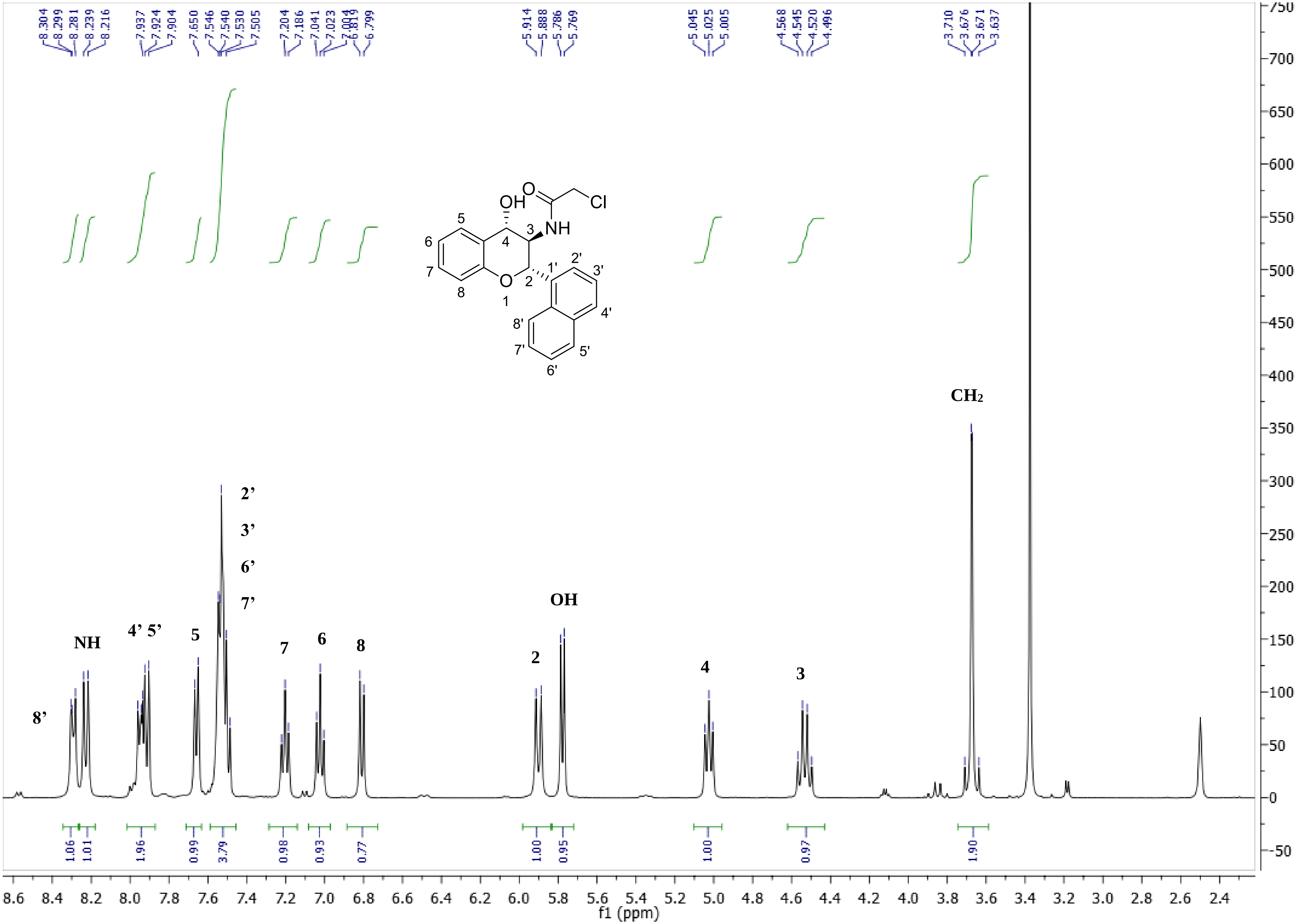

Figure S101. ^{1}H-NMR spectrum of *rac*-**19f** in DMSO-d_6

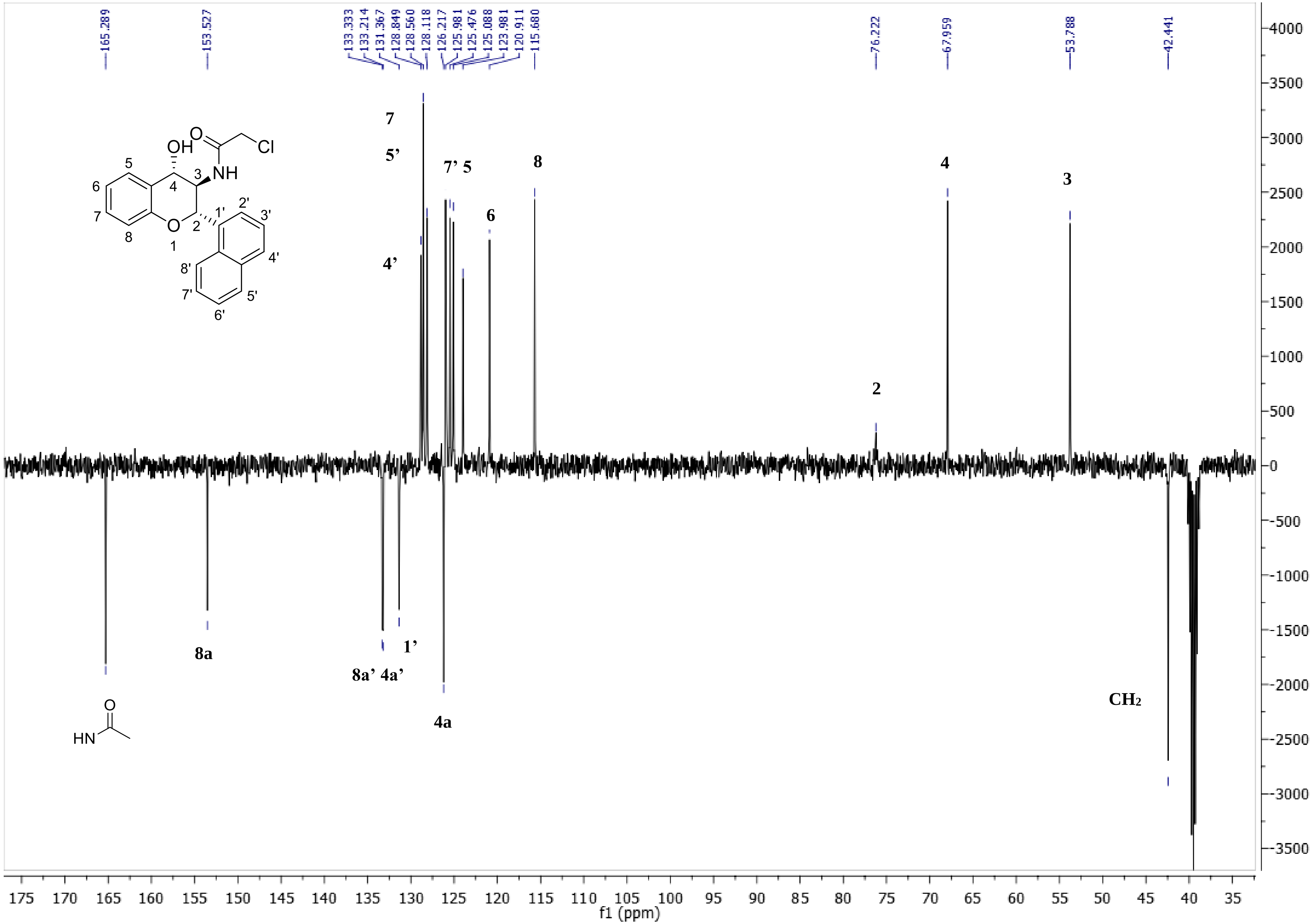

Figure S102. ^{13}C-NMR spectrum of *rac*-**19f** in DMSO-d_6

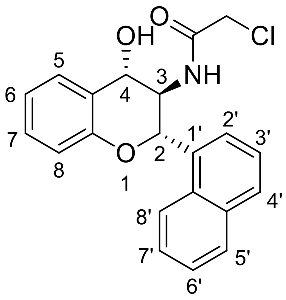

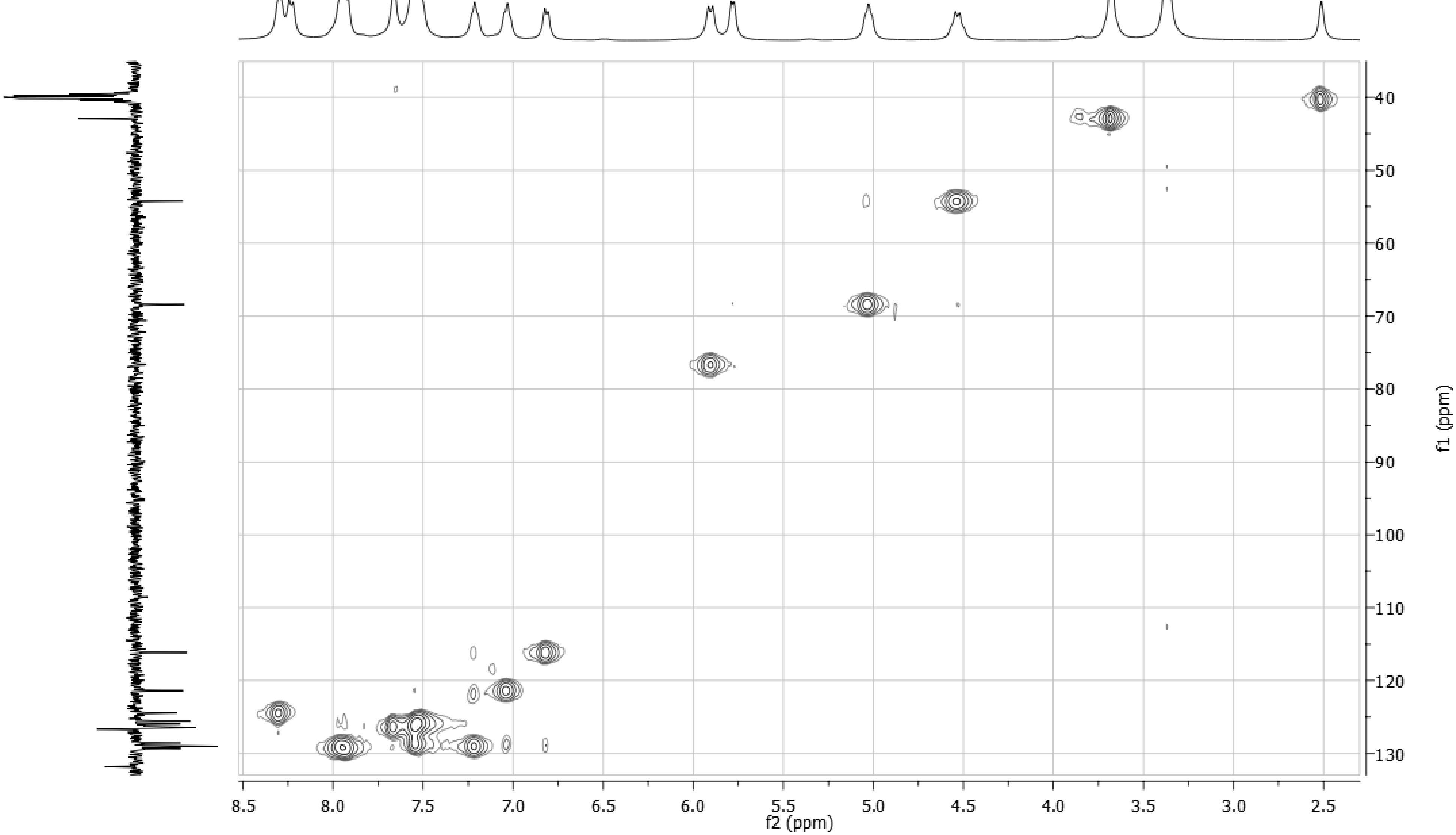

Figure S103. HSQC spectrum of *rac*-**19f** in DMSO-d_6

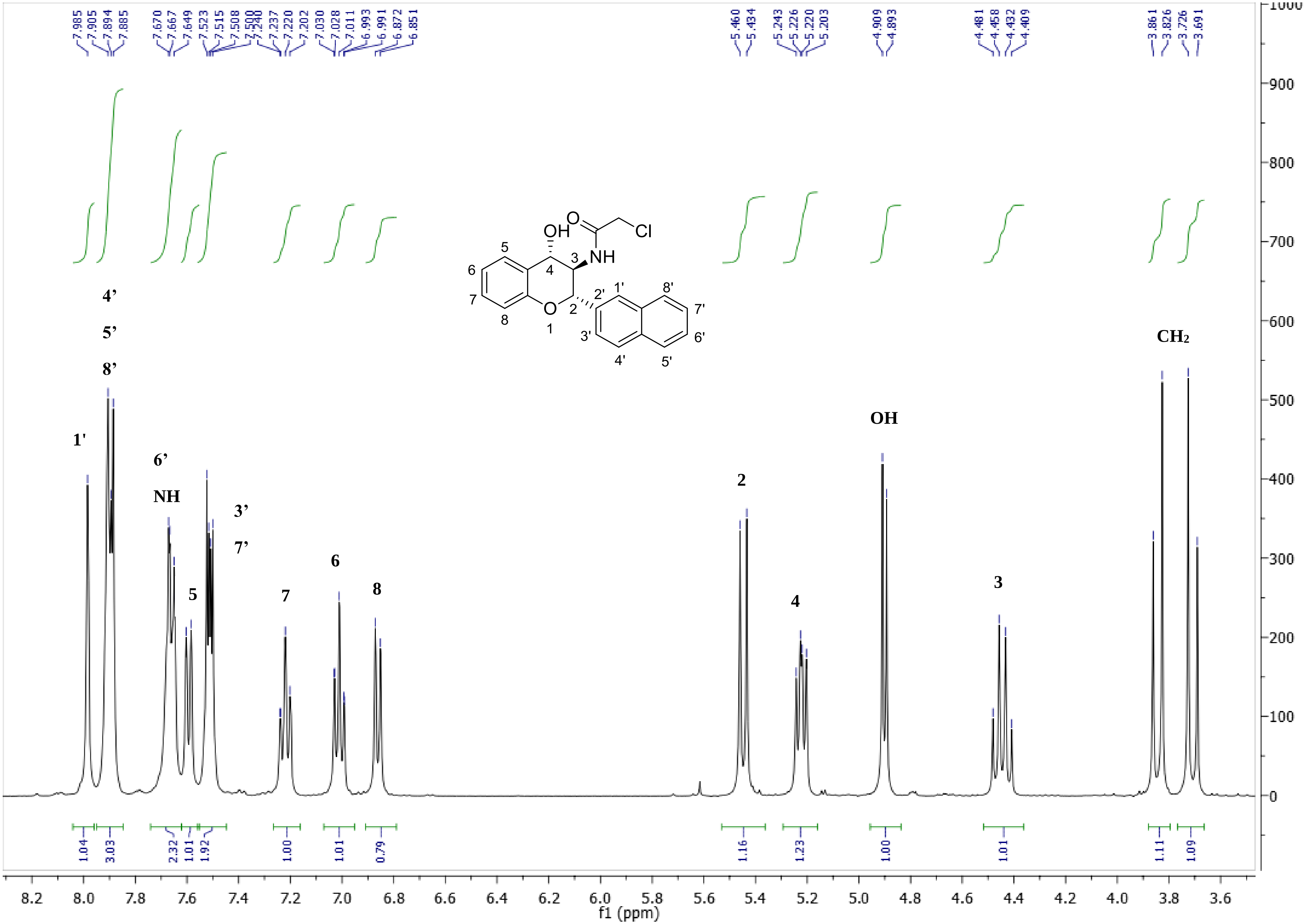

Figure S104. ^{1}H-NMR spectrum of *rac*-**19g** in Acetone-d$_6$

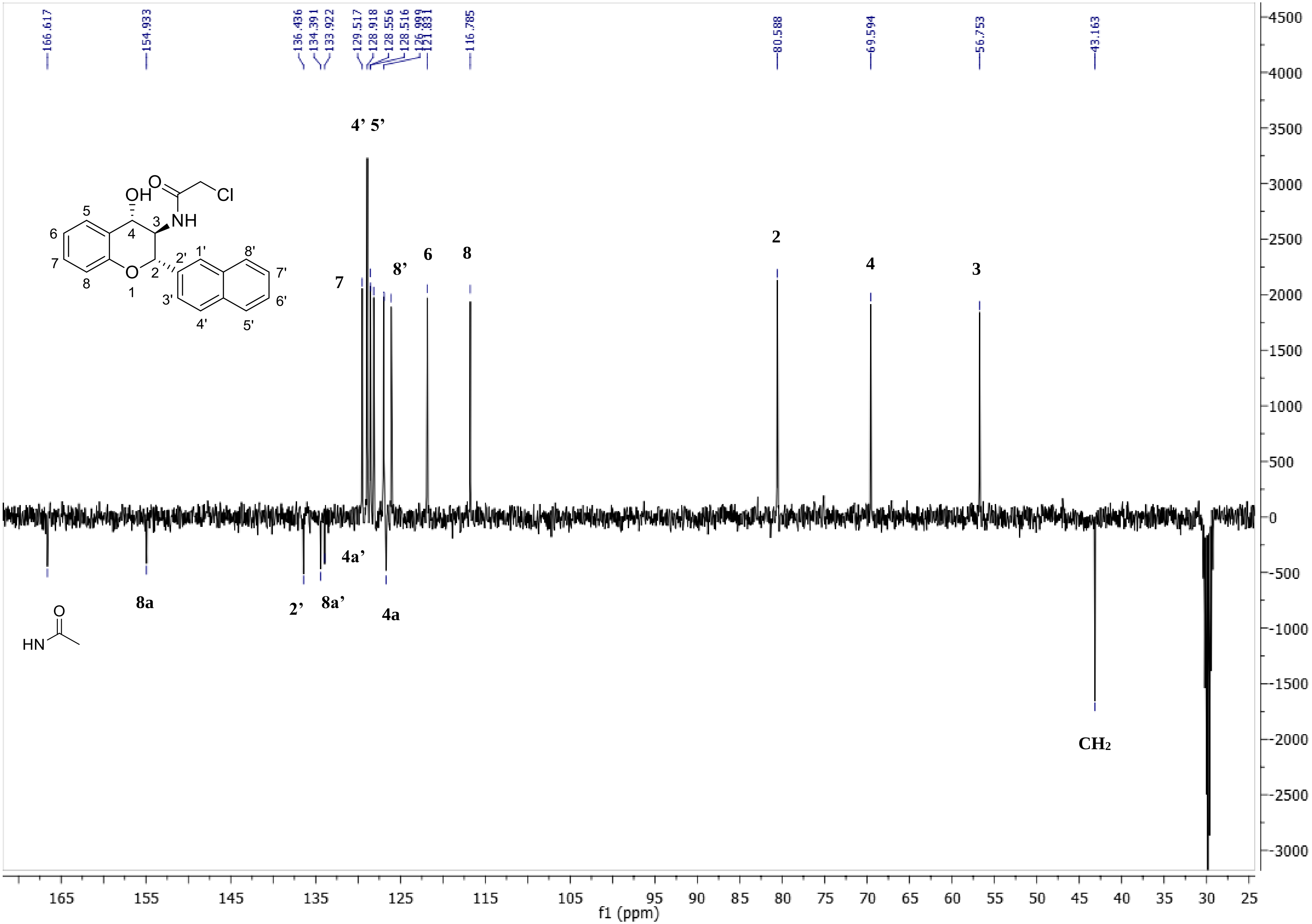

Figure S105. ^{13}C-NMR spectrum of *rac*-**19g** in Acetone-d_6

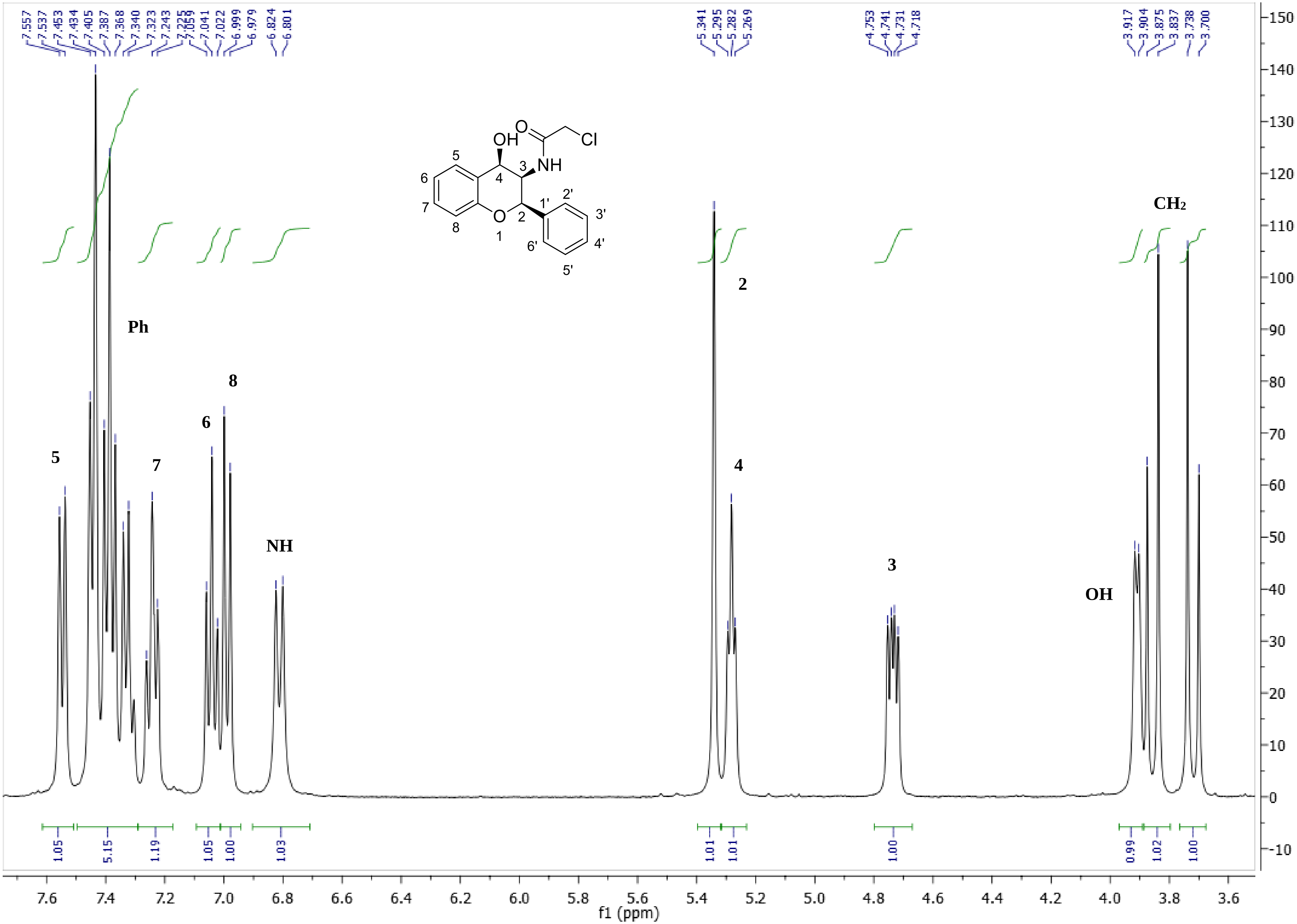

Figure S106. ^{1}H-NMR spectrum of *rac*-**22a** in $CDCl_3$

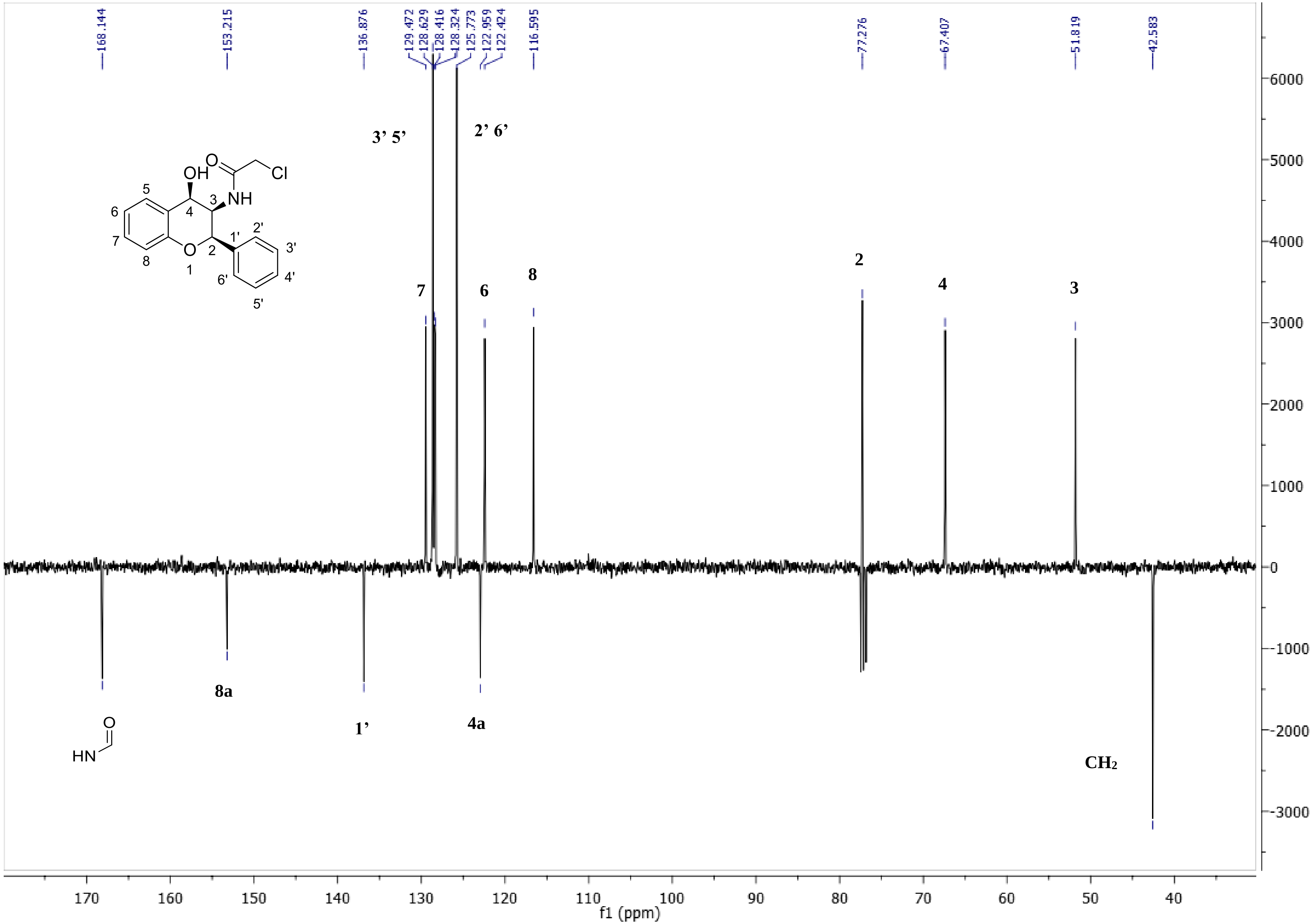

Figure S107. ^{13}C-NMR spectrum of *rac*-**22a** in $CDCl_3$

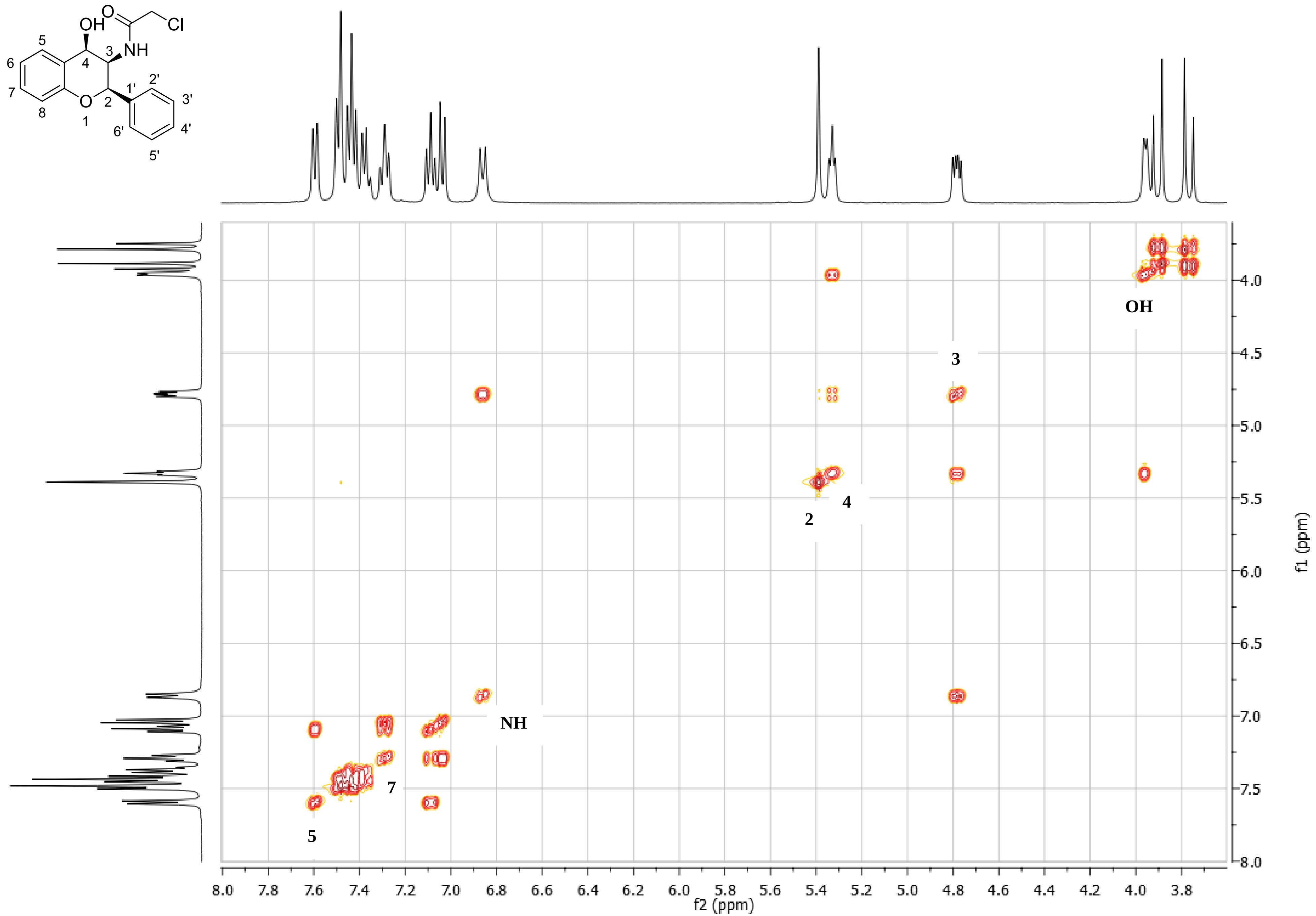

Figure S108. COSY spectrum of *rac*-**22a** in $CDCl_3$

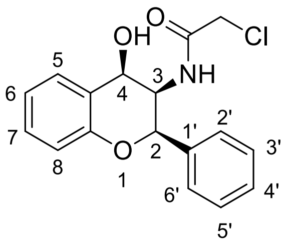

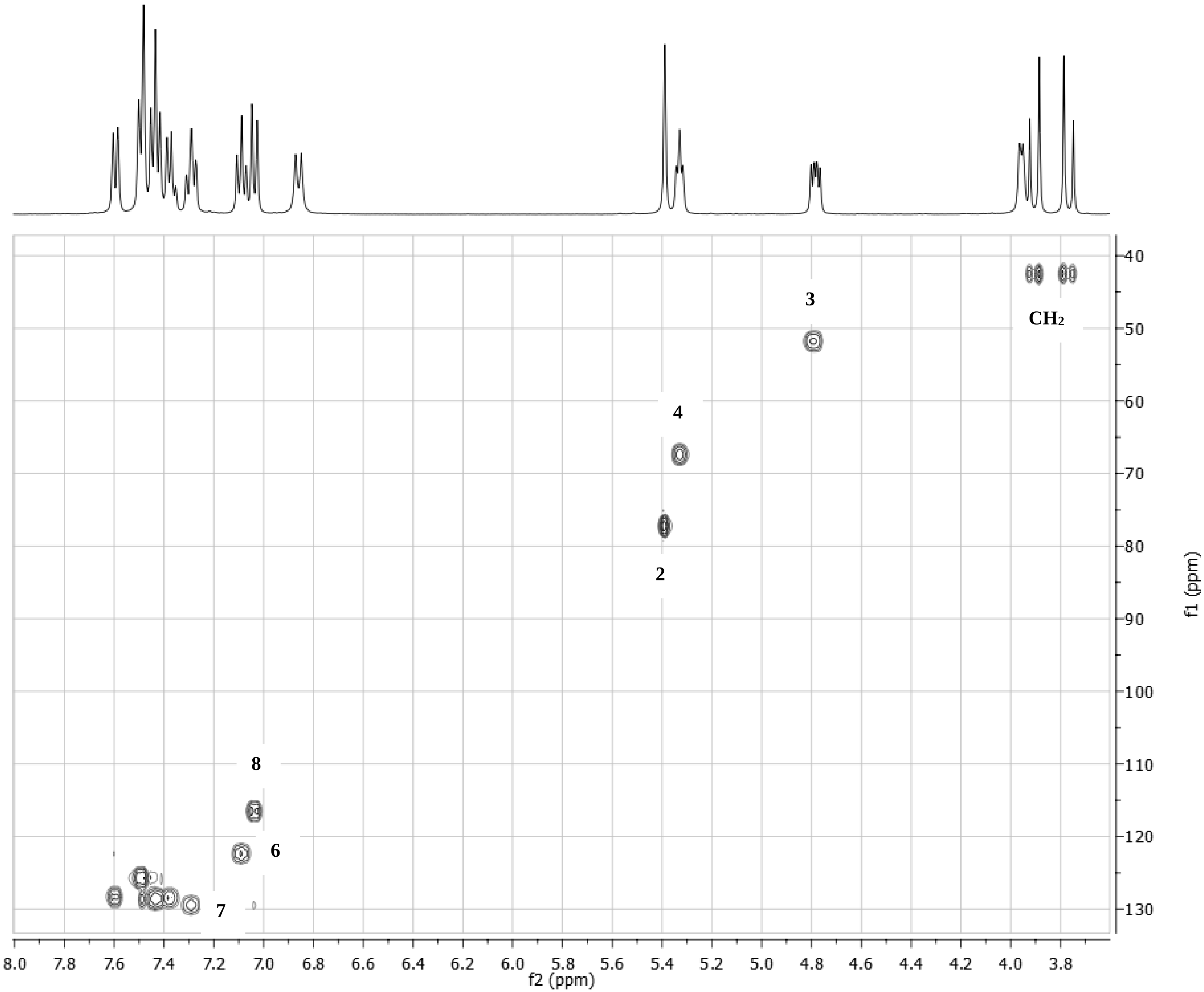

Figure S109. HSQC spectrum of *rac*-**22a** in $CDCl_3$

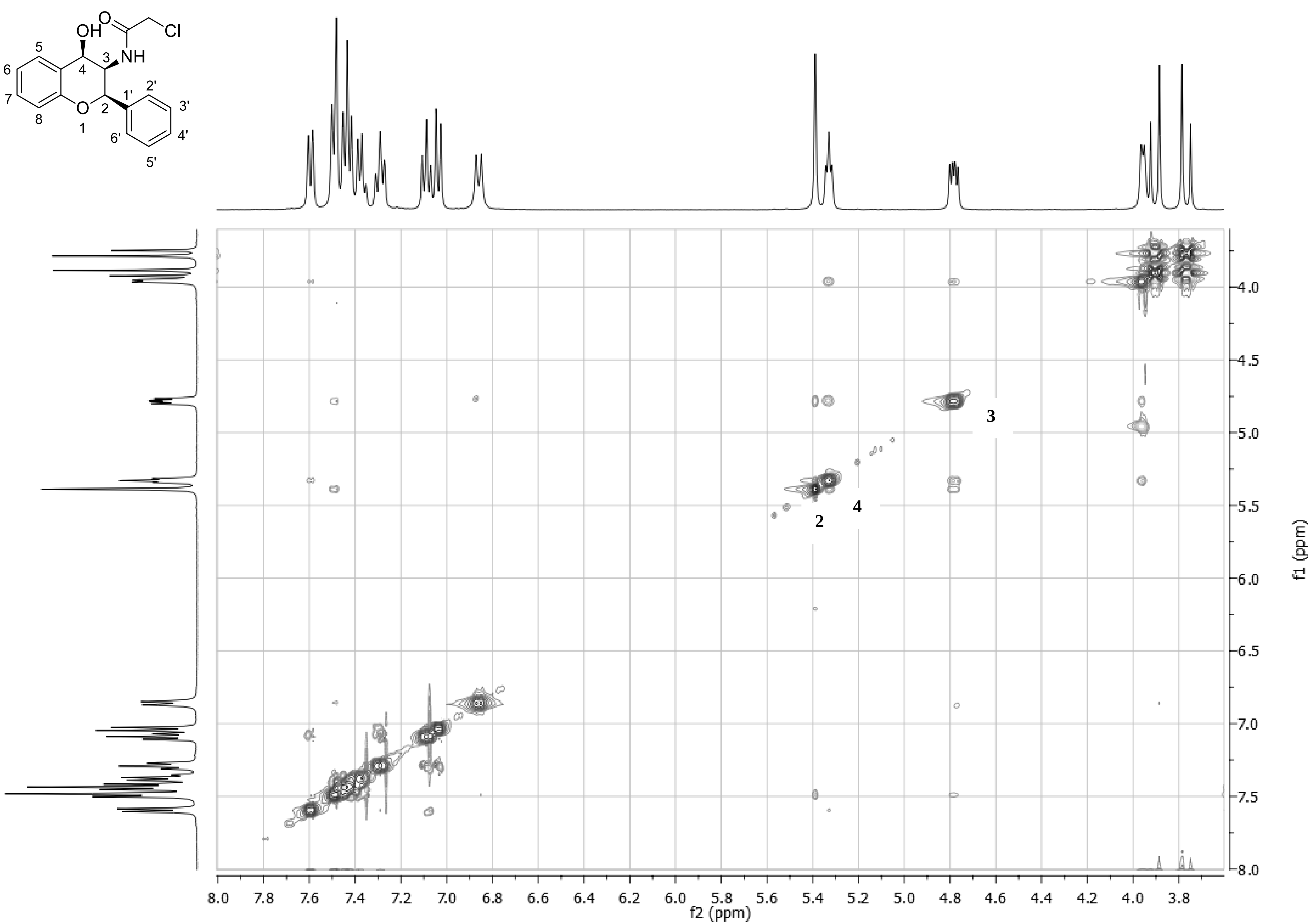

Figure S110. NOESY spectrum of *rac*-**22a** in $CDCl_3$

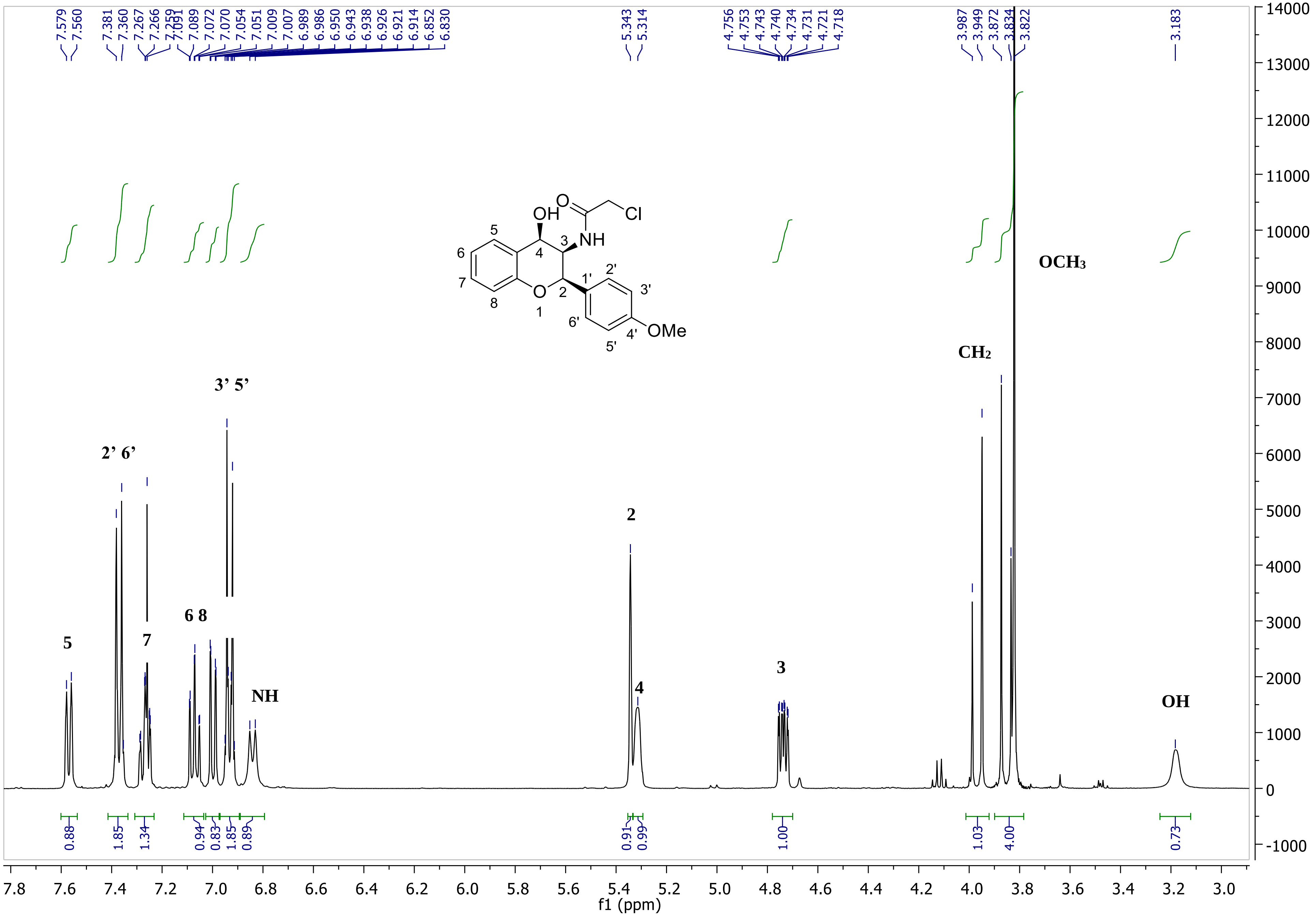

Figure S111. ^{1}H-NMR spectrum of *rac*-**22b** in $CDCl_3$

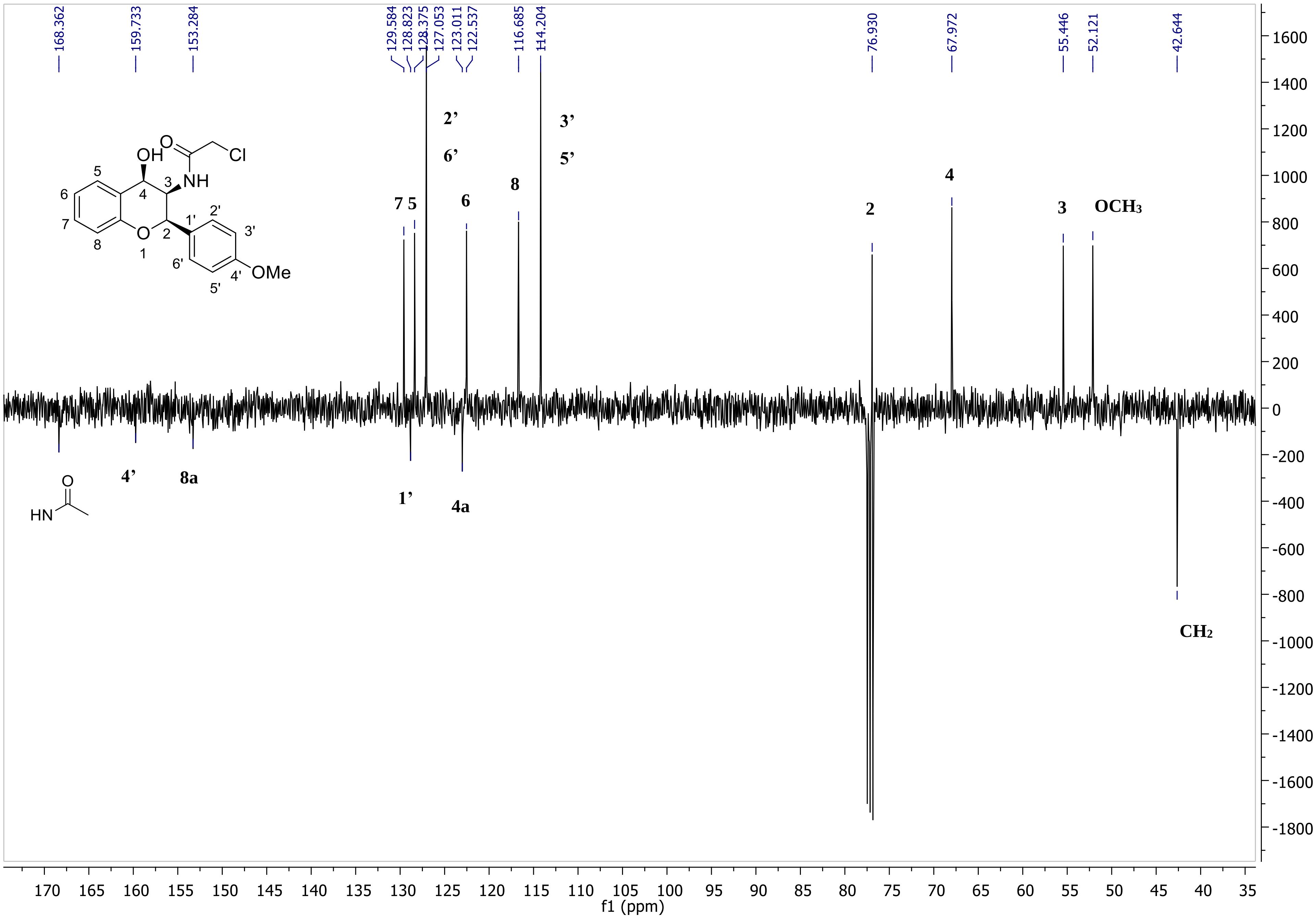

Figure S112. ^{13}C-NMR spectrum of *rac*-**22b** in $CDCl_3$

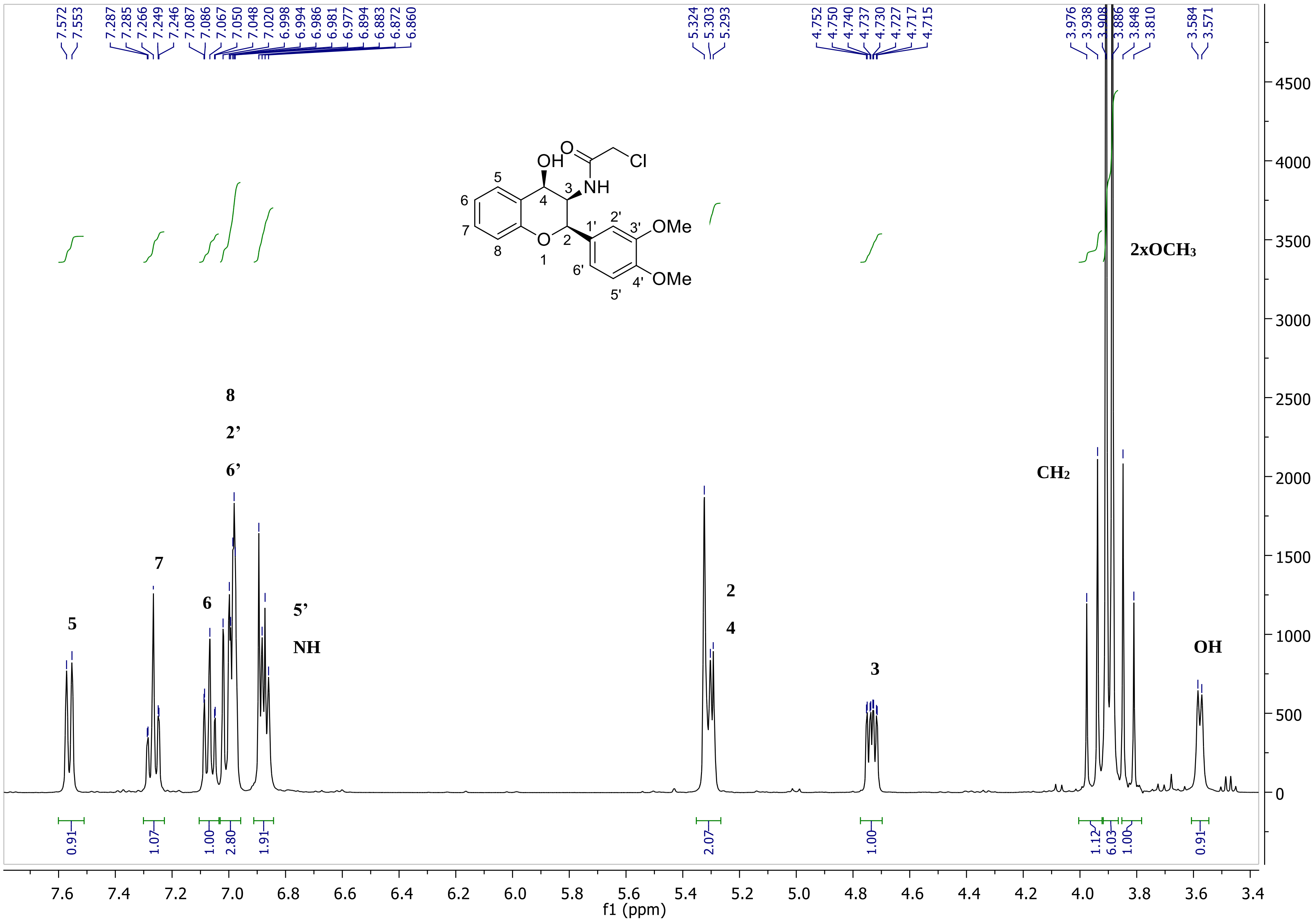

Figure S113. ^{1}H-NMR spectrum of *rac*-**22c** in $CDCl_3$

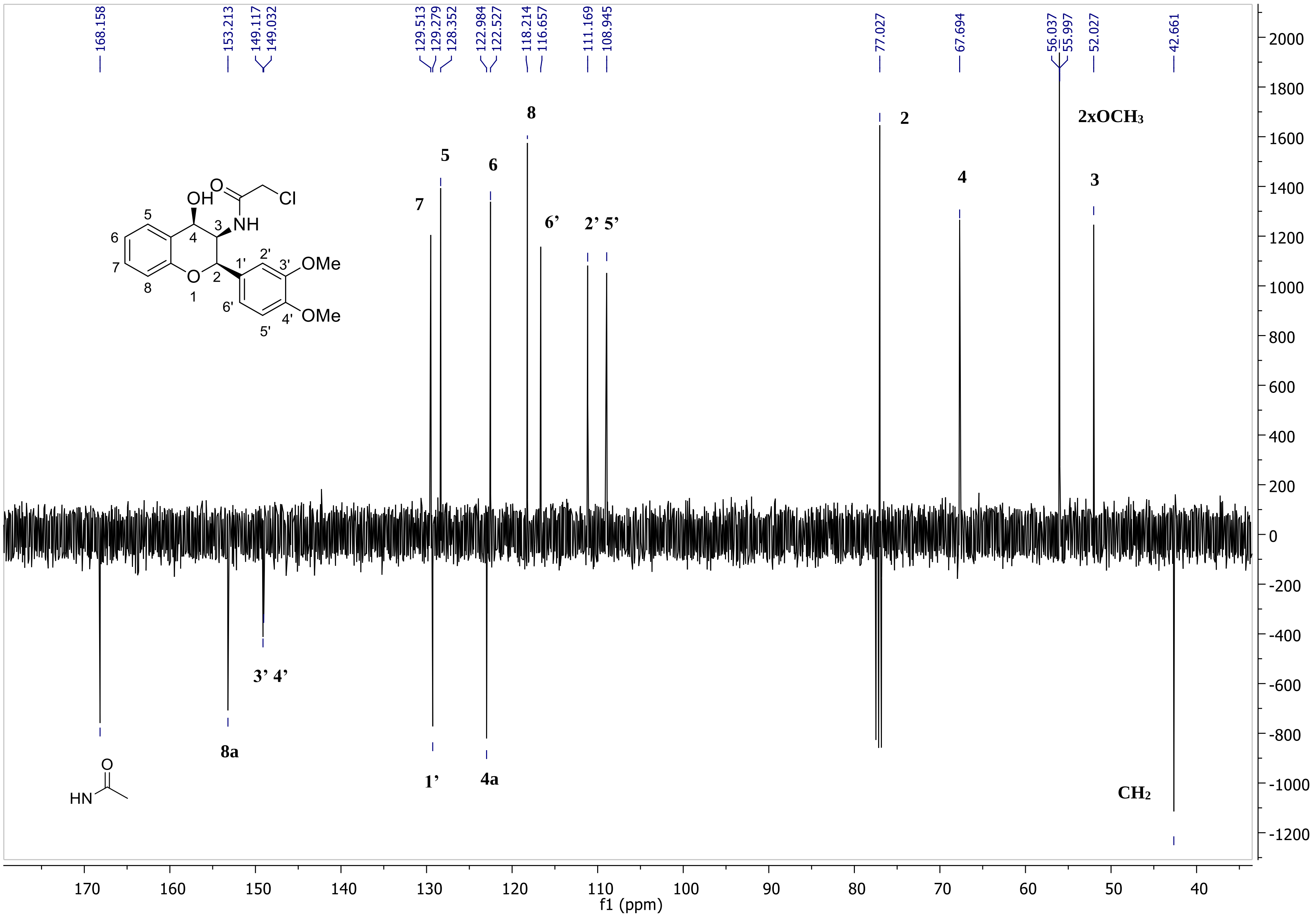

Figure S114. ^{13}C-NMR spectrum of *rac*-**22c** in $CDCl_3$

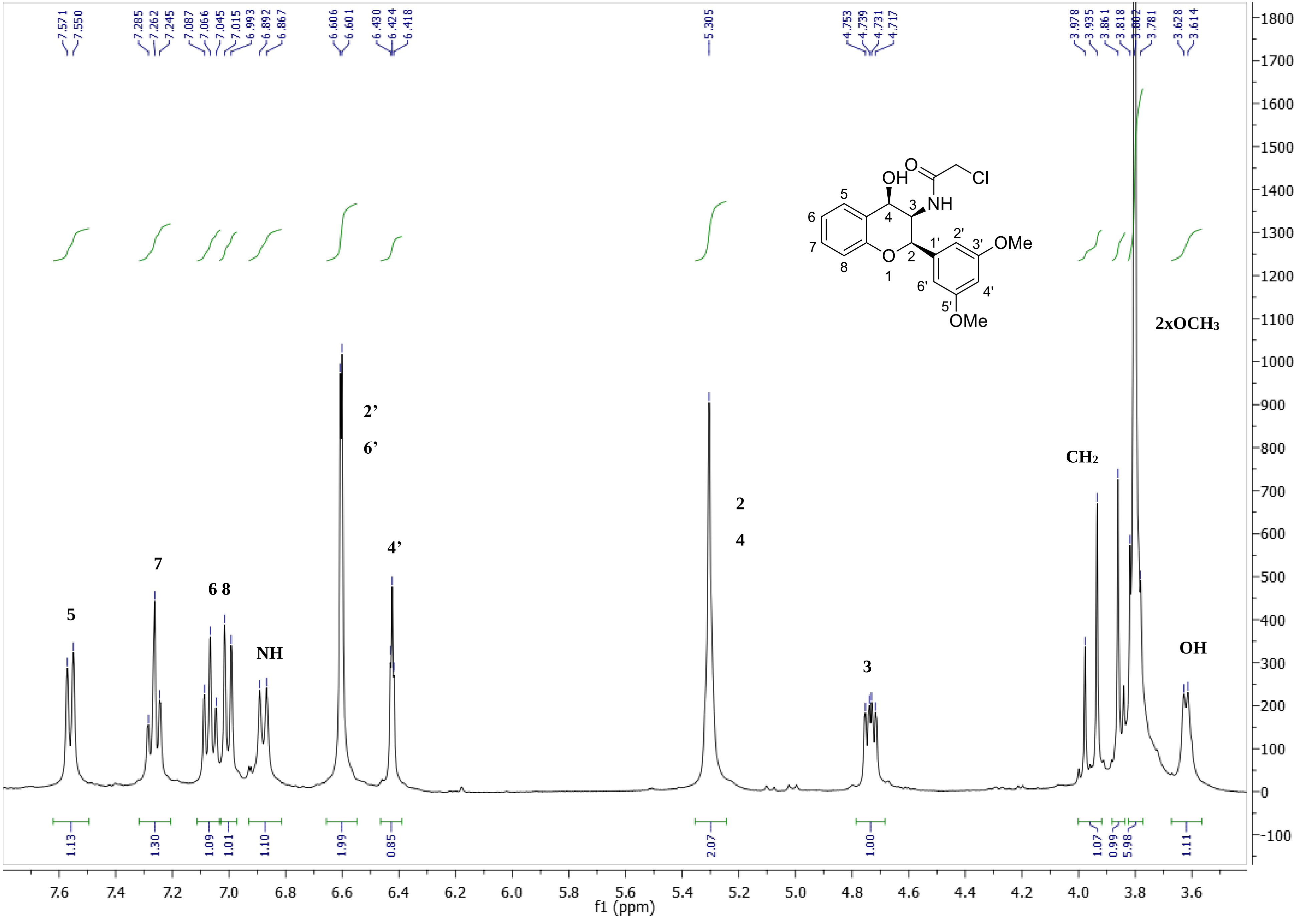

Figure S115. ^{1}H-NMR spectrum of *rac*-**22d** in $CDCl_3$

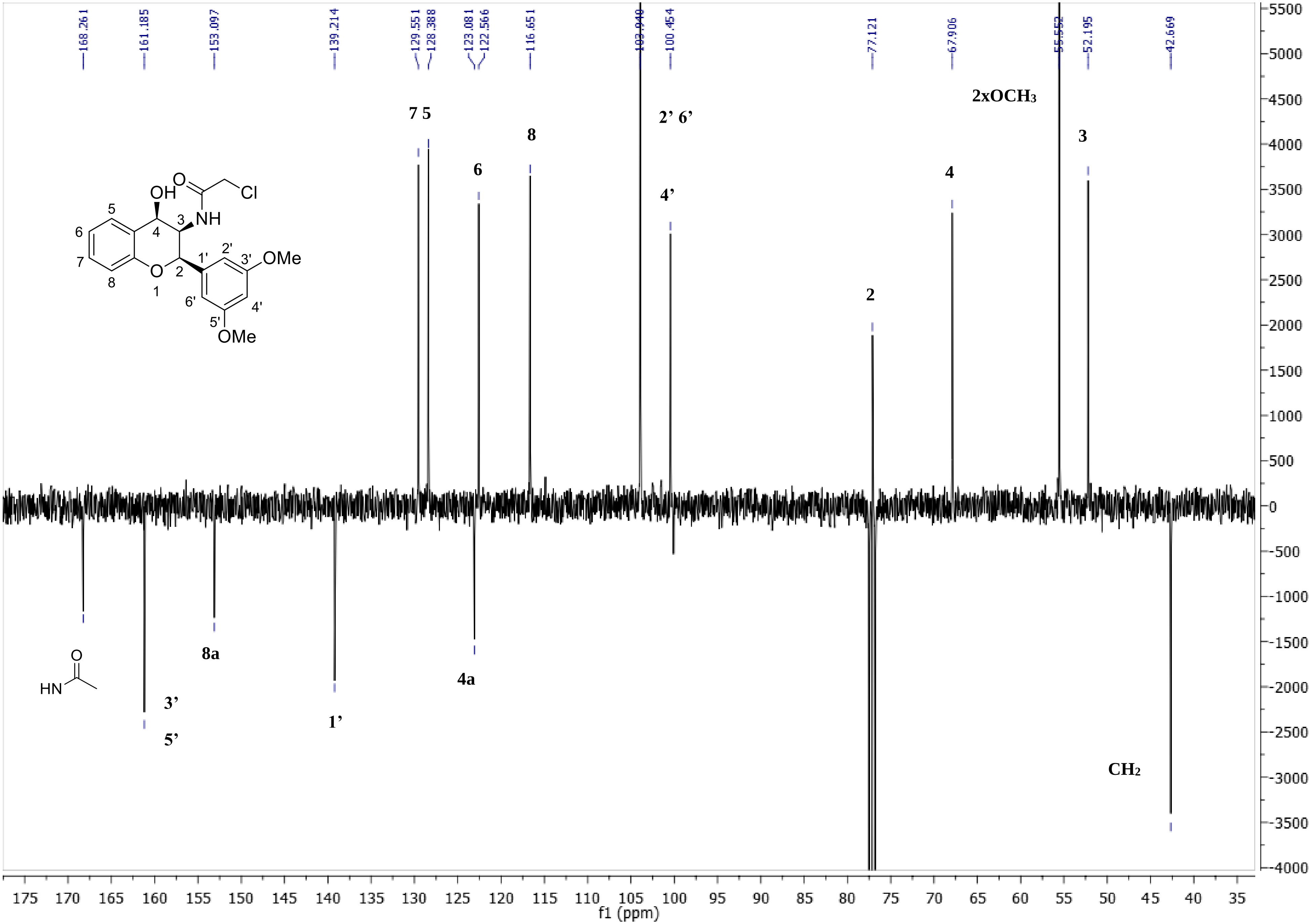

Figure S116. ^{13}C-NMR spectrum of *rac*-**22d** in $CDCl_3$

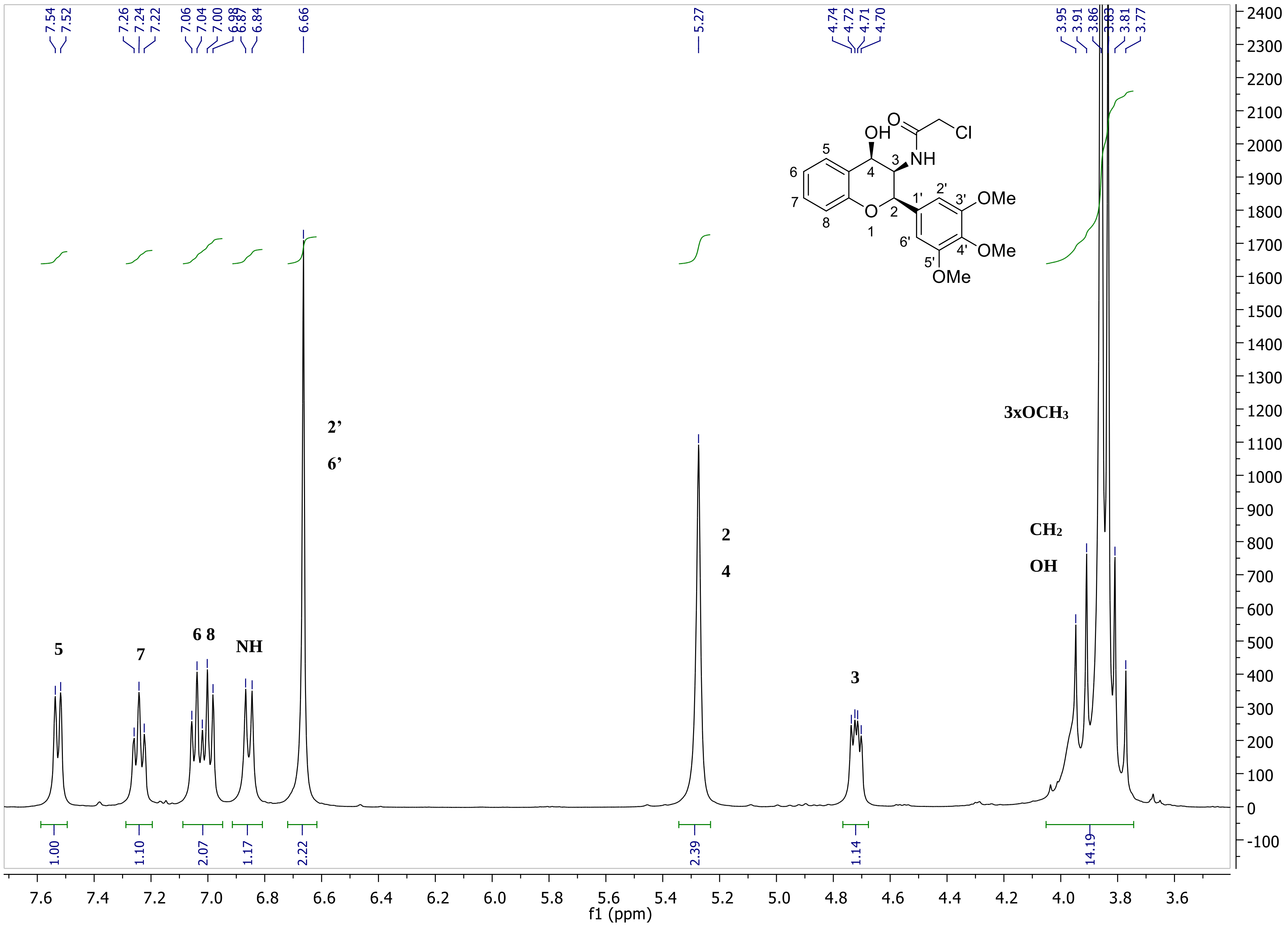

Figure S117. ^{1}H-NMR spectrum of *rac*-**22e** in $CDCl_3$

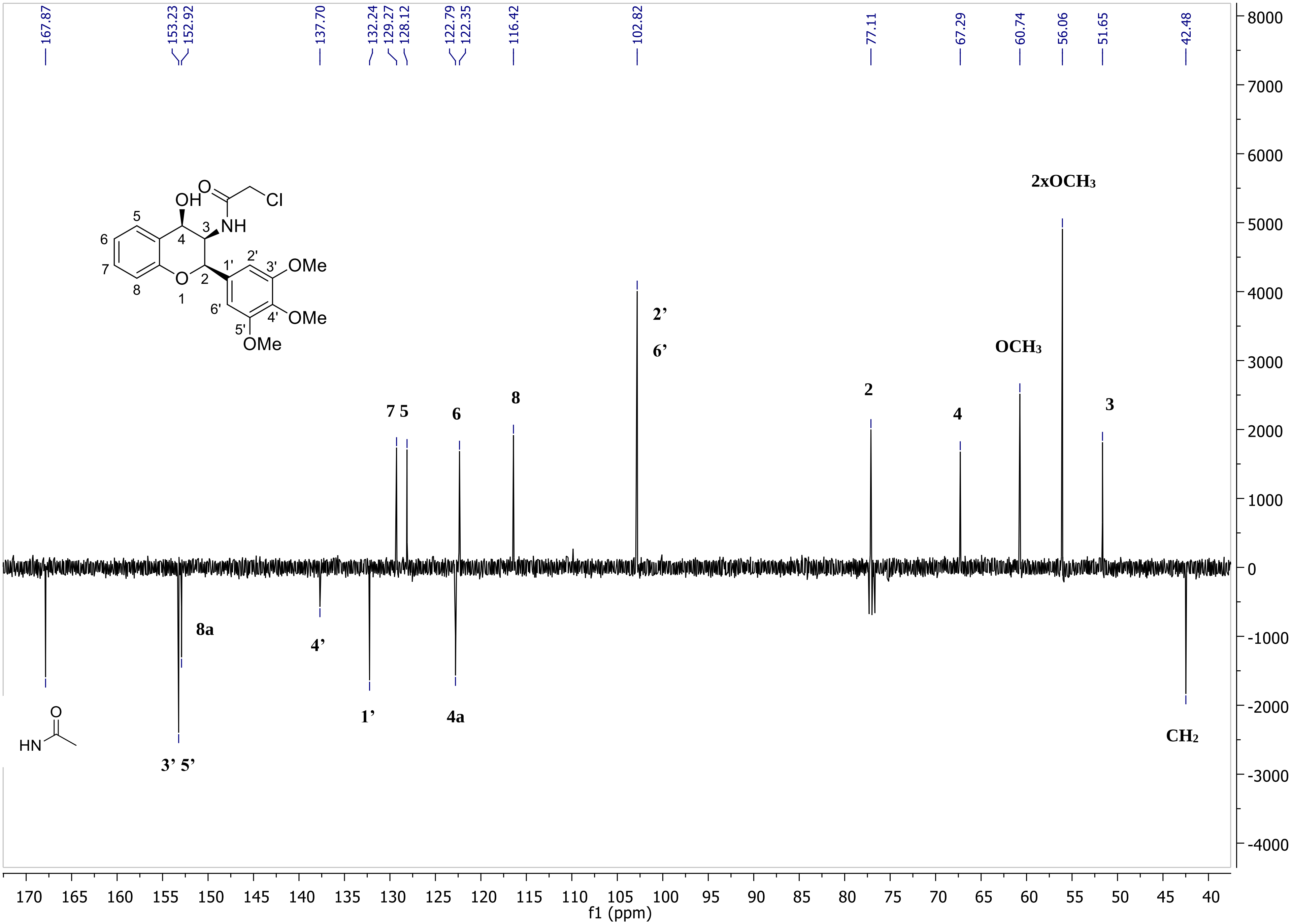

Figure S118. ^{13}C-NMR spectrum of *rac*-**22e** in $CDCl_3$

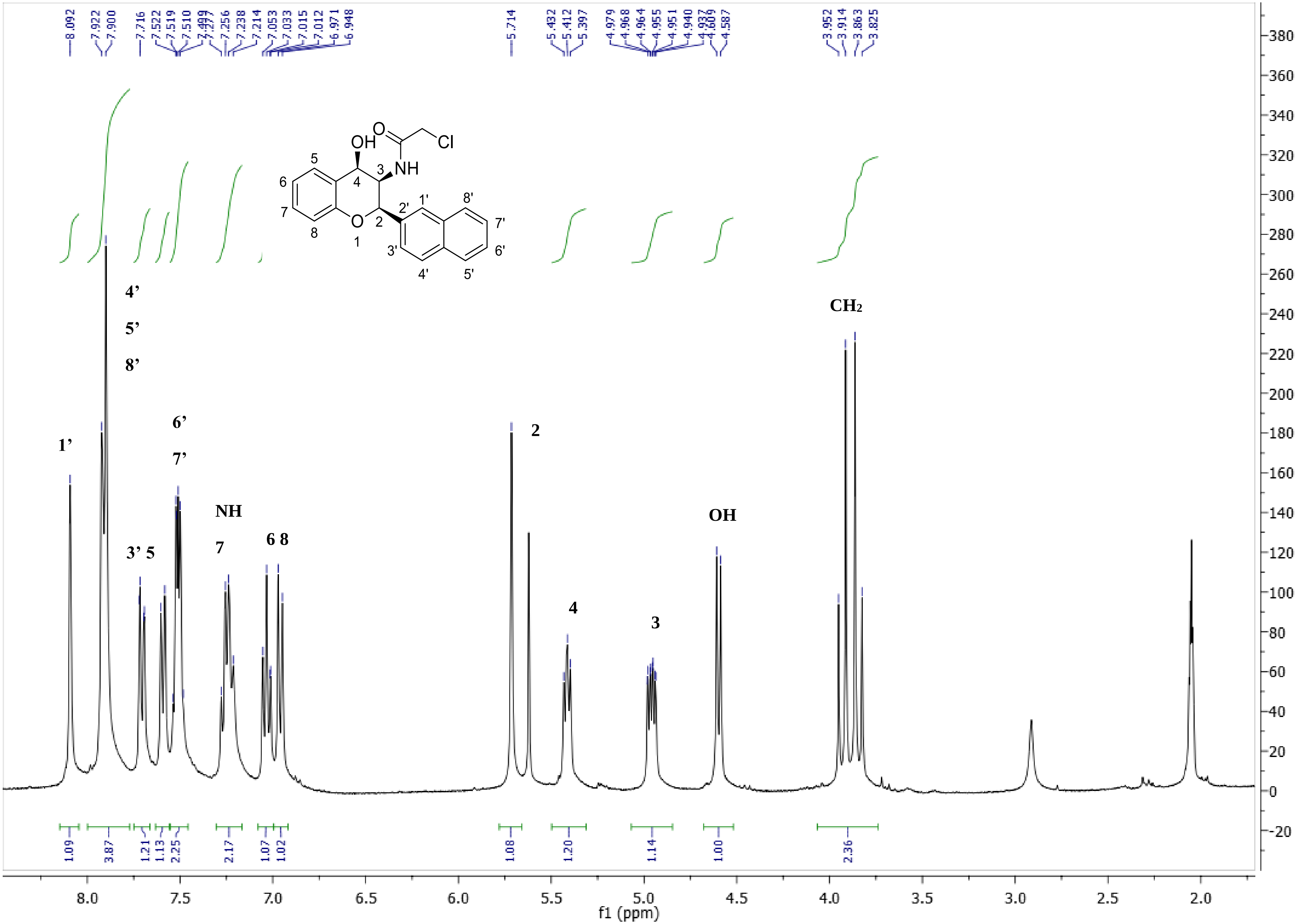

Figure S119. ^{1}H-NMR spectrum of *rac*-**22g** in Acetone-d_6

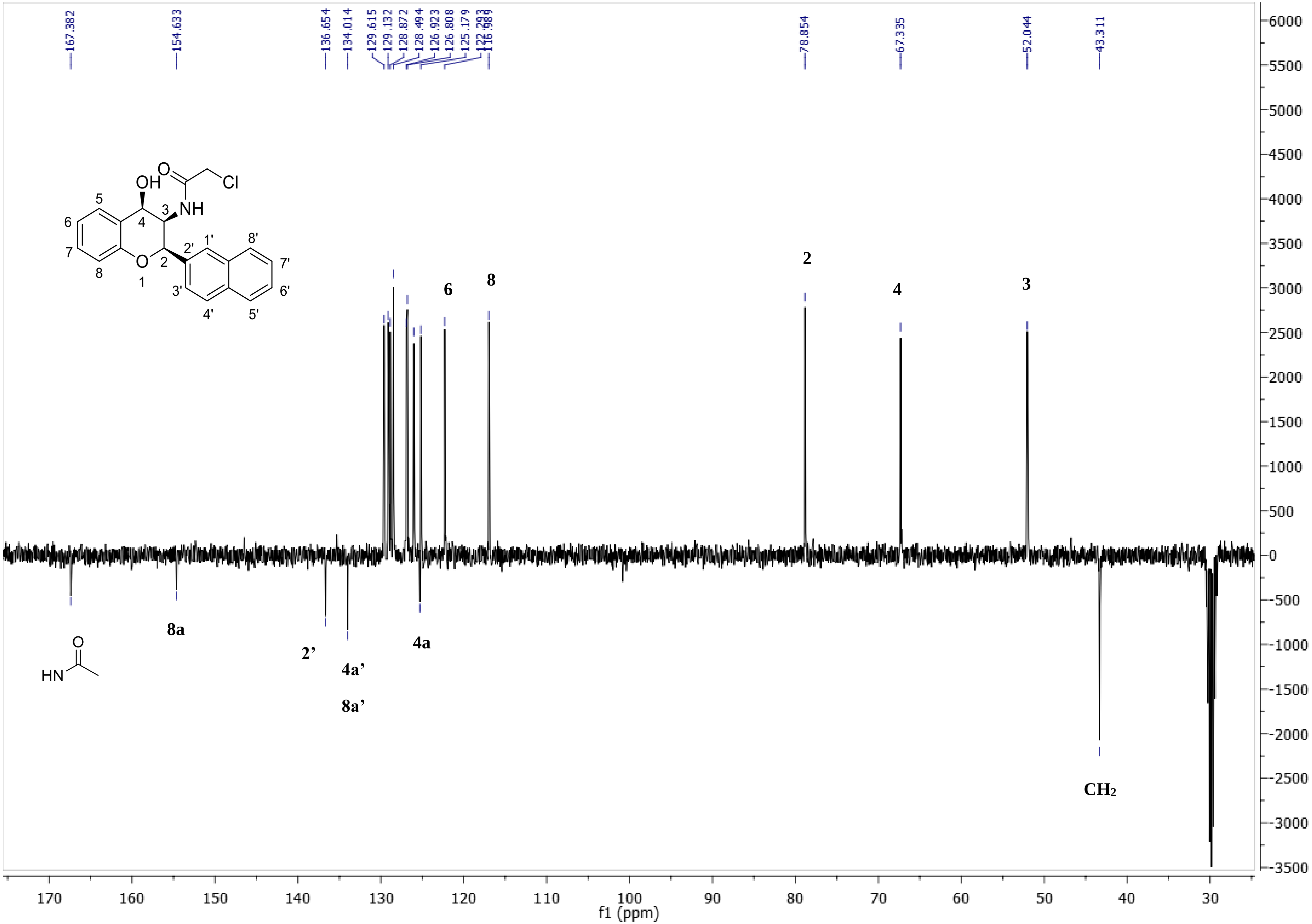

Figure S120. ^{13}C-NMR spectrum of *rac*-**22g** in Acetone-d_6

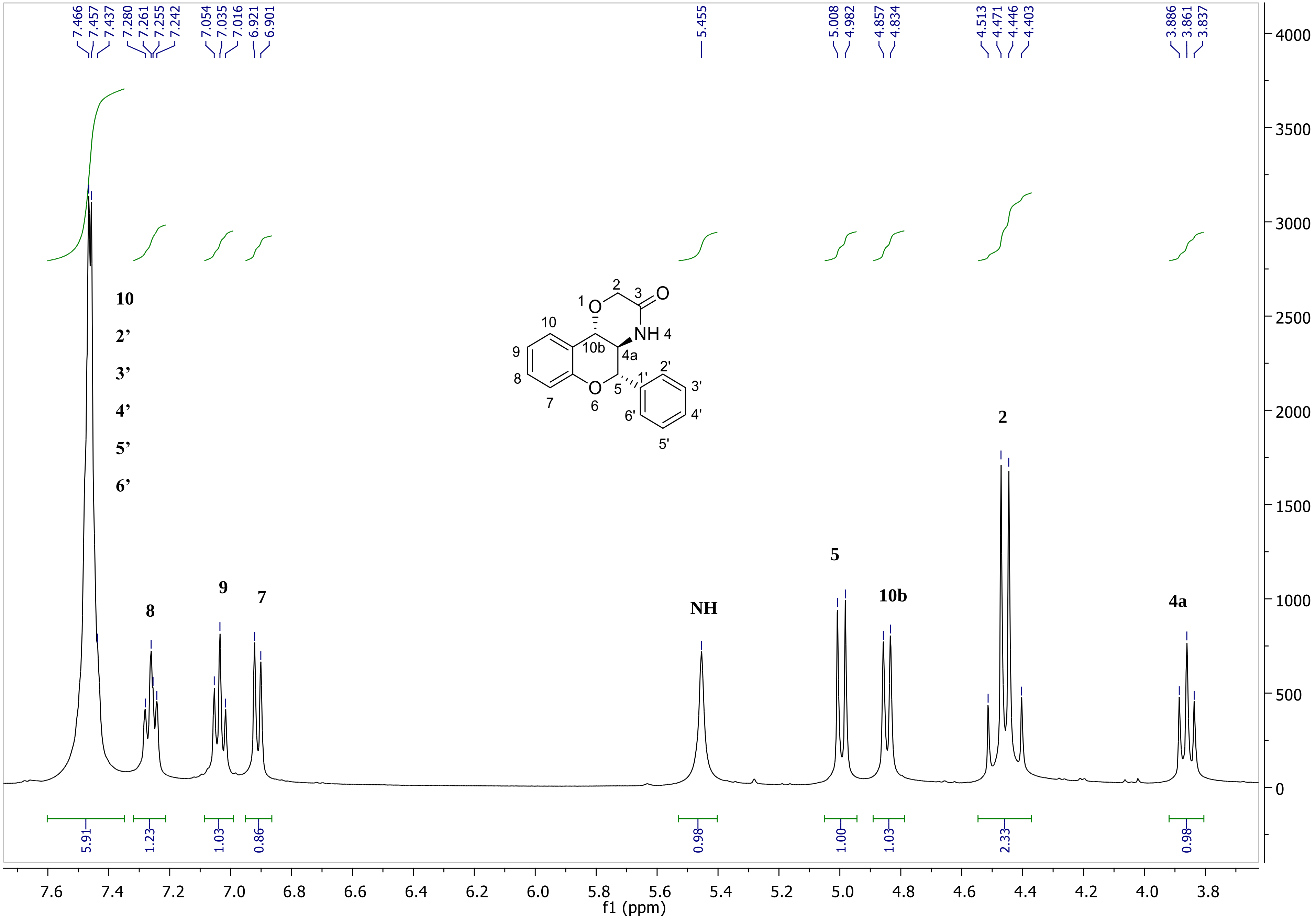

Figure S121. ^{1}H-NMR spectrum of *rac*-**20a** in $CDCl_3$

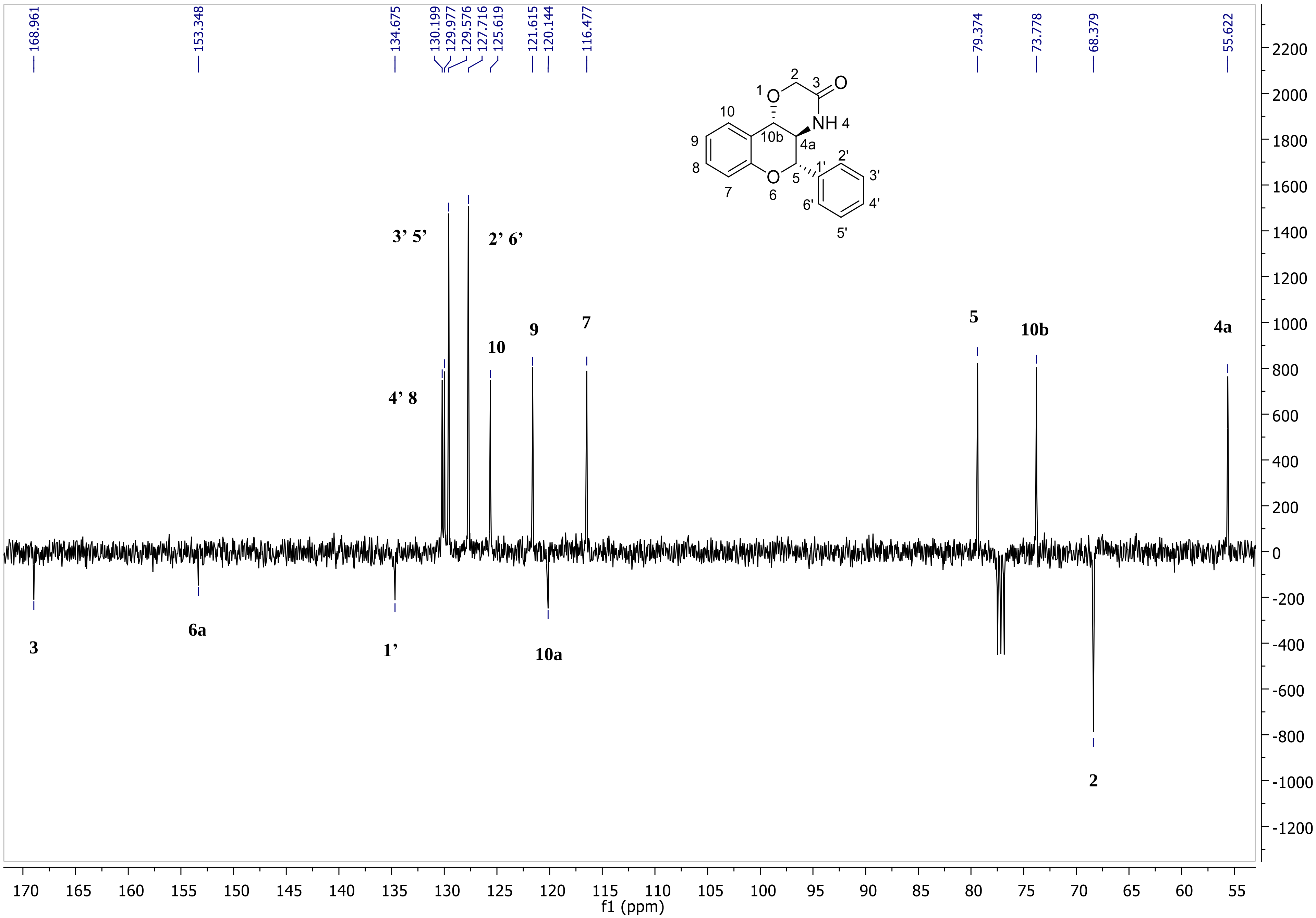

Figure S122. ^{13}C-NMR spectrum of *rac*-**20a** in $CDCl_3$

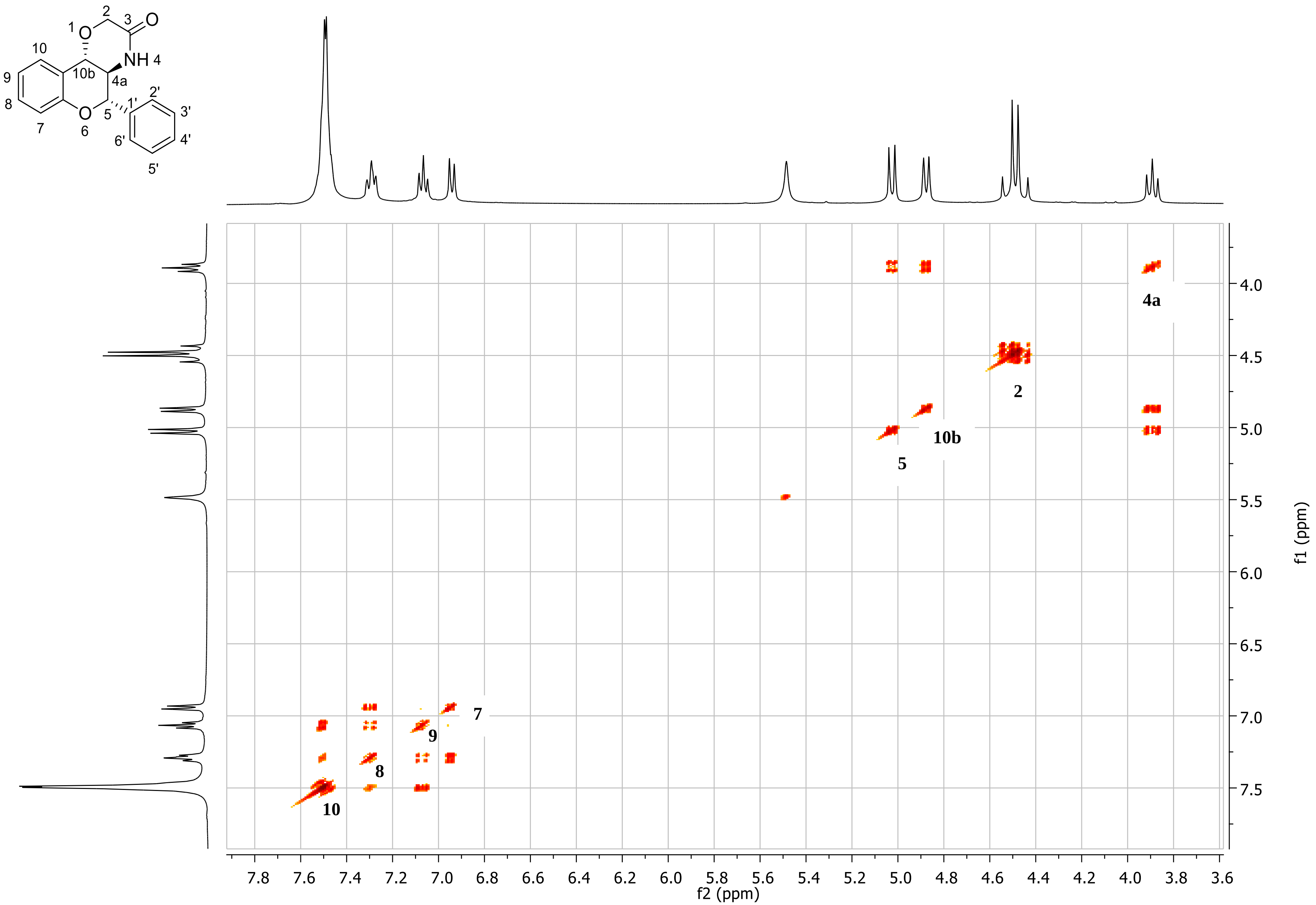

Figure S123. COSY spectrum of *rac*-**20a** in $CDCl_3$

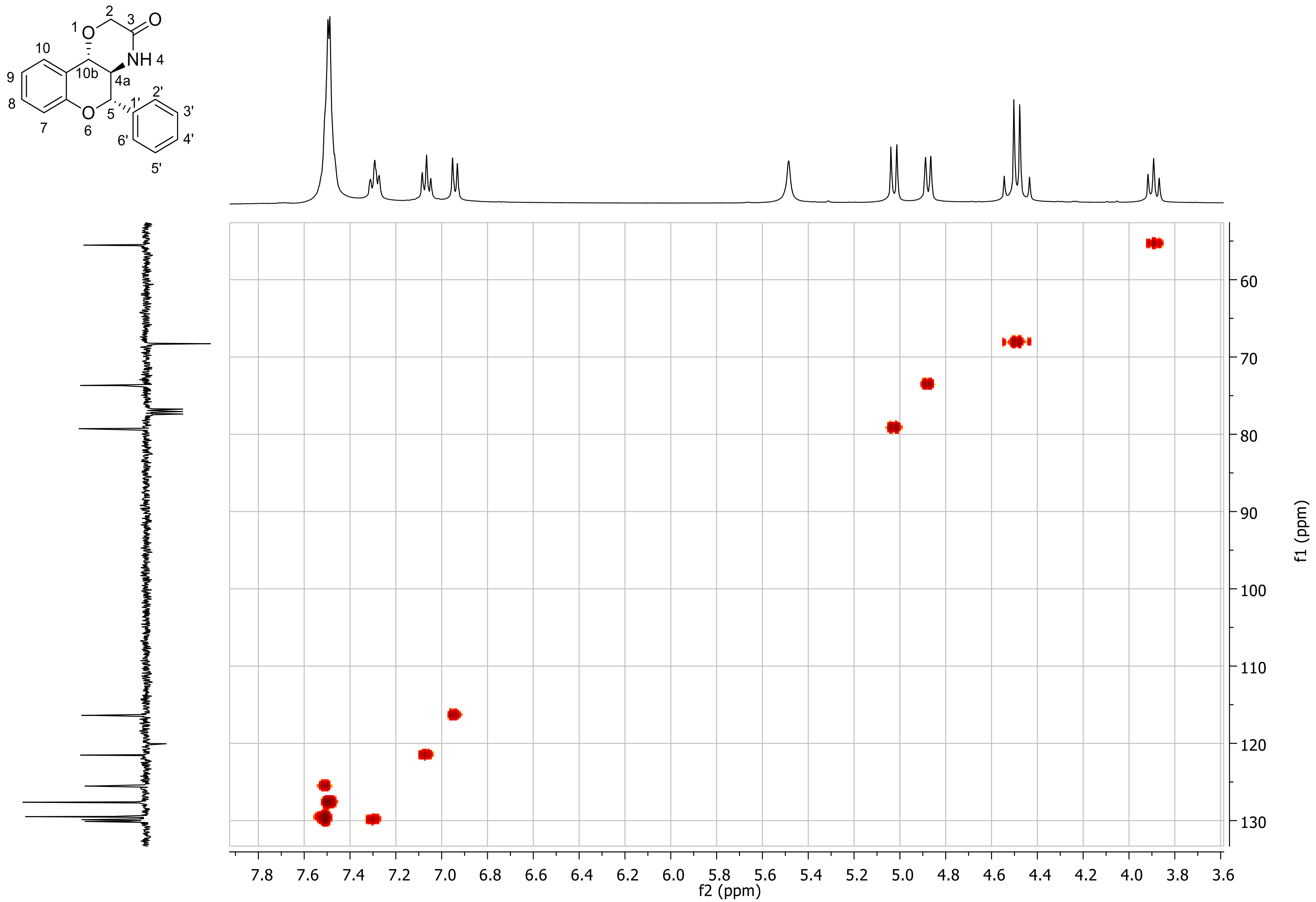

Figure S124. HSQC spectrum of *rac*-**20a** in $CDCl_3$

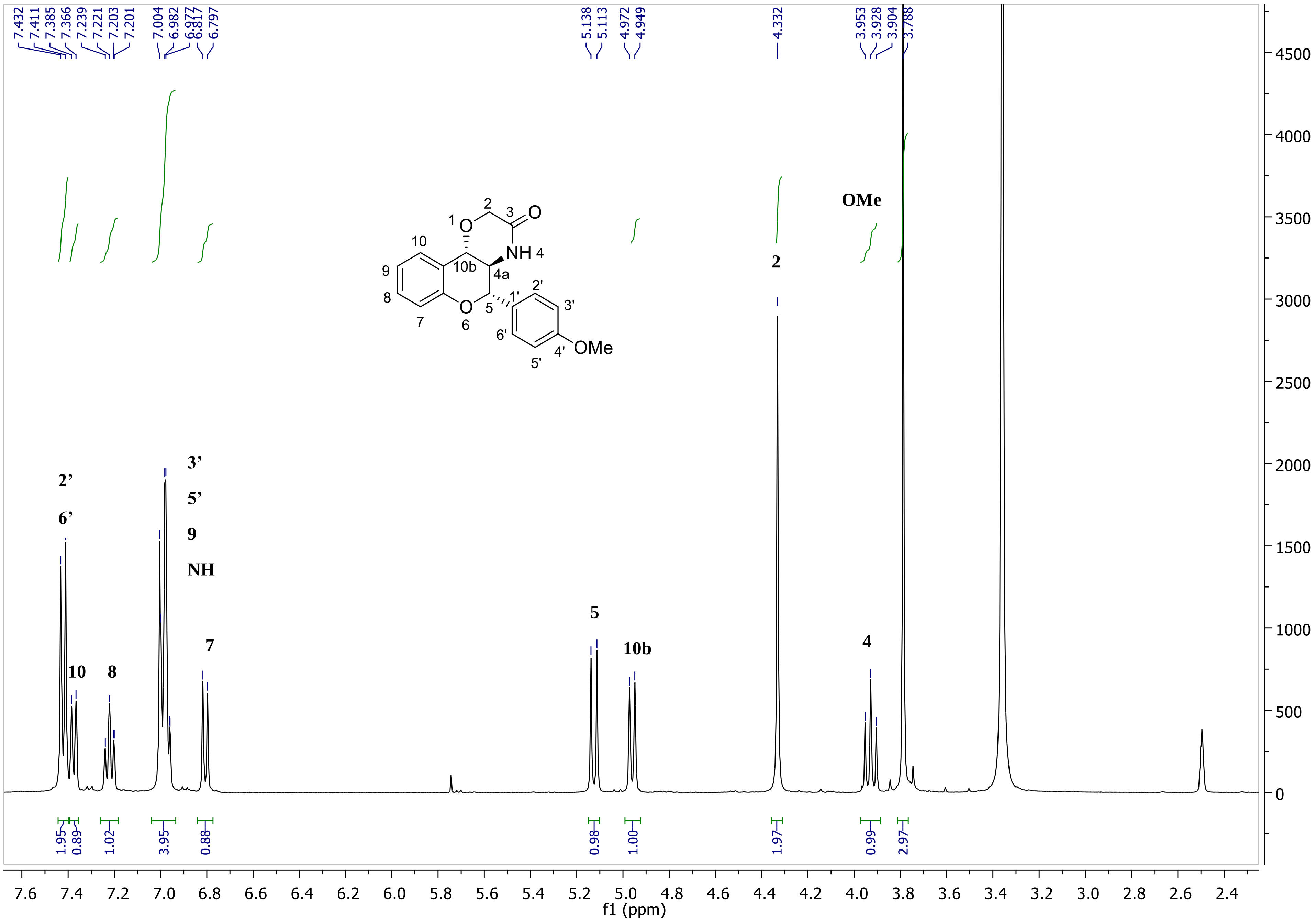

Figure S125. ^{1}H-NMR spectrum of *rac*-**20b** in DMSO-d$_6$

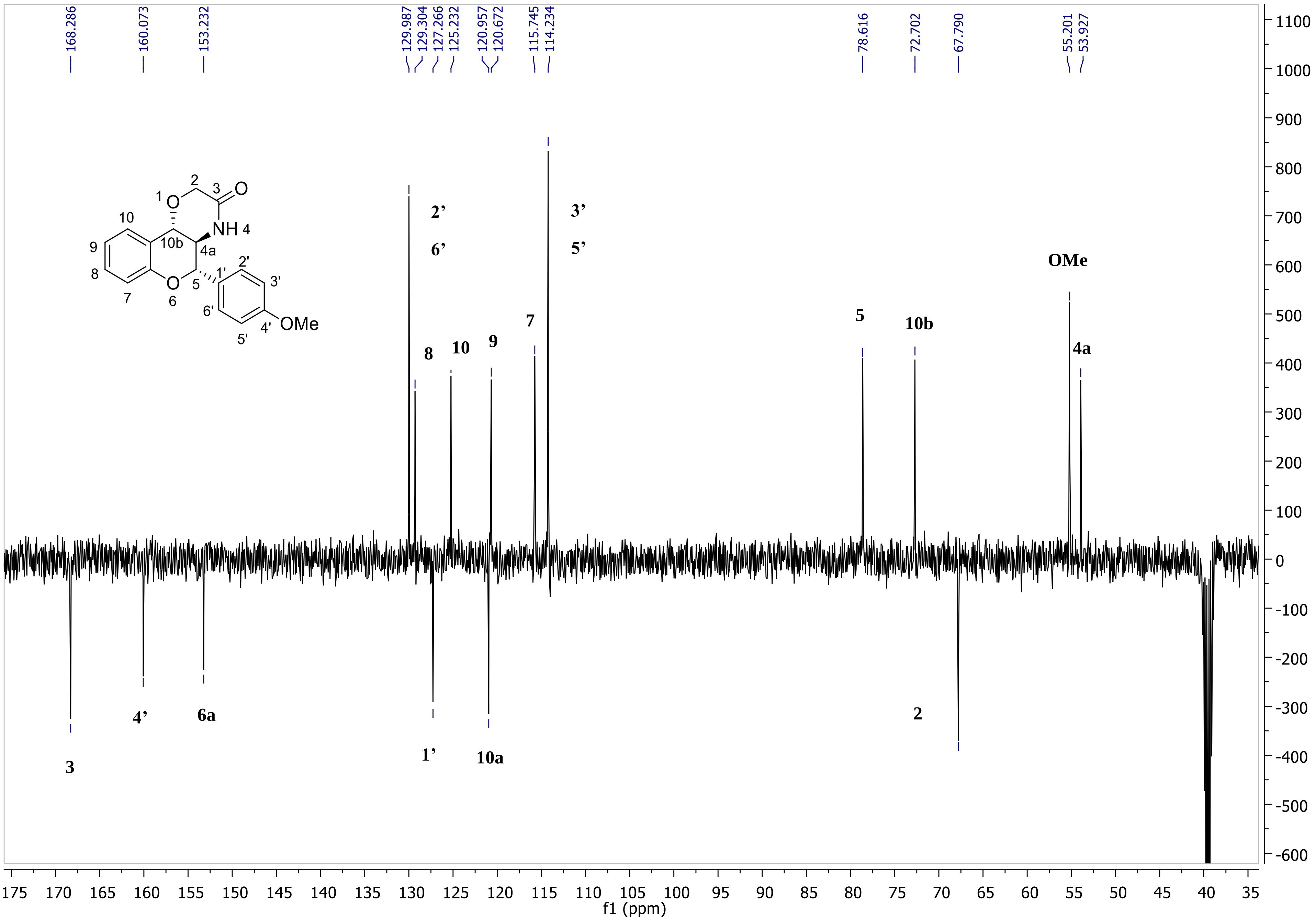

Figure S126. ^{13}C-NMR spectrum of *rac*-**20b** in DMSO-d_6

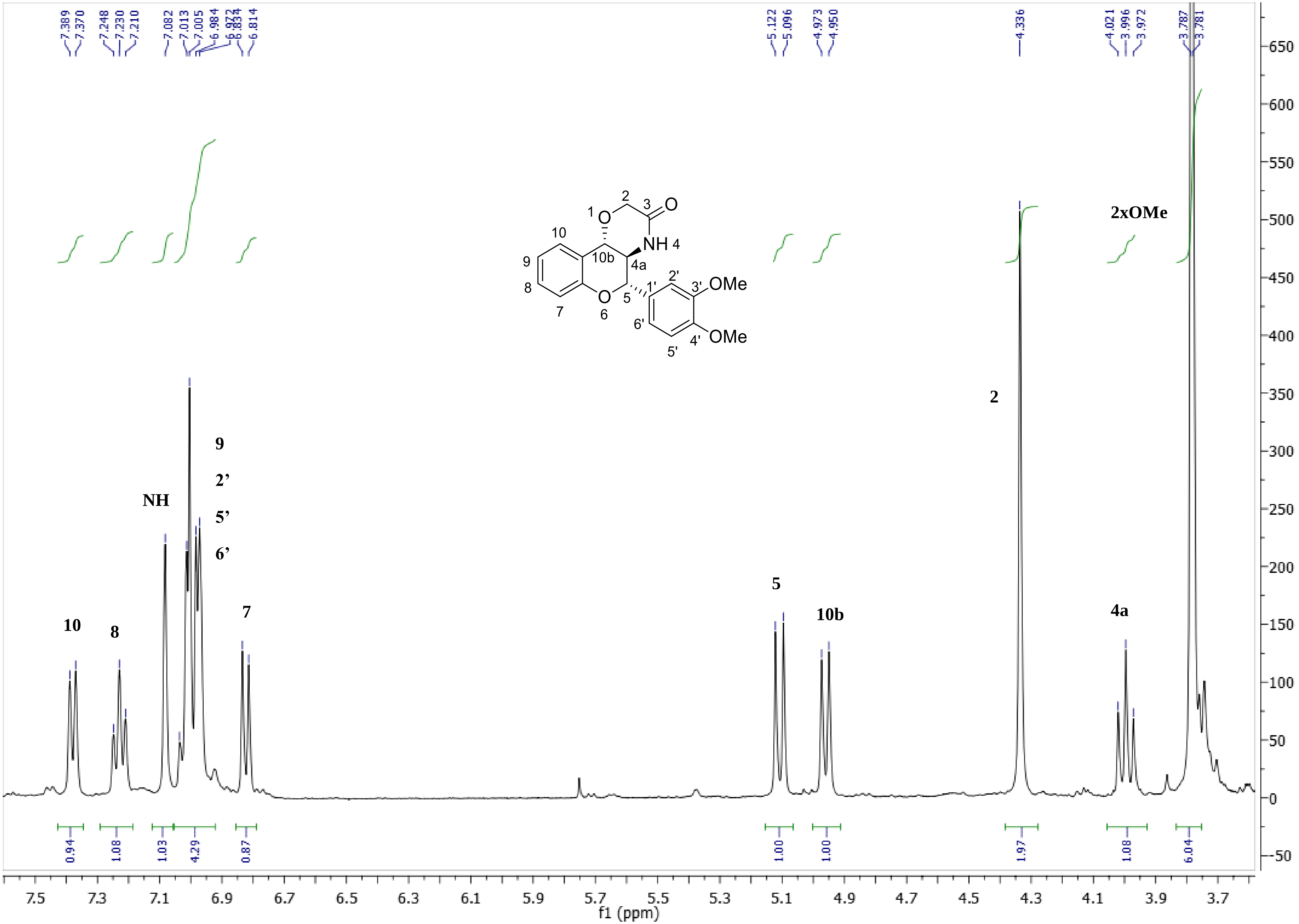

Figure S127. ^{1}H-NMR spectrum of *rac*-**20c** in DMSO-d_6

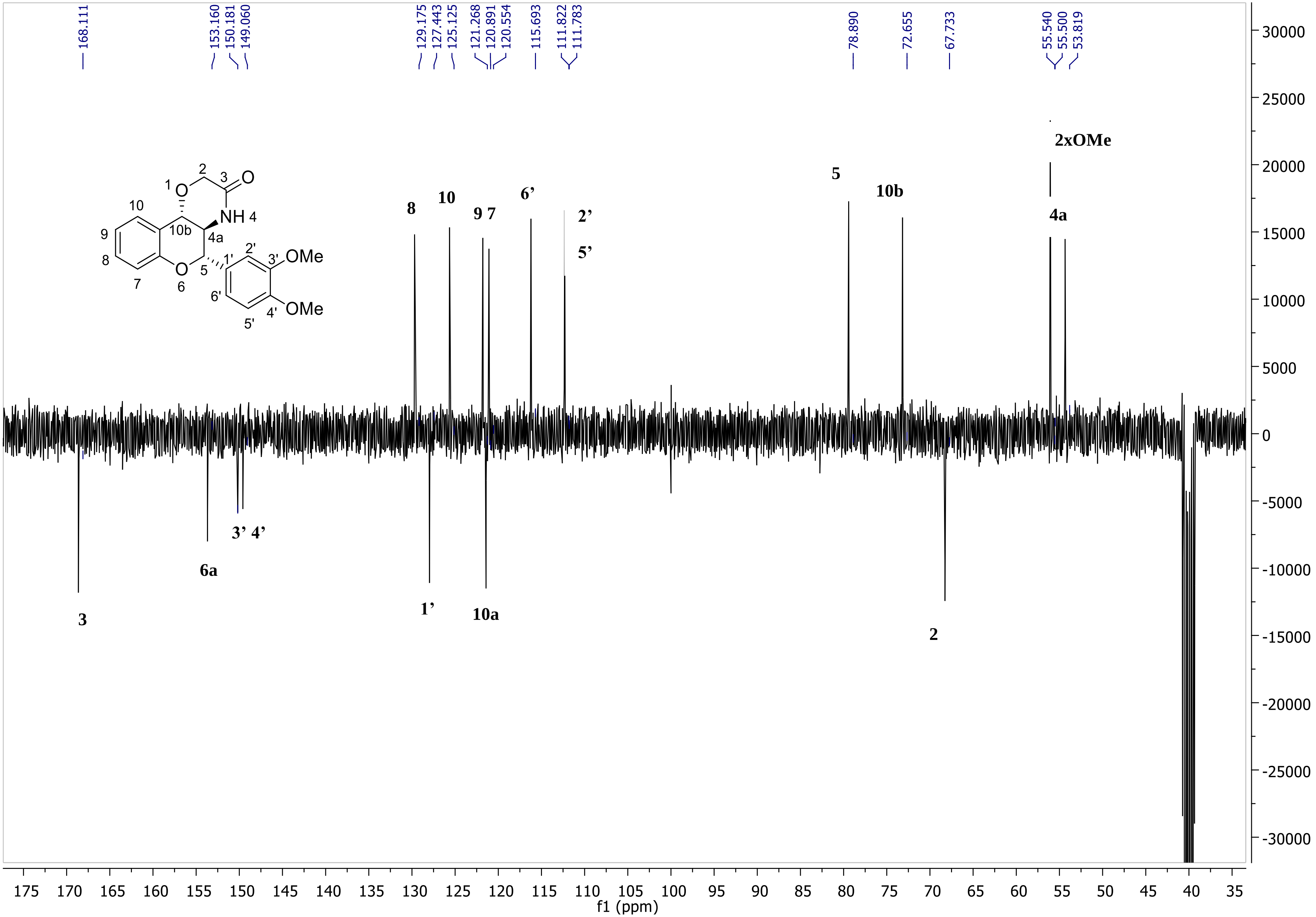

Figure S128. ^{13}C-NMR spectrum of *rac*-**20c** in DMSO-d$_6$

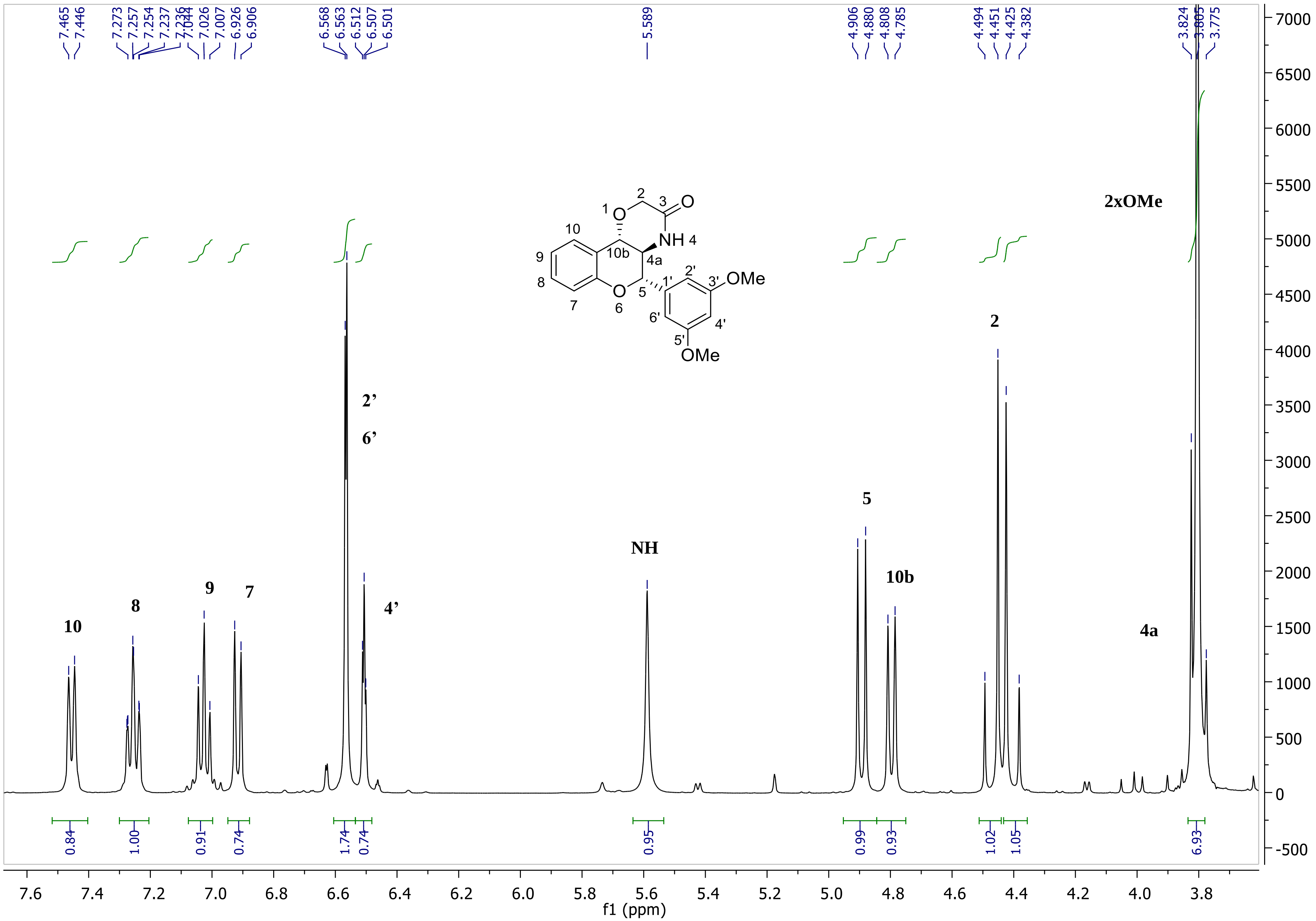

Figure S129. ^{1}H-NMR spectrum of *rac*-**20d** in $CDCl_3$

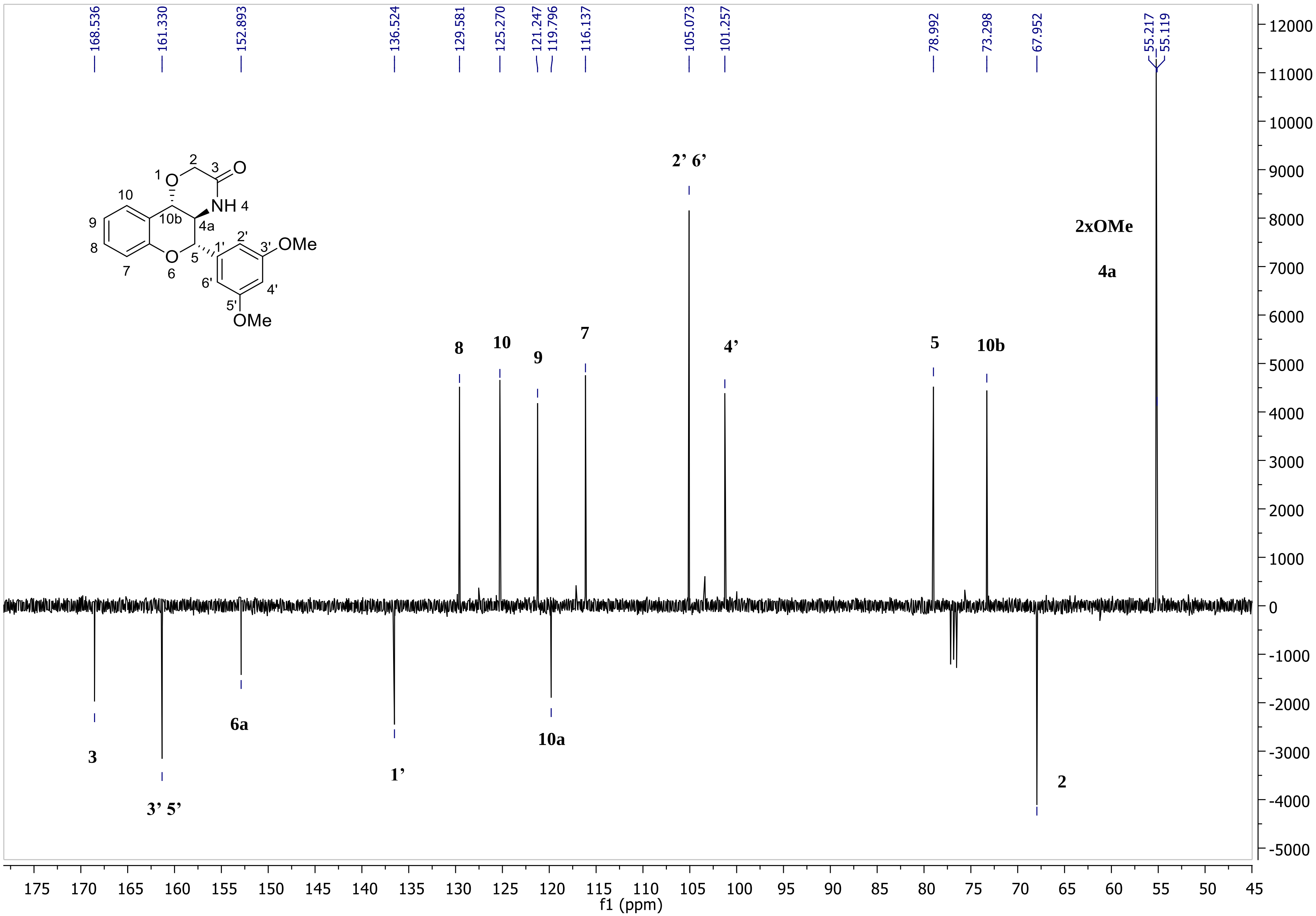

Figure S130. ^{13}C-NMR spectrum of *rac*-**20d** in $CDCl_3$

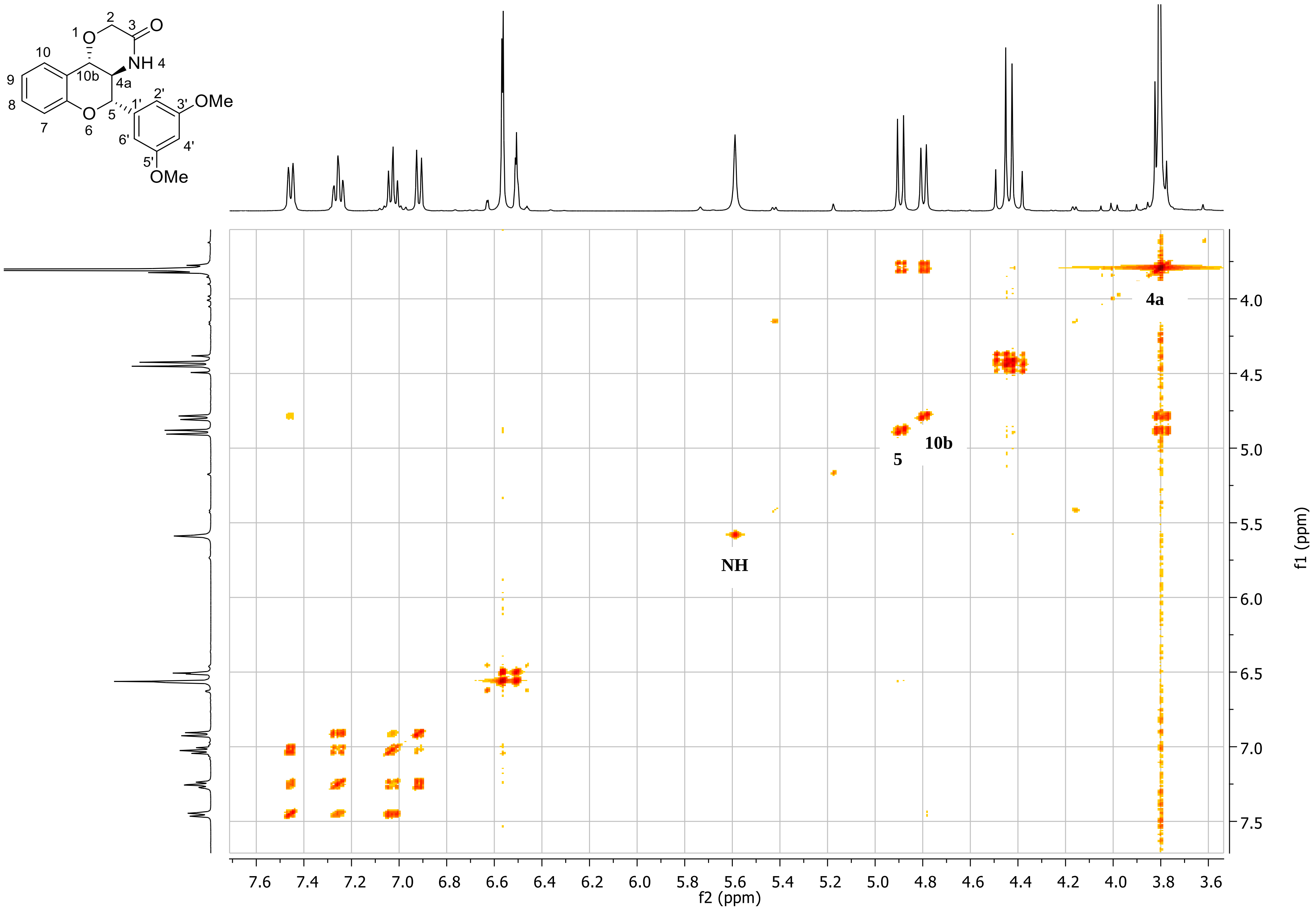

Figure S131. COSY-spectrum of *rac*-**20d** in $CDCl_3$

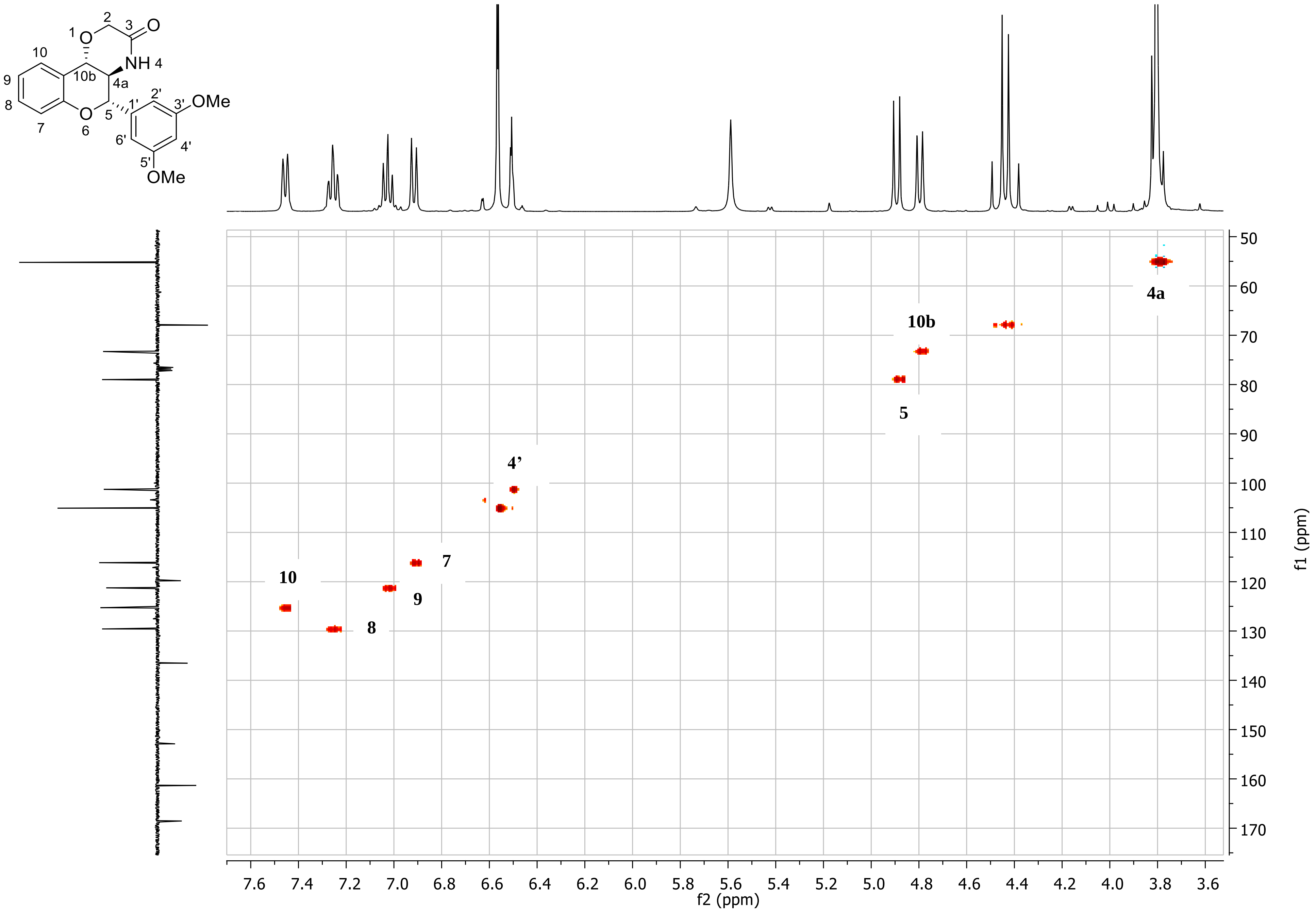

Figure S132. COSY-spectrum of *rac*-**20d** in $CDCl_3$

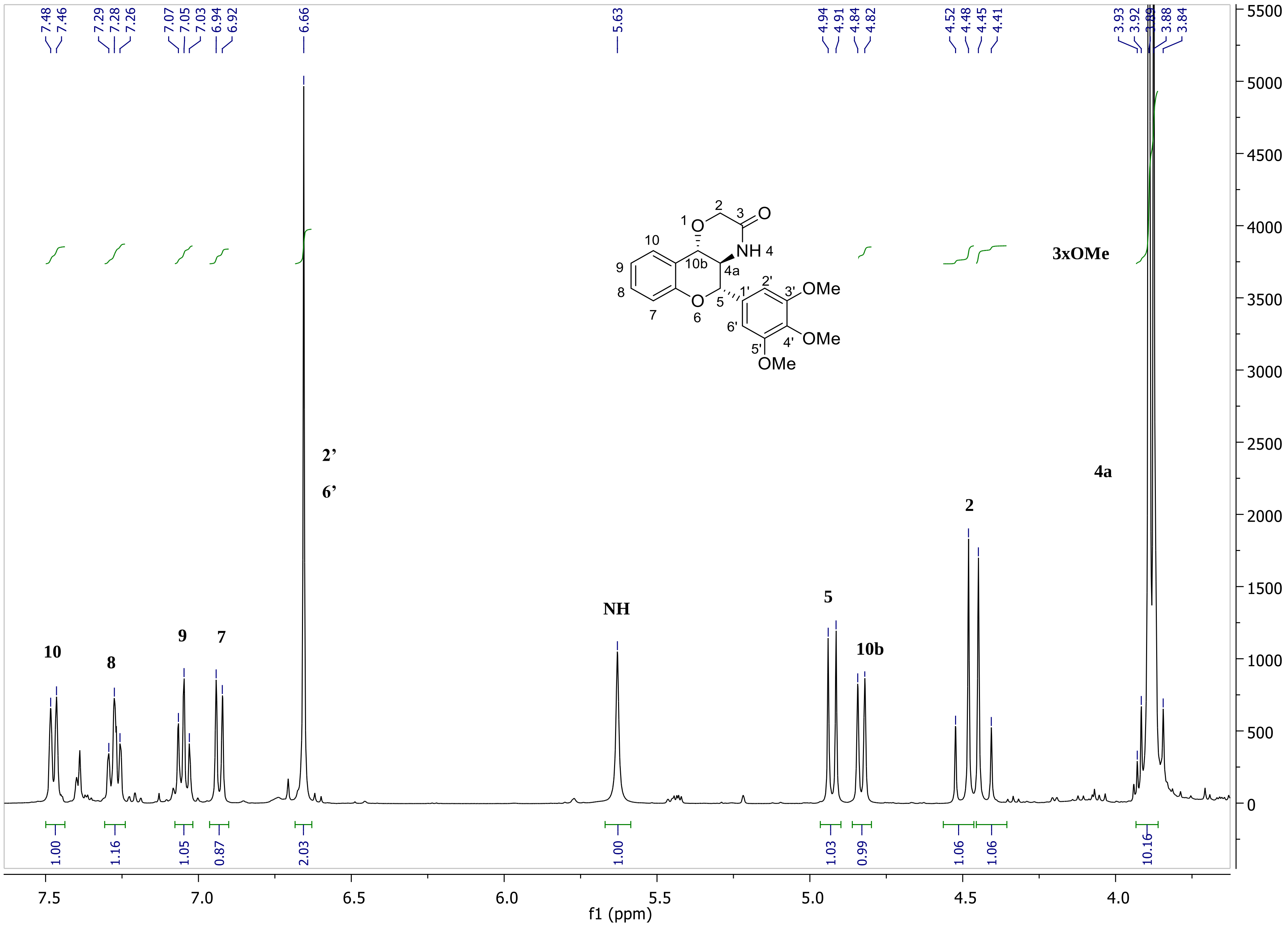

Figure S133. ^{1}H-NMR spectrum of *rac*-**20e** in $CDCl_3$

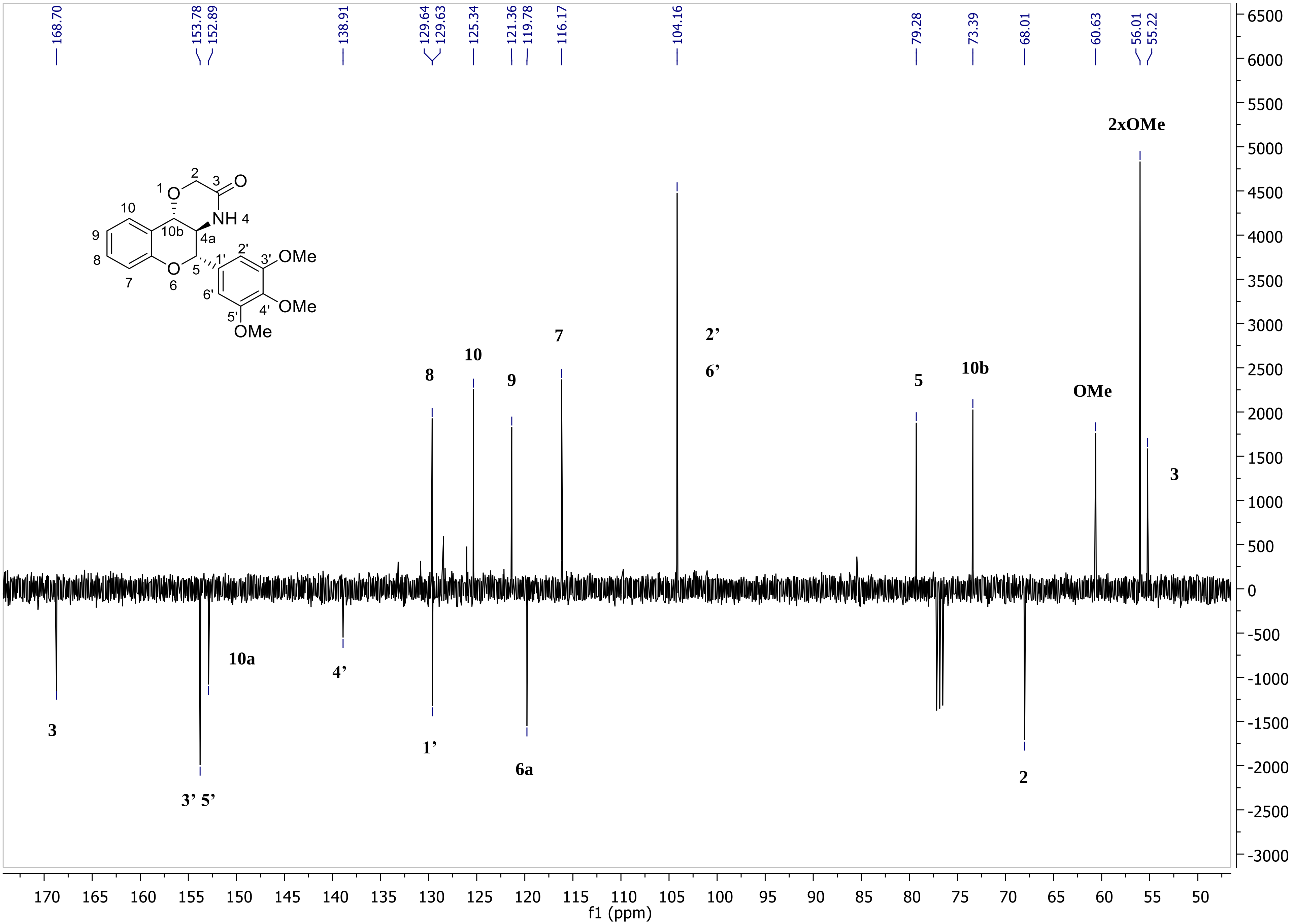

Figure S134. ^{13}C-NMR spectrum of *rac*-**20e** in $CDCl_3$

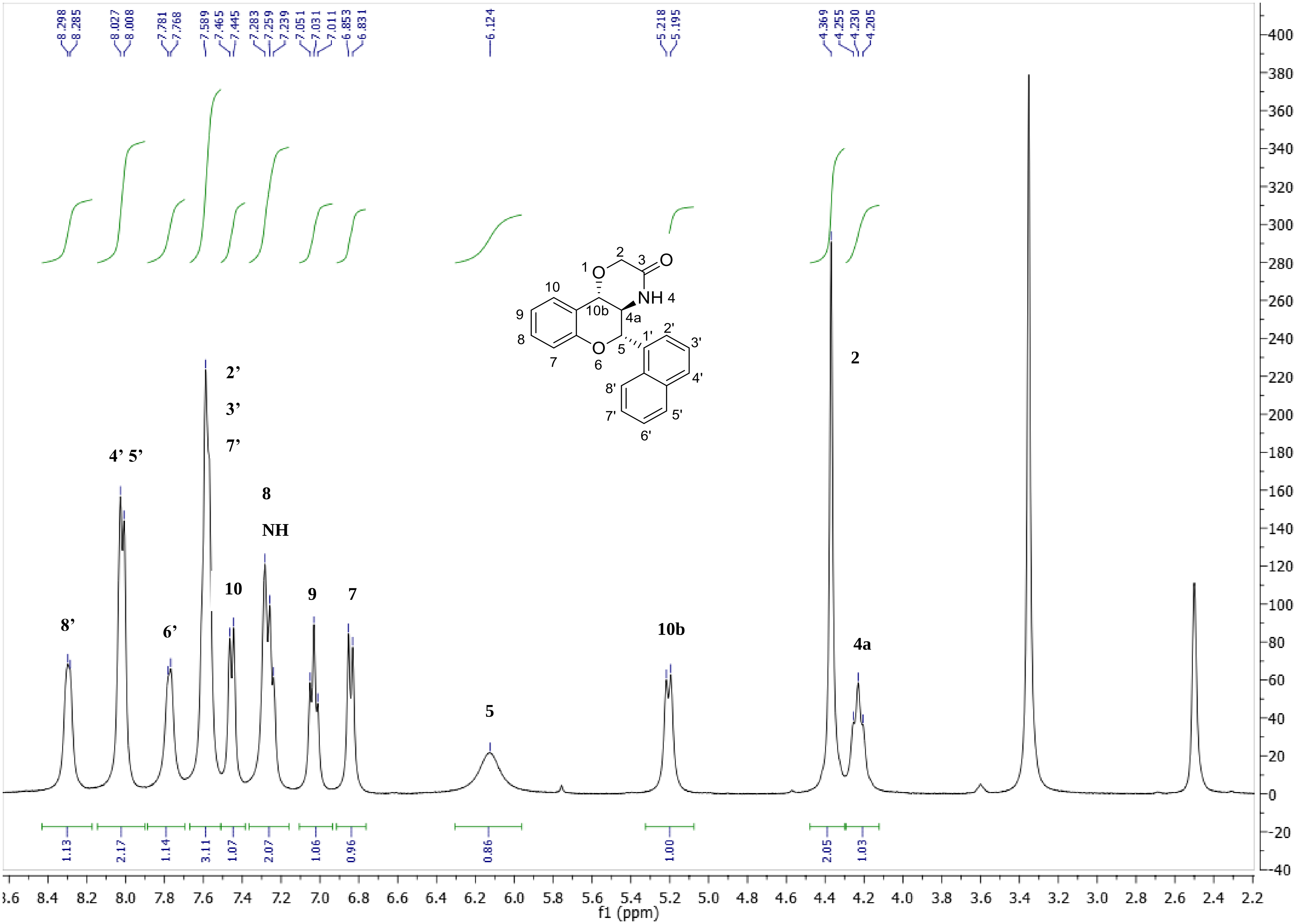

Figure S135. ^{1}H-NMR spectrum of *rac*-**20f** in DMSO-d_6

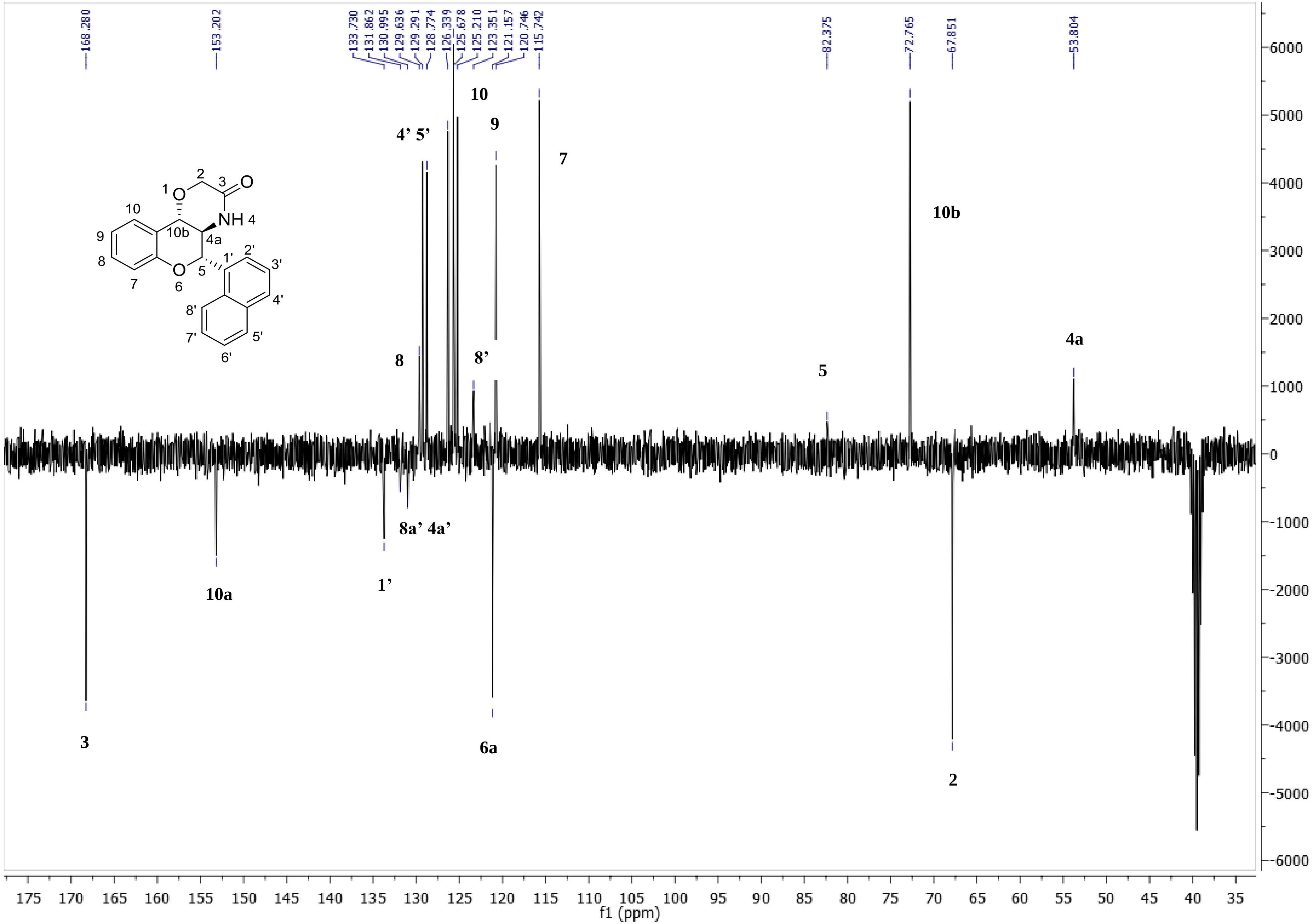

Figure S136. ^{13}C-NMR spectrum of *rac*-**20f** in DMSO-d_6

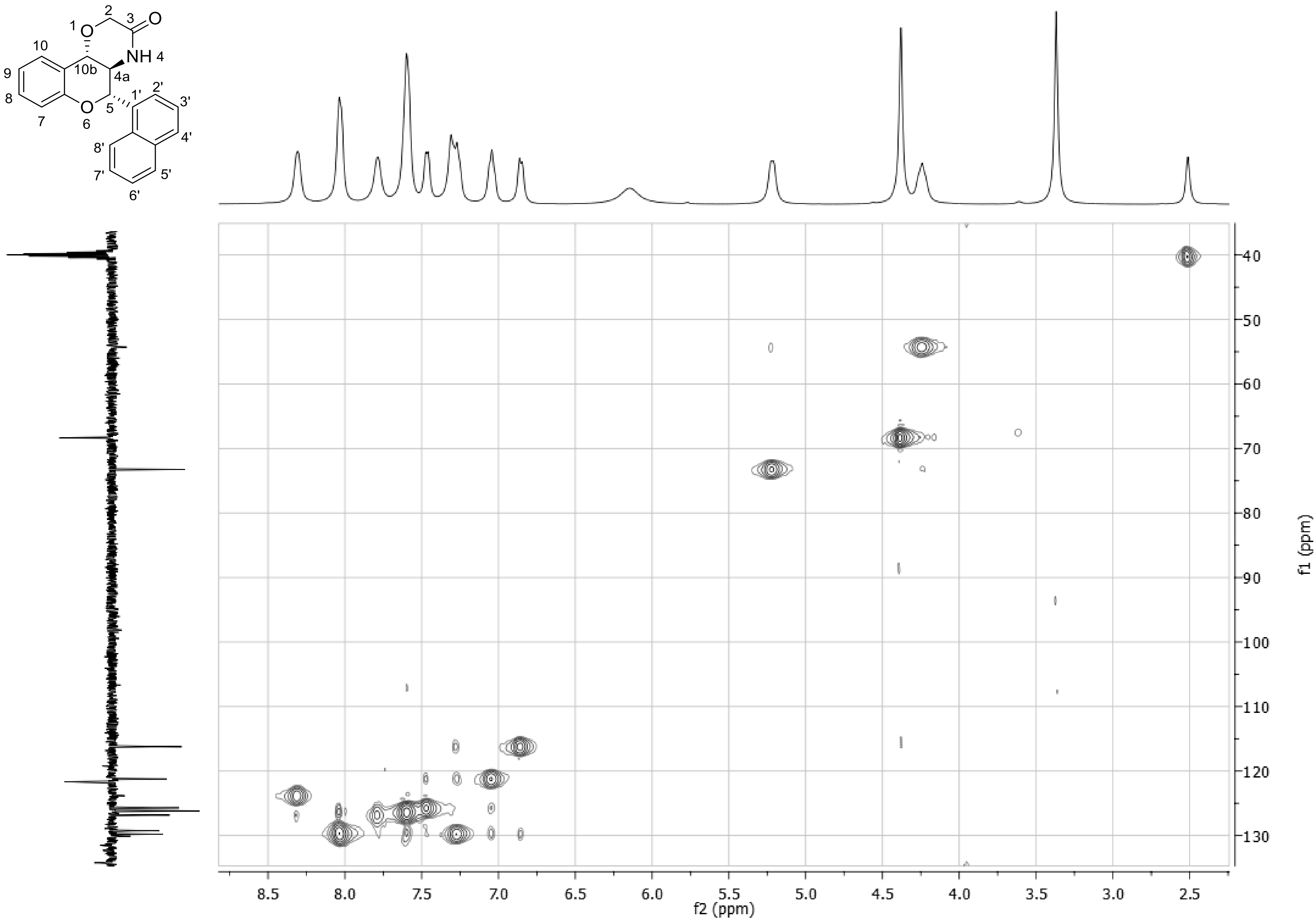

Figure S137. HSQC spectrum of *rac*-**20f** in DMSO-d_6

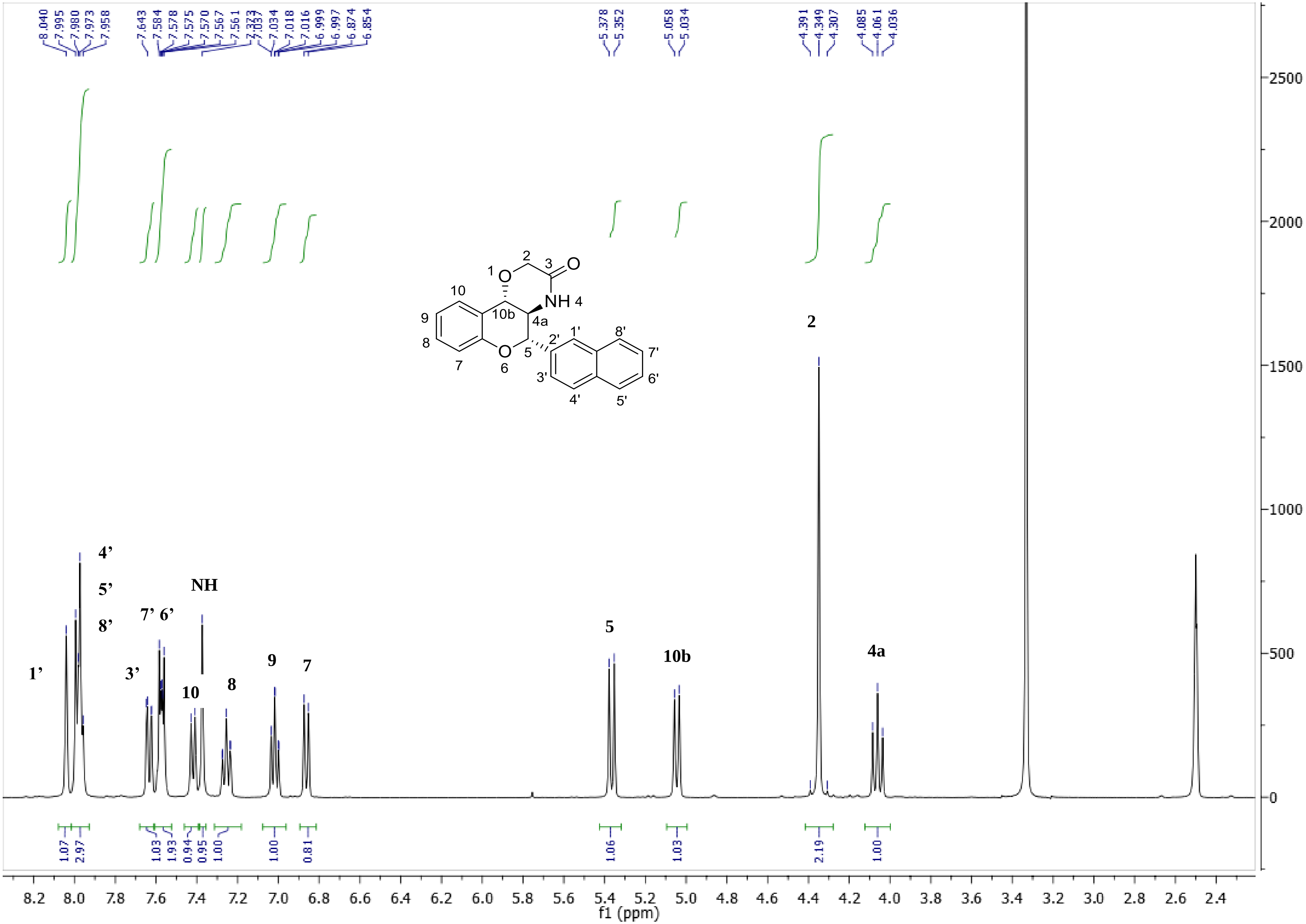

Figure S138. ^{1}H-NMR spectrum of *rac*-**20g** in DMSO-d_6

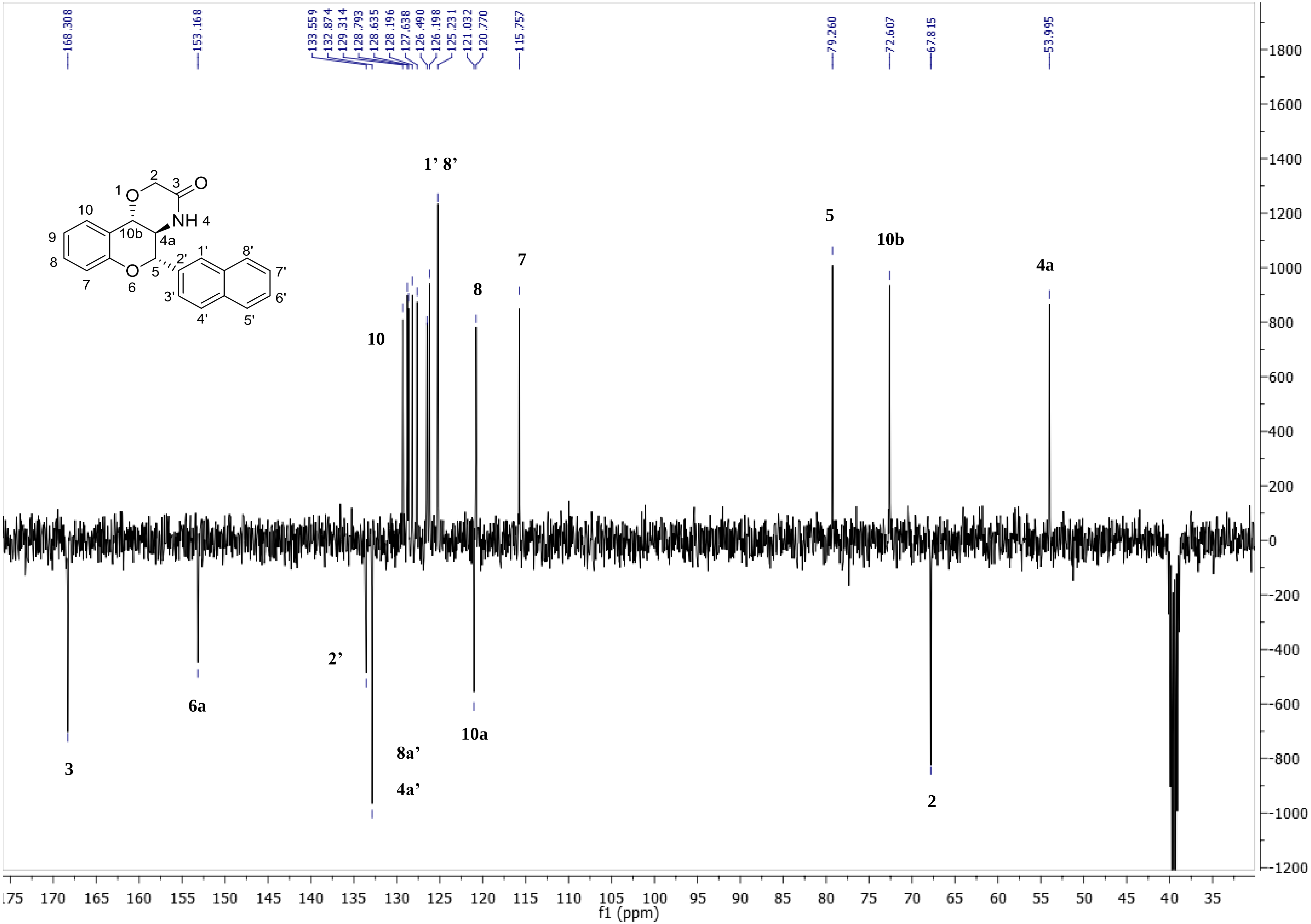

Figure S139. ^{13}C-NMR spectrum of *rac*-**20g** in DMSO-d_6

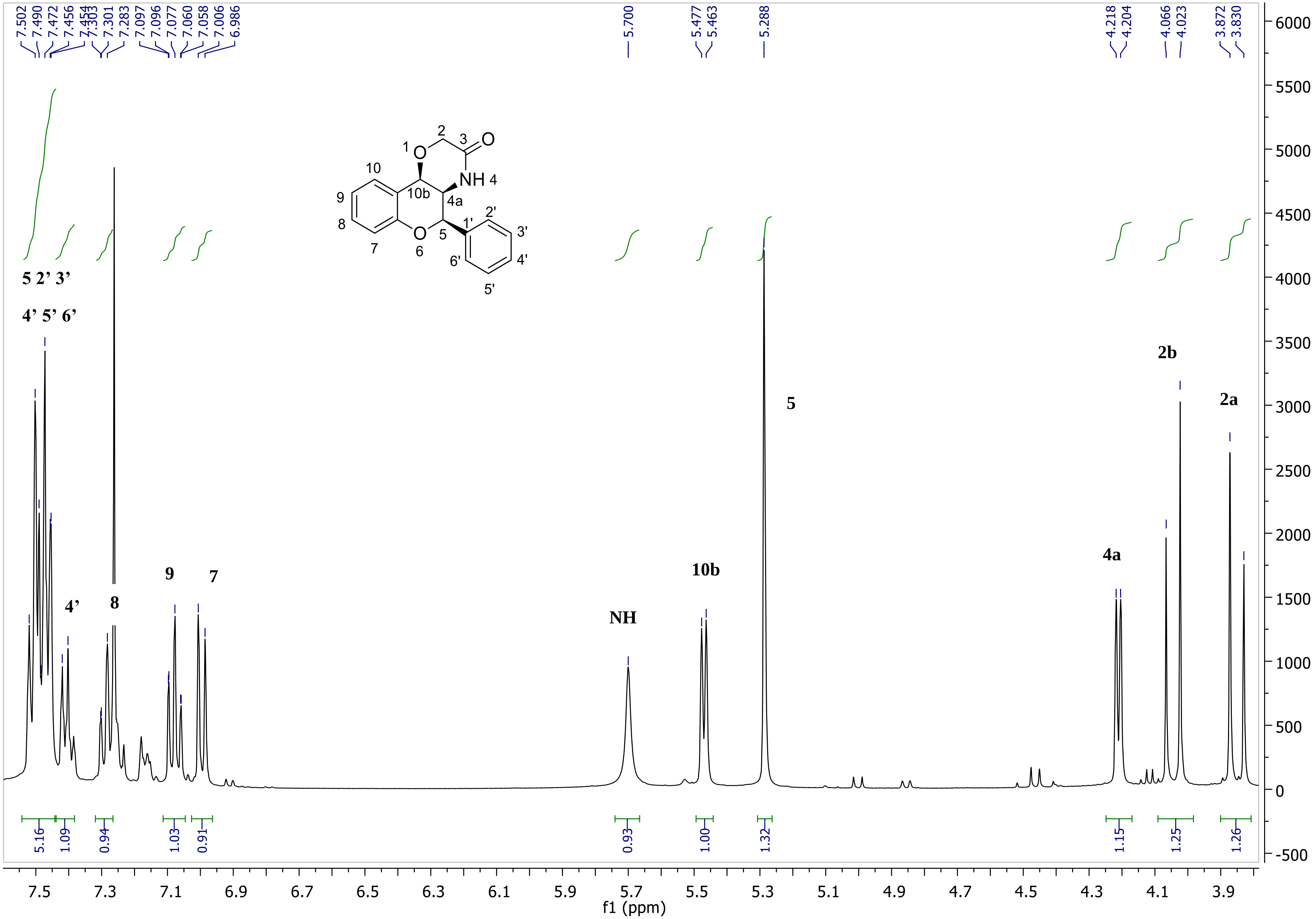

Figure S140. ^{1}H-NMR spectrum of *rac*-**23a** in $CDCl_3$

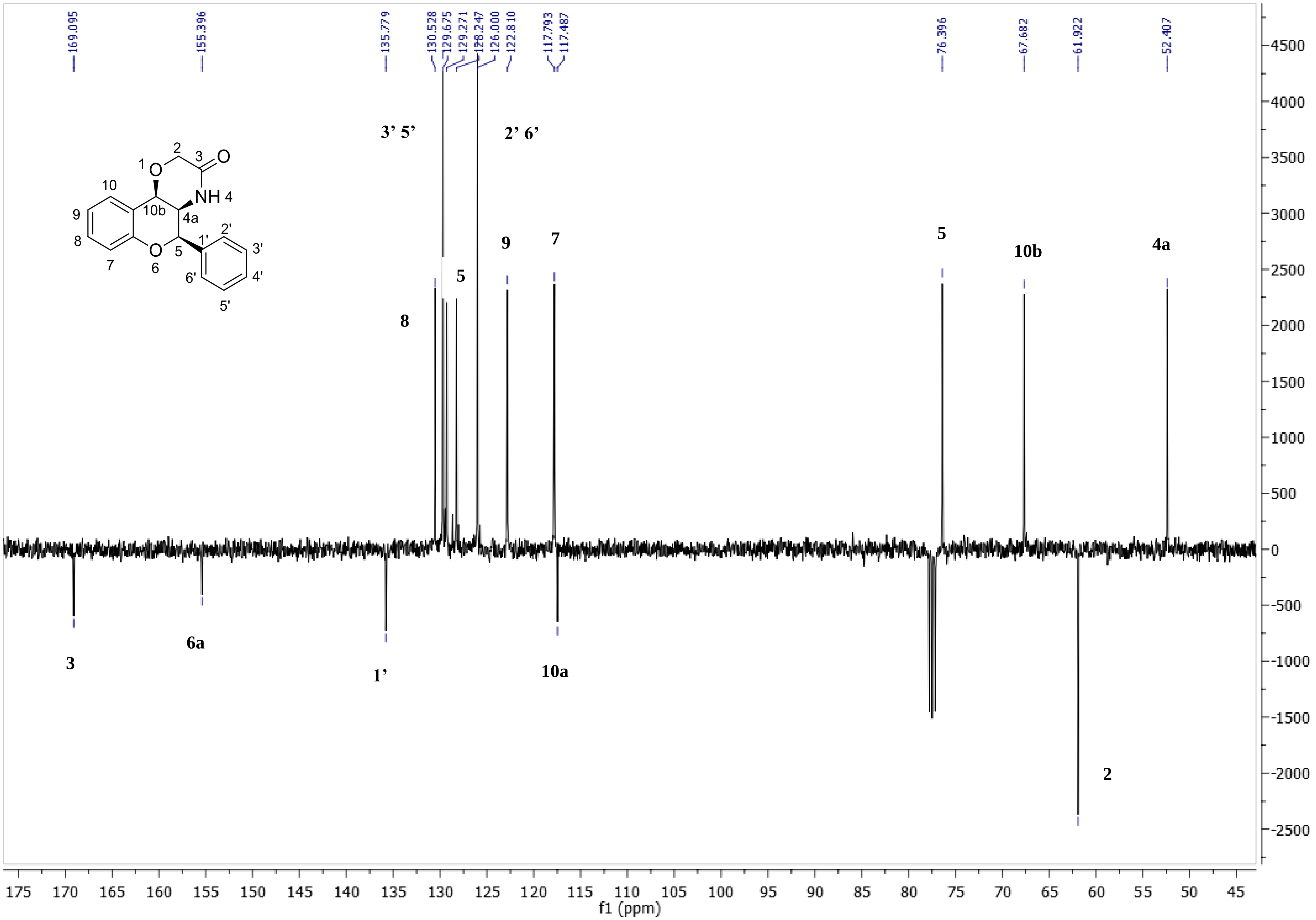

Figure S141. ^{13}C-NMR spectrum of *rac*-**23a** in $CDCl_3$

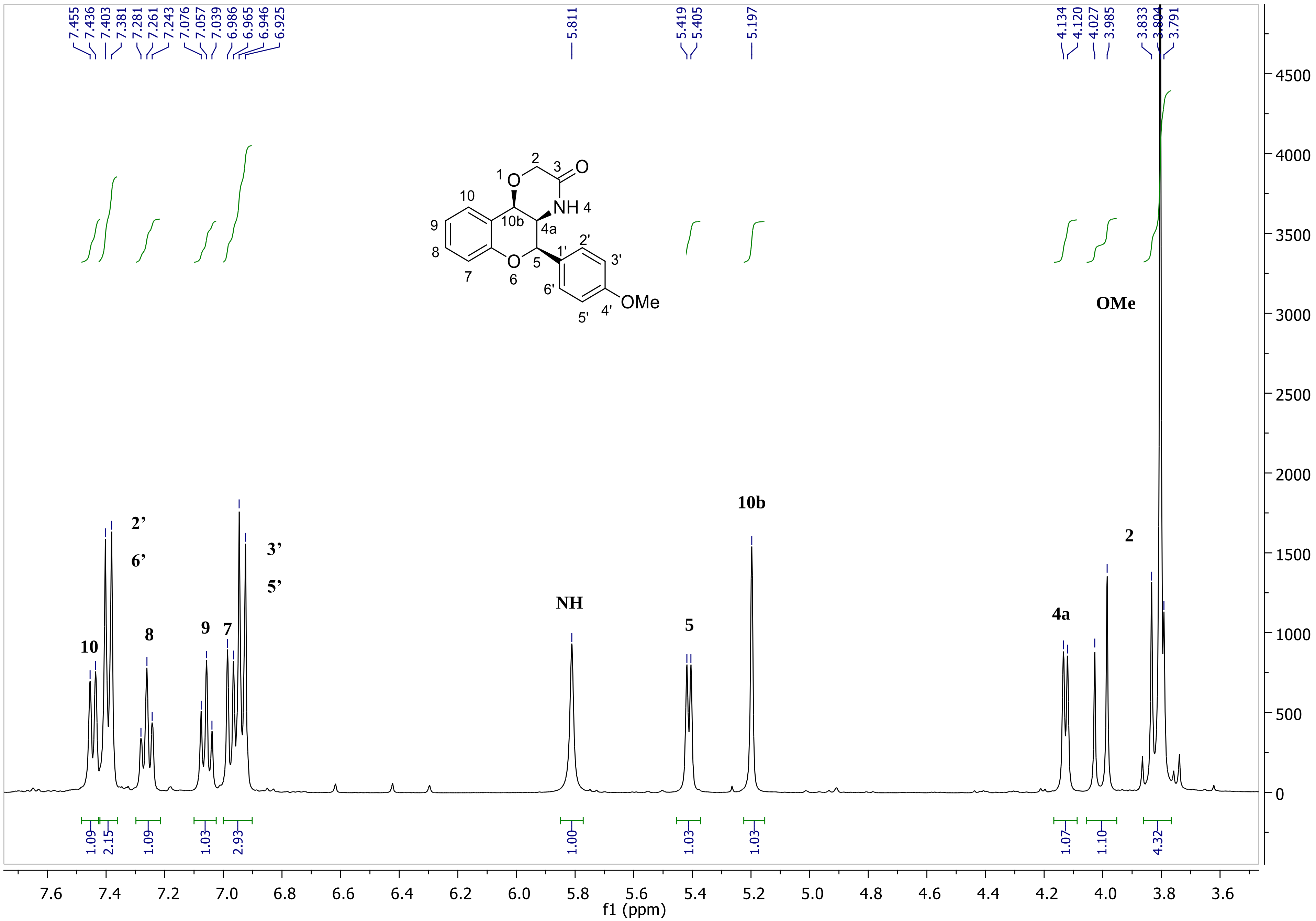

Figure S142. ^{1}H-NMR spectrum of *rac*-**23b** in $CDCl_3$

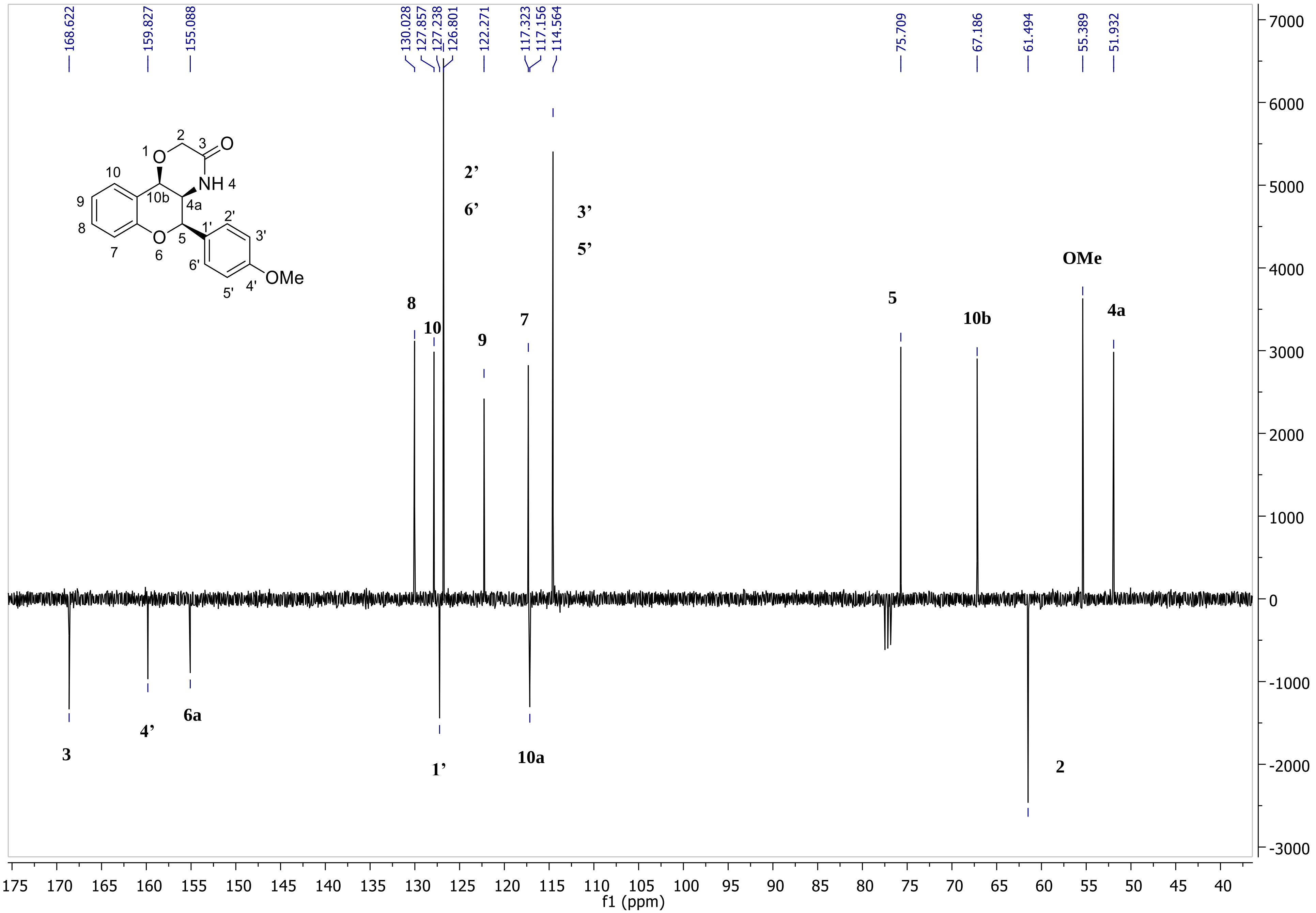

Figure S143. ^{13}C-NMR spectrum of *rac*-**23b** in $CDCl_3$

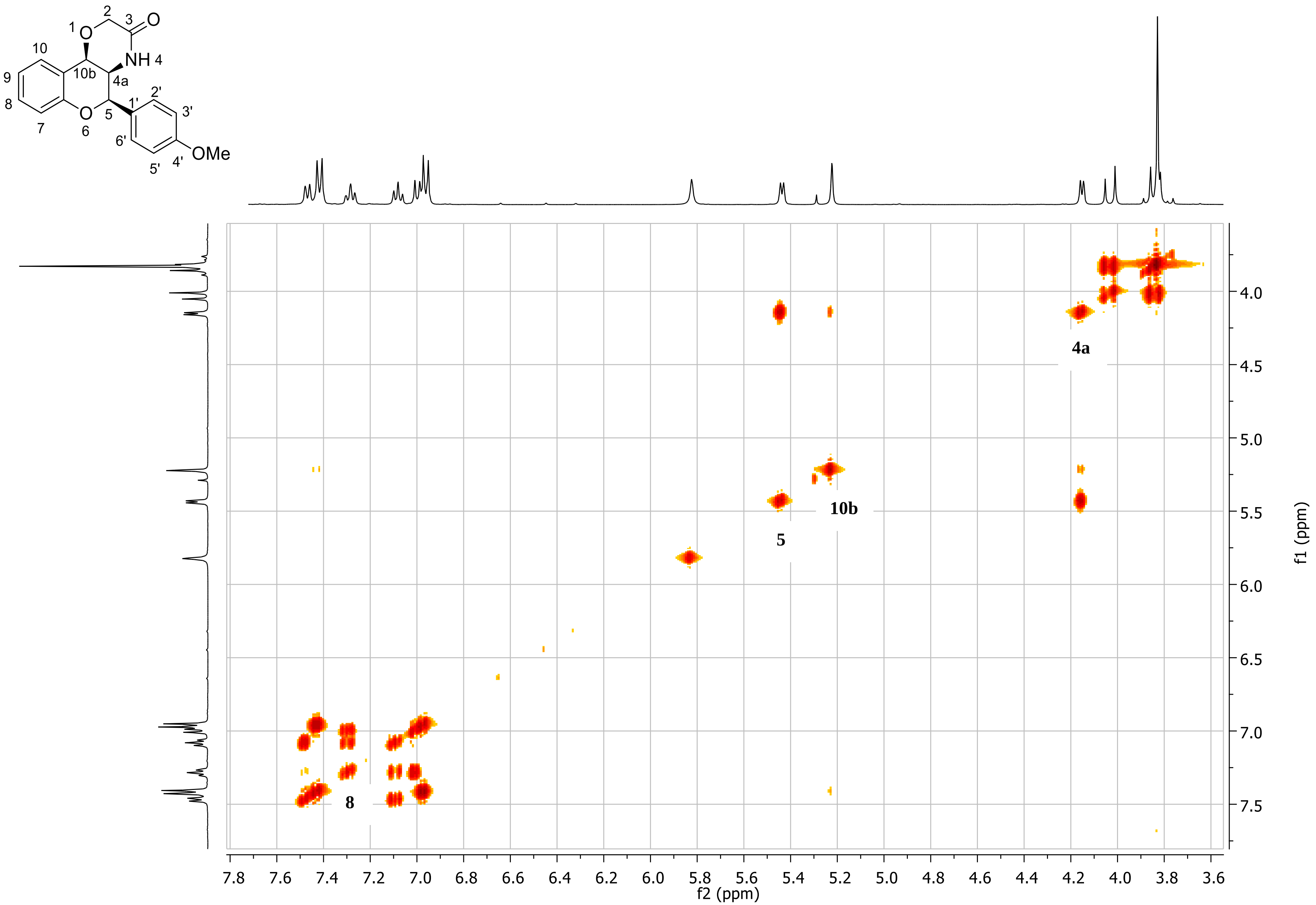

Figure S144. COSY spectrum of *rac*-**23b** in $CDCl_3$

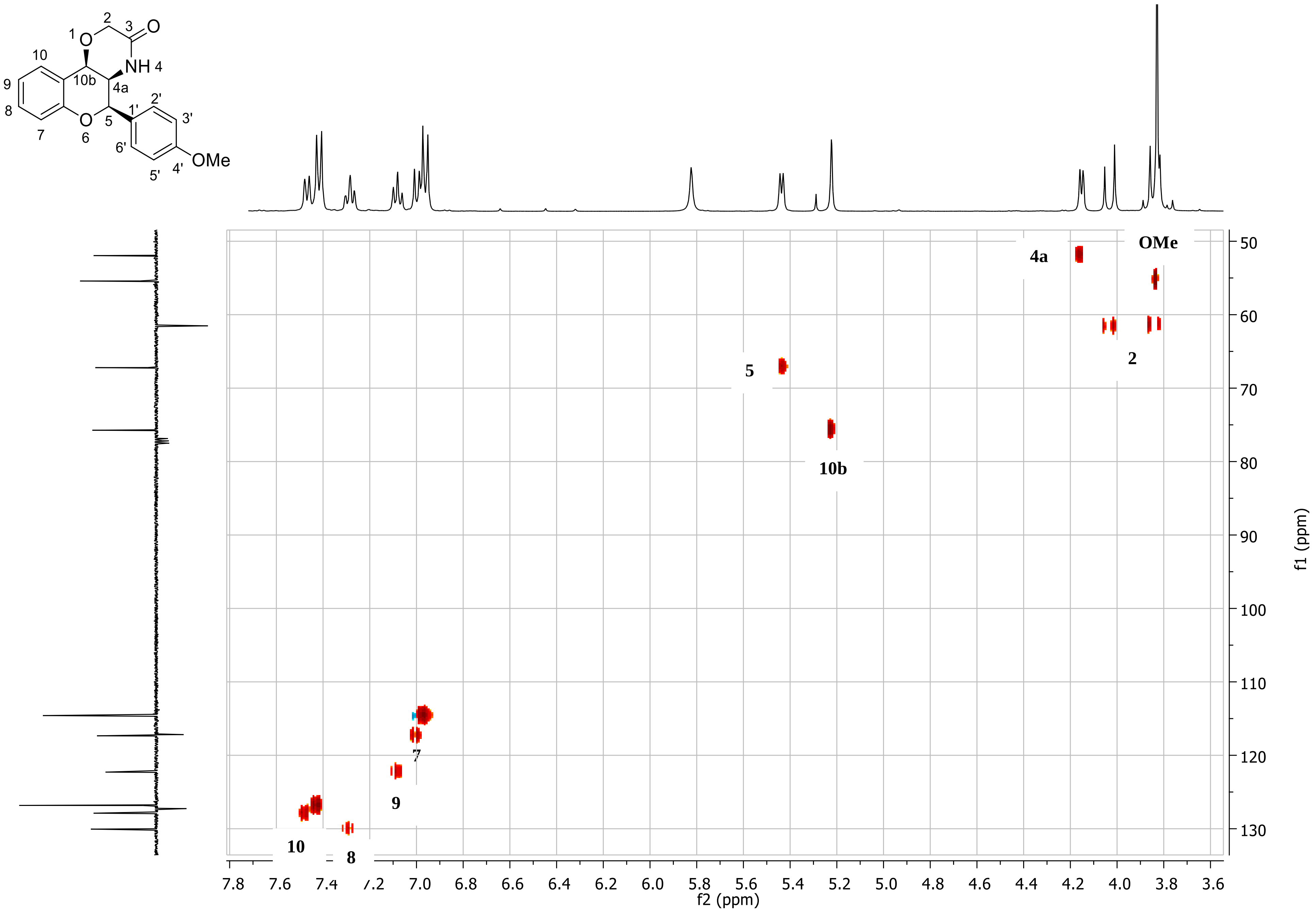

Figure S145. HSQC spectrum of *rac*-**23b** in $CDCl_3$

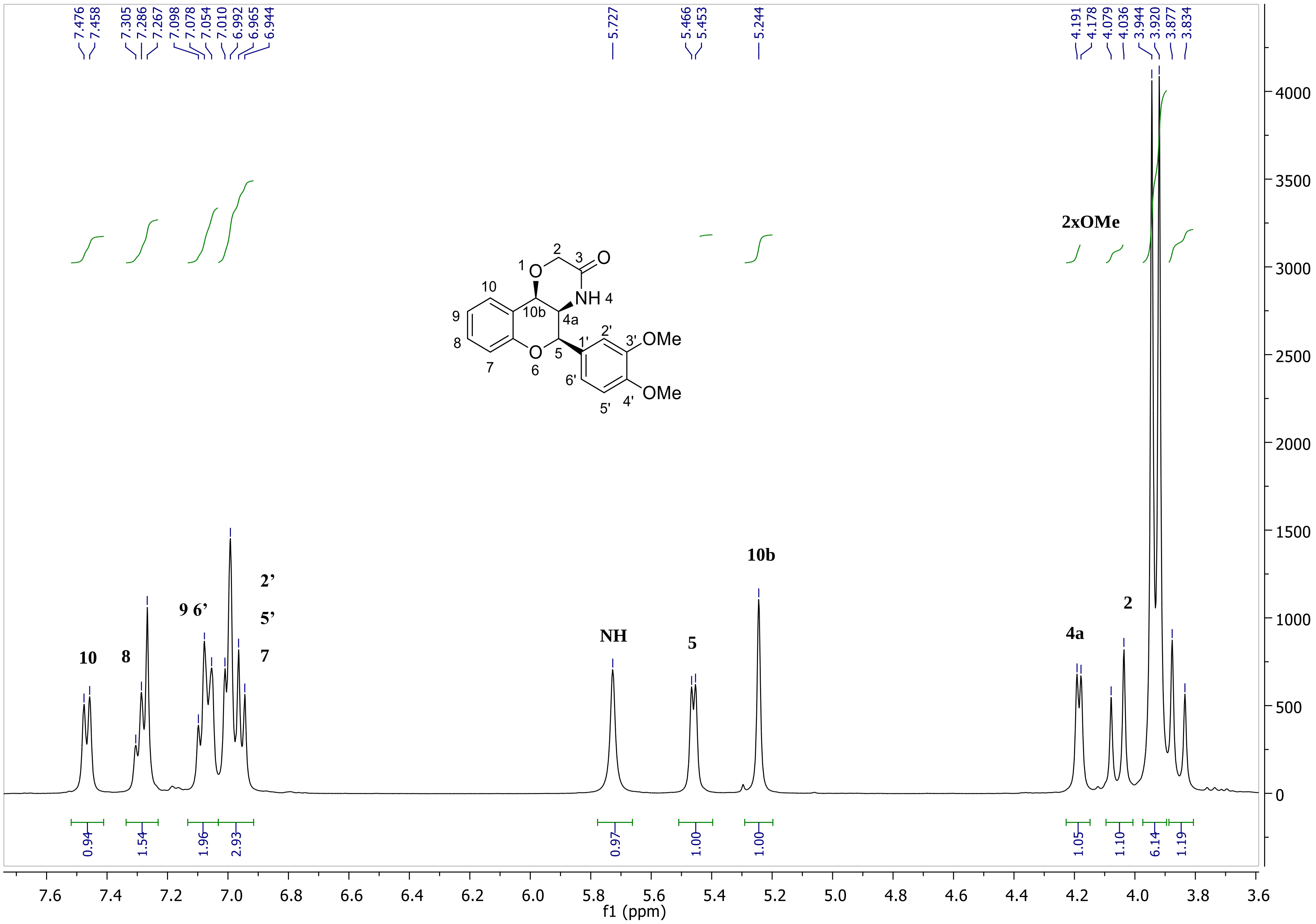

Figure S146. ^{1}H-NMR spectrum of *rac*-**23c** in $CDCl_3$

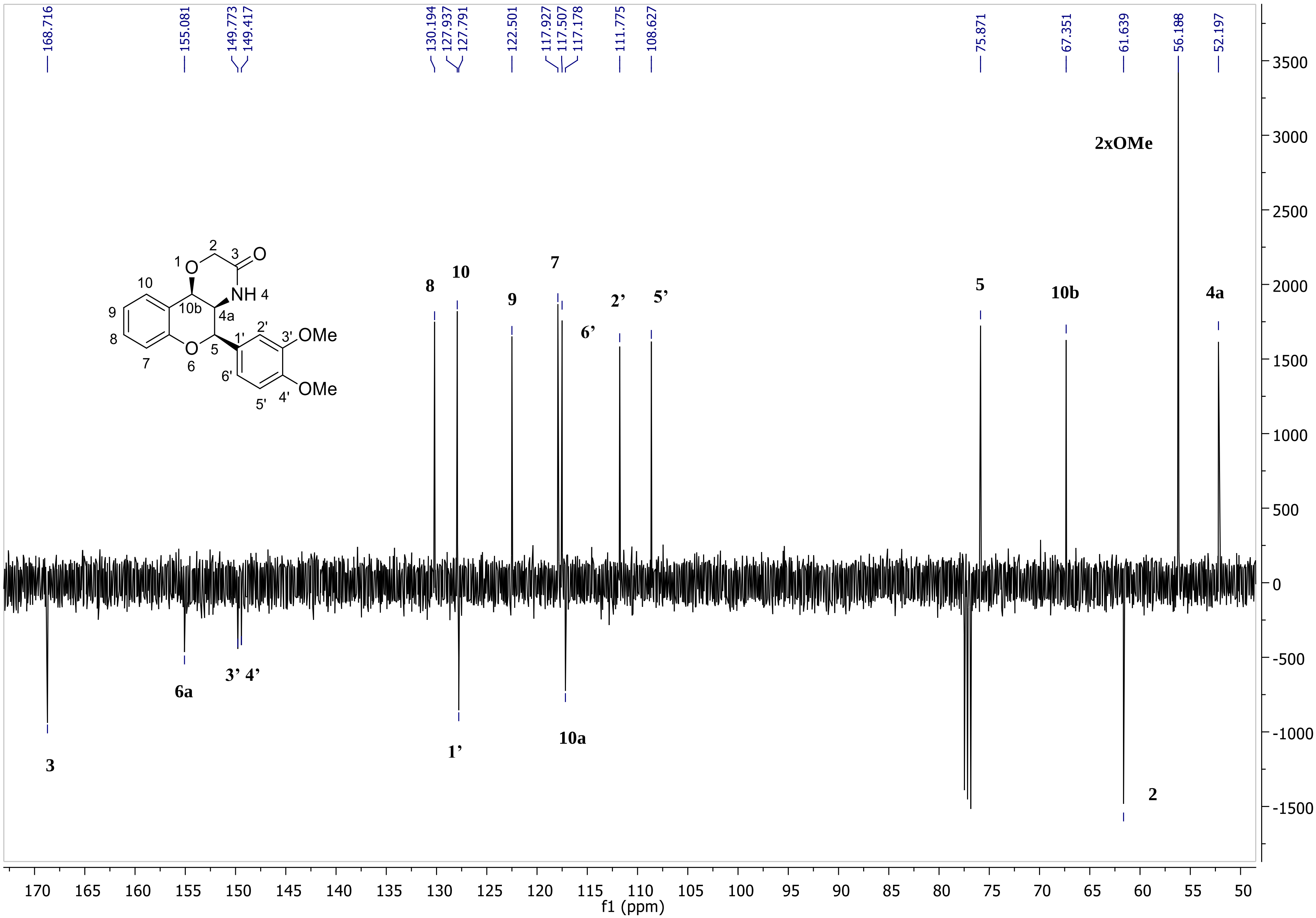

Figure S147. ^{13}C-NMR spectrum of *rac*-**23c** in $CDCl_3$

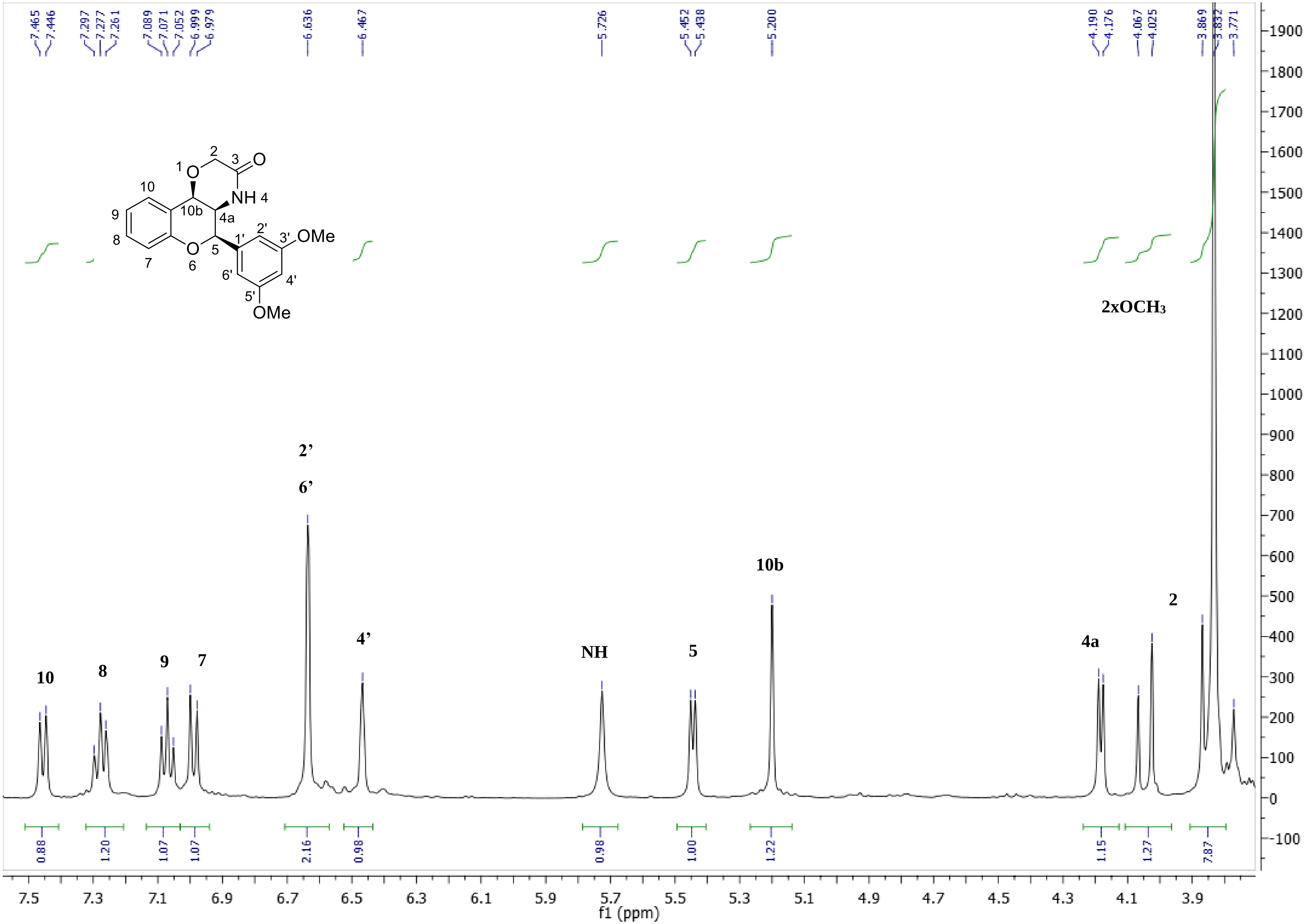

Figure S148. ^{1}H-NMR spectrum of *rac*-**23d** in $CDCl_3$

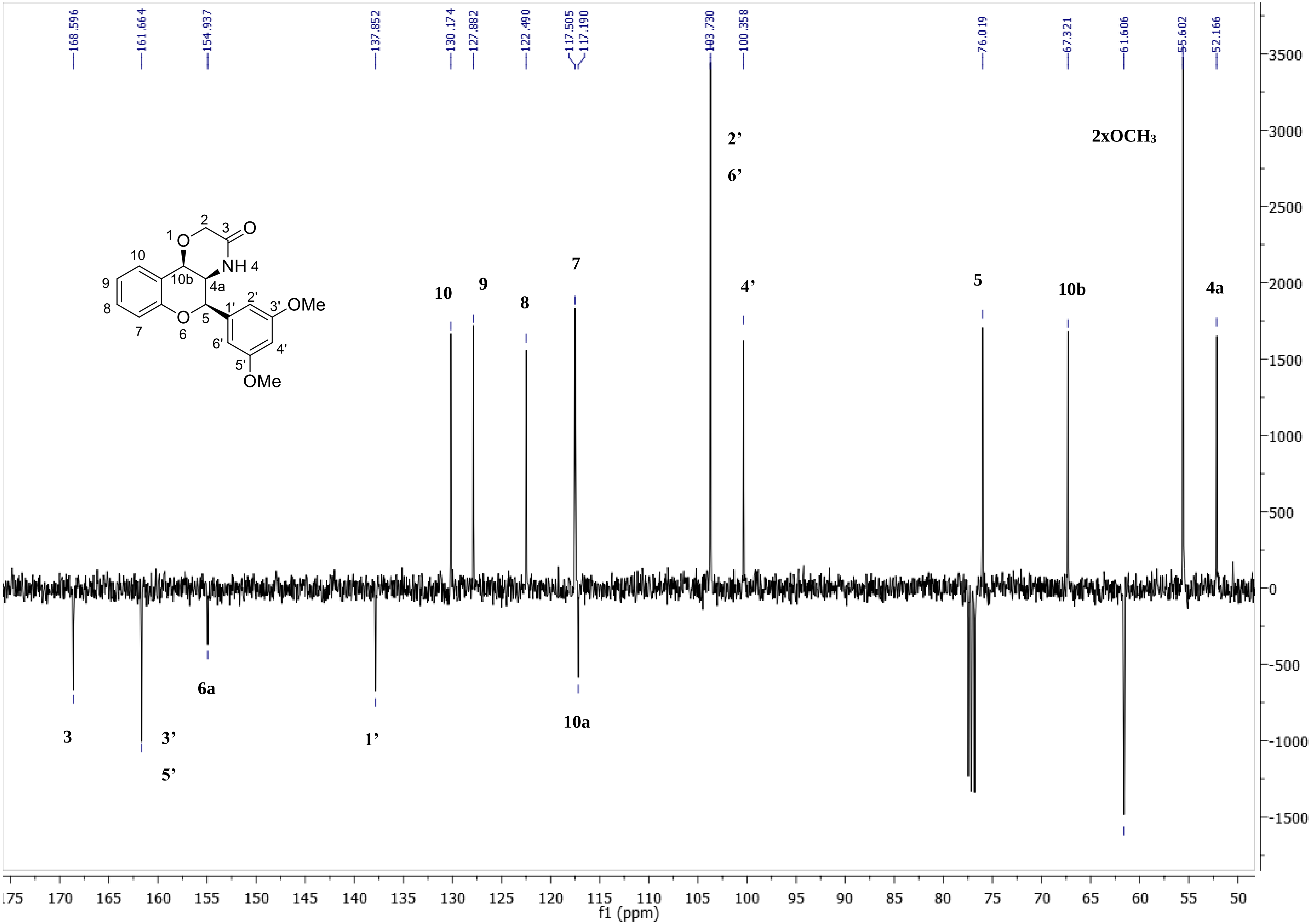

Figure S149. ^{13}C-NMR spectrum of *rac*-**23d** in $CDCl_3$

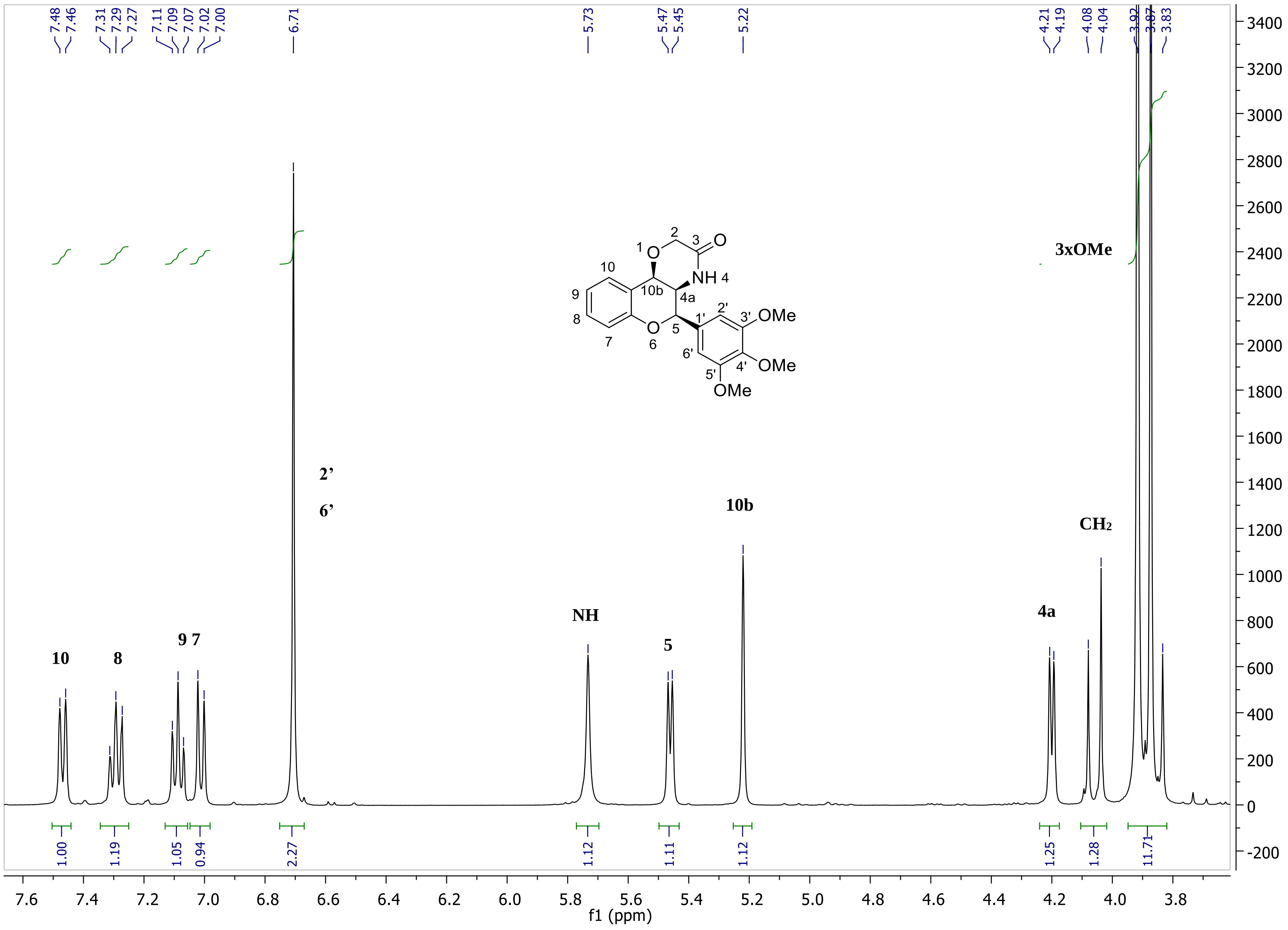

Figure S150. ^{1}H-NMR spectrum of *rac*-**23e** in $CDCl_3$

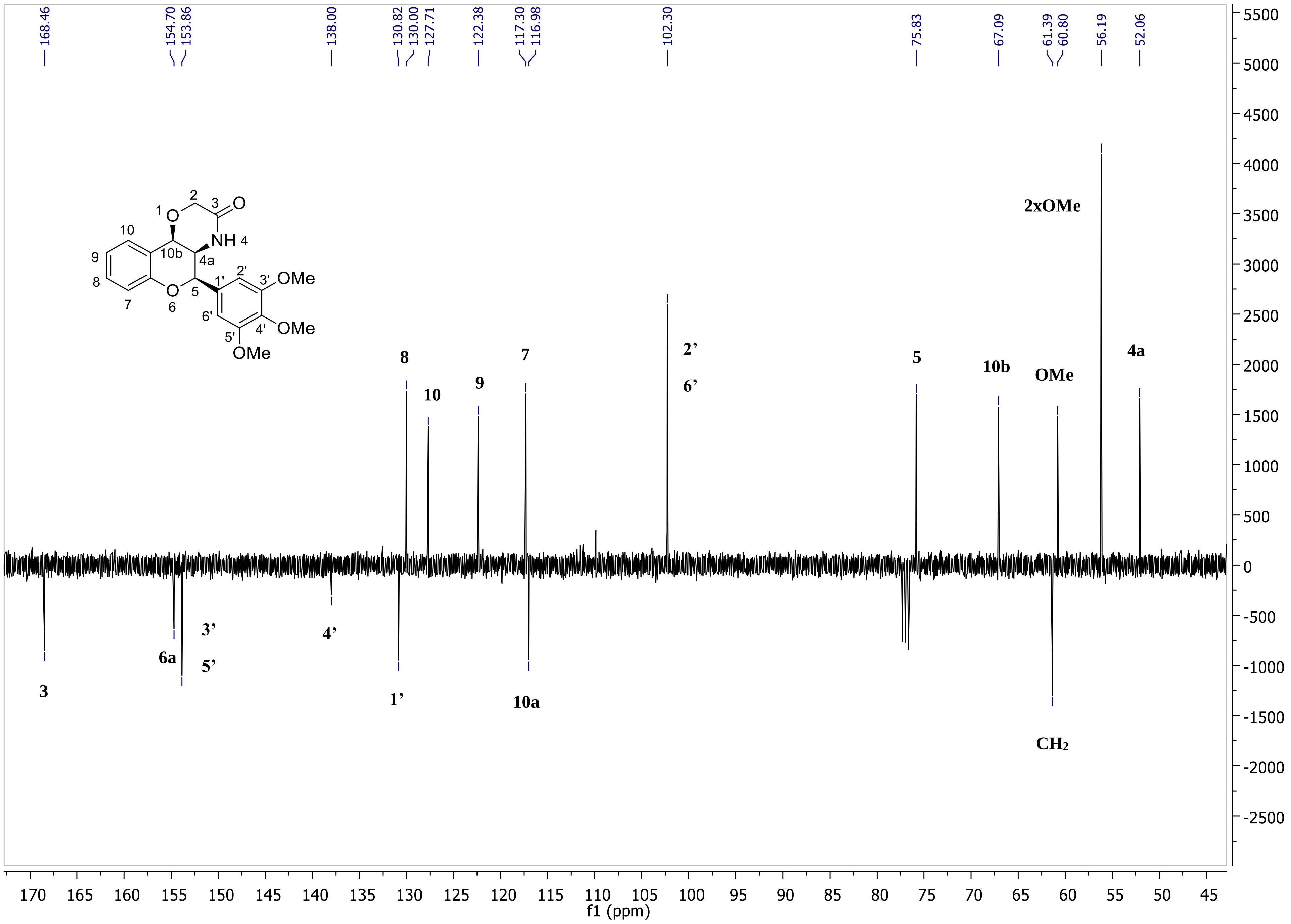

Figure S151. ^{13}C-NMR spectrum of *rac*-**23e** in $CDCl_3$

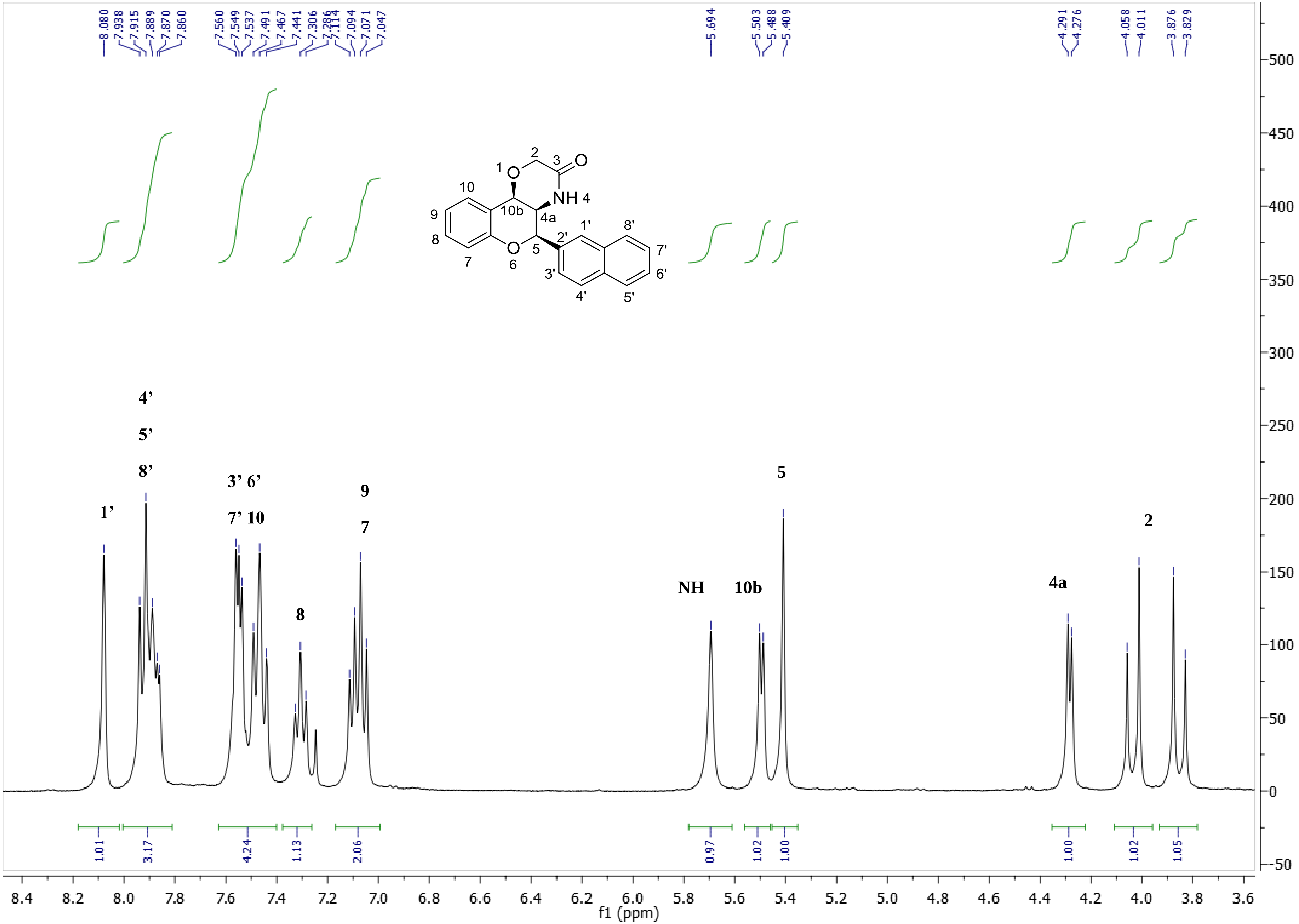

Figure S152. ^{1}H-NMR spectrum of *rac*-**23g** in $CDCl_3$

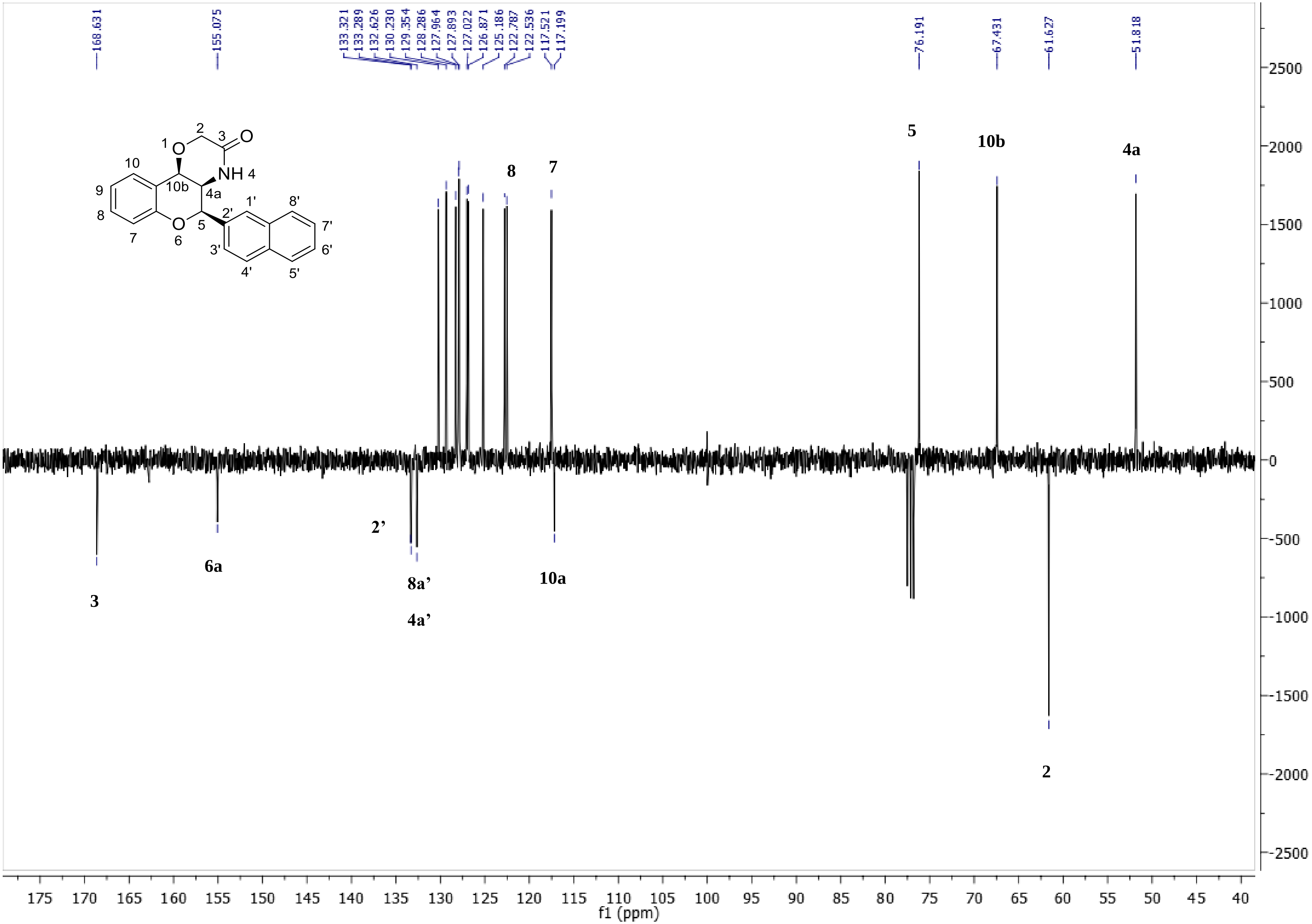

Figure S153. ^{13}C-NMR spectrum of *rac*-**23g** in $CDCl_3$

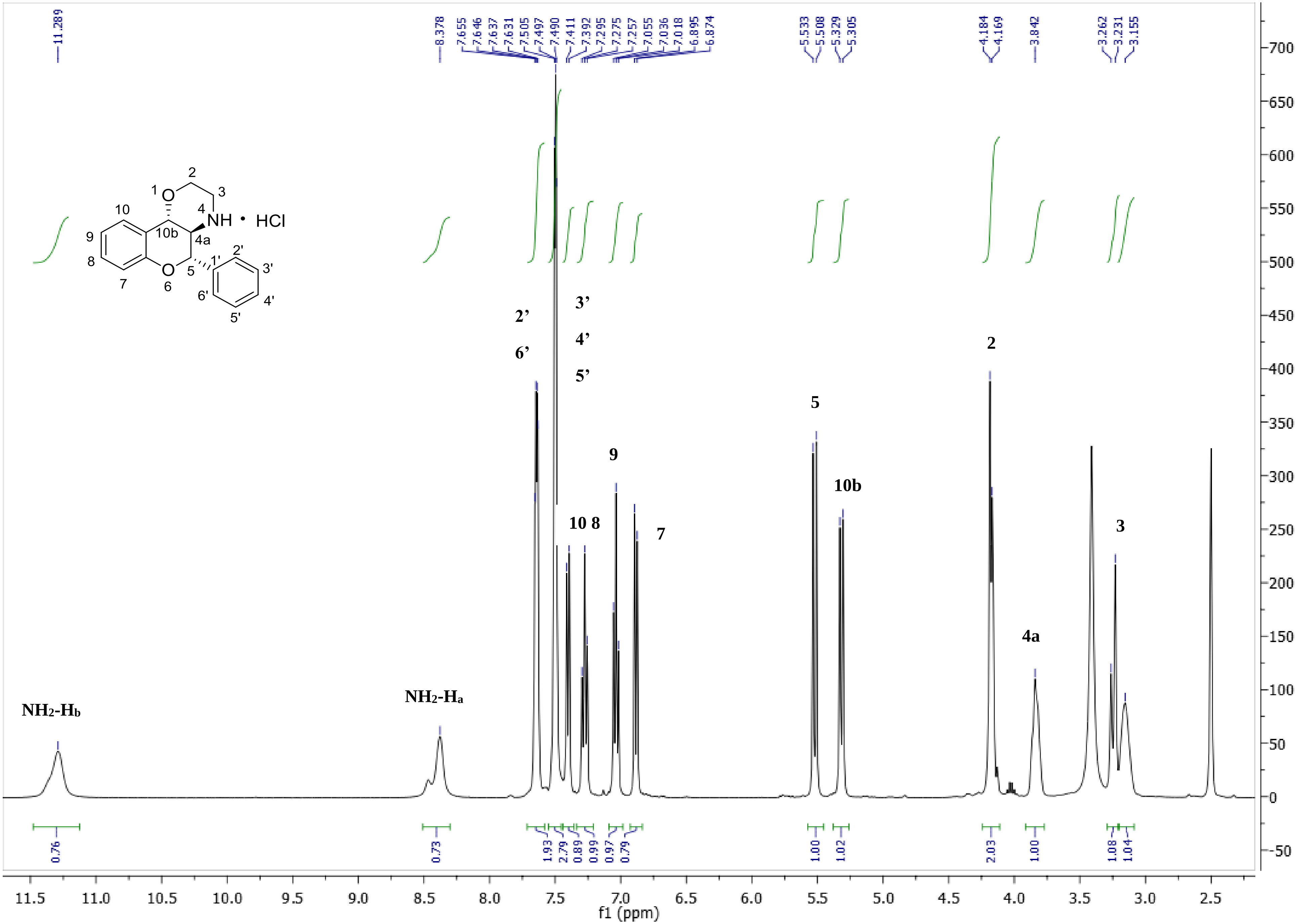

Figure S154. ^{1}H-NMR spectrum of *rac*-(4a*R**,5*S**,10a*S**)-**2a** in DMSO-d_6

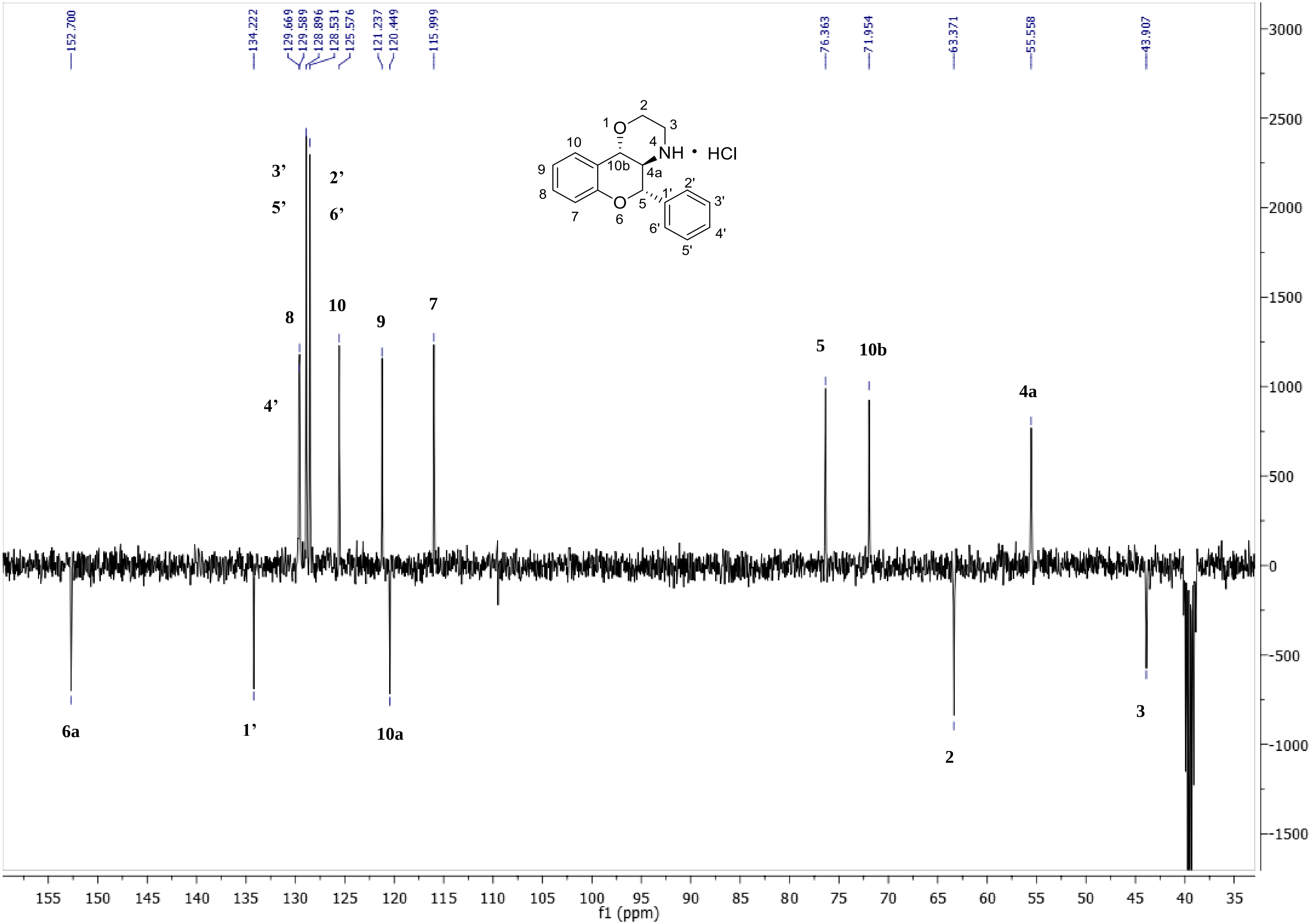

Figure S155. ^{13}C-NMR spectrum of *rac*-(4a*R**,5*S**,10a*S**)-**2a** in DMSO-d_6

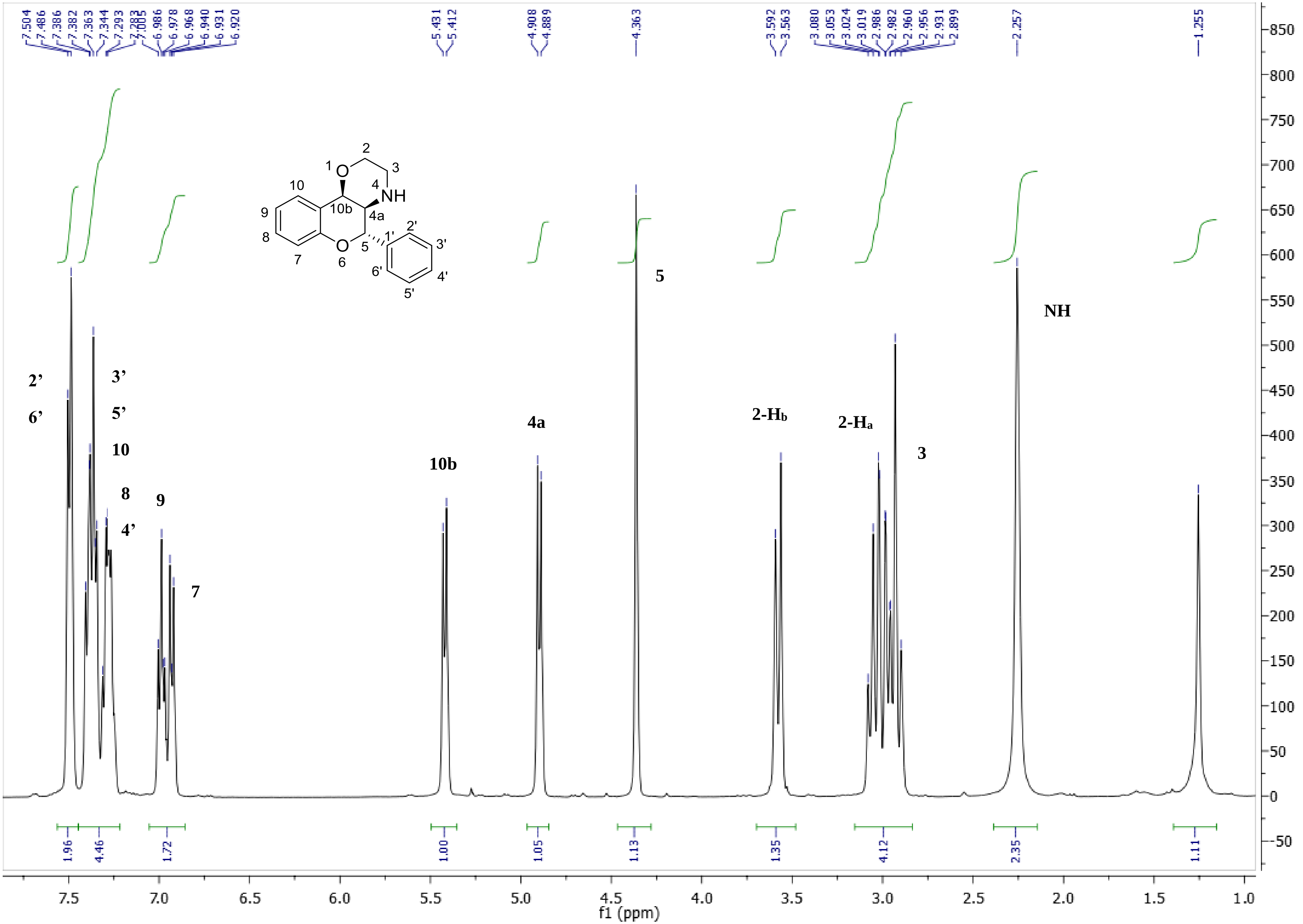

Figure S156. ^{1}H-NMR spectrum of *rac*-(4a*R**,5*S**,10a*R**)-**2a** in $CDCl_3$

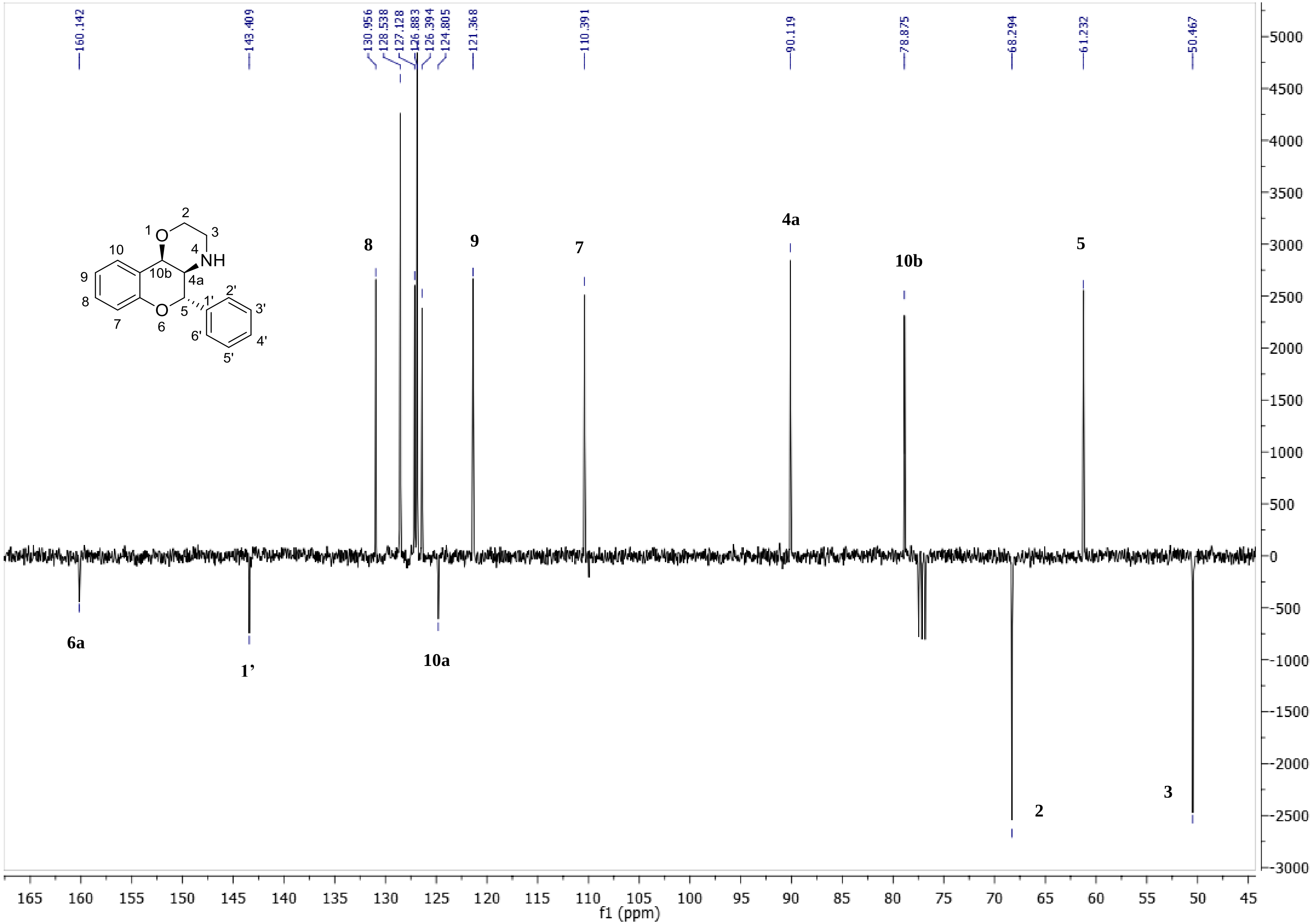

Figure S157. ^{13}C-NMR spectrum of *rac*-(4a*R**,5*S**,10a*R**)-**2a** in $CDCl_3$

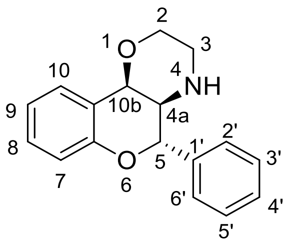

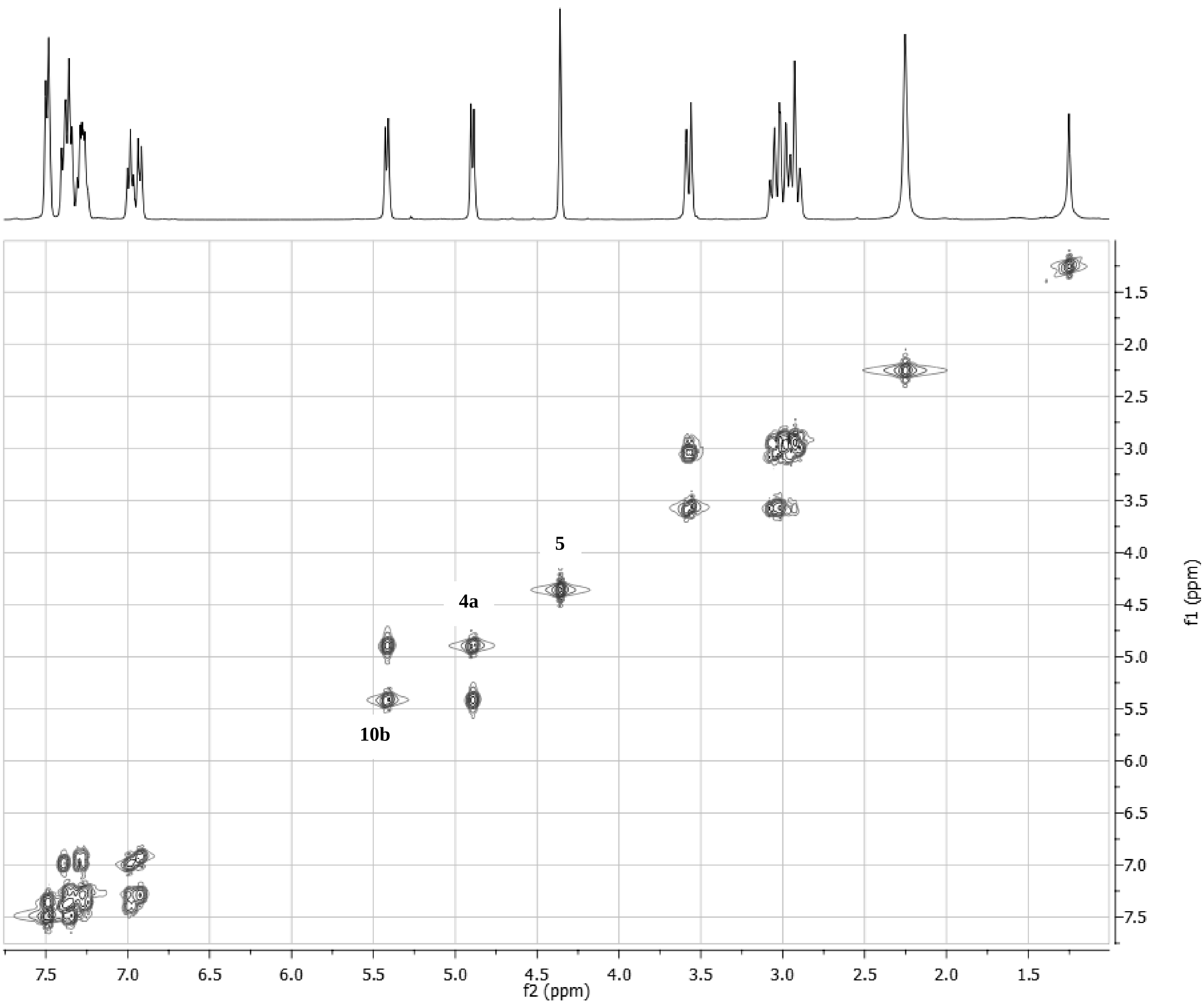

Figure S158. COSY spectrum of *rac*-(4a*R**,5*S**,10a*R**)-**2a** in $CDCl_3$

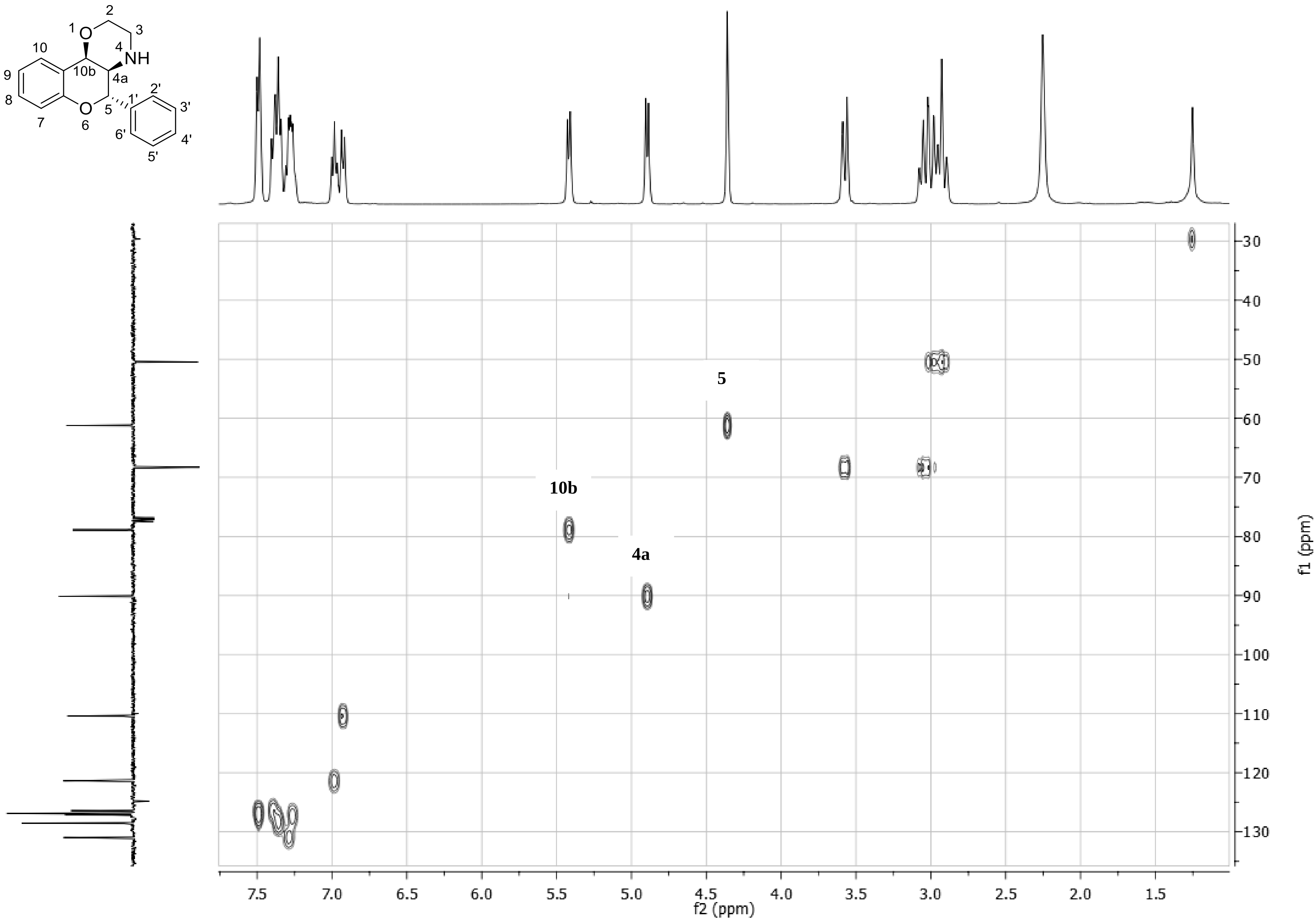

Figure S159. HSQC spectrum of *rac*-(4a*R**,5*S**,10a*R**)-**2a** in $CDCl_3$

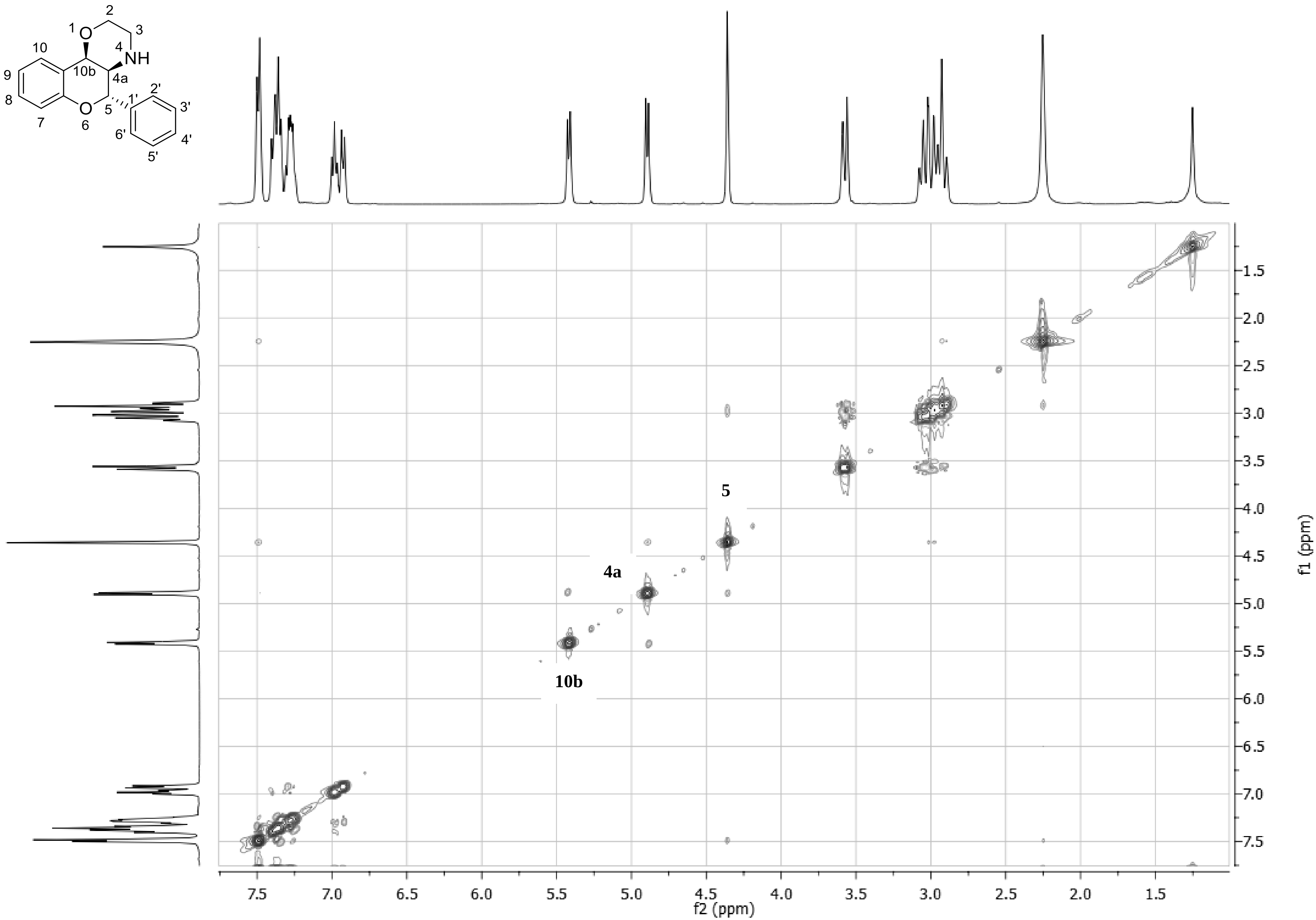

Figure S160. NOESY spectrum of *rac*-(4a*R**,5*S**,10a*R**)-**2a** in $CDCl_3$

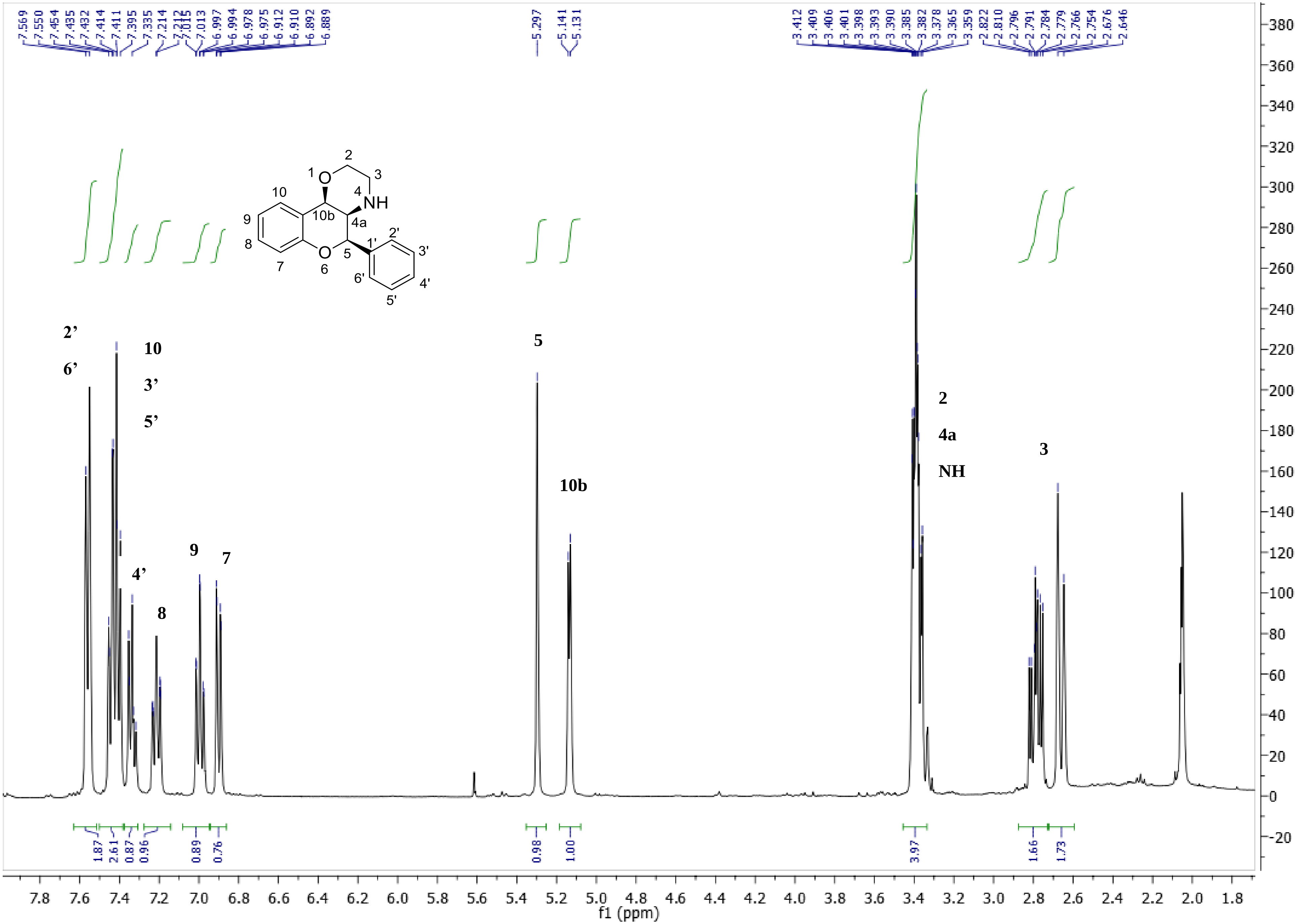

Figure S161. ^{1}H-NMR spectrum of *rac*-(4a*R**,5*R**,10a*R**)-**2a** in Acetone-d_6

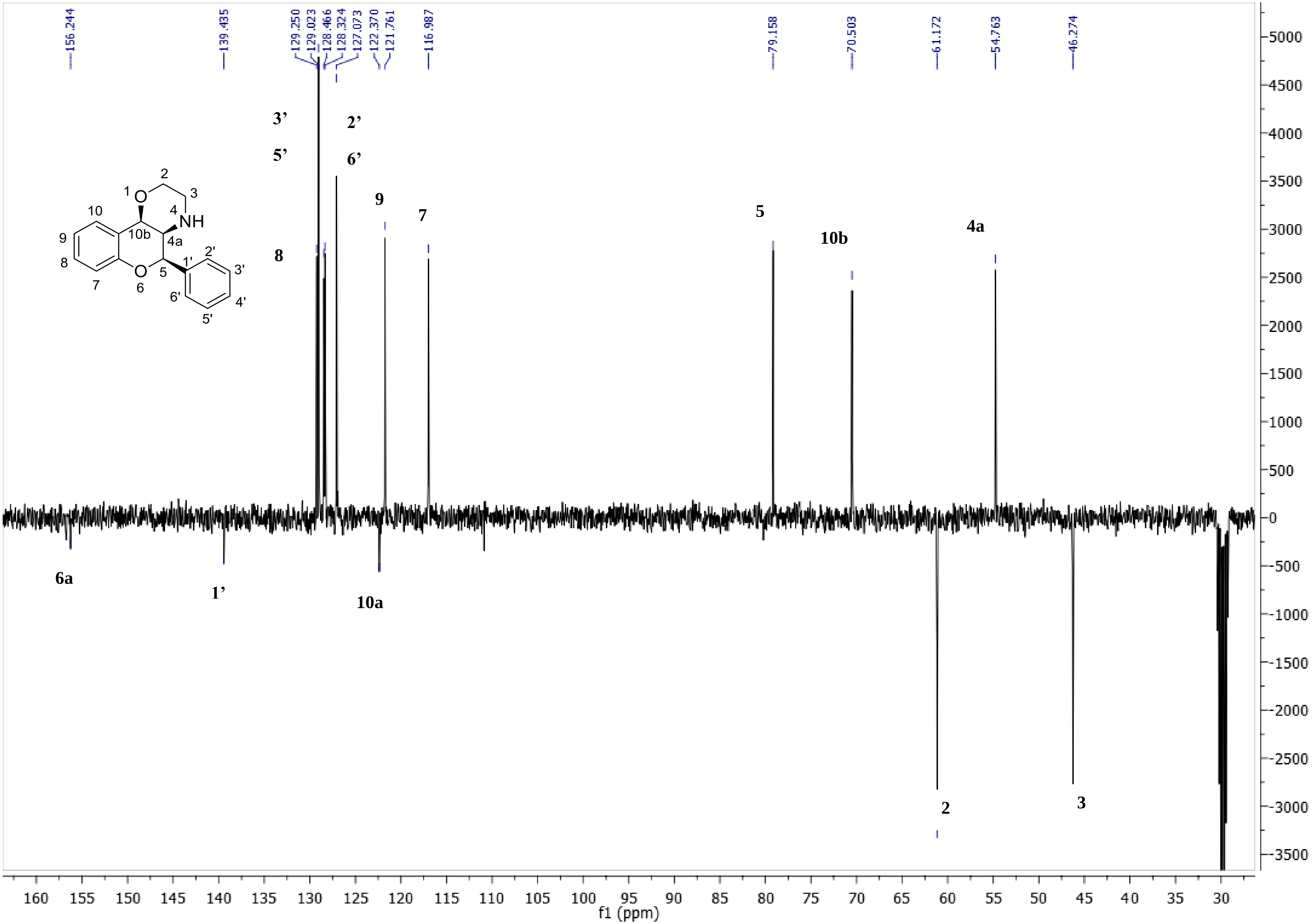

Figure S162. ^{13}C-NMR spectrum of *rac*-(4a*R**,5*R**,10a*R**)-**2a** in Acetone-d_6

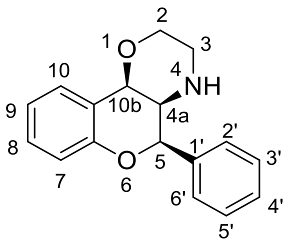

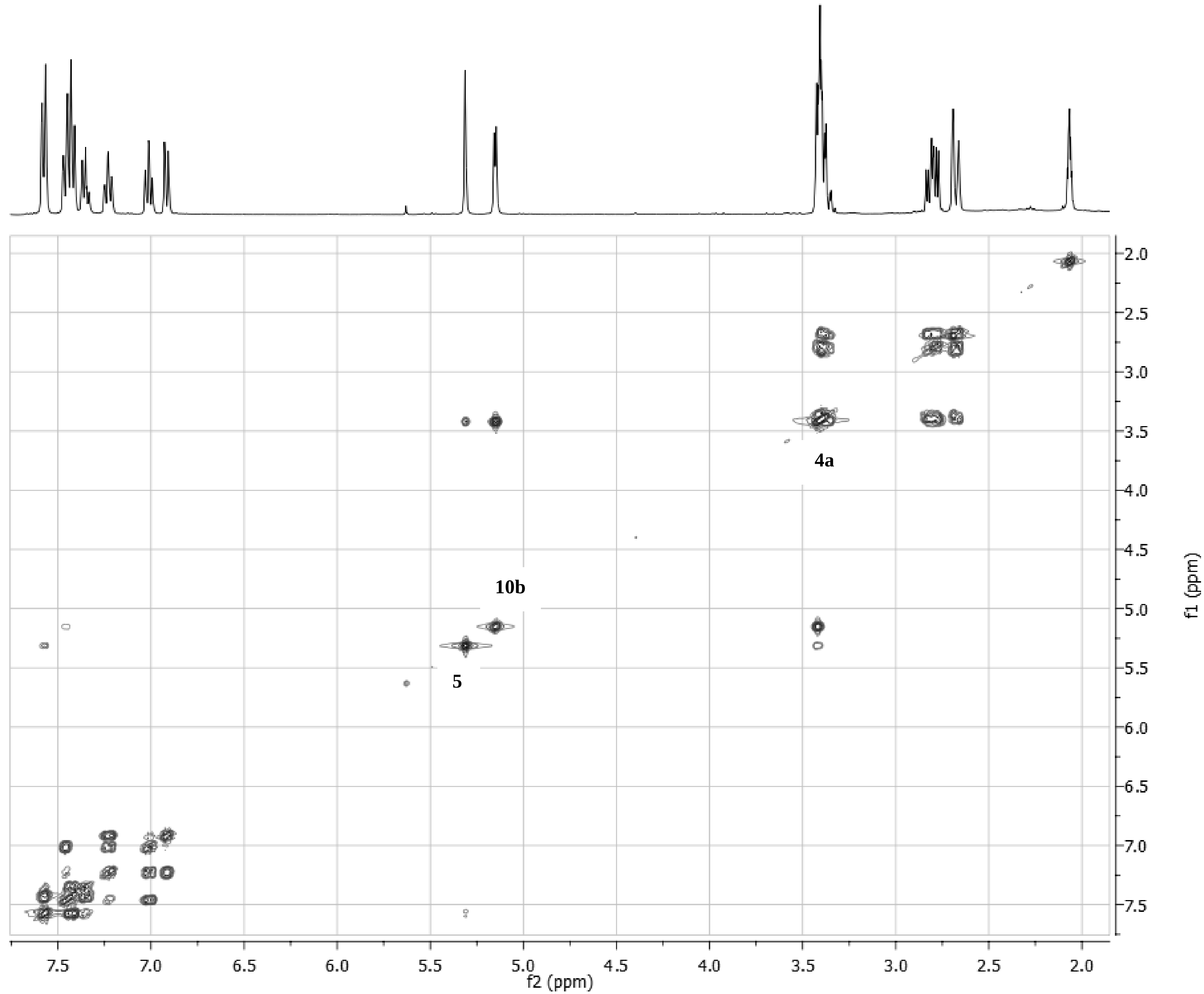

Figure S163. COSY spectrum of *rac*-(4a*R**,5*R**,10a*R**)-**2a** in Acetone-d_6

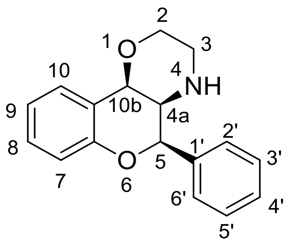

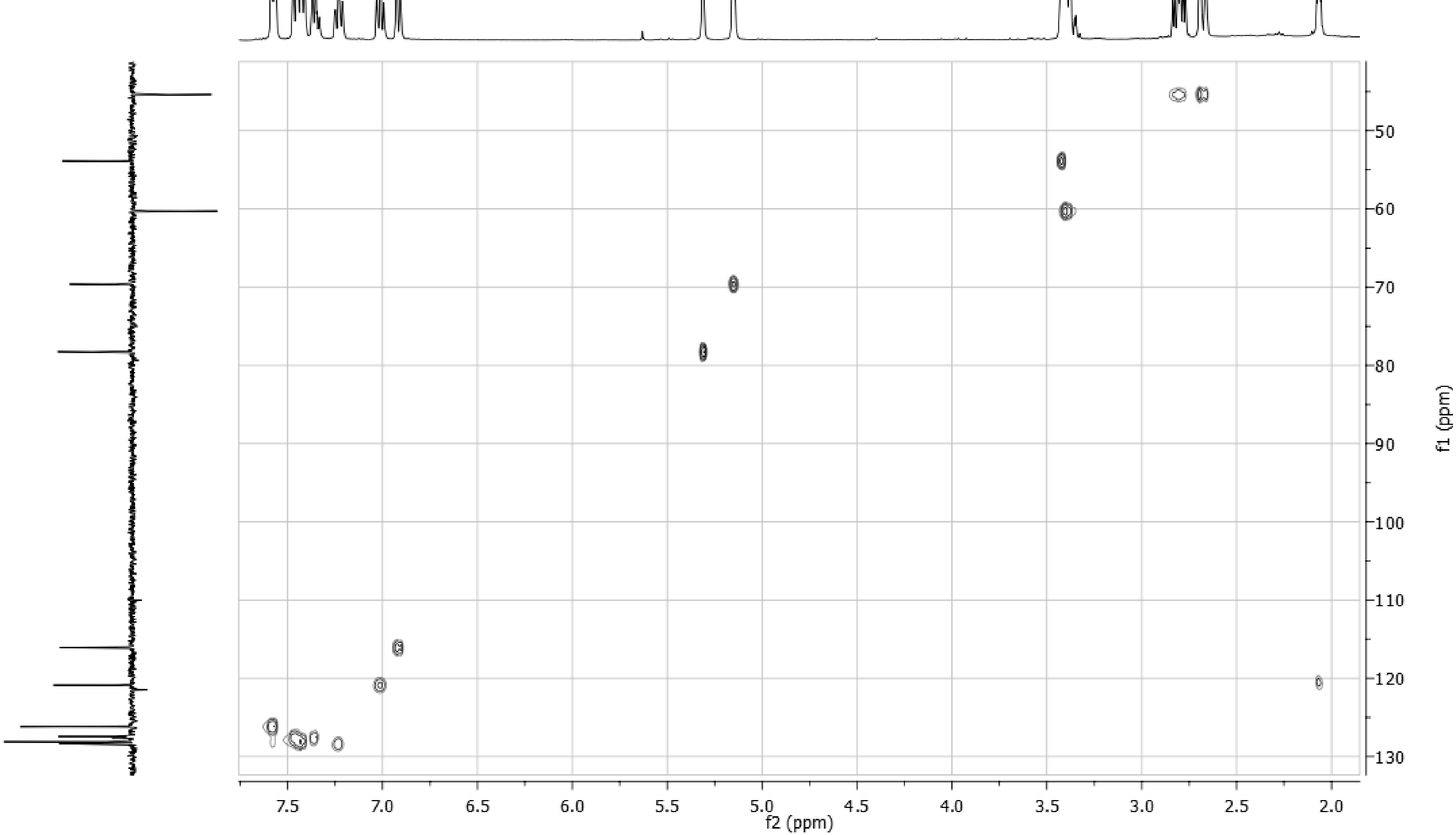

Figure S164. HSQC spectrum of *rac*-(4a*R**,5*R**,10a*R**)-**2a** in Acetone-d_6

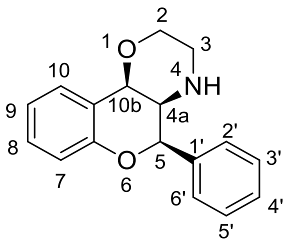

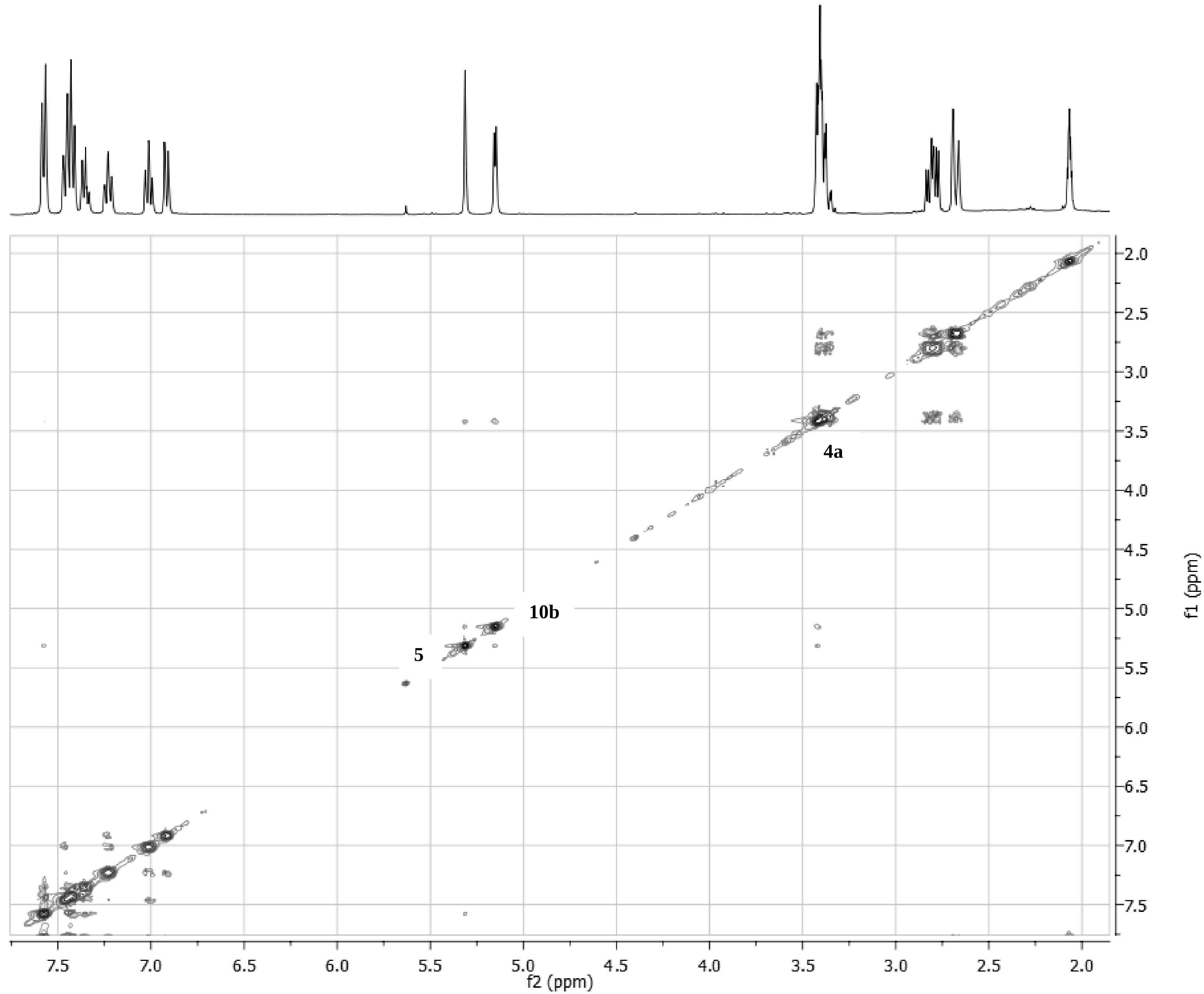

Figure 165. NOESY spectrum of *rac*-(4a*R**,5*R**,10a*R**)-**2a** in Acetone-d_6

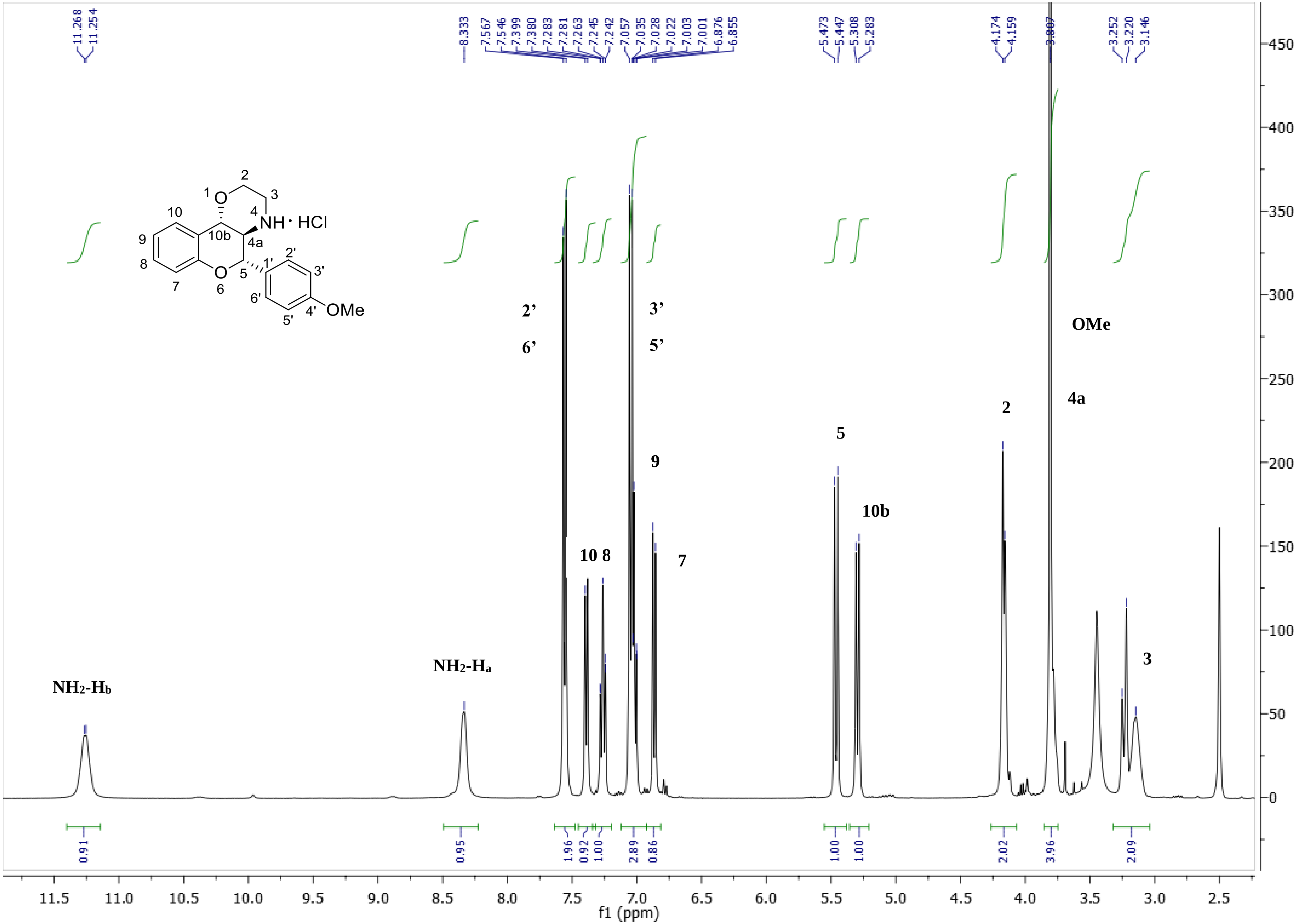

Figure S166. ^{1}H-NMR spectrum of *rac*-(4a*R**,5*S**,10a*S**)-**2b** in DMSO-d$_6$

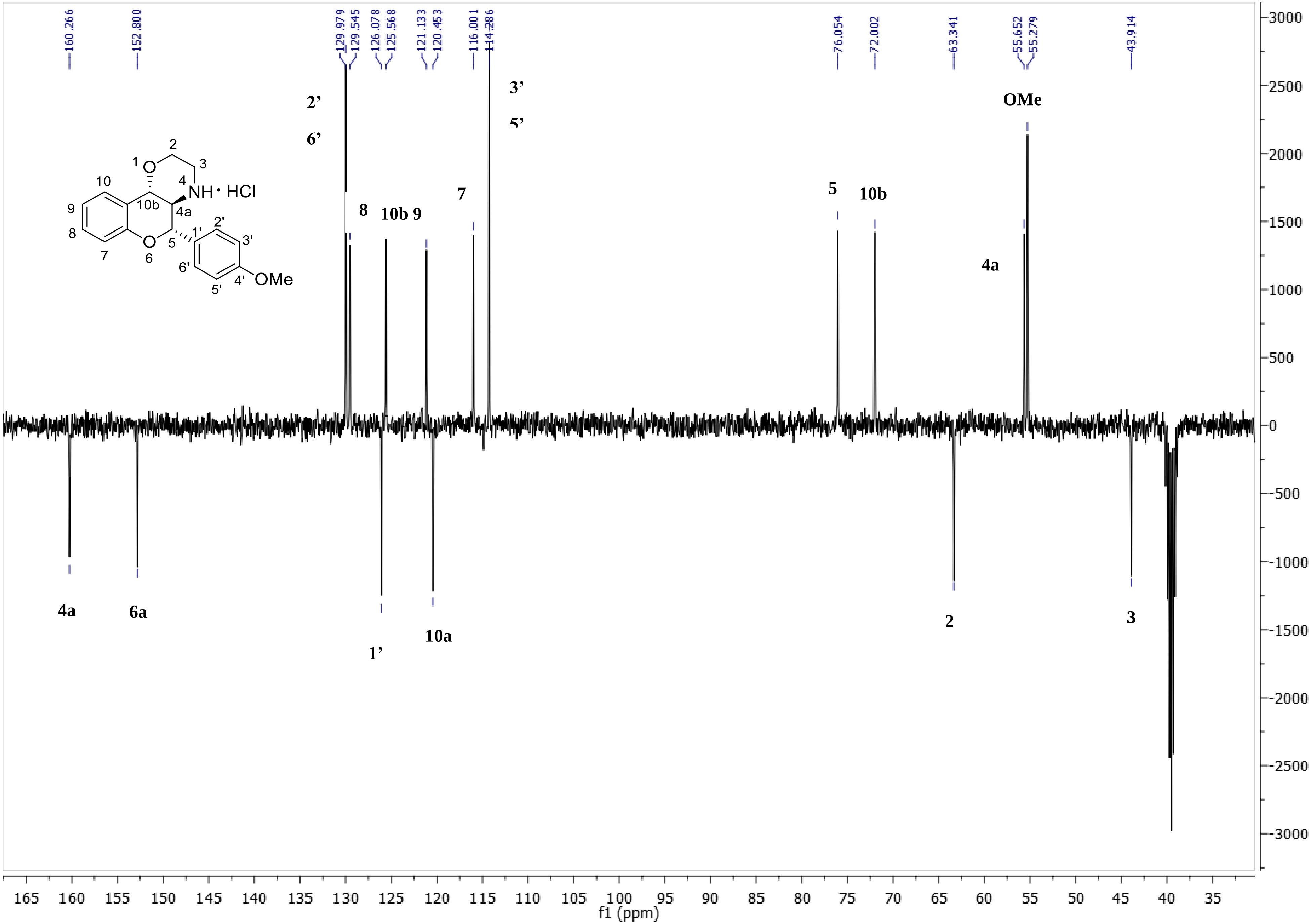

Figure S167. ^{13}C-NMR spectrum of *rac*-(4a*R**,5*S**,10a*S**)-**2b** in DMSO-d_6

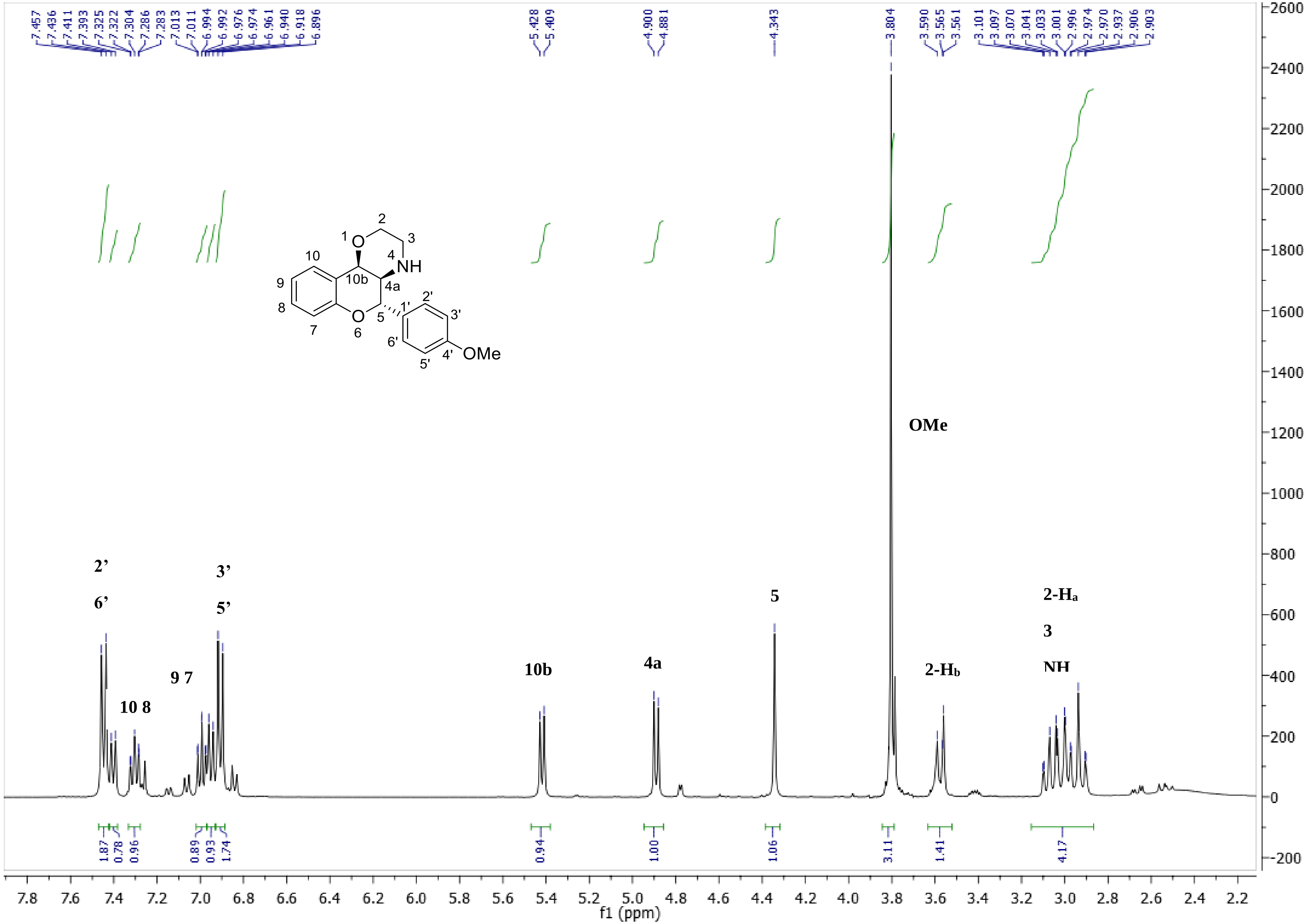

Figure S168. ^{1}H-NMR spectrum of *rac*-(4a*R**,5*S**,10a*R**)-**2b** in $CDCl_3$

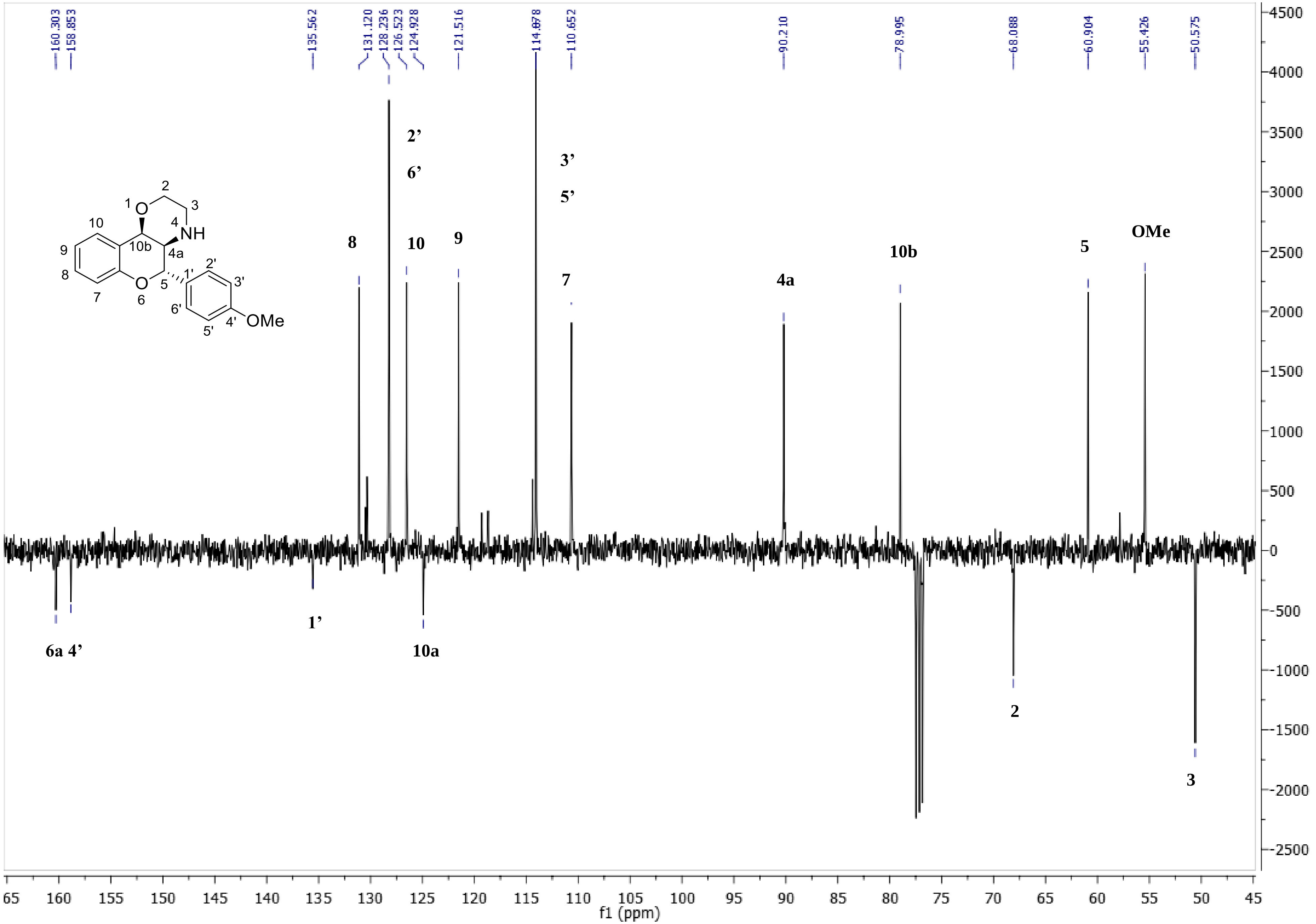

Figure S169. ^{13}C-NMR spectrum of *rac*-(4a*R**,5*S**,10a*R**)-**2b** in $CDCl_3$

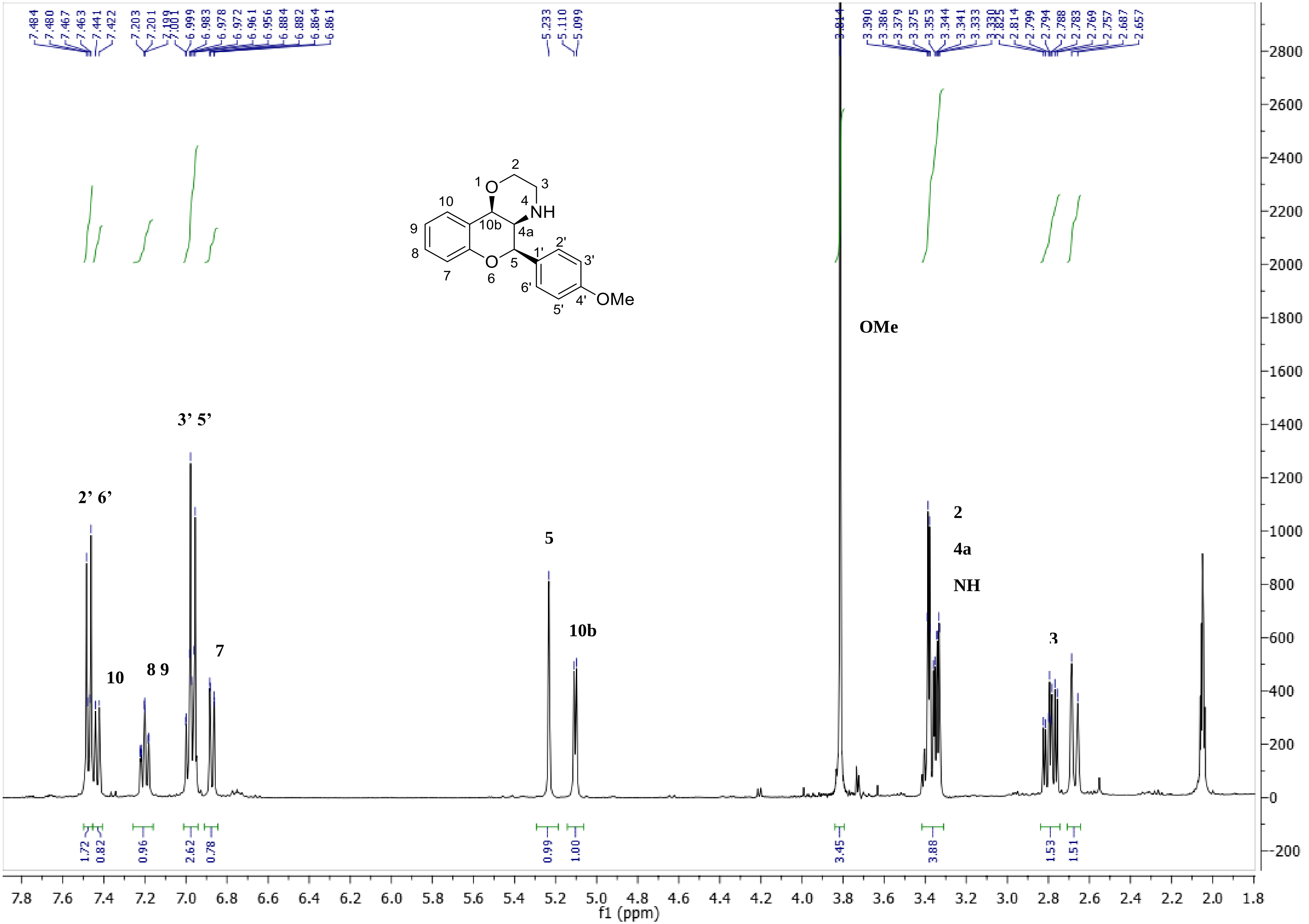

Figure S170. ^{1}H-NMR spectrum of *rac*-(4a*R**,5*R**,10a*R**)-**2b** in Acetone-d$_6$

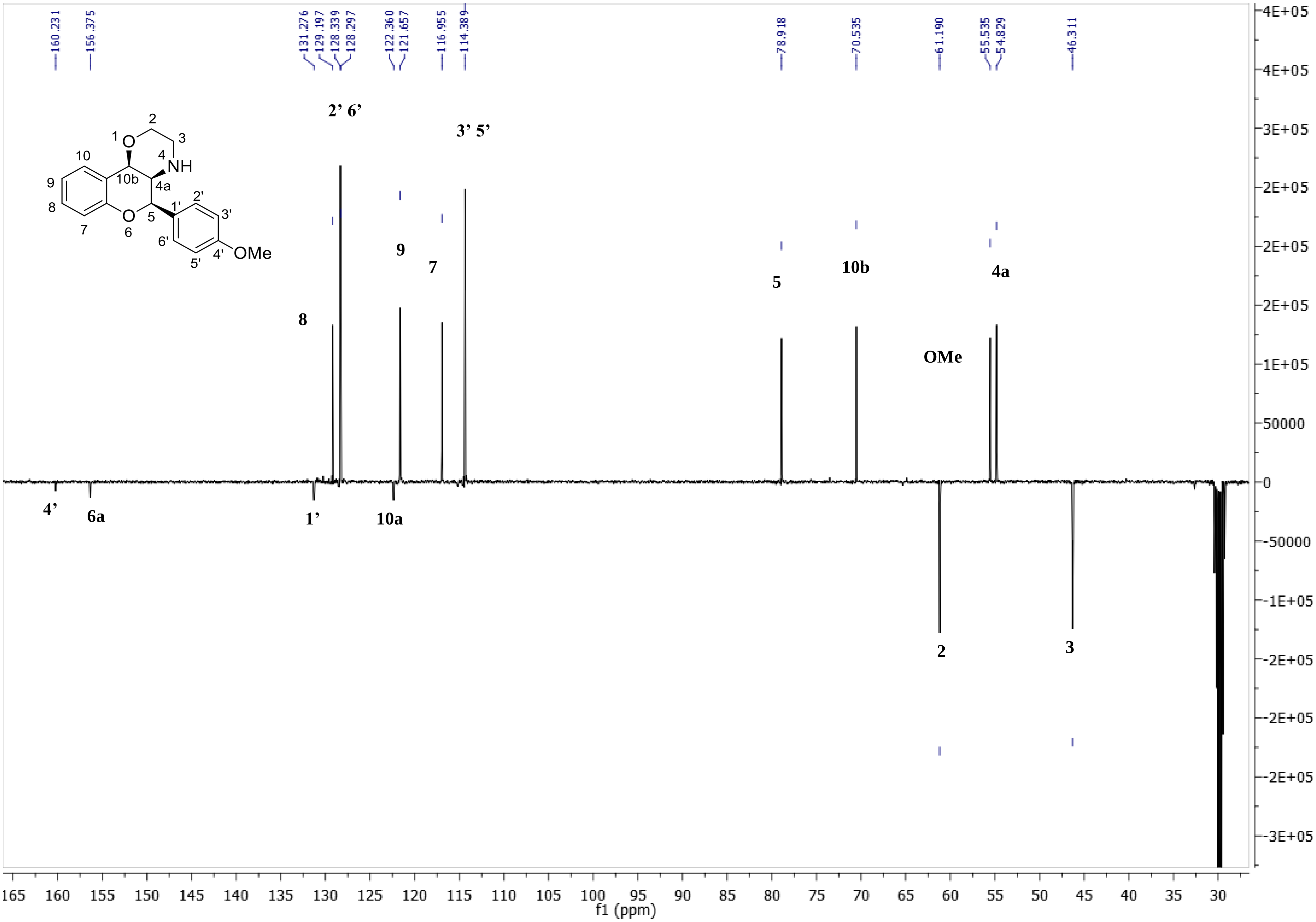

Figure S171. ^{13}C-NMR spectrum of *rac*-(4a*R**,5*R**,10a*R**)-**2b** in Acetone-d$_6$

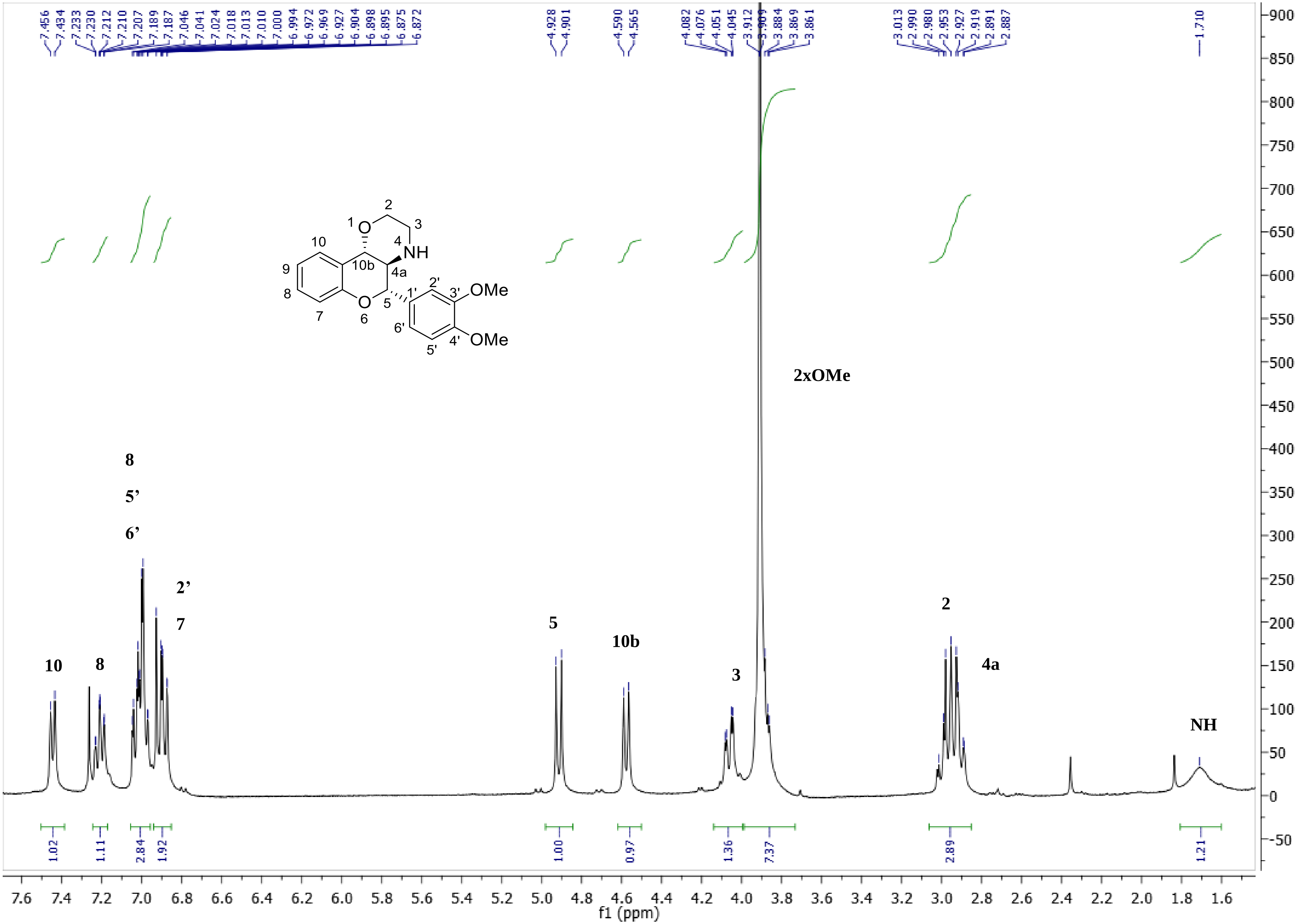

Figure S172. ^{1}H-NMR spectrum of *rac*-(4a*R**,5*S**,10a*S**)-**2c** in $CDCl_3$

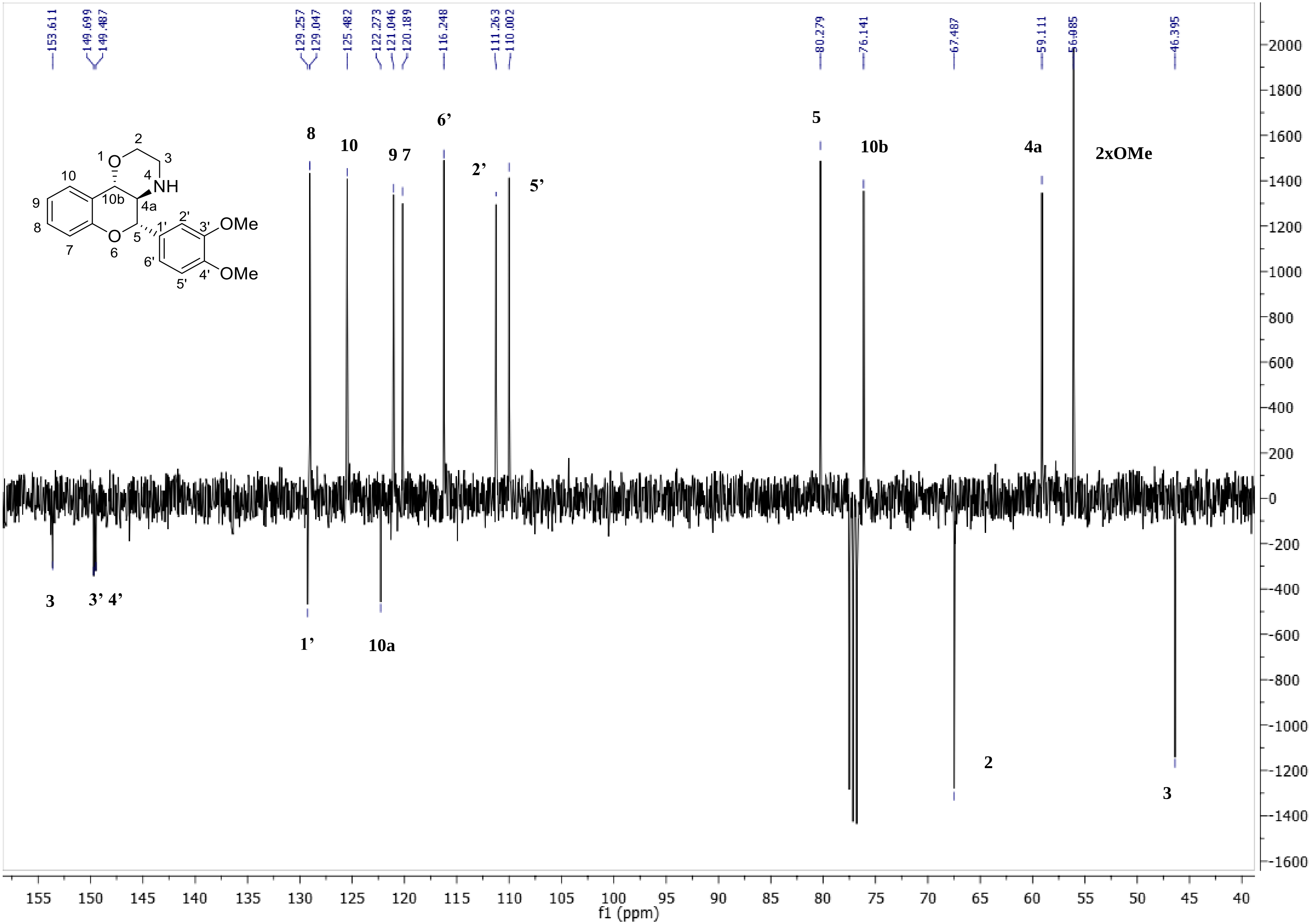

Figure S173. ^{13}C-NMR spectrum of *rac*-(4a*R**,5*S**,10a*S**)-**2c** in $CDCl_3$

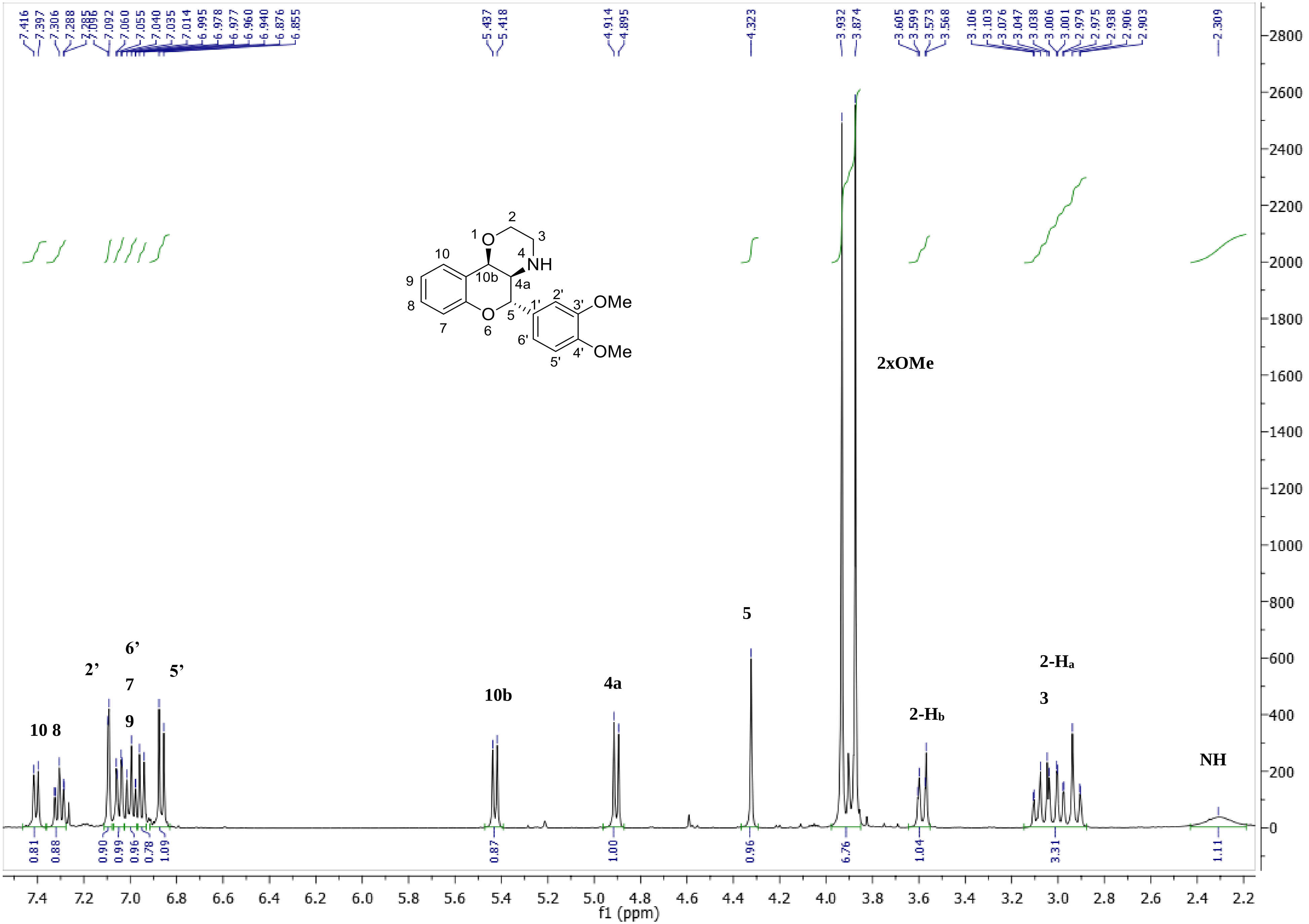

Figure S174. ^{1}H-NMR spectrum of *rac*-(4a*R**,5*S**,10a*R**)-**2c** in $CDCl_3$

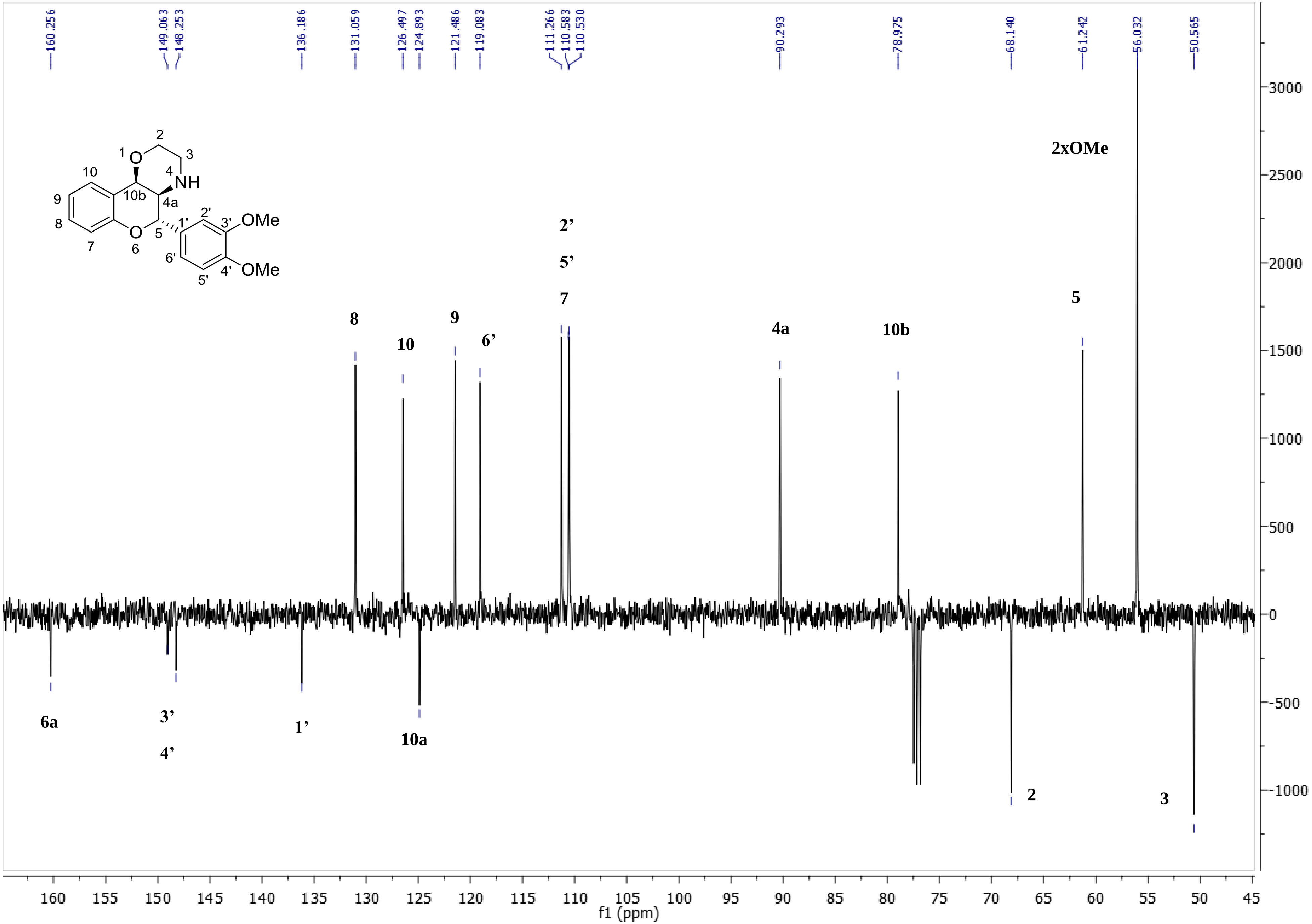

Figure S175. ^{13}C-NMR spectrum of *rac*-(4a*R**,5*S**,10a*R**)-**2c** in $CDCl_3$

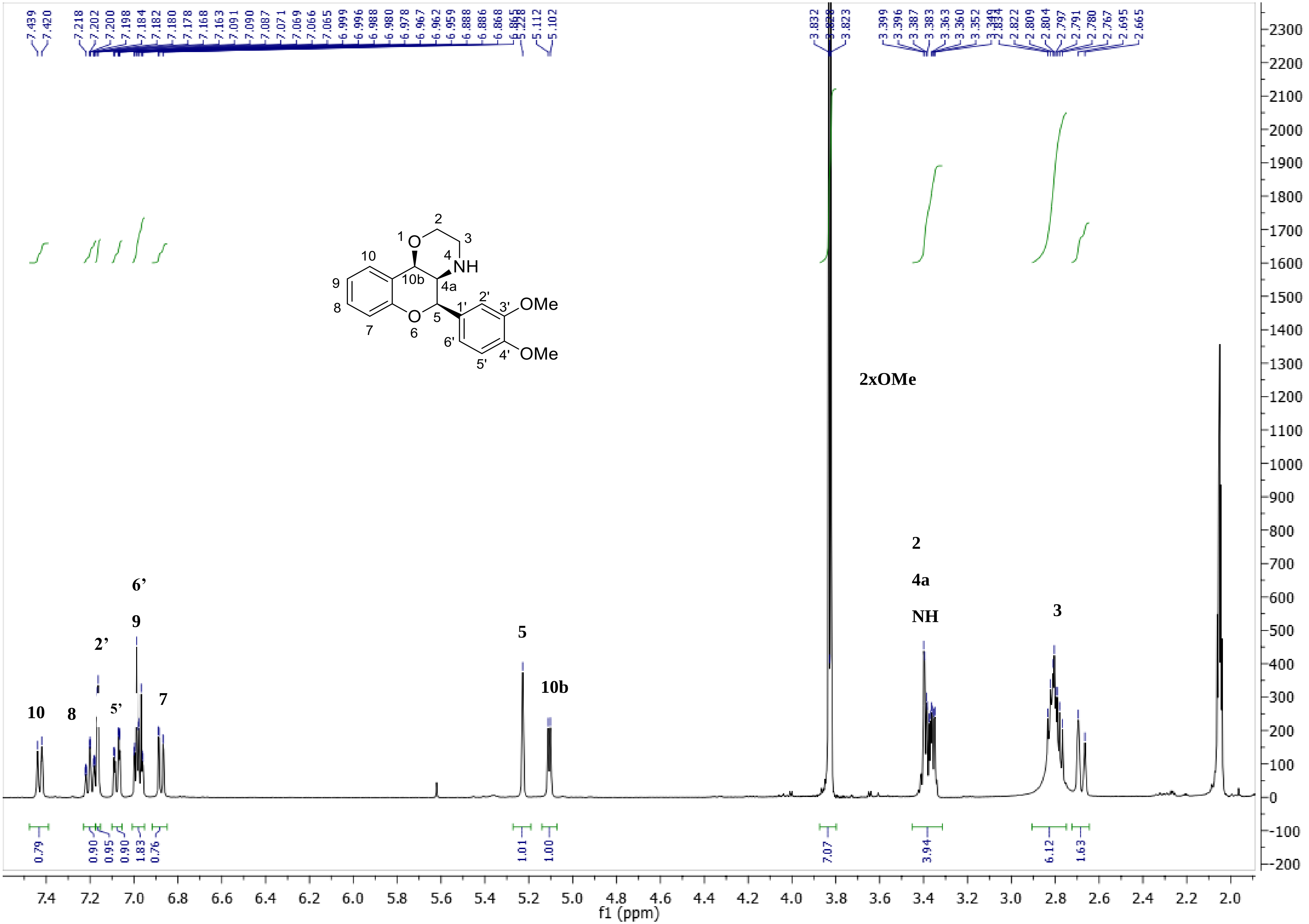

Figure S176. ^{1}H-NMR spectrum of *rac*-(4a*R**,5*R**,10a*R**)-**2c** in Acetone-d_6

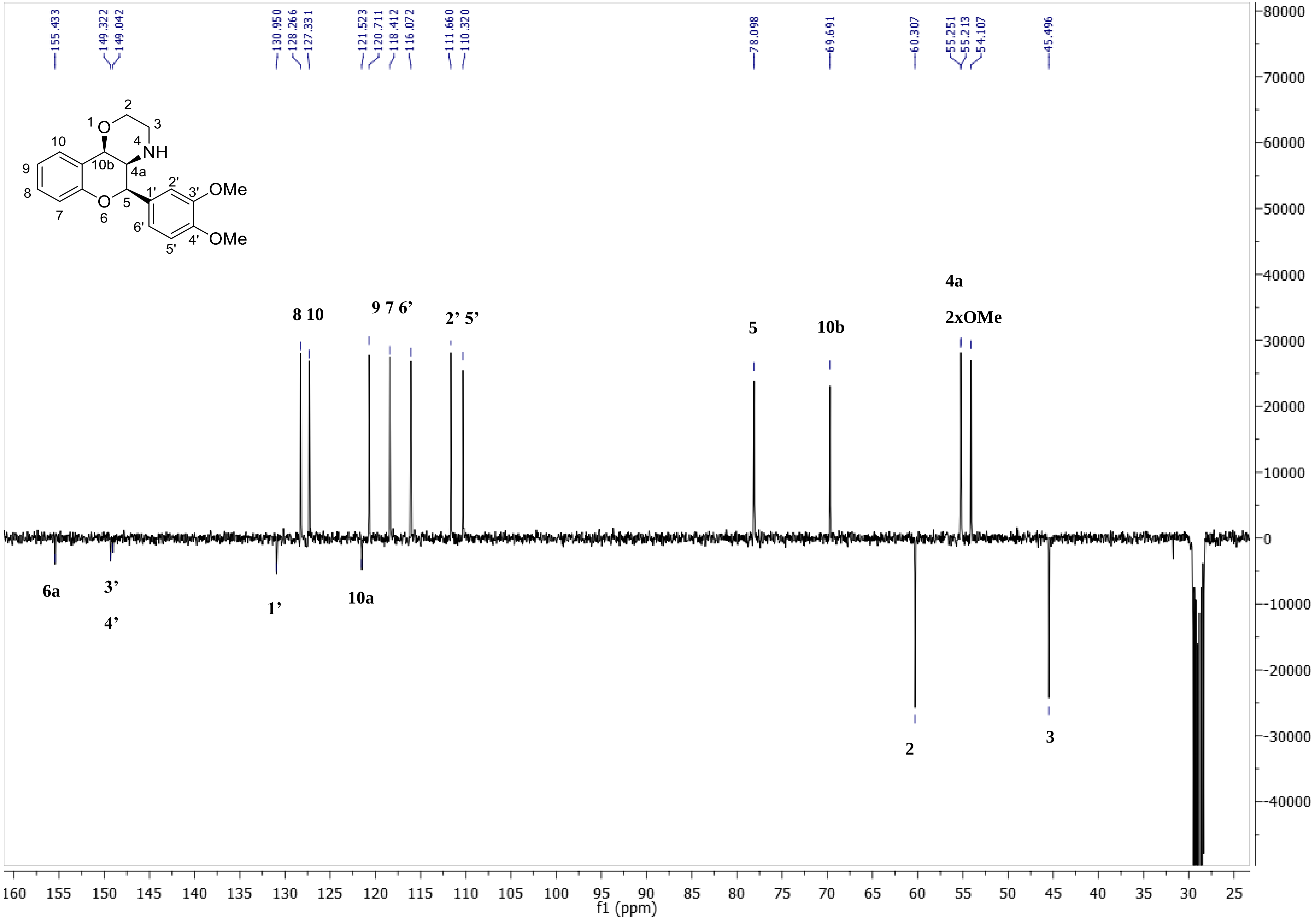

Figure S177. ^{13}C-NMR spectrum of *rac*-(4a*R**,5*R**,10a*R**)-**2c** in Acetone-d_6

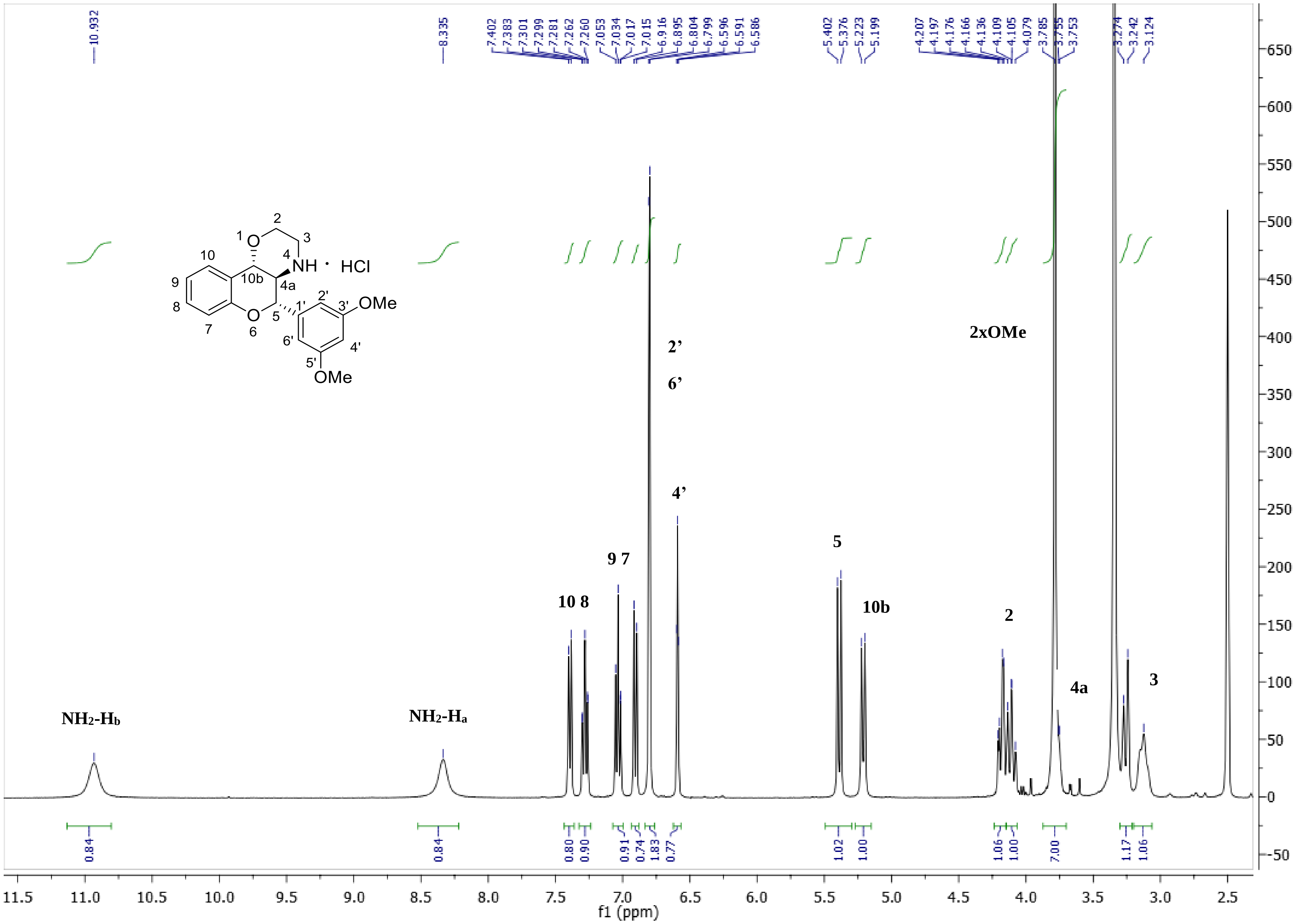

Figure S178. ^{1}H-NMR spectrum of *rac*-(4a*R**,5*S**,10a*S**)-**2d** in DMSO-d_6

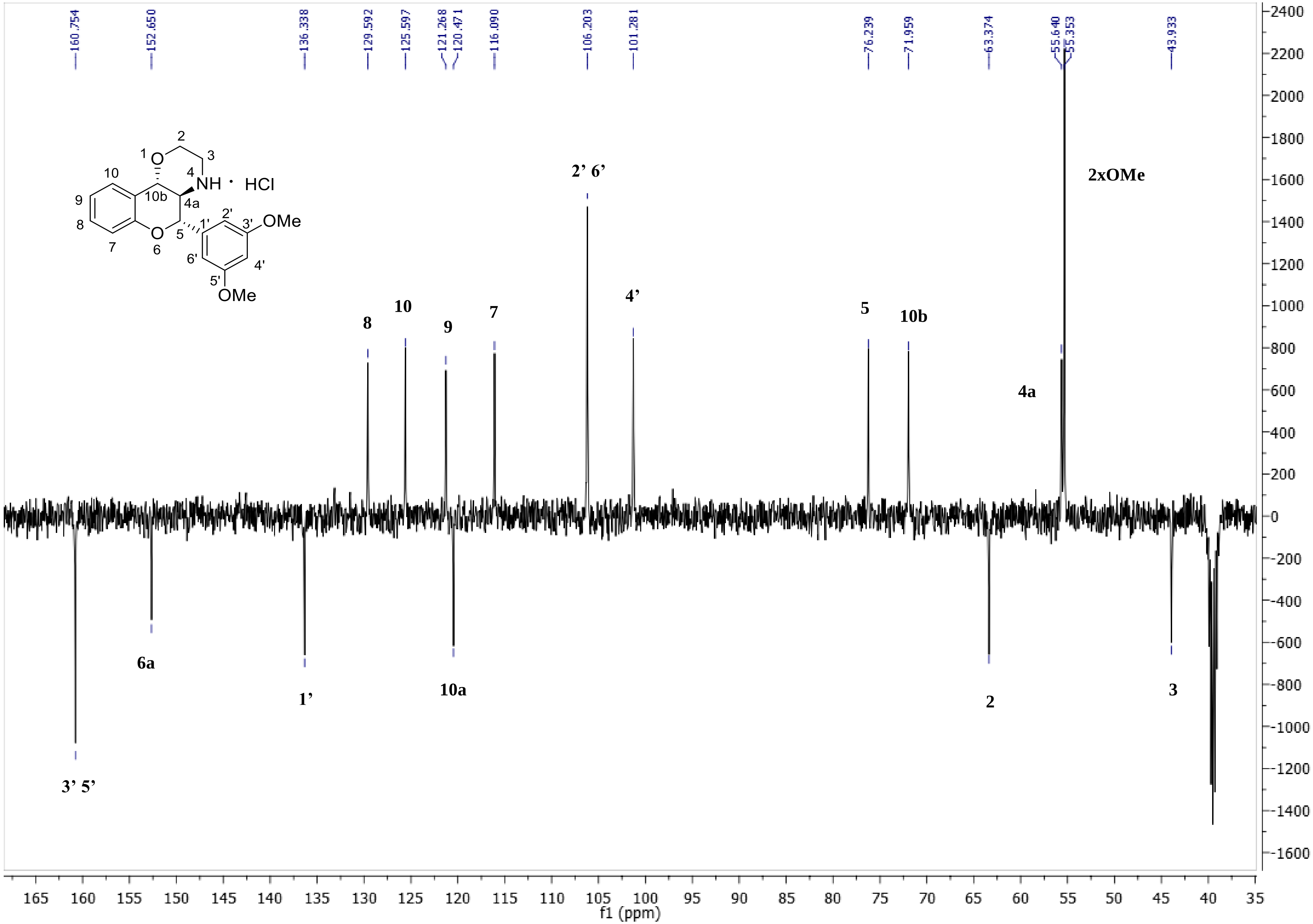

Figure S179. ^{13}C-NMR spectrum of *rac*-(4a*R**,5*S**,10a*S**)-**2d** in DMSO-d$_6$

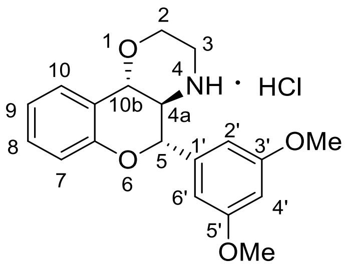

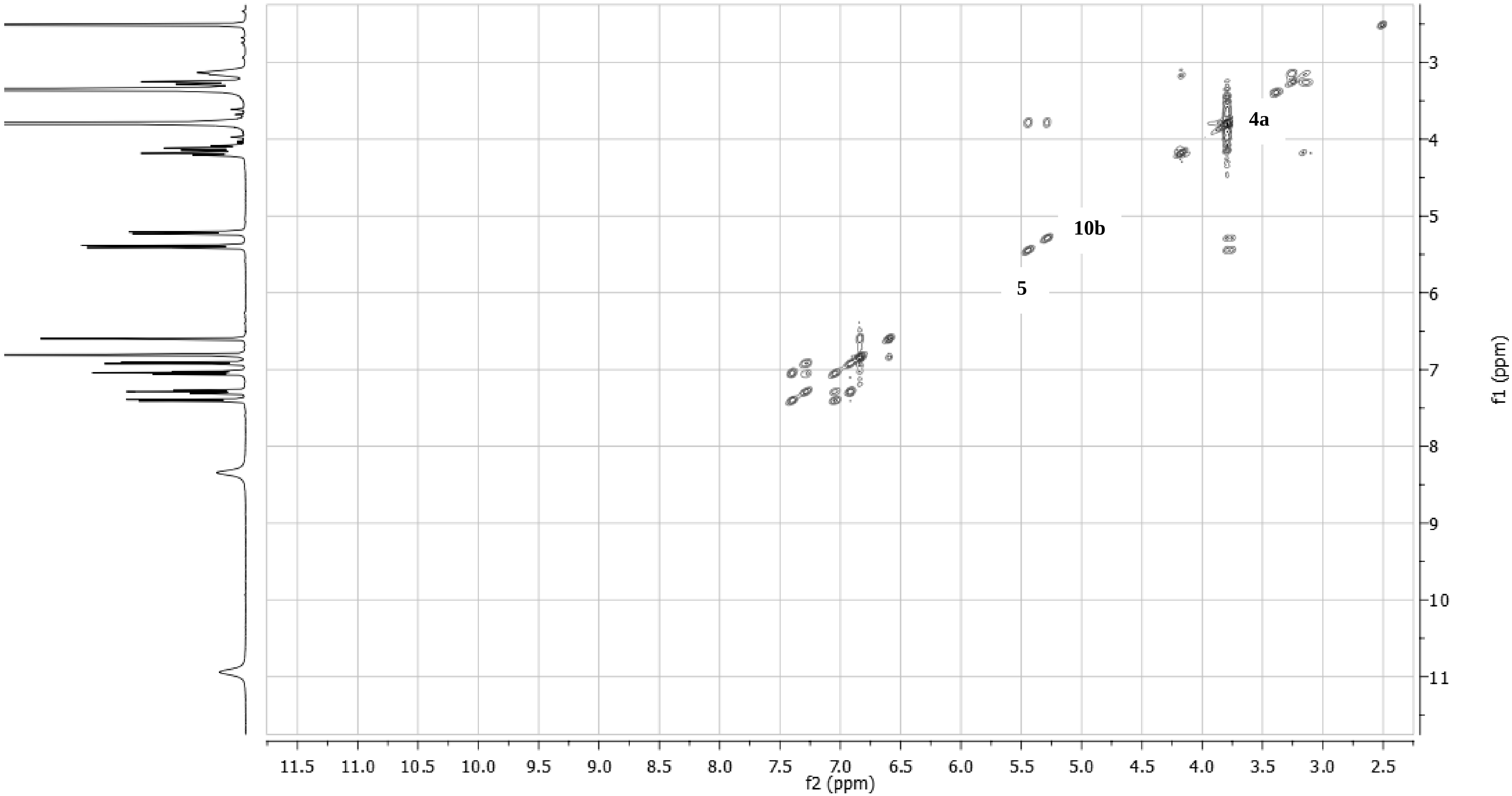

Figure S180. COSY spectrum of *rac*-(4a*R**,5*S**,10a*S**)-**2d** in DMSO-d_6

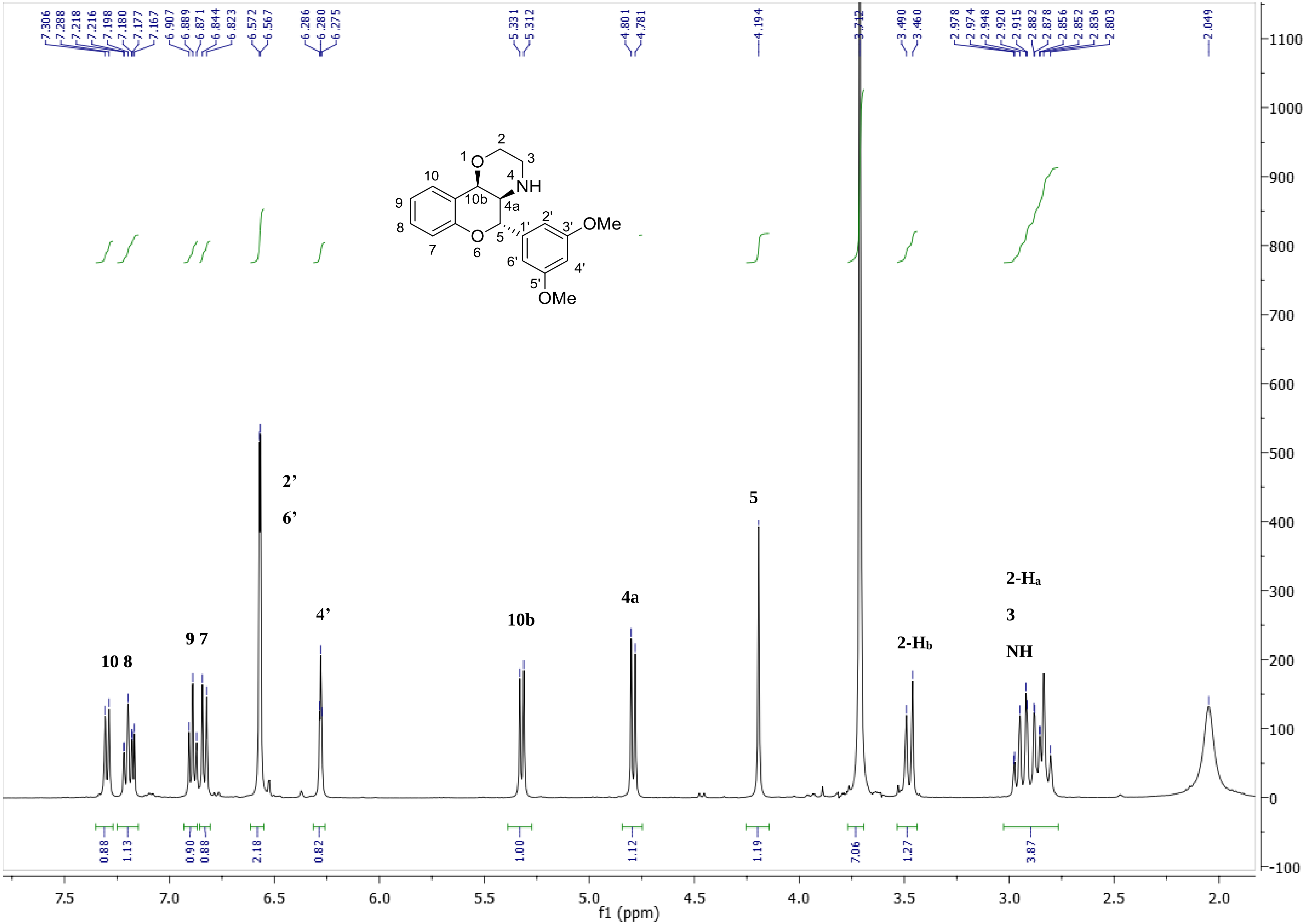

Figure S181. ^{1}H-NMR spectrum of *rac*-(4a*R**,5*S**,10a*R**)-**2d** in $CDCl_3$

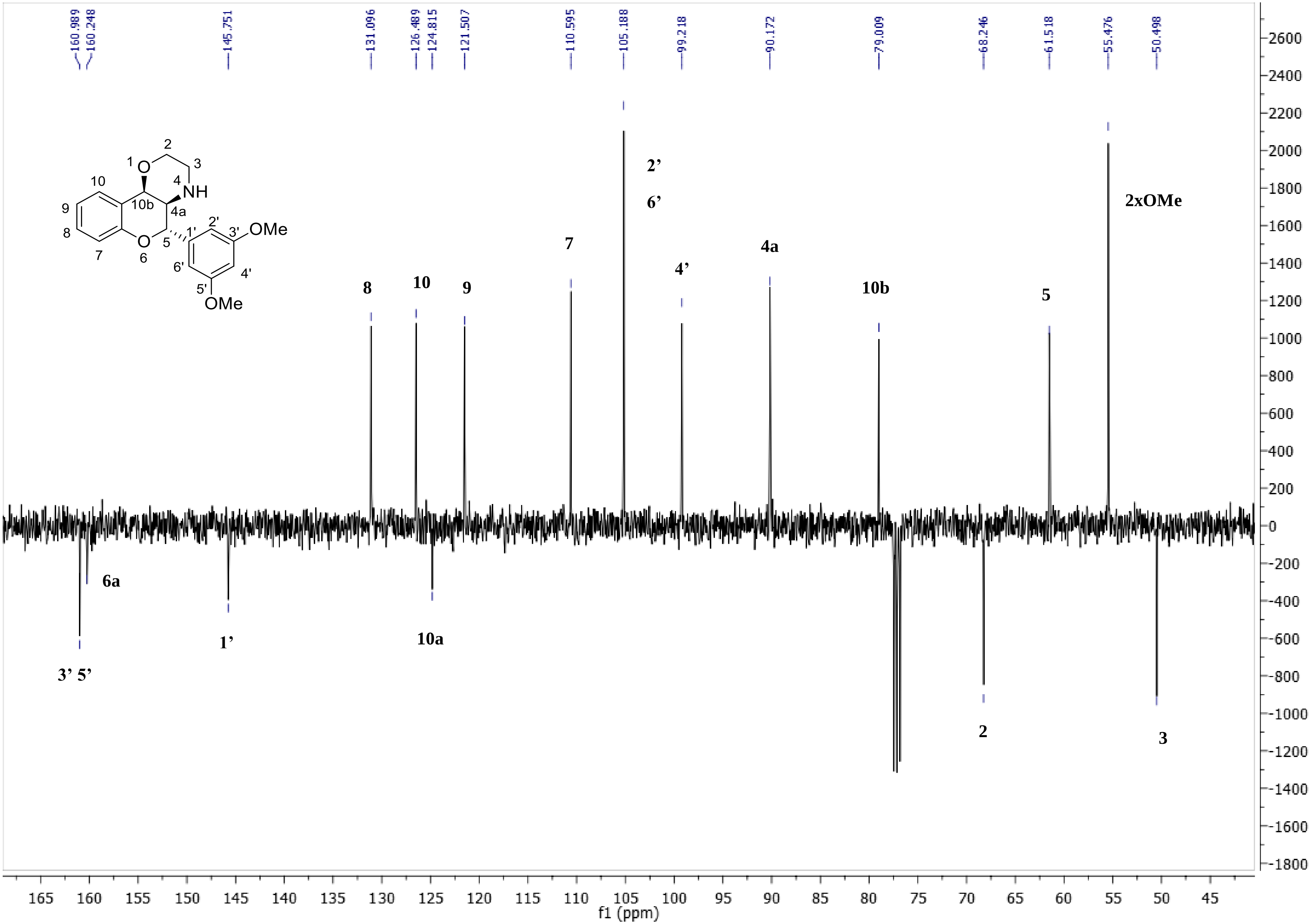

Figure S182. ^{13}C-NMR spectrum of *rac*-(4a*R**,5*S**,10a*R**)-**2d** in $CDCl_3$

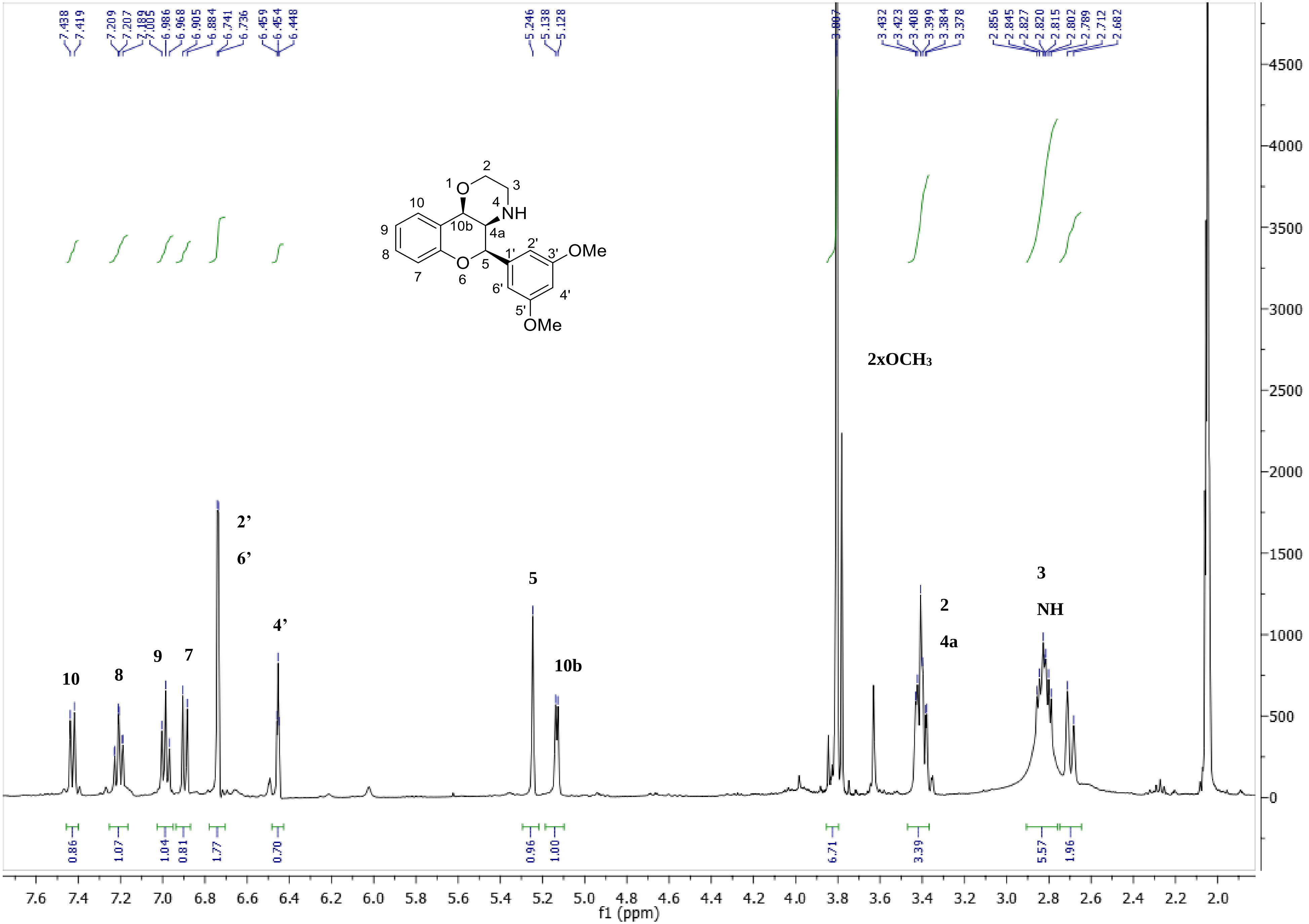

Figure S183. ^{1}H-NMR spectrum of *rac*-(4a*R**,5*R**,10a*R**)-**2d** in Acetone-d$_6$

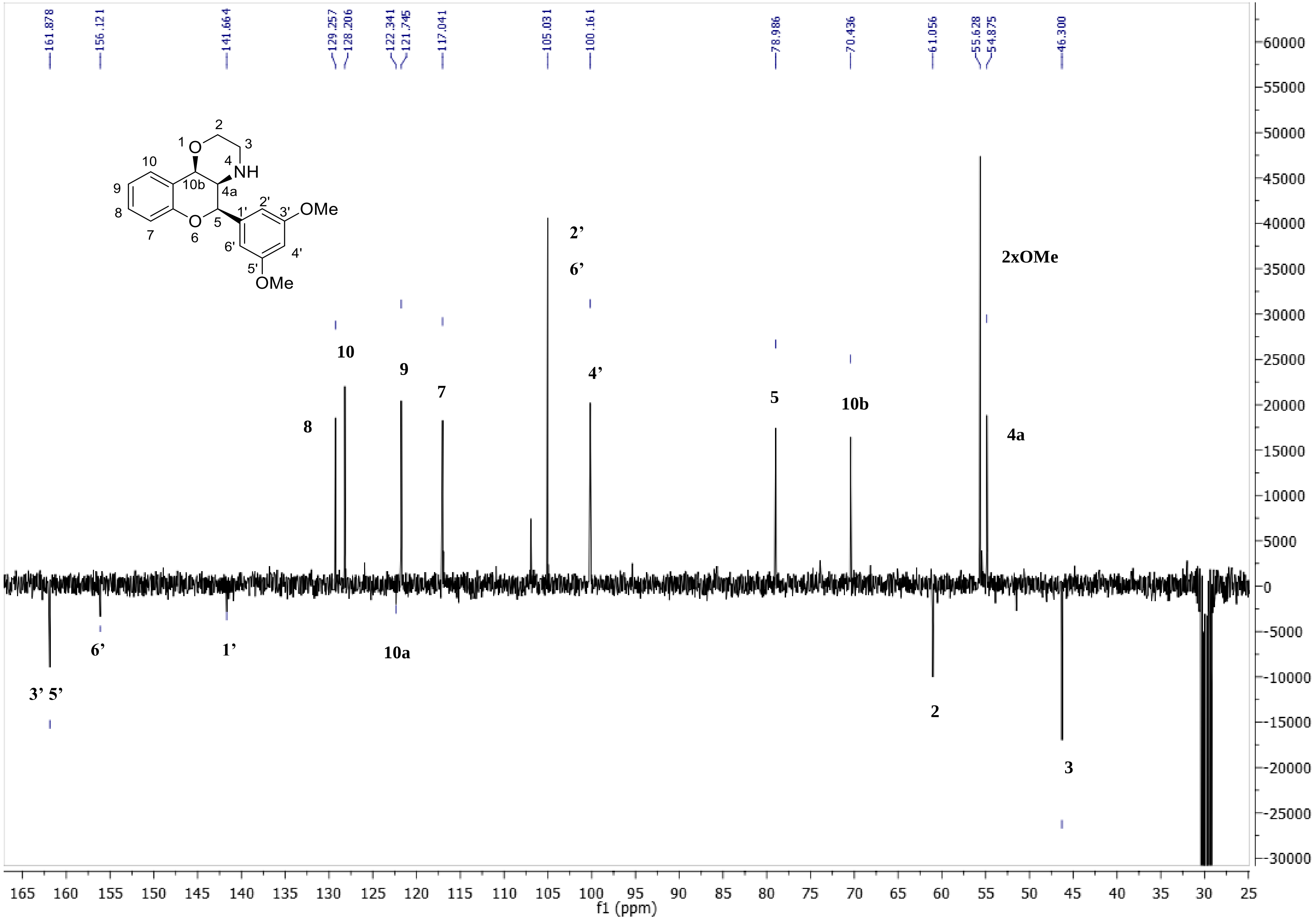

Figure S184. ^{13}C-NMR spectrum of *rac*-(4a*R**,5*R**,10a*R**)-**2d** in Acetone-d_6

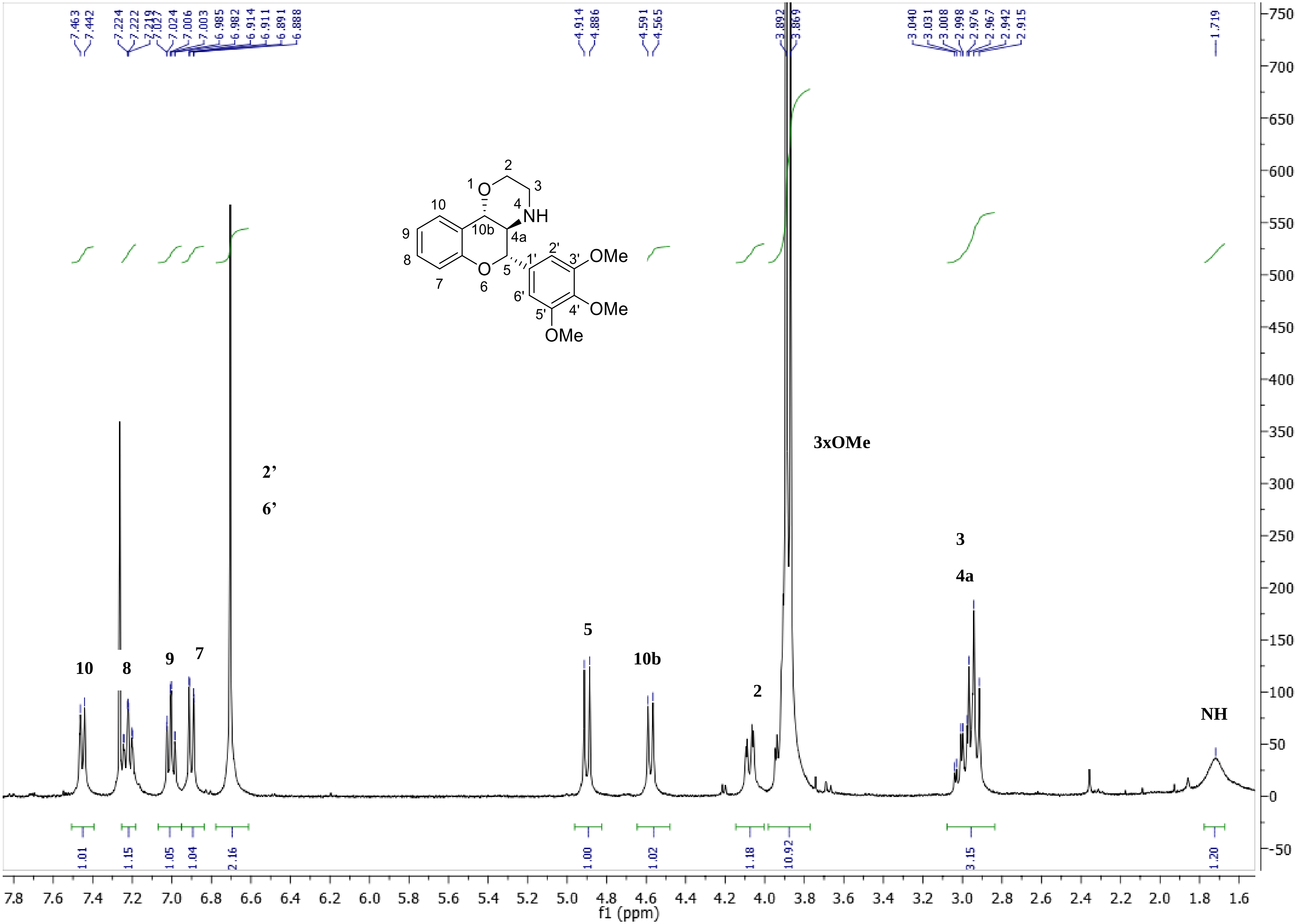

Figure S185. ^{1}H-NMR spectrum of *rac*-(4a*R**,5*S**,10a*S**)-**2e** in $CDCl_3$

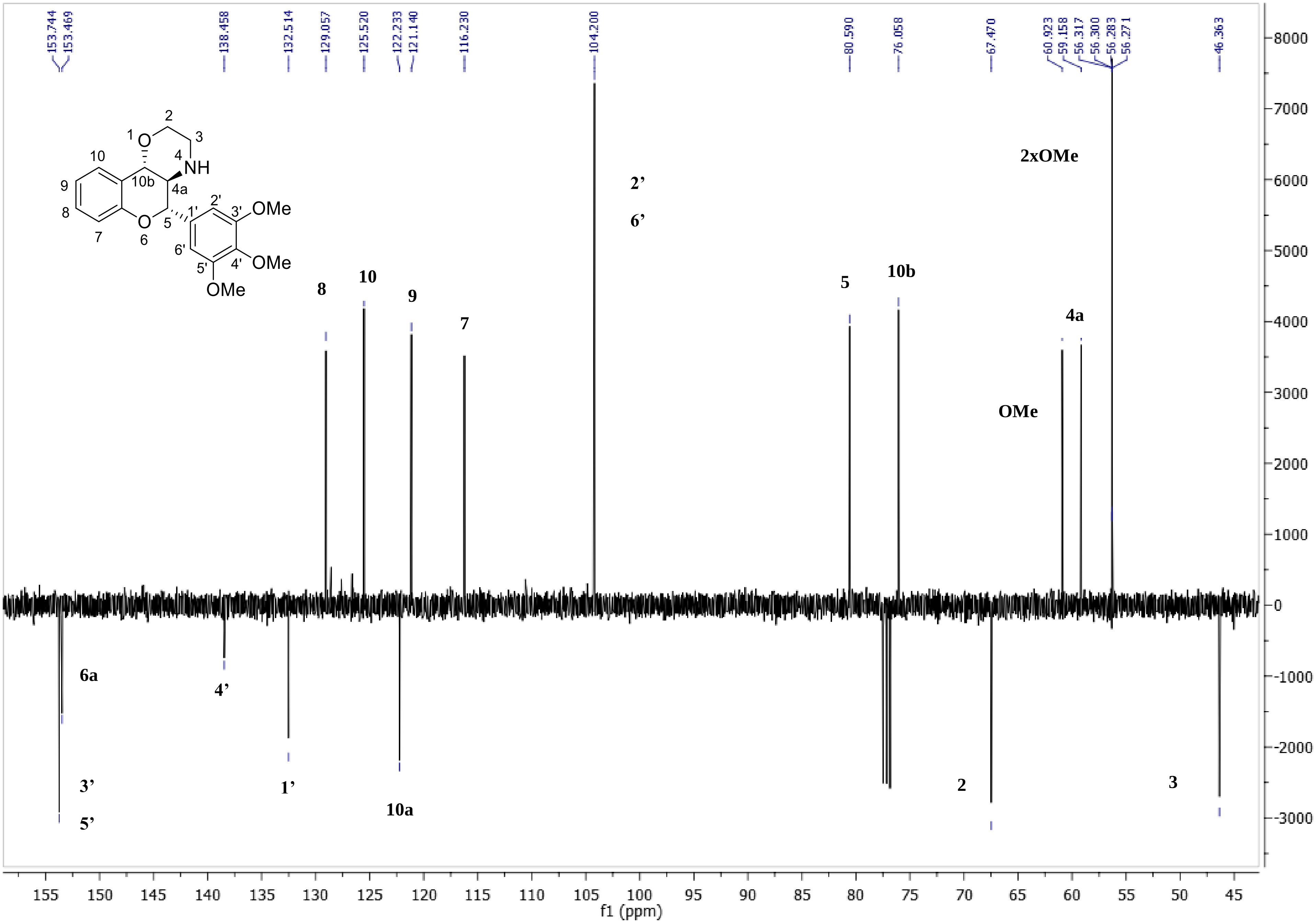

Figure S186. ^{13}C-NMR spectrum of *rac*-(4a*R**,5*S**,10a*S**)-**2e** in $CDCl_3$

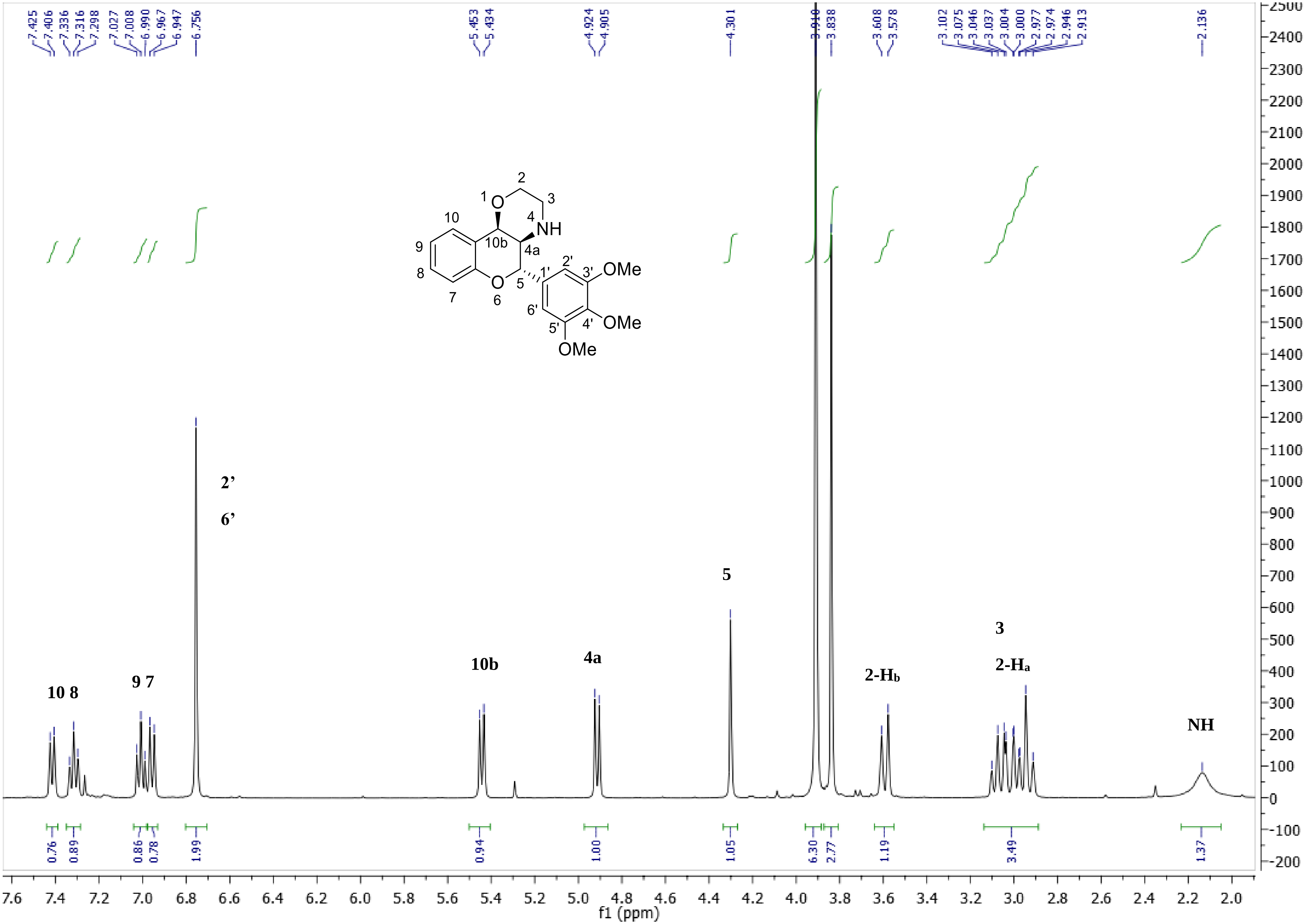

Figure S187. ^{1}H-NMR spectrum of *rac*-(4a*R**,5*S**,10a*R**)-**2e** in $CDCl_3$

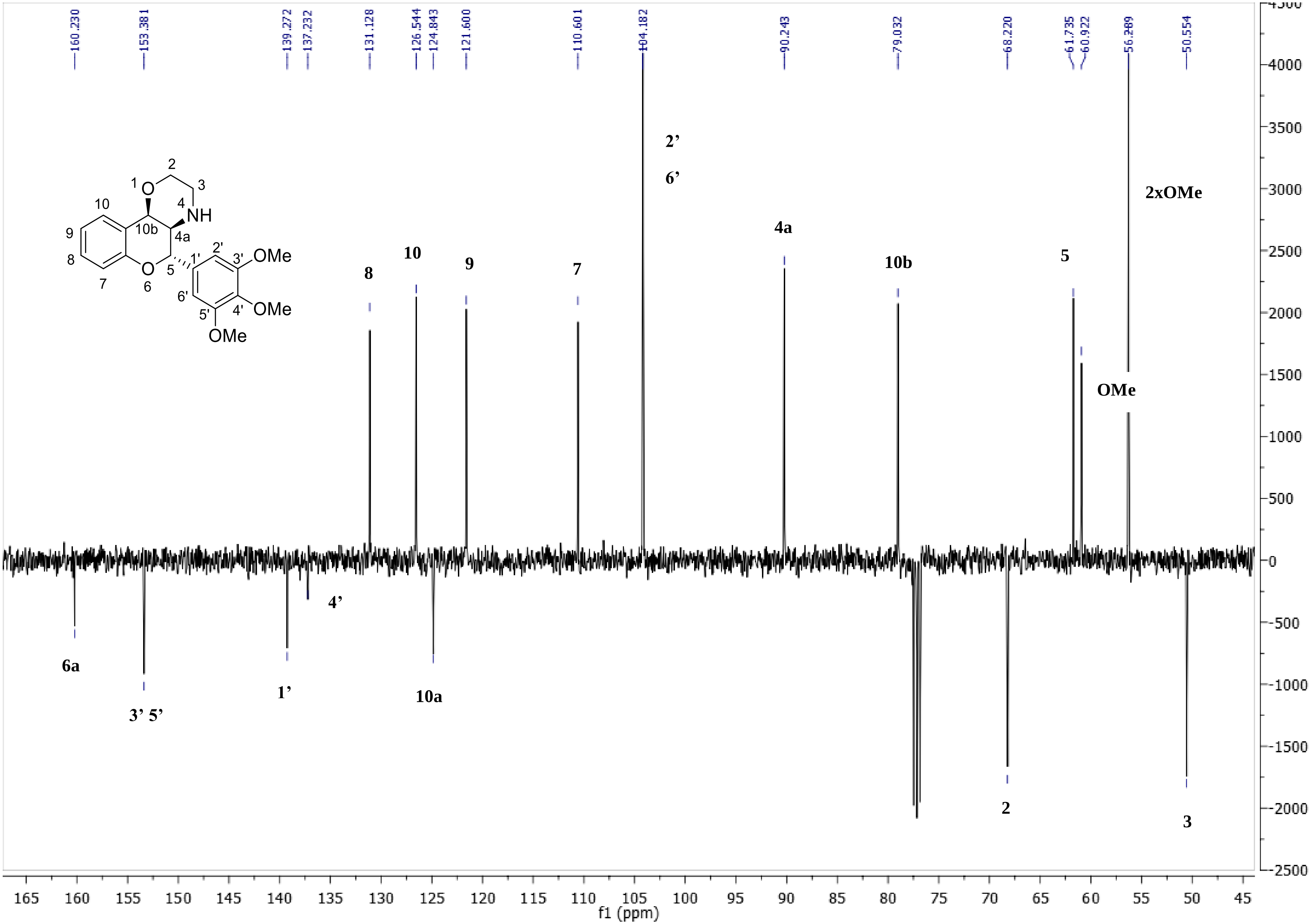

Figure S188. ^{13}C-NMR spectrum of *rac*-(4a*R**,5*S**,10a*R**)-**2e** in $CDCl_3$

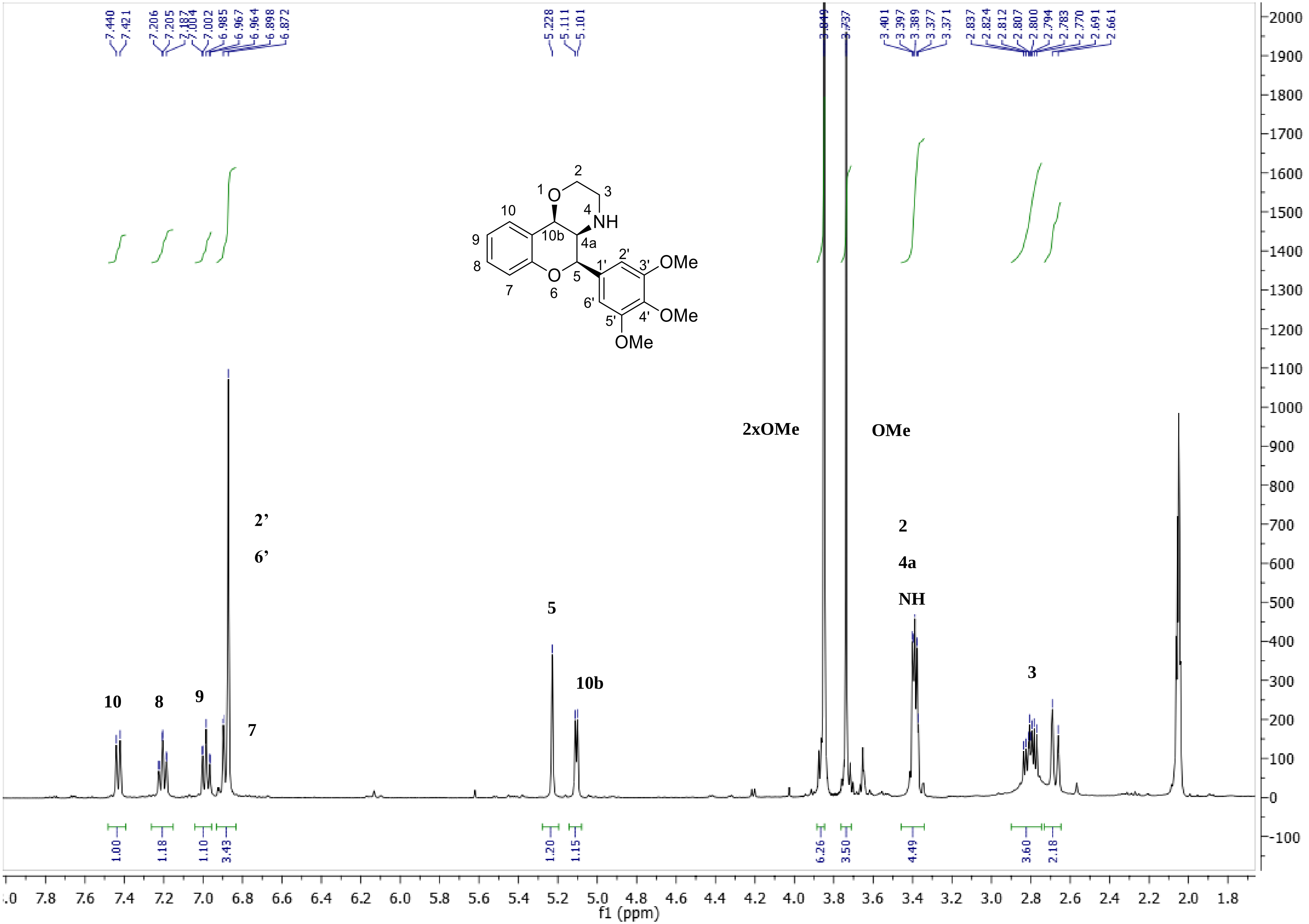

Figure S189. ^{1}H-NMR spectrum of *rac*-(4a*R**,5*R**,10a*R**)-**2e** in Acetone-d$_6$

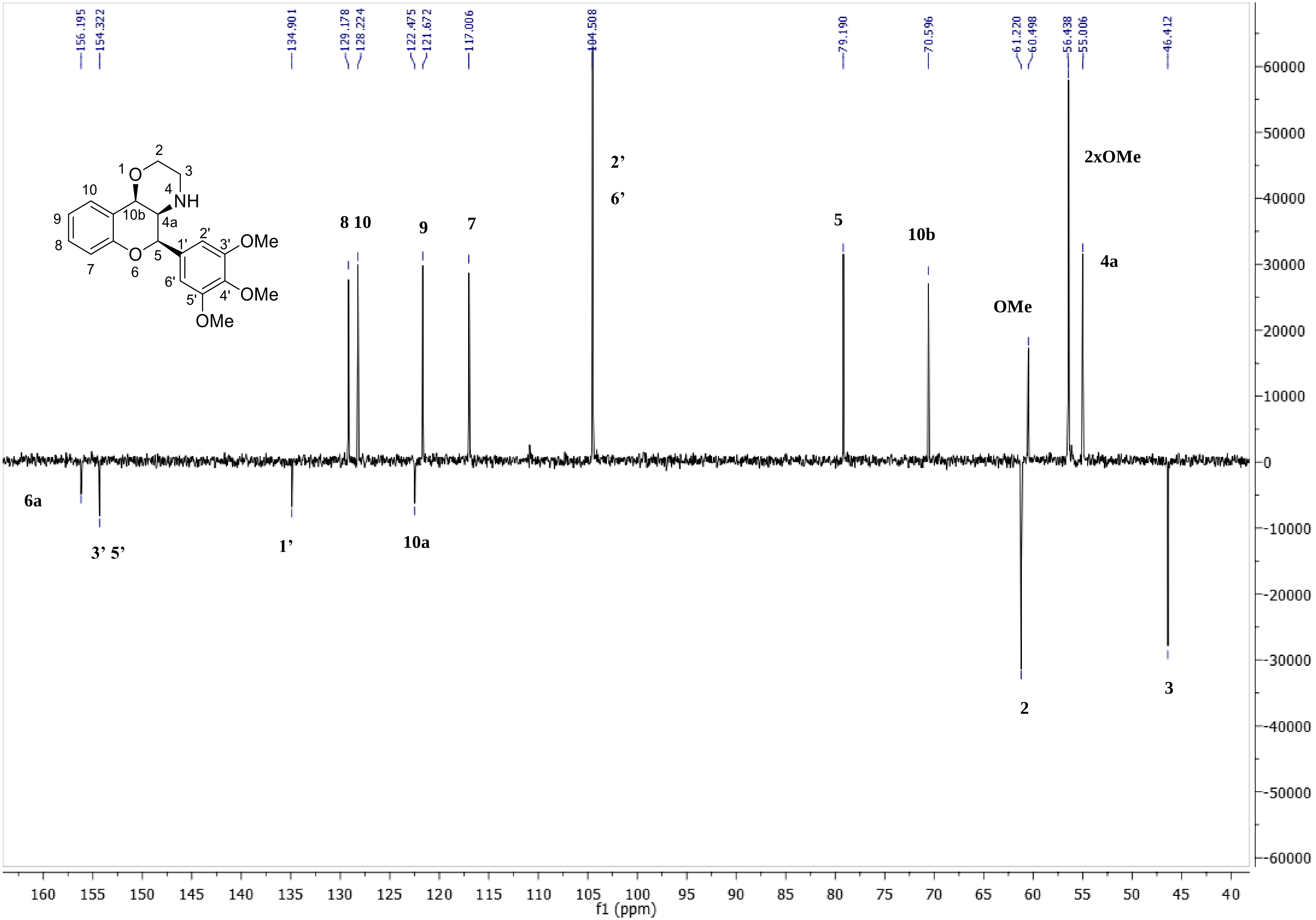

Figure S190. ^{13}C-NMR spectrum of *rac*-(4a*R**,5*R**,10a*R**)-**2e** in Acetone-d_6

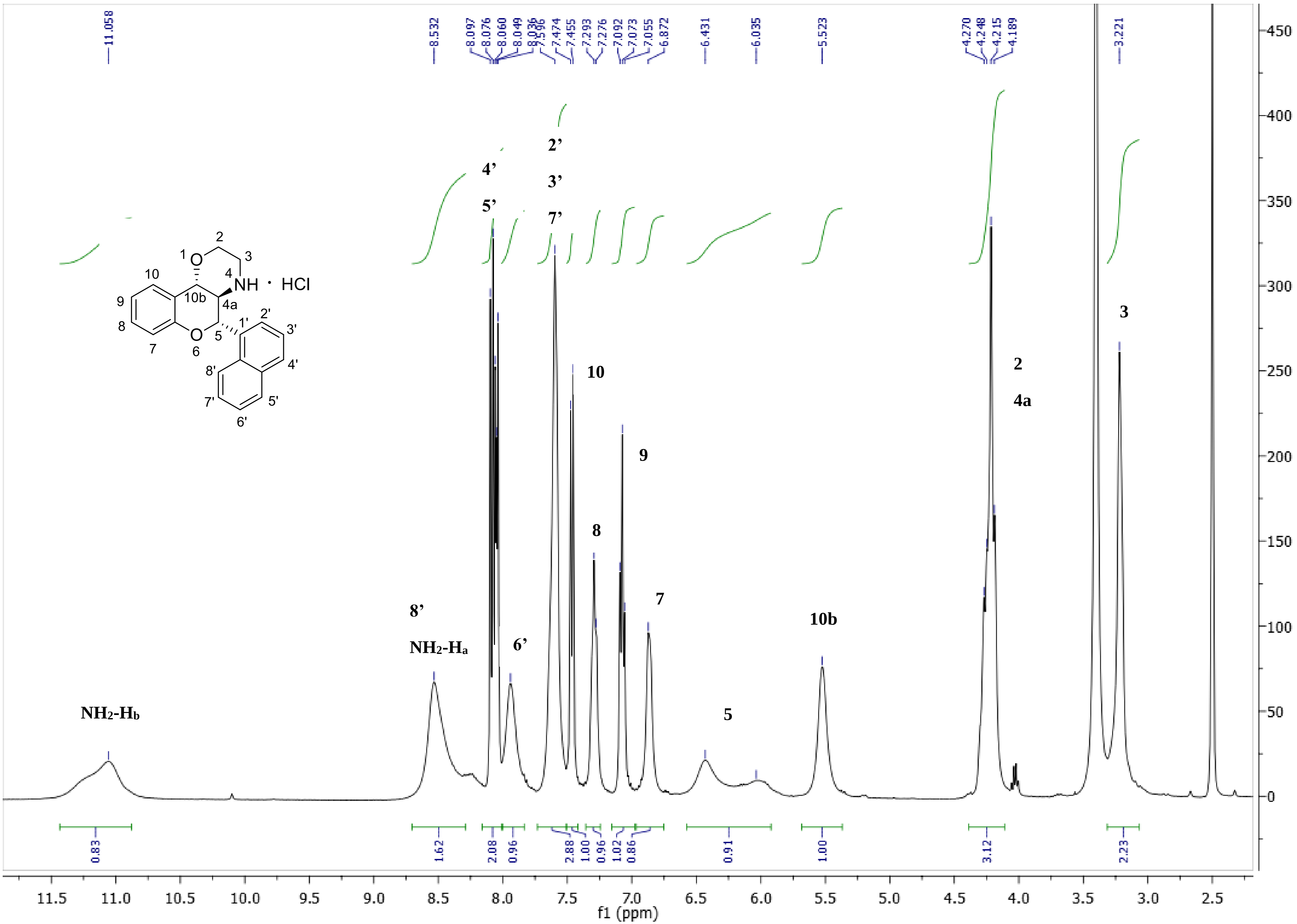

Figure S191. ^{1}H-NMR spectrum of *rac*-(4a*R**,5*S**,10a*S**)-**2f** in DMSO-d$_6$

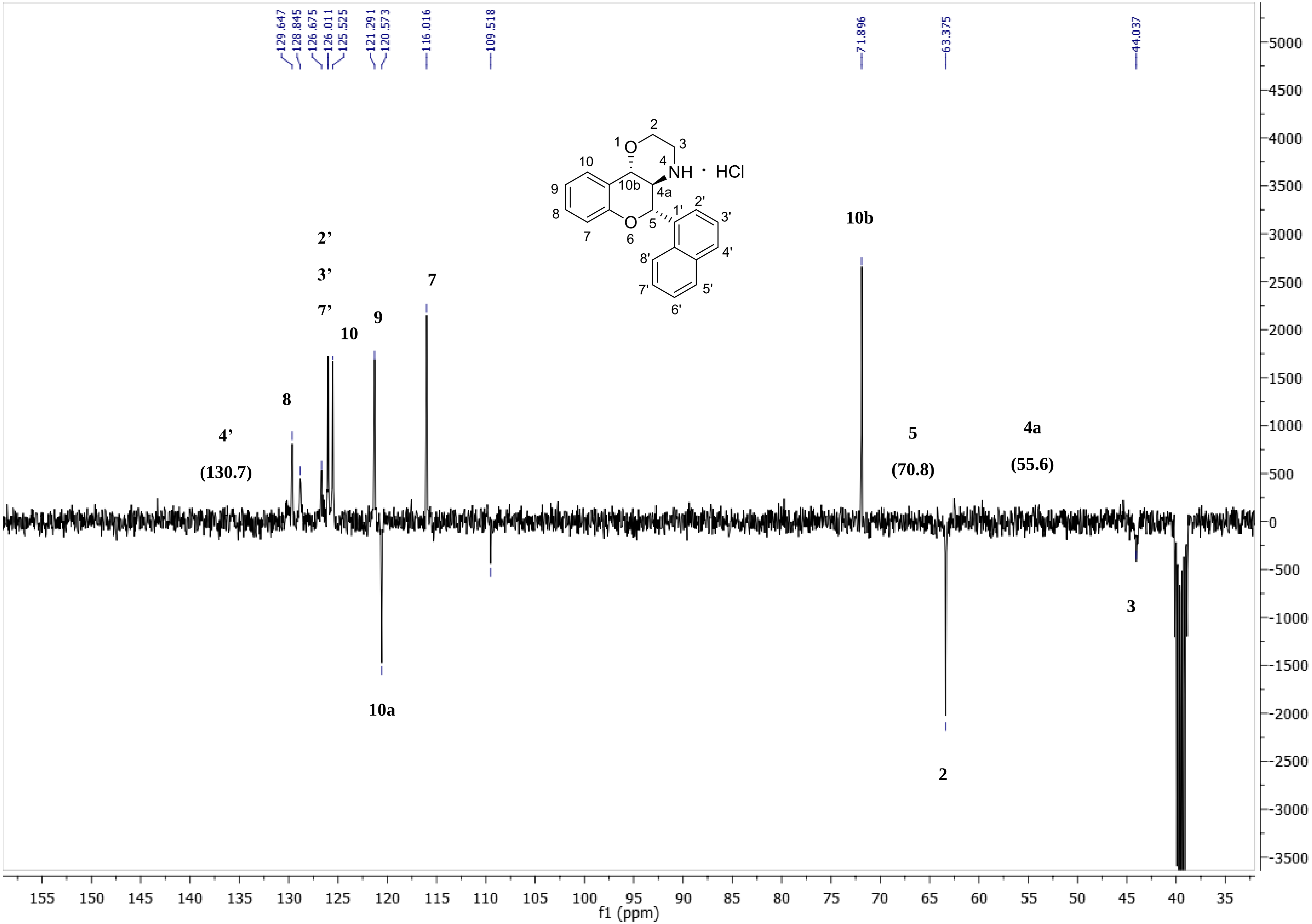

Figure S192. ^{13}C-NMR spectrum of *rac*-(4a*R**,5*S**,10a*S**)-**2f** in DMSO-d$_6$

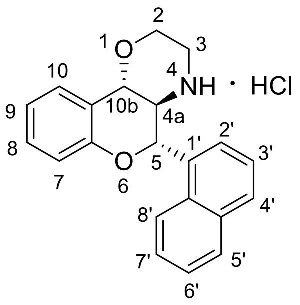

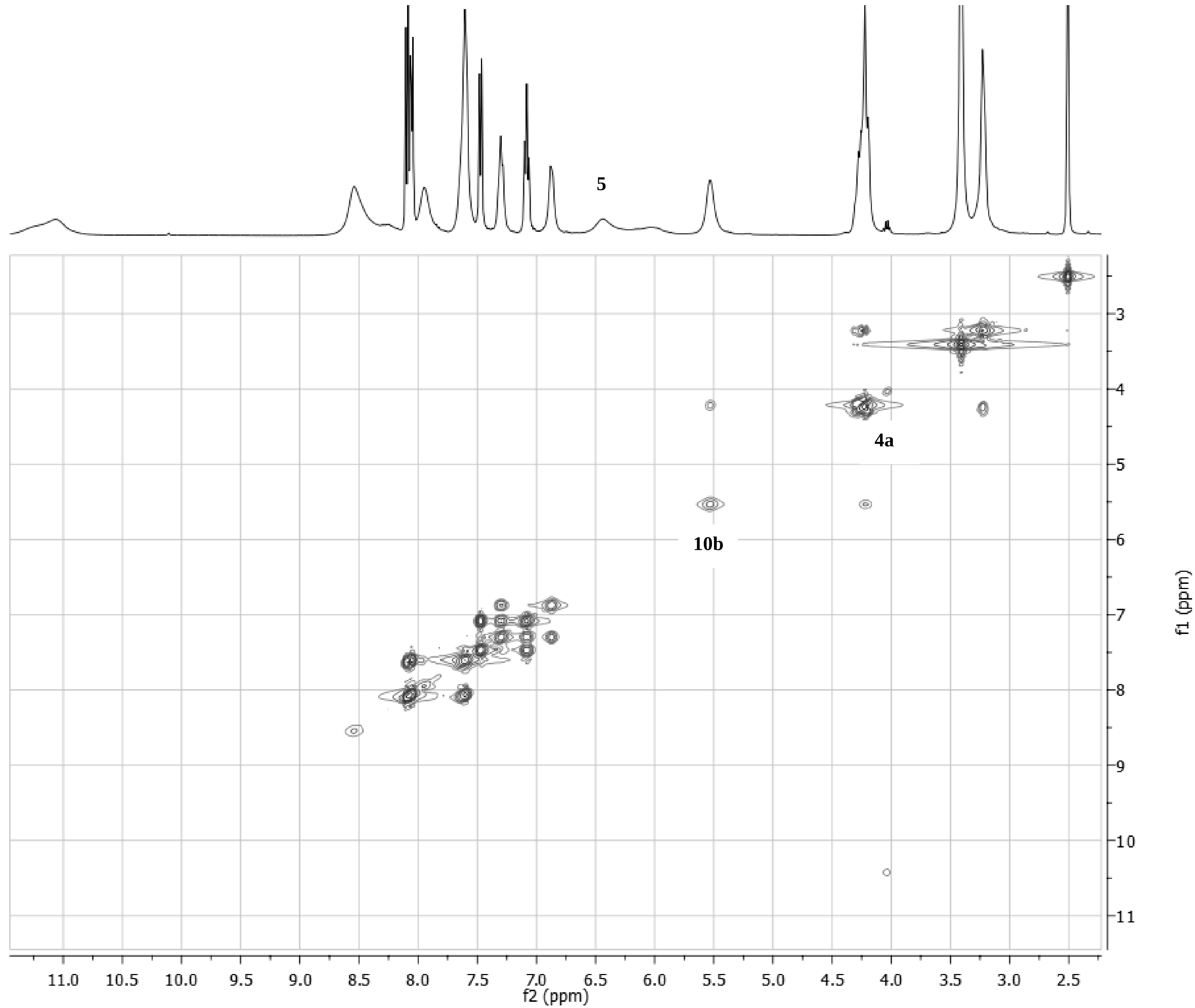

Figure S193. COSY spectrum of *rac*-(4a*R**,5*S**,10a*S**)-**2f** in DMSO-d_6

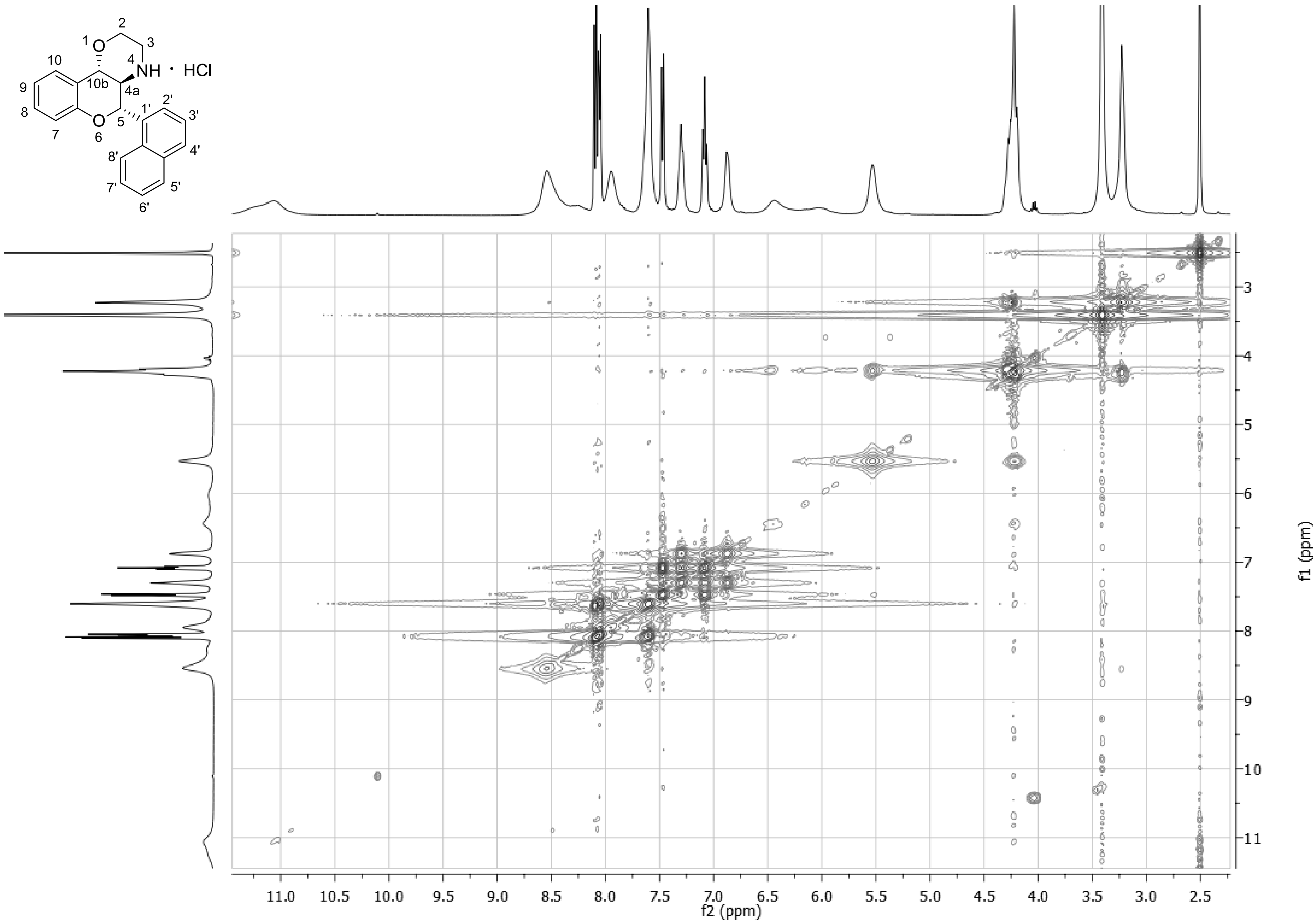

Figure S194. COSY spectrum of *rac*-(4a*R**,5*S**,10a*S**)-**2f** in DMSO-d$_6$ (maximized)

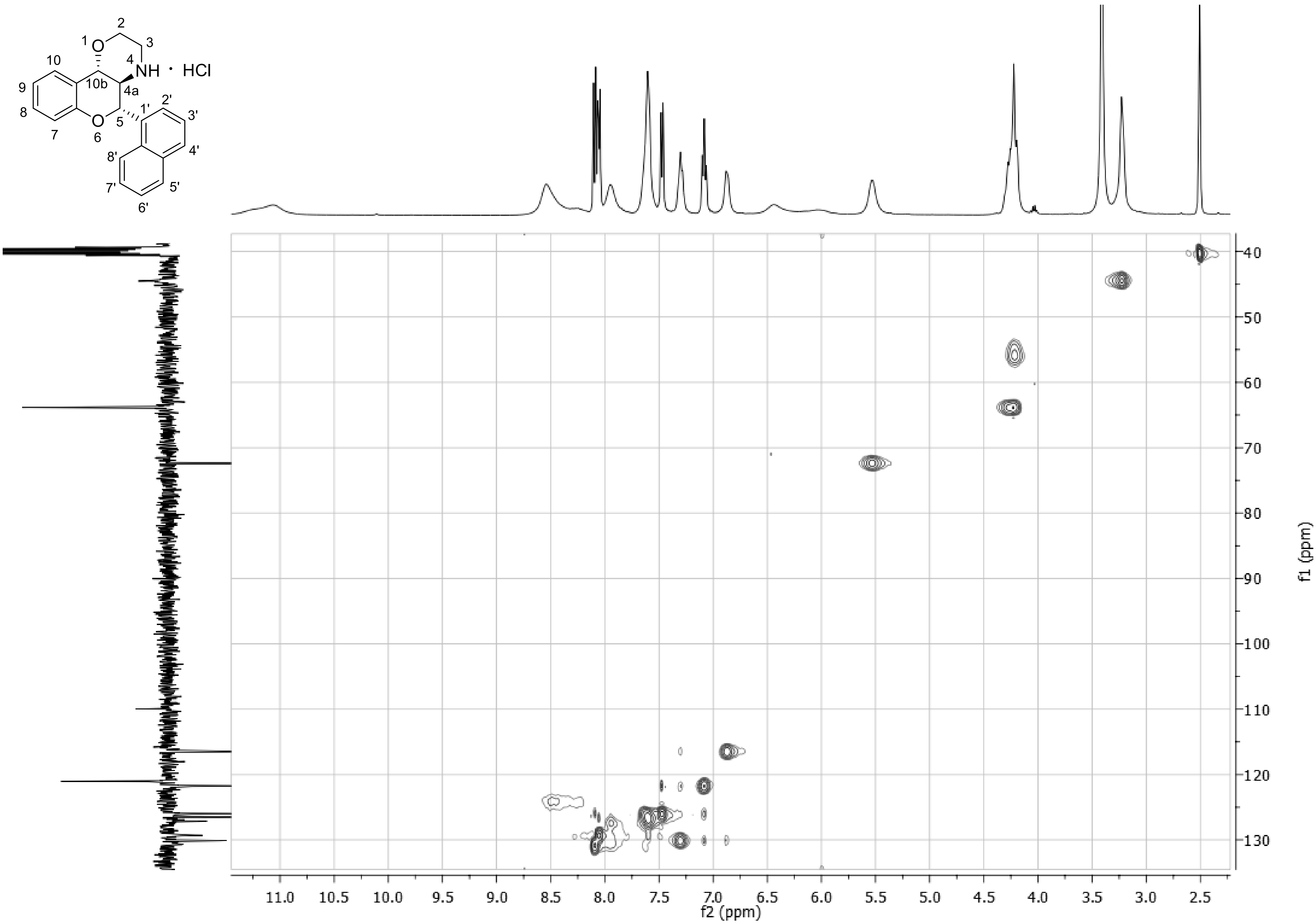

Figure S195. HSQC spectrum of *rac*-(4a*R**,5*S**,10a*S**)-**2f** in DMSO-d$_6$

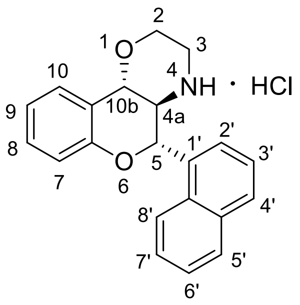

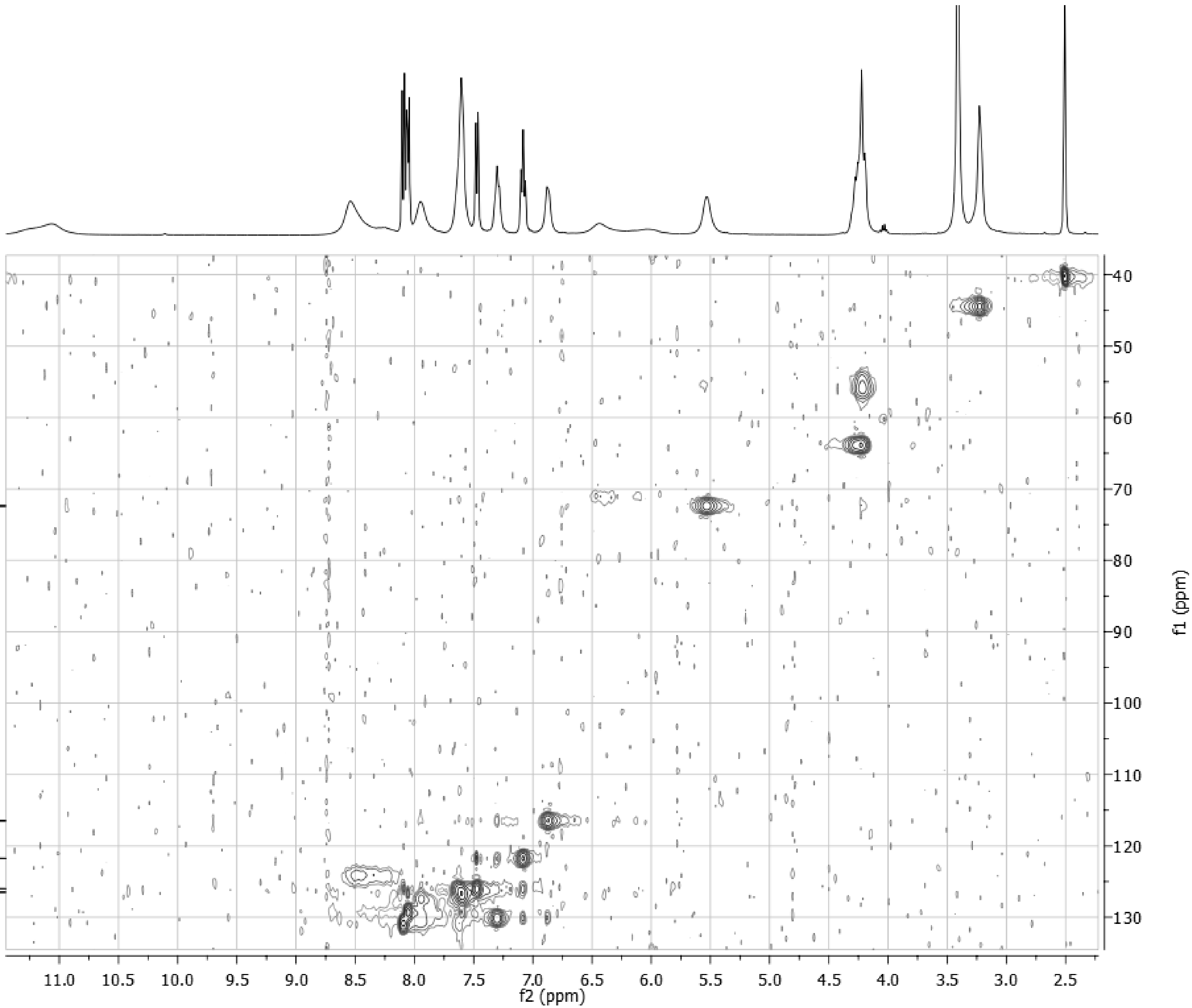

Figure S196. HSQC spectrum of *rac*-(4a*R**,5*S**,10a*S**)-**2f** in DMSO-d_6 (maximized)

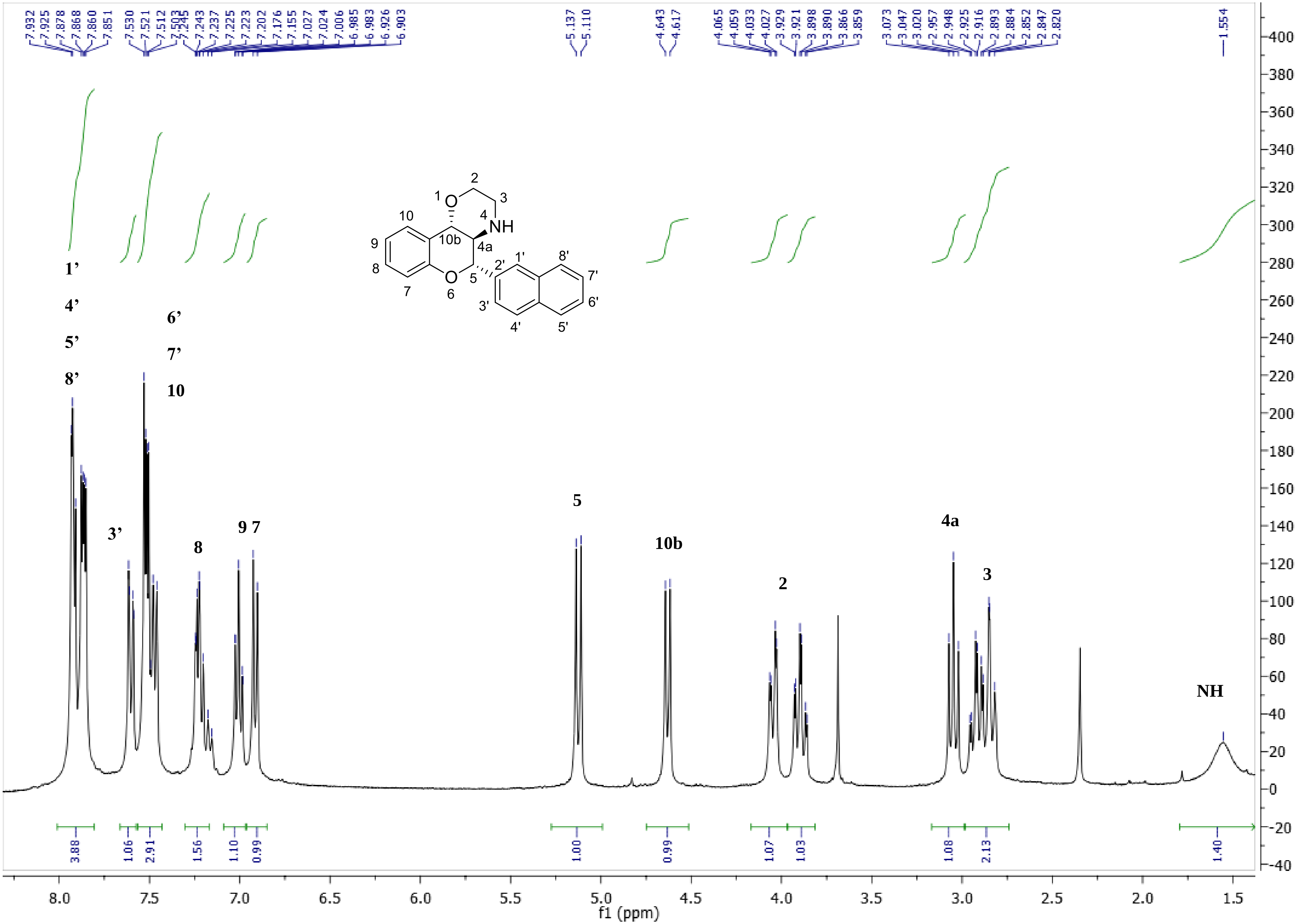

Figure S197. ^{1}H-NMR spectrum of *rac*-(4a*R**,5*S**,10a*S**)-**2g** in $CDCl_3$

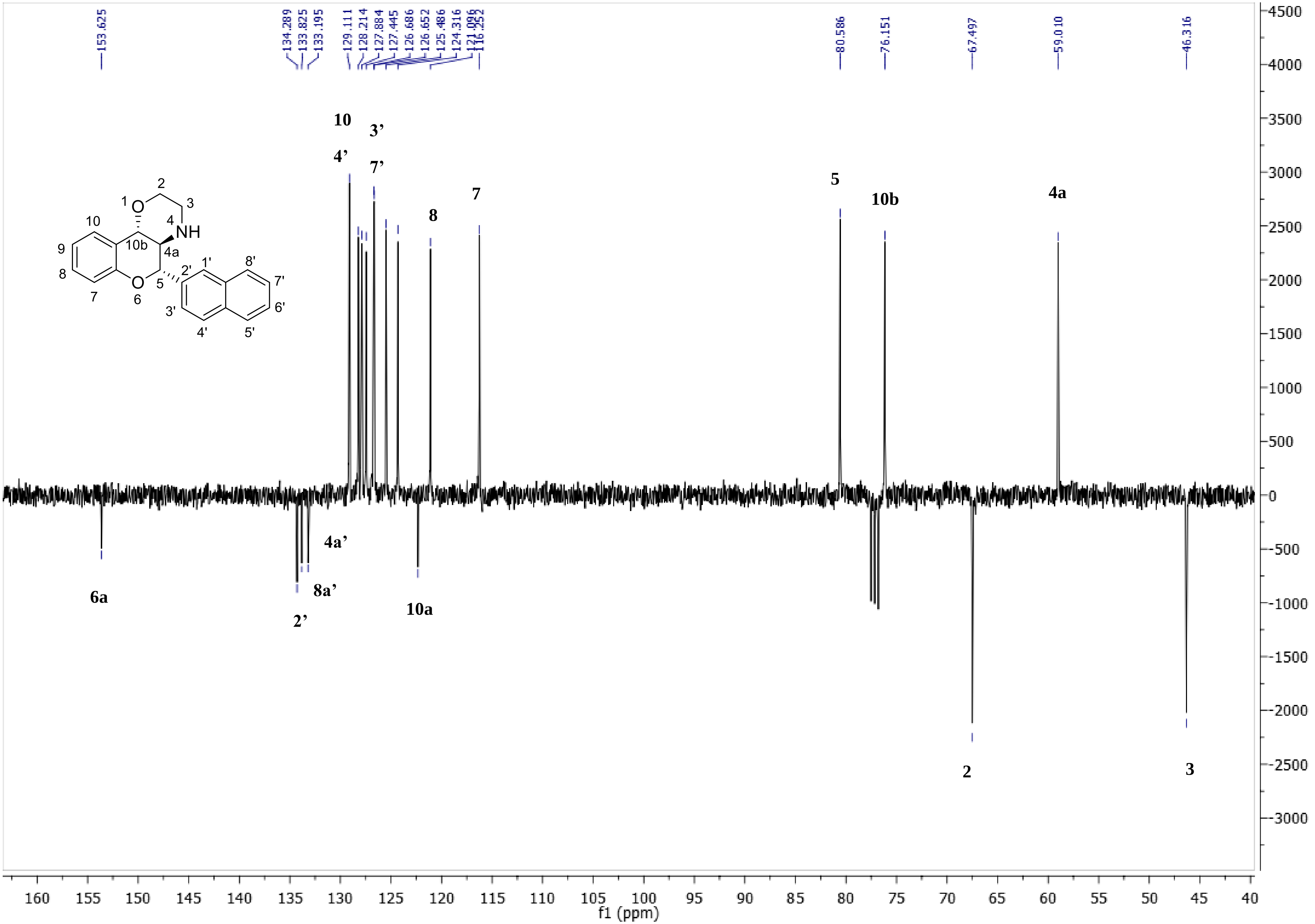

Figure S198. ^{13}C-NMR spectrum of *rac*-(4a*R**,5*S**,10a*S**)-**2g** in $CDCl_3$

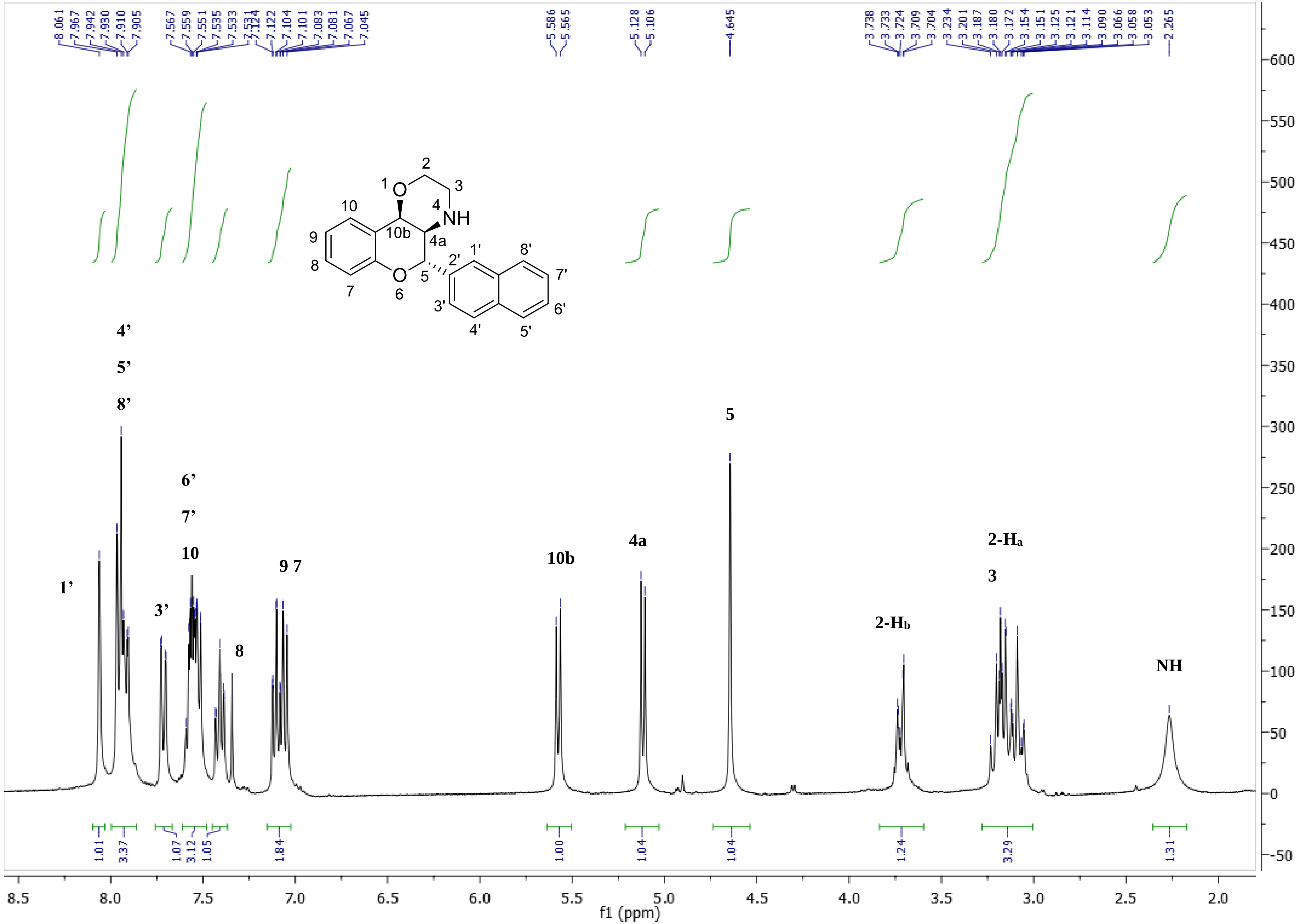

Figure S199. ^{1}H-NMR spectrum of *rac*-(4a*R**,5*S**,10a*R**)-**2g** in $CDCl_3$

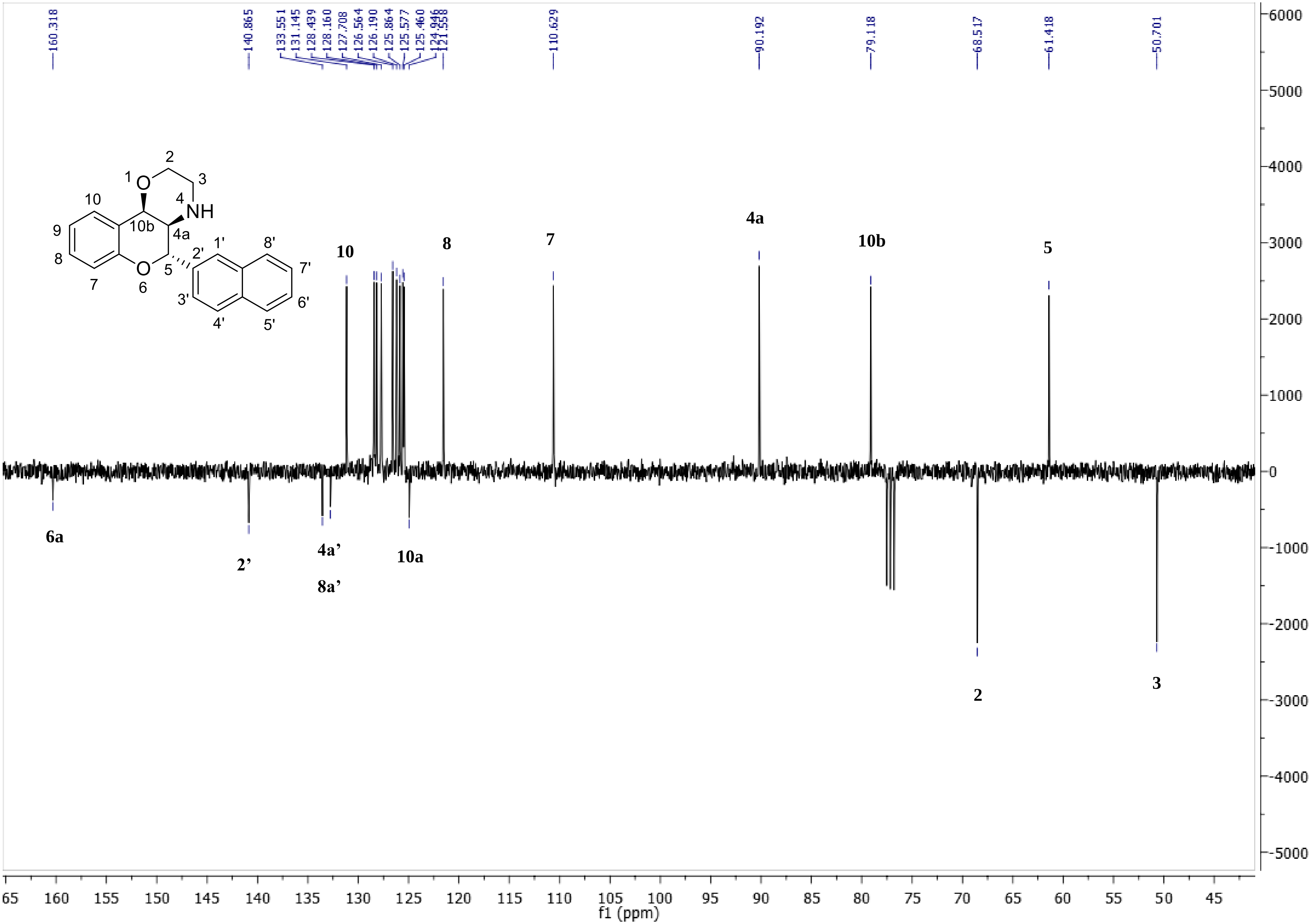

Figure S200. ^{13}C-NMR spectrum of *rac*-(4a*R**,5*S**,10a*R**)-**2g** in $CDCl_3$

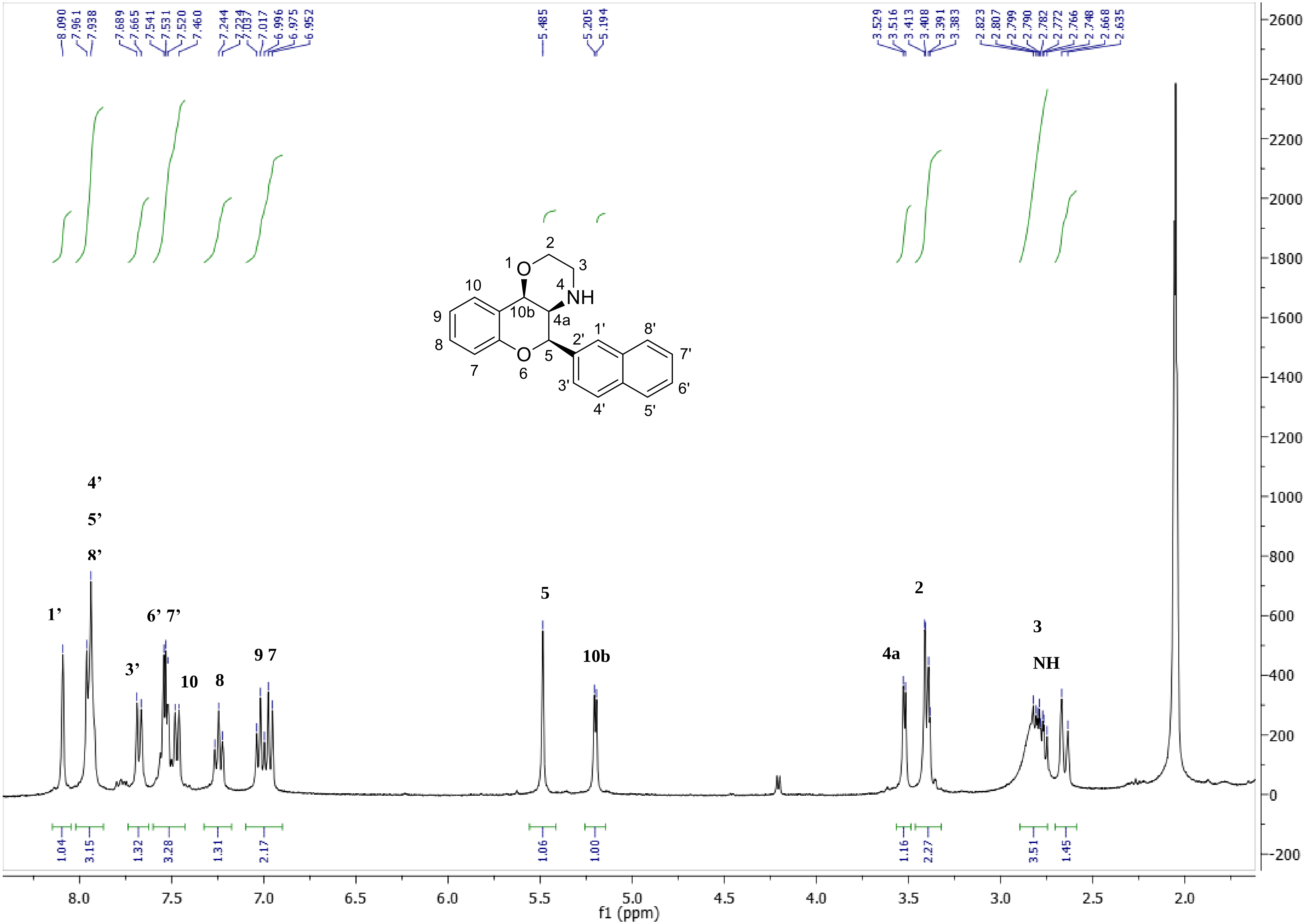

Figure S201. ^{1}H-NMR spectrum of *rac*-(4a*R**,5*R**,10a*R**)-**2g** in Acetone-d_6

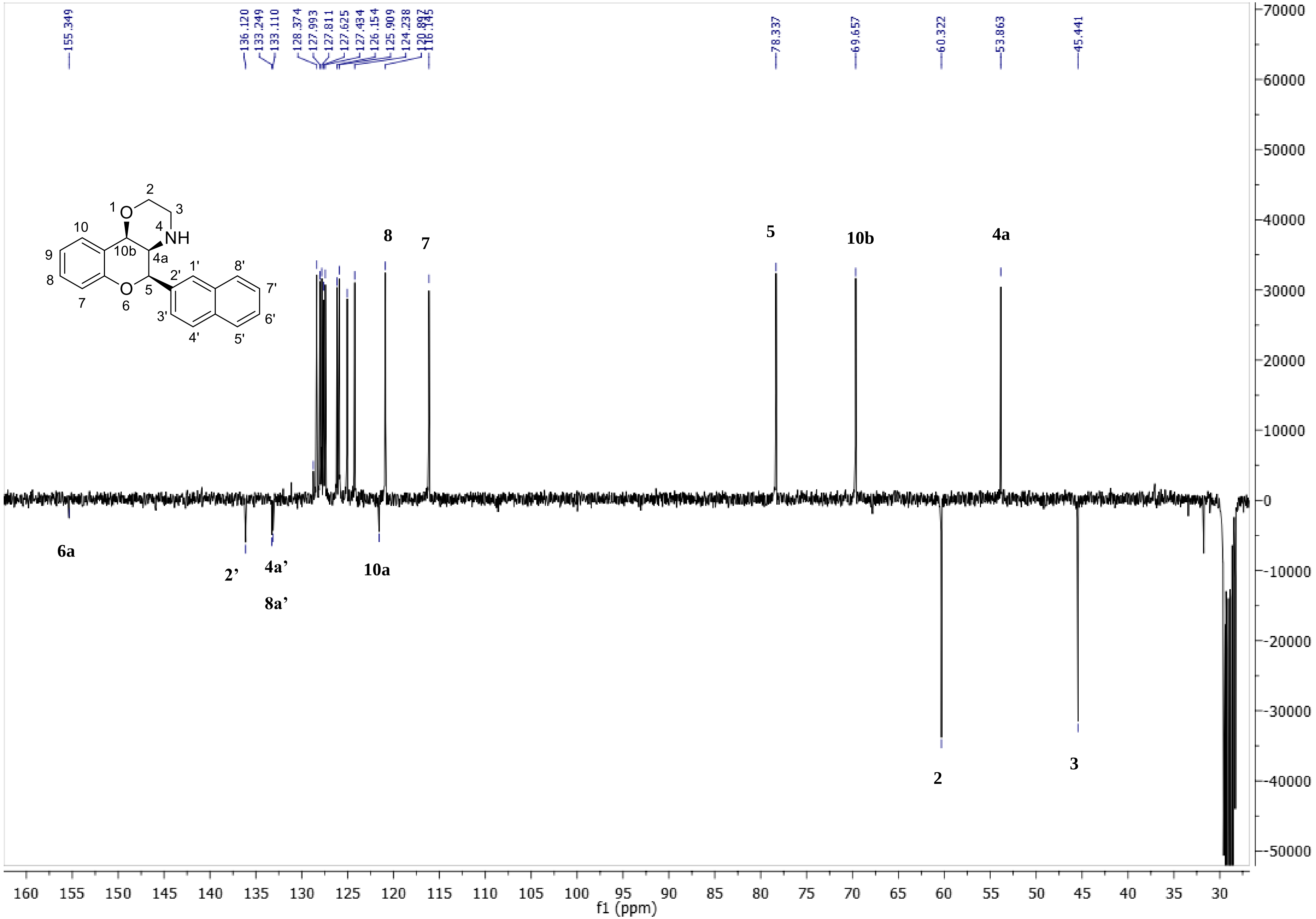

Figure S202. ^{13}C-NMR spectrum of *rac*-(4a*R**,5*R**,10a*R**)-**2g** in Acetone-d_6

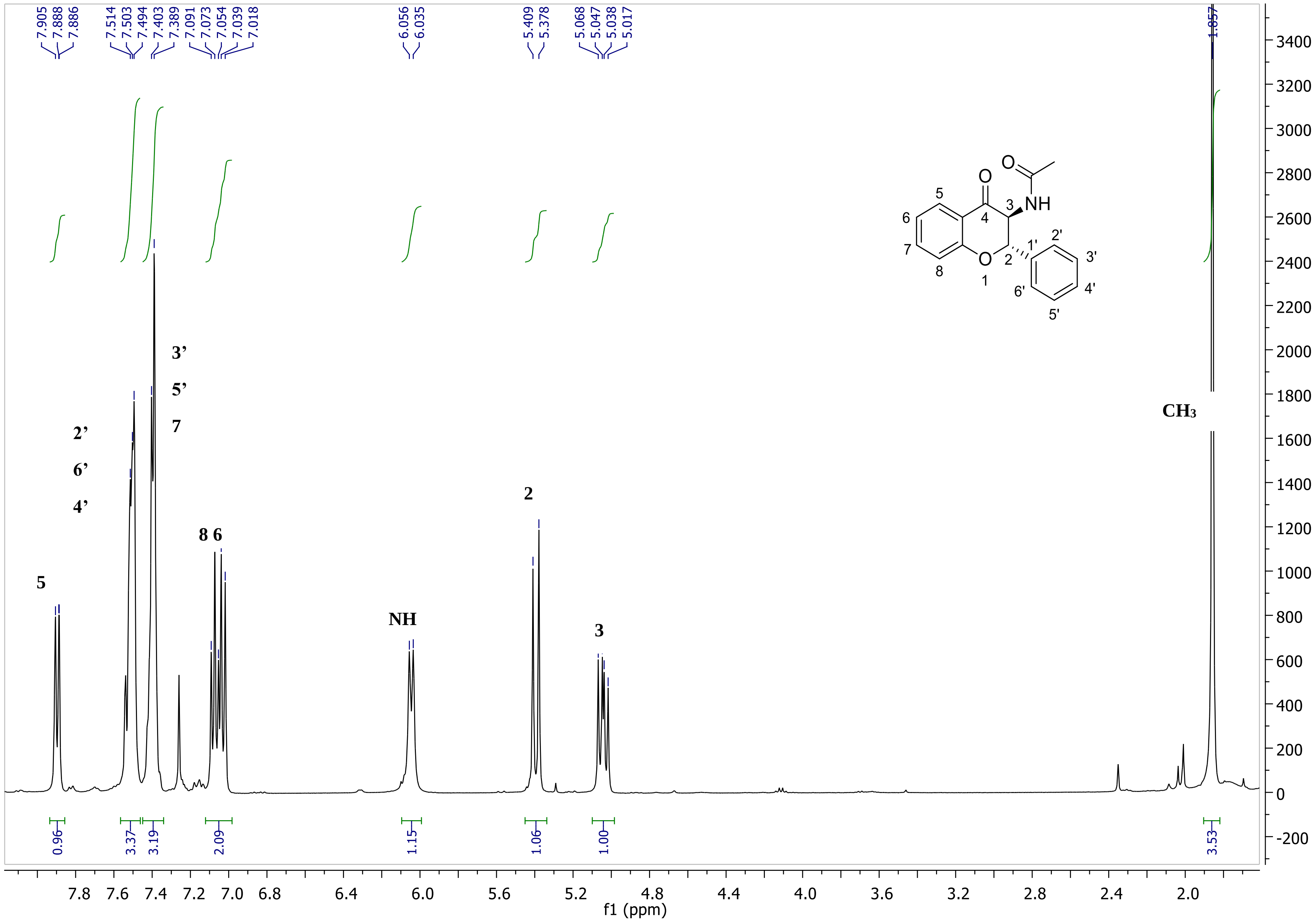

Figure S203. ^{1}H-NMR spectrum of *rac-trans*-**24a** in $CDCl_3$

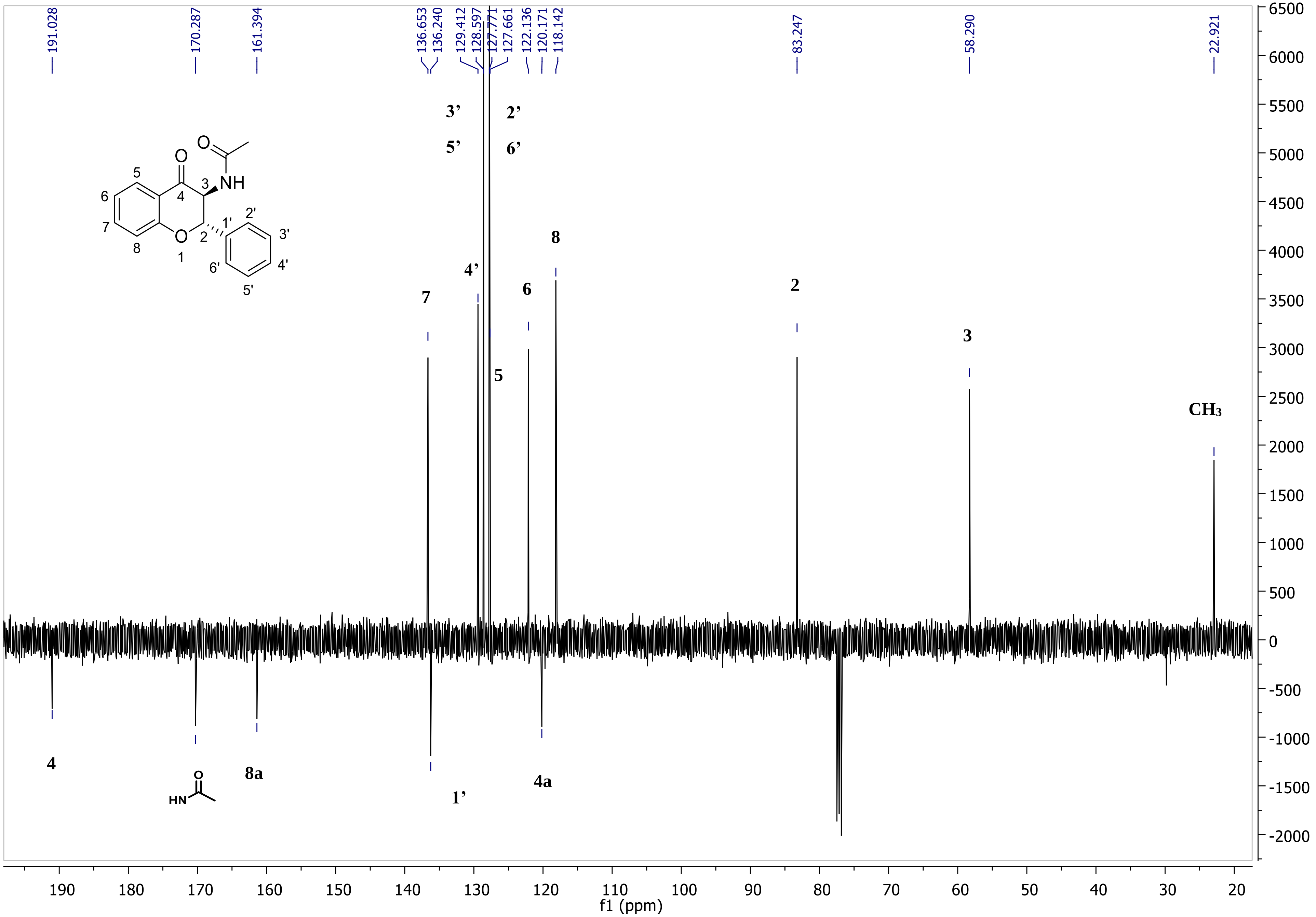

Figure S204. ^{13}C-NMR spectrum of *rac-trans*-**24a** in $CDCl_3$

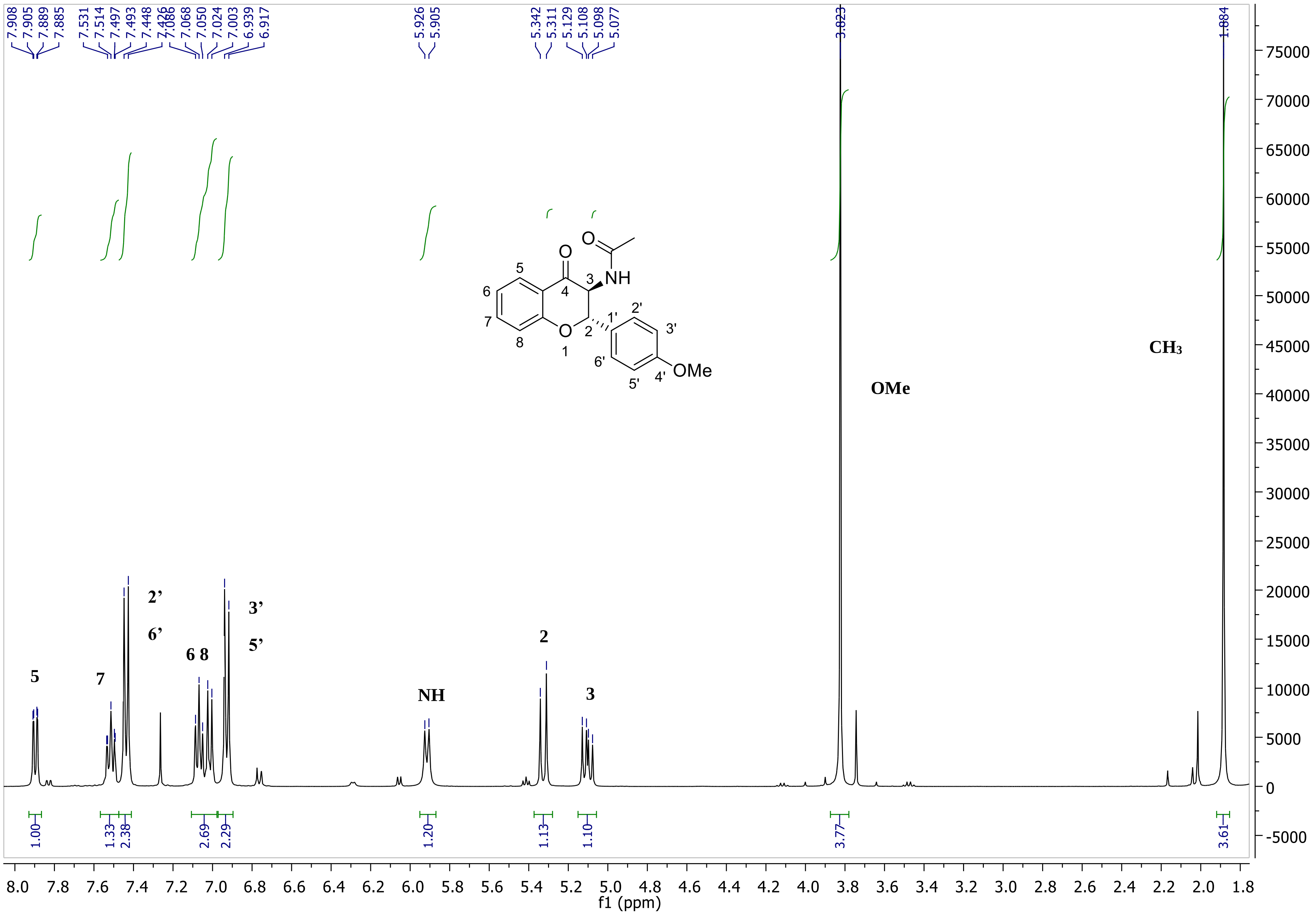

Figure S205. ^{1}H-NMR spectrum of *rac-trans*-**24b** in $CDCl_3$

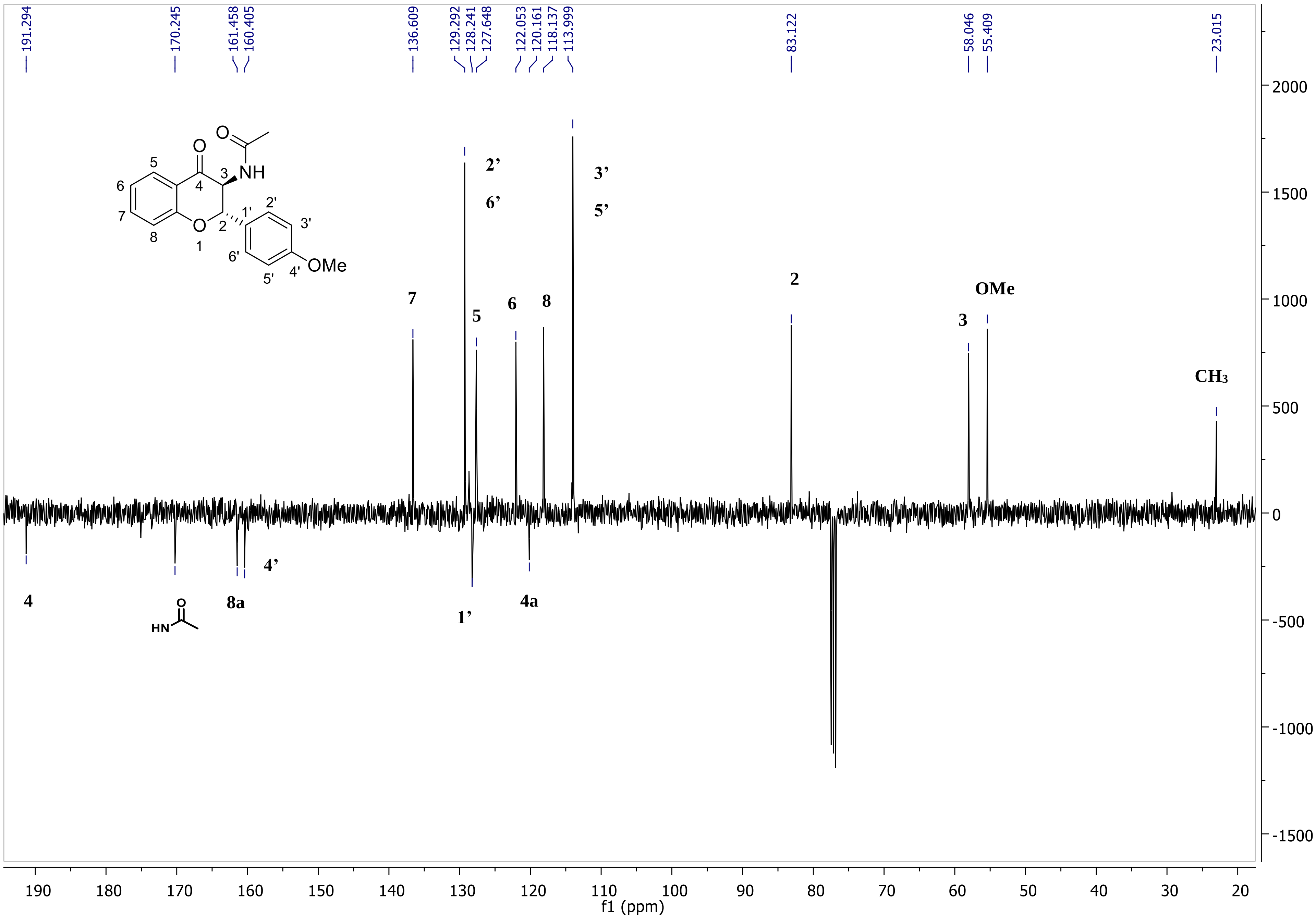

Figure S206. ^{13}C-NMR spectrum of *rac*-*trans*-**24b** in $CDCl_3$

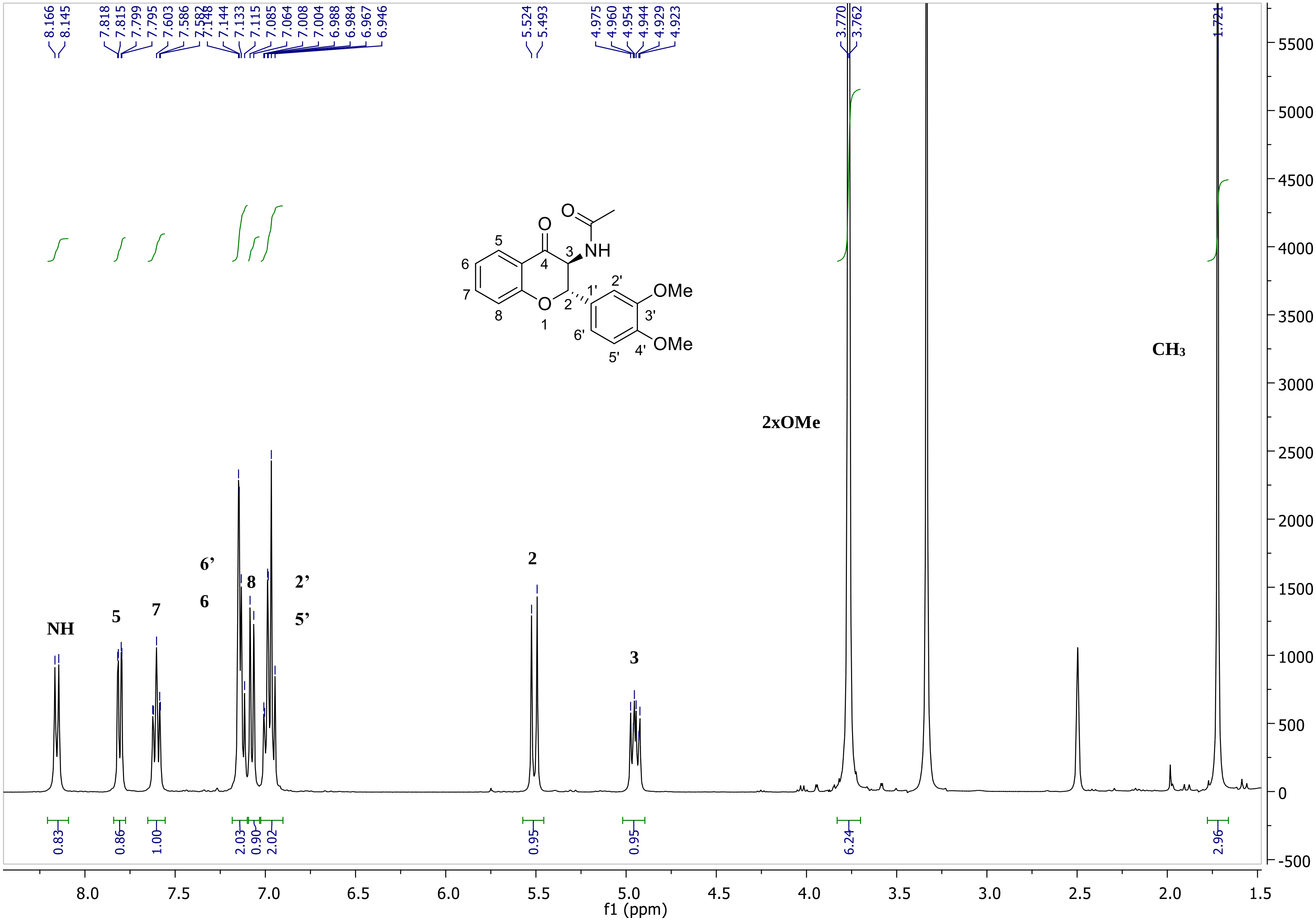

Figure S207. ^{1}H-NMR spectrum of *rac-trans*-**24c** in DMSO-d_6

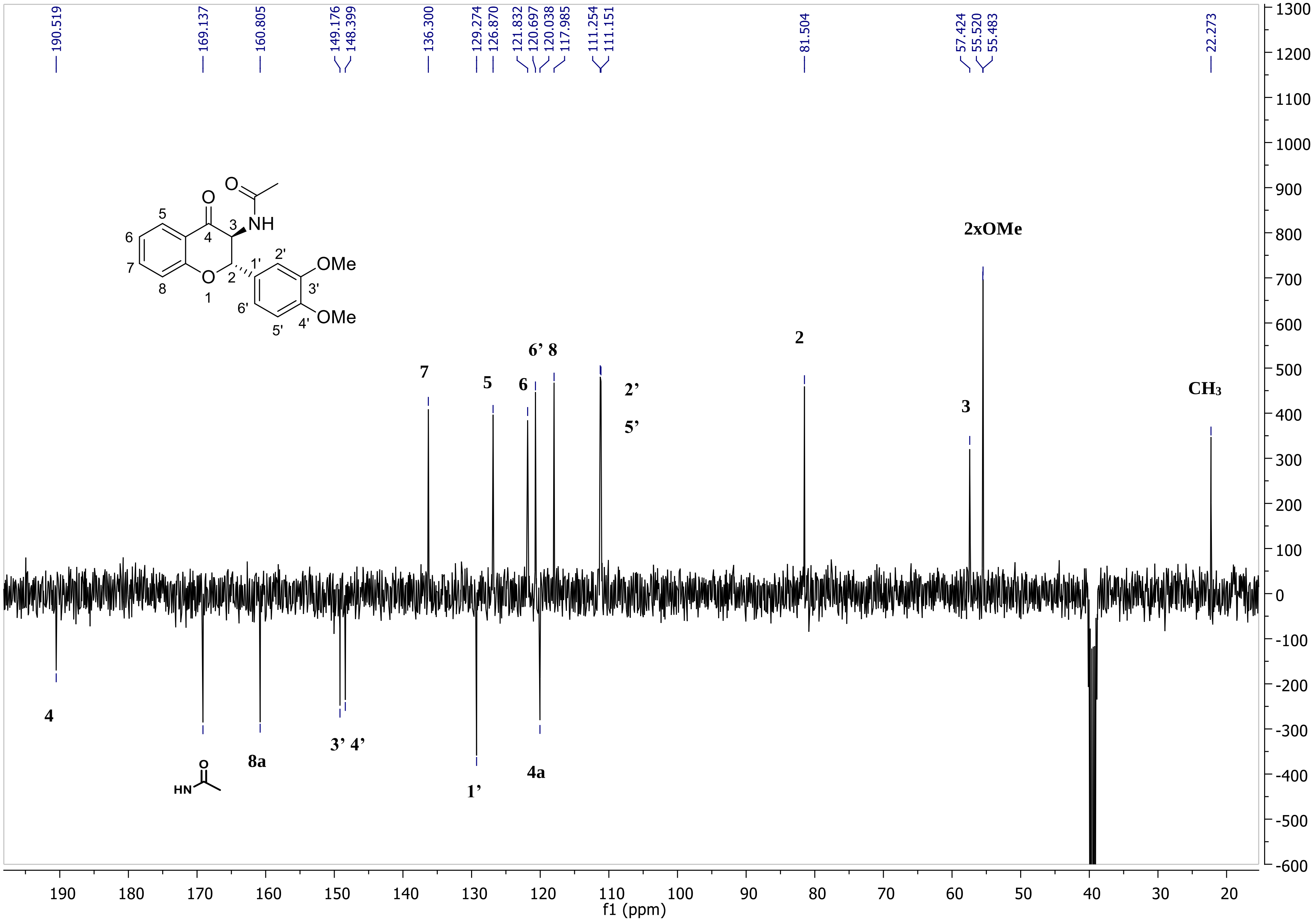

Figure S208. ^{13}C-NMR spectrum of *rac*-*trans*-**24c** in DMSO-d_6

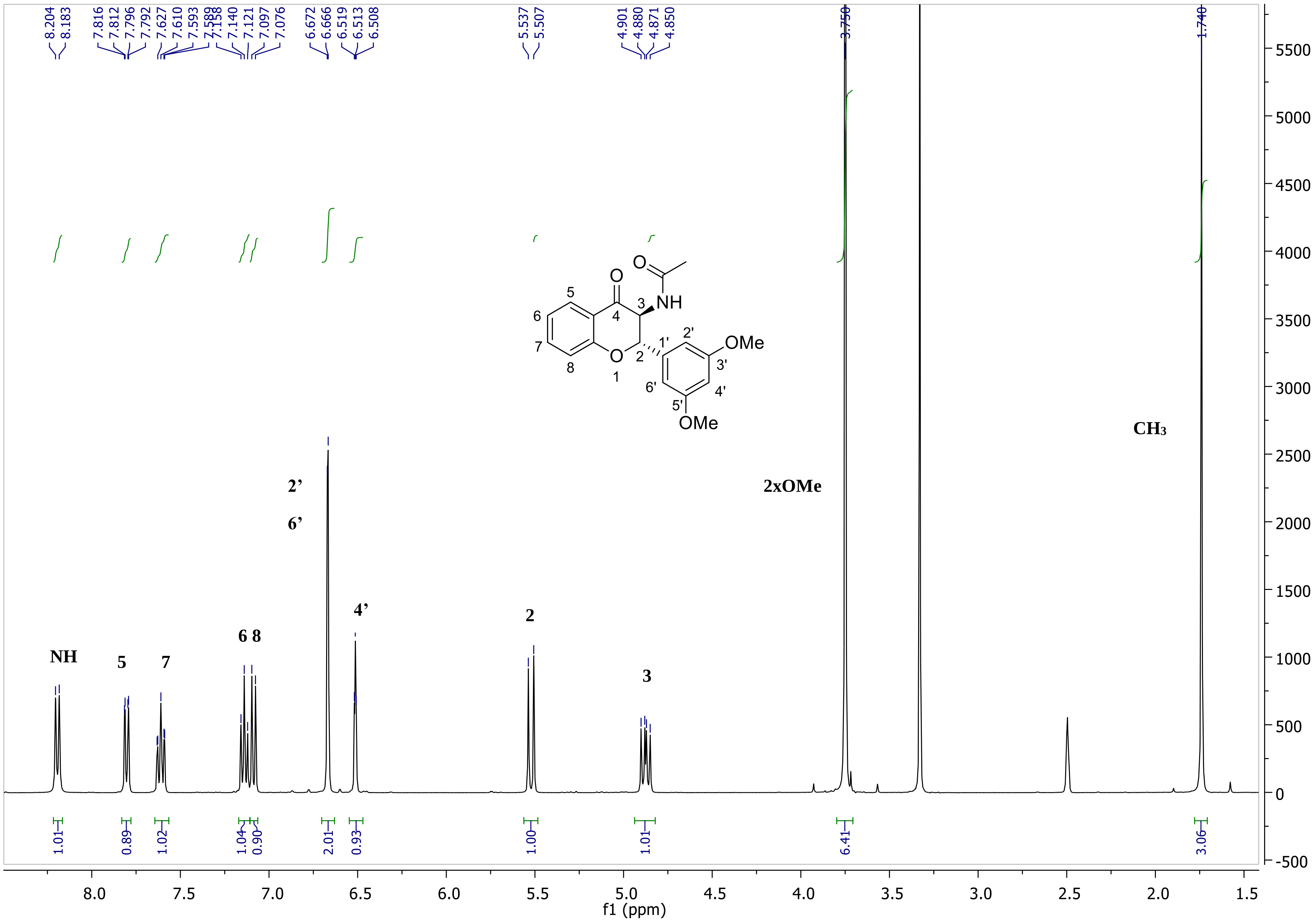

Figure S209. ^{1}H-NMR spectrum of *rac-trans*-**24d** in DMSO-d_6

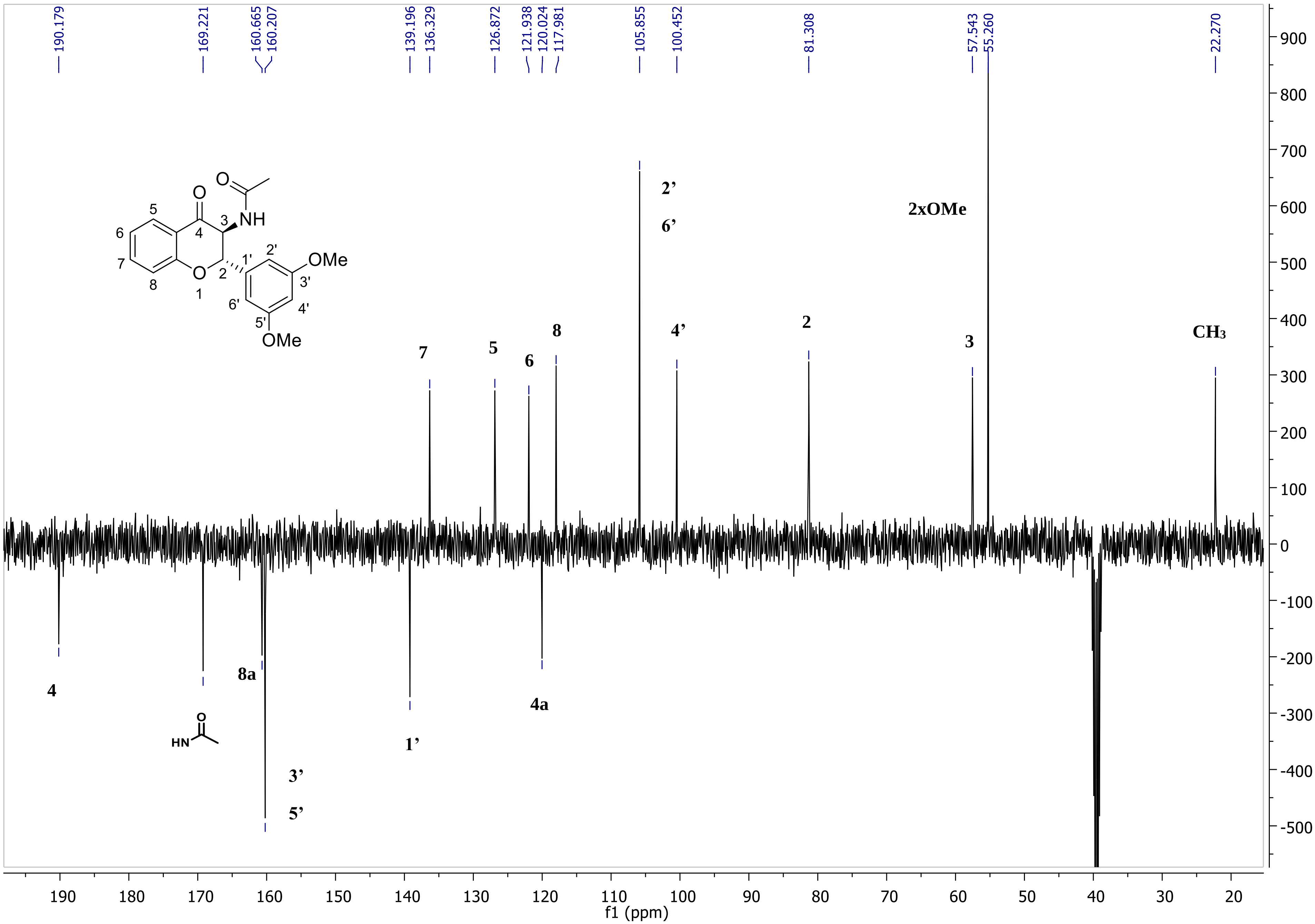

Figure S210. ^{13}C-NMR spectrum of *rac-trans*-**24d** in DMSO-d$_6$

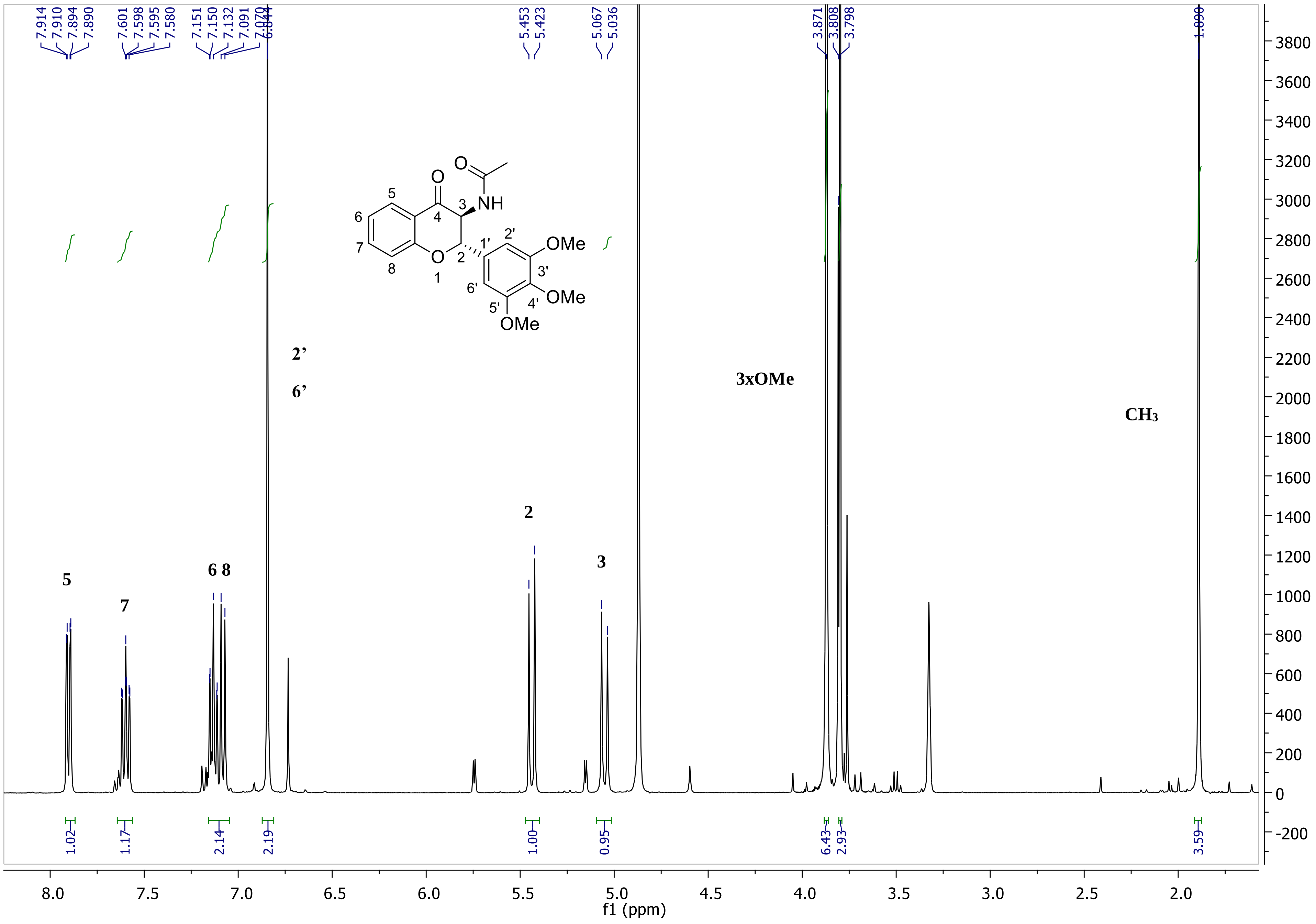

Figure S211. ^{1}H-NMR spectrum of *rac-trans*-**24e** in CD_3OD

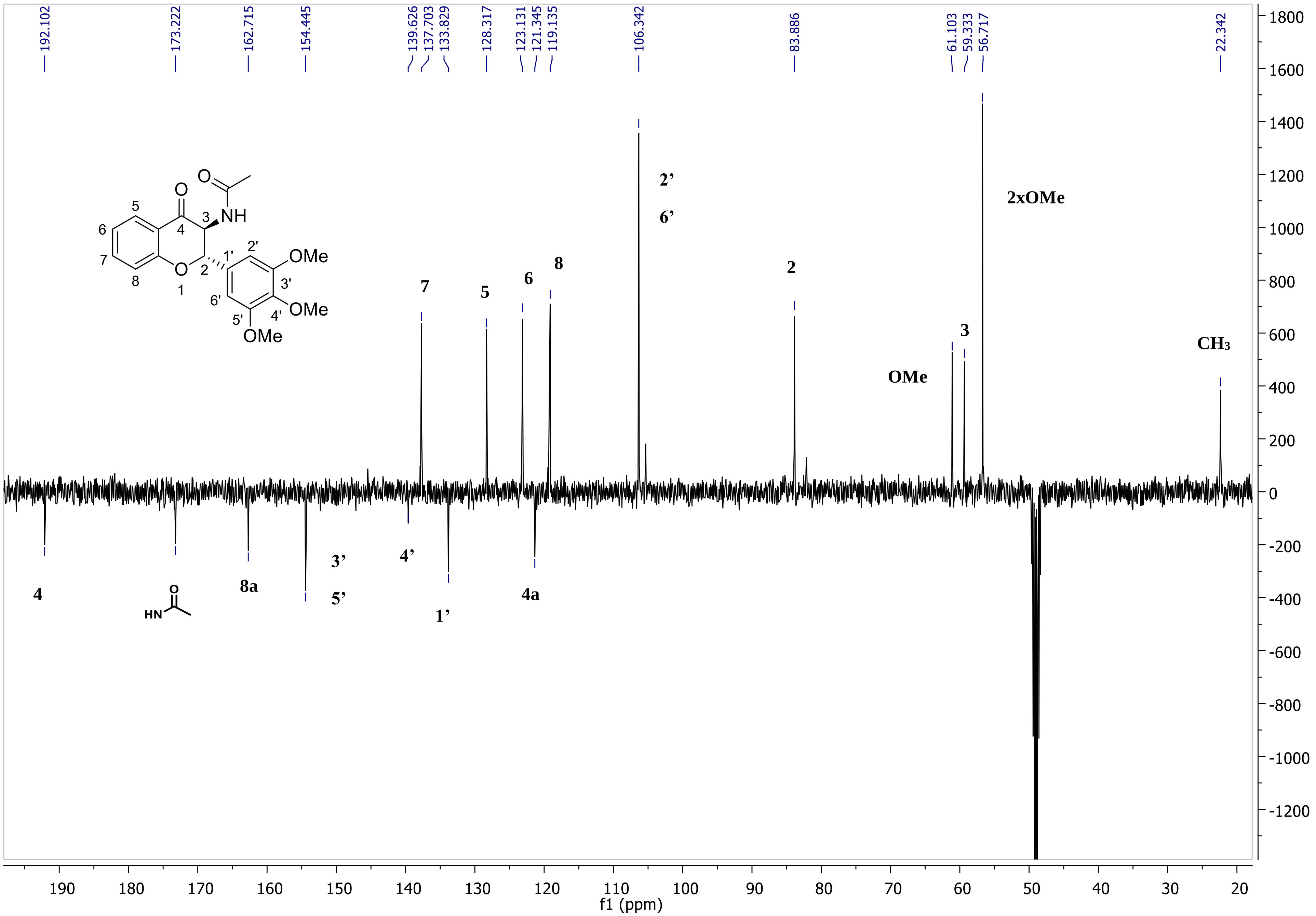

Figure S212. ^{13}C-NMR spectrum of *rac*-*trans*-**24e** in CD_3OD

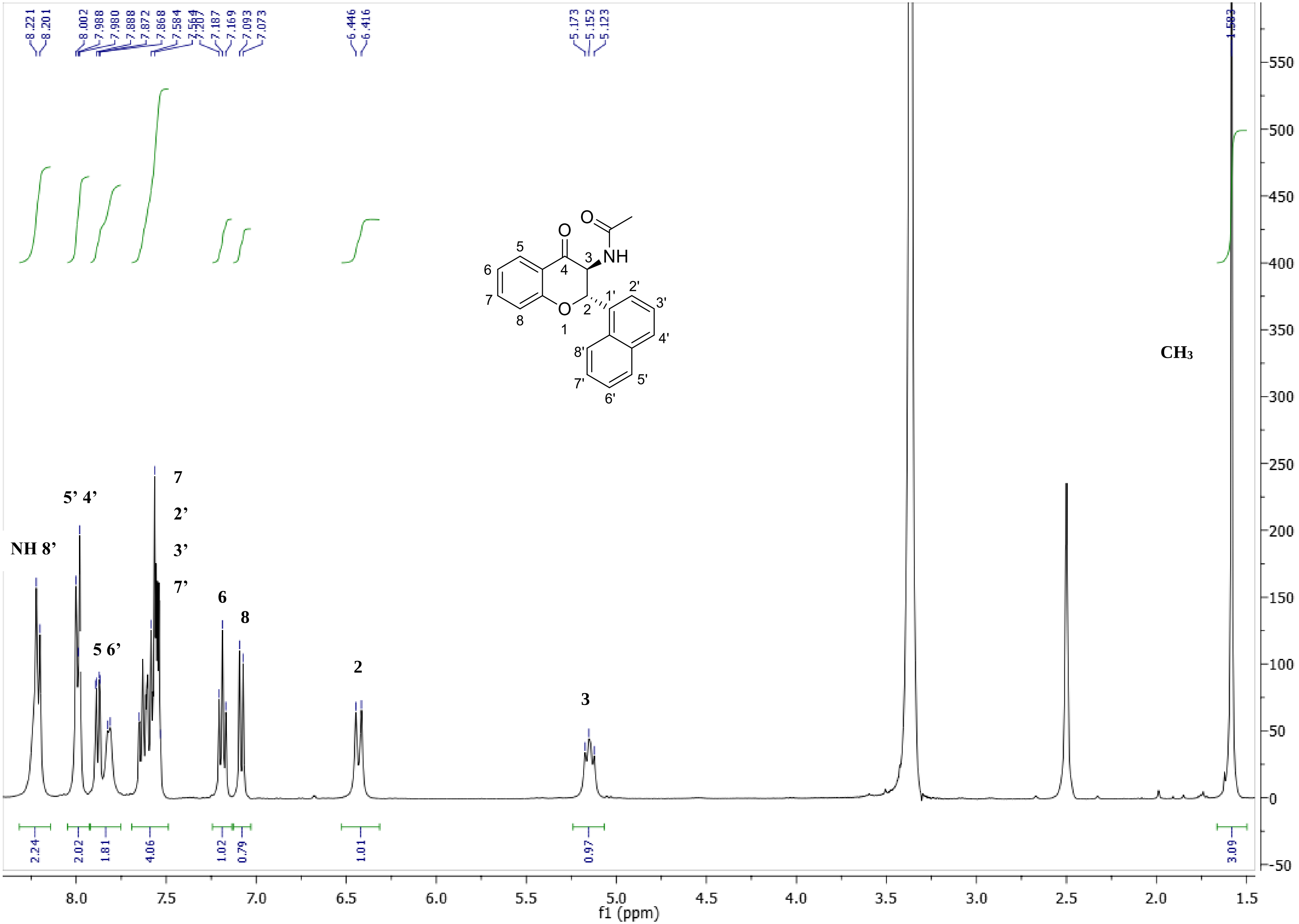

Figure S213. ^{1}H-NMR spectrum of *rac-trans*-**24f** in DMSO-d$_6$

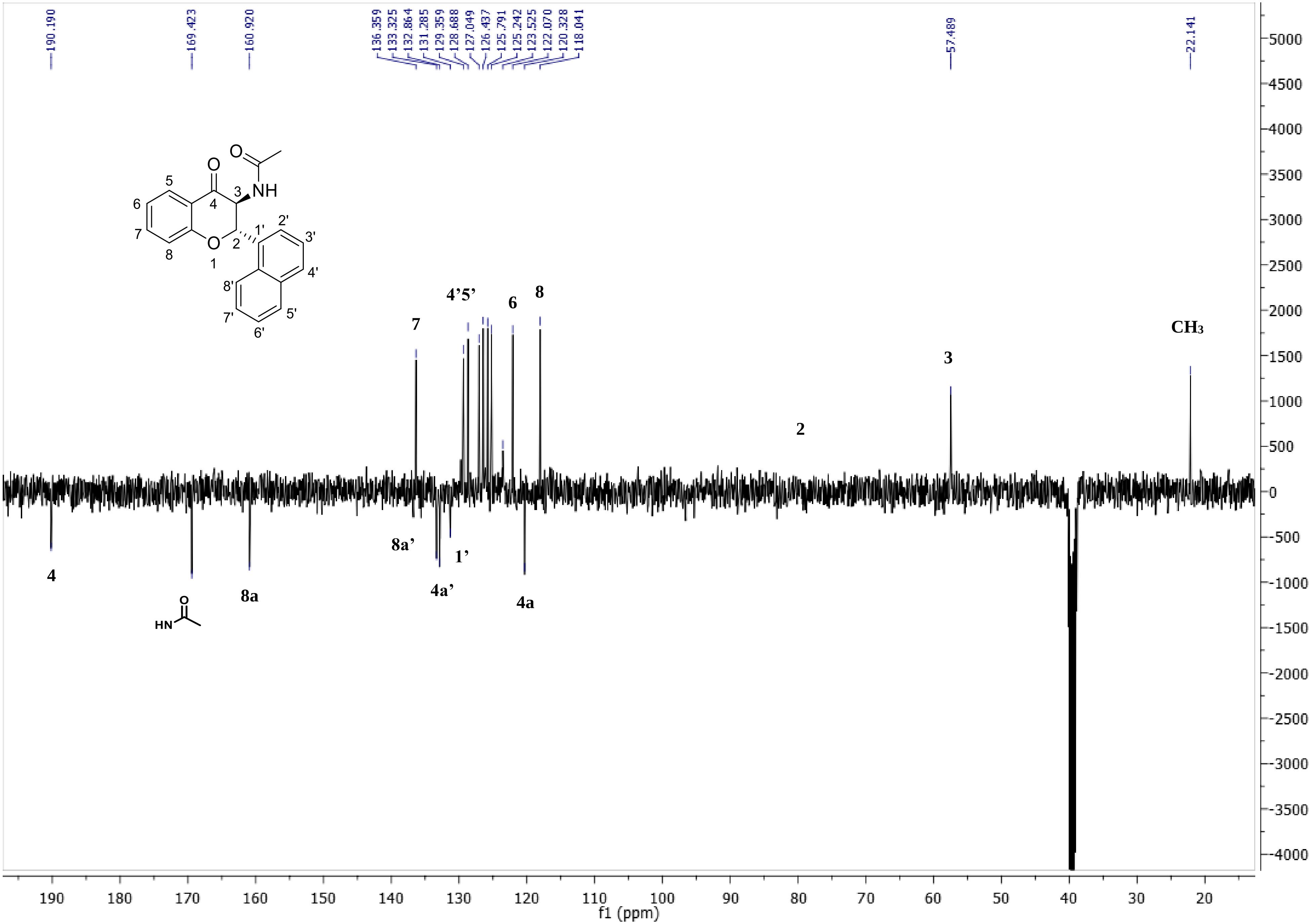

Figure S214. ^{13}C-NMR spectrum of *rac*-*trans*-**24f** in DMSO-d_6

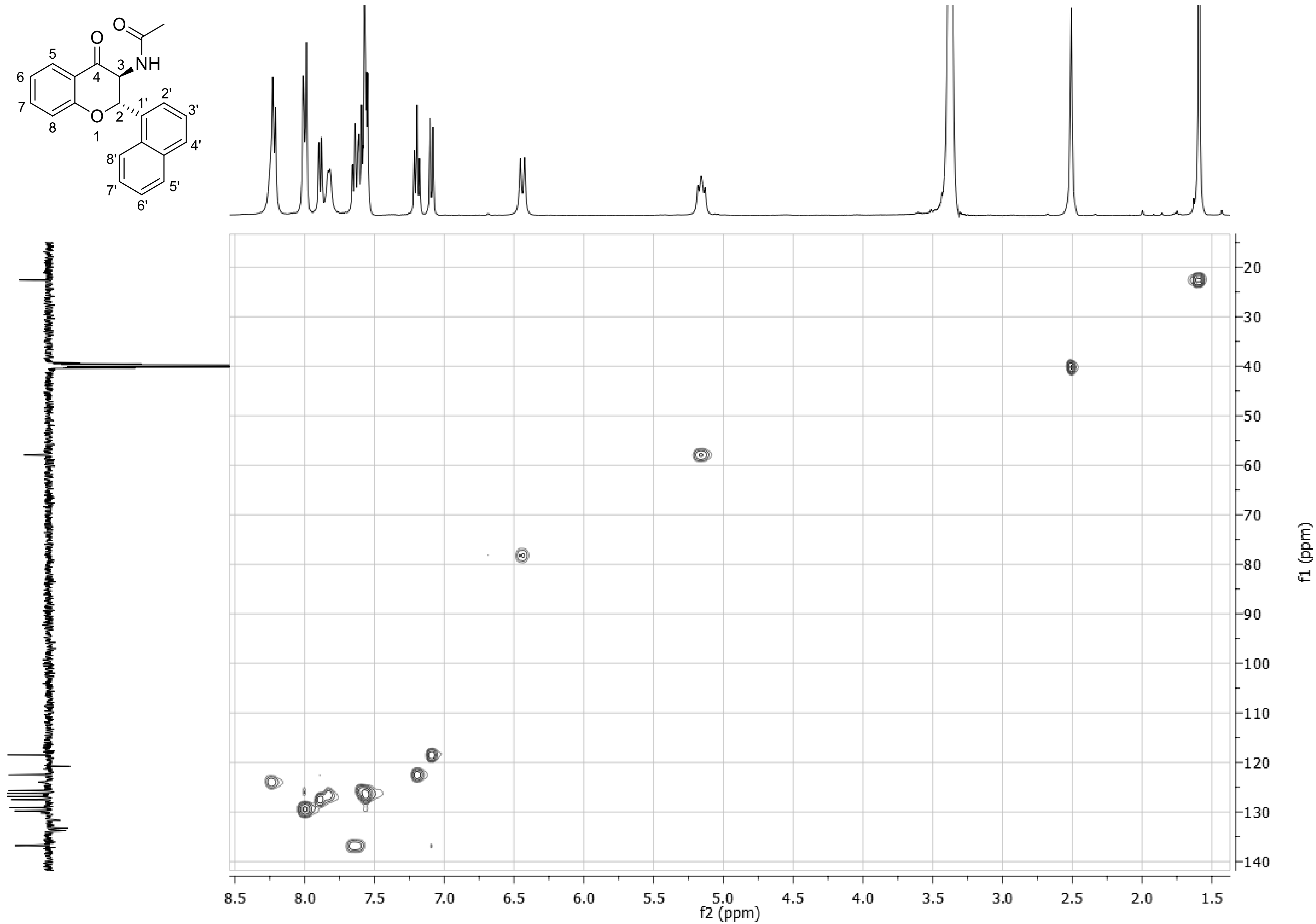

Figure S215. HSQC spectrum of *rac-trans*-**24f** in DMSO-d$_6$

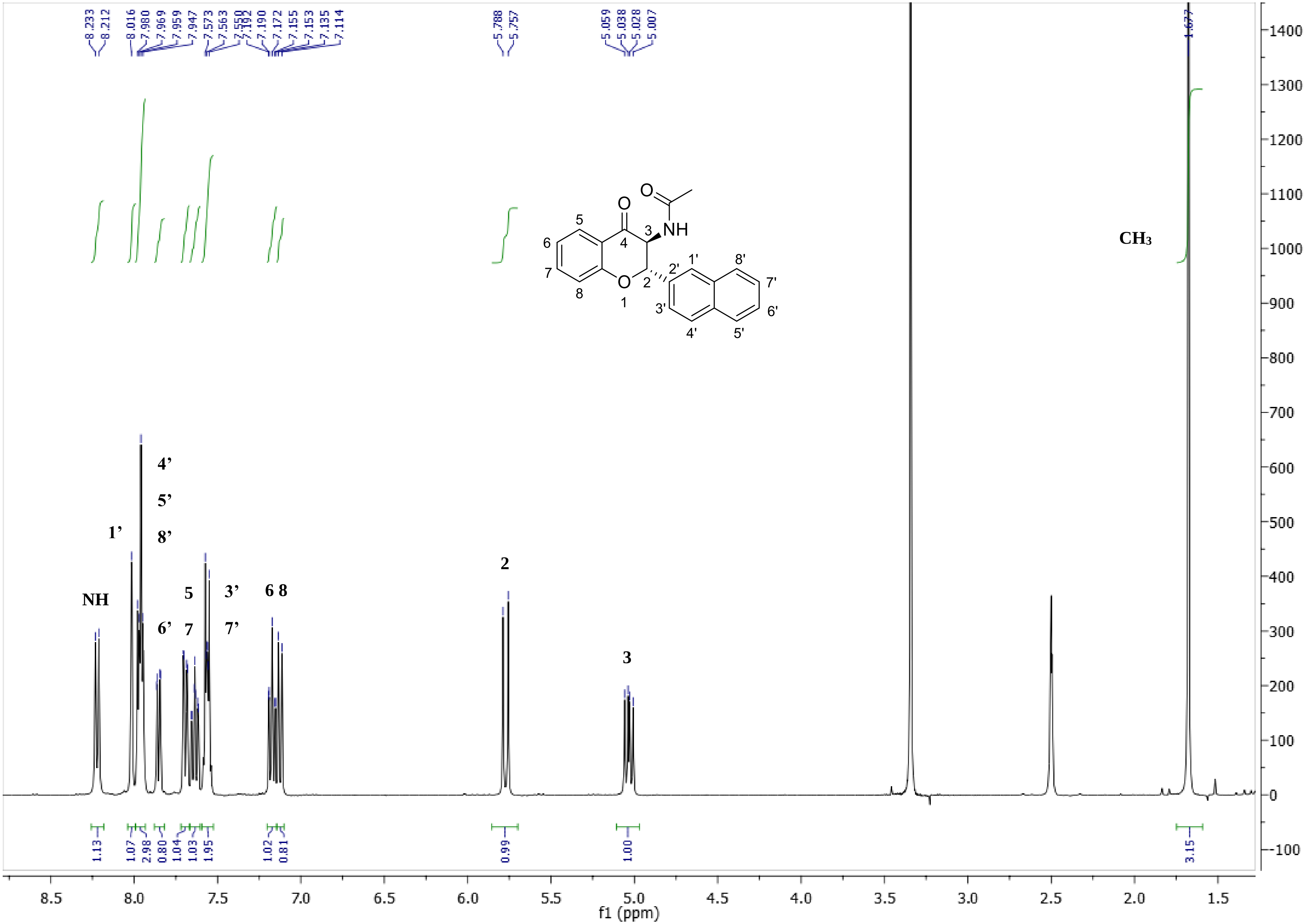

Figure S216. ^{1}H-NMR spectrum of *rac*-*trans*-**24g** in DMSO-d_6

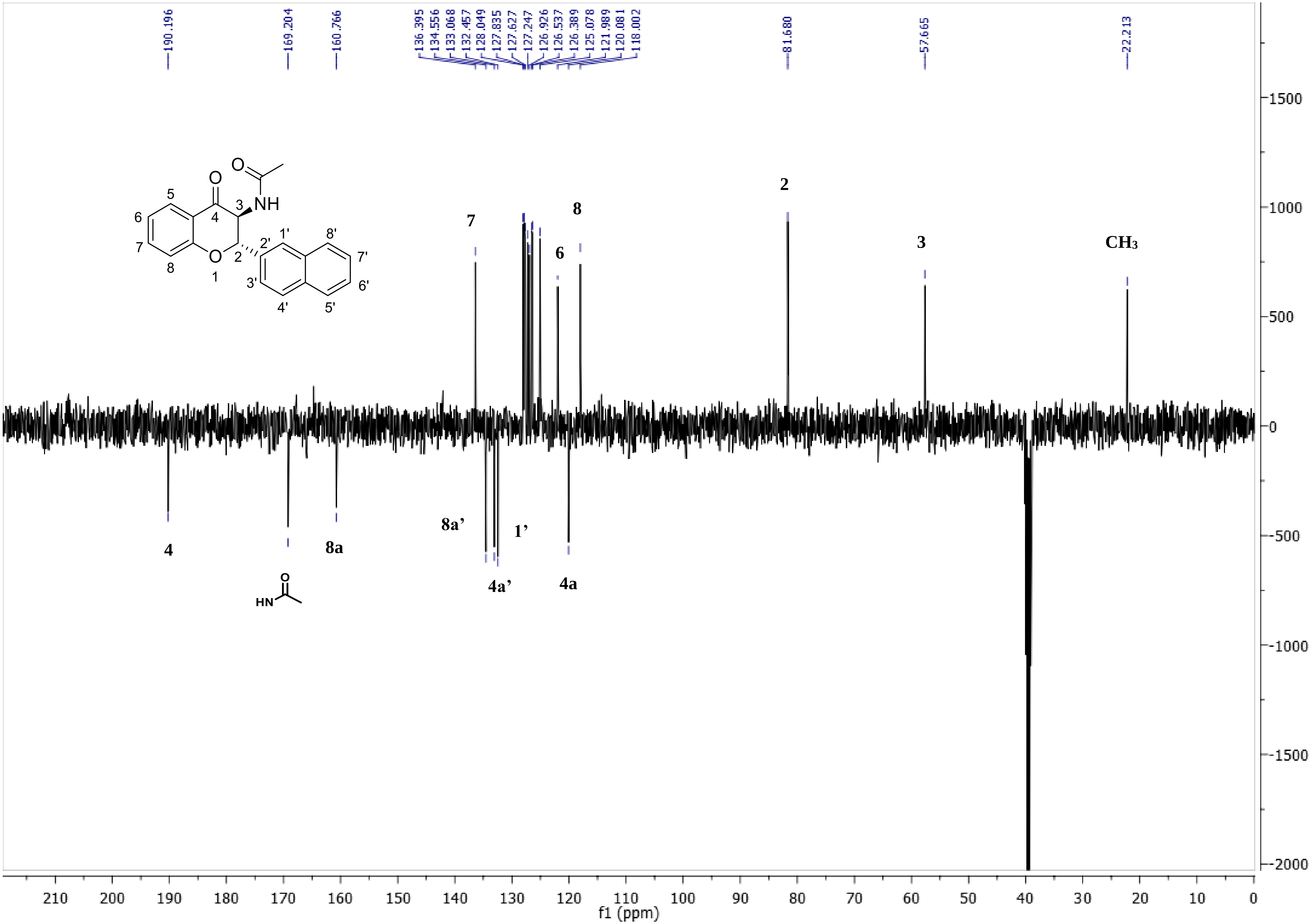

Figure S217. ^{13}C-NMR spectrum of *rac-trans*-**24g** in DMSO-d$_6$

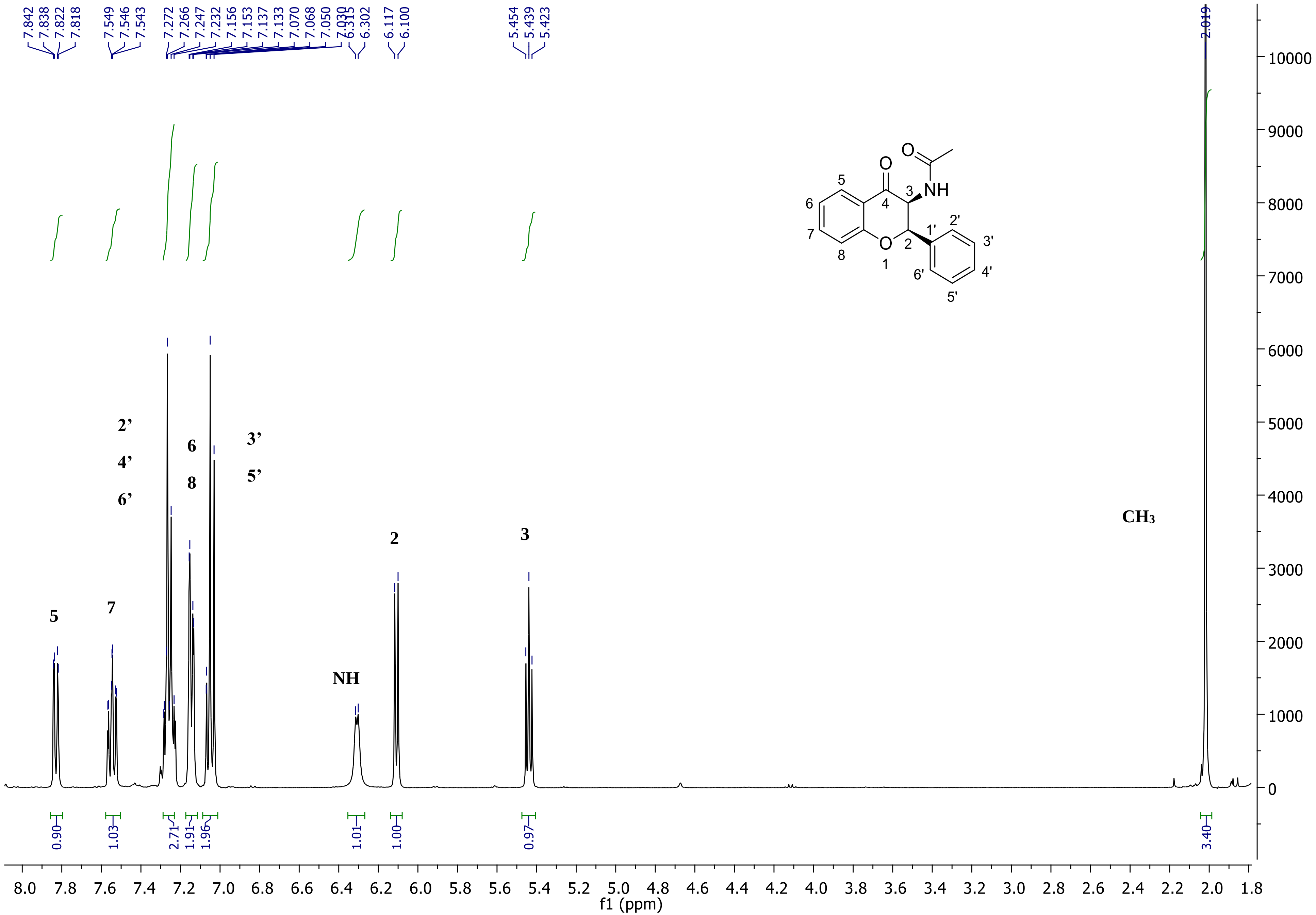

Figure S218. ^{1}H-NMR spectrum of *rac-cis*-**24a** in $CDCl_3$

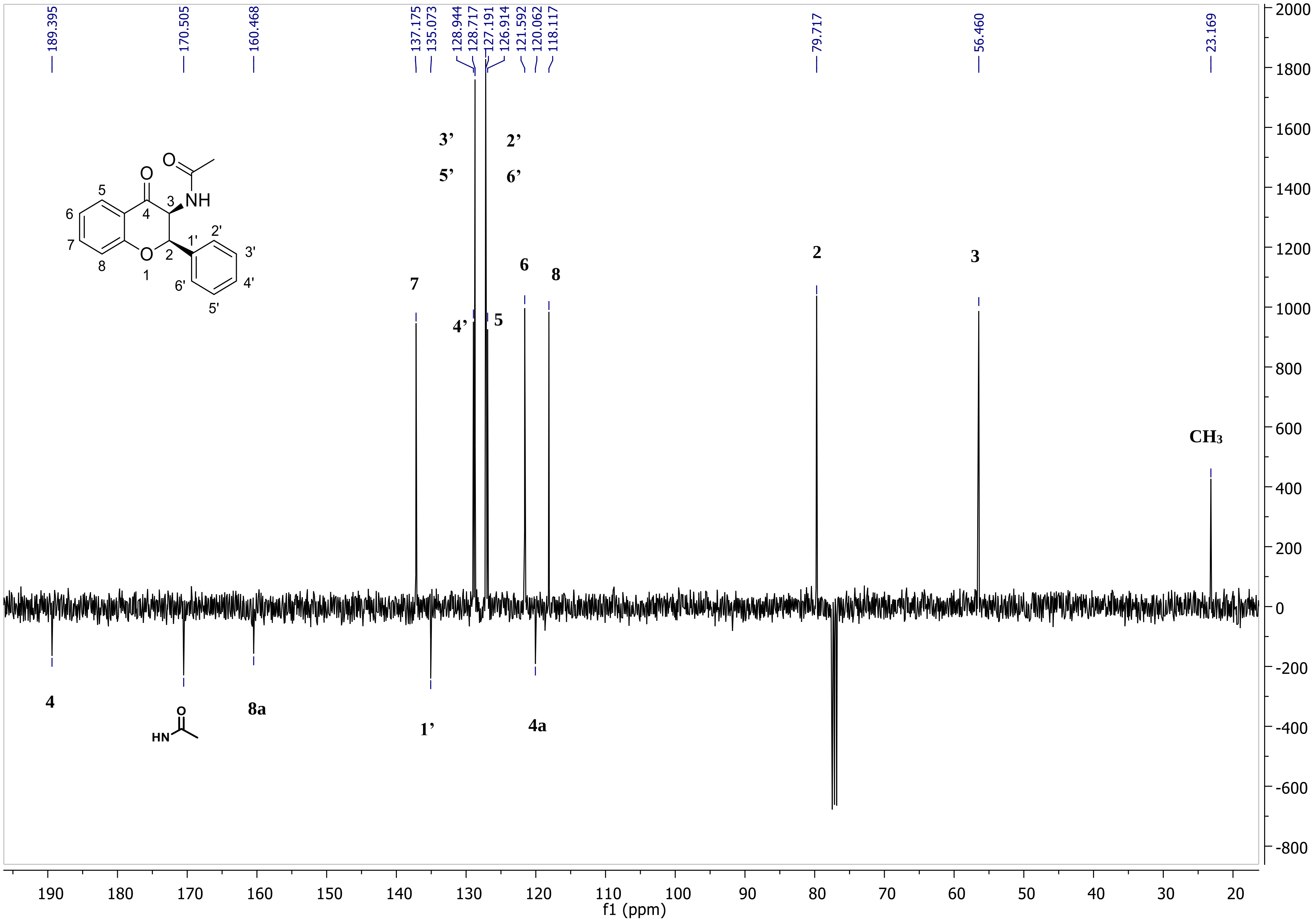

Figure S219. ^{13}C-NMR spectrum of *rac*-*cis*-**24a** in $CDCl_3$

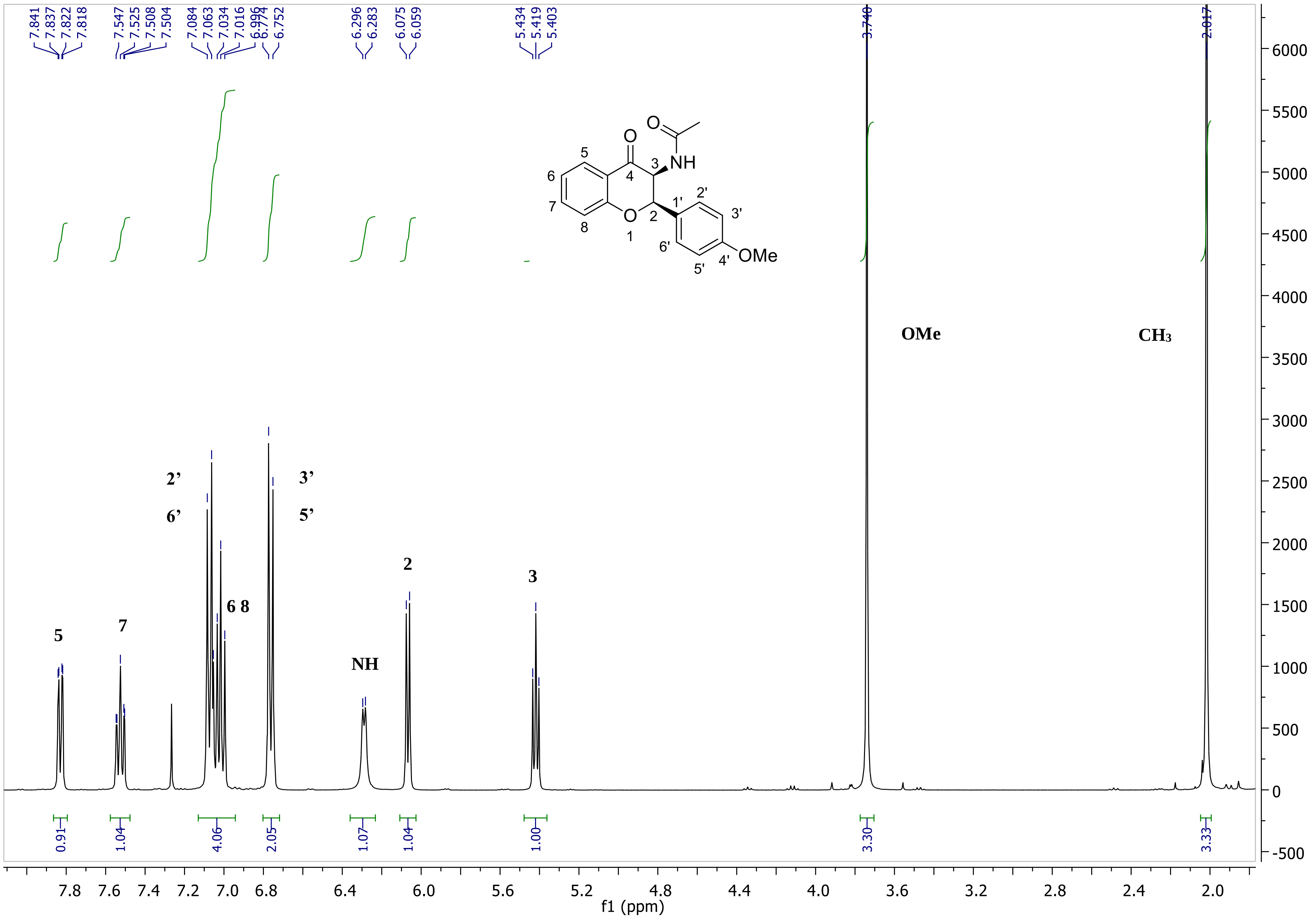

Figure S220. ^{1}H-NMR spectrum of *rac*-*cis*-**24b** in $CDCl_3$

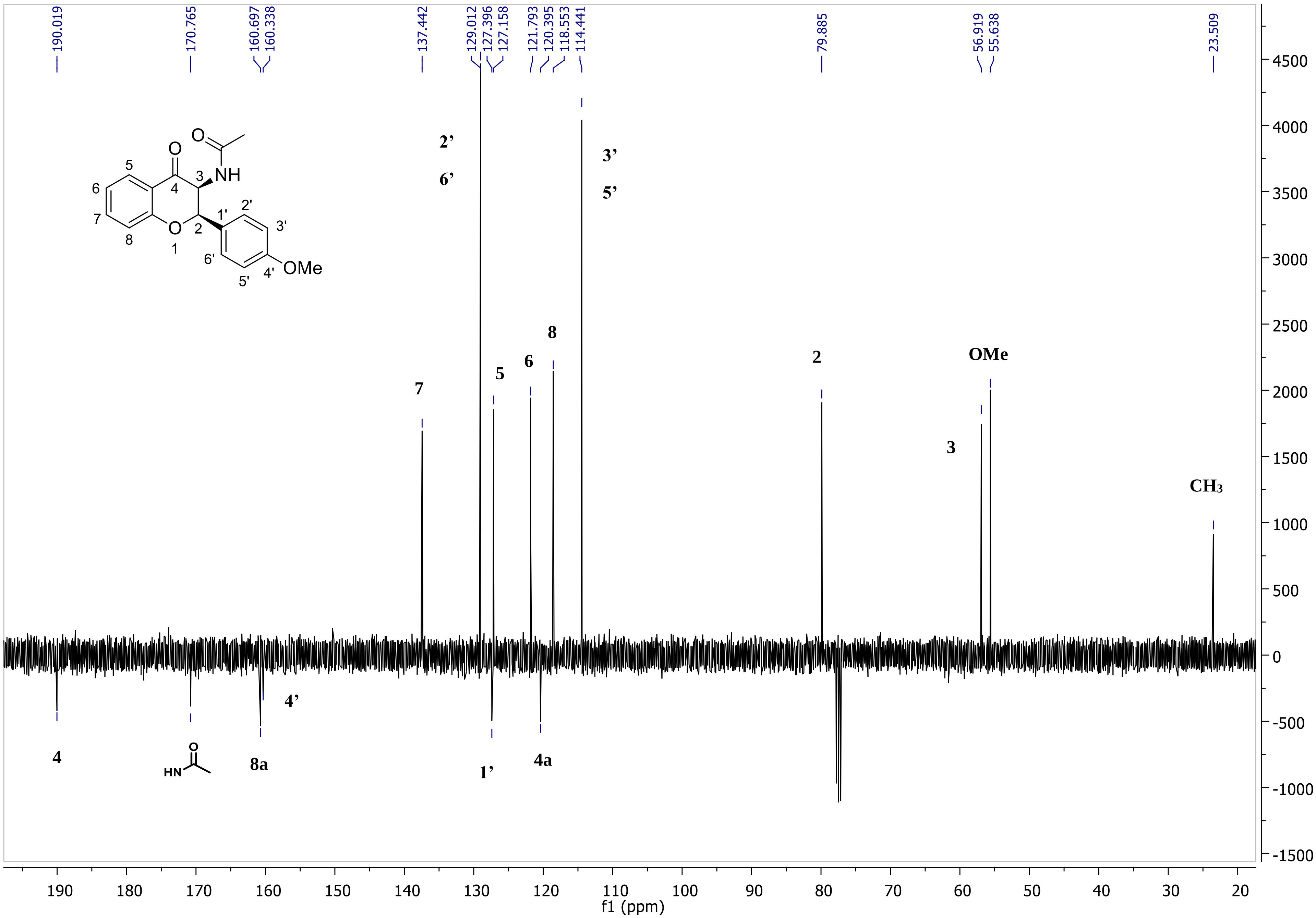

Figure S221. ^{13}C-NMR spectrum of *rac*-*cis*-**24b** in $CDCl_3$

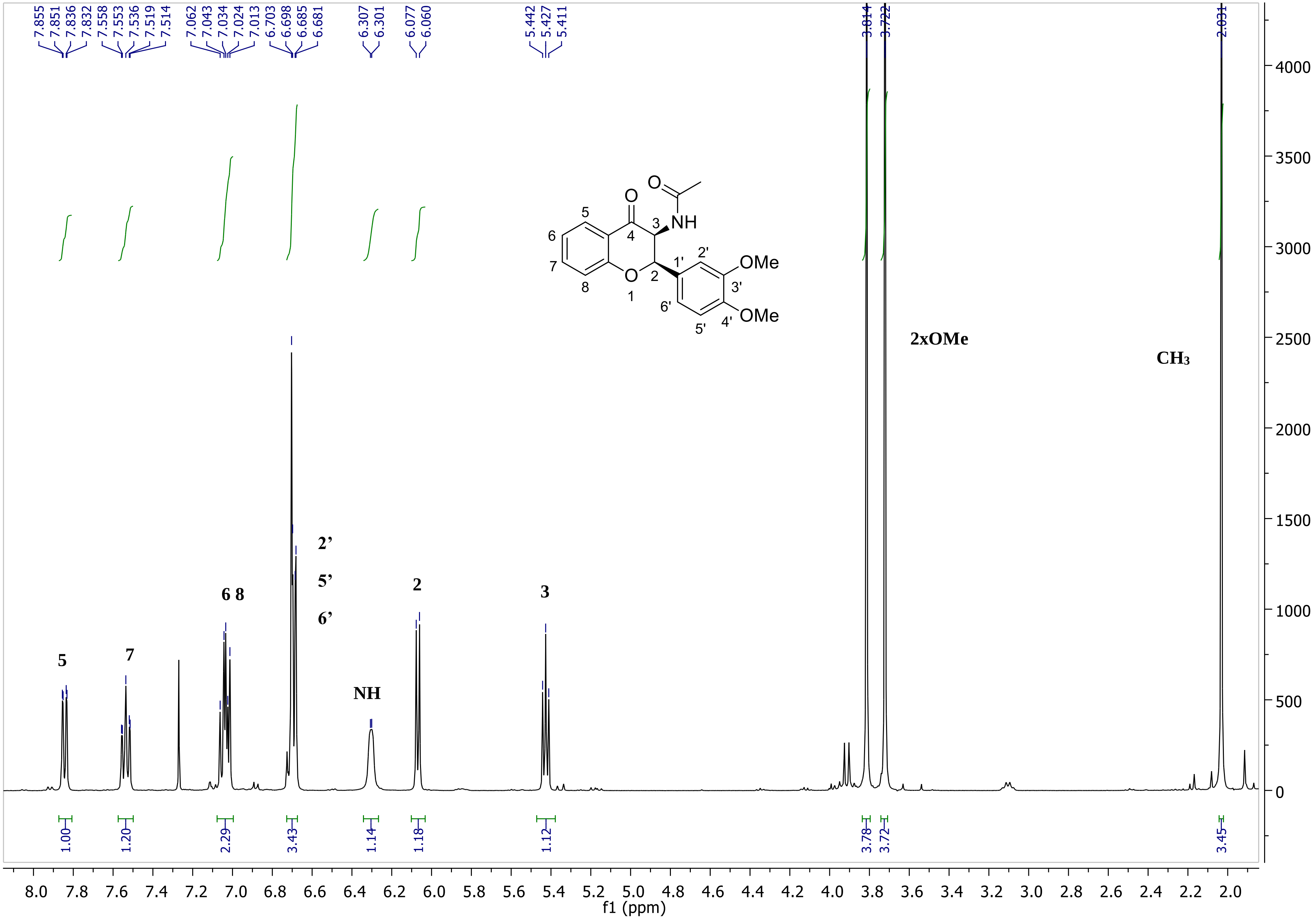

Figure S222. ^{1}H-NMR spectrum of *rac*-*cis*-**24c** in $CDCl_3$

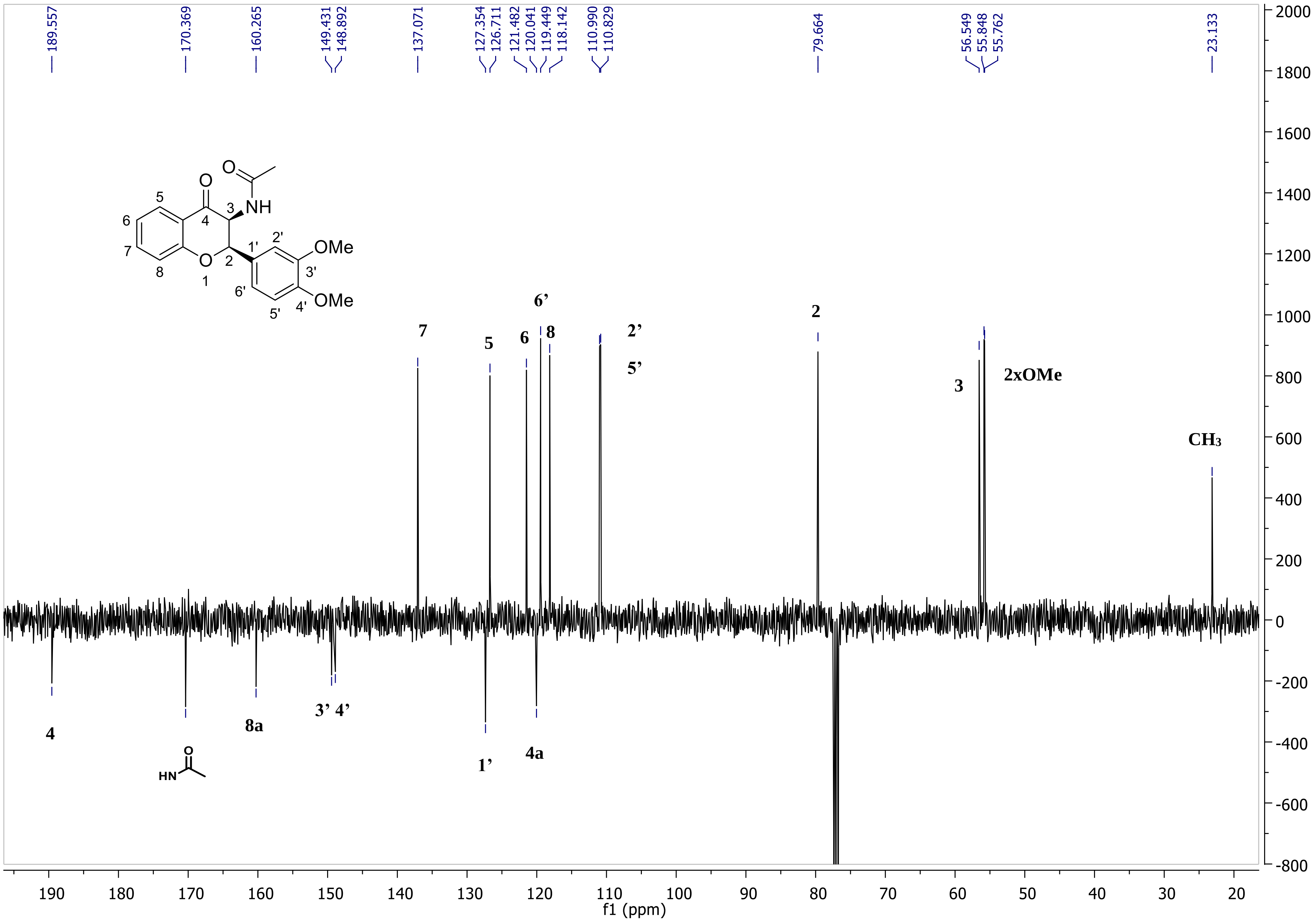

Figure S223. ^{13}C-NMR spectrum of *rac-cis*-**24c** in $CDCl_3$

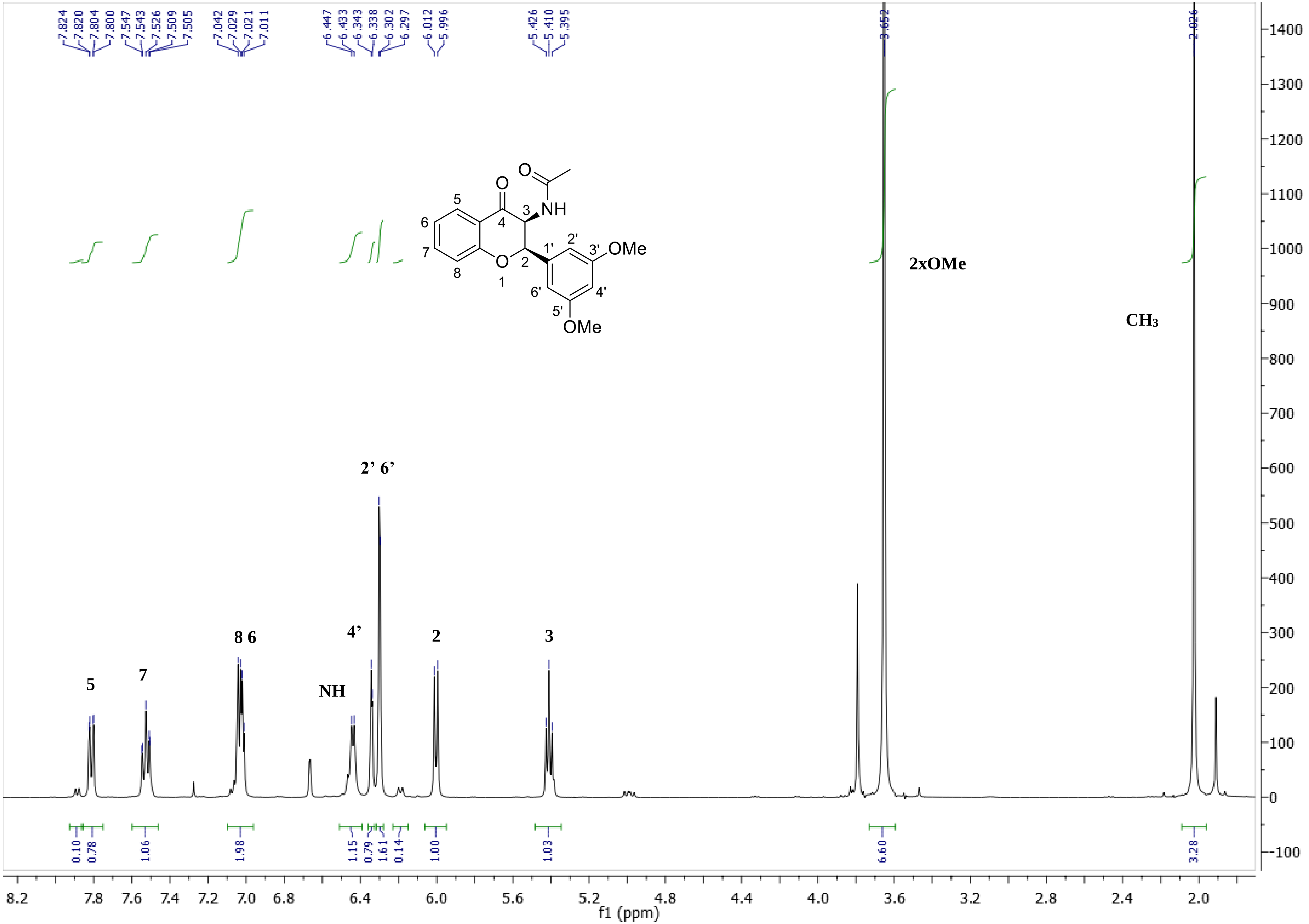

Figure S224. ^{1}H-NMR spectrum of *rac-cis*-**24d** in $CDCl_3$

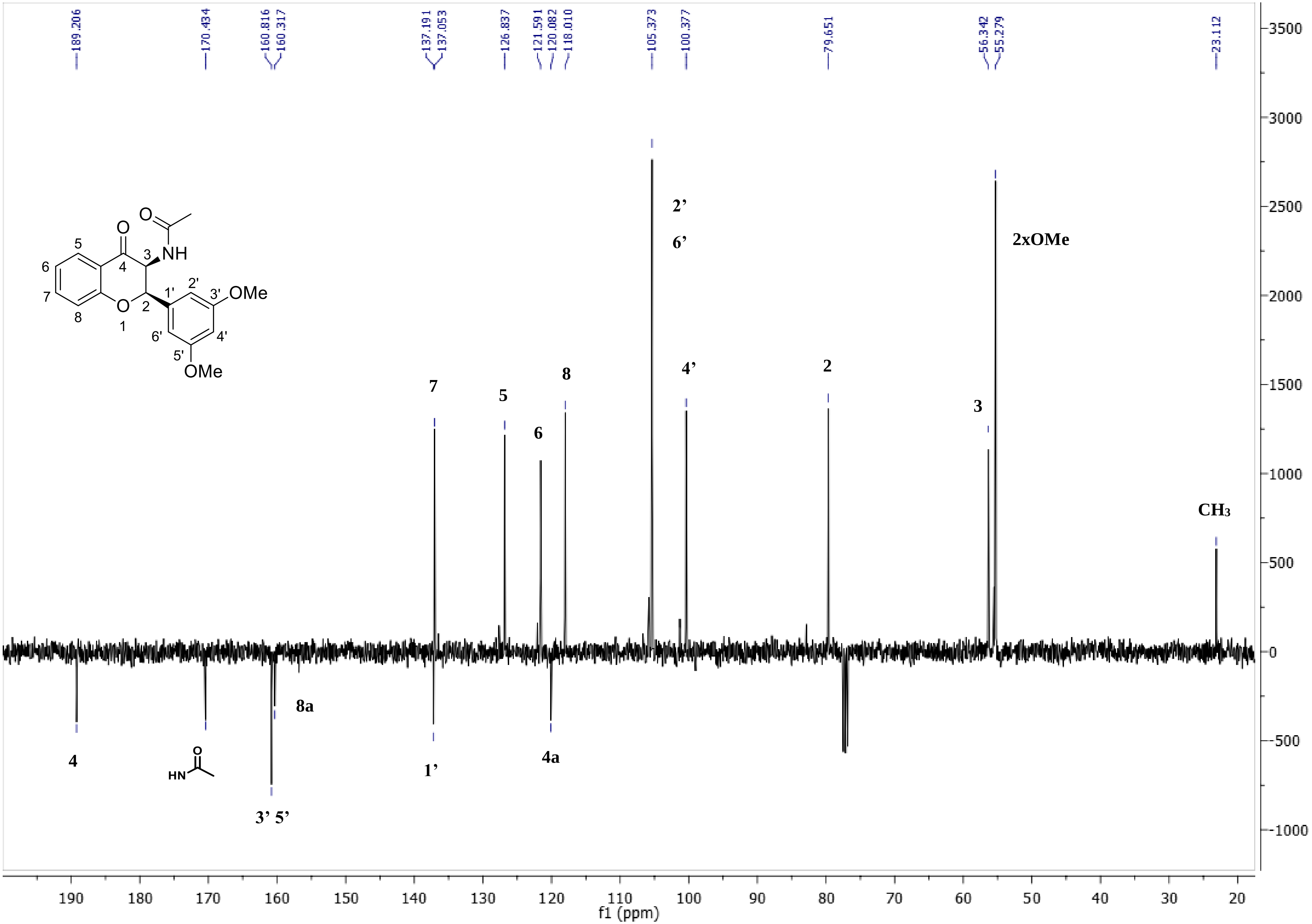

Figure S225. ^{13}C-NMR spectrum of *rac*-*cis*-**24d** in $CDCl_3$

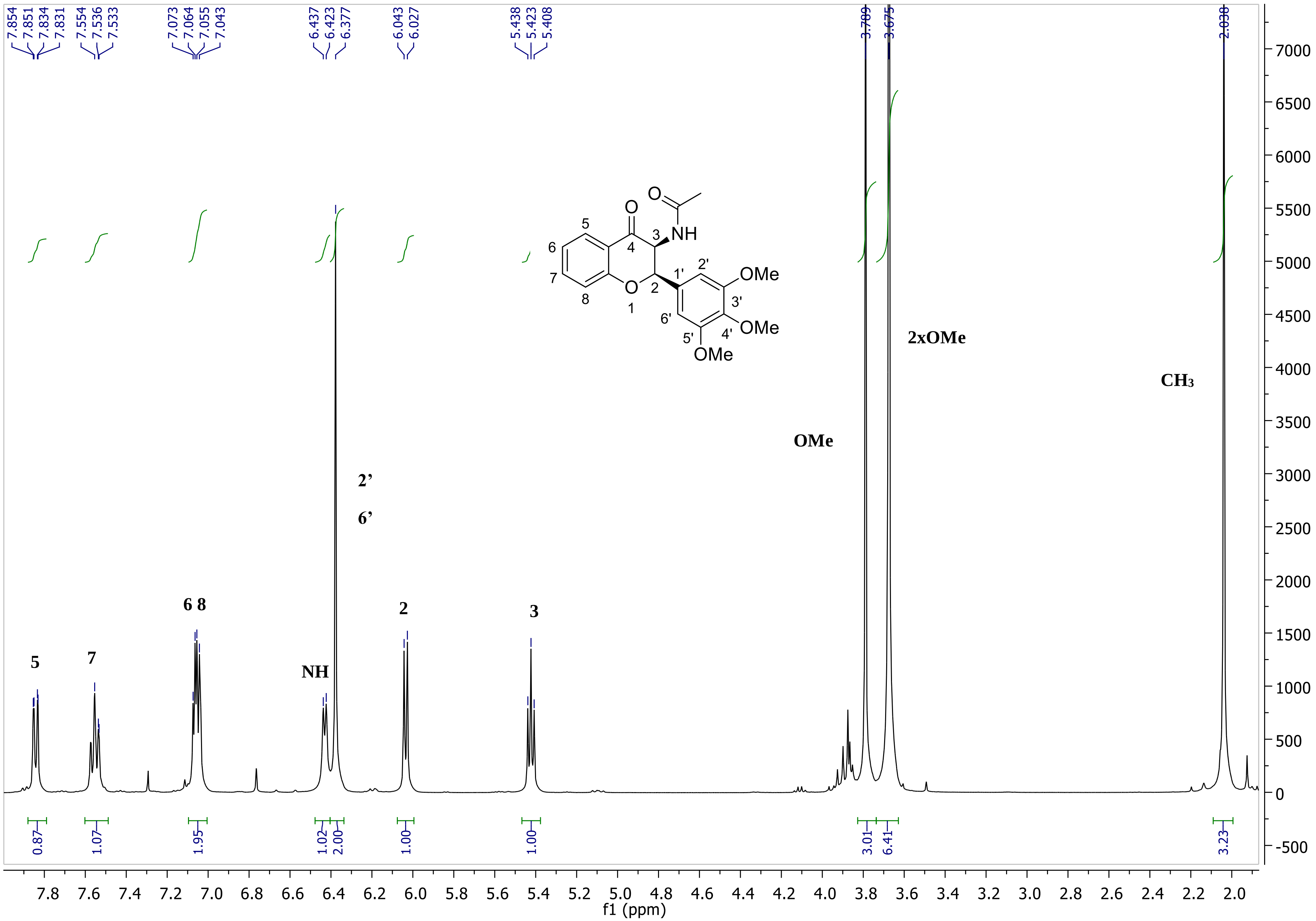

Figure S226. ^{1}H-NMR spectrum of *rac*-*cis*-**24e** in $CDCl_3$

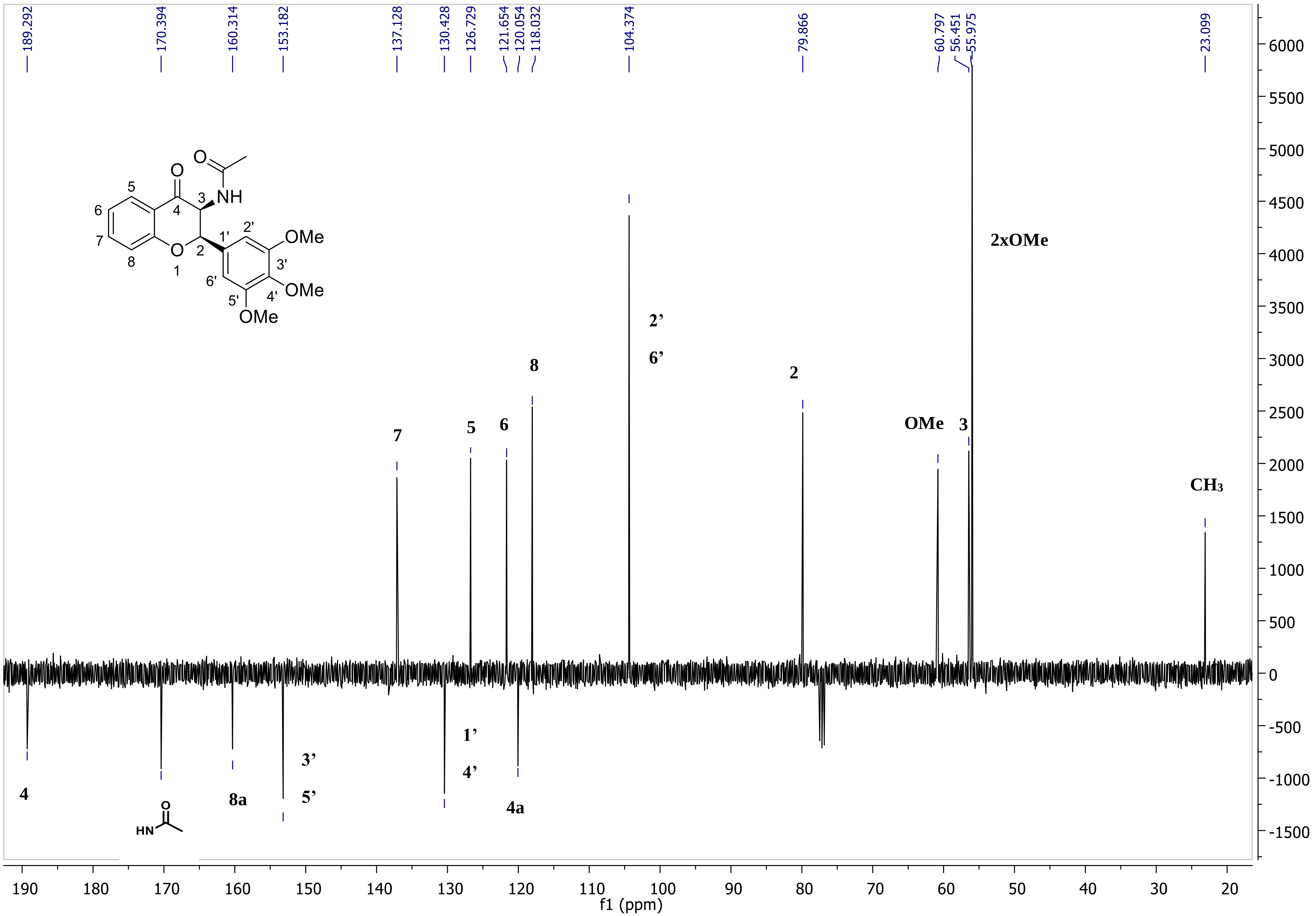

Figure S227. ^{13}C-NMR spectrum of *rac-cis*-**24e** in $CDCl_3$

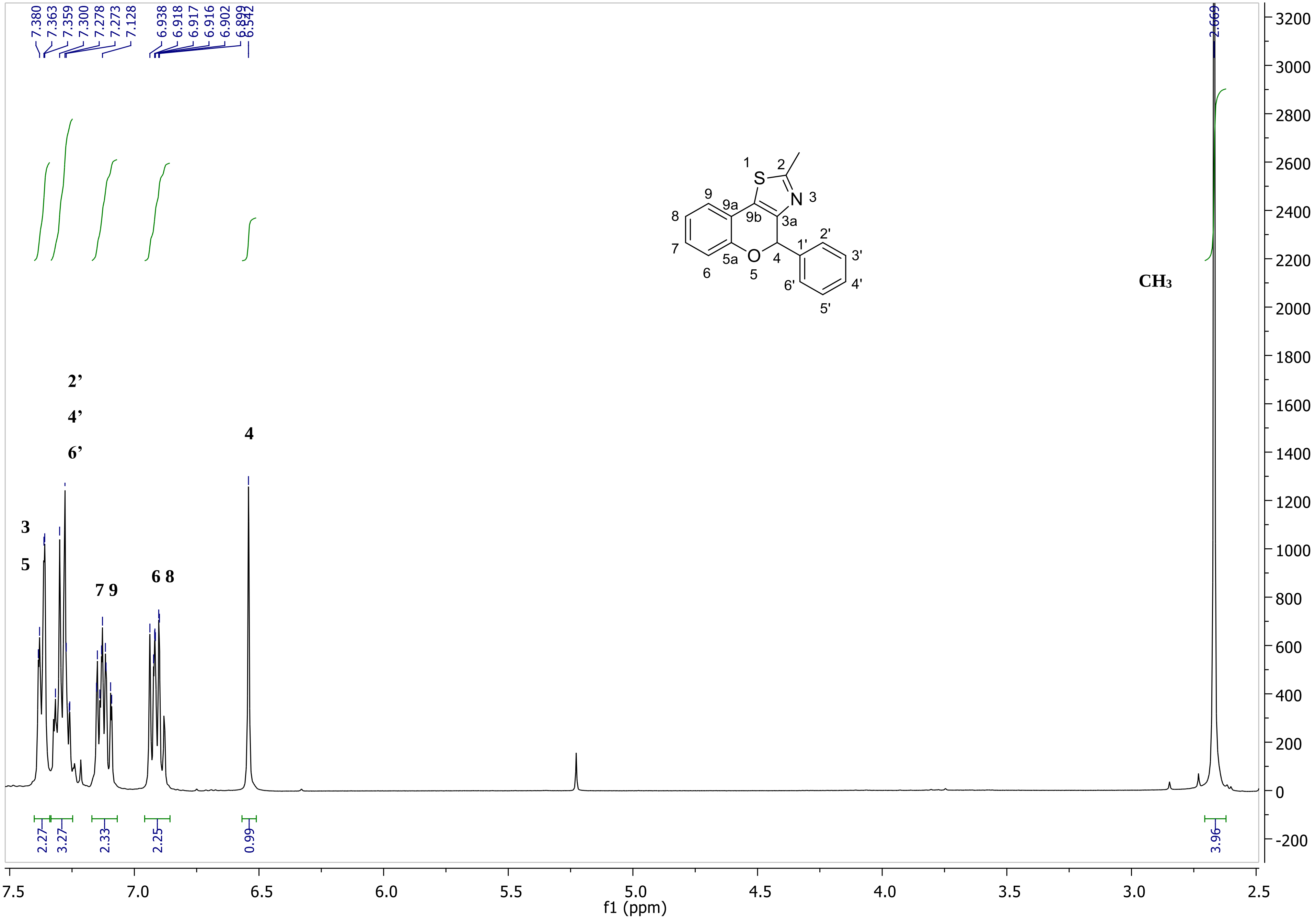

Figure S228. ^{1}H-NMR spectrum of *rac*-**3a** in $CDCl_3$

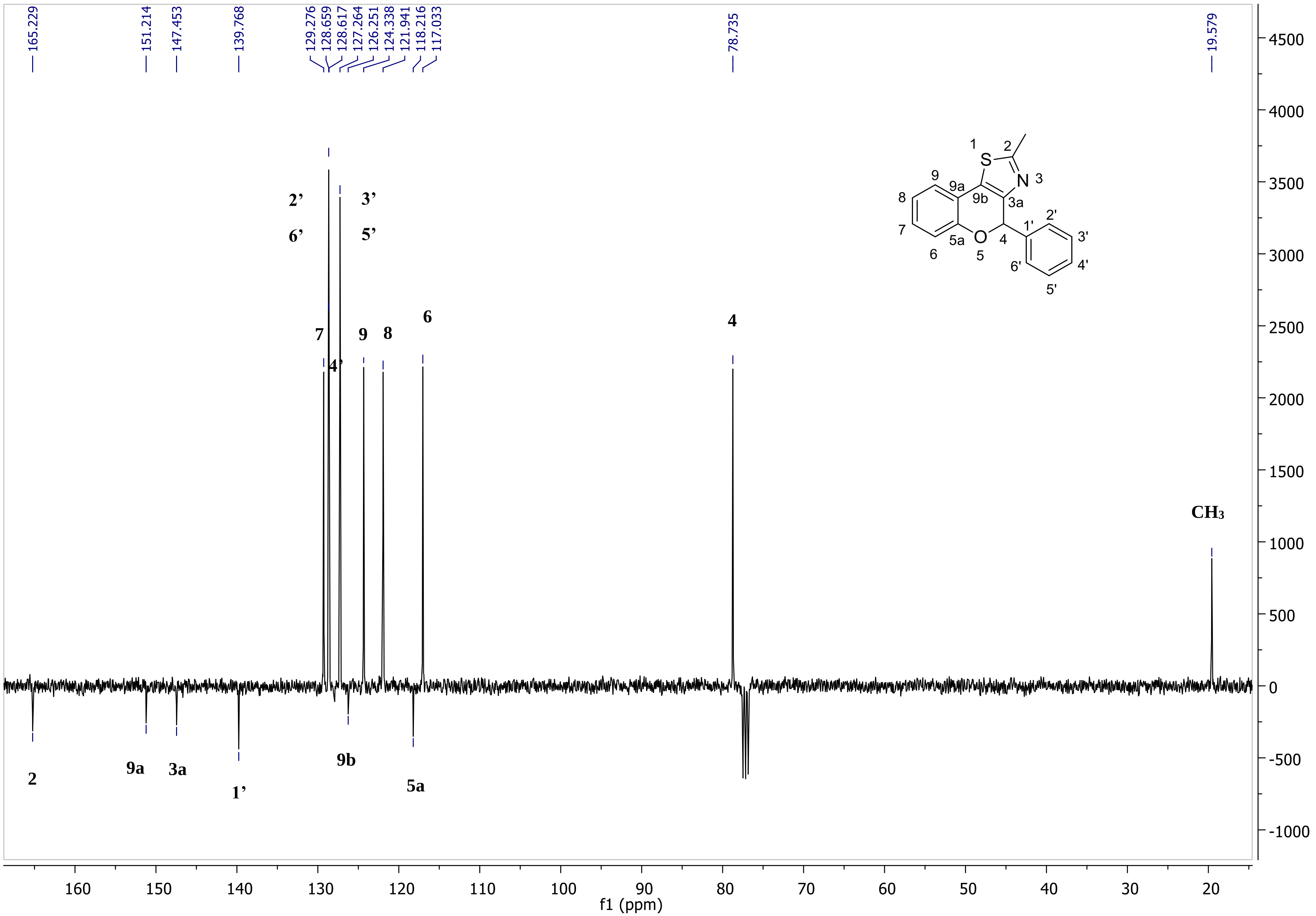

Figure S229. ^{13}C-NMR spectrum of *rac*-**3a** in $CDCl_3$

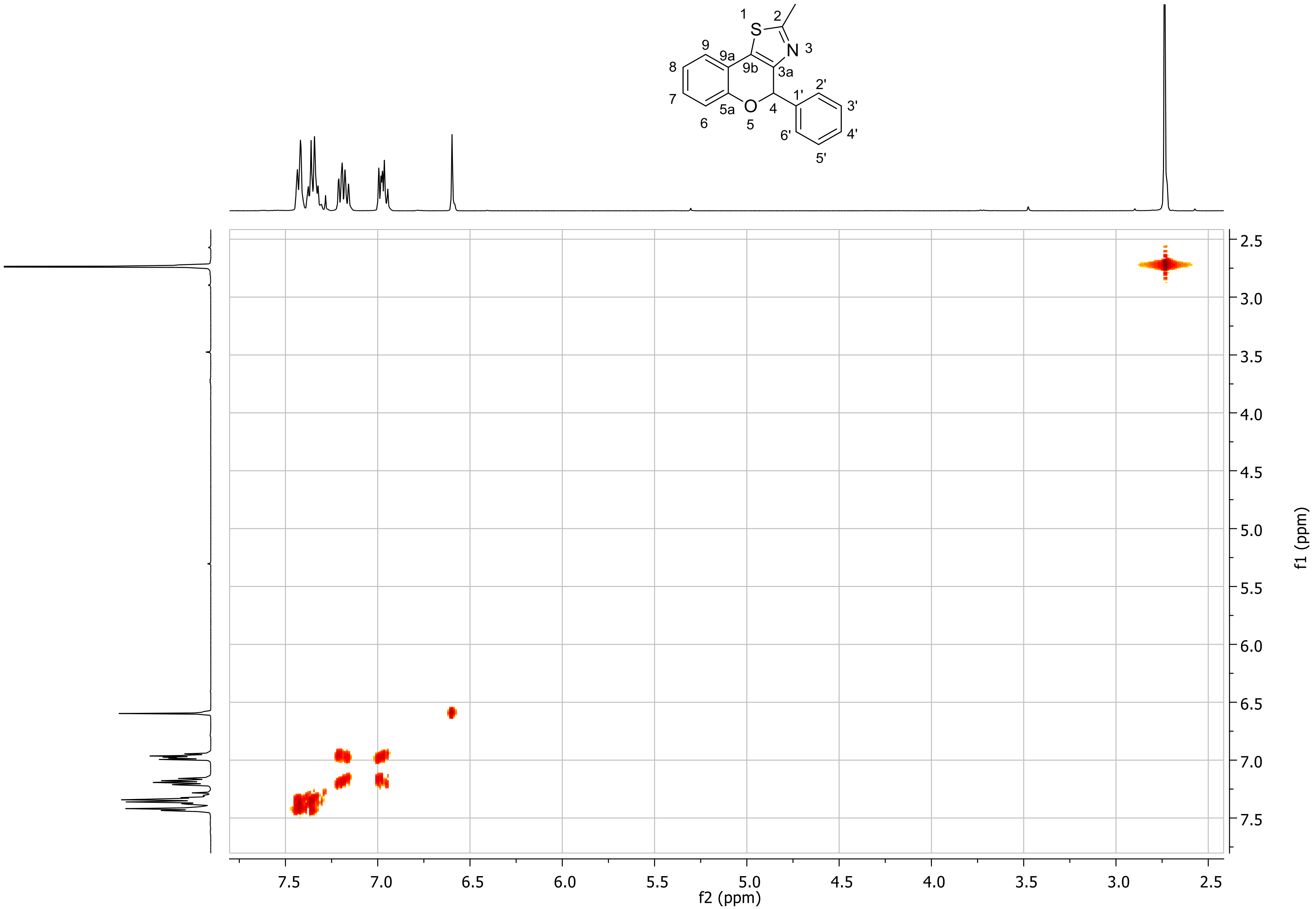

Figure S230. COSY spectrum of *rac*-**3a** in $CDCl_3$

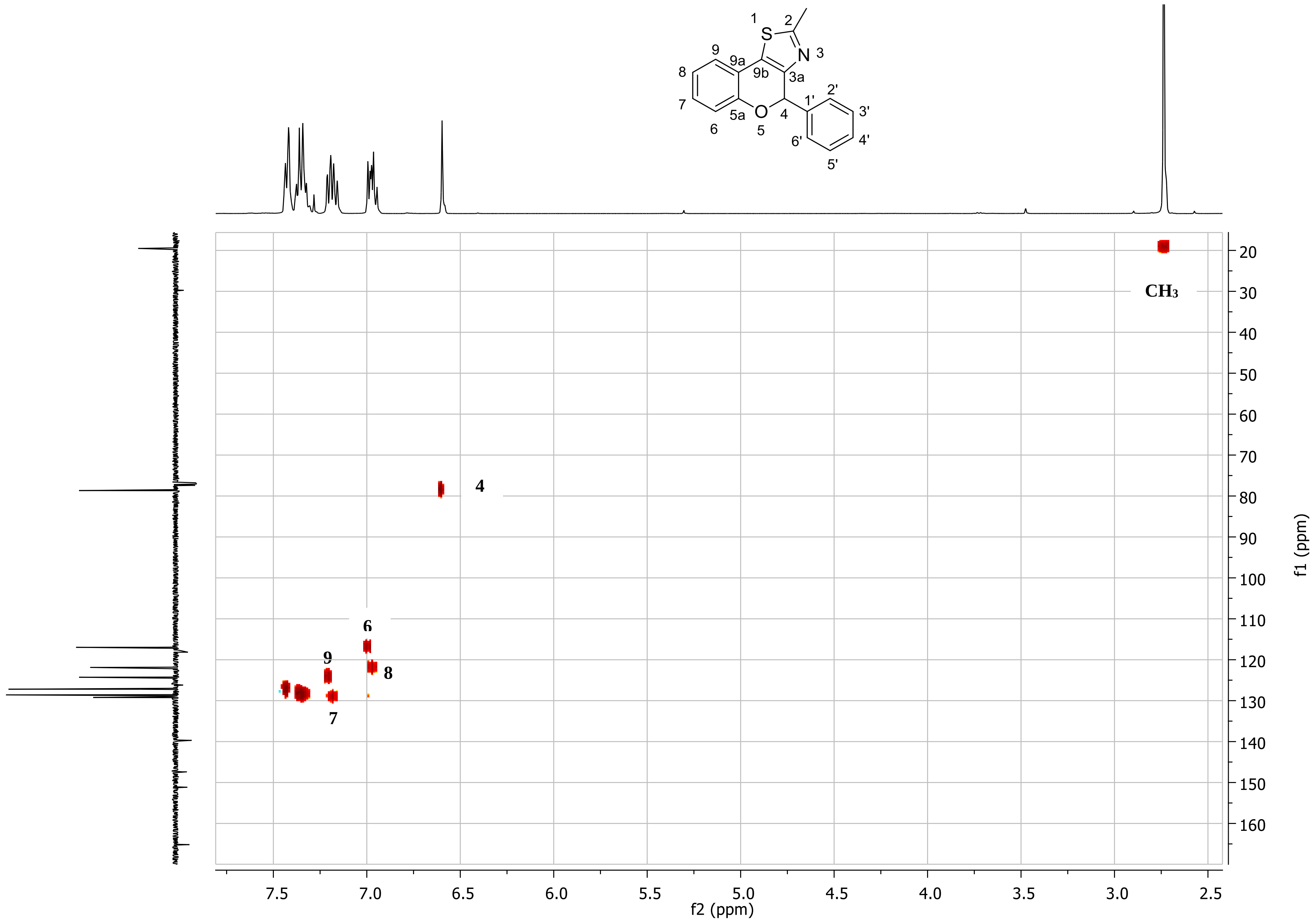

Figure S231. HSQC spectrum of *rac*-**3a** in $CDCl_3$

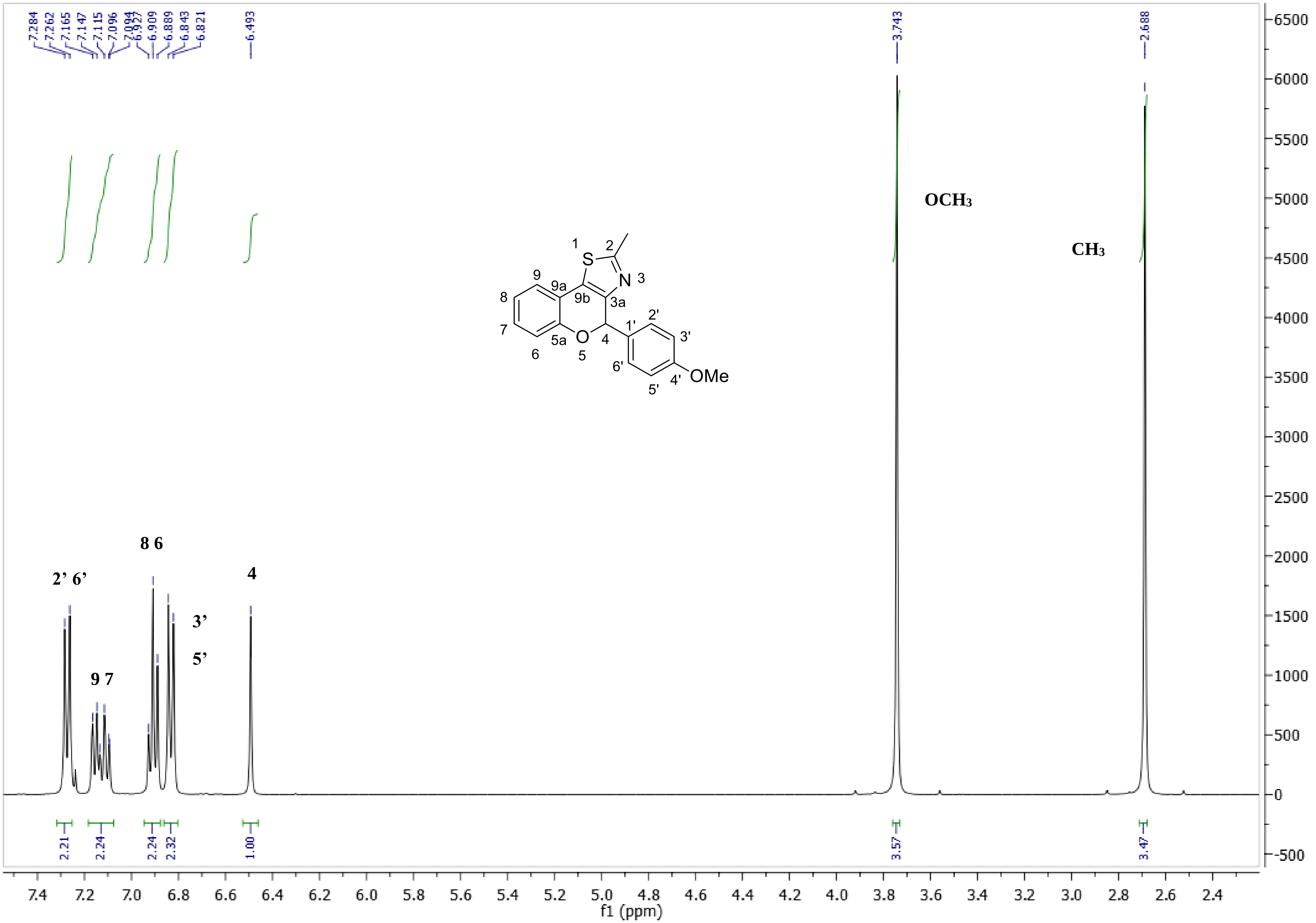

Figure S232. ^{1}H-NMR spectrum of *rac*-**3b** in $CDCl_3$

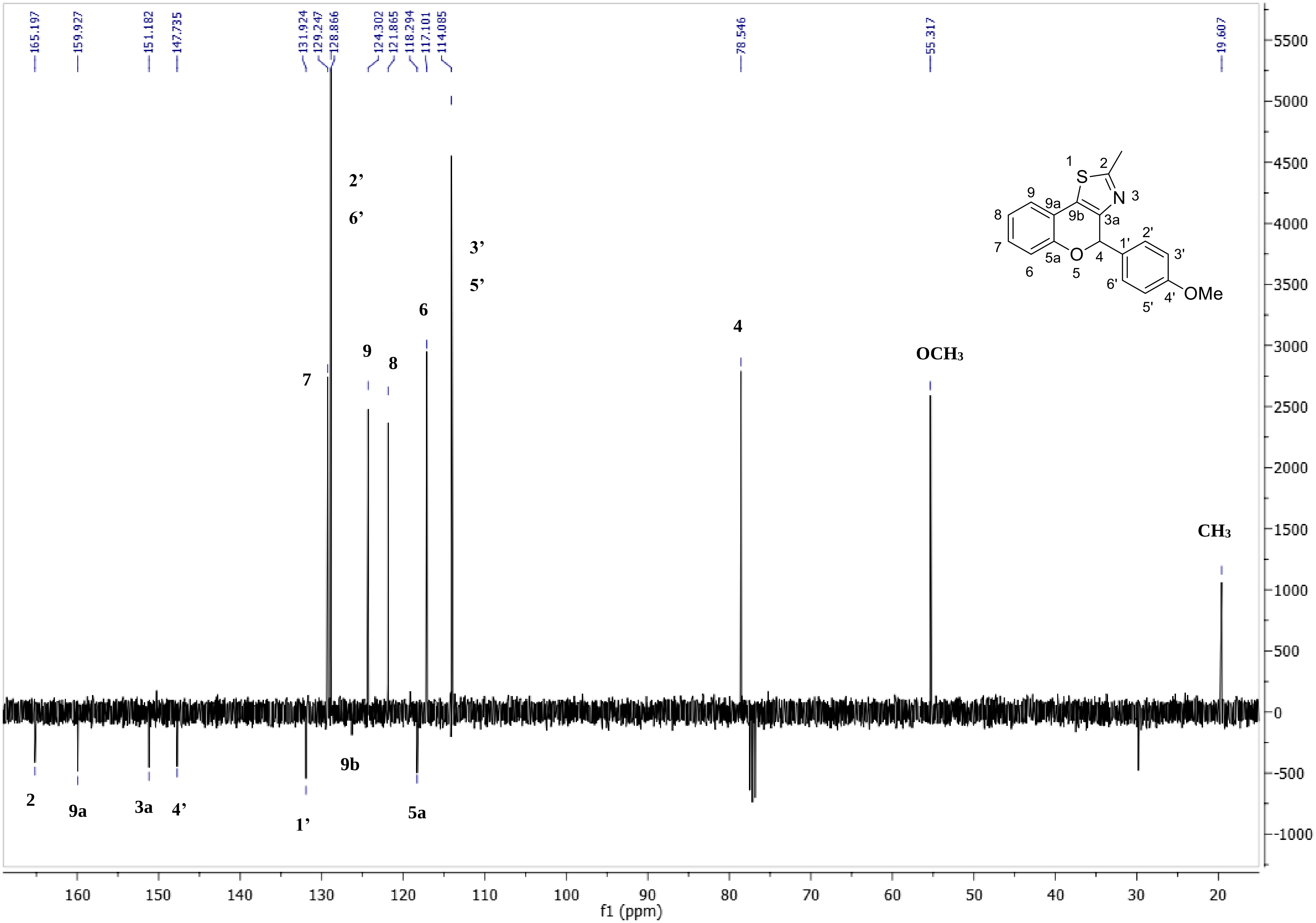

Figure S233. ^{13}C-NMR spectrum of *rac*-**3b** in $CDCl_3$

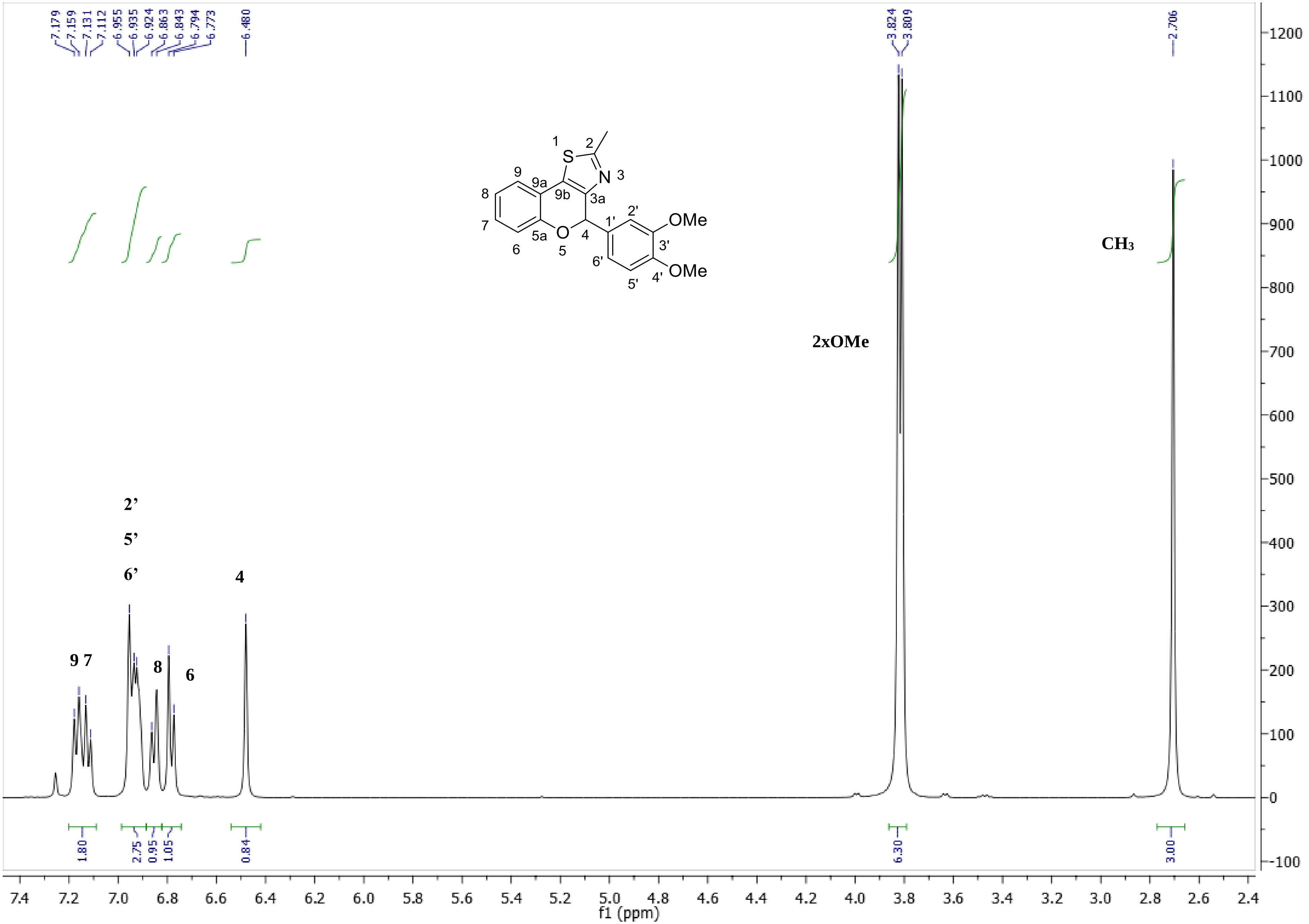

Figure S234. ^{1}H-NMR spectrum of *rac*-**3c** in $CDCl_3$

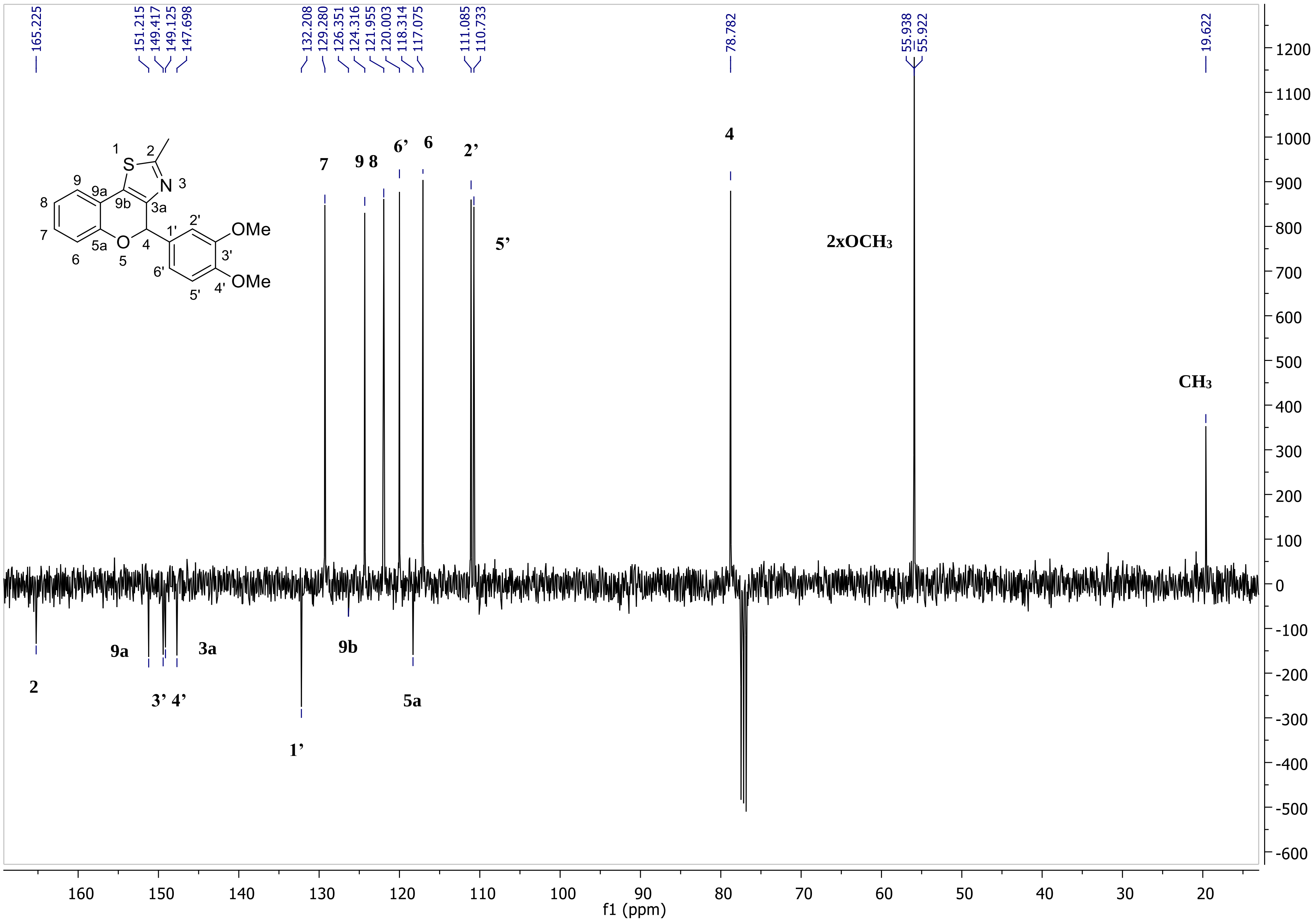

Figure S235. ^{13}C-NMR spectrum of *rac*-**3c** in $CDCl_3$

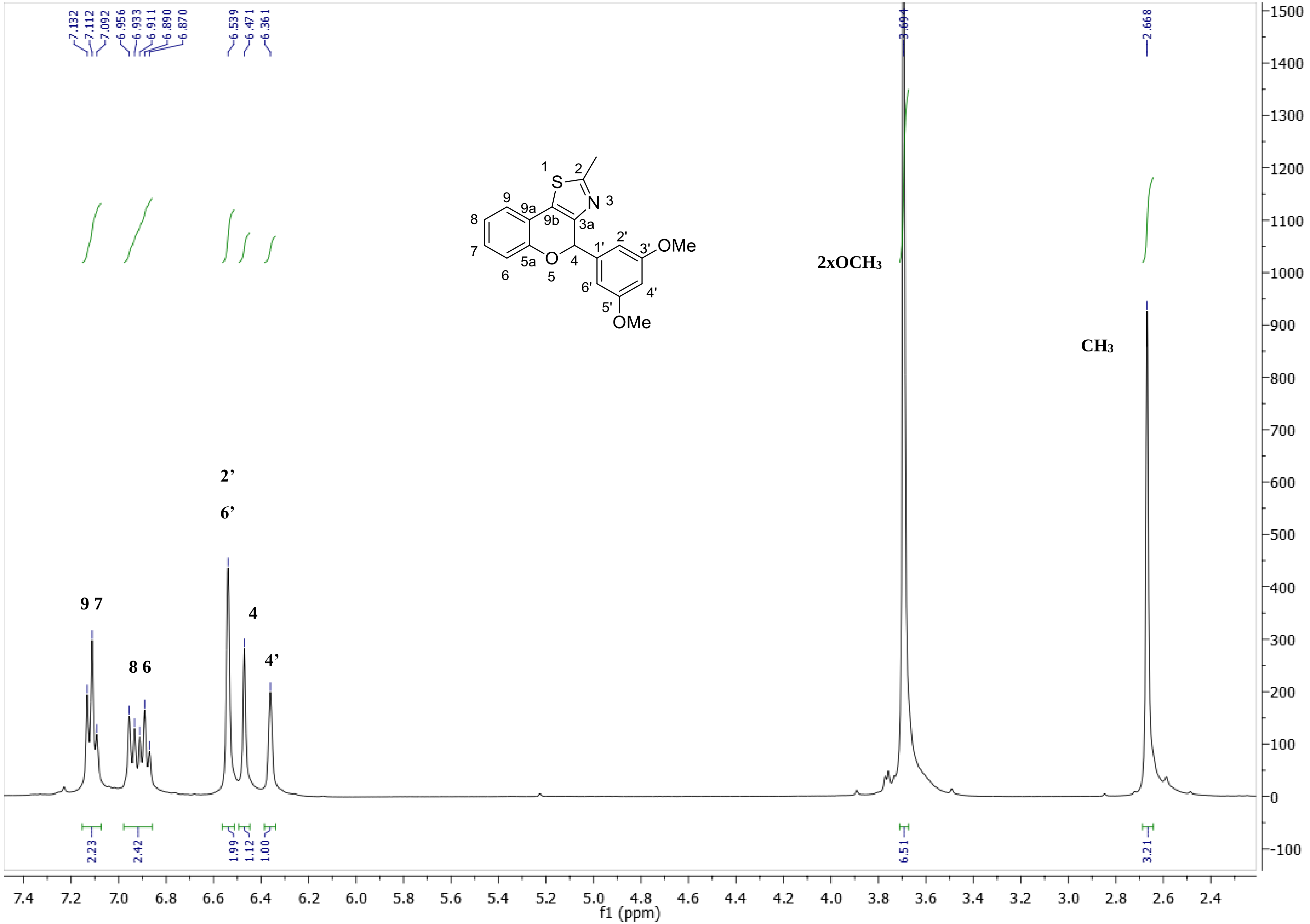

Figure S236. ^{1}H-NMR spectrum of *rac*-**3d** in $CDCl_3$

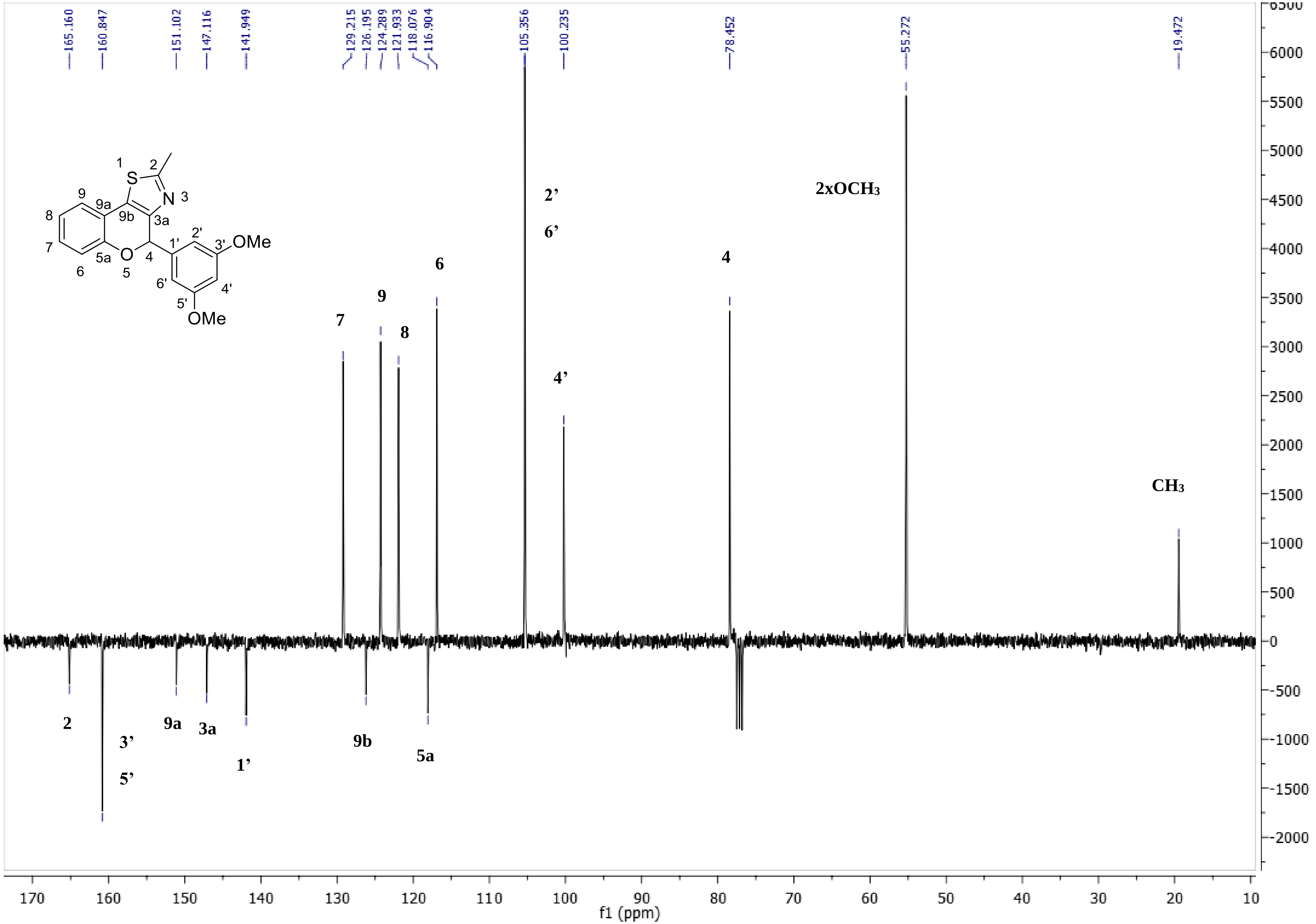

Figure S237. ^{13}C-NMR spectrum of *rac*-**3d** in $CDCl_3$

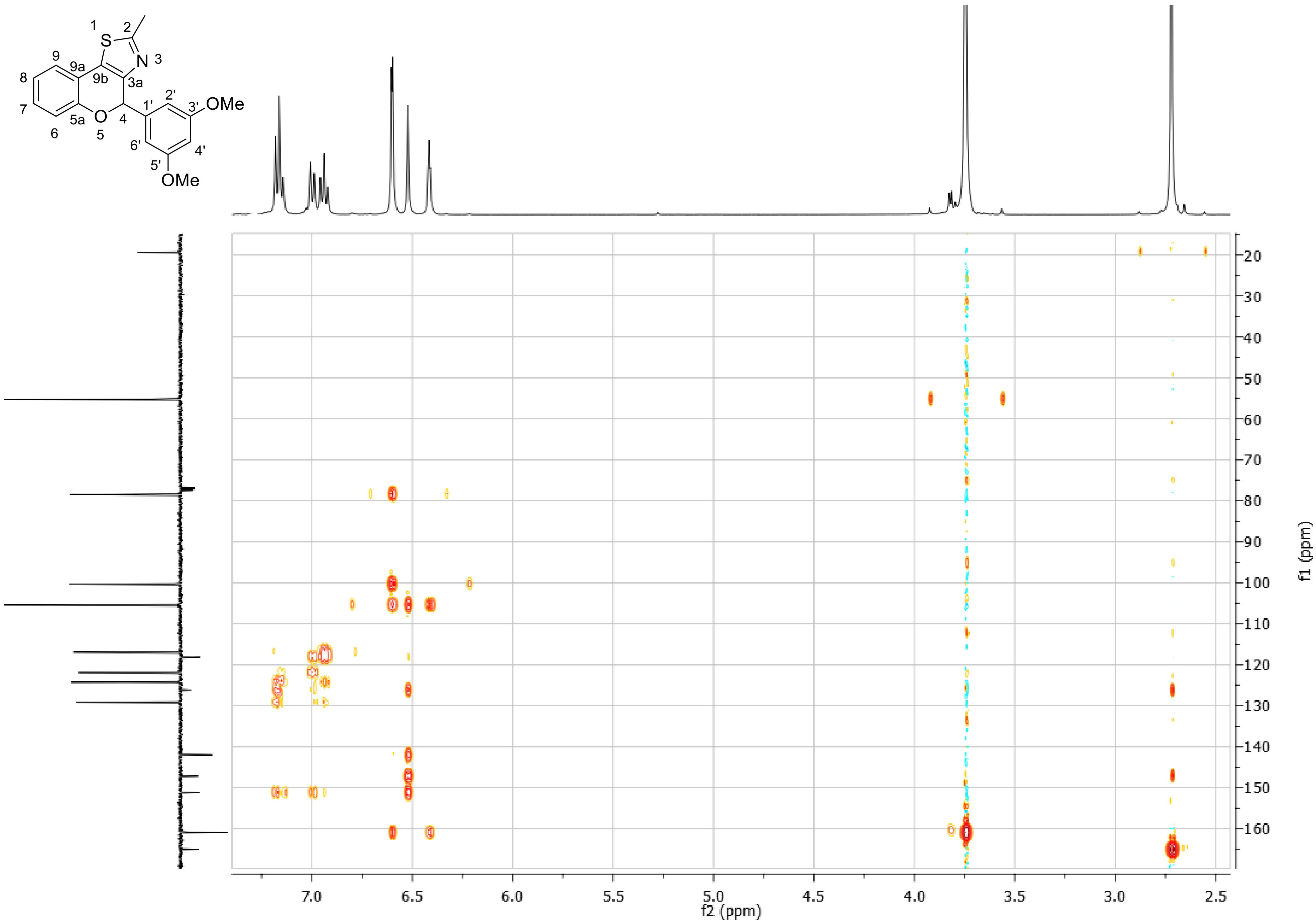

Figure S238. HMBC-spectrum of *rac*-**3d** in $CDCl_3$

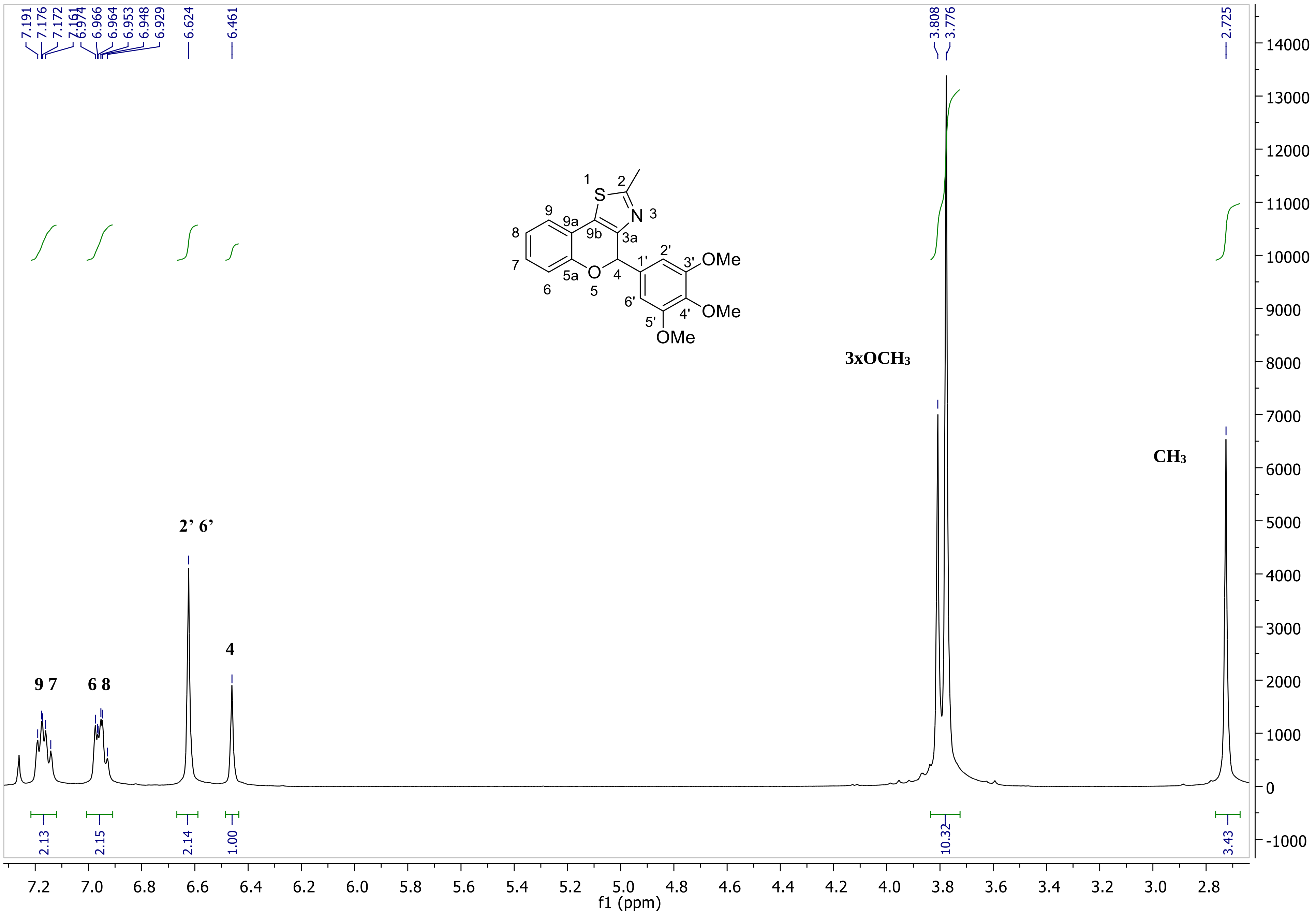

Figure S239. ^{1}H-NMR spectrum of *rac*-**3e** in $CDCl_3$

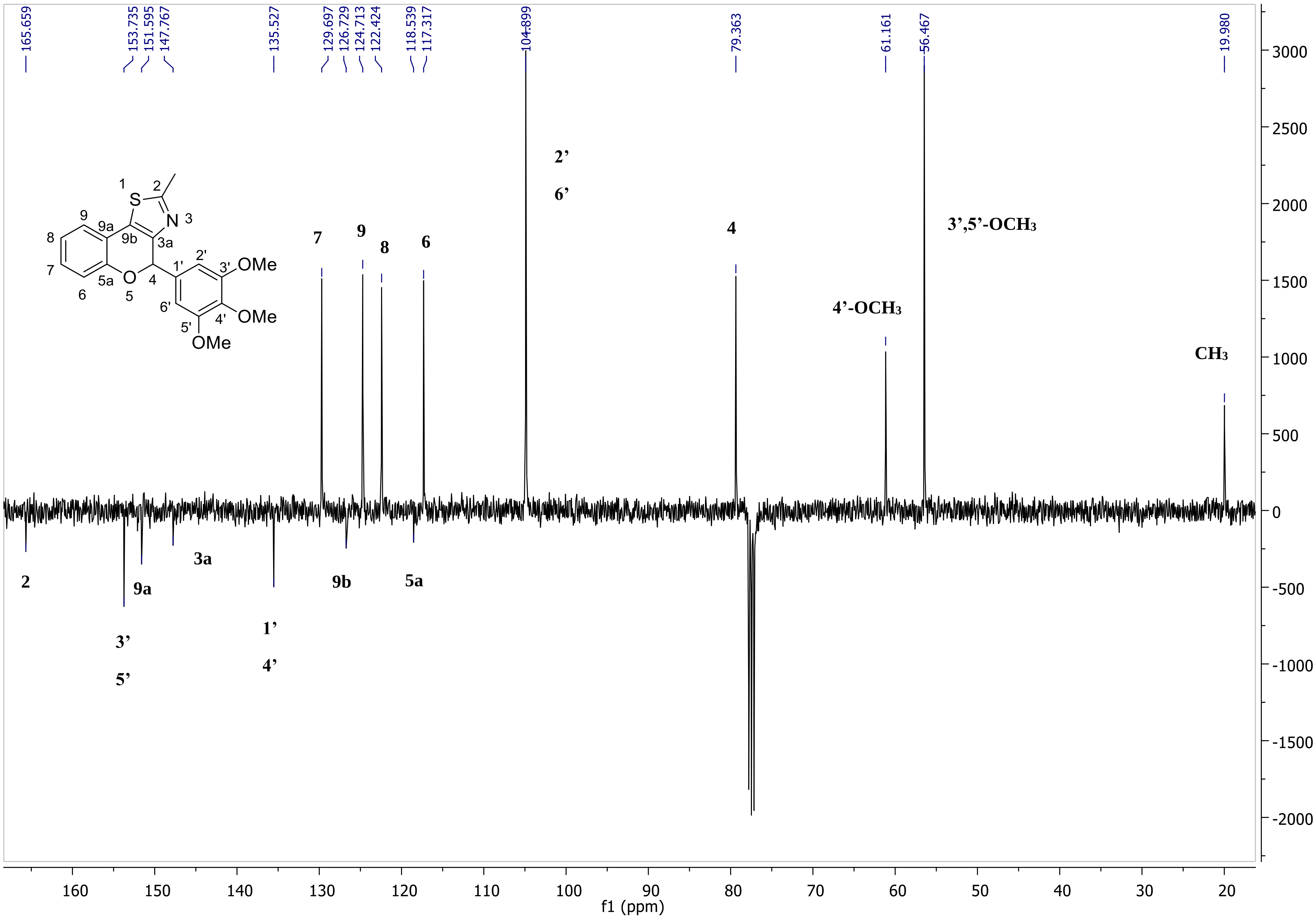

Figure S240. ^{13}C-NMR spectrum of *rac*-**3e** in $CDCl_3$

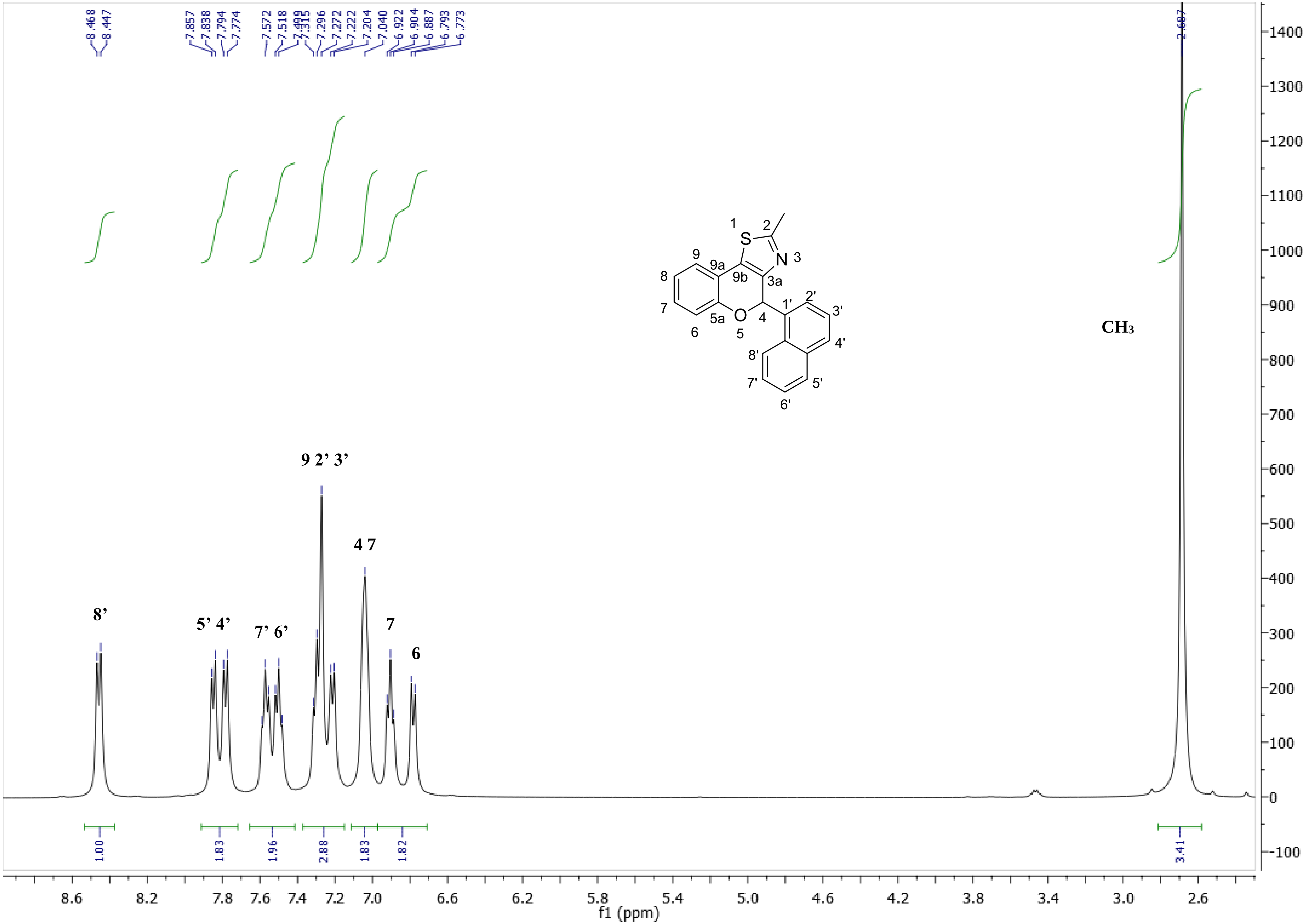

Figure 241. ^{1}H-NMR spectrum of *rac*-**3f** in $CDCl_3$

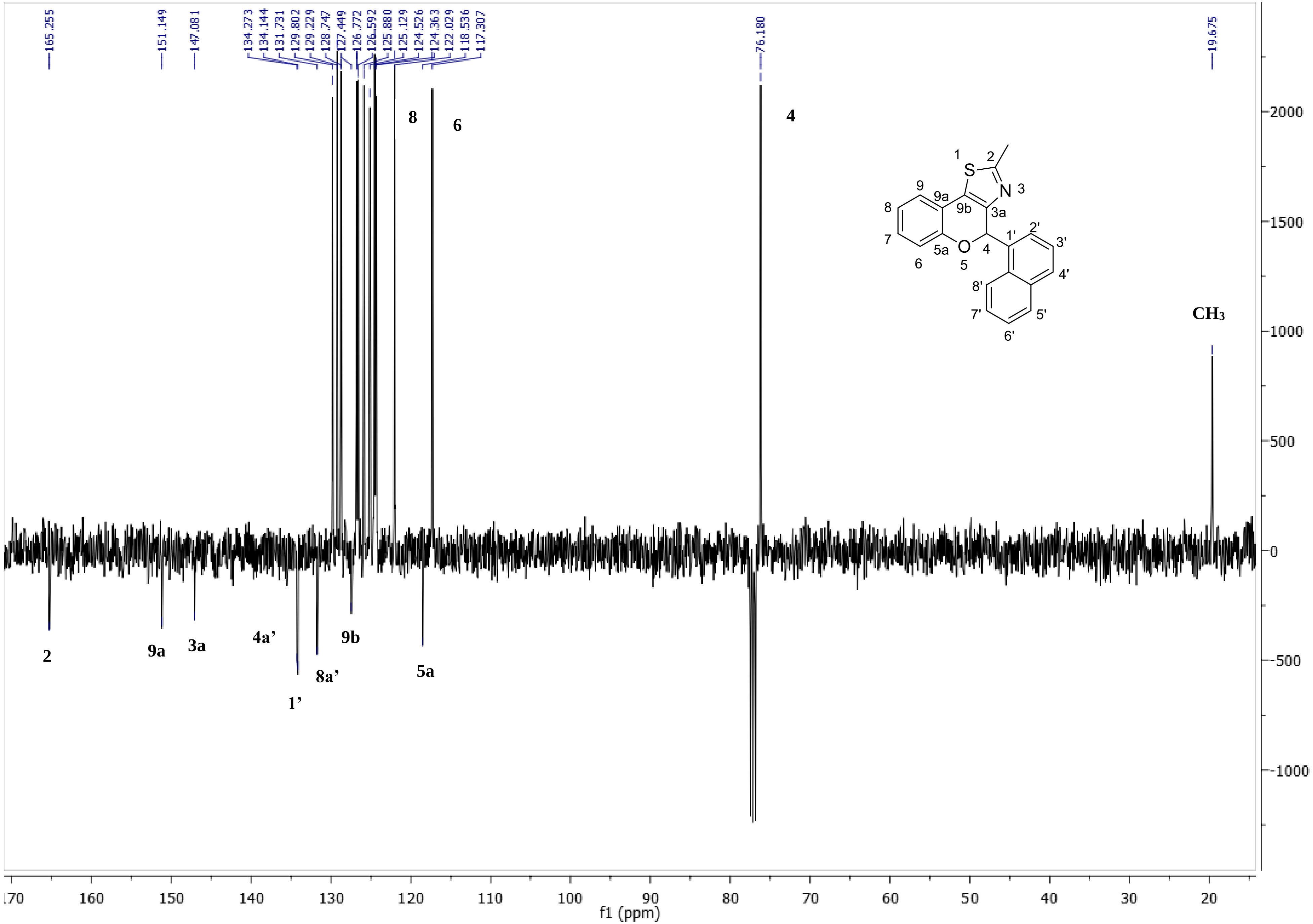

Figure S242. ^{13}C-NMR spectrum of *rac*-**3f** in $CDCl_3$

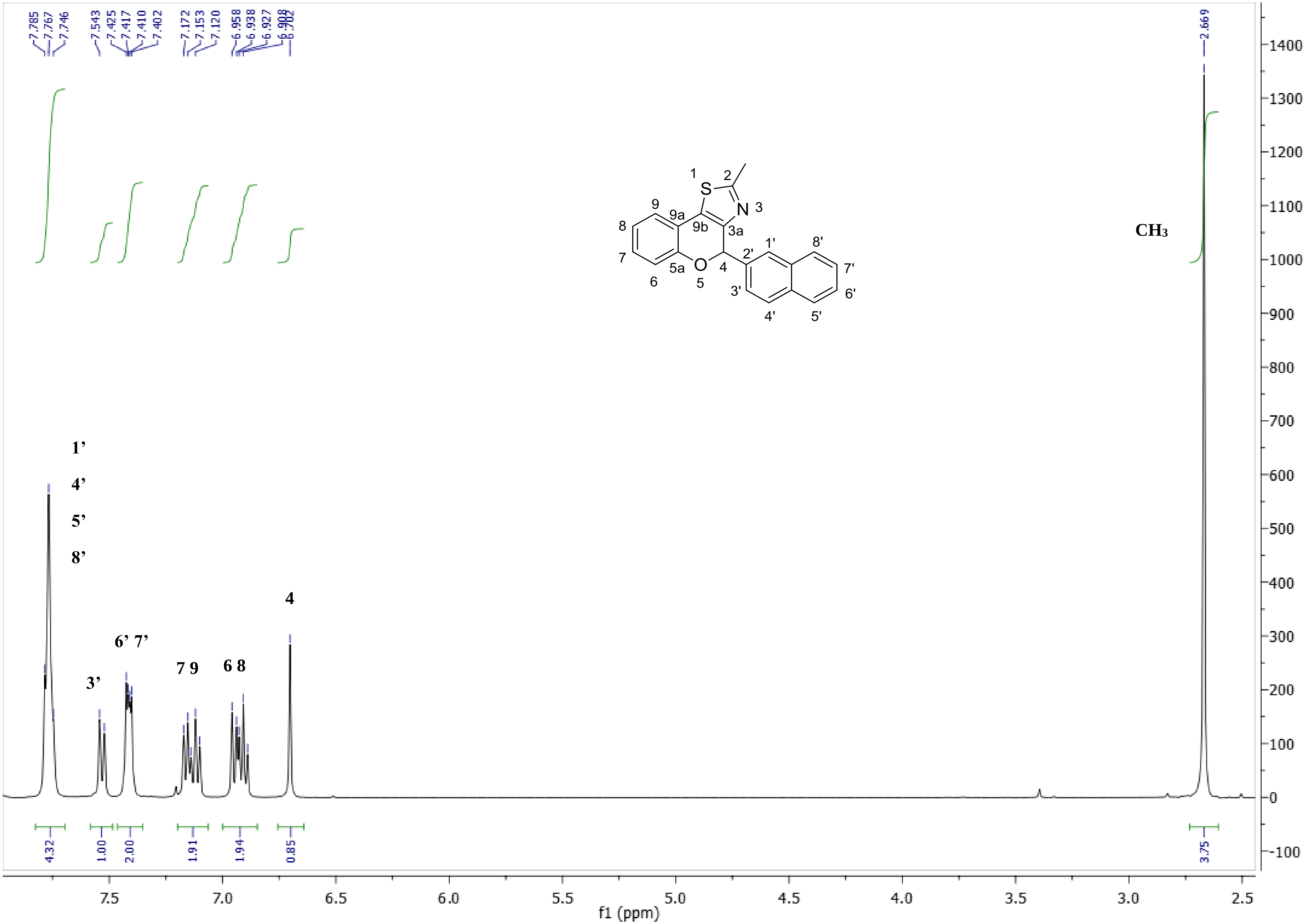

Figure S243. ^{1}H-NMR spectrum of *rac*-**3g** in $CDCl_3$

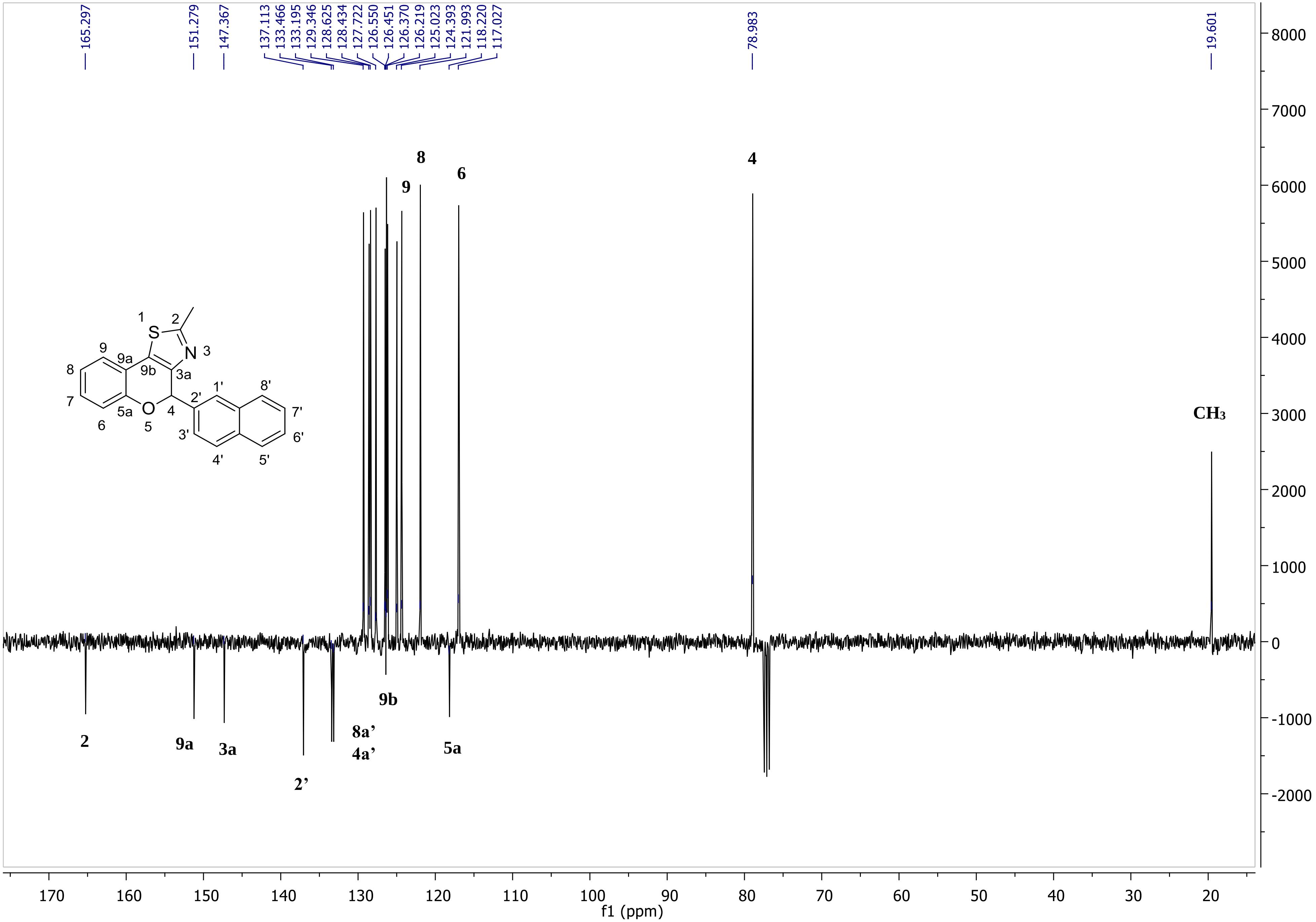

Figure S244. ^{13}C-NMR spectrum of *rac*-**3g** in $CDCl_3$

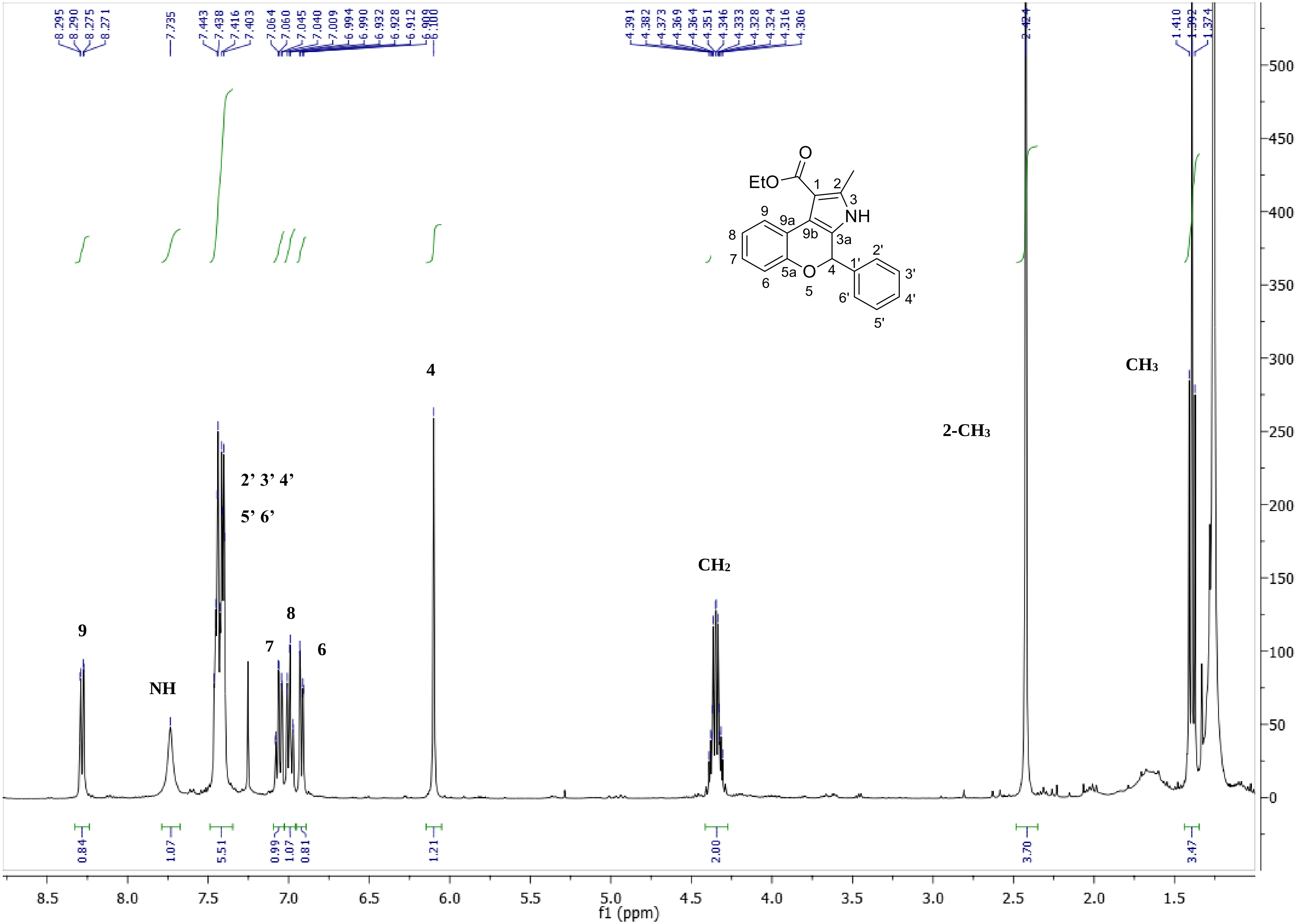

Figure S245. ^{1}H-NMR spectrum of *rac*-**4a** in $CDCl_3$

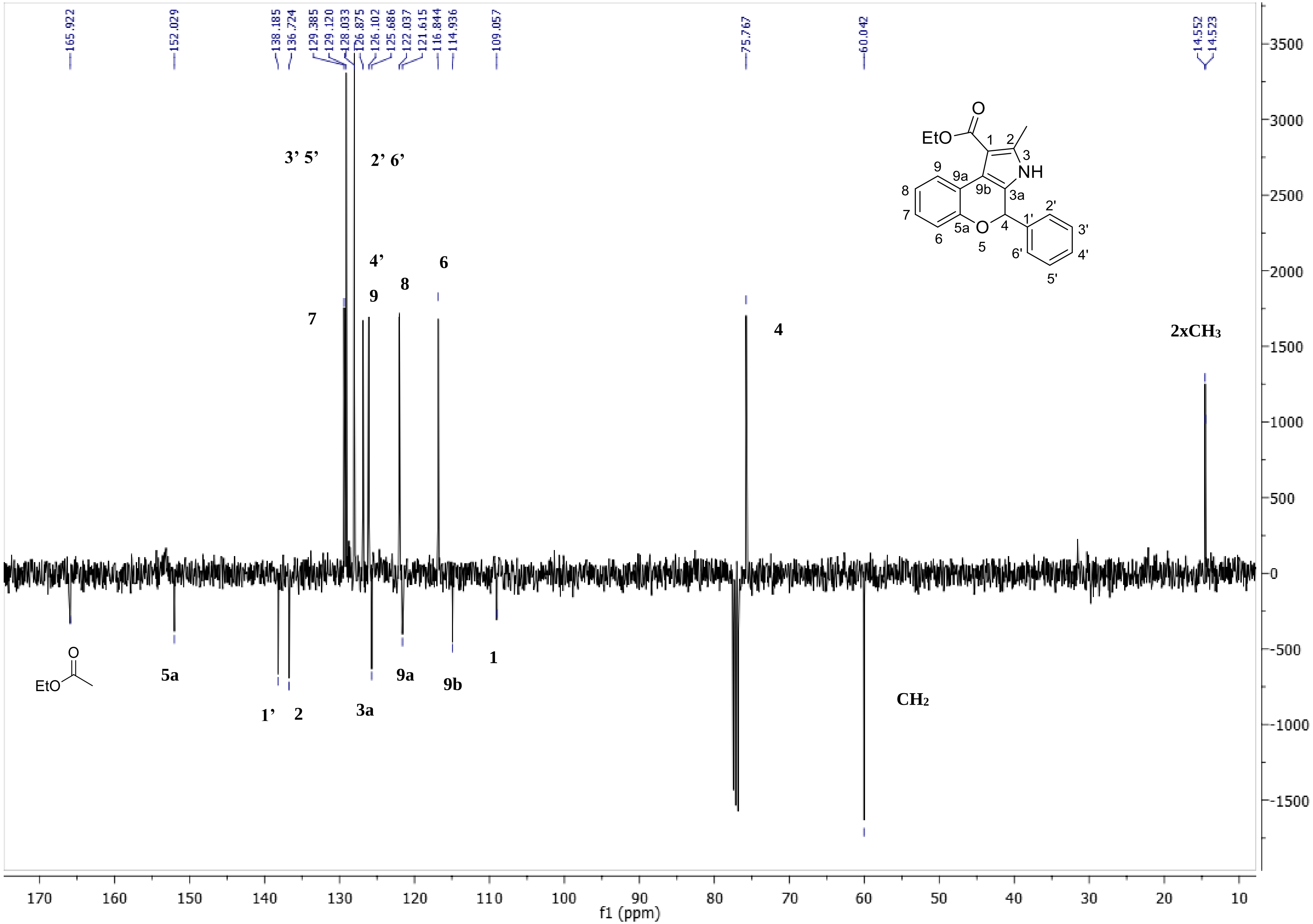

Figure S246. ^{13}C-NMR spectrum of *rac*-**4a** in $CDCl_3$

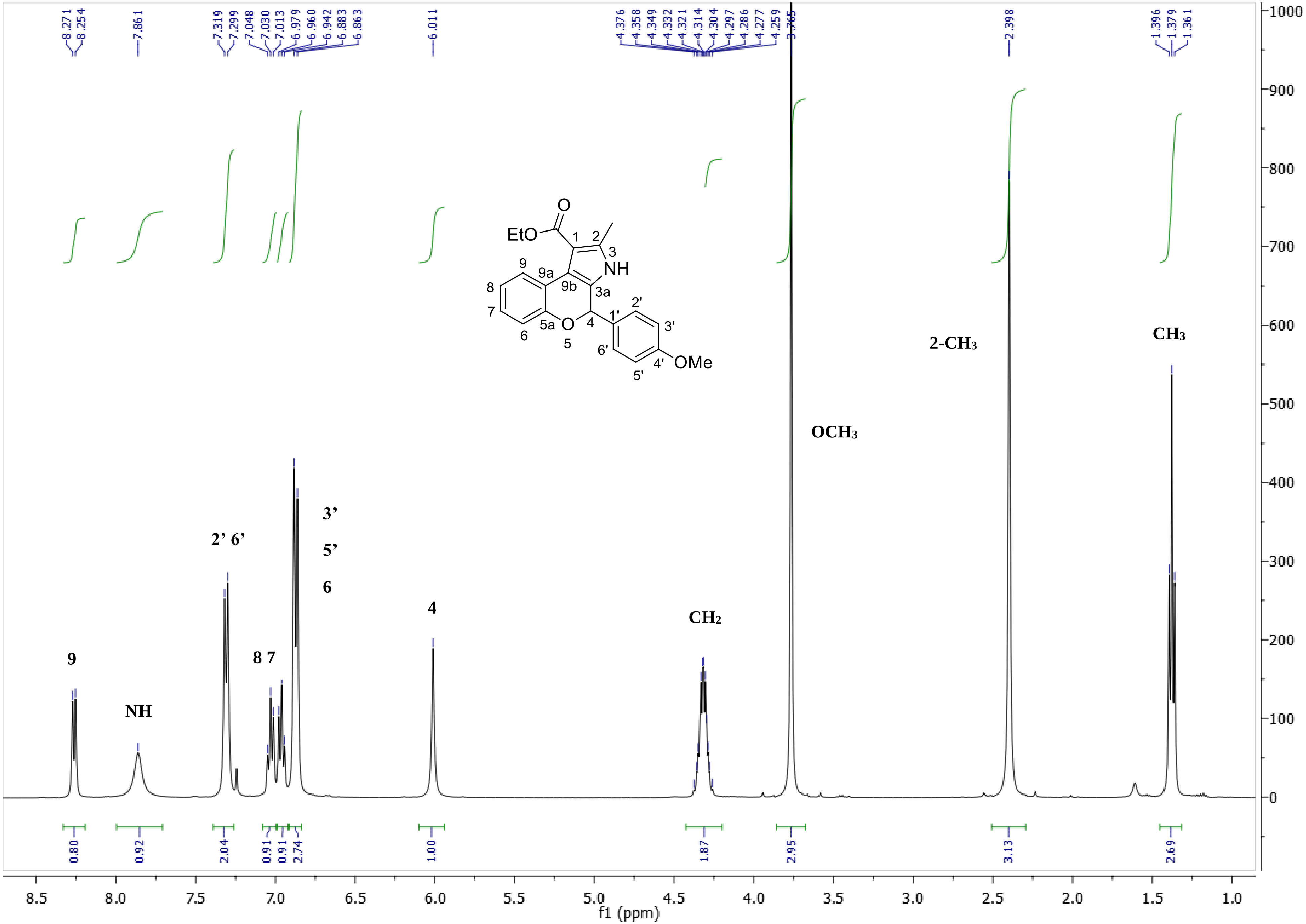

Figure S247. ^{1}H-NMR spectrum of *rac*-**4b** in $CDCl_3$

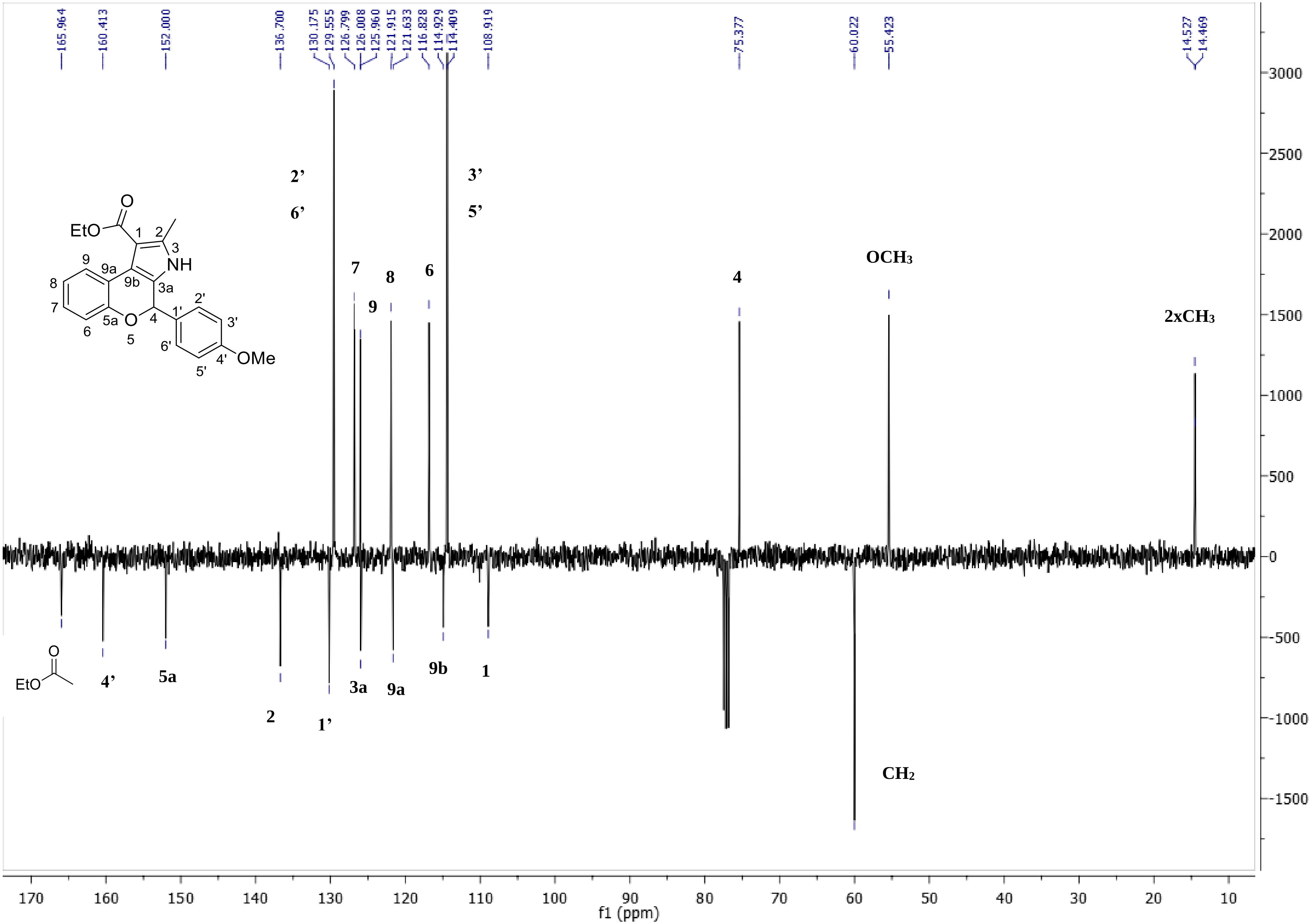

Figure S248. ^{13}C-NMR spectrum of *rac*-**4b** in $CDCl_3$

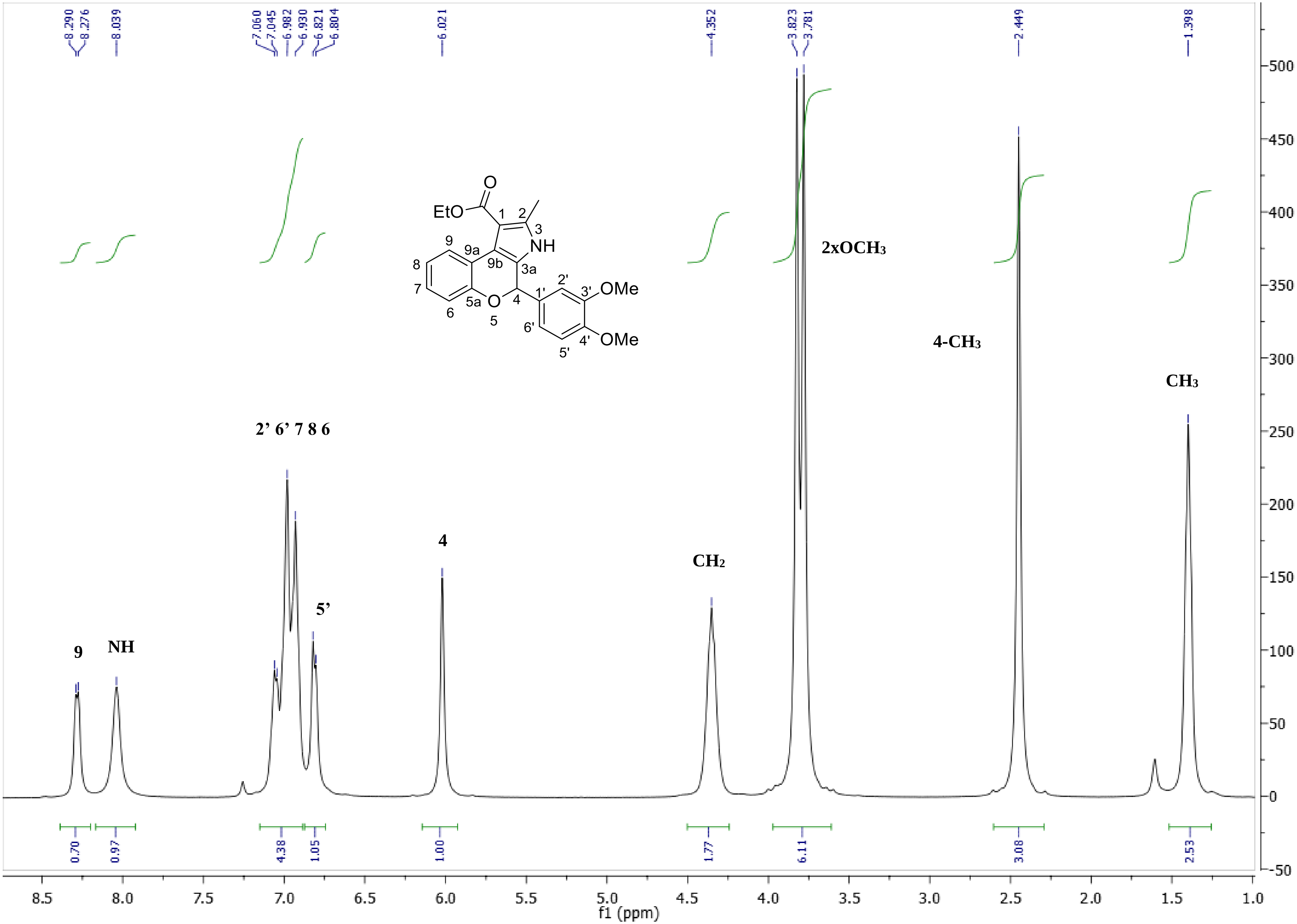

Figure S249. ^{1}H-NMR spectrum of *rac*-**4c** in $CDCl_3$

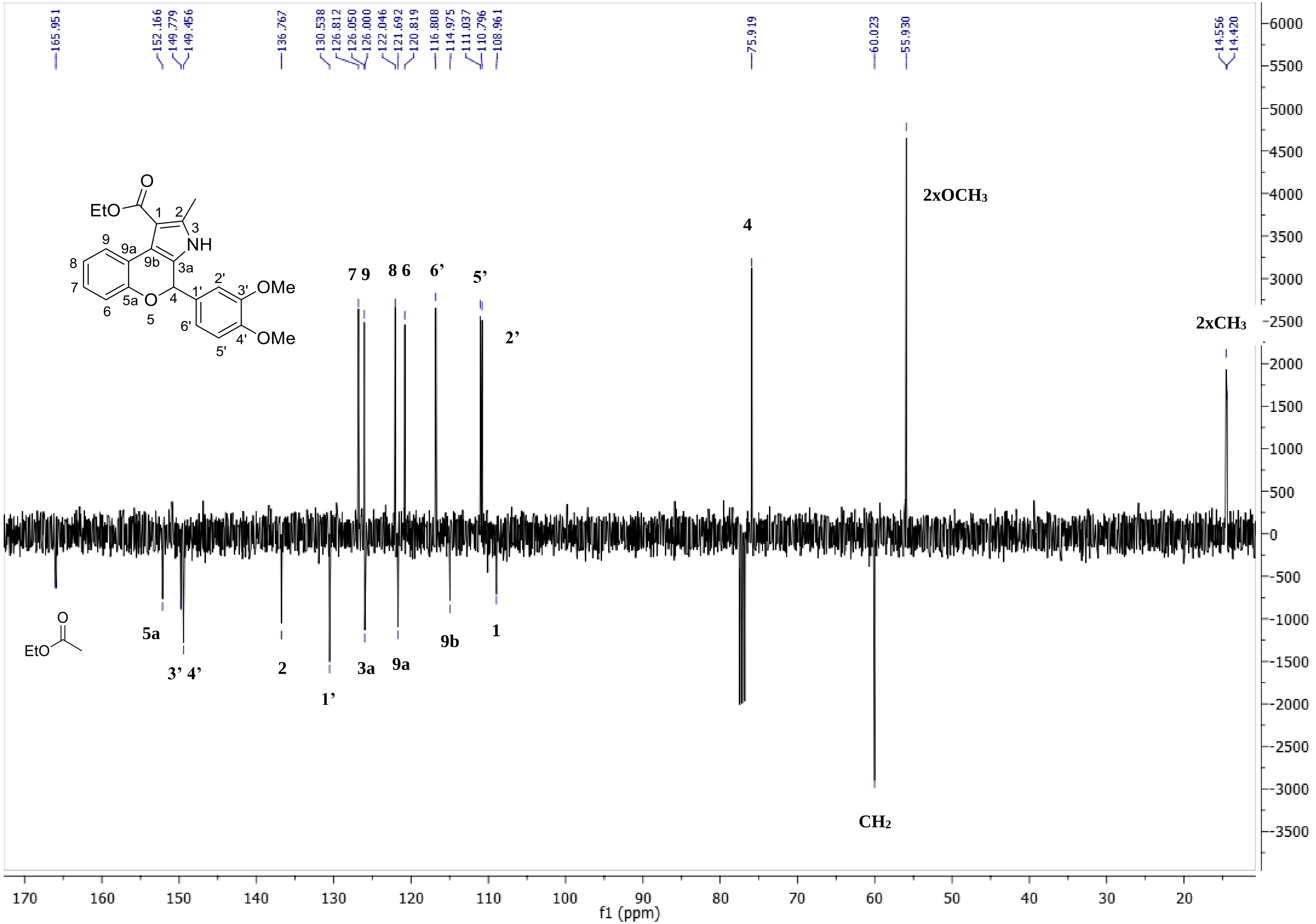

Figure S250. ^{13}C-NMR spectrum of *rac*-**4c** in $CDCl_3$

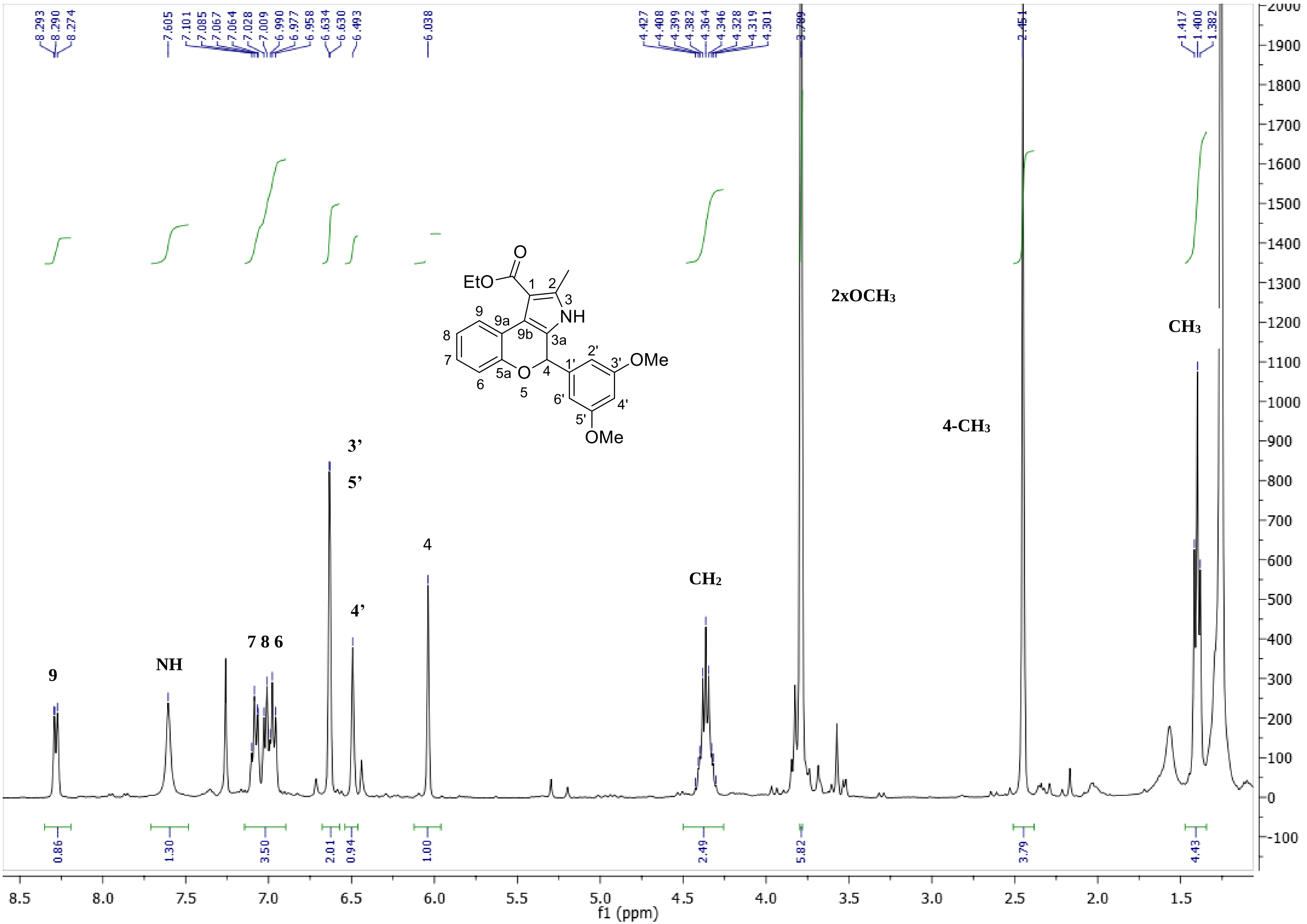

Figure S251. ^{1}H-NMR spectrum of *rac*-**4d** in $CDCl_3$

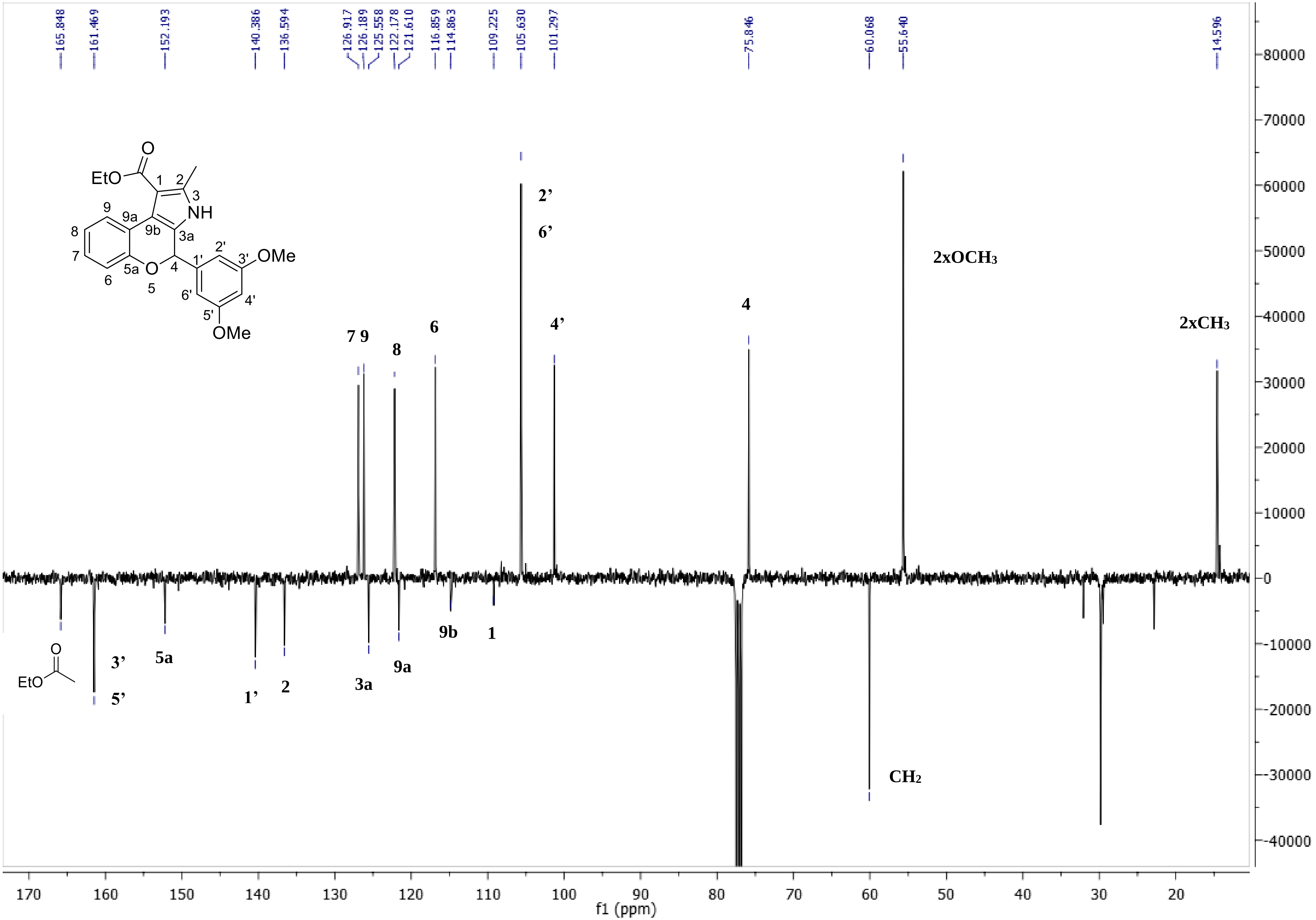

Figure S252. ^{13}C-NMR spectrum of *rac*-**4d** in $CDCl_3$

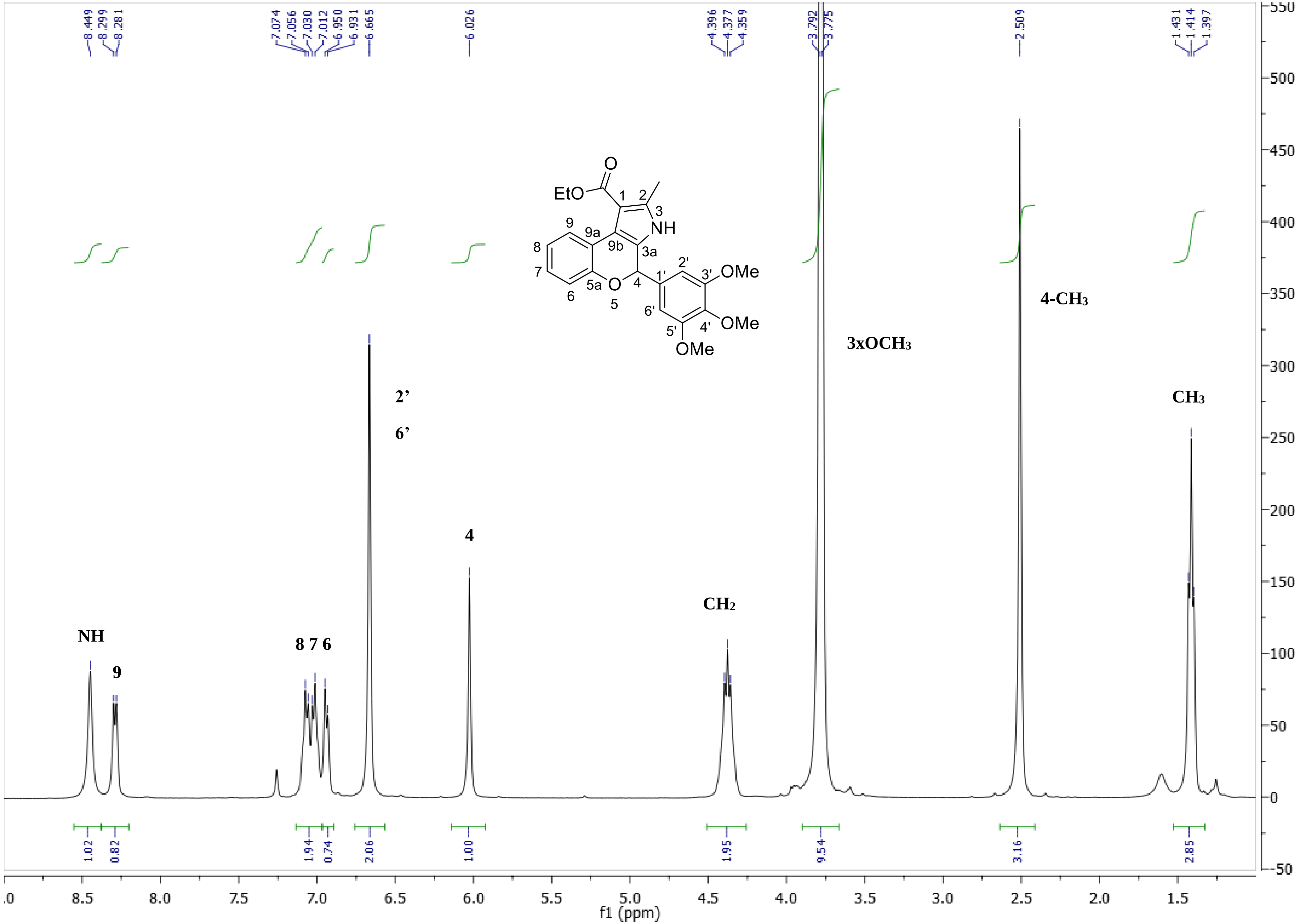

Figure S253. ^{1}H-NMR spectrum of *rac*-**4e** in $CDCl_3$

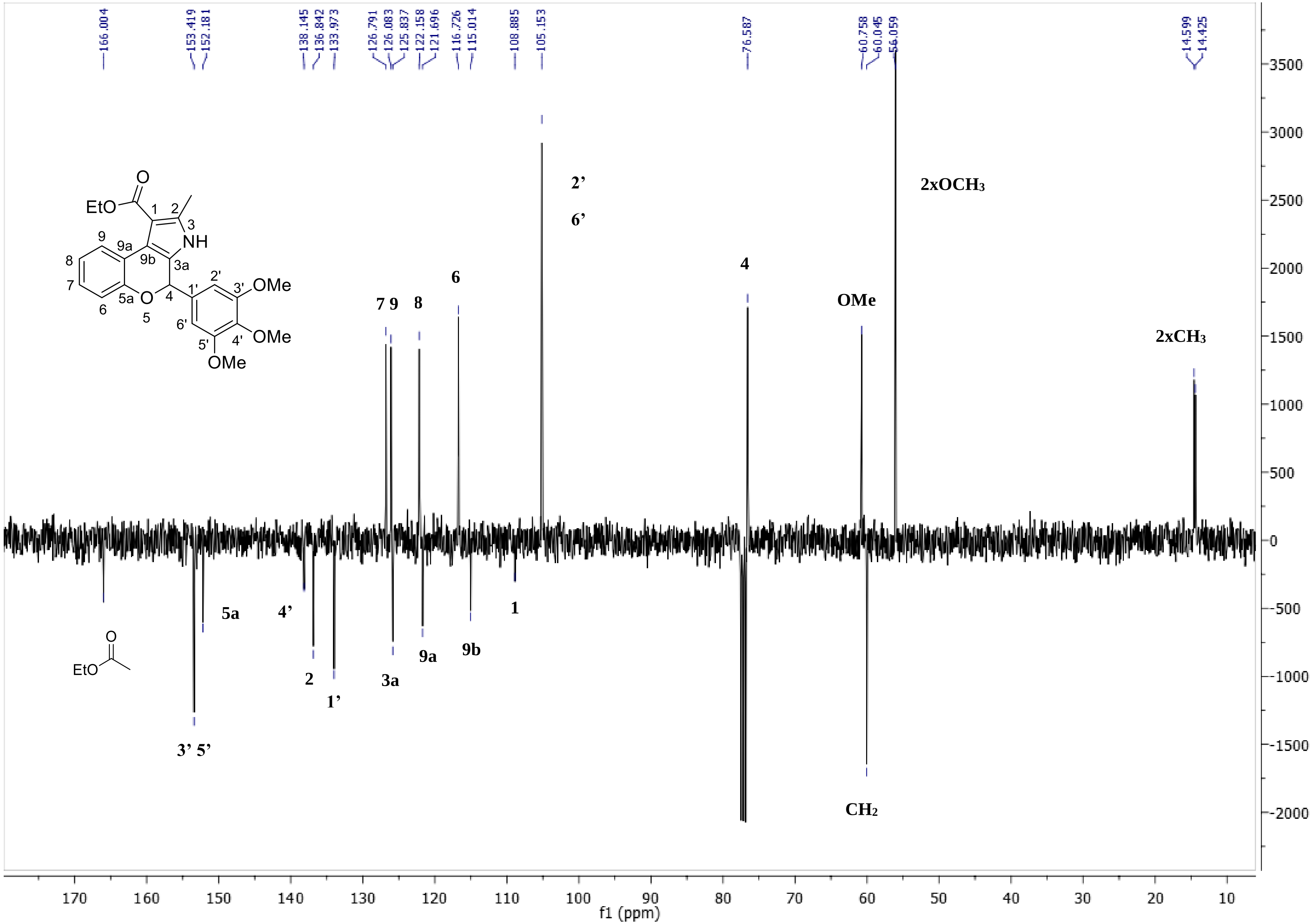

Figure S254. ^{13}C-NMR spectrum of *rac*-**4e** in $CDCl_3$

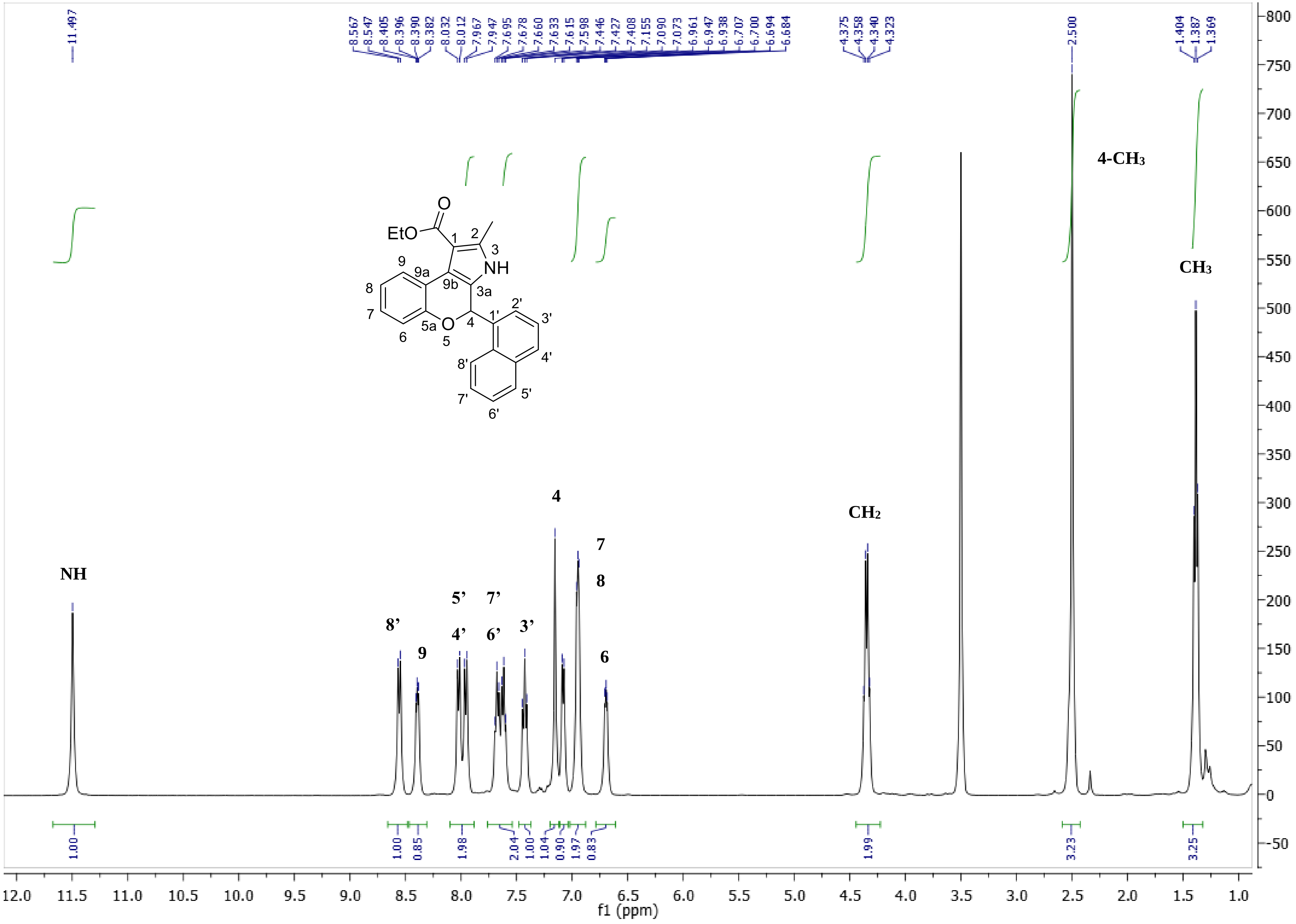

Figure S255. ^{1}H-NMR spectrum of *rac*-**4f** in DMSO-d_6

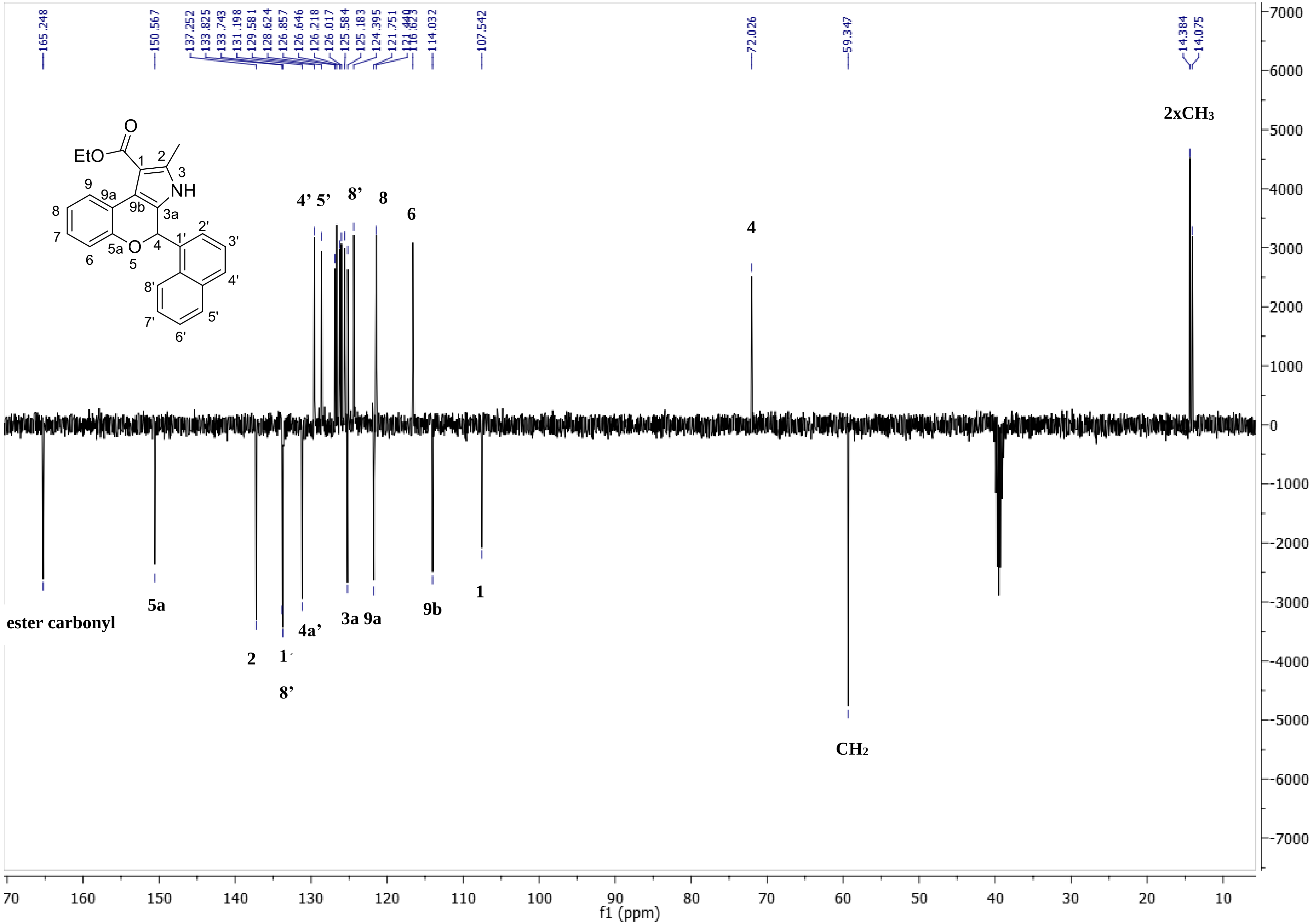

Figure S256. ^{13}C-NMR spectrum of *rac*-**4f** in DMSO-d$_6$

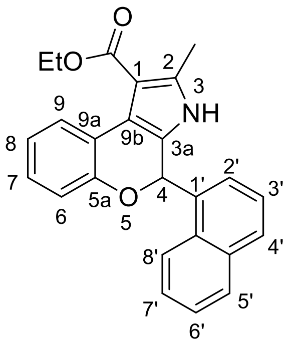

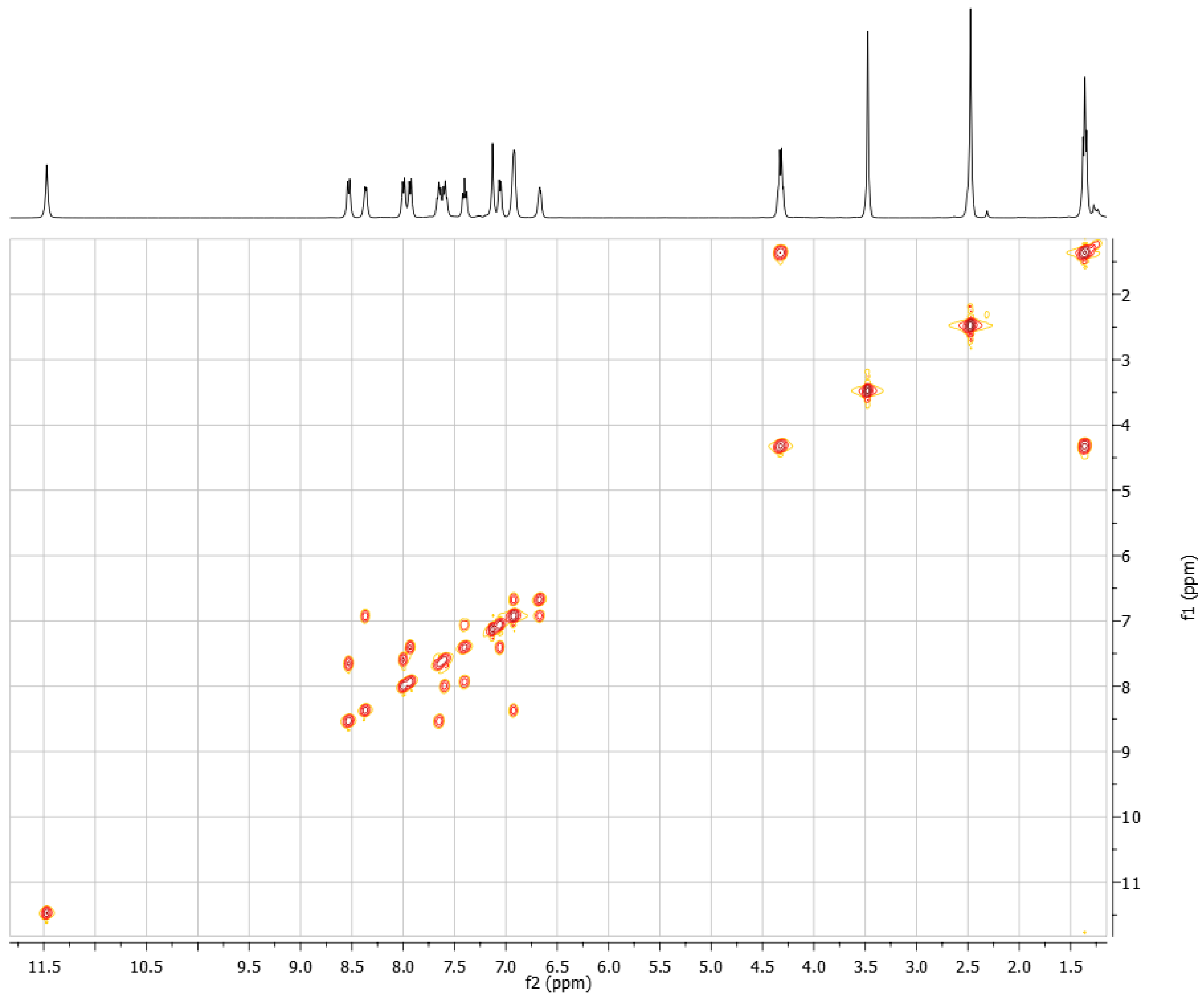

Figure S257. COSY-spectrum of *rac*-**4f** in DMSO-d_6

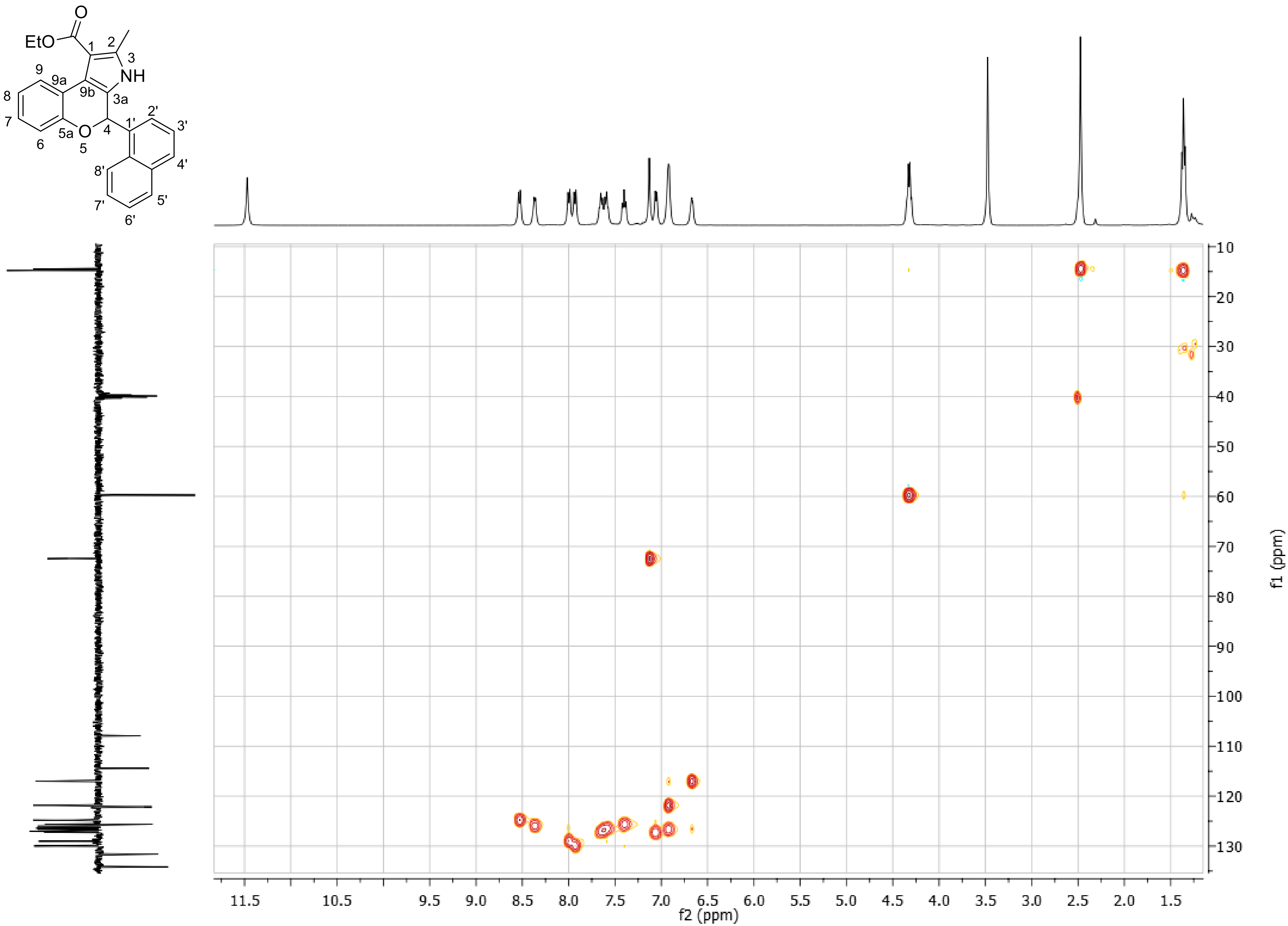

Figure S258. HSQC-spectrum of *rac*-**4f** in DMSO-d$_6$

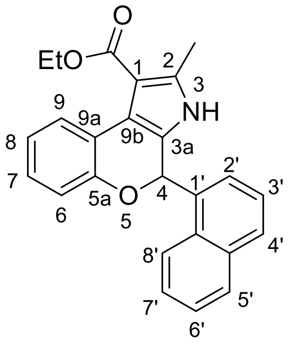

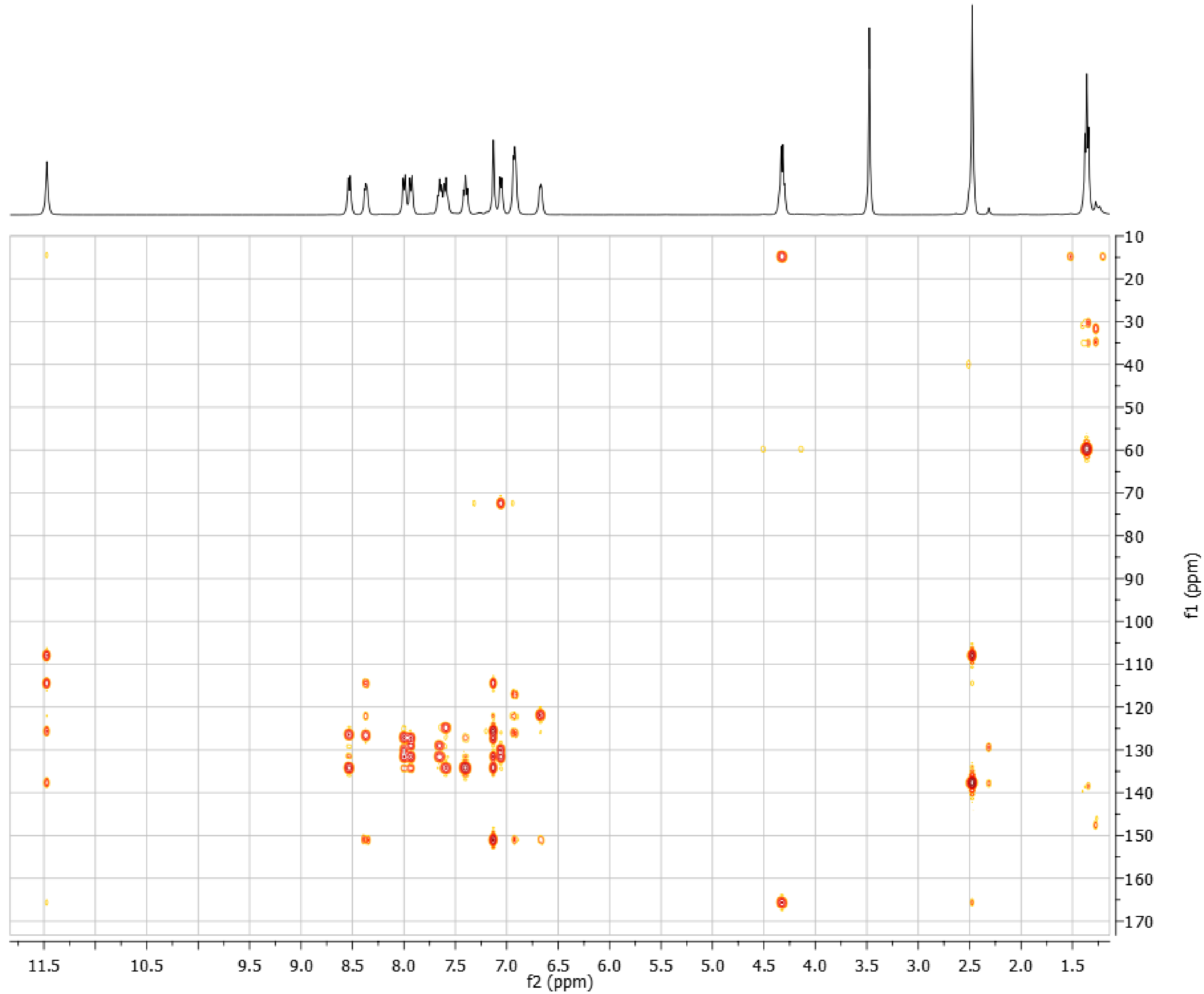

Figure S259. HMBC-spectrum of *rac*-**4f** in DMSO-d_6

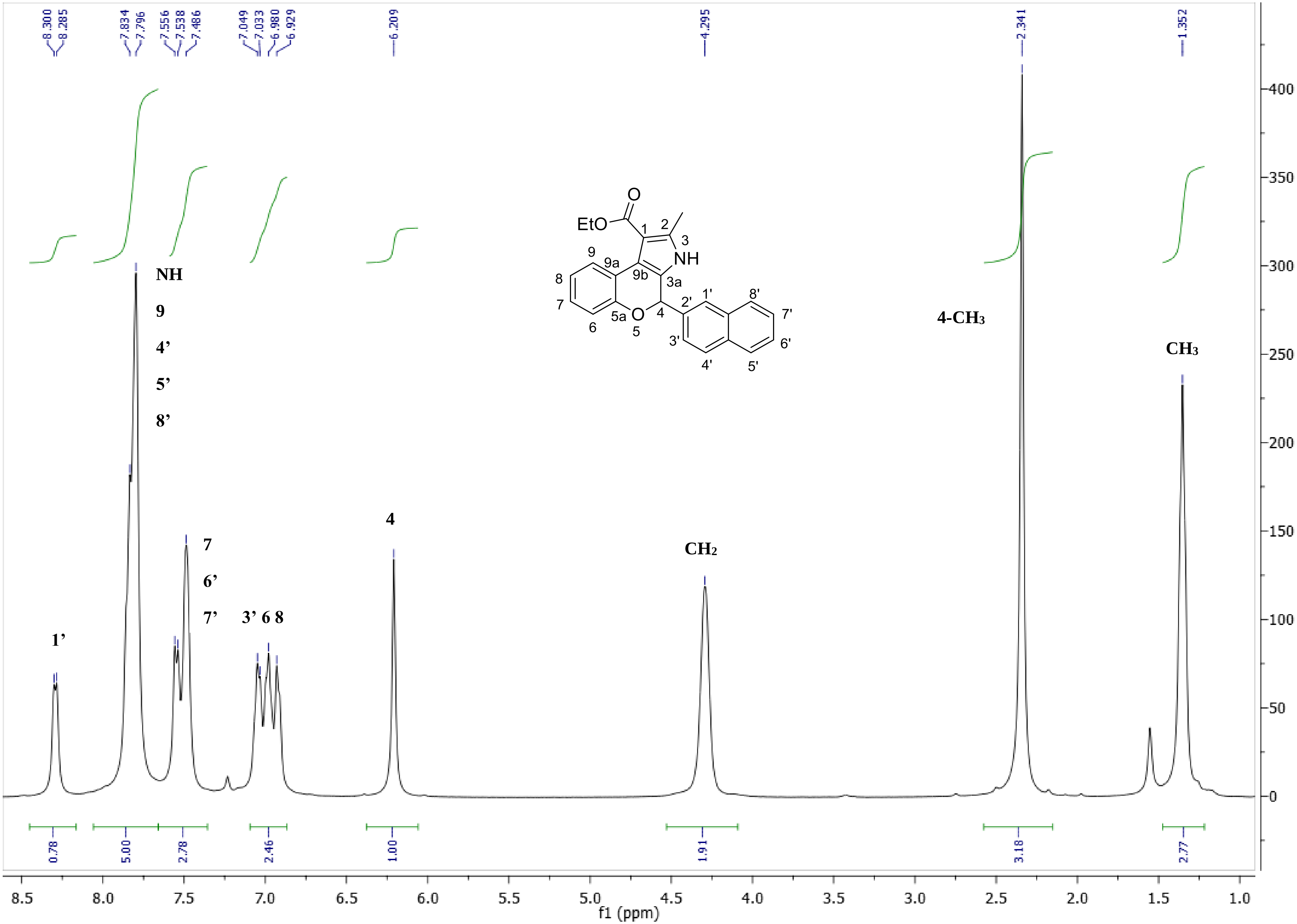

Figure S260. ^{1}H-NMR spectrum of *rac*-**4g** in $CDCl_3$

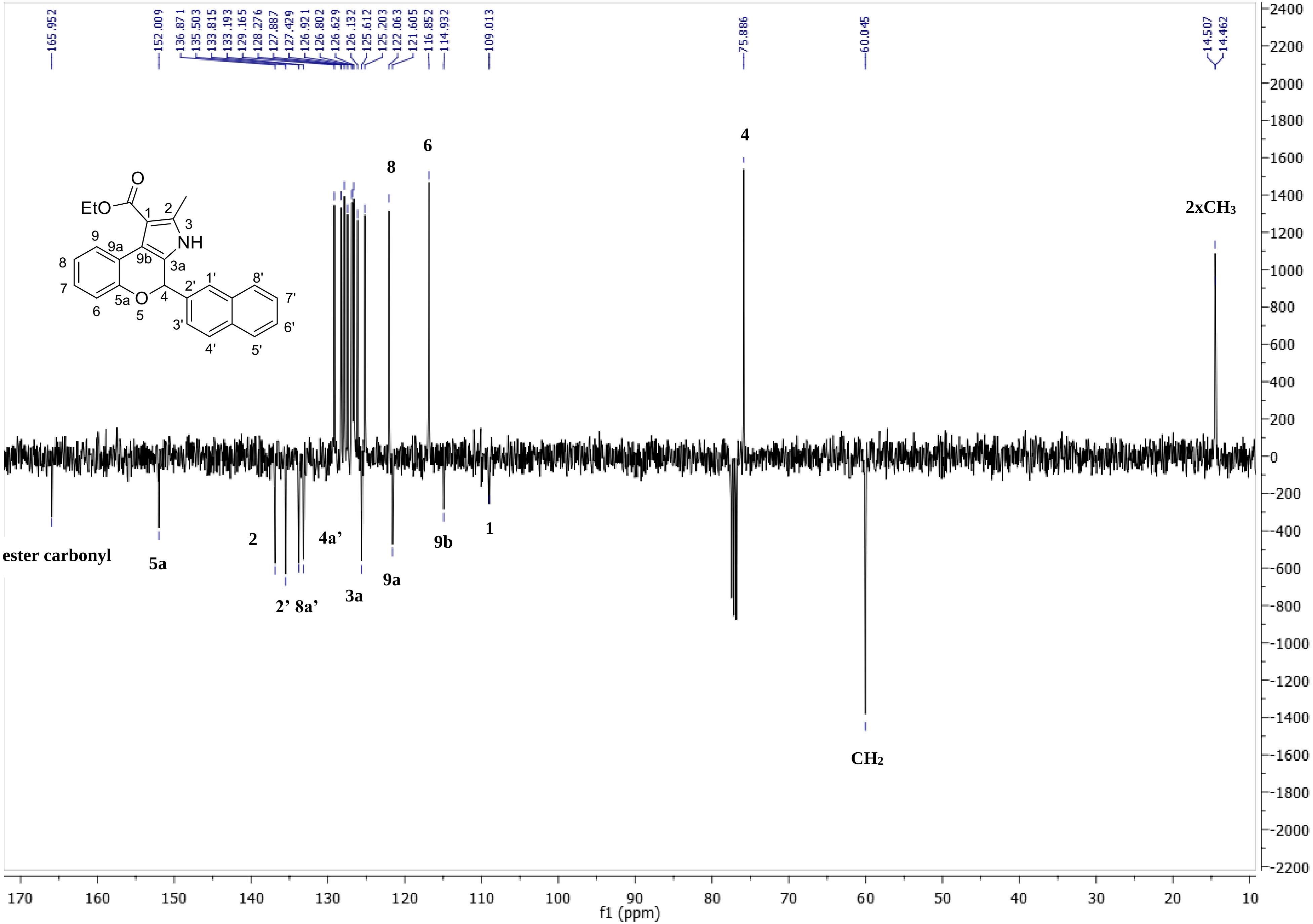

Figure S261. ^{13}C-NMR spectrum of *rac*-**4g** in $CDCl_3$

Table S1. Yields in the preparation of thiazole-condensed derivatives *rac*-**3a-g** from *rac-cis*- and *trans*-**1a-g**

Entry	Substrate	R¹	R²	R³	Yield 1[b] (%)	Yield 2[c] (%)
1	*rac-cis*-**24a** or *rac-trans*-**24a**	H	H	H	*rac*-**3a** (76)	*rac*-**3a** (82)
2	*rac-cis*-**24b** or *rac-trans*-**24b**	H	OMe	H	*rac*-**3b** (44)	*rac*-**3b** (52)
3	*rac-cis*-**24c** or *rac-trans*-**24c**	OMe	OMe	H	*rac*-**3c** (34)	*rac*-**3c** (66)
4	*rac-cis*-**24d** or *rac-trans*-**24d**	OMe	H	OMe	*rac*-**3d** (52)	*rac*-**3d** (70)
5	*rac-cis*-**24e** or *rac-trans*-**24e**	OMe	OMe	OMe	*rac*-**3e** (66)	*rac*-**3e** (84)
6	*rac-trans*-**24f**		1-naphthyl		-	*rac*-**3f** (60)
7	*rac-trans*-**24g**		2-naphthyl		-	*rac*-**3g** (55)

[a] Reaction condition: *rac-cis*-**24a-**e, **g** or *rac-trans*-**24a-g** (0.355 mmol), Lawesson reagent (0.355 mmol) in 5 mL toluene, 4h, 70°C

[b] Isolated yields of product prepared from *rac-cis*-**24a-e**, **g**

[c] Isolated yields of product prepared from *rac-trans*-**24a-g**
